# Using ChatGPT-4 for Lay Summarization in Prostate Cancer Research to Advance Patient-Centered Communication: Large-Scale Generative AI Performance Evaluation

**DOI:** 10.2196/76598

**Published:** 2025-11-19

**Authors:** Emily Rinderknecht, Simon U Engelmann, Veronika Saberi, Clemens Kirschner, Anton P Kravchuk, Anna Schmelzer, Johannes Breyer, Christopher Goßler, Roman Mayr, Christian Gilfrich, Maximilian Burger, Dominik von Winning, Hendrik Borgmann, Christian Wülfing, Axel S Merseburger, Maximilian Haas, Matthias May

**Affiliations:** 1 Working Group on Artificial Intelligence and Digitalization of the German Society of Urology, Germany; 2 Department of Urology, University of Regensburg, Caritas St Josef Medical Center, Landshuter Street 65, Regensburg, 93053, Germany, 49 9417821000; 3 Department of Urology, St. Elisabeth Hospital Straubing, Straubing, Germany; 4 Department of Urology, Nuremberg General Hospital, Paracelsus Medical University, Nuremberg, Germany; 5 Department of Urology, University Hospital Augsburg, Augsburg, Germany; 6 Department of Urology, Faculty of Health Sciences Brandenburg, Brandenburg Medical School Theodor Fontan, Brandenburg, Germany; 7 Department of Urology, Asklepios Klinik Altona, Hamburg, Germany; 8 Department of Urology, University Hospital Schleswig-Holstein, Campus Lübeck, Lübeck, Germany

**Keywords:** health literacy, large language models, prompt engineering, digital health communication, patient engagement, artificial intelligence in publishing, readability assessment, human-AI collaboration, cancer information accessibility, natural language generation

## Abstract

**Background:**

The increasing volume and complexity of biomedical literature pose challenges for making scientific knowledge accessible to lay audiences. Lay summaries, now widely encouraged or required by journals, aim to bridge this gap by promoting health literacy, patient engagement, and public trust. However, many are written by scientists without formal training in plain-language communication, often resulting in limited clarity, readability, and consistency. Generative large language models such as ChatGPT-4 offer a scalable opportunity to support lay summary creation, though their effectiveness within specific clinical domains has not been systematically evaluated at scale.

**Objective:**

This study aimed to assess ChatGPT-4’s performance in generating lay summaries for prostate cancer studies. A secondary objective was to evaluate how prompt design influences summary quality, aiming to provide practical guidance for the use of generative artificial intelligence (AI) in scientific publishing.

**Methods:**

A total of 204 consecutive articles on prostate cancer were extracted from a high-ranking oncology journal mandating lay summaries. Each abstract was processed with ChatGPT-4 using 2 prompts: a simple prompt based on the journal’s guidelines and an extended prompt refined to improve readability. AI-generated and original summaries were evaluated using 3 criteria: readability (Flesch-Kincaid Reading Ease [FKRE]), factual accuracy (5-point Likert scale, blinded rating by 2 clinical experts), and compliance with word count instructions (120‐150 words). Summaries were classified as high-quality as a composite outcome if they met all 3 benchmarks: FKRE >30, accuracy ≥4 from both raters, and word count within range. Statistical comparisons used Wilcoxon signed-rank and paired 2-tailed *t* tests (*P*<.05).

**Results:**

ChatGPT-4-generated lay summaries showed an improvement in readability compared to human-written versions, with the extended prompt achieving higher scores than the simple prompt (median FKRE: extended prompt 47, IQR 42-56; simple prompt 36, IQR 29-43; original 20, IQR 9.5‐29; *P*<.001). Factual accuracy was higher for the AI-generated lay summaries compared to originals (median factual accuracy score: extended prompt 5, IQR 5-5; simple prompt 5, IQR 5-5; original 5, IQR 4-5; *P*<.001) in this dataset. Compliance with word count instructions was greater for both AI-generated summaries in comparison to originals (wrong number of words; extended prompt 39 (19%), simple prompt 40 (20%), original 140 (69%)*; P*<.001). Between simple and extended prompts, there were no significant differences in accuracy (*P*=.53) and word count compliance (*P*=.87). The proportion rated as high-quality was 79.4% for the extended prompt, 54.9% for the simple prompt, and 5.4% for original summaries (*P*<.001).

**Conclusions:**

With optimized prompting, ChatGPT-4 produced lay summaries that, on average, scored higher than author-written versions in readability, factual accuracy, and structural compliance within our dataset. These results support integrating generative AI into editorial workflows to improve science communication for nonexpert audiences. Limitations include focus on a single clinical domain and journal, and absence of layperson evaluation.

## Introduction

In recent years, the inclusion of patient voices in the design, communication, and dissemination of medical research has gained prominence as a central tenet of participatory health care. Meaningful involvement of patients and caregivers is increasingly recognized not only as an ethical imperative but also as a key determinant of research relevance, knowledge translation, and patient empowerment [1-5]. Central to this evolving paradigm is the availability of scientific content in formats that are understandable and accessible to laypersons.

Lay summaries (also referred to as plain language summaries in some publishing contexts) are an increasingly common tool intended to bridge the gap between complex biomedical research and the informational needs of patients and the wider public. In this paper, we use the term lay summary as the preferred descriptor, while acknowledging plain language summary as a recognized synonym. In response to regulatory frameworks [6,7] and patient engagement initiatives, several publishers and institutions have implemented policies requiring authors to provide summaries in language that is free from jargon and suitable for non-specialist audiences [8,9]. The European Union’s Clinical Trials Regulation (EU No 536/2014), for example, explicitly mandates that clinical trial results be made available in a lay-accessible format [6,7].

Despite such mandates, the quality of lay summaries remains variable. Prior studies have identified substantial deficits in readability, coherence, and alignment with health literacy standards [10-14]. Even with detailed guidance, translating complex scientific content into clear, accurate, and engaging language for nonexpert audiences remains a considerable challenge [10-15].

Recent advances in generative artificial intelligence (AI) offer promising avenues for addressing these challenges. Large language models (LLMs), most notably ChatGPT-4, have demonstrated remarkable capabilities in natural language generation, including summarization, paraphrasing, and simplification of complex content [16-21]. Their potential to generate lay-accessible summaries—when appropriately prompted—may alleviate the burden on researchers and improve the consistency and accessibility of scientific communication. Recent scholarship further illustrates the potential of AI-assisted tools in science communication. For example, Markowitz [22] shows that AI can improve the clarity of complex information and positively influence perceptions of science, while Šuto Pavičić et al [23] provide empirical evidence that ChatGPT can enhance plain language summaries of Cochrane oncology reviews.

In the field of oncology, the journal *Cancers* provides a uniquely structured environment for evaluating such technologies. As one of the few journals that consistently requires lay summaries for all accepted papers, it offers a standardized editorial framework against which AI-generated outputs can be compared [8]. In this context, this study aimed to evaluate the performance of ChatGPT-4 in generating lay summaries of prostate cancer research articles, comparing them to human-written counterparts in terms of readability, factual accuracy, and adherence to editorial standards (operationalized as compliance with word count requirements).

## Methods

### Article Selection

This study includes consecutive articles on the topic of prostate cancer published in *Cancers* in 2024. To identify the articles, the PubMed database was searched using the search string:

“Cancers (Basel)”[Journal] AND (“prostate cancer” OR “prostate neoplasm” OR “prostate carcinoma”).

All articles with an EPUB date between January 1, 2024, and December 31, 2024, were included. Articles were excluded if they were not related to prostate cancer, had an EPUB date outside the defined time frame, lacked an abstract, original lay summary, or keywords, or if they were not classified as either original research articles or reviews.

Although this study does not involve clinical implementation, it applies key principles articulated in the Developmental and Exploratory Clinical Investigations of Decision-Support Systems driven by Artificial Intelligence (DECIDE-AI) framework, including transparency, structured prompt design, and methodological rigor, thereby aligning with the early evaluative steps required for responsible, patient-centered AI applications [24]. The DECIDE-AI checklist (Checklist 1) was selected because it specifically addresses the methodological and ethical challenges associated with the early-stage evaluation of AI-driven decision support systems. As a formative assessment of generative AI in the context of patient-facing communication, this study reflects the type of preparatory work envisioned by DECIDE-AI prior to real-world deployment [24].

Article characteristics concerning the affiliation of the corresponding author and type of article (original research vs meta-analysis or review) were extracted from the articles’ metadata. Article classification into the categories diagnostic, therapy, both, or others was conducted manually and independently by 3 experts (ER, MH, and MM); discrepancies were resolved by joint consensus. Similarly, articles were manually classified into basic, clinical, or translational science, based on predefined criteria considering the study’s primary focus, methodology, and translational relevance. Basic science studies investigate molecular, cellular, or genetic mechanisms typically using in vitro or animal models, clinical science studies involve patients or patient-derived data focusing on diagnosis, treatment, or outcomes, and translational science studies bridge both by applying mechanistic insights to patient-oriented investigations such as biomarker validation or early-phase therapeutic studies.

### Development of Standardized Prompts for Data Input Into ChatGPT-4

ChatGPT-4 was selected as the LLM because of its widespread use and in accordance with methodologies applied in previous studies [19]. We created a simple prompt to instruct ChatGPT-4 to create a layperson summary based on the abstract, keywords, and title of the paper, adhering to the guidelines provided by the journals. Subsequently, an extended prompt was developed with the aim of optimizing the lay summary in line with the guidelines outlined in the Good Lay Summary Practice Guidelines [7]. The goal was to ensure that the lay summary was comprehensible to readers with a reading equivalent to sixth grade, without compromising factual accuracy or disregarding the journals’ requirements. The prompts are depicted in Textbox 1. More detailed information on prompt development and refinement is included in Multimedia Appendix 1. Each article was processed using both prompts, with a new ChatGPT-4 session initiated for each input.

Textbox 1. **ChatGPT-4 input prompts for creating a layperson summary. Differences are highlighted in italics.**

**Simple prompt**

Dear ChatGPT-4o,

I kindly request your assistance in crafting a Simple Summary as part of a scientific study. The Simple Summary must adhere to the following guidelines:

It should be written in one paragraph, in layman’s terms, to explain why the research is being suggested, what the authors aim to achieve, and how the findings from this research may impact the research community. Please use as few abbreviations as possible, and do not cite references in the Simple Summary. The Simple Summary must not exceed 150 words.

To provide you with the necessary context for creating this Simple Summary, I will supply you with the study title, a scientifically accurate abstract (not in layman’s terms), and the relevant keywords.

Study title: “…”

Scientifically accurate abstract: “…”

Keywords: “…”

Please note: Summarize this unstructured abstract (simple summary) in lay language, highlighting the study purpose, methods, key findings, and practical importance of these findings for the general public. Additionally, be aware that the Simple Summary must not exceed 150 words, but it should make the most of this limit.

**Extended prompt**

Dear ChatGPT-4o,

I kindly request your assistance in crafting a Simple Summary as part of a scientific study. The Simple Summary must adhere to the following guidelines:

It should be written in one paragraph, in layman’s terms, to explain why the research is being suggested, what the authors aim to achieve, and how the findings from this research may impact the research community. Please use as few abbreviations as possible, and do not cite references in the Simple Summary. The Simple Summary must not exceed 150 words.

*The Simple Summary should be crafted with a focus on maximizing readability, aiming for the highest possible Flesch-Kincaid Reading Ease score.*

To provide you with the necessary context for creating this Simple Summary, I will supply you with the study title, a scientifically accurate abstract (not in layman’s terms), and the relevant keywords.

Study title: “…”

Scientifically accurate abstract: “…”

Keywords: “…”

Please note: Summarize this unstructured abstract (simple summary) in lay language at a 6th grade reading level, highlighting the study purpose, methods, key findings, and practical importance of these findings for the general public. Additionally, be aware that the Simple Summary must not exceed 150 words, but it should make the most of this limit.

### Readability Assessment

Readability indices, grade-level indicators, and text metrics were automatically calculated for the original lay summary, the ChatGPT-4 simple prompt summary, and the ChatGPT-4 extended prompt summary using the Readability Test Tool provided by WebFx (WebFx, Inc) [25] as previously described [14,18,19]. The assessment encompassed multiple validated readability indices, including the Flesch-Kincaid Reading Ease (FKRE), Flesch-Kincaid Grade Level (FKGL), Gunning Fog Score, Simple Measure of Gobbledygook Index, Coleman-Liau Index, and Automated Readability Index. In addition, text metrics were analyzed, comprising the number of sentences, total word count, count and proportion of complex words, average words per sentence, and average syllables per word. The readability assessment was conducted between February 1, 2025, and March 31, 2025.

### Factual Accuracy Assessment

The factual accuracy of the lay summaries was evaluated in a blinded manner by 2 independent raters (JB and MM), both of whom possess sufficient scientific expertise (authors of >100 peer-reviewed scientific articles). The assessment was conducted using a 5-point Likert scale to evaluate the alignment with the abstract and keywords, ranging from 1=very poor to 5=excellent. Table 1 outlines the specific criteria used for the evaluation. Both quality assessments were incorporated into the overall quality assessment of the lay summaries’ performance. For the graphical representation of results, only the factual accuracy ratings from rater 1 were considered. To reduce evaluation bias, all summaries were anonymized prior to review. Evaluators were blinded to both the origin (human vs AI-generated) and the prompt type. The order of presentation was randomized for each reviewer. To ensure transparency, examples of lay summaries—with their corresponding ratings (by rater 1, MM) and explanations for the assigned scores—are provided in Multimedia Appendix 2.

**Table 1.** Description of the 5-point Likert scale used for the evaluation of the factual accuracy of the lay summaries.

Score	Explanation
1=very poor	The lay summary contains significant factual errors and diverges substantially from the scientific abstract. Essential information is missing, which severely compromises its clarity and accuracy.
2=poor	The lay summary has multiple factual inaccuracies and diverges in certain areas from the scientific abstract. Some key information is missing, diminishing its overall effectiveness.
3=acceptable	The lay summary is mostly accurate but contains minor factual inaccuracies or omissions. It generally aligns with the scientific abstract, though some details could be more precise or comprehensive.
4=good	The lay summary is factually accurate and largely consistent with the scientific abstract. Only minor, nonessential information may be missing or slightly simplified.
5=excellent	The lay summary is completely accurate, fully aligns with the scientific abstract, and includes all essential information. It conveys the content clearly and effectively, without omitting any important details.

### Adherence to Journal Instructions Assessment

Adherence to journal instructions was operationalized as compliance with the required summary length of 120‐150 words.

### Overall Quality Assessment

To facilitate an integrative evaluation of lay summary quality, a composite score was introduced that incorporated the 3 primary outcome measures: readability, factual accuracy, and adherence to journal instructions (operationalized solely as compliance with the required summary length). High-quality lay summaries were defined using a composite threshold of FKRE≥30, factual accuracy≥4 (defined by 2 content assessments), and word count between 120 and 150 words. The FKRE cut-off of ≥30 was chosen as a pragmatic boundary informed by the Flesch original classification distinguishing scientific from non-scientific texts and by general health literacy recommendations that patient-directed materials should aim for a sixth- to eighth-grade reading level. While some frameworks suggest FKRE≥40 as a stricter benchmark for lay accessibility, we adopted ≥30 to capture the range of readability levels realistically encountered in oncology communication [26-28].

The factual accuracy threshold of ≥4 was selected to denote minimal deviation from the source text, consistent with prior LLM assessment protocols [19].

The word count range of 120 to 150 words reflected the editorial requirements of *Cancers*, the journal that provided the testbed for this evaluation [8].

If these 3 criteria were not met, a scaling was applied based on the definitions outlined in Table 2. The overall quality assessment represents an exploratory composite measure and was not defined as a primary outcome.

**Table 2.** Overall quality assessment of the lay summaries. Exploratory composite measure integrating the 3 measures: readability, factual accuracy, and correct text length.

Measure	Scaling^a^
Readability	
FKRE^b^<30	1 point
FKRE<20	2 points
Factual accuracy	
One content assessment <4	1 point
Both content assessments <4	2 points
Correct text length	
Text length <120 words	1 point
Text length >150 words	1 point

a Overall quality of the lay summaries: 0 point (high quality), 1-2 points (minor limitations), 3 points (moderate limitation), and 4-5 points (major limitations).

b FKRE: Flesch-Kincaid Reading Ease.

### Statistical Analysis

Statistical analyses were performed using SPSS (version 29.0; IBM Corp). Normality of distribution was assessed using the Shapiro-Wilk test (data available upon request). Descriptive statistics were reported as frequencies or as medians with IQR, as appropriate. To compare the different types of lay summaries (original author-provided summaries vs ChatGPT-4 simple prompt vs ChatGPT-4 extended prompt), paired 2-tailed *t* tests were applied for normally distributed continuous variables, while the Wilcoxon signed-rank test was used for nonnormally distributed or ordinal data. Interrater reliability for factual accuracy ratings was evaluated using the Cohen κ coefficient. Differences between articles from different topic categories (clinical science, basic science, and translational science) were initially assessed using the Kruskal-Wallis test. In cases where significant overall differences were observed, pairwise post hoc comparisons were conducted using the Dunn test with Bonferroni correction. A *P* value <.05 was considered statistically significant. All tests were 2-tailed. Visualizations were generated using R (R Foundation for Statistical Computing).

### Ethical Considerations

All journal content used in this study was exclusively obtained from publicly accessible sources. The use of publicly accessible abstracts for scientific analysis complies with the principles of “fair use” as defined by the US Copyright Act (17 US Code § 107) and the corresponding provisions of the German Copyright Act (UrhG, § 51). All referenced materials have been duly cited and acknowledged in accordance with academic standards (Multimedia Appendix 3). Although the study only involved public data and no human participants, a positive ethical approval was obtained from the Ethics Committee of the University of Regensburg (UKR-EK-24-3835-104). In our study setting, obtaining informed consent was not required. The use of ChatGPT-4 was subject to internal governance procedures, including documentation of prompt engineering and blinded human evaluation to mitigate bias. All expert raters involved in this study were transparently identified, including their academic qualifications, institutional affiliations, and roles within the project. Ethical aspects concerning the use of generative AI in medical and scientific communication were carefully considered. Potential limitations, risks, and implications related to AI-assisted content generation were addressed where relevant and are discussed in detail in the respective sections of the paper. All prompts and outputs were archived locally in structured, version-controlled Microsoft Excel files that were accessible only to the research team, thereby safeguarding integrity and enabling retrospective auditing. The complete set of prompts and all outputs are provided in Multimedia Appendix 3 to ensure transparency and reproducibility. Moreover, we strived for maximum transparency in the presentation of our methodology, including data sources, analytical procedures, and reviewer involvement.

## Results

### Article Characteristics

From January 1, 2024, to December 31, 2024, a total of 229 articles were screened. A total of 23 articles (10%) were excluded because they were not primarily related to prostate cancer. Two (0.87%) additional articles were excluded as they were neither classified as original research articles nor reviews, consequently lacking a lay summary. This resulted in the inclusion of 204 articles (Multimedia Appendix 3).

From the 204 articles, 60 (29%) focused on prostate cancer diagnostics, 79 (39%) on prostate cancer therapy, and 14 (6.9%) covered both prostate cancer diagnostics and therapy. The remaining 51 (25%) articles addressed other topics. Accordingly, 101 (50%) articles were categorized as clinical research, 36 (18%) as basic research, and 67 (33%) as translational research. In total, 123 (60%) were original research articles, while 81 (40%) were meta-analyses or review articles.

The corresponding authors of 96 (47%) articles were affiliated with institutions in Europe, of 68 (33%) with institutions in North America, of 3 (1.5%) in South America, of 30 (15%) in Asia, and of 7 (3.4%) in Australia.

### Readability, Factual Accuracy, Word Count, and Composite Overall Quality Assessment

**Figure 1.**
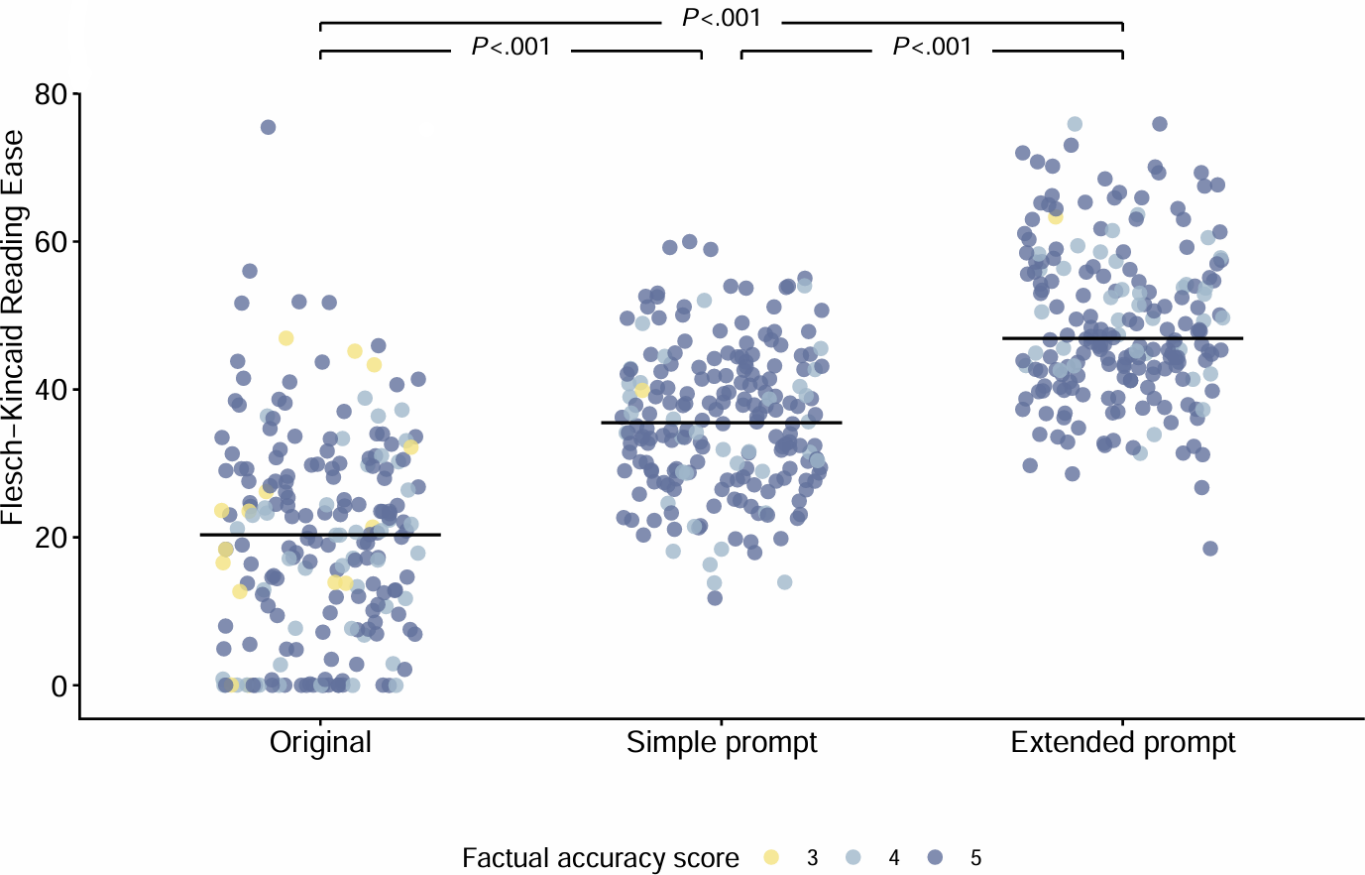
Readability scores measured by the Flesch-Kincaid Reading Ease (FKRE) for the original lay summaries and for ChatGPT-4-generated summaries using simple and extended prompts. The x-axis shows the 3 summary types, and the y-axis displays FKRE values (higher scores indicate easier readability). Color coding represents corresponding factual accuracy scores, with higher scores reflecting better fidelity to the source text. Median values are displayed as horizontal lines. Negative FKRE values were reset to 0 for visualization to preserve interpretability of the scale. Group comparisons were performed using the Wilcoxon signed-rank test.

Compared to the original lay summaries, those generated by ChatGPT-4 (using both simple and extended prompts) exhibited improved readability metrics, generally higher factual accuracy, and better adherence to the predefined correct word count. Consequently, a greater proportion of the ChatGPT-4 generated lay summaries met criteria for high-quality classification (ChatGPT-4 extended prompt 79%; ChatGPT-4 simple prompt 55%; original lay summary 5.4%; *P*<.001). Interobserver agreement for the content assessments was substantial (K=0.679; *P*<.001). Tables3^5^ present a detailed description and statistical comparison of text metrics, readability scores, factual accuracy, and overall assessment across the original lay summary, the ChatGPT-4 simple prompt, and the ChatGPT-4 extended prompt. Figure 1 displays a comparative grid plot of FKRE scores for the three lay summary versions, illustrating the higher median readability values alongside the corresponding factual accuracy scores.

**Table 3.** Descriptive data regarding length metrics and readability scores of the original lay summaries and those generated by ChatGPT-4 (simple vs extended prompt; N=204). The highest readability performance indices are highlighted in italic.

Parameter	Original lay summary	ChatGPT-4 simple prompt	ChatGPT-4 extended prompt	Standardized test statistic (Z values)	*P* values
Text metrics					
Sentences, median (IQR)	5 (4-7)	6 (6-7)	7 (6-7)	5.528^a^^b^ 4.627^b^^c^ 6.572^b^^d^	<.001^a^^c^^d^
Words, median (IQR)	117 (95‐140)	139 (129‐144)	139 (129‐145)	.121^a^^e^ 6.956^b^^c^ 6.625^b^^d^	.90^a^ <.001^c^^d^
Complex words, median (IQR)	31 (23‐39)	26 (21‐30)	20 (15‐24)	11.619^a^^e^ 6.712^c^^e^ 11.319^d^^e^	<.001^a^^c^^d^
Percent of complex words, median (IQR)	27 (23‐31)	19 (16‐22)	14 (11‐18)	11.818^a^^e^ 11.237^c^^e^ 12.338^d^^e^	<.001^a^^c^^d^
Average words per sentence, median (IQR)	22 (19‐25)	22 (20‐24)	20 (19‐22)	6.191^a^^e^ .894^c^^e^ 3.790^d^^e^	.37^c^ <**.**001^a^^d^
Average syllables per word, median (IQR)	1.9 (1.9‐2.1)	1.8 (1.7‐1.9)	1.6 (1.6‐1.7)	11.714^a^^e^ 10.852^c^^e^ 12.234^d^^e^	<.001^a^^c^^d^
Readability Scores^f^					
FKRE^g^, median (IQR)	20 (9.5‐29)	36 (29‐43)	47 (42‐56)	12.106^a^^b^ 10.852^c^^b^ 12.268^d^^b^	<.001^a^^c^^d^
FKGL^h^, median (IQR)	16 (14‐18)	14 (13‐15)	12 (10‐13)	11.693^a^^e^ 9.446^c^^e^ 11.936^d^^e^	<.001^a^^c^^d^
GFS^i^, median (IQR)	19 (17‐22)	16 (15‐17)	14 (12‐15)	11.770^a^^e^ 10.042^c^^e^ 12.007^d^^e^	<.001^a^^c^^d^
SMOG^j^ Index, median (IQR)	14 (13‐15)	12 (11‐13)	10 (9‐11)	11.784^a^^e^ 10.451^c^^e^ 12.187^d^^e^	<.001^a^^c^^d^
CLI^k^, median (IQR)	18 (17‐20)	17 (16‐18)	15 (14‐16)	11.475^a^^e^ 4.746^c^^e^ 10.811^d^^e^	<.001^a^^c^^d^
ARI^l^, median (IQR)	17 (15‐19)	16 (15‐17)	14 (12‐15)	11.408^a^^e^ 3.759^c^^e^ 9.641^d^^e^	<.001^a^^c^^d^
Reading age (y); median (IQR)	23 (21‐24)	21 (20‐22)	19 (17‐20)	11.511^a^^e^ 8.210^c^^e^ 11.545^d^^e^	<.001^a^^c^^d^

a ChatGPT-4 simple prompt versus ChatGPT-4 extended prompt.

b Wilcoxon signed ranks test based on negative ranks.

c Original lay summary versus ChatGPT-4 simple prompt.

d Original lay summary versus ChatGPT-4 extended prompt.

e Wilcoxon signed-ranks test based on positive ranks.

f In FKRE, the higher values indicate easier readability. For all indices except FKRE, lower values indicate easier readability.

g FKRE: Flesch-Kincaid Reading Ease.

h FKGL: Flesch-Kincaid Grade Level.

i GFS: Gunning Fog Score.

j SMOG: Simple Measure of Gobbledygook.

k CLI: Coleman-Liau Index.

l ARI: Automated Readability Index.

**Table 4.** Factual accuracy of the original lay summaries and those generated by ChatGPT-4 (simple vs extended prompt). Italic letters indicate statistical significance (N=204).

Assessment of factual accuracy, readability (FKRE^a^), and word count	Original lay summary	ChatGPT-4 simple prompt	ChatGPT-4 extended prompt	Standardized test statistic (*Z *values)	*P *values
Factual accuracy score 1 (performed by MM)				.626^b^^f^ 3.994^c^^e^ 3.631^d^^e^	.53^b^ <.001^c^^d^
1 point, n (%)	0 (0)	0 (0)	0 (0)		
2 points, n (%)	0 (0)	0 (0)	0 (0)		
3 points, n (%)	15 (7.4)	1 (0.5)	1 (0.5)		
4 points, n (%)	45 (22)	35 (17)	38 (19)		
5 points, n (%)	144 (71)	168 (82)	165 (81)		
Median (IQR)	5 (4-5)	5 (5-5)	5 (5-5)		
Factual accuracy score 2 (performed by JB)				1.512^b^^e^ 4.845^c^^e^ 5.507^d^^e^	.13^b^ <.001^c^^d^
1 point, n (%)	0 (0)	0 (0)	0 (0)		
2 points, n (%)	2 (1.0)	0 (0)	0 (0)		
3 points, n (%)	20 (9.8)	4 (2.0)	2 (1.0)		
4 points, n (%)	55 (27)	38 (19)	34 (17)		
5 points, n (%)	127 (62)	162 (79)	168 (82)		
Median (IQR)	5 (4-5)	5 (5-5)	5 (5-5)		

a FKRE: Flesch-Kincaid Reading Ease.

b ChatGPT-4 simple prompt versus ChatGPT-4 extended prompt.

c Original lay summary versus ChatGPT-4 simple prompt.

d Original lay summary versus ChatGPT-4 extended prompt.

e Wilcoxon signed ranks test based on negative ranks.

f Wilcoxon signed ranks test based on positive ranks.

**Table 5.** Assessment of factual accuracy, readability (Flesch-Kincaid Reading Ease [FKRE]), and word count, leading to an overall quality assessment of the original lay summaries and those generated by ChatGPT-4 (simple vs extended prompt). Italic letters indicate statistical significance (N=204).

Parameter	Original lay summary	ChatGPT-4 simple prompt	ChatGPT-4 extended prompt	Standardized test statistic (*Z* values)	*P* values
Factual accuracy scores; overall evaluation				.816^a^^b^ 3.789^b^^c^ 3.980^b^^d^	.41^a^ <.001^c^^d^
1 rating <4, n (%)	13 (6.4)	3 (1.5)	1 (0.5)		
2 ratings <4; n (%)	12 (5.9)	1 (0.5)	1 (0.5)		
FKRE				7.066^a^^b^ 9.869^b^^c^ 11.252^b^^d^	<.001^a^^c^^d^
FKRE 29.9‐20, n (%)	59 (29)	50 (24.5)	3 (1.5)		
FKRE<20, n (%)	99 (49)	10 (4.9)	1 (0.5)		
Wrong number of words, n (%)	140 (69)	40 (20)	39 (19)	.160^a^^b^ 9.869^b^^c^ 8.962^b^^d^	.87^a^ <.001^c^^d^
Overall quality assessment				5.758^a^^b^ 11.260^b^^c^ 11.741^b^^d^	<.001^a^^c^^d^
High quality					
0 points, n (%)	11 (5.4)	112 (55)	161 (79)		
Minor limitations					
1 point; n (%)	48 (24)	72 (35)	40 (20)		
2 points, n (%)	68 (33)	17 (8.3)	2 (1)		
Total, n (%)	116 (57)	89 (44)	42 (21)		
Moderate limitations					
3 points, n (%)	64 (31)	3 (1.5)	1 (0.5)		
Major limitations					
4 points, n (%)	7 (3.4)	0 (0)	0 (0)		
5 points, n (%)	6 (2.9)	0 (0)	0 (0)		
Total, n (%)	13 (6.4)	0 (0)	0 (0)		

a ChatGPT-4 simple prompt versus ChatGPT-4 extended prompt.

b Wilcoxon signed ranks test based on positive ranks.

c Original lay summary versus ChatGPT-4 simple prompt.

d Original lay summary versus ChatGPT-4 extended prompt.

e Wilcoxon signed ranks test based on negative ranks.

These findings were consistent across the subgroups of clinical, basic, and translational research articles. In each domain, prompts generated by ChatGPT-4 yielded a higher proportion of high-quality patient summaries than the original lay summaries. This was primarily driven by improvements in readability metrics and factual accuracy. Detailed analyses are provided in Tables S1-S3 in Multimedia Appendix 4.

Group differences among basic, clinical, and translational research articles regarding length metrics, readability scores, and factual accuracy

### Original Lay Summaries

We found that the lay summaries of translational science articles and clinical science articles contained significantly fewer words compared to those of basic science articles. Translational science lay summaries also contained fewer sentences than basic science lay summaries. Basic research lay summaries showed fewer factual inaccuracies than translational science lay summaries. Basic science lay summaries received a more favorable overall evaluation compared to those from clinical science. Apart from this, no significant differences were observed among the lay summaries of clinical, basic, or translational science articles with respect to readability metrics, text length metrics, factual accuracy, or the overall evaluation of the summaries. Detailed analyses are depicted in Tables S4 and S5 in Multimedia Appendix 4.

### ChatGPT-4 Simple Prompt

Compared to clinical science and translational science lay summaries, basic science lay summaries contained fewer complex words, a lower percentage of complex words, and fewer syllables per word. They also showed significantly higher FKRE and lower FKGL scores. In addition, the reading age was lower than that of translational science lay summaries (Tables S4 and S6, in Multimedia Appendix 4).

### ChatGPT-4 Extended Prompt

Basic science lay summaries contained fewer complex words and fewer syllables per word compared to clinical science lay summaries. They also showed higher FKRE, lower FKGL, and lower Gunning Fog Scores, a lower Smog index, and a lower reading age than clinical science lay summaries. Compared to translational science lay summaries, clinical lay summaries contained more sentences and more words. Tables S4 and S7 in Multimedia Appendix 4 provide a comparative overview including detailed analyses.

## Discussion

### Principal Findings

This study provides a large-scale evaluation of ChatGPT-4’s ability to generate lay summaries for biomedical research, using prostate cancer articles published in *Cancers* as a testbed. Through a direct comparison of human-written and AI-generated lay summaries across 2 prompting strategies, we assessed differences in readability, factual accuracy, and adherence to editorial guidelines. Findings suggest that generative AI, when properly guided, can significantly enhance the clarity and accessibility of scientific communication.

Consistent with prior work, our results confirm that many author-generated summaries exceed recommended reading levels and fail to meet readability thresholds, reflecting the difficulty of translating technical content for a general audience [12-14]. Domain expertise alone does not ensure clarity, as lay language writing remains an untrained skill for many scientists [10,15]. Against this backdrop, our findings demonstrate that ChatGPT-4 can produce summaries with improved readability and a more coherent structure than human-written alternatives.

These findings are consistent with emerging work demonstrating how AI systems may improve both the accessibility and trustworthiness of biomedical communication. Markowitz [22] highlights the broader societal potential of AI to support science communication, and Šuto Pavičić et al [23] document direct improvements in readability and presentation of oncology-related lay summaries, reinforcing the practical implications of our results.

The observed performance gap between simple and extended prompts highlights the importance of prompt design. This finding is consistent with our prior study, which demonstrated that carefully tailored prompts can improve both linguistic quality and content precision in AI-generated summaries [19]. Subgroup analysis revealed consistent domain-specific differences: basic science summaries, both human- and AI-generated, tended to use simpler language and, in some cases, contained fewer factual inaccuracies than clinical or translational summaries. This suggests that summarization performance may vary across biomedical domains, indicating a potential need for domain-adapted prompts or training and domain-sensitive quality checks in editorial workflows.

Our evaluation framework extends earlier work that focused predominantly on linguistic simplicity [17,18,23] by integrating measures of editorial integrity, such as adherence to word count and factual accuracy, into a standardized comparison with human-authored content. Conducting the study within the editorial environment of a journal requiring lay summaries ensured assessment under realistic conditions and offers a preliminary transferable model for future implementation.

Beyond improving editorial efficiency, AI-assisted summarization may reduce variability in author performance, alleviate researchers' workload, and promote more equitable access to knowledge, thereby supporting broader goals of patient and public engagement [1-5,29].

Alongside these practical benefits, the responsible use of generative AI must be guided by ethical and practical safeguards. Although ChatGPT-4 outputs showed strong quality in this study, LLMs remain vulnerable to hallucinations and lack intrinsic fact-checking mechanisms. Human oversight remains indispensable to ensure accuracy and ethical integrity, and concerns about reproducibility, bias, and transparency require ongoing attention [21].

Editorial boards should carefully evaluate the integration of AI-assisted summarization within a structured peer-review process to ensure the integrity and trustworthiness of content delivered to the public.

Finally, our methodology operationalizes several DECIDE-AI recommendations, such as prompt standardization, performance benchmarking, and blinded evaluation. Although this study does not constitute a clinical deployment, it may serve as a preparatory model for future AI-assisted health communication tools [24].

### Limitations

Several limitations merit consideration. First, this study focused exclusively on prostate cancer articles published in a single journal, which limits the generalizability of our findings to other medical disciplines or editorial ecosystems. Second, while independent experts evaluated all summaries, qualitative aspects such as tone, nuance, and audience engagement remain partially subjective, even when assessed using structured rubrics [10,15]. Third, the composite quality definition using thresholds for FKRE, factual accuracy, and word count, while pragmatic, is necessarily somewhat arbitrary given the absence of consensus on minimal FKRE standards for lay summaries. Alternative thresholds could yield different classification outcomes. These parameters should therefore be regarded as exploratory benchmarks rather than universal standards. Fourth, the performance of ChatGPT-4 is specific to its current model iteration; as LLMs continue to evolve, future updates may produce different results. Therefore, the reproducibility and temporal consistency of AI-generated outputs warrant ongoing scrutiny. Fifth, the potential for hallucinations must be carefully considered when applying LLMs in any context. Although no evidence for such hallucinations was observed in this study’s setting, likely due to the constrained task of generating lay summaries on the basis of article metadata, LLMs are inherently prone to these errors due to the probabilistic nature of their architecture. This limitation is particularly relevant in health care contexts and should be addressed through editorial safeguards, including expert oversight and review processes that combine automated generation with human validation.

Finally, and most importantly, this study did not include patients, caregivers, or members of the general public to evaluate comprehension, perceived clarity, or trust from the perspective of lay readers. These endpoints are critical for determining the real-world communicative effectiveness of lay summaries [13,17,22], highlighting an important gap given the growing emphasis on co-designed digital health communication [1-5,29,30]. Practical approaches should follow scientifically rigorous methodological protocols. For example, blinded rating of comprehension using Likert scales or testing understanding by asking lay persons to reproduce the content of a lay summary in their own words, with meaningful operationalization of results, can help ensure validity and reproducibility. In addition, inclusion of lay readers could involve structured comprehension surveys, focus groups, or co-design workshops, thereby supporting the development of lay summaries that meet the informational needs and expectations of end users. Prior research indicates that users often cannot reliably distinguish AI-generated from human-authored texts [31-33], and the impact of labeling content as AI-generated remains unclear. Some evidence suggests that explicit AI disclosure may reduce trust [34-36], yet transparency is essential for ethical communication.

Future work should prioritize rigorous usability testing that incorporates feedback from lay audiences through thoughtfully designed studies. Such evaluations should go beyond assessing comprehension to also examine potential downstream effects, including improved patient knowledge, increased confidence, and enhanced shared decision-making. Such efforts will be vital to ensuring that generative AI truly enhances patient-centered communication rather than merely optimizing textual outputs.

### Conclusions

This study suggests that, when guided by carefully structured prompts, ChatGPT-4 can generate lay summaries that, within the context of prostate cancer articles and editorial requirements evaluated here, demonstrate improved readability, factual accuracy, and adherence to word count guidelines compared to human-written versions. Prompt optimization notably influences output quality, indicating a scalable approach to enhancing accessibility in scientific communication.

The broader adoption of generative AI tools in editorial workflows offers a promising opportunity to democratize knowledge, reduce variability in lay communication, and strengthen public trust in science. To realize these benefits responsibly, journals should consider implementing concrete measures. First, structured prompt templates could be offered to authors at submission to encourage more consistent and high-quality lay summaries. Second, all AI-assisted summaries should undergo mandatory human editorial review to ensure factual accuracy and safeguard against potential errors or omissions. Third, alignment with established health literacy and plain language frameworks is essential to guarantee accessibility across diverse readerships. Finally, publishers may also explore the development or adoption of in-house AI models to maintain institutional control, protect data privacy, and reduce dependence on external providers.

Future research should extend beyond technical evaluations to include direct user testing with diverse patient populations, integrating comprehension studies, focus groups, and co-design workshops. Such efforts will be pivotal in validating the accessibility and trustworthiness of AI-generated lay communication and in shaping evidence-based editorial policies that balance innovation with responsibility.

## Supplementary material

10.2196/76598 Multimedia Appendix 1 Detailed information on prompt development.

10.2196/76598 Multimedia Appendix 2 Examples of lay summaries with their corresponding factual accuracy ratings and explanations for the assigned scores.

10.2196/76598 Multimedia Appendix 3 Details of included articles, abstracts, keywords, and original and ChatGPT-4-generated lay summaries with readability metrics, word counts, and comprehension scores.

10.2196/76598 Multimedia Appendix 4 Evaluation of lay summaries stratified into clinical, basic, and translational research.

10.2196/76598 Checklist 1 DECIDE-AI checklist

## Abbreviations

AI: artificial intelligence
DECIDE-AI: Developmental and Exploratory Clinical Investigations of Decision-Support Systems driven by Artificial Intelligence
FKGL: Flesch-Kincaid Grade Level
FKRE: Flesch-Kincaid Reading Ease
LLM: large language model
