# Supplementary material for: Using ChatGPT-4 for Lay Summarization in Prostate Cancer Research to Advance Patient-Centered Communication: Large-Scale Generative AI Performance Evaluation
Source: J Med Internet Res. 2025 Nov 19;27:e76598. doi: 10.2196/76598 (PMC12629520; doi:10.2196/76598)
Supplement: Multimedia Appendix 1 [file jmir-v27-e76598-s001.pdf]

Multimedia Appendix 1

Advancing Patient-Centered Communication with Generative AI: A Large-Scale Evaluation of ChatGPT-4 for Lay Summarization in Prostate Cancer Research

Emily Rinderknecht[1,2,*], Simon Engelmann[1], Veronika Saberi[1], Clemens Kirschner[1], Anton P. Kravchuk[3], Anna Schmelzer[4], Johannes Breyer[1], Christopher Goßler[1], Roman Mayr[1], Christian Gilfrich[3], Maximilian Burger[1], Dominik von Winning[3], Hendrik Borgmann[2,5], Christian Wülfing[2,6], Axel S. Merseburger[7], Maximilian Haas[1,#], and Matthias May[2,3,#]

[1] Department of Urology, University of Regensburg, Caritas St. Josef Medical Center, 93053 Regensburg, Germany.

[2] Working Group on Artificial Intelligence and Digitalization of the German Society of Urology.

[3] Department of Urology, St. Elisabeth Hospital Straubing, 94315 Straubing, Germany.

[4] Department of Urology, Nuremberg General Hospital, Paracelsus Medical University, 90419 Nuremberg, Germany.

[5] Department of Urology, Brandenburg Medical School Theodor Fontane (MHB), 14770 Brandenburg an der Havel, Germany.

[6] Department of Urology, Asklepios Klinik Altona, 22763 Hamburg, Germany.

[7] Department of Urology, University Hospital Schleswig-Holstein, Campus Lübeck, 23562 Lübeck, Germany.

[#] Shared senior-authors / These authors contributed equally to this work.

[*] Correspondence: erinderknecht@csj.de

Multimedia Appendix 1

Detailed information on prompt development.

Simple Prompt:

Dear ChatGPT-4o,

I kindly request your assistance in crafting a Simple Summary as part of a scientific study.

The Simple Summary must adhere to the following guidelines:

It should be written in one paragraph, in layman's terms, to explain why the research is being suggested, what the authors aim to achieve, and how the findings from this research may impact the research community. Please use as few abbreviations as possible, and do not cite references in the Simple Summary. The Simple Summary must not exceed 150 words.

To provide you with the necessary context for creating this Simple Summary, I will supply you with the study title, a scientifically accurate abstract (not in layman's terms), and the relevant keywords.

Study title: "…"

Scientifically accurate abstract: "…"

Keywords: "…"

Please note: Summarize this unstructured abstract (simple summary) in lay language, highlighting the study purpose, methods, key findings, and practical importance of these findings for the general public. Additionally, be aware that the Simple Summary must not exceed 150 words, but it should make the most of this limit.

The different components of the simple prompt are highlighted using color codes, each representing a functional element. Below is a rationale for including each component in its current form. A closely related prompt was previously developed and applied by our research group in the context of a preliminary study exploring AI-assisted generation of lay summaries[1].

- Yellow: Clearly defines the task to ensure clarity and focus.
- Green: Reflects author instructions derived from the journal's editorial guidelines[2].
- Blue: Provides essential context.

- Purple: Reduces technical jargon, following the lay summary prompt structure suggested by Shyr et al.[3]
- Red: Ensures key information is retained, in alignment with the prompt used by Shyr et al.[3]
- Brown: Emphasizing practical importance, in alignment with the prompt used by Shyr et al.[3]
- Grey: Verifies compliance with specified length.

The simple prompt was then tested, and the general results indicated good alignment with maximum word count, and good factual accuracy, however readability measured by the FKRE was not optimal, yet. This is why, analog to the extended prompt used in our preliminary study[1], the addition of the following two passages was tested.

1) *The Simple Summary should be crafted with a focus on maximizing readability, aiming for the highest Possible Flesch-Kincaid Reading Ease score.*
2) *at a 6th grade reading level (as propsed by Shyr et al. [3]).*

These passages were first added individually, however adding both together seemed to receive the best results, which is why we decided to do so in the resulting extended prompt.

Following, two examples are shown, to illustrate the different resulting lay summaries received using the different prompts with respective FKRE-measures.

Example 1: **Castration-Resistant Prostate Cancer: From Uncovered Resistance Mechanisms to Current Treatments[4]**

Simple Prompt:

Prostate cancer is one of the most common cancers in men, and while early treatment can be effective, some forms become resistant and continue to grow despite hormone therapy. This condition is called castration-resistant prostate cancer (CRPC), and it is especially hard to treat. In this study, we explain how prostate cancer cells manage to survive and grow even when testosterone is blocked. We describe different resistance mechanisms, such as changes in hormone receptors and alternative growth pathways, that allow the cancer to keep spreading. We also summarize the current treatment options for CRPC, including chemotherapy, immunotherapy, radiation, and targeted drugs like PARP inhibitors. By better understanding how CRPC develops and how it resists treatment, researchers and doctors can find more effective ways to fight the disease. This knowledge may lead to new and improved treatment strategies that offer patients longer and better lives.

- ➔ FKRE 41.7

Simple Prompt plus passage 1:

Prostate cancer is one of the most common cancers in men. When it no longer responds to hormone-blocking treatment—a stage called castration-resistant prostate cancer—it becomes much harder to treat. This study explains how prostate cancer cells can become resistant by changing their behavior or finding new ways to grow even without male hormones. The authors explore the most important reasons why this resistance happens and describe the treatments that are currently available, such as hormone therapy, chemotherapy, radiation, immune-based treatments, and new targeted drugs. They also look at how combining different treatments might improve outcomes. By pulling together the latest information, this study helps doctors and researchers better understand how this cancer works and what can be done to treat it more effectively. The goal is to support the development of smarter, more personalized treatments that can improve the lives of men living with advanced prostate cancer.

- ➔ FKRE 43.5

Simple Prompt plus passage 2:

Prostate cancer is a common disease in men, and many patients respond well to early treatment. However, some cancers become harder to treat over time and stop responding to hormone therapy—a stage known as castration-resistant prostate cancer. This study explains how these cancer cells find ways to grow even without the male hormones they usually need. The authors looked at how the cancer resists treatment and what options are currently available, such as chemotherapy, radiation, immune-based therapies, and newer drugs that target cancer in different ways. By exploring these treatment choices and the ways cancer escapes control, the study helps doctors and researchers better understand the disease. This knowledge may lead to improved treatment plans and give hope for better outcomes in the future. The goal is to help patients live longer and improve their quality of life, even in advanced stages of prostate cancer.

➔ FKRE 51.4

Extended Prompt:

Prostate cancer is a common cancer in men. Many men do well with treatments that lower male hormones, but over time, the cancer can stop responding. This harder-to-treat stage is called castration-resistant prostate cancer. In this study, the authors explain how cancer cells find ways to keep growing even without male hormones. They explore different ways the cancer becomes stronger, such as changing how it uses signals in the body or using other growth paths. The study also looks at current treatments, including hormone therapy, chemotherapy, radiation, immune-based treatments, and special drugs that target cancer cells. The authors highlight the need to understand these changes better so doctors can choose the best treatment for each patient. By sharing this information, the study helps researchers and doctors work together to find better ways to treat advanced prostate cancer and improve the lives of men living with the disease.

➔ FKRE 57.1

Example 2: **Unraveling the Role of EV-Derived miR-150-5p in Prostate Cancer Metastasis and Its Association with High-Grade Gleason Scores: Implications for Diagnosis[5]**

Simple Prompt

Prostate cancer can become life-threatening when it spreads to other parts of the body, especially the lymph nodes. Detecting this spread early is crucial for choosing the right treatment. In this study, researchers looked at tiny molecules called microRNAs, which travel in small particles called extracellular vesicles found in blood. By studying tissue and blood samples from patients with and without cancer spread, the team discovered that one specific microRNA—called miR-150-5p—was found at lower levels in patients with aggressive prostate cancer. This microRNA may play a role in how cancer spreads to the bones. The study suggests that measuring miR-150-5p in blood samples could help identify patients with high-risk prostate cancer earlier and more accurately. This approach could lead to less invasive testing and better decision-making for treatment, offering hope for earlier diagnosis and improved outcomes in men facing this common disease.

- ➔ FKRE 47.8

Simple Prompt plus passage 1:

Prostate cancer can become life-threatening when it spreads to other parts of the body. Detecting this spread early is important, but current tools are not always reliable. This study looked at tiny molecules called microRNAs, which are released by cancer cells into the bloodstream inside small particles known as extracellular vesicles. The goal was to see if one of these microRNAs, called miR-150-5p, could help doctors detect aggressive prostate cancer and early signs of spread to the lymph nodes. Researchers compared samples from tumors, blood, and lymph nodes and found that patients with more aggressive cancer had lower levels of miR-150-5p. These findings suggest that measuring miR-150-5p in a simple blood test may help spot dangerous forms of prostate cancer sooner and guide better treatment choices. This could offer a less invasive and more accurate way to diagnose and monitor prostate cancer in the future.

- ➔ FKRE 55.7

Simple Prompt plus passage 2:

Prostate cancer can become life-threatening when it spreads to other parts of the body. Doctors are looking for better ways to detect this spread early. This study explored tiny molecules called microRNAs that travel in the blood inside small particles from cancer cells. The researchers focused on one specific microRNA, called miR-150-5p, and studied how it appears in both cancer tissue and blood samples. They found that men with more aggressive prostate cancer had much lower levels of this molecule. These findings suggest that checking for miR-150-5p in a blood test could help doctors spot cancer that is likely to spread, even before it becomes visible on scans. This may help guide treatment decisions earlier and more accurately. The results are important because they bring us closer to a simple blood test that could warn of dangerous prostate cancer before it gets worse.

- ➔ FRKE 61.2

Extended Prompt:

Prostate cancer can spread to other parts of the body, which makes it harder to treat. This study looked at tiny messages, called microRNAs, that travel between cells inside small particles called extracellular vesicles. The goal was to find out if these microRNAs could help doctors detect when prostate cancer is more aggressive or likely to spread. The researchers studied tissue and blood samples from patients and found that one microRNA, called miR-150-5p, was much lower in patients with more dangerous cancer. They also found signs that this microRNA might be linked to cancer spreading to the bones. These findings suggest that measuring miR-150-5p in blood could help spot prostate cancer earlier and tell how severe it is. This could give doctors a better way to diagnose and treat prostate cancer before it gets worse, helping patients get the right care at the right time.

- ➔ FKRE 64.3

References

1. Rinderknecht E, Schmelzer A, Kravchuk A, Goßler C, Breyer J, Gilfrich C, Burger M, Engelmann S, Saberi V, Kirschner C, Winning D, Mayr R, Wülfing C, Borgmann H, Buse S, Haas M, May M. Leveraging Large Language Models for High-Quality Lay Summaries: Efficacy of ChatGPT-4 with Custom Prompts in a Consecutive Series of Prostate Cancer Manuscripts. Current Oncology 2025;32(2):102. doi:10.3390/curroncol32020102
2. Cancers - Instructions for Authors URL: https://www.mdpi.com/journal/cancers/instructions [accessed 2025-02-19].
3. Shyr C, Grout RW, Kennedy N, Akdas Y, Tischbein M, Milford J, Tan J, Quarles K, Edwards TL, Novak LL, White J, Wilkins CH, Harris PA. Leveraging artificial intelligence to summarize abstracts in lay language for increasing research accessibility and transparency. J Am Med Inform Assoc 2024. PMID:39008829
4. Le TK, Duong QH, Baylot V, Fargette C, Baboudjian M, Colleaux L, Taïeb D, Rocchi P. Castration-Resistant Prostate Cancer: From Uncovered Resistance Mechanisms to Current Treatments. Cancers (Basel) 2023;15(20). PMID:37894414
5. Cruz-Burgos M, Cortés-Ramírez SA, Losada-García A, Morales-Pacheco M, Martínez-Martínez E, Morales-Montor JG, Servín-Haddad A, Izquierdo-Luna JS, Rodríguez-Martínez G, Del Ramos-Godínez MP, González-Covarrubias V, Cañavera-Constantino A, González-Ramírez I, Su B, Leong HS, Rodríguez-Dorantes M. Unraveling the Role of EV-Derived miR-150-5p in Prostate Cancer Metastasis and Its Association with High-Grade Gleason Scores: Implications for Diagnosis. Cancers (Basel) 2023;15(16). PMID:37627176
