# Supplementary material for: Using ChatGPT-4 for Lay Summarization in Prostate Cancer Research to Advance Patient-Centered Communication: Large-Scale Generative AI Performance Evaluation
Source: J Med Internet Res. 2025 Nov 19;27:e76598. doi: 10.2196/76598 (PMC12629520; doi:10.2196/76598)
Supplement: Multimedia Appendix 2 [file jmir-v27-e76598-s002.pdf]

Multimedia Appendix 2

Advancing Patient-Centered Communication with Generative AI: A Large-Scale Evaluation of ChatGPT-4 for Lay Summarization in Prostate Cancer Research

Emily Rinderknecht[1,2,*], Simon Engelmann[1], Veronika Saberi[1], Clemens Kirschner[1], Anton P. Kravchuk[3], Anna Schmelzer[4], Johannes Breyer[1], Christopher Goßler[1], Roman Mayr[1], Christian Gilfrich[3], Maximilian Burger[1], Dominik von Winning[3], Hendrik Borgmann[2,5], Christian Wülfing[2,6], Axel S. Merseburger[7], Maximilian Haas[1,#], and Matthias May[2,3,#]

[1] Department of Urology, University of Regensburg, Caritas St. Josef Medical Center, 93053 Regensburg, Germany.

[2] Working Group on Artificial Intelligence and Digitalization of the German Society of Urology.

[3] Department of Urology, St. Elisabeth Hospital Straubing, 94315 Straubing, Germany.

[4] Department of Urology, Nuremberg General Hospital, Paracelsus Medical University, 90419 Nuremberg, Germany.

[5] Department of Urology, Brandenburg Medical School Theodor Fontane (MHB), 14770 Brandenburg an der Havel, Germany.

[6] Department of Urology, Asklepios Klinik Altona, 22763 Hamburg, Germany.

[7] Department of Urology, University Hospital Schleswig-Holstein, Campus Lübeck, 23562 Lübeck, Germany.

[#] Shared senior-authors / These authors contributed equally to this work.

[*] Correspondence: erinderknecht@csj.de

Multimedia Appendix 2

Examples of lay summaries with their corresponding factual accuracy ratings and explanations for the assigned scores. To represent the full scoring spectrum, manuscripts 15, 39, 79, 99, and 124 were selected as exemplars.

Manuscript 15

Jongen CAM, Heemsbergen WD, Incrocci L, Heijmen BJM, Rossi L. Added Value of Biological Effective Dose in Dosiomics-Based Modelling of Late Rectal Bleeding in Prostate Cancer. Cancers [Internet]. 2024 Dec 17;16(24):4208. Available from: http://dx.doi.org/10.3390/cancers16244208

Abstract

Background/objectives: Extracting spatial features (texture analysis) from dose distributions (dosiomics) for outcome prediction is a rapidly evolving field in radiotherapy. To account for fraction size differences, the biological effective dose (BED) is often calculated. We evaluated the impact and added value of the BED in the dosiomics prediction modelling of grade ≥ 2 late rectal bleeding (LRB) probability within 5 years after treatment in three parts.

Methods: For N = 656 prostate cancer patients previously treated in a randomized trial with conventional (CF) or hypofractionated (HF) radiotherapy, 42 dosiomic features were extracted from the dose distributions of the delineated rectum in physical doses and from dose distributions converted to the BED. Part 1: To assess whether an HF BED dosiomics model is generalizable to CF and vice versa, multivariate logistic regression BED models were constructed for HF and CF separately and tested on the other fractionation scheme. Part 2: The BED models were fitted to combined HF and CF data together to test whether this resulted in better models. Part 3: Separate physical HF and CF models were constructed and compared to the BED models.

Results: Part 1: Dosiomics related to large-zone and long-run high-dose levels were predictive for both HF and CF. Deviation from the mean gray level was only predictive for HF. The BED HF model calibrations with CF data and vice versa were generally poor. AUCs ranged from 0.55 to 0.65. Part 2: Compared to the separate models, the models fitted to the combined HF and CF data showed better discriminative ability in

CF but not in HF. Part 3: The apparent performances of models for the BED and physical dose were similar.

Conclusions: Using the BED in the predictive dosiomic modelling of late rectal bleeding after prostate cancer radiotherapy to account for differences in fraction doses was of limited value.

Keywords:

NTCP, dosiomics, late rectal bleeding, radiobiological modelling, texture analysis

Original Lay Summary

By identifying patterns and voxel correlations (texture analysis), it is possible to characterize dose distributions. These dose parameters are called dosiomic features. The linear-quadratic model is a widely applied model in radiotherapy to calculate the biological effective dose for a tissue of interest based on the fraction size sensitivity, fraction size and total dose. In this study, we investigated the added value and impact of applying biological effective dose to dosiomic features in prediction models.

Comprehension Score	Explanation
3	The lay summary is generally accurate and reflects the study's topic, but it omits key details such as the clinical outcome assessed (late rectal bleeding), the study population, and the main findings.

Lay Summary (Simple Prompt)

Radiotherapy is a common treatment for prostate cancer, but it can cause side effects like late rectal bleeding, which impacts quality of life. Scientists are exploring new ways to predict who might develop these side effects by analyzing patterns in how radiation doses are distributed—a technique called dosiomics. This study investigated whether adjusting these radiation dose patterns based on biological effects, known as the biological effective dose (BED), could improve the accuracy of predicting late rectal bleeding. Data from 656 prostate cancer patients treated with different radiotherapy methods were analyzed. The results showed that using BED did not significantly improve the ability to predict rectal bleeding compared to traditional dose measurements. This finding suggests that while dosiomics is a promising tool, adding BED may not provide extra benefits for predicting this specific side effect, helping researchers focus on more effective prediction methods in the future.

Comprehension Score	Explanation
5	The lay summary is factually accurate and fully aligned with the scientific abstract. It includes all essential information: the clinical context (late rectal bleeding), the method used (dosiomics, BED), the study population (656 patients with different radiotherapy regimens), and the main result.

<u>Lay Summary (Extended Prompt)</u>

Radiotherapy is a common treatment for prostate cancer, but it can sometimes cause long-term side effects like rectal bleeding. To better predict who might experience this side effect, researchers use advanced computer models that analyze how radiation is distributed in the body—a method called dosiomics. This study explored whether including a special calculation called the biological effective dose (BED), which adjusts for differences in treatment schedules, could improve these predictions. We analyzed data from 656 patients treated with different radiation approaches to see if BED-based models worked better than models using standard radiation dose data. Our results showed that adding BED did not significantly improve the accuracy of predicting rectal bleeding. This finding suggests that simpler models without BED may be just as effective, helping doctors focus on more straightforward ways to predict and manage side effects in prostate cancer patients.

Comprehension Score	Explanation
5	The lay summary is factually accurate. It is aligned with the abstract. It includes all essential information including clinical background, treatment population, methods, and results.

Manuscript 39

Pisotskyi O, Petrasz P, Zorga P, Gałęski M, Szponar P, Brzeźniakiewicz-Janus K, et al. Enhancing Prostate Cancer Staging: Association of 68Ga-PSMA PET/CT Imaging with Histopathological Grading in Treatment-Naive Patients. Cancers [Internet]. 2024 Oct 18;16(20):3526. Available from: http://dx.doi.org/10.3390/cancers16203526

Abstract

Objective: This study aimed to evaluate the correlation between 68Ga-PSMA uptake in PSMA PET/CT in primary prostate cancer (PC) and its histopathological grading (Gleason score and ISUP grade). Additionally, we compared preoperative biopsy histopathological findings with definitive pathology results in radical prostatectomy (RP) specimens.

Methods: We retrospectively analyzed 86 patients who underwent 68Ga-PSMA PET/CT for primary PC staging, of which 40 patients later underwent radical prostatectomy. PET/CT results, including SUVmax values, were correlated with GS and PSA concentrations. Histopathology reports were analyzed and compared between biopsy and final pathology results following RP.

Results: A significant positive correlation was observed between SUVmax and ISUP grades (Pearson's $\rho = 0.34$, $p < 0.001$), with higher SUVmax values associated with more advanced grades. A cut-off SUVmax value of 5.64 was determined to predict upstaging in patients, yielding a sensitivity of 76% and a specificity of 60% (AUC = 0.82, 95% CI: 0.70-0.94). Additionally, 57.5% of patients experienced a grade shift following RP, with a 35% upgrade and 22.5% downgrade in ISUP grades.

Conclusion: 68Ga-PSMA PET/CT demonstrated high sensitivity in detecting high-risk prostate cancer, particularly in patients with GS > 7 or PSA levels ≥ 10 ng/mL. The findings suggest that this imaging modality may be less effective for the staging of

patients with lower GS or PSA values, that is, low-risk PCa. Further prospective studies are necessary to validate these results.

Keywords:

68Ga-PSMA PET/CT, PSMA expression, SUVmax correlation, biopsy vs. final pathology, histopathological grading, prostate cancer staging

<u>Original Lay Summary</u>

This study investigates the correlation between 68Ga-PSMA PET/CT imaging and histopathological grading in treatment-naive prostate cancer patients. We retrospectively analyzed 86 patients who underwent 68Ga-PSMA PET/CT scans for prostate cancer staging, with a subset of 40 patients also undergoing radical prostatectomy. The PET/CT results, specifically SUVmax values, were compared with Gleason scores and PSA levels to assess their association with cancer grades. Results indicated a significant positive correlation between SUVmax and ISUP grades, suggesting that higher SUVmax values correspond to more advanced prostate cancer stages. Furthermore, the analysis of biopsy and radical prostatectomy pathology reports revealed a substantial rate of grading changes, underscoring the limitations of biopsy alone for accurate staging. These findings highlight the potential utility of 68Ga-PSMA PET/CT as a diagnostic tool in stratifying prostate cancer risk and guiding treatment decisions. Future studies are recommended to validate these observations and explore further applications of this imaging modality.

Comprehension Score	Explanation
5	The lay summary is accurate, complete and well-aligned with the abstract. It clearly states the study objective. It specifies the study population and methodology, and explains key concepts such as SUVmax, Gleason score, and ISUP grade. Main findings are presented accurately. Clinical relevance is articulated. No important details are omitted.

<u>Lay Summary (Simple Prompt)</u>

Prostate cancer staging helps doctors determine the best treatment options for patients. This study explored whether a specialized imaging technique, called 68Ga-PSMA PET/CT, can accurately predict the severity of prostate cancer before surgery. Researchers analyzed scans from 86 patients and compared imaging results with tissue samples collected during surgery. They found that higher tumor activity on the scan was linked to more aggressive cancer. However, in over half of the cases, the initial biopsy results differed from the final diagnosis after surgery, showing that early assessments may sometimes be inaccurate. The study identified a specific scan value that may help predict when a patient's cancer stage will change after surgery. These findings suggest that 68Ga-PSMA PET/CT could improve prostate cancer staging, leading to better treatment decisions. Further research is needed to confirm its full potential in routine clinical practice.

Comprehension Score	Explanation
5	The lay summary accurately reflects the study’s objectives, methods, and key findings. It clearly states the correlation between 68Ga-PSMA PET/CT imaging and prostate cancer aggressiveness, as well as the significant discrepancies between biopsy and final pathology results. The summary also mentions the identified predictive scan value and the need for further research, making it complete and aligned with the abstract.

<u>Lay Summary (Extended Prompt)</u>

Prostate cancer is a common disease, and accurate staging is crucial for choosing the best treatment. This study explores how a special imaging technique, called 68Ga-PSMA PET/CT, helps predict the severity of prostate cancer before surgery. By analyzing scans from patients with untreated prostate cancer, researchers found that higher scan values were linked to more aggressive disease. The study also showed that initial biopsy results often differed from final surgical findings, meaning some cancers were actually more or less severe than first thought. These results suggest that 68Ga-PSMA PET/CT could improve how doctors assess prostate cancer, especially in higher-risk cases. However, the scan may be less useful for patients with less aggressive cancer. Future studies are needed to confirm these findings, which could lead to better treatment decisions and improved outcomes for patients with prostate cancer.

Comprehension Score	Explanation
5	The lay summary accurately covers the study’s aims, methods, and main findings. It also clearly explains the association between 68Ga-PSMA PET/CT scan values and prostate cancer aggressiveness, as well as the discrepancies between biopsy and surgical pathology. The summary also appropriately notes the scan’s limitations in low-risk cases and the need for further research. It aligns well with the abstract and includes all essential information.

Manuscript 79

Fekete B, Biró K, Gyergyay F, Polk N, Horváth O, Géczi L, et al. Prediction of PSA Response after Dexamethasone Switch during Abiraterone Acetate + Prednisolone Treatment of Metastatic Castration-Resistant Prostate Cancer Patients. Cancers [Internet]. 2024 Aug 3;16(15):2760. Available from: http://dx.doi.org/10.3390/cancers16152760

Abstract

Background: The aim was to elaborate a predictive model to find responders for the corticosteroid switch (from prednisolone to dexamethasone) at the first prostate-specific antigen (PSA) progression (≥25% increase) during abiraterone acetate (AA) treatment of metastatic castration-resistant prostate cancer (mCRPC) patients.

Methods: If PSA has decreased (≥25%) after switch, patients were considered responders. Logistic regression of 19 dichotomized parameters from routine laboratory and patients' history was used to find the best model in a cohort of 67 patients. The model was validated in another cohort of 42 patients.

Results: The model provided 92.5% and 90.5% accuracy in the testing and the validation cohorts, respectively. Overall the accuracy was 91.7%. The AUC of ROC curve was 0.92 (95% CI 0.85-0.96). After a median follow-up of 27.9 (26.3-84) months, the median AA+dexamethasone treatment duration (TD) in non-responders and responders was 4.7 (3.1-6.5) and 11.1 (8.5-12.9) months and the median overall survival (OS) was 23.2 (15.6-25.8) and 33.5 (26.1-38) months, respectively. Multivariate Cox regression revealed that responsiveness was an independent marker of TD and OS.

Conclusions: A high accuracy model was developed for mCRPC patients in predicting cases which might benefit from the switch. For non-responders, induction of the next systemic treatment is indicated.

Keywords:

corticosteroid switch, overall survival, predictive model, responders, treatment duration

<u>Original Lay Summary</u>

A model predicting responders to corticosteroid switch during abiraterone therapy in patients with metastatic castration-resistant prostate cancer is warranted. The model using logistic regression was developed in a cohort of 67 and validated in another cohort of 42 patients. The use of this model is easy and all needed parameters are available from routine laboratory and history of patients. The overall accuracy was 92%. Multivariate analysis revealed that responsiveness was a marker of longer treatment duration and overall survival. For non-responders a new systemic treatment was indicated.

Comprehension Score	Explanation
4	The lay summary is factually accurate and largely consistent with the scientific abstract. It describes the development and validation of the predictive model, the high overall accuracy, and the clinical significance of distinguishing responders from non-responders regarding treatment duration and survival. While some specific details—such as the PSA progression criterion and exact outcome differences—are omitted, these do not detract from the overall factual correctness. The summary effectively conveys the main findings and their relevance, with only minor, non-essential information missing or simplified.

<u>Lay Summary (Simple Prompt)</u>

In men with advanced prostate cancer that no longer responds to hormone therapy, treatment often includes a drug called abiraterone acetate combined with a steroid called prednisolone. When this treatment stops working, doctors sometimes switch the steroid from prednisolone to dexamethasone, which can lower prostate-specific antigen (PSA) levels in some patients. However, not all patients benefit from this switch. This study aimed to develop a model to predict which patients are likely to respond to the steroid change. Researchers analyzed medical data from 109 patients and identified key factors that could help predict a positive response. The model was highly accurate, correctly identifying responders in over 90% of cases. Patients who responded to the switch lived longer and had better treatment outcomes. This tool could help doctors make more informed decisions, ensuring patients receive the most effective treatment at the right time.

Comprehension Score	Explanation
5	The lay summary is fully accurate and aligns with the scientific abstract. It explains the clinical context of metastatic castration-resistant prostate cancer and the use of abiraterone acetate plus prednisolone. It accurately describes the steroid switch to dexamethasone and the goal of predicting responders. The summary correctly reports the patient cohort size (109 total), the high accuracy of the predictive model, and the association between response and improved survival and treatment duration. The information is complete, with no significant omissions or factual inaccuracies.

Lay Summary (Extended Prompt)

When treating advanced prostate cancer, some patients stop responding to a common drug combination of abiraterone acetate and prednisolone. In these cases, doctors sometimes switch the steroid from prednisolone to dexamethasone, but it's hard to predict who will benefit from this change. Our study aimed to create a simple model to help doctors identify which patients are likely to respond to this switch. We looked at the medical history and routine lab results of 67 patients to find key factors linked to treatment success. We then tested the model in another group of 42 patients to make sure it worked well. The model was very accurate, correctly predicting outcomes in over 90% of cases. Patients who responded to the switch lived longer and stayed on treatment for more time. This model can help doctors make better treatment decisions, ensuring patients get the most effective care at the right time.

Comprehension Score	Explanation
5	The lay summary is accurate and consistent with the scientific abstract. It explains the clinical challenge of predicting which patients will benefit from switching steroids during abiraterone acetate treatment for advanced prostate cancer. The summary correctly describes the study design. The reported model accuracy and the clinical relevance of response in terms of longer survival and treatment duration are accurately conveyed. The summary includes all essential information without significant omissions or factual errors.

Manuscript 99

Foley GR, Marthick JR, Lucas SE, Raspin K, Banks A, Stanford JL, et al. Germline Sequencing of DNA Damage Repair Genes in Two Hereditary Prostate Cancer Cohorts Reveals New Disease Risk-Associated Gene Variants. Cancers [Internet]. 2024 Jul 7;16(13):2482. Available from: http://dx.doi.org/10.3390/cancers16132482

Abstract

Rare, inherited variants in DNA damage repair (DDR) genes have a recognised role in prostate cancer (PrCa) susceptibility. In addition, these genes are therapeutically targetable. While rare variants are informing clinical management in other common cancers, defining the rare disease-associated variants in PrCa has been challenging. Here, whole-genome and -exome sequencing data from two independent, high-risk Australian and North American familial PrCa datasets were interrogated for novel DDR risk variants. Rare DDR gene variants (predicted to be damaging and present in two or more family members) were identified and subsequently genotyped in 1963 individuals (700 familial and 459 sporadic PrCa cases, 482 unaffected relatives, and 322 screened controls), and association analyses accounting for relatedness (MQLS) undertaken. In the combined datasets, rare ERCC3 (rs145201970, p = 2.57 × 10-4) and BRIP1 (rs4988345, p = 0.025) variants were significantly associated with PrCa risk. A PARP2 (rs200603922, p = 0.028) variant in the Australian dataset and a MUTYH (rs36053993, p = 0.031) variant in the North American dataset were also associated with risk. Evaluation of clinicopathological characteristics provided no evidence for a younger age or higher-grade disease at diagnosis in variant carriers, which should be taken into consideration when determining genetic screening eligibility criteria for targeted, gene-based treatments in the future. This study adds valuable knowledge to our understanding of PrCa-associated DDR genes, which will underpin effective clinical screening and treatment strategies.

Keywords:

DNA repair, genetic susceptibility, germline variants, prostate cancer, rare high-risk genes

<u>Original Lay Summary</u>

An urgent demand exists to identify inherited genetic risk variants for prostate cancer (PrCa), particularly in DNA damage repair genes targetable with precision medicine-based strategies. Though the most heritable common cancer, discovery of rare germline PrCa risk variants is hampered by their low frequency, even in sizeable population datasets. Through utilising two large, independent, familial PrCa resources and their likely enrichment of rare causative variants, we provide robust evidence for several novel risk variants in DNA damage repair genes.

Comprehension Score	Explanation
3	The lay summary accurately highlights the identification of novel inherited DNA damage repair gene variants associated with prostate cancer risk using familial datasets, while it omits some important specifics from the abstract. For example, it does not mention the exact genes found (such as ERCC3, BRIP1, PARP2, MUTYH) or that the variants were confirmed across two independent cohorts. Additionally, it lacks the detail that variant carriers did not show differences in age at diagnosis or disease aggressiveness, which is a relevant finding. These omissions mean the summary is generally accurate but not fully comprehensive or precise according to the scientific abstract.

<u>Lay Summary (Simple Prompt)</u>

Prostate cancer can run in families, partly due to inherited changes in certain genes that help repair damaged DNA. These genes are important because they not only influence cancer risk but can also guide treatment options. In this study, researchers analyzed genetic data from two groups of men with a family history of prostate cancer in Australia and North America. They searched for rare genetic changes that might increase the risk of developing the disease. By comparing the genes of men with and without prostate cancer, they identified new variations in four specific genes—ERCC3, BRIP1, PARP2, and MUTYH—that are linked to a higher risk of prostate cancer. Interestingly, men with these gene changes did not develop cancer at a younger age or have more aggressive disease. These findings could help improve genetic screening and support the development of personalized treatments for prostate cancer in the future.

Comprehension Score	Explanation
5	The summary is fully accurate and aligns well with the scientific abstract, including key details such as the focus on inherited DNA repair gene variants, the study populations from Australia and North America, and the identification of specific genes (ERCC3, BRIP1, PARP2, MUTYH) linked to prostate cancer risk. It also correctly notes that these variants were not associated with earlier onset or more aggressive disease. The summary effectively conveys the essential findings without omitting important information.

Lay Summary (Extended Prompt)

Prostate cancer can run in families, partly because of changes in certain genes that help repair damaged DNA. These gene changes, called variants, can increase the risk of developing cancer. In this study, researchers looked for new gene variants linked to prostate cancer risk by studying the DNA of families from Australia and North America with a history of the disease. They used advanced genetic tests to find rare gene changes that might cause prostate cancer. The study found several new gene variants that are linked to a higher risk of prostate cancer. Interestingly, having these variants did not mean the cancer would be more aggressive or appear at a younger age. These findings can help doctors better understand who is at risk and may lead to improved genetic screening and personalized treatments for prostate cancer in the future.

Comprehension Score	Explanation
4	The summary is factually accurate and largely consistent with the abstract, covering key points such as the familial aspect of prostate cancer, the focus on DNA repair gene variants, and the identification of new rare variants linked to increased risk. It also correctly mentions that these variants were not associated with earlier onset or higher-grade disease. However, it omits the specific genes identified (ERCC3, BRIP1, PARP2, MUTYH) and does not mention the use of two independent cohorts or the scale of the genetic testing and analysis.

Manuscript 124

Waseem M, Wang BD. Combination of miR-99b-5p and Enzalutamide or Abiraterone Synergizes the Suppression of EMT-Mediated Metastasis in Prostate Cancer. Cancers [Internet]. 2024 May 19;16(10):1933. Available from: http://dx.doi.org/10.3390/cancers16101933

Abstract

Prostate cancer (PCa) is the most frequently diagnosed cancer and second leading cause of cancer deaths among American men. Androgen deprivation therapy (ADT) has been systemically applied as a first-line therapy for PCa patients. Despite the initial responses, the majority of patients under ADT eventually experienced tumor progression to castration-resistant prostate cancer (CRPC), further leading to tumor metastasis to distant organs. Therefore, identifying the key molecular mechanisms underlying PCa progression remains crucial for the development of novel therapies for metastatic PCa. Previously, we identified that tumor-suppressive miR-99b-5p is frequently downregulated in aggressive African American (AA) PCa and European American (EA) CRPC, leading to upregulation of mTOR, androgen receptor (AR), and HIF-1α signaling. Given the fact that mTOR and HIF-1α signaling are critical upstream pathways that trigger the activation of epithelial-mesenchymal transition (EMT), we hypothesized that miR-99b-5p may play a critical functional role in regulating EMT-mediated PCa metastasis. To test this hypothesis, a series of cell biology, biochemical, and in vitro functional assays (wound healing, transwell migration, cell/ECM adhesion, and capillary-like tube formation assays) were performed to examine the effects of miR-99b-5p mimic on regulating EMT-mediated PCa metastasis processes. Our results have demonstrated that miR-99b-5p simultaneously targets MTOR and AR signaling, leading to upregulation of E-cadherin, downregulation of Snail/N-cadherin/Vimentin, and suppression of EMT-mediated PCa metastasis. MiR-99b-5p alone and in combination with enzalutamide or abiraterone significantly inhibits the EMT-mediated metastasis of AA PCa and EA CRPC.

Keywords:

AA PCa, AR signaling, CRPC, EMT, PI3K/AKT/mTOR signaling, abiraterone, enzalutamide, metastasis, miR-99b-5p, prostate cancer

<u>Original Lay Summary</u>

To date, effective treatment for metastatic prostate cancer (PCa) remains a clinical challenge. Our previous study demonstrated that tumor suppressive miR-99b-5p negatively regulates the expression of androgen receptor (AR) and mTOR, potentially serving as a therapeutic agent for aggressive PCa. In this study, we show for the first time that combination of miR-99b-5p mimic and second-generation AR antagonist (such as enzalutamide or abiraterone) simultaneously targets AR and mTOR signaling, consequently inhibiting epithelial-mesenchymal transition (EMT) in PCa. This novel combination therapy may provide a unique opportunity for effectively suppressing EMT-mediated metastasis in aggressive PC, especially African American PCa and castration-resistant prostate cancer (CRPC).

Comprehension Score	Explanation
5	The lay summary is factually accurate and fully consistent with the abstract. It correctly states that miR-99b-5p suppresses AR and mTOR signaling, and that the combination with second-generation AR antagonists inhibits EMT in prostate cancer. It reflects that this is a novel finding ("for the first time"). It highlights the relevance to aggressive prostate cancer, just as the original study specifies. There are no factual errors or misleading simplifications. All essential points are covered.

Lay Summary (Simple Prompt)

Prostate cancer is a common and life-threatening disease in men. While standard treatments that reduce male hormones (androgen deprivation therapy) can slow cancer growth, many patients eventually develop a more aggressive form called castration-resistant prostate cancer (CRPC), which spreads to other parts of the body. This study explores a potential new approach to slow the spread of prostate cancer by using a small molecule called miR-99b-5p. Researchers found that miR-99b-5p can block key signals that drive cancer cell movement, helping to prevent metastasis. When combined with existing treatments like enzalutamide or abiraterone, miR-99b-5p further enhances the suppression of cancer spread. These findings suggest that miR-99b-5p could be a promising addition to current therapies, offering new hope for patients with advanced prostate cancer. Further research is needed to explore its full potential in clinical settings.

Comprehension Score	Explanation
4	The lay summary is factually accurate and aligns well with the abstract. It correctly explains the clinical context of prostate cancer and CRPC, and the potential role of miR-99b-5p in blocking signals that drive metastasis. It mentions the synergistic effect when combined with enzalutamide or abiraterone. However, it simplifies some mechanistic details (e.g., it says “block key signals” without specifying AR and mTOR signaling or EMT) and does not explicitly mention the focus on African American and European American populations. It communicates the main findings clearly but omits some detailed specifics from the abstract.

<u>Lay Summary (Extended Prompt)</u>

Prostate cancer is a common and serious disease that can spread to other parts of the body, making treatment difficult. Many patients receive hormone-based therapies like enzalutamide or abiraterone to slow cancer growth, but over time, the cancer often becomes resistant and continues to spread. This study explores the role of a small molecule called miR-99b-5p, which is naturally found in the body but is often missing in aggressive prostate cancer. Researchers found that restoring miR-99b-5p helps block key pathways that drive cancer spread. When combined with existing treatments, miR-99b-5p further reduces cancer cells' ability to move and invade new areas. These findings suggest that adding miR-99b-5p to current therapies could improve treatment outcomes for patients with advanced prostate cancer and potentially slow down or prevent its spread. This discovery may lead to better treatment options in the future.

Comprehension Score	Explanation
4	The summary is factually accurate and consistent with the scientific abstract. It correctly describes prostate cancer's progression, resistance to hormone therapies, and the potential role of miR-99b-5p in suppressing metastatic pathways. However, some detailed mechanisms are simplified, and the focus on African American and European American populations is omitted. Furthermore, specific experimental approaches are omitted. Overall, it provides a clear and accurate overview but with minor omissions.
