# Supplementary material for: Using ChatGPT-4 for Lay Summarization in Prostate Cancer Research to Advance Patient-Centered Communication: Large-Scale Generative AI Performance Evaluation
Source: J Med Internet Res. 2025 Nov 19;27:e76598. doi: 10.2196/76598 (PMC12629520; doi:10.2196/76598)
Supplement: Multimedia Appendix 4 [file jmir-v27-e76598-s004.pdf]

# *Multimedia Appendix 4*

## Advancing Patient-Centered Communication with Generative AI: A Large-Scale Evaluation of ChatGPT-4 for Lay Summarization in Prostate Cancer Research

Emily Rinderknecht[1,2,*], Simon Engelmann[1], Veronika Saberi[1], Clemens Kirschner[1], Anton P. Kravchuk[3], Anna Schmelzer[4], Johannes Breyer[1], Christopher Goßler[1], Roman Mayr[1], Christian Gilfrich[3], Maximilian Burger[1], Dominik von Winning[3], Hendrik Borgmann[2,5], Christian Wülfing[2,6], Axel S. Merseburger[7], Maximilian Haas[1,#], and Matthias May[2,3,#]

[1] Department of Urology, University of Regensburg, Caritas St. Josef Medical Center, 93053 Regensburg, Germany.

[2] Working Group on Artificial Intelligence and Digitalization of the German Society of Urology.

[3] Department of Urology, St. Elisabeth Hospital Straubing, 94315 Straubing, Germany.

[4] Department of Urology, Nuremberg General Hospital, Paracelsus Medical University, 90419 Nuremberg, Germany.

[5] Department of Urology, Brandenburg Medical School Theodor Fontane (MHB), 14770 Brandenburg an der Havel, Germany.

[6] Department of Urology, Asklepios Klinik Altona, 22763 Hamburg, Germany.

[7] Department of Urology, University Hospital Schleswig-Holstein, Campus Lübeck, 23562 Lübeck, Germany.

[#] Shared senior-authors / These authors contributed equally to this work.

[*] Correspondence: erinderknecht@csj.de

**Multimedia Appendix 4**

Supplementary Tables concerning evaluation of lay summaries stratified into clinical, basic, and translational research.

*Supplementary Table 1:* Descriptive data regarding length metrics, readability scores, and factual accuracy for the original lay summaries and the lay summaries created by ChatGPT-4 (simple prompt) and ChatGPT-4 (extended prompt) with regards to clinical research articles, respectively.

| **Parameter** | **Original Lay summary** | **ChatGPT-4 Simple Prompt** | **ChatGPT-4 Extended Prompt** | **Standardized Test Statistic (Z-Values)** | ***P*-Values** |
|---|---|---|---|---|---|
| **Text metrics** | | | | | |
| Sentences; median (IQR) | 5 (4-7) | 6 (6-7) | 7 (6-8) | [a] 4.062[d]<br>[b] 3.425[d]<br>[c] 4.388[d] | [a, b, c] **<.001** |
| Words; median (IQR) | 116 (91-142) | 140 (133-144) | 141 (135-146) | [a] .742[d]<br>[b] 5.692[d]<br>[c] 5.487[d] | [a] .46; [b, c] **<.001** |
| Complex words; median (IQR) | 31 (23-39) | 27 (22-32) | 21 (16-25) | [a] 8.018[e]<br>[b] 3.905[e]<br>[c] 7.676[e] | [a, b, c] **<.001** |
| Percent of complex words; median (IQR) | 27 (23-31) | 20 (16-23) | 16 (12-18) | [a] 8.036[e]<br>[b] 7.709[e]<br>[c] 8.677[e] | [a, b, c] **<.001** |
| Average words per sentence; median (IQR) | 22 (18-26) | 22 (20-23) | 20 (19-22) | [a] 4.573[e]<br>[b] .386[e]<br>[c] 2.046[e] | [a] **<.001**; [b] .70; [c] **.04** |
| Average syllables per word; median (IQR) | 1.9 (1.9-2) | 1.8 (1.7-1.9) | 1.7 (1.6-1.7) | [a] 7.951[e]<br>[b] 7.336[e]<br>[c] 8.578[e] | [a, b, c] **<.001** |
| **Readability Scores** | | | | | |
| Flesch–Kincaid Reading Ease (FKRE); median (IQR) | 20 (10-29) | 34 (28-42) | 45 (42-54) | [a] 8.384[d]<br>[b] 7.370[d]<br>[c] 8.576[d] | [a, b, c] **<.001** |
| Flesch–Kincaid Grade Level; median (IQR) | 16 (14-18) | 14 (13-15) | 12 (11-13) | [a] 8.132[e]<br>[b] 6.303[e]<br>[c] 8.210[e] | [a, b, c] **<.001** |
| Gunning Fog Score; median (IQR) | 19 (17-22) | 16 (15-18) | 14 (13-15) | [a] 8.165[e]<br>[b] 6.898[e]<br>[c] 8.410[e] | [a, b, c] **<.001** |
| Smog Index; median (IQR) | 14 (12-15) | 12 (11-13) | 10 (9-11) | [a] 8.165[e]<br>[b] 6.977[e]<br>[c] 8.468[e] | [a, b, c] **<.001** |
| Coleman–Liau Index; median (IQR) | 18 (16-20) | 17 (16-18) | 15 (14-16) | [a] 7.535[e]<br>[b] 2.370[e]<br>[c] 6.917[e] | [b] **.02**; [a, c] **<.001** |
| Automated Readability Index; median (IQR) | 17 (14-19) | 16 (15-17) | 14 (12-15) | [a] 7.847[e]<br>[b] 2.087[e]<br>[c] 5.869[e] | [b] **.04**; [a, c] **<.001** |

| | | | | | | |
|---|---|---|---|---|---|---|
| Reading age in years; median (IQR) | | 23 (21-24) | 21 (20-22) | 19 (18-20) | [a] 7.977[e]<br>[b] 4.756[e]<br>[c] 7.686[e] | **[a, b, c] <.001** |
| **Assessment of factual accuracy, readability (FKRE), and correct wordcount** | | | | | | |
| Factual accuracy Score 1 (performed by M. M.) | | | | | [a] .277[d]<br>[b] 2.647[d]<br>[c] 2.806[d] | [a] .78; **[b] .008; [c] .005** |
| 1 point; n (%) | | 0 (0) | 0 (0) | 0 (0) | | |
| 2 points; n (%) | | 0 (0) | 0 (0) | 0 (0) | | |
| 3 points; n (%) | | 7 (6.9) | 1 (1.0) | 1 (1.0) | | |
| 4 points; n (%) | | 28 (28) | 21 (21) | 20 (20) | | |
| 5 points; n (%) | | 66 (65) | 79 (78) | 80 (80) | | |
| Median (IQR) | | 5 (4-5) | 5 (5-5) | 5 (5-5) | | |
| Factual accuracy Score 2 (performed by J. B.) | | | | | [a] 1.500[d]<br>[b] 3.492[d]<br>[c] 4.377[d] | [a] .13; **[b, c] <0.001** |
| 1 point; n (%) | | 0 (0) | 0 (0) | 0 (0) | | |
| 2 points; n (%) | | 0 (0) | 0 (0) | 0 (0) | | |
| 3 points; n (%) | | 12 (12) | 4 (4.0) | 2 (2.0) | | |
| 4 points; n (%) | | 33 (33) | 18 (18) | 16 (16) | | |
| 5 points; n (%) | | 56 (55) | 79 (78) | 83 (82) | | |
| Median (IQR) | | 5 (4-5) | 5 (5-5) | 5 (5-5) | | |
| Factual accuracy Scores; overall evaluation | | | | | [a] .816[e]<br>[b] 2.329[e]<br>[c] 2.653[e] | [a] .41; **[b] .02; [c] .008** |
| 1 Rating < 4; n (%) | | 9 (8.9) | 3 (3.0) | 1 (1.0) | | |
| 2 Ratings < 4; n (%) | | 5 (5.0) | 1 (1.0) | 1 (1.0) | | |
| FKRE | | | | | [a] 5.336[e]<br>[b] 6.823[e]<br>[c] 7.985[e] | **[a, b, c] <0.001** |
| FKRE 29.9-20; n (%) | | 33 (33) | 26 (26) | 1 (1.0) | | |
| FKRE < 20; n (%) | | 47 (47) | 7 (6.9) | 1 (1.0) | | |
| Wrong number of words; n (%) | | 75 (74) | 16 (16) | 19 (19) | [a] .655[d]<br>[b] 7.318[e]<br>[c] 6.893[e] | [a] .51; **[b, c] <0.001** |
| **Overall quality assessment** | | | | | [a] 3.960[e]<br>[b] 7.979[e]<br>[c] 8.332[e] | **[a, b, c] <.001** |
| High-quality | 0 points; n (%) | 5 (5.0) | 54 (54) | 80 (79) | | |
| Minor limitations | 1 point; n (%) | 20 (20) | 35 (35) | 18 (18) | | |
| | 2 points; n (%) | 36 (36) | 10 (9.9) | 2 (2.0) | | |
| Moderate limitations | 3 points; n (%) | 33 (33) | 2 (2.0) | 1 (1.0) | | |
| Major limitations | 4 points; n (%) | 5 (5.0) | 0 (0) | 0 (0) | | |
| | 5 points; n (%) | 2 (2.0) | 0 (0) | 0 (0) | | |

Bold letters indicate statistical significance. Total n = 101.

[a] ChatGPT-4 Simple Prompt vs. ChatGPT-4 Extended Prompt.

[b] Original Lay Summary vs. ChatGPT-4 Simple Prompt.

[c] Original Lay Summary vs. ChatGPT-4 Extended Prompt.

[d] Wilcoxon Signed Ranks Test based on negative ranks.

[e] Wilcoxon Signed Ranks Test based on positive ranks.

*Supplementary Table 2:* Descriptive data regarding length metrics, readability scores, and factual accuracy for the original lay summaries and the lay summaries created by ChatGPT-4 (simple prompt) and ChatGPT-4 (extended prompt) with regards to basic research articles, respectively.

| **Parameter** | **Original Lay summary** | **ChatGPT-4 Simple Prompt** | **ChatGPT-4 Extended Prompt** | **Standardized Test Statistic (Z-Values)** | ***P*-Values** |
|---|---|---|---|---|---|
| | | | **Text metrics** | | |
| Sentences; median (IQR) | 6 (5-7) | 7 (6-7) | 7 (6-7) | [a] 2.604[d]<br>[b] .947[d]<br>[c] 2.339[d] | **[a] .009;** [b] 0.34;<br>**[c] .02** |
| Words; median (IQR) | 135 (116-150) | 141 (130-145) | 137 (129-145) | [a] .474[e]<br>[b] 1.262[d]<br>[c] .723[d] | [a] .64; [b] .21;<br>[c] .47 |
| Complex words; median (IQR) | 36 (27-40) | 23 (19-27) | 17 (12-20) | [a] 4.969[e]<br>[b] 5.006[e]<br>[c] 5.235[e] | **[a, b, c] <.001** |
| Percent of complex words; median (IQR) | 27 (23-31) | 16 (14-20) | 12 (9-15) | [a] 4.996[e]<br>[b] 5.090[e]<br>[c] 5.232[e] | **[a, b, c] <.001** |
| Average words per sentence; median (IQR) | 21 (13-25) | 21 (20-24) | 20 (18-21) | [a] 3.240[e]<br>[b] .503[e]<br>[c] 1.995[e] | **[a] <.001; [b]**.62;<br>**[c] .046** |
| Average syllables per word; median (IQR) | 1.9 (1.8-2.1) | 1.7 (1.6-1.8) | 1.6 (1.5-1.7) | [a] 5.091[e]<br>[b] 4.948[e]<br>[c] 5.233[e] | **[a, b, c] <.001** |
| | | | **Readability Scores** | | |
| Flesch–Kincaid Reading Ease (FKRE); median (IQR) | 22 (7.6-34) | 41 (34-47) | 52 (45-59) | [a] 5.185[d]<br>[b] 4.949[d]<br>[c] 5.232[d] | **[a, b, c] <.001** |
| Flesch–Kincaid Grade Level; median (IQR) | 16 (13-18) | 13 (12-14) | 11 (10-12) | [a] 5.091[e]<br>[b] 4.479[e]<br>[c] 5.216 | **[a, b, c] <.001** |
| Gunning Fog Score; median (IQR) | 19 (18-22) | 15 (14-16) | 13 (12-14) | [a] 5.037[e]<br>[b] 4.965[e]<br>[c] 5.232[e] | **[a, b, c] <.001** |
| Smog Index; median (IQR) | 14 (12-16) | 11 (10-12) | 9 (8-10) | [a] 5.046[e]<br>[b] 5.013[e]<br>[c] 5.232[e] | **[a, b, c] <.001** |
| Coleman–Liau Index; median (IQR) | 18 (16-19) | 17 (15-18) | 15 (13-16) | [a] 5.145[e]<br>[b] 2.884[e]<br>[c] 4.793[e] | **[b] .004;**<br>**[a, c] <.001** |
| Automated Readability Index; median (IQR) | 17 (14-19) | 15 (14-17) | 13 (12-14) | [a] 5.212[e]<br>[b] 2.074[e]<br>[c] 4.965[e] | **[b] .04; [a, c] <.001** |

| | | | | | | |
|---|---|---|---|---|---|---|
| Reading age in years; median (IQR) | | 22 (21-24) | 20 (19-21) | 18 (17-19) | [a] 5.000[e]<br>[b] 4.581[e]<br>[c] 5.243[e] | **[a, b, c] <.001** |
| **Assessment of factual accuracy, readability (FKRE), and correct wordcount** | | | | | | |
| Factual accuracy Score 1 (performed by M. M.) | | | | | [a] 1.414[e]<br>[b] .816[d]<br>[c] .000 | [a] .16; [b] 0.41;<br>[c] 1.00 |
| 1 point; n (%) | | 0 (0) | 0 (0) | 0 (0) | | |
| 2 points; n (%) | | 0 (0) | 0 (0) | 0 (0) | | |
| 3 points; n (%) | | 0 (0) | 0 (0) | 0 (0) | | |
| 4 points; n (%) | | 5 (14) | 3 (8.3) | 5 (14) | | |
| 5 points; n (%) | | 31 (86) | 33 (92) | 31 (86) | | |
| Median (IQR) | | 5 (5-5) | 5 (5-5) | 5 (5-5) | | |
| Factual accuracy Score 2 (performed by J. B.) | | | | | [a] .000<br>[b] 1.342<br>[c] 1.000[d] | [a] 1.00; [b] 0.18;<br>[c] 0.32 |
| 1 point; n (%) | | 0 (0) | 0 (0) | 0 (0) | | |
| 2 points; n (%) | | 0 (0) | 0 (0) | 0 (0) | | |
| 3 points; n (%) | | 0 (0) | 0 (0) | 0 (0) | | |
| 4 points; n (%) | | 9 (25) | 6 (17) | 6 (17) | | |
| 5 points; n (%) | | 27 (75) | 30 (83) | 30 (83) | | |
| Median (IQR) | | 5 (4-5) | 5 (5-5) | 5 (5-5) | | |
| Factual accuracy Scores; overall evaluation | | | | | [a] .000<br>[b] .000<br>[c] .000 | [a, b, c] 1.00 |
| 1 Rating < 4; n (%) | | 0 (0) | 0 (0) | 0 (0) | | |
| 2 Ratings < 4; n (%) | | 0 (0) | 0 (0) | 0 (0) | | |
| FKRE | | | | | [a] 2.000[e]<br>[b] 4.196[e]<br>[c] 4.420[e] | **[a] 0.046; [b, c] <0.001** |
| FKRE 29.9-20; n (%) | | 6 (17) | 4 (11) | 0 (0) | | |
| FKRE < 20; n (%) | | 17 (47) | 0 (0) | 0 (0) | | |
| Wrong number of words; n (%) | | 19 (53) | 7 (19) | 6 (17) | [a] .447[e]<br>[b] 2.683[e]<br>[c] 2.837[e] | [a] .66; **[b] 0.007;**<br>**[c] .005** |
| **Overall quality assessment** | | | | | [a] 1.667[e]<br>[b] 4.553[e]<br>[c] 4.951[e] | [a] .10; **[b, c] <.001** |
| High-quality | 0 points; n (%) | 3 (8.3) | 25 (69) | 30 (84) | | |
| Minor limitations | 1 point; n (%) | 14 (39) | 11 (31) | 6 (17) | | |
| | 2 points; n (%) | 12 (33) | 0 (0) | 0 (0) | | |
| Moderate limitations | 3 points; n (%) | 7 (19) | 0 (0) | 0 (0) | | |
| Major limitations | 4 points; n (%) | 0 (0) | 0 (0) | 0 (0) | | |
| | 5 points; n (%) | 0 (0) | 0 (0) | 0 (0) | | |

Bold letters indicate statistical significance. Total n = 36.
[a] ChatGPT-4 Simple Prompt vs. ChatGPT-4 Extended Prompt.
[b] Original Lay Summary vs. ChatGPT-4 Simple Prompt.
[c] Original Lay Summary vs. ChatGPT-4 Extended Prompt.
[d] Wilcoxon Signed Ranks Test based on negative ranks.
[e] Wilcoxon Signed Ranks Test based on positive ranks.

*Supplementary Table 3:* Descriptive data regarding length metrics, readability scores, and factual accuracy for the original lay summaries and the lay summaries created by ChatGPT-4 (simple prompt) and ChatGPT-4 (extended prompt) with regards to translational research articles, respectively.

| Parameter | Original Lay summary | ChatGPT-4 Simple Prompt | ChatGPT-4 Extended Prompt | Standardized Test Statistic (Z-Values) | *P*-Values |
|---|---|---|---|---|---|
| **Text metrics** | | | | | |
| Sentences; median (IQR) | 5 (4-6) | 6 (5-7) | 7 (6-7) | [a] 2.727[d]<br>[b] 3.083[d]<br>[c] 4.162[d] | **[a] .006; [b] .002; [c] <.001** |
| Words; median (IQR) | 111 (95-136) | 136 (123-143) | 136 (126-144) | [a] .507[e]<br>[b] 3.996[d]<br>[c] 4.008[d] | [a] .61; **[b, c] <.001** |
| Complex words; median (IQR) | 29 (23-136) | 27 (21-30) | 19 (15-25) | [a] 6.789[e]<br>[b] 2.792[e]<br>[c] 6.177[e] | **[b] .005; [a, c] <.001** |
| Percent of complex words; median (IQR) | 27 (23-31) | 20 (16-23) | 14 (11-18) | [a] 7.021[e]<br>[b] 6.299[e]<br>[c] 7.084[e] | **[a, b, c] <.001** |
| Average words per sentence; median (IQR) | 22 (20-25) | 22 (20-24) | 21 (20-22) | [a] 2.855[e]<br>[b] .722[e]<br>[c] 2.546[e] | **[a] 0.004;** [b] .47; **[c] .01** |
| Average syllables per word; median (IQR) | 1.9 (1.9-2.0) | 1.8 (1.7-1.9) | 1.6 (1.5-1.7) | [a] 6.964[e]<br>[b] 6.097[e]<br>[c] 7.035[e] | **[a, b, c] <.001** |
| **Readability Scores** | | | | | |
| Flesch–Kincaid Reading Ease (FKRE); median (IQR) | 19 (7.7-28) | 34 (28-40) | 47 (41-57) | [a] 7.040[d]<br>[b] 6.232[d]<br>[c] 7.090[d] | **[a, b, c] <.001** |
| Flesch–Kincaid Grade Level; median (IQR) | 16 (15-18) | 14 (13-15) | 12 (10-13) | [a] 6.722[e]<br>[b] 5.431[e]<br>[c] 6.928[e] | **[a, b, c] <.001** |
| Gunning Fog Score; median (IQR) | 19 (18-22) | 17 (16-18) | 14 (12-16) | [a] 6.804[e]<br>[b] 5.346[e]<br>[c] 6.803[e] | **[a, b, c] <.001** |
| Smog Index; median (IQR) | 14 (13-16) | 12 (11-13) | 9.9 (8.6-11) | [a] 6.815[e]<br>[b] 5.889[e]<br>[c] 7.019[e] | **[a, b, c] <.001** |
| Coleman–Liau Index; median (IQR) | 18 (17-20) | 17 (16-18) | 15 (14-16) | [a] 6.979[e]<br>[b] 3.379[e]<br>[c] 6.747[e] | **[a, b, c] <.001** |
| Automated Readability Index; median (IQR) | 17 (15-19) | 16 (15-17) | 14 (12-15) | [a] 6.459[e]<br>[b] 2.412[e]<br>[c] 6.147[e] | **[b] .02; [a, c] <.001** |

| | | | | | | |
|---|---|---|---|---|---|---|
| Reading age in years; median (IQR) | | 23 (22-24) | 21 (20-22) | 19 (18-20) | [a] 6.657[e]<br>[b] 5.075[e]<br>[c] 6.869[e] | **[a, b, c] <.001** |
| **Assessment of factual accuracy, readability (FKRE), and correct wordcount** | | | | | | |
| Factual accuracy Score 1 (performed by M. M.) | | | | | [a] .707[e]<br>[b] 2.954[d]<br>[c] 2.541[d] | [a].48; **[b] .003;**<br>**[c] .01** |
| 1 point; n (%) | | 0 (0) | 0 (0) | 0 (0) | | |
| 2 points; n (%) | | 0 (0) | 0 (0) | 0 (0) | | |
| 3 points; n (%) | | 8 (12) | 0 (0) | 0 (0) | | |
| 4 points; n (%) | | 12 (18) | 11 (16) | 13 (19) | | |
| 5 points; n (%) | | 47 (70) | 56 (84) | 54 (81) | | |
| Median (IQR) | | 5 (4-5) | 5 (5-5) | 5 (5-5) | | |
| Factual accuracy Score 2 (performed by J. B.) | | | | | [a] 707[d]<br>[b] 3.111[d]<br>[c] 3.201[d] | [a] .48; **[b] .002;**<br>**[c] .001** |
| 1 point; n (%) | | 0 (0) | 0 (0) | 0 (0) | | |
| 2 points; n (%) | | 2 (3.0) | 0 (0) | 0 (0) | | |
| 3 points; n (%) | | 8 (12) | 0 (0) | 0 (0) | | |
| 4 points; n (%) | | 13 (19) | 14 (21) | 12 (18) | | |
| 5 points; n (%) | | 44 (66) | 53 (79) | 55 (82) | | |
| Median (IQR) | | 5 (4-5) | 5 (5-5) | 5 (5-5) | | |
| Factual accuracy Scores; overall evaluation | | | | | [a] .000<br>[b] 3.035[e]<br>[c] 3.035[e] | [a] 1.00; **[b] .002;**<br>**[c] .002** |
| 1 Rating < 4; n (%) | | 4 (6.0) | 0 (0) | 0 (0) | | |
| 2 Ratings < 4; n (%) | | 7 (10) | 0 (0) | 0 (0) | | |
| FKRE | | | | | [a] 4.217[e]<br>[b] 5.811[e]<br>[c] 6.650[e] | **[a, b, c] <0.001** |
| FKRE 29.9-20; n (%) | | 20 (30) | 20 (30) | 2 (3.0) | | |
| FKRE < 20; n (%) | | 35 (52) | 3 (4.5) | 0 (0) | | |
| Wrong number of words; n (%) | | 46 (69) | 17 (25) | 14 (21) | [a] .832<br>[b] 4.902[e]<br>[c] 5.060[e] | [a] .41; **[b, c] <0.001** |
| **Overall quality assessment** | | | | | [a] 3.894[e]<br>[b] 6.535[e]<br>[c] 6.709[e] | **[a, b, c] <.001** |
| High-quality | 0 points; n (%) | 3 (4.5) | 33 (49) | 51 (76) | | |
| Minor limitations | 1 point; n (%) | 14 (21) | 26 (39) | 16 (24) | | |
| | 2 points; n (%) | 20 (30) | 7 (10) | 0 (0) | | |
| Moderate limitations | 3 points; n (%) | 24 (36) | 1 (1.5) | 0 (0) | | |
| Major limitations | 4 points; n (%) | 2 (3.0) | 0 (0) | 0 (0) | | |
| | 5 points; n (%) | 4 (6.0) | 0 (0) | 0 (0) | | |

Bold letters indicate statistical significance. Total n = 67.

[a] ChatGPT-4 Simple Prompt vs. ChatGPT-4 Extended Prompt.

[b] Original Lay Summary vs. ChatGPT-4 Simple Prompt.

[c] Original Lay Summary vs. ChatGPT-4 Extended Prompt.

[d] Wilcoxon Signed Ranks Test based on negative ranks.

[e] Wilcoxon Signed Ranks Test based on positive ranks.

*Supplementary Table 4:* Group differences among basic, clinical, and translational research articles regarding length metrics, readability scores, and factual accuracy for the original lay summaries and the lay summaries created by ChatGPT-4 (simple prompt) and ChatGPT-4 (extended prompt).

| **Parameter** | **Original Lay Summary** | | **ChatGPT-4 Simple Prompt** | | **ChatGPT-4 Extended Prompt** | |
|---|---|---|---|---|---|---|
| | **Standardized Test Statistic (Z-Values)[a]** | ***P*-Values** | **Standardized Test Statistic (Z-Values)[a]** | ***P*-Values** | **Standardized Test Statistic (Z-Values)[a]** | ***P*-Values** |
| **Text metrics** | | | | | | |
| Sentences | 6.149 | **.046** | 5.322 | .07 | 6.949 | **.03** |
| Words | 9.072 | **.01** | 4.414 | .11 | 6.751 | **.03** |
| Complex words | 4.850 | .09 | 11.672 | **.003** | 10.880 | **.004** |
| Percent of complex words | .008 | .996 | 14.133 | **<.001** | 9.425 | **.009** |
| Average words per sentence | .631 | .73 | .966 | .62 | 1.646 | .44 |
| Average syllables per word | .549 | .76 | 10.909 | **.004** | 7.199 | **.03** |
| **Readability Scores** | | | | | | |
| Flesch–Kincaid Reading Ease (FKRE) | .828 | .66 | 12.701 | **.002** | 7.644 | **.02** |
| Flesch–Kincaid Grade Level | .828 | .66 | 11.602 | **.003** | 7.674 | **.02** |
| Gunning Fog Score | .339 | .84 | 13.884 | **<.001** | 8.603 | **.01** |
| Smog Index | .686 | .71 | 15.184 | **<.001** | 10.282 | **.006** |
| Coleman–Liau Index | 1.746 | .42 | 2.948 | .38 | 1.773 | .412 |
| Automated Readability Index | .795 | .67 | 3.470 | .18 | 1.975 | .373 |
| Reading age in years | 1.329 | .51 | 8.616 | **.01** | 6.218 | **.045** |
| **Assessment of factual accuracy, readability (FKRE), and correct wordcount** | | | | | | |
| Factual accuracy Score 1 (performed by M. M.) | 5.773 | .06 | 3.454 | .18 | .852 | .65 |
| Factual accuracy Score 2 (performed by J. B.) | 5.176 | .08 | .547 | .76 | .041 | .98 |
| Factual accuracy Scores; overall evaluation | 6.402 | **.04** | 4.140 | .13 | 2.050 | .36 |
| FKRE | 1.473 | .48 | 7.330 | **.03** | 1.070 | .59 |
| Wrong number of words | 5.660 | .06 | 2.311 | .31 | .282 | .87 |
| **Overall quality assessment** | 9.457 | **.009** | 5.298 | .07 | .705 | .70 |

Bold letters indicate statistical significance.

[a] Kruskal-Wallis Test for comparing 3 independent samples.

*Supplementary Table 5:* Post hoc comparisons for variables showing group differences among basic, clinical, and translational research articles regarding length metrics, readability scores, and factual accuracy for the original lay summaries.

| **Parameter** | **Translational vs. clinical research articles** | | **Translational vs. basic research articles** | | **Clinical vs. basic research articles** | |
|---|---|---|---|---|---|---|
| | **Standardized Test Statistic (Z-Values)[a]** | ***P*-Values** | **Standardized Test Statistic (Z-Values)[a]** | ***P*-Values** | **Standardized Test Statistic (Z-Values)[a]** | ***P*-Values** |
| **Text metrics** | | | | | | |
| Sentences | 1.230 | .66 | 2.477 | **.04** | 1.639 | .30 |
| Words | .797 | 1.0 | 2.947 | **.01** | 2.490 | **.04** |
| **Assessment of factual accuracy, readability (FKRE), and correct wordcount** | | | | | | |
| Factual accuracy Scores; overall evaluation | .593 | 1.000 | 2.458 | **.04** | 2.135 | .10 |
| **Overall quality assessment** | .468 | 1.000 | 2.916 | .01 | 2.724 | **.02** |

Bold letters indicate statistical significance.
[a] Dunn´s test with bonferroni correction.

*Supplementary Table 6:* Post hoc comparisons for variables showing group differences among basic, clinical, and translational research articles regarding length metrics, readability scores, and factual accuracy for the ChatGPT-4 simple prompt.

| **Parameter** | **Translational vs. clinical research articles** | | **Translational vs. basic research articles** | | **Clinical vs. basic research articles** | |
|---|---|---|---|---|---|---|
| | **Standardized Test Statistic (Z-Values)[a]** | ***P*-Values** | **Standardized Test Statistic (Z-Values)[a]** | ***P*-Values** | **Standardized Test Statistic (Z-Values)[a]** | ***P*-Values** |
| **Text metrics** | | | | | | |
| Complex words | .421 | 1.000 | 2.820 | **.01** | 3.344 | **.002** |
| Percent of complex words | .774 | 1.000 | 3.624 | **.001** | 3.230 | **.004** |
| Average syllables per word | .663 | 1.000 | 3.180 | **.004** | 2.847 | **.013** |
| **Readability Scores** | | | | | | |
| Flesch–Kincaid Reading Ease (FKRE) | .679 | 1.000 | 3.421 | **.002** | 3.090 | **.006** |
| Flesch–Kincaid Grade Level | 1.071 | .85 | 3.365 | **.002** | 2.713 | **.02** |
| Gunning Fog Score | 1.475 | .42 | 3.717 | .001 | 2.760 | .017 |
| Smog Index | 1.054 | .88 | 3.817 | <.001 | 3.208 | .004 |
| Reading age in years | 1.078 | .84 | 2.921 | **.01** | 2.234 | .08 |
| **Assessment of factual accuracy, readability (FKRE), and correct wordcount** | | | | | | |
| FKRE | .117 | 1.000 | 2.458 | .04 | 2.521 | **.04** |

Bold letters indicate statistical significance.
[a] Dunn´s test with bonferroni correction.

*Supplementary Table 7:* Post hoc comparisons for variables showing group differences among basic, clinical, and translational research articles regarding length metrics, readability scores, and factual accuracy for the ChatGPT-4 extended prompt.

| **Parameter** | **Translational vs. clinical research articles** | | **Translational vs. basic research articles** | | **Clinical vs. basic research articles** | |
|---|---|---|---|---|---|---|
| | **Standardized Test Statistic (Z-Values)[a]** | ***P*-Values** | **Standardized Test Statistic (Z-Values)[a]** | ***P*-Values** | **Standardized Test Statistic (Z-Values)[a]** | ***P*-Values** |
| **Text metrics** | | | | | | |
| Sentences | 2.486 | **.04** | 1.918 | .17 | .024 | 1.00 |
| Words | 2.562 | **.03** | .791 | 1.00 | 1.237 | .65 |
| Complex words | 1.923 | .17 | 1.500 | .40 | 3.158 | **.005** |
| Percent of complex words | 1.570 | .35 | 1.625 | .31 | 3.004 | **.008** |
| Average syllables per word | 1.336 | .55 | 1.455 | .44 | 2.634 | **.03** |
| **Readability Scores** | | | | | | |
| Flesch–Kincaid Reading Ease (FKRE) | 1.016 | .929 | 1.820 | .20 | 2.762 | **.02** |
| Flesch–Kincaid Grade Level | .549 | 1.00 | 2.162 | .09 | 2.747 | **.02** |
| Gunning Fog Score | .679 | 1.00 | 2.225 | .08 | 2.920 | **.01** |
| Smog Index | 1.040 | .89 | 2.219 | .08 | 3.207 | **.004** |
| Reading age in years | .893 | 1.00 | 1.660 | .291 | 2.492 | **.04** |

Bold letters indicate statistical significance.
[a] Dunn´s test with bonferroni correction.
