# Supplementary material for: Using ChatGPT-4 for Lay Summarization in Prostate Cancer Research to Advance Patient-Centered Communication: Large-Scale Generative AI Performance Evaluation
Source: J Med Internet Res. 2025 Nov 19;27:e76598. doi: 10.2196/76598 (PMC12629520; doi:10.2196/76598)
Supplement: Multimedia Appendix 3 [file jmir-v27-e76598-s003.pdf]

Multimedia Appendix 3

Advancing Patient-Centered Communication with Generative AI: A Large-Scale Evaluation of ChatGPT-4 for Lay Summarization in Prostate Cancer Research

Emily Rinderknecht[1,2,*], Simon Engelmann[1], Veronika Saberi[1], Clemens Kirschner[1], Anton P. Kravchuk[3], Anna Schmelzer[4], Johannes Breyer[1], Christopher Goßler[1], Roman Mayr[1], Christian Gilfrich[3], Maximilian Burger[1], Dominik von Winning[3], Hendrik Borgmann[2,5], Christian Wülfing[2,6], Axel S. Merseburger[7], Maximilian Haas[1,#], and Matthias May[2,3,#]

1 Department of Urology, University of Regensburg, Caritas St. Josef Medical Center, 93053 Regensburg, Germany.

2 Working Group on Artificial Intelligence and Digitalization of the German Society of Urology.

3 Department of Urology, St. Elisabeth Hospital Straubing, 94315 Straubing, Germany.

4 Department of Urology, Nuremberg General Hospital, Paracelsus Medical University, 90419 Nuremberg, Germany.

5 Department of Urology, Brandenburg Medical School Theodor Fontane (MHB), 14770 Brandenburg an der Havel, Germany.

6 Department of Urology, Asklepios Klinik Altona, 22763 Hamburg, Germany.

7 Department of Urology, University Hospital Schleswig-Holstein, Campus Lübeck, 23562 Lübeck, Germany.

# Shared senior-authors / These authors contributed equally to this work.

* Correspondence: erinderknecht@csj.de

Multimedia Appendix 3.1: Details of Included Articles, Abstracts, Keywords, and Original and ChatGPT-4-Generated Lay Summaries with Readability Metrics, Word Counts, and Comprehension Scores.

See on pages 3 to 953

Multimedia Appendix 3.2: Flowchart Depicting the Selection Process of Included Articles.

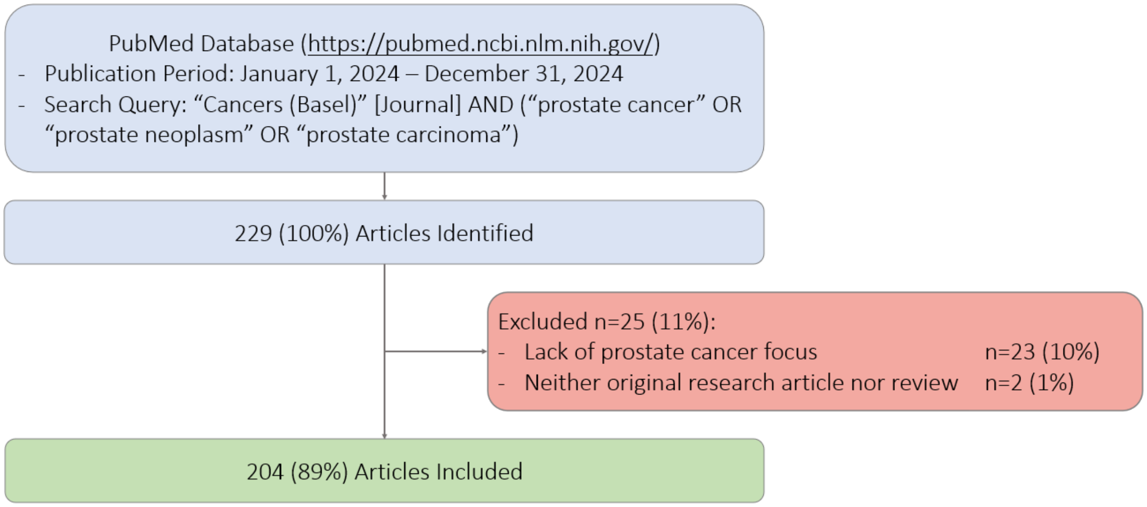

Manuscript 1

Gómez Rivas J, Ortega Polledo LE, De La Parra Sánchez I, Gutiérrez Hidalgo B, Martín Monterrubio J, Marugán Álvarez MJ, et al. Current Status of Neoadjuvant Treatment Before Surgery in High-Risk Localized Prostate Cancer. Cancers [Internet]. 2024 Dec 31;17(1):99. Available from: http://dx.doi.org/10.3390/cancers17010099

Abstract

Localized high-risk (HR) prostate cancer (PCa) is a heterogeneous disease whose likelihood of a biochemical recurrence, metastatic progression and cancer-related mortality after initial treatment is higher when compared with patients with low (LR) or intermediate-risk (IR) disease. In the past, neoadjuvant therapy has shown an improvement in postoperative oncological variables but failed to demonstrate any survival advantages. With the promising results from novel treatments in metastatic and non-metastatic castration resistant PCa settings, new evidence has appeared in the literature in the neoadjuvant setting. Background/Objectives: To describe the current evidence for different neoadjuvant treatments before a radical prostatectomy in high-risk prostate cancer. Methods: We performed a comprehensive English literature search for original and review articles through January-August 2024, using Pubmed, Medline and ClinicalTrials.gov databases, as well as a comprehensive review of different international guidelines, searching the following terms: "neoadjuvant ADT prostate cancer", "neoadjuvant ADT", "prostate cancer surgery" and "neoadjuvant high-risk prostate cancer". We included 61 papers for the final review. Results and Discussion: Neoadjuvant therapy is not recommended in daily practice by any international guideline. The National Comprehensive Cancer Network (NCCN) guidelines strongly discourage the use of ADT as a neoadjuvant therapy outside of clinical trials. ADT + ARTAs show promising data in phase-II trials, including favorable pCR, MRD, PSA relapse and salvage therapy rates. Clinical trials on chemotherapy, 177Lu-PSMA, genomic-targeted therapies and markers of response leave room for further evidence acquisition due to their encouraging results. Conclusions: Currently, no phase III data supports

systemic neoadjuvant therapy before RP. Phase II studies show promising data for ADT with second-generation agents, including favorable pCR, MRD, PSA relapse and salvage therapy rates.

Keywords:

ADT, ARTA, chemotherapy, high risk, lutetium, neoadjuvant, prostate cancer, radical prostatectomy, surgery

Original Lay Summary

The likelihood of a biochemical recurrence, metastatic progression and cancer-related mortality after the initial treatment in patients diagnosed with localized high-risk (HR) prostate cancer (PCa) is higher when compared with patients with low (LR) or intermediate-risk (IR) disease. The use of conventional androgen deprivation therapy (ADT) as a neoadjuvant therapy prior to radical prostatectomy (RP) has already been explored by several studies, showing a reduction in positive surgical margins and tumor volume but failing to demonstrate any survival advantages. However, novel agents such as androgen receptor-targeted agents (ARTAs) have exhibited significant survival benefits in both metastatic and non-metastatic castration-resistant PCa, as well as metastatic hormone-sensitive PCa. The significance of the pathological response following neoadjuvant hormonal therapy remains uncertain. In this narrative review, we cover the current status of neoadjuvant therapies before surgery in high-risk localized PCa.

**Assessment Category**	**Score**	**Assessment Category**	**Score**	**Assessment Category**	**Score**
Comprehension Score Rater 1	4	Gunning Fog Score	26.2	Words	144
Comprehension Score Rater 2	4	SMOG Index	18.7	Complex words	53
Reading Age (years)	24.0	Coleman Liau Index	19.1	Percentage of complex words	36.81
Flesch-Kincaid Reading Ease	-0.4	Automated Readability Index	20.9	Average words per sentence	28.8
Flesch-Kincaid Grade Level	20.5	Sentences	5	Average syllables per word	2.1

Lay Summary (Simple Prompt)

Prostate cancer is a common disease, and for men with high-risk localized prostate cancer, the chance of cancer returning after treatment is higher than in lower-risk cases. Some treatments given before surgery, known as neoadjuvant therapies, have been explored to improve outcomes. While past approaches have not shown clear survival benefits, new treatments originally developed for advanced prostate cancer are now being tested in earlier stages. This study reviewed the latest evidence on different neoadjuvant treatments before surgery, analyzing 61 studies from medical literature and clinical trials. Current international guidelines do not recommend these treatments in routine practice, but promising results have been seen in clinical trials with hormonal therapies, targeted treatments, chemotherapy, and radioactive drugs. While more research is needed, these findings suggest that new strategies may help improve surgery outcomes and reduce the risk of cancer returning in high-risk patients.

**Assessment Category**	**Score**	**Assessment Category**	**Score**	**Assessment Category**	**Score**
Comprehension Score Rater 1	4	Gunning Fog Score	18.2	Words	145
Comprehension Score Rater 2	4	SMOG Index	13.1	Complex words	31
Reading Age (years)	22.5	Coleman Liau Index	18.4	Percentage of complex words	21.38
Flesch-Kincaid Reading Ease	28.9	Automated Readability Index	18.0	Average words per sentence	24.17
Flesch-Kincaid Grade Level	15.2	Sentences	6	Average syllables per word	1.81

<u>Lay Summary (Extended Prompt)</u>

Prostate cancer can be more aggressive in some men, especially those with high-risk disease, meaning the cancer is more likely to return or spread after surgery. In other types of cancer, doctors sometimes use treatments before surgery—called neoadjuvant therapy—to shrink the tumor and improve outcomes. However, for prostate cancer, past studies have not shown a clear survival benefit. With new treatments showing success in advanced prostate cancer, researchers are now exploring whether these therapies might help high-risk patients before surgery. This study reviewed existing research on different neoadjuvant treatments, including hormone therapy, chemotherapy, and targeted therapies. While some early studies show promise, especially with advanced hormone treatments, there is no strong evidence yet from large clinical trials to support using these therapies before surgery. More research is needed to determine if these treatments can help improve long-term outcomes for men with high-risk prostate cancer.

**Assessment Category**	**Score**	**Assessment Category**	**Score**	**Assessment Category**	**Score**
Comprehension Score Rater 1	4	Gunning Fog Score	16.0	Words	148
Comprehension Score Rater 2	4	SMOG Index	11.6	Complex words	28
Reading Age (years)	20.5	Coleman Liau Index	18.1	Percentage of complex words	18.92
Flesch-Kincaid Reading Ease	37.3	Automated Readability Index	16.2	Average words per sentence	21.14
Flesch-Kincaid Grade Level	13.1	Sentences	7	Average syllables per word	1.75

Manuscript 2

Mazzucato G, Falkenbach F, Schmalhofer ML, Shenas F, Cerruto MA, Antonelli A, et al. Robot-Assisted PSMA-Radioguided Salvage Surgery for Oligorecurrent Prostate Cancer Using the Novel SENSEI® Drop-in Gamma Probe: Correlation of Intraoperative Measurements to Preoperative Imaging and Final Histology. Cancers [Internet]. 2024 Dec 31;17(1):93. Available from: http://dx.doi.org/10.3390/cancers17010093

Abstract

Background: To examine the feasibility and safety of the SENSEI® drop-in gamma probe for robot-assisted, prostate-specific membrane antigen (PSMA)-radioguided salvage surgery (RGS) in lymph node or local oligorecurrent prostate cancer (PCa), detected via PSMA positron emission tomography/computed tomography (PET/CT).

Methods: The first thirteen patients with pelvic oligorecurrent PCa who underwent [99mTc]Tc-PSMA-I&S RGS using the SENSEI® drop-in gamma probe at the Martini-Klinik (February-June 2024) were retrospectively analyzed. Radioactivity measurements in counts per second (CPS) as absolute values or ratios (CPS of tumor specimens/mean CPS from the patients' benign tissues) were correlated with preoperative imaging and pathological findings (benign/malignant, lesion size). Postoperative complete biochemical response (cBR) was defined as prostate-specific antigen (PSA) levels of <0.2 ng/mL.

Results: Fifty-four specimens were removed from 13 patients, with nineteen (35%) containing PCa. All patients had one PSMA PET/CT-positive lesion, which were all detected intraoperatively. These lesions showed higher ex vivo CPS, CPS ratios, and larger cancer diameters than PSMA PET/CT-negative lesions (all $p < 0.05$). Cancer-containing specimens exhibited higher CPS and CPS ratios than benign tissues (median values of 45 vs. 3, and 9.9 vs. 1.0, both $p < 0.001$). In total, 12/13 (92%) patients achieved cBR.

Conclusions: This device yielded excellent detection rates with good correlation to preoperative imaging and histological results without adverse events.

Keywords:

CPS count, biochemical recurrence, detection rate, metastasis-directed therapy, minimal invasive

<u>Original Lay Summary</u>

Salvage surgeries for prostate cancer are a challenging topic in this era of metastasis-directed therapy. Here, we present the first thirteen robot-assisted, prostate-specific membrane antigen-radioguided procedures in patients with oligorecurrent disease, performed using the SENSEI® drop-in gamma probe. The aim of this study was to assess the feasibility and safety of this new device, as well as to evaluate the oncologic outcome based on prostate-specific antigen post-discharge.

**Assessment Category**	**Score**	**Assessment Category**	**Score**	**Assessment Category**	**Score**
Comprehension Score Rater 1	4	Gunning Fog Score	19.1	Words	74
Comprehension Score Rater 2	3	SMOG Index	13.7	Complex words	17
Reading Age (years)	21.5	Coleman Liau Index	16.3	Percentage of complex words	22.97
Flesch-Kincaid Reading Ease	24.0	Automated Readability Index	16.6	Average words per sentence	24.67
Flesch-Kincaid Grade Level	16.0	Sentences	3	Average syllables per word	1.86

Lay Summary (Simple Prompt)

Prostate cancer can return after treatment, sometimes in only a few spots (oligorecurrence). Detecting and removing these cancerous lymph nodes or local recurrences precisely is crucial for better outcomes. This study explored a new tool, the SENSEI® drop-in gamma probe, used during robot-assisted surgery to locate and remove cancerous tissue marked with a special radioactive tracer. Thirteen patients underwent this procedure, and cancer was found in 35% of the removed tissue samples. The probe accurately identified cancerous areas, confirming preoperative imaging results and aligning with final laboratory findings. Importantly, 92% of patients had a significant drop in prostate-specific antigen (PSA) levels after surgery, suggesting effective cancer removal. This research highlights the potential of PSMA-radioguided surgery to improve treatment for recurrent prostate cancer, helping doctors detect and remove cancer more precisely while minimizing unnecessary tissue removal.

**Assessment Category**	**Score**	**Assessment Category**	**Score**	**Assessment Category**	**Score**
Comprehension Score Rater 1	4	Gunning Fog Score	18.6	Words	139
Comprehension Score Rater 2	4	SMOG Index	13.1	Complex words	37
Reading Age (years)	22.5	Coleman Liau Index	19.1	Percentage of complex words	26.62
Flesch-Kincaid Reading Ease	18.1	Automated Readability Index	16.5	Average words per sentence	19.86
Flesch-Kincaid Grade Level	15.7	Sentences	7	Average syllables per word	1.99

<u>Lay Summary (Extended Prompt)</u>

When prostate cancer comes back after treatment, it can sometimes spread to small areas in the body. Detecting and removing these small cancer spots is challenging. This study tested a new tool, the SENSEI® gamma probe, which helps surgeons find and remove these cancerous areas more accurately during robot-assisted surgery. Researchers used this tool on 13 patients and compared its real-time cancer detection ability to pre-surgery scans and final lab results. The probe successfully identified all cancerous areas seen on scans and helped surgeons remove them with high accuracy. Most patients (92%) had no signs of cancer in their blood after surgery. These results show that this new technique is safe, effective, and could improve how doctors treat recurring prostate cancer in a less invasive way. This could lead to better outcomes and fewer side effects for patients in the future.

**Assessment Category**	**Score**	**Assessment Category**	**Score**	**Assessment Category**	**Score**
Comprehension Score Rater 1	5	Gunning Fog Score	13.0	Words	144
Comprehension Score Rater 2	4	SMOG Index	9.4	Complex words	21
Reading Age (years)	17.5	Coleman Liau Index	13.9	Percentage of complex words	14.58
Flesch-Kincaid Reading Ease	54.0	Automated Readability Index	11.3	Average words per sentence	18.0
Flesch-Kincaid Grade Level	10.2	Sentences	8	Average syllables per word	1.59

Manuscript 3

Bhoir S, De Benedetti A. Beyond the Horizon: Rethinking Prostate Cancer Treatment Through Innovation and Alternative Strategies. Cancers [Internet]. 2024 Dec 29;17(1):75. Available from: http://dx.doi.org/10.3390/cancers17010075

Abstract

For nearly a century, fundamental observations that prostate cancer (PCa) cells nearly always require AR stimulation for sustained proliferation have led to a unidirectional quest to abrogate such a pathway. Similarly focused have been efforts to understand AR-driven processes in the context of elevated expression of its target genes, and much less so on products that become overexpressed when AR signaling is suppressed. Treatment with ARSI results in an increased expression of the TLK1B splice variant via a 'translational' derepression driven by the compensatory mTOR activation and consequent activation of the TLK1 > NEK1 > ATR > Chk1 and NEK1 > YAP axes. In due course, this results first in a pro-survival quiescence and then adaptation to ADT and CRPC progression. This constitutes a novel liability for PCa that we have targeted for several years and novel approaches.

Keywords:

J54, TLK1 inhibitor, androgen receptor signaling, prostate cancer (PCa), tousled-like kinase 1 (TLK1) pathway inhibition

Original Lay Summary

Prostate cancer (PCa) cells rely on androgen receptor (AR) signaling, and therapies targeting this pathway can inadvertently activate compensatory mechanisms like mTOR. This drives TLK1B overexpression, promoting survival and progression to castration-resistant prostate cancer (CRPC) via NEK1 > ATR > Chk1 and NEK1 > YAP pathways. Targeting this adaptation presents a novel therapeutic opportunity for CRPC.

**Assessment Category**	**Score**	**Assessment Category**	**Score**	**Assessment Category**	**Score**
Comprehension Score Rater 1	5	Gunning Fog Score	20.9	Words	57
Comprehension Score Rater 2	4	SMOG Index	14.5	Complex words	19
Reading Age (years)	22.5	Coleman Liau Index	18.7	Percentage of complex words	33.33
Flesch-Kincaid Reading Ease	16.9	Automated Readability Index	15.7	Average words per sentence	19.0
Flesch-Kincaid Grade Level	15.6	Sentences	3	Average syllables per word	2.02

Lay Summary (Simple Prompt)

Prostate cancer is often treated by blocking a key pathway that helps cancer cells grow. However, when this pathway is shut down, cancer cells can adapt and continue to survive, making treatment less effective over time. This study explores an alternative approach by targeting a different protein, TLK1, which becomes more active when traditional treatments suppress the usual cancer growth signals. Researchers have discovered that TLK1 plays a critical role in helping cancer cells enter a temporary dormant state before becoming resistant to treatment. By developing a new way to block TLK1, this study aims to prevent cancer cells from adapting and make current therapies more effective. These findings could lead to improved treatment strategies for prostate cancer, offering new hope for patients whose cancer no longer responds to standard therapies.

**Assessment Category**	**Score**	**Assessment Category**	**Score**	**Assessment Category**	**Score**
Comprehension Score Rater 1	5	Gunning Fog Score	15.1	Words	131
Comprehension Score Rater 2	5	SMOG Index	10.8	Complex words	21
Reading Age (years)	19.5	Coleman Liau Index	16.2	Percentage of complex words	16.03
Flesch-Kincaid Reading Ease	42.0	Automated Readability Index	15.1	Average words per sentence	21.83
Flesch-Kincaid Grade Level	12.8	Sentences	6	Average syllables per word	1.69

<u>Lay Summary (Extended Prompt)</u>

Prostate cancer is one of the most common cancers in men, and current treatments often target a key growth pathway driven by male hormones. However, cancer cells can adapt and become resistant to these treatments, making the disease harder to control. Our research explores a new approach by studying how prostate cancer cells change when this pathway is blocked. We found that a specific protein, TLK1B, becomes more active in response to treatment, helping cancer cells survive and grow despite therapy. By targeting this protein and its effects, we aim to develop new strategies to stop cancer from adapting and becoming resistant. This could lead to better treatments for patients with advanced prostate cancer and improve long-term outcomes. Our findings open new possibilities for tackling treatment resistance and may change how doctors approach prostate cancer therapy in the future.

**Assessment Category**	**Score**	**Assessment Category**	**Score**	**Assessment Category**	**Score**
Comprehension Score Rater 1	5	Gunning Fog Score	12.3	Words	140
Comprehension Score Rater 2	5	SMOG Index	8.6	Complex words	15
Reading Age (years)	17.5	Coleman Liau Index	15.2	Percentage of complex words	10.71
Flesch-Kincaid Reading Ease	51.2	Automated Readability Index	13.4	Average words per sentence	20.0
Flesch-Kincaid Grade Level	11.1	Sentences	7	Average syllables per word	1.6

Manuscript 4

Ladoukakis E, Oliver T, Wilks M, Lane EF, Chinegwundoh F, Shaw G, et al. Exploring the Link Between Obligate Anaerobe-Related Dysbiosis and Prostate Cancer Development: A Pilot Study. Cancers [Internet]. 2024 Dec 29;17(1):70. Available from: http://dx.doi.org/10.3390/cancers17010070

Abstract

Background/objectives: Several independent studies have associated prostate cancer (PCa) with specific groups of bacteria, most of them reporting the presence of anaerobic or microaerophilic species such as Cutibacterium acnes (C. acnes). Such findings suggest a prostate cancer-related bacterial dysbiosis, in a manner similar to the association between Helicobacter pylori infection and gastric cancer. In an earlier exploratory study looking for such dysbiosis events, using a culturomics approach, we discovered that the presence of obligate anaerobes (OAs) along with C. acnes was associated with increased prostate-specific antigen (PSA) levels in 39 participants.

Methods: Building on this, in this study, we analyzed 89 post-rectal examination urine samples, from men with prostate cancer attending the PROVENT trial, using 16S rDNA sequencing. Our investigation focused on the impact of six previously identified OA genera (Finegoldia, Fusobacterium, Prevotella, Peptoniphilus_A, Peptostreptococcus, and Veillonella_A) on PSA levels. However, an additional data-driven approach was followed to uncover more taxa linked to increased PSA.

Results: Our analysis revealed a statistically significant association between Peptostreptococcus and elevated PSA levels. Additionally, there were potential interactions between Prevotella and Fusobacterium. Interestingly, we also found that an aerobe, Ochrobactrum_A,was significantly linked to higher PSA levels.

Conclusions: These findings suggest that OA-related dysbiosis may contribute to

elevated PSA levels through prostate cell damage even before prostate cancer develops, possibly playing a role in chronic inflammation and the hypervascular changes seen in precancerous lesions. Future clinical trials with larger cohorts are needed to further evaluate the role of OA in prostate cancer development and progression.

Keywords:

anaerobic bacteria, circumcision, immune deficiency, prostate cancer

<u>Original Lay Summary</u>

Prostate cancer (PCa) is associated with the inflammation caused by anaerobic bacteria such as Cutibacterium acnes. In a previous exploratory study using 39 participants and a conventional bacterial culture approach (culturomics), we discovered a link between obligate anaerobic bacteria and increased prostate-specific antigen (PSA), a biomarker for prostate cancer risk. Our current study further validates this discovery by examining the microbial content in urine samples from 89 PCa patients using the more sensitive approach of 16s rDNA sequencing. The results show a clear association between specific obligate anaerobic bacteria such as Peptostreptococcus and increased PSA levels. Our findings indicate a potential contribution of obligate anaerobic bacteria in chronic inflammation that can lead to precancerous lesions, but larger cohorts are needed to further evaluate their role in prostate cancer development and progression.

**Assessment Category**	**Score**	**Assessment Category**	**Score**	**Assessment Category**	**Score**
Comprehension Score Rater 1	5	Gunning Fog Score	22.4	Words	132
Comprehension Score Rater 2	5	SMOG Index	16.5	Complex words	41
Reading Age (years)	24.0	Coleman Liau Index	19.4	Percentage of complex words	31.06
Flesch-Kincaid Reading Ease	0.7	Automated Readability Index	19.9	Average words per sentence	26.4
Flesch-Kincaid Grade Level	19.9	Sentences	5	Average syllables per word	2.14

Lay Summary (Simple Prompt)

Prostate cancer is a common disease among men, and recent research suggests that certain bacteria might play a role in its development. In this study, we focused on bacteria that thrive without oxygen, known as obligate anaerobes, to see if they are linked to changes in prostate health. We analyzed urine samples from men with prostate cancer to detect specific bacteria and examined how their presence affected prostate-specific antigen (PSA) levels, a marker often used to screen for prostate issues. We found that a particular type of bacteria, Peptostreptococcus, was significantly associated with higher PSA levels. We also observed potential interactions between other bacteria, such as Prevotella and Fusobacterium. Interestingly, we discovered that even an oxygen-loving bacterium, Ochrobactrum, was linked to increased PSA levels. These findings suggest that bacterial imbalances may contribute to early prostate changes, possibly leading to cancer, highlighting the need for further research in this area.

**Assessment Category**	**Score**	**Assessment Category**	**Score**	**Assessment Category**	**Score**
Comprehension Score Rater 1	5	Gunning Fog Score	16.3	Words	151
Comprehension Score Rater 2	5	SMOG Index	12.5	Complex words	33
Reading Age (years)	20.5	Coleman Liau Index	17.1	Percentage of complex words	21.85
Flesch-Kincaid Reading Ease	25.8	Automated Readability Index	15.7	Average words per sentence	21.57
Flesch-Kincaid Grade Level	15.0	Sentences	7	Average syllables per word	1.88

Lay Summary (Extended Prompt)

Prostate cancer is one of the most common cancers in men, but the exact causes are still not fully understood. Some studies have found links between certain bacteria and prostate cancer, especially bacteria that can only live without oxygen, called obligate anaerobes. In this pilot study, we looked at urine samples from men with prostate cancer to see if these bacteria might be connected to higher levels of prostate-specific antigen (PSA), a marker often used to detect prostate problems. We discovered that a specific bacterium, Peptostreptococcus, was linked to higher PSA levels. We also found possible connections with other bacteria, like Prevotella and Fusobacterium. Interestingly, we identified one oxygen-using bacterium, Ochrobactrum_A, that also seemed related to higher PSA. These findings suggest that certain bacteria might cause inflammation and changes in the prostate before cancer develops. More research is needed to confirm how these bacteria might influence prostate cancer risk.

**Assessment Category**	**Score**	**Assessment Category**	**Score**	**Assessment Category**	**Score**
Comprehension Score Rater 1	5	Gunning Fog Score	14.4	Words	151
Comprehension Score Rater 2	5	SMOG Index	11.2	Complex words	30
Reading Age (years)	19.5	Coleman Liau Index	16.6	Percentage of complex words	19.87
Flesch-Kincaid Reading Ease	31.4	Automated Readability Index	14.0	Average words per sentence	18.88
Flesch-Kincaid Grade Level	13.6	Sentences	8	Average syllables per word	1.85

Manuscript 5

Morote J, Celma A, Semidey ME, Antolín A, Miró B, Méndez O, et al. Analysis of Inflammatory Features in Suspicious Lesions for Significant Prostate Cancer on Magnetic Resonance Imaging—Are They Mimickers of Prostate Cancer? Cancers [Internet]. 2024 Dec 27;17(1):53. Available from: http://dx.doi.org/10.3390/cancers17010053

Abstract

Background: Inflammatory features can mimic PCa in suspicious MRI-lesions.

Objectives: To assess the incidence of inflammatory features in targeted biopsies to suspicious lesions.

Methods: A prospective analysis was conducted of 531 MRI-suspicious lesions with Prostate Imaging-Reporting and Data System (PI-RADS) scores of 3 to 5 in 364 men suspected of having PCa.

Results: The incidence of inflammatory features in the MRI-suspicious lesions without PCa was 69.6%, compared to 48.1% in those with PCa ($p < 0.001$). Among the suspicious lesions without PCa, the incidence of inflammatory features ranged from 68.6% to 71.2% across the PI-RADS categories ($p = 0.870$). Mild chronic prostatitis increased with higher PI-RADS scores, while acute prostatitis decreased, and granulomatous prostatitis was exclusively observed in patients with PI-RADS scores of 4 and 5. The incidence of inflammatory features in the lesions with insignificant PCa (grade group 1) was 66.7%, compared to 42.7% in those with significant PCa (grade group 2 to 5; $p = 0.027$). The detection of inflammatory features in MRI-suspicious lesions was identified as an independent predictor of a lower likelihood of significant PCa detection, with an odds ratio (OR) of 0.326 (95% CI 0.196-0.541). Mild chronic prostatitis was the only type of prostatitis which was an independent predictor of a lower likelihood of significant PCa, with an OR of 0.398

(95% CI 0.268-0.590).

Conclusions: These data suggest that inflammatory features may be considered mimickers of significant PCa on MRI.

Keywords:

inflammation, magnetic resonance imaging, prostate biopsy, prostate cancer, prostatitis

Original Lay Summary

The early detection of significant prostate cancer (PCa) has markedly improved with the widespread use of pre-biopsy magnetic resonance imaging (MRI) for men suspected of having PCa. However, challenges persist in scenarios involving the low detection rates of significant PCa and the overdetection of insignificant tumors. Inflammatory features are frequently observed in MRI-suspicious lesions that either lack significant PCa or harbor insignificant tumors. These findings suggest that such inflammatory features may mimic significant PCa. Comprehensive radiomic studies focusing on the types and extent of inflammatory features could help reduce the need for unnecessary prostate biopsies.

**Assessment Category**	**Score**	**Assessment Category**	**Score**	**Assessment Category**	**Score**
Comprehension Score Rater 1	4	Gunning Fog Score	19.3	Words	97
Comprehension Score Rater 2	4	SMOG Index	13.6	Complex words	28
Reading Age (years)	22.5	Coleman Liau Index	19.8	Percentage of complex words	28.87
Flesch-Kincaid Reading Ease	16.2	Automated Readability Index	16.8	Average words per sentence	19.4
Flesch-Kincaid Grade Level	15.8	Sentences	5	Average syllables per word	2.02

Lay Summary (Simple Prompt)

Many men undergo prostate biopsies after magnetic resonance imaging (MRI) shows areas that look suspicious for prostate cancer. However, not all of these suspicious areas actually contain cancer—sometimes, inflammation in the prostate can look very similar to cancer on MRI scans. In this study, we analyzed over 500 suspicious areas from prostate MRIs in more than 360 men to see how often inflammation was present. We found that inflammation was very common, especially in areas without cancer, and even in some cases where low-risk cancer was found. Interestingly, certain types of inflammation, like mild chronic prostatitis, were linked to a lower chance of having significant prostate cancer. This means that inflammation can "mimic" cancer on MRI, leading to unnecessary biopsies. Understanding these patterns can help doctors better interpret MRI results and possibly reduce the number of unnecessary prostate biopsies in the future.

**Assessment Category**	**Score**	**Assessment Category**	**Score**	**Assessment Category**	**Score**
Comprehension Score Rater 1	5	Gunning Fog Score	16.3	Words	143
Comprehension Score Rater 2	5	SMOG Index	11.8	Complex words	29
Reading Age (years)	19.5	Coleman Liau Index	16.0	Percentage of complex words	20.28
Flesch-Kincaid Reading Ease	34.1	Automated Readability Index	14.2	Average words per sentence	20.43
Flesch-Kincaid Grade Level	13.6	Sentences	7	Average syllables per word	1.8

Lay Summary (Extended Prompt)

Prostate cancer is often detected using magnetic resonance imaging (MRI), which helps doctors find suspicious areas that may need a biopsy. However, not all suspicious spots on an MRI are cancer. Inflammation, which is the body's response to irritation or infection, can sometimes look like cancer on these scans. In this study, we examined over 500 suspicious areas in the prostates of men who were being checked for cancer. We found that inflammation was very common in these areas, especially when there was no cancer or only a small, less dangerous type of cancer. This means inflammation can "mimic" or look like cancer on MRI, which might lead to unnecessary biopsies. Understanding this can help doctors make better decisions when interpreting MRI results, reducing unnecessary procedures and focusing more on cases that are likely to be significant prostate cancer.

**Assessment Category**	**Score**	**Assessment Category**	**Score**	**Assessment Category**	**Score**
Comprehension Score Rater 1	5	Gunning Fog Score	16.3	Words	139
Comprehension Score Rater 2	5	SMOG Index	11.8	Complex words	29
Reading Age (years)	18.5	Coleman Liau Index	14.3	Percentage of complex words	20.86
Flesch-Kincaid Reading Ease	44.3	Automated Readability Index	12.6	Average words per sentence	19.86
Flesch-Kincaid Grade Level	12.0	Sentences	7	Average syllables per word	1.68

Manuscript 6

Nturubika BDD, Logan J, Johnson IRD, Moore C, Li KL, Tang J, et al. Components of the Endosome-Lysosome Vesicular Machinery as Drivers of the Metastatic Cascade in Prostate Cancer. Cancers [Internet]. 2024 Dec 26;17(1):43. Available from: http://dx.doi.org/10.3390/cancers17010043

Abstract

Prostate cancer remains a significant global health concern, with over 1.4 million new cases diagnosed and more than 330,000 deaths each year. The primary clinical challenge that contributes to poor patient outcomes involves the failure to accurately predict and treat at the onset of metastasis, which remains an incurable stage of the disease. This review discusses the emerging paradigm that prostate cancer metastasis is driven by a dysregulation of critical molecular machinery that regulates endosome-lysosome homeostasis. Endosome and lysosome compartments have crucial roles in maintaining normal cellular function but are also involved in many hallmarks of cancer pathogenesis, including inflammation, immune response, nutrient sensing, metabolism, proliferation, signalling, and migration. Here we discuss new insight into how alterations in the complex network of trafficking machinery, responsible for the microtubule-based transport of endosomes and lysosomes, may be involved in prostate cancer progression. A better understanding of endosome-lysosome dynamics may facilitate the discovery of novel strategies to detect and manage prostate cancer metastasis and improve patient outcomes.

Keywords:

Rabs, biomarkers, endosome-lysosome dynamics, metastasis, molecular motors, prognosis, prostate cancer, vesicular trafficking machinery

<u>Original Lay Summary</u>

Prostate cancer is a major global health issue, with over 1.4 million new cases and more than 330,000 deaths annually. The main challenge in treating prostate cancer is predicting and managing metastasis, which is currently incurable. This review highlights that metastasis may be driven by disruptions in the vesicular trafficking machinery that controls endosome-lysosome biology. Understanding how changes in the transport of endosomes and lysosomes contribute to cancer progression could lead to new ways to detect and treat metastatic prostate cancer, improving patient outcomes.

**Assessment Category**	**Score**	**Assessment Category**	**Score**	**Assessment Category**	**Score**
Comprehension Score Rater 1	5	Gunning Fog Score	16.6	Words	87
Comprehension Score Rater 2	5	SMOG Index	11.9	Complex words	21
Reading Age (years)	19.5	Coleman Liau Index	16.9	Percentage of complex words	24.14
Flesch-Kincaid Reading Ease	33.6	Automated Readability Index	13.4	Average words per sentence	17.4
Flesch-Kincaid Grade Level	12.9	Sentences	5	Average syllables per word	1.84

Lay Summary (Simple Prompt)

Prostate cancer is a major health issue worldwide, and its most dangerous stage occurs when the cancer spreads to other parts of the body, known as metastasis. Unfortunately, predicting and treating prostate cancer at this stage is very difficult. This study explores how tiny structures inside cells, called endosomes and lysosomes, which normally help with essential cell functions, might play a key role in how prostate cancer spreads. These structures are part of a complex system that helps move materials within cells, and when this system doesn't work properly, it may contribute to cancer growth and progression. By understanding how changes in this cellular machinery influence cancer spread, researchers hope to find new ways to detect aggressive prostate cancer earlier and develop better treatments to improve patient outcomes.

**Assessment Category**	**Score**	**Assessment Category**	**Score**	**Assessment Category**	**Score**
Comprehension Score Rater 1	5	Gunning Fog Score	16.5	Words	128
Comprehension Score Rater 2	5	SMOG Index	11.6	Complex words	20
Reading Age (years)	20.5	Coleman Liau Index	16.3	Percentage of complex words	15.63
Flesch-Kincaid Reading Ease	41.4	Automated Readability Index	17.0	Average words per sentence	25.6
Flesch-Kincaid Grade Level	13.8	Sentences	5	Average syllables per word	1.65

Lay Summary (Extended Prompt)

Prostate cancer is one of the most common cancers in men and can become deadly when it spreads to other parts of the body, a process called metastasis. Scientists are still trying to understand why and how this spread happens, as it is hard to predict and treat. This study looks at tiny parts inside cells called endosomes and lysosomes, which help with important tasks like breaking down waste, moving nutrients, and sending signals. When these parts don't work properly, they may help cancer cells grow, move, and spread. By learning how changes in these cell parts affect prostate cancer, researchers hope to find new ways to detect the disease early and develop better treatments. This could lead to improved outcomes for patients with prostate cancer in the future.

**Assessment Category**	**Score**	**Assessment Category**	**Score**	**Assessment Category**	**Score**
Comprehension Score Rater 1	5	Gunning Fog Score	11.1	Words	129
Comprehension Score Rater 2	5	SMOG Index	6.8	Complex words	8
Reading Age (years)	15.5	Coleman Liau Index	12.8	Percentage of complex words	6.2
Flesch-Kincaid Reading Ease	65.0	Automated Readability Index	12.2	Average words per sentence	21.5
Flesch-Kincaid Grade Level	9.5	Sentences	6	Average syllables per word	1.42

Manuscript 7

Yanev I, Aprikian AG, Raizenne BL, Dragomir A. Cost-Effectiveness of PARP Inhibitors for Patients with BRCA1/2-Positive Metastatic Castration-Resistant Prostate Cancer—The Canadian Perspective. Cancers [Internet]. 2024 Dec 26;17(1):40. Available from: http://dx.doi.org/10.3390/cancers17010040

Abstract

Background/objectives: Through phase III clinical trials, PARP inhibitors have demonstrated outcome improvements in mCRPC patients with alterations in BRCA1/2 genes who have progressed on a second-generation androgen receptor pathway inhibitor (ARPI). While improving outcomes, PARP inhibitors contribute to the ever-growing economic burden of PCa. The objective of this project is to evaluate the cost-effectiveness of PARP inhibitors (olaparib, rucaparib, or talazoparib) versus the SOC (docetaxel or androgen receptor pathway inhibitors (ARPI)) for previously progressed mCRPC patients with BRCA1/2 mutations from the Canadian healthcare system perspective.

Methods: Partitioned survival models were created to represent mCRPC disease after progression until death. Survival inputs for BRCA1/2-mutated patients were extracted from the PROfound, TRITON3, and TALAPRO-1 clinical trials, while Canadian-specific costs are presented in 2023 dollars. Upon progression, patients were treated with chemotherapy. The considered time horizon was 5 years and outcomes were discounted at 1.5% per year.

Results: PARP inhibitors provide an additional survival of 0.19 quality-adjusted life years (QALY) when compared to the current standard of care, with additional costs of CAD 101,679 resulting in an incremental cost-utility ratio (ICUR) of CAD 565,383/QALY. The results were most sensitive to PARP inhibitors' acquisition costs and health-state utilities. PARP inhibitors required price reductions of up to 83% to meet the CAD 50,000/QALY willingness-to-pay threshold (WTP).

Conclusions: While providing survival benefits to previously progressed mCRPC patients presenting deleterious BRCA1/2 gene mutations, PARP inhibitors are not cost-effective and require major price reductions to reach local WTP thresholds.

Keywords:

PARP inhibitors, cost-effectiveness, healthcare system perspective, mCRPC, olaparib, partitioned survival model, prostate cancer, rucaparib, talazoparib

Original Lay Summary

While considering previously progressed metastatic castration-resistant prostate cancer (mCRPC) patients presenting deleterious BRCA1/2 mutations, poly(adenosine diphosphate–ribose) polymerase (PARP) inhibitors provide survival benefits over the current standard of care (SOC). PARP inhibitors are, however, associated with additional costs and their use is constrained by access to genetic testing, potentially placing an economic strain on the healthcare system. This paper aims to assess the cost-effectiveness of PARP inhibitors as a therapeutic class and not only as a single agent, considering approved and soon-to-be approved therapies. This flexible approach considers multiple treatment options comparing the SOC and PARP inhibitors to represent more accurately the clinical management of mCRPC and the potential impact of PARP inhibitors on the treatment landscape. Results from the Canadian healthcare system perspective suggest that PARP inhibitors are not cost-effective. This research impacts policymakers, guiding future reimbursement negotiations and future health economic research in the field.

**Assessment Category**	**Score**	**Assessment Category**	**Score**	**Assessment Category**	**Score**
Comprehension Score Rater 1	5	Gunning Fog Score	21.7	Words	152
Comprehension Score Rater 2	4	SMOG Index	15.8	Complex words	45
Reading Age (years)	24.0	Coleman Liau Index	20.5	Percentage of complex words	29.61
Flesch-Kincaid Reading Ease	6.9	Automated Readability Index	20.3	Average words per sentence	25.33
Flesch-Kincaid Grade Level	18.6	Sentences	6	Average syllables per word	2.06

Lay Summary (Simple Prompt)

Prostate cancer that has spread and no longer responds to hormone therapy can be challenging to treat, especially in patients with specific genetic changes in the BRCA1 or BRCA2 genes. Certain drugs called PARP inhibitors have shown they can help these patients live longer. However, these treatments are very expensive, and it is important to understand if their benefits are worth the high cost. This study looked at how cost-effective PARP inhibitors are compared to standard treatments like chemotherapy in Canada. The researchers used data from clinical trials and Canadian healthcare costs to evaluate the value of these drugs over five years. They found that while PARP inhibitors do help patients live longer with better quality of life, the cost is much higher than what is considered acceptable in the Canadian healthcare system. To be cost-effective, the prices of these drugs would need to be significantly reduced. This information can help guide healthcare decisions and drug pricing discussions.

**Assessment Category**	**Score**	**Assessment Category**	**Score**	**Assessment Category**	**Score**
Comprehension Score Rater 1	5	Gunning Fog Score	14.5	Words	160
Comprehension Score Rater 2	5	SMOG Index	11.0	Complex words	29
Reading Age (years)	18.5	Coleman Liau Index	15.0	Percentage of complex words	18.13
Flesch-Kincaid Reading Ease	50.1	Automated Readability Index	13.2	Average words per sentence	20.0
Flesch-Kincaid Grade Level	11.2	Sentences	8	Average syllables per word	1.61

Lay Summary (Extended Prompt)

Prostate cancer that spreads and no longer responds to hormone treatment is hard to manage, especially in patients with specific gene changes called BRCA1/2 mutations. New drugs called PARP inhibitors can help these patients live longer, but they are very expensive. This study looked at whether these drugs are worth the cost compared to standard treatments like chemotherapy in Canada. Using data from clinical trials and Canadian healthcare costs, we found that PARP inhibitors slightly improved survival but were much more expensive. To be considered cost-effective in Canada, their prices would need to drop by up to 83%. While PARP inhibitors can help patients with advanced prostate cancer, their current high cost makes them less affordable for the healthcare system. This information can help guide decisions about drug pricing and treatment choices in Canada.

**Assessment Category**	**Score**	**Assessment Category**	**Score**	**Assessment Category**	**Score**
Comprehension Score Rater 1	5	Gunning Fog Score	12.5	Words	135
Comprehension Score Rater 2	5	SMOG Index	9.8	Complex words	20
Reading Age (years)	17.5	Coleman Liau Index	15.5	Percentage of complex words	14.81
Flesch-Kincaid Reading Ease	53.2	Automated Readability Index	13.2	Average words per sentence	19.29
Flesch-Kincaid Grade Level	10.6	Sentences	7	Average syllables per word	1.59

Manuscript 8

Salgado-Hernández SV, Martínez-Retamoza L, Ocadiz-Delgado R, Pérez-Mora S, Cedeño-Arboleda GE, Gómez-García M del C, et al. miRNAs Dysregulated in Human Papillomavirus-Associated Benign Prostatic Lesions and Prostate Cancer. Cancers [Internet]. 2024 Dec 25;17(1):26. Available from: http://dx.doi.org/10.3390/cancers17010026

Abstract

Background/Objectives: Chronic inflammation is a critical factor in the development of prostatic diseases, including prostatitis, benign prostatic hyperplasia (BPH), and prostate cancer (PCa). Although this is recognized as a significant risk factor, the molecular mechanisms underlying these conditions remain unclear. Studies have shown that human papillomavirus (HPV) can infect the prostatic epithelium; however, its role in prostatic pathologies is not fully understood. This study aimed to evaluate the expression profiles of miRNAs involved in inflammation, proliferation, cell cycle regulation, apoptosis, angiogenesis, migration, invasion, and metastasis in HPV-positive and HPV-negative prostatic tissues. Methods: Using bioinformatics analyses, including interactomic and functional enrichment analyses, miRNAs associated with PCa and their potential roles in key cellular processes related to tumor progression were identified. Subsequently, some of these miRNAs were selected to evaluate their expression profiles in biopsies from patients with BPH/prostatitis and PCa, including HPV-positive and HPV-negative samples. Results: Our results revealed that 67.2% of benign lesions and 93.4% of cancer samples were HPV-positive. Interactomic analysis identified 284 miRNAs associated with PCa, with key miRNAs such as let-7c, miR-21, miR-34a, miR-126, miR-18a, miR-145, miR-221, miR-106a, miR-222, and miR-143 highlighted. Dysregulated expression of miRNAs was observed in HPV-positive tissues: miR-34a, miR-143, and miR-145 were downregulated in BPH/prostatitis, while miR-221 was upregulated. In HPV-positive PCa, additional miRNAs, including let-7c, miR-126, and miR-106a, showed altered expression. Complementarily, the functional enrichment analysis

revealed that these miRNAs are associated with key biological processes that are crucial to cancer, such as cell cycle regulation, apoptosis, immune response, angiogenesis, metastasis, and tumor suppressor pathways. This finding is consistent with the predicted target genes of the evaluated miRNAs and their potential roles in processes related to cancer development and progression. Conclusions: The differential expression of HPV-induced miRNAs suggests their potential as biomarkers for diagnosing, prognosing, and treating prostatic diseases. These findings offer new insights into the molecular mechanisms linking HPV to prostatic pathologies.

Keywords:

BPH, HPV, angiogenesis, apoptosis, functional enrichment analysis, inflammation, interactomic analysis, metastasis, miRNAs, proliferation, prostate cancer, prostatitis

<u>Original Lay Summary</u>

Chronic inflammation is a key factor in prostatic diseases, such as prostatitis, benign prostatic hyperplasia (BPH), and prostate cancer (PCa). This study explored the role of human papillomavirus (HPV) infection in these conditions by analyzing the expression of specific microRNAs (miRNAs) linked to various cellular processes involved in cancer. We identified 284 miRNAs associated with PCa by performing in silico analysis; these were found to be involved in cancer development and progression. Notably, a subset of these miRNAs exhibited dysregulated expression in HPV-positive tissues, suggesting their role in promoting a pro-oncogenic environment. These findings identify potential miRNA biomarkers for improving the diagnosis and treatment of prostatic diseases.

**Assessment Category**	**Score**	**Assessment Category**	**Score**	**Assessment Category**	**Score**
Comprehension Score Rater 1	5	Gunning Fog Score	22.3	Words	109
Comprehension Score Rater 2	5	SMOG Index	15.6	Complex words	37
Reading Age (years)	23.5	Coleman Liau Index	18.8	Percentage of complex words	33.94
Flesch-Kincaid Reading Ease	10.9	Automated Readability Index	17.2	Average words per sentence	21.8
Flesch-Kincaid Grade Level	17.2	Sentences	5	Average syllables per word	2.06

<u>Lay Summary (Simple Prompt)</u>

Prostate diseases like prostatitis, benign prostatic hyperplasia (BPH), and prostate cancer are common in men, but the exact causes are not fully understood. Chronic inflammation is a known risk factor, and certain viruses, such as human papillomavirus (HPV), may play a role. This study investigated how small molecules called microRNAs (miRNAs), which control important cell processes like growth, immune response, and cell death, behave in prostate tissues with and without HPV infection. Researchers analyzed tissue samples from patients with BPH, prostatitis, and prostate cancer to identify changes in miRNA activity. They found that HPV was present in many prostate tissue samples, especially in cancer cases, and that specific miRNAs showed abnormal activity when HPV was present. These miRNA changes could help explain how HPV contributes to prostate diseases and may serve as useful biomarkers to improve diagnosis, predict disease outcomes, and guide new treatment approaches.

**Assessment Category**	**Score**	**Assessment Category**	**Score**	**Assessment Category**	**Score**
Comprehension Score Rater 1	5	Gunning Fog Score	16.6	Words	145
Comprehension Score Rater 2	5	SMOG Index	11.8	Complex words	25
Reading Age (years)	21.5	Coleman Liau Index	17.3	Percentage of complex words	17.24
Flesch-Kincaid Reading Ease	32.9	Automated Readability Index	17.1	Average words per sentence	24.17
Flesch-Kincaid Grade Level	14.7	Sentences	6	Average syllables per word	1.77

Lay Summary (Extended Prompt)

Prostate diseases like prostatitis, benign prostatic hyperplasia (BPH), and prostate cancer are common, but we still don't fully understand what causes them. In this study, we looked at how certain small molecules in the body, called microRNAs (miRNAs), behave in prostate tissues. We focused on tissues that were either infected with human papillomavirus (HPV) or not, to see if HPV affects these molecules. By examining prostate samples from patients with both benign conditions and prostate cancer, we found that HPV was present in most cases, especially in cancer tissues. We also discovered that specific miRNAs had different activity levels in HPV-infected tissues, which might influence how prostate diseases develop and grow. These changes could help doctors find better ways to diagnose and treat prostate conditions. Our findings suggest that miRNAs affected by HPV could serve as useful markers for identifying and understanding prostate diseases more effectively.

**Assessment Category**	**Score**	**Assessment Category**	**Score**	**Assessment Category**	**Score**
Comprehension Score Rater 1	5	Gunning Fog Score	14.9	Words	147
Comprehension Score Rater 2	5	SMOG Index	10.7	Complex words	24
Reading Age (years)	19.5	Coleman Liau Index	16.4	Percentage of complex words	16.33
Flesch-Kincaid Reading Ease	40.5	Automated Readability Index	14.9	Average words per sentence	21.0
Flesch-Kincaid Grade Level	12.8	Sentences	7	Average syllables per word	1.71

Manuscript 9

Santarelli V, Carino D, Corvino R, Salciccia S, De Berardinis E, Krajewski W, et al. Surgical Technique and Perioperative Outcomes of the "Sapienza" Urology Residency Program's Trocar Placement Configuration During Robotic-Assisted Radical Prostatectomy (RARP): A Retrospective, Single-Centre Observational Study Comparing Experienced Attendings vs. Post-Graduate Year I–III Residents as Bedside Assistants. Cancers [Internet]. 2024 Dec 25;17(1):20. Available from: http://dx.doi.org/
10.3390/cancers17010020

Abstract

Background/Objectives: Robot-assisted radical prostatectomy (RARP) for the treatment of prostate cancer (PCa) has been standardized over the last 20 years. At our institution, only n = 3 rob arms are used for RARP. In addition, n = 2, 12 mm lap trocars are placed for the bedside assistant symmetrically at the midclavicular lines, which allows for direct pelvic triangulation and greater involvement of the assisting surgeon. The aim of our study was to compare surgical and perioperative outcomes of RARP performed using our alternative trocar placement with no fourth robotic arm in the subgroups of experienced attending surgeons and post-graduate residents as bedside assistants. Residents' satisfaction was also explored. Methods: RARPs performed within the urology residency program between 2019 and 2024 were retrospectively analyzed. Only rob procedures performed using our 3+2 trocars configuration were included. Intra- and postoperative outcomes, as well as long-term functional outcomes including continence recovery and potency, were assessed, stratified by the level of expertise of the bedside assistant, i.e., an experienced attending or post-graduate Year I-III resident. Satisfaction of residents assigned to the two groups during their robotic rotation was evaluated considering three domains with a score from 1 to 10: insight into surgical procedure, confidence level, and gratification level. Results: Out of n = 281 RARP procedures, the bedside assistant was an attending in 104 cases and a resident in 177. Operative time was

found to be slightly longer in cases where the second operator was a resident (attendings vs. residents: 134 ± 40 vs. 152 ± 24; p p = 0.025). However, cases where the second operator was a resident had a lower rate of positive surgical margins, with rates of 19.7% in the resident and 43.3% in the attending surgeon cohorts (OR = 0.32; 95% CI 0.18-0.55). This difference remained significant in multivariate analysis. There was no significant difference in postoperative blood transfusion rates (attendings vs. residents: 1.9% vs. 1.2%; p = 0.6). Similarly, long-term functional outcomes in terms of erectile dysfunction and urinary incontinence rates mostly overlapped between groups. The mean score in all three domains evaluating residents' satisfaction was significantly higher when residents actively participated in the surgical procedure as bedside assistants (p = 0.02, p = 0.004, and p Conclusions: These findings provide insight into how an alternative port positioning during RARP could improve the involvement of the bedside assistant, particularly residents, without compromising perioperative outcomes or surgical safety.

Keywords:

RARP, laparoscopy, residency, robotic-assisted, training, urology

<u>Original Lay Summary</u>

Robot-assisted radical prostatectomy (RARP) has been standardized for prostate cancer (PCa) over the last 20 years. Using the Sapienza residency program 3+2 trocar configuration with a second laparoscopic port replacing the fourth robotic arm, the bedside assistant is more involved during the surgical procedure and requires higher laparoscopic skills. In this study, we examine perioperative, functional, and oncological results of RARP using the Sapienza 3+2 trocar configuration in cases where the bedside assistant was either an attending surgeon or a urology resident. We identified that the level of expertise of the bedside assistant did not influence oncological and functional results. With this premise, the Sapienza 3+2 trocar configuration could be employed in residency programs to allow laparoscopic training of surgical residents without compromising the current request for robotic-assisted surgeries.

**Assessment Category**	**Score**	**Assessment Category**	**Score**	**Assessment Category**	**Score**
Comprehension Score Rater 1	5	Gunning Fog Score	21.5	Words	131
Comprehension Score Rater 2	5	SMOG Index	16.1	Complex words	39
Reading Age (years)	24.0	Coleman Liau Index	18.5	Percentage of complex words	29.77
Flesch-Kincaid Reading Ease	7.2	Automated Readability Index	19.1	Average words per sentence	26.2
Flesch-Kincaid Grade Level	18.8	Sentences	5	Average syllables per word	2.05

Lay Summary (Simple Prompt)

In prostate cancer surgery, robotic-assisted radical prostatectomy (RARP) has become a standard procedure. This study explored a unique surgical approach used at our institution, where only three robotic arms and two assistant ports are utilized, allowing residents in training to play a more active role during surgery. We compared the outcomes of surgeries assisted by experienced surgeons versus those assisted by residents in their early years of training. Our analysis of 281 cases showed that while operations with residents as assistants took slightly longer, these procedures had a lower rate of cancer-positive surgical margins, an important indicator of surgical success. There were no significant differences in complication rates or long-term recovery, including urinary control and sexual function. Residents also reported higher satisfaction when actively involved. These findings suggest that involving residents more directly in surgery can be both safe and beneficial for patient outcomes and surgical training.

**Assessment Category**	**Score**	**Assessment Category**	**Score**	**Assessment Category**	**Score**
Comprehension Score Rater 1	5	Gunning Fog Score	20.8	Words	150
Comprehension Score Rater 2	5	SMOG Index	14.8	Complex words	46
Reading Age (years)	22.5	Coleman Liau Index	18.0	Percentage of complex words	30.67
Flesch-Kincaid Reading Ease	22.7	Automated Readability Index	16.3	Average words per sentence	21.43
Flesch-Kincaid Grade Level	15.4	Sentences	7	Average syllables per word	1.92

Lay Summary (Extended Prompt)

This study looked at a new way to place surgical tools during robotic-assisted prostate cancer surgery. Normally, surgeons use four robotic arms, but at our hospital, we use only three robotic arms along with two special tools handled by an assistant. We wanted to see if having less experienced surgical residents assist, instead of senior surgeons, would affect the surgery's safety and success. We reviewed 281 surgeries to compare outcomes when residents helped versus when experienced surgeons did. We found that surgeries with residents took a little longer, but these cases had fewer signs that cancer was left behind after surgery. Blood loss and recovery of functions like bladder control and sexual health were similar in both groups. Residents also felt more confident and satisfied when they were actively involved. This suggests that our technique helps train new surgeons effectively without reducing patient safety or surgical success.

**Assessment Category**	**Score**	**Assessment Category**	**Score**	**Assessment Category**	**Score**
Comprehension Score Rater 1	5	Gunning Fog Score	15.8	Words	148
Comprehension Score Rater 2	5	SMOG Index	11.4	Complex words	31
Reading Age (years)	18.5	Coleman Liau Index	15.4	Percentage of complex words	20.95
Flesch-Kincaid Reading Ease	45.2	Automated Readability Index	12.8	Average words per sentence	18.5
Flesch-Kincaid Grade Level	11.6	Sentences	8	Average syllables per word	1.69

Manuscript 10

Cussenot O, Poupel L, Mousset C, Lavergne J, Bruyere F, Fontaine A, et al. Spatial Genomics Identifies Heat Shock Proteins as Key Molecular Changes Associated to Adipose Periprostatic Space Invasion in Prostate Cancer. Cancers [Internet]. 2024 Dec 24;17(1):2. Available from: http://dx.doi.org/10.3390/cancers17010002

Abstract

Purpose: To identify molecular changes during PCa invasion of adipose space using Spatial Transcriptomic Profiling of PCa cells.

Methods: This study was performed on paired intraprostatic and extraprostatic samples obtained from radical prostatectomy with pT3a pathological stages. Results: Differential gene expression revealed upregulation of heat shock protein genes: DNAJB1, HSPA8, HSP90AA1, HSPA1B, HSPA1A in PCa PanCK+ cells from the adipose periprostatic space. Extraprostatic extension was significantly associated with overexpression of genes involved in metastatic spread (EGR1, OR51E2, SPON2), of aggressiveness ERG negative signature of enhancers of androgen receptor (HOXB13, FOXA1), and of PSMA (FOLH1). They were associated with loss at 6q, 10q, 16q, and gain at 8q24 locus.

Conclusions: PCa invasion of adipose EPE induces adaptative process related to heat shock proteins; PCa cells in EPE also present transcriptomics signatures for ERG independent aggressiveness, androgen receptor co-activation, and specific CNV changes.

Keywords:

extra prostatic extension, heat shock proteins, prostate cancer, spatial transcriptomics

<u>Original Lay Summary</u>

Extraprostatic extension (EPE) of prostate cancer (PCa) cells in the adipose space (pT3a stage) is an unfavourable prognostic factor associated with recurrence and poor free metastatic disease-free survival outcomes of patients. However, molecular adaptation of PCa cells associated with EPE remains poorly understood. The aim of our research was to identify genomic determinants associated with EPE. We identified an overexpression of the heat shock protein genes: HSP90s, HSP70s, and HSP40s in PCa cells which infiltrate the adipose space. Moreover, we showed that EPE is also related to specific pathways associated with metastatic spread and enhancement of the androgen receptor pathway. These results provided valuable additional and new information to understand adaptative tumour changes during invasion of the adipose space This could be useful for the development of new markers or targeted therapies.

**Assessment Category**	**Score**	**Assessment Category**	**Score**	**Assessment Category**	**Score**
Comprehension Score Rater 1	5	Gunning Fog Score	20.9	Words	133
Comprehension Score Rater 2	5	SMOG Index	15.0	Complex words	41
Reading Age (years)	22.5	Coleman Liau Index	17.6	Percentage of complex words	30.83
Flesch-Kincaid Reading Ease	19.0	Automated Readability Index	16.4	Average words per sentence	22.17
Flesch-Kincaid Grade Level	16.1	Sentences	6	Average syllables per word	1.95

<u>Lay Summary (Simple Prompt)</u>

Prostate cancer can spread beyond the prostate into nearby fatty tissue, which often signals a more aggressive disease. This study aimed to understand the molecular changes that occur when prostate cancer invades this surrounding fat. By analyzing tissue samples from men who had surgery for prostate cancer, researchers identified key genetic changes in cancer cells that had spread outside the prostate. They discovered that certain heat shock proteins—molecules that help cells survive under stress—were significantly increased in these invasive cancer cells. Additionally, these cells showed genetic signs linked to faster cancer growth and spread. Understanding these changes can help scientists develop better ways to predict how aggressive a prostate cancer might become and could lead to new treatment strategies targeting these specific molecular pathways.

**Assessment Category**	**Score**	**Assessment Category**	**Score**	**Assessment Category**	**Score**
Comprehension Score Rater 1	5	Gunning Fog Score	15.4	Words	124
Comprehension Score Rater 2	5	SMOG Index	11.1	Complex words	22
Reading Age (years)	20.5	Coleman Liau Index	18.9	Percentage of complex words	17.74
Flesch-Kincaid Reading Ease	37.8	Automated Readability Index	16.7	Average words per sentence	20.67
Flesch-Kincaid Grade Level	13.1	Sentences	6	Average syllables per word	1.75

Lay Summary (Extended Prompt)

Prostate cancer can spread beyond the prostate into nearby fatty tissue, which often signals a more aggressive disease. This study aimed to understand the molecular changes that happen when prostate cancer invades this area. Researchers analyzed tissue samples from men who had surgery to remove their prostate due to advanced cancer. By using a special technique called spatial transcriptomics, they identified key changes in cancer cells that had spread into the fatty tissue. They found that certain proteins, called heat shock proteins, were more active in these cancer cells. These proteins help cells survive stress, which may help cancer spread. The study also discovered genetic changes linked to more aggressive cancer behavior. Understanding these changes can help researchers develop better ways to detect, predict, and potentially treat aggressive forms of prostate cancer in the future.

**Assessment Category**	**Score**	**Assessment Category**	**Score**	**Assessment Category**	**Score**
Comprehension Score Rater 1	5	Gunning Fog Score	12.1	Words	135
Comprehension Score Rater 2	5	SMOG Index	8.8	Complex words	18
Reading Age (years)	17.5	Coleman Liau Index	16.9	Percentage of complex words	13.33
Flesch-Kincaid Reading Ease	51.2	Automated Readability Index	13.2	Average words per sentence	16.88
Flesch-Kincaid Grade Level	10.3	Sentences	8	Average syllables per word	1.64

Manuscript 11

Kempin S, Buchner A, Brose SF, Schmidt-Hegemann N, May M, Wolff I, et al. The Effect of Resistance and/or Aerobic Training on Quality of Life, Fitness, and Body Composition in Prostate Cancer Patients—A Systematic Review and Meta-Analysis. Cancers [Internet]. 2024 Dec 23;16(24):4286. Available from: http://dx.doi.org/10.3390/cancers16244286

Abstract

Background: Prostate cancer (PC) and its treatment are often associated with side effects such as fatigue, muscle loss, and diminished quality of life (QoL). Physical exercise, particularly resistance training (RT) and aerobic training (AT), has been suggested as a strategy to mitigate these effects. However, the comparative efficacy of RT, AT, and combined RT/AT on QoL, body composition, physical fitness, and laboratory markers in PC patients is still insufficiently understood. Methods: Randomized controlled trials (RCTs) investigating structured RT, AT, or combined RT/AT programs in PC patients undergoing various treatments were included. The primary outcome was QoL, assessed using EORTC QLQ-C30 and EORTC QLQ-PR25 questionnaires. Secondary outcomes included body composition, fitness, and laboratory parameters. The studies were sourced from PubMed, Embase, and CENTRAL through May 2024. The effect sizes were pooled using random-effects models, and the risk of bias was systematically assessed following the GRADE approach. Results: A total of 30 RCTs, encompassing 2216 PC patients, were analyzed. Combined RT/AT significantly improved QoL subdomains, including global health, and cognitive and sexual function, while reducing fatigue and urinary symptoms. RT alone improved body composition by increasing lean body mass and reducing body fat percentage. Both RT and combined RT/AT enhanced strength (chest and leg press) and VO2peak. No significant changes were observed in laboratory markers, such as PSA or lipid levels. The effects of isolated AT remain unclear due to limited data. Conclusions: RT and combined RT/AT significantly improve QoL, fitness, and body composition in PC patients, with no detectable effect

on PSA or lipid levels. Further research is needed to elucidate the specific effects of AT and to investigate long-term outcomes.

Keywords:

biomarkers, cardiovascular health, depression reduction, endurance training, exercise intervention, fatigue management, fitness tests, functional capacity, mental health, muscle strength, physical activity, physical rehabilitation, prostate cancer, side effects of androgen deprivation therapy, strength training

<u>Original Lay Summary</u>

Worldwide, prostate cancer is the second most common cancer affecting men. Both the disease and treatments like surgery, radiotherapy, or drug therapy can cause unpleasant side effects, such as fatigue, muscle loss, and reduced quality of life. Exercise, particularly strength training and aerobic workouts, has been suggested to help reduce these effects. This study reviewed findings from controlled trials to evaluate how strength training, aerobic exercise, or a combination of both, affected men receiving prostate cancer treatment. The results show that combining strength and aerobic exercise significantly improves quality of life, especially in general health, mental clarity, and sexual wellbeing. Strength training alone helped by increasing muscle mass and lowering body fat. Both types of exercise improved physical strength and fitness. However, the training had no impact on laboratory results like PSA or cholesterol levels. More research is needed on long-term effects, particularly of aerobic exercise alone.

**Assessment Category**	**Score**	**Assessment Category**	**Score**	**Assessment Category**	**Score**
Comprehension Score Rater 1	5	Gunning Fog Score	16.0	Words	148
Comprehension Score Rater 2	5	SMOG Index	11.4	Complex words	35
Reading Age (years)	19.5	Coleman Liau Index	18.2	Percentage of complex words	23.65
Flesch-Kincaid Reading Ease	33.5	Automated Readability Index	14.0	Average words per sentence	16.44
Flesch-Kincaid Grade Level	12.7	Sentences	9	Average syllables per word	1.85

Lay Summary (Simple Prompt)

Prostate cancer and its treatments can lead to side effects like fatigue, muscle loss, and a lower quality of life. This study reviewed and analyzed data from 30 clinical trials involving over 2,200 prostate cancer patients to see how different types of exercise—resistance training (like weightlifting), aerobic training (like walking or cycling), and a combination of both—can help. The findings show that combining resistance and aerobic exercises significantly improves overall health, mental sharpness, and sexual well-being while reducing fatigue and urinary problems. Resistance training alone helps build muscle and reduce body fat, and both resistance and combined exercises boost physical strength and fitness. However, aerobic training on its own needs further research. These results suggest that regular, structured exercise can play an important role in improving the well-being and fitness of men with prostate cancer during and after treatment.

**Assessment Category**	**Score**	**Assessment Category**	**Score**	**Assessment Category**	**Score**
Comprehension Score Rater 1	4	Gunning Fog Score	17.9	Words	142
Comprehension Score Rater 2	4	SMOG Index	12.9	Complex words	30
Reading Age (years)	21.5	Coleman Liau Index	17.0	Percentage of complex words	21.13
Flesch-Kincaid Reading Ease	31.5	Automated Readability Index	16.7	Average words per sentence	23.67
Flesch-Kincaid Grade Level	14.7	Sentences	6	Average syllables per word	1.79

Lay Summary (Extended Prompt)

Prostate cancer and its treatments can cause problems like tiredness, muscle loss, and lower quality of life. This study looked at how different types of exercise—strength training, aerobic exercise, or a mix of both—can help men with prostate cancer feel better, get stronger, and improve their overall health. We reviewed 30 studies with over 2,200 men to see which exercises work best. We found that combining strength and aerobic exercises improved quality of life, mental sharpness, sexual health, and reduced tiredness and urinary problems. Strength training alone helped build muscle and reduce body fat. Both types of exercise made men stronger and improved their heart and lung fitness. However, exercise did not affect certain lab results, like prostate-specific markers. This shows that regular exercise can be a safe and effective way to help prostate cancer patients feel better and stay healthier during and after treatment.

**Assessment Category**	**Score**	**Assessment Category**	**Score**	**Assessment Category**	**Score**
Comprehension Score Rater 1	5	Gunning Fog Score	13.6	Words	147
Comprehension Score Rater 2	5	SMOG Index	9.9	Complex words	23
Reading Age (years)	17.5	Coleman Liau Index	14.8	Percentage of complex words	15.65
Flesch-Kincaid Reading Ease	52.4	Automated Readability Index	12.3	Average words per sentence	18.38
Flesch-Kincaid Grade Level	10.5	Sentences	8	Average syllables per word	1.61

Manuscript 12

von Stauffenberg F, Poyet C, Beintner-Skawran S, Maurer A, Schmid FA. Current Clinical Applications of PSMA-PET for Prostate Cancer Diagnosis, Staging, and Treatment. Cancers [Internet]. 2024 Dec 21;16(24):4263. Available from: http://dx.doi.org/10.3390/cancers16244263

Abstract

Over the past decade, prostate-specific membrane antigen positron emission tomography (PSMA-PET) has revolutionized prostate cancer (PCa) imaging, offering greater sensitivity and specificity compared to conventional imaging modalities such as CT, MRI, and bone scintigraphy. PSMA-PET is particularly valuable in staging newly diagnosed patients with intermediate- and high-risk disease, detecting biochemical recurrence, and evaluating metastatic cases. By utilizing radiotracers that accumulate specifically in PSMA-expressing cells, even small metastases can be detected, offering a detailed assessment of cancer extent and enabling more targeted diagnostic evaluations. Among the most utilized radiotracers, [68Ga]- and [18F]-labeled PSMA tracers enable precise imaging even with low disease burden. This diagnostic precision also supports advanced therapeutic approaches, including metastasis-directed therapy for oligometastatic cases and systemic treatment options, such as radioligand therapy, which presents new treatment perspectives for metastatic, castration-resistant PCa. This review examines the evolution of PSMA-PET in the diagnostics and therapy of PCa while comparing the current recommendations from leading clinical guidelines. The integration of PSMA-PET into clinical practice has redefined the management of PCa, improving diagnostic accuracy and enabling personalized treatment strategies, while lacking prospective long-term outcome data. As PSMA-PET continues to expand in clinical application, this review highlights its significant advancements while critically addressing limitations to ensure balanced and evidence-based implementation in prostate cancer care.

Keywords:

PSMA-PET, PSMA-RLT, [177Lu]Lu-PSMA, biochemical recurrence, diagnosis, mCRPC, prostate cancer, radical prostatectomy, radioligand therapy, staging

<u>Original Lay Summary</u>

Prostate-specific membrane antigen positron emission tomography (PSMA-PET) has emerged as a highly sensitive imaging modality for prostate cancer (PCa), outperforming conventional methods like CT, MRI, and bone scintigraphy. Over the past decade, PSMA-PET has gained widespread clinical adoption, particularly for staging intermediate- and high-risk PCa, detecting biochemical recurrence, and identifying metastatic disease. Its use of targeted radiotracers enables the detection of even small metastases, offering detailed tumor assessments and supporting advanced therapeutic strategies, especially in radioligand therapy for advanced cases. Integrated into leading clinical guidelines, PSMA-PET has significantly impacted diagnostic workflows and treatment planning. This review explores the evolution of PSMA-PET diagnostics and therapy, addressing its benefits, its integration into clinical practice, and its transformative potential. While limitations remain, PSMA-PET represents a critical step forward in the management of prostate cancer.

**Assessment Category**	**Score**	**Assessment Category**	**Score**	**Assessment Category**	**Score**
Comprehension Score Rater 1	5	Gunning Fog Score	24.6	Words	138
Comprehension Score Rater 2	4	SMOG Index	17.1	Complex words	53
Reading Age (years)	24.0	Coleman Liau Index	22.0	Percentage of complex words	38.41
Flesch-Kincaid Reading Ease	0.8	Automated Readability Index	20.3	Average words per sentence	23.0
Flesch-Kincaid Grade Level	18.9	Sentences	6	Average syllables per word	2.16

<u>Lay Summary (Simple Prompt)</u>

Prostate cancer is a common disease that can spread to other parts of the body if not detected early. A new imaging technique called PSMA-PET has transformed how doctors diagnose and treat prostate cancer. Unlike traditional scans, PSMA-PET can detect even very small cancer spread, helping doctors to see the full extent of the disease more accurately. This technology uses special tracers that highlight prostate cancer cells, making it easier to find cancer early, track its return after treatment, and guide therapy decisions. It also supports advanced treatments like targeted radiation for cancer that has spread to just a few areas and new therapies for advanced cases. This review explores how PSMA-PET is being used in clinical practice, compares current medical guidelines, and discusses both its benefits and limitations. Understanding PSMA-PET's role can help improve prostate cancer care through earlier detection and more personalized treatment strategies.

**Assessment Category**	**Score**	**Assessment Category**	**Score**	**Assessment Category**	**Score**
Comprehension Score Rater 1	5	Gunning Fog Score	15.0	Words	150
Comprehension Score Rater 2	5	SMOG Index	10.7	Complex words	24
Reading Age (years)	19.5	Coleman Liau Index	15.8	Percentage of complex words	16.0
Flesch-Kincaid Reading Ease	44.6	Automated Readability Index	14.6	Average words per sentence	21.43
Flesch-Kincaid Grade Level	12.4	Sentences	7	Average syllables per word	1.66

Lay Summary (Extended Prompt)

Prostate cancer is a common disease in men, and finding it early and understanding how far it has spread is key to effective treatment. A new type of imaging called PSMA-PET has greatly improved how doctors detect and monitor prostate cancer. Unlike older methods like CT scans or MRIs, PSMA-PET can spot even very small areas of cancer because it uses special markers that attach to cancer cells. This helps doctors see exactly where the cancer is, which makes it easier to plan the right treatment. PSMA-PET is useful for newly diagnosed patients, those whose cancer has come back, and men with cancer that has spread. It also helps guide advanced treatments like targeted radiation therapy. In this review, we explain how PSMA-PET is being used in prostate cancer care today, its benefits for patients, and what challenges remain to ensure it is used in the best possible way.

**Assessment Category**	**Score**	**Assessment Category**	**Score**	**Assessment Category**	**Score**
Comprehension Score Rater 1	5	Gunning Fog Score	11.9	Words	153
Comprehension Score Rater 2	5	SMOG Index	7.7	Complex words	12
Reading Age (years)	16.5	Coleman Liau Index	11.6	Percentage of complex words	7.84
Flesch-Kincaid Reading Ease	61.3	Automated Readability Index	11.4	Average words per sentence	21.86
Flesch-Kincaid Grade Level	10.1	Sentences	7	Average syllables per word	1.46

Manuscript 13

Arafa AT, Ludwig M, Tuncer O, Kollitz L, Gustafson A, Boytim E, et al. Isolation of Plasma Extracellular Vesicles for High-Depth Analysis of Proteomic Biomarkers in Metastatic Castration-Resistant Prostate Cancer Patients. Cancers [Internet]. 2024 Dec 21;16(24):4261. Available from: http://dx.doi.org/10.3390/cancers16244261

Abstract

Introduction: Prostate cancer treatment has been revolutionized by targeted therapies, including PARP inhibitors, checkpoint immunotherapies, and PSMA-targeted radiotherapies. Despite such advancements, accurate patient stratification remains a challenge, with current methods relying on genomic markers, tissue staining, and imaging. Extracellular vesicle (EV)-derived proteins offer a novel non-invasive alternative for biomarker discovery, holding promise for improving treatment precision. However, the characterization of plasma-derived EVs in prostate cancer patients remains largely unexplored. Methods: We conducted proteomic analyses on EVs isolated from plasma in 27 metastatic castration-resistant prostate cancer (mCRPC) patients. EVs were purified using ultracentrifugation and analyzed via mass spectrometry. Proteomic data were correlated with clinical markers such as serum prostate-specific antigen (PSA) and bone lesion counts. Statistical significance was assessed using Mann-Whitney t-tests and Spearman correlation. Results: The median age of patients was 74 (range: 44-94) years. At the time of blood collection, the median PSA level was 70 (range: 0.5-1000) ng/mL. All patients had bone metastasis. A total of 5213 proteins were detected, including EV-related proteins (CD9, CD81, CD63, FLOT1, TSG101) and cancer-related proteins (PSMA, B7-H3, PD-L1). Proteomic profiling of plasma EVs revealed a significant correlation between specific EV-derived proteins and clinical prognostic markers. B7-H3, LAT1, and SLC29A1 showed a strong association with serum PSA levels and number of bone lesions, indicating potential for these proteins to serve as biomarkers of disease burden and therapy response. Conclusions: Our findings demonstrate the potential of EV-based proteomics for identifying biomarkers in mCRPC patients. Proteins such

as B7-H3 and LAT1 could guide precision oncology approaches, improving patient stratification. Future research incorporating outcomes data and EV subpopulation analysis is needed to establish clinical relevance.

Keywords:

B7-H3, PSMA, extracellular vesicles, prostate cancer, proteomics

<u>Original Lay Summary</u>

We explored the potential of using proteins found in extracellular vesicles (EVs), which are tiny particles released by cancer cells, to predict treatment response and identify new drug targets. Unlike traditional tissue biopsies, analyzing EVs from blood samples offers a non-invasive way to obtain valuable information about the cancer. Our findings showed that certain proteins in EVs, such as B7-H3 and LAT1, are correlated with clinical markers such as PSA levels, PET scans, and serum alkaline phosphatase levels that are used to monitor disease burden and cancer progression. This approach could lead to more precise and personalized treatment options for patients with advanced prostate cancer, helping doctors choose the most effective therapies and improve outcomes.

**Assessment Category**	**Score**	**Assessment Category**	**Score**	**Assessment Category**	**Score**
Comprehension Score Rater 1	5	Gunning Fog Score	18.9	Words	117
Comprehension Score Rater 2	4	SMOG Index	13.2	Complex words	21
Reading Age (years)	22.5	Coleman Liau Index	16.6	Percentage of complex words	17.95
Flesch-Kincaid Reading Ease	29.6	Automated Readability Index	19.1	Average words per sentence	29.25
Flesch-Kincaid Grade Level	16.4	Sentences	4	Average syllables per word	1.74

Lay Summary (Simple Prompt)

Prostate cancer can become very difficult to treat when it spreads and stops responding to standard hormone therapies. To improve treatment, doctors need better ways to identify which therapies will work best for each patient. In this study, we explored tiny particles in the blood called extracellular vesicles, which carry proteins that may provide important clues about the cancer's behavior. By analyzing blood samples from patients with advanced prostate cancer, we discovered specific proteins linked to how severe the disease is, such as the level of prostate-specific antigen (PSA) in the blood and the number of cancerous bone lesions. These proteins, like B7-H3 and LAT1, could help doctors predict how a patient's cancer will progress and how they might respond to different treatments. This approach could lead to more personalized, effective therapies without the need for invasive procedures, offering new hope for patients with advanced prostate cancer.

**Assessment Category**	**Score**	**Assessment Category**	**Score**	**Assessment Category**	**Score**
Comprehension Score Rater 1	5	Gunning Fog Score	16.1	Words	149
Comprehension Score Rater 2	4	SMOG Index	11.3	Complex words	23
Reading Age (years)	20.5	Coleman Liau Index	15.7	Percentage of complex words	15.44
Flesch-Kincaid Reading Ease	43.7	Automated Readability Index	16.2	Average words per sentence	24.83
Flesch-Kincaid Grade Level	13.3	Sentences	6	Average syllables per word	1.63

<u>Lay Summary (Extended Prompt)</u>

Prostate cancer can be difficult to treat because it spreads differently in each patient. Doctors need better ways to understand how the disease is progressing and which treatments will work best. In this study, we looked at tiny particles in the blood called extracellular vesicles. These particles carry proteins that can give important clues about a person's cancer. We collected blood samples from 27 men with advanced prostate cancer and used special techniques to find and study over 5,000 different proteins in these particles. We discovered that some proteins, like B7-H3 and LAT1, are linked to how much cancer is in the body and how severe it is. These proteins could help doctors predict how the disease will behave and choose the right treatment for each patient. This research could lead to simple blood tests that make cancer care more precise and personalized.

**Assessment Category**	**Score**	**Assessment Category**	**Score**	**Assessment Category**	**Score**
Comprehension Score Rater 1	5	Gunning Fog Score	11.4	Words	145
Comprehension Score Rater 2	5	SMOG Index	8.0	Complex words	15
Reading Age (years)	15.5	Coleman Liau Index	13.0	Percentage of complex words	10.34
Flesch-Kincaid Reading Ease	63.0	Automated Readability Index	10.7	Average words per sentence	18.13
Flesch-Kincaid Grade Level	9.0	Sentences	8	Average syllables per word	1.48

Manuscript 14

Roukoz C, Lazrek A, Bardoscia L, Rubini G, Liu CM, Serre AA, et al. Evidences on the Use of Hypofractionation in Postoperative/Salvage Radiotherapy for Prostate Cancer: Systematic Review of the Literature and Recent Developments. Cancers [Internet]. 2024 Dec 18;16(24):4227. Available from: http://dx.doi.org/10.3390/cancers16244227

Abstract

Background: Radical prostatectomy (RP) is one possible curative treatment for localized prostate cancer. Despite that, up to 40% of patients will later relapse. Currently, post-operative radiotherapy (PORT) courses deliver 1.8-2 Gy daily to reach a total dose ranging between 64 and 74 Gy, completed in 7-8 weeks. Several articles reported encouraging data in terms of the effectiveness and the related toxicities using hypofractionation schedules. The objective of the present systematic review was to evaluate the clinical outcomes and toxicity of the use of hypofractionation in adjuvant/salvage prostate cancer treatments.

Methods: Medline was searched via PubMed and Scopus from inception to July 2024 to retrieve studies on hypofractionation in adjuvant/salvage prostate cancer treatments. This study was conducted under PRISMA guidelines.

Results: A total of 139 articles were identified from the initial search. Subsequently, the 139 studies were reviewed by title and abstract. Ninety-five studies were excluded due to being either abstracts or articles not available in English. In the second step, the full texts of 44 studies were reviewed. Eleven studies were excluded for being reviews, study protocols, or focused on SBRT treatments. Finally, 33 studies were included in our analysis, with a total number of 4269 patients. Of the 33 selected studies, 20 were retrospective trials and 11 were phase I/II prospective trials, while 2 studies were prospective phase III trials. The follow-up ranged from 18 to 217 months. Failure-free survival, for those with the longer follow-up, ranged

between 85% and 91% at 3 years, 47 and 78.6% at 5 years and 51.5% at 10 years. Genitourinary (GU) and gastrointestinal acute toxicity was mild to moderate with similar rates across the normofractionated and hypofractionated groups. Acute grade-3 GU toxicity events were unusual, occurring in less than 4% of the cases overall.

Conclusion: The present study is the first systematic review of the literature that includes the first two randomized phase III studies published in the literature. Hypofractionated treatment has been shown to be safe, effective, with moderate toxicity and not inferior to conventional RT, with good biochemical control rates.
Keywords:

hypofractionated post-operative radiotherapy, hypofractionated radiotherapy, prostatic bed radiotherapy, salvage radiotherapy

<u>Original Lay Summary</u>

The objective of the present systematic review was to evaluate the clinical outcomes and toxicity of the use of hypofractionation in adjuvant/salvage prostate cancer treatments. A total of 139 studies were identified and, finally, 33 studies were included in our analysis, with a total number of 4269 patients. Biochemical relapse-free survival (bRFS) ranged between 85% and 91% at 3 years, and between 47% and 78.6% at 5 years. Genitourinary (GU) and gastrointestinal (GI) acute toxicity was mild to moderate with comparable rates across the studies. Although some doubts remain on late toxic effects, hypofractionated treatment has been shown to be safe, effective, with moderate toxicity and not inferior to conventional RT, with good biochemical control rates.

**Assessment Category**	**Score**	**Assessment Category**	**Score**	**Assessment Category**	**Score**
Comprehension Score Rater 1	5	Gunning Fog Score	16.7	Words	118
Comprehension Score Rater 2	5	SMOG Index	12.0	Complex words	26
Reading Age (years)	19.5	Coleman Liau Index	14.3	Percentage of complex words	22.03
Flesch-Kincaid Reading Ease	29.9	Automated Readability Index	12.5	Average words per sentence	19.67
Flesch-Kincaid Grade Level	14.0	Sentences	6	Average syllables per word	1.86

Lay Summary (Simple Prompt)

After surgery for prostate cancer, many men may still experience a return of the disease, requiring additional treatment with radiotherapy. Traditionally, this involves small daily doses over 7 to 8 weeks. However, a newer approach called hypofractionation delivers higher doses in fewer sessions, potentially saving time and offering similar results. This study systematically reviewed 33 clinical studies involving 4,269 patients to understand how effective and safe hypofractionated radiotherapy is after prostate cancer surgery. The results showed that this shorter treatment was just as effective as the traditional approach in controlling cancer, with similar rates of mild to moderate side effects affecting the urinary and digestive systems. Serious side effects were rare. These findings suggest that hypofractionated radiotherapy is a safe, effective, and more convenient option for men needing radiotherapy after prostate cancer surgery, which could improve both treatment experiences and healthcare efficiency.

**Assessment Category**	**Score**	**Assessment Category**	**Score**	**Assessment Category**	**Score**
Comprehension Score Rater 1	4	Gunning Fog Score	18.8	Words	143
Comprehension Score Rater 2	3	SMOG Index	13.4	Complex words	38
Reading Age (years)	22.5	Coleman Liau Index	19.4	Percentage of complex words	26.57
Flesch-Kincaid Reading Ease	13.9	Automated Readability Index	17.0	Average words per sentence	20.43
Flesch-Kincaid Grade Level	16.4	Sentences	7	Average syllables per word	2.03

<u>Lay Summary (Extended Prompt)</u>

After surgery to treat prostate cancer, many men face the risk of the cancer returning. To reduce this risk, doctors often use radiation therapy, which usually takes 7–8 weeks to complete. This study looked at a different approach called hypofractionation, where higher doses of radiation are given in fewer sessions, making the treatment shorter. We reviewed 33 studies involving 4,269 patients to see if this shorter treatment is as safe and effective as the traditional method. The results showed that hypofractionated radiation works just as well in controlling cancer, with similar side effects that were mostly mild to moderate. Serious side effects were rare. These findings suggest that hypofractionation is a safe, effective option that can shorten treatment time without increasing risks, making it more convenient for patients.

**Assessment Category**	**Score**	**Assessment Category**	**Score**	**Assessment Category**	**Score**
Comprehension Score Rater 1	4	Gunning Fog Score	13.9	Words	129
Comprehension Score Rater 2	4	SMOG Index	10.1	Complex words	21
Reading Age (years)	18.5	Coleman Liau Index	15.5	Percentage of complex words	16.28
Flesch-Kincaid Reading Ease	43.2	Automated Readability Index	12.8	Average words per sentence	18.43
Flesch-Kincaid Grade Level	11.8	Sentences	7	Average syllables per word	1.71

Manuscript 15

Jongen CAM, Heemsbergen WD, Incrocci L, Heijmen BJM, Rossi L. Added Value of Biological Effective Dose in Dosiomics-Based Modelling of Late Rectal Bleeding in Prostate Cancer. Cancers [Internet]. 2024 Dec 17;16(24):4208. Available from: http://dx.doi.org/10.3390/cancers16244208

Abstract

Background/objectives: Extracting spatial features (texture analysis) from dose distributions (dosiomics) for outcome prediction is a rapidly evolving field in radiotherapy. To account for fraction size differences, the biological effective dose (BED) is often calculated. We evaluated the impact and added value of the BED in the dosiomics prediction modelling of grade ≥ 2 late rectal bleeding (LRB) probability within 5 years after treatment in three parts.

Methods: For N = 656 prostate cancer patients previously treated in a randomized trial with conventional (CF) or hypofractionated (HF) radiotherapy, 42 dosiomic features were extracted from the dose distributions of the delineated rectum in physical doses and from dose distributions converted to the BED. Part 1: To assess whether an HF BED dosiomics model is generalizable to CF and vice versa, multivariate logistic regression BED models were constructed for HF and CF separately and tested on the other fractionation scheme. Part 2: The BED models were fitted to combined HF and CF data together to test whether this resulted in better models. Part 3: Separate physical HF and CF models were constructed and compared to the BED models.

Results: Part 1: Dosiomics related to large-zone and long-run high-dose levels were predictive for both HF and CF. Deviation from the mean gray level was only predictive for HF. The BED HF model calibrations with CF data and vice versa were generally poor. AUCs ranged from 0.55 to 0.65. Part 2: Compared to the separate models, the models fitted to the combined HF and CF data showed better discriminative ability in

CF but not in HF. Part 3: The apparent performances of models for the BED and physical dose were similar.

Conclusions: Using the BED in the predictive dosiomic modelling of late rectal bleeding after prostate cancer radiotherapy to account for differences in fraction doses was of limited value.

Keywords:

NTCP, dosiomics, late rectal bleeding, radiobiological modelling, texture analysis

<u>Original Lay Summary</u>

By identifying patterns and voxel correlations (texture analysis), it is possible to characterize dose distributions. These dose parameters are called dosiomic features. The linear-quadratic model is a widely applied model in radiotherapy to calculate the biological effective dose for a tissue of interest based on the fraction size sensitivity, fraction size and total dose. In this study, we investigated the added value and impact of applying biological effective dose to dosiomic features in prediction models.

**Assessment Category**	**Score**	**Assessment Category**	**Score**	**Assessment Category**	**Score**
Comprehension Score Rater 1	3	Gunning Fog Score	18.7	Words	76
Comprehension Score Rater 2	3	SMOG Index	13.2	Complex words	21
Reading Age (years)	21.5	Coleman Liau Index	17.6	Percentage of complex words	27.63
Flesch-Kincaid Reading Ease	13.9	Automated Readability Index	14.8	Average words per sentence	19.0
Flesch-Kincaid Grade Level	16.0	Sentences	4	Average syllables per word	2.05

Lay Summary (Simple Prompt)

Radiotherapy is a common treatment for prostate cancer, but it can cause side effects like late rectal bleeding, which impacts quality of life. Scientists are exploring new ways to predict who might develop these side effects by analyzing patterns in how radiation doses are distributed—a technique called dosiomics. This study investigated whether adjusting these radiation dose patterns based on biological effects, known as the biological effective dose (BED), could improve the accuracy of predicting late rectal bleeding. Data from 656 prostate cancer patients treated with different radiotherapy methods were analyzed. The results showed that using BED did not significantly improve the ability to predict rectal bleeding compared to traditional dose measurements. This finding suggests that while dosiomics is a promising tool, adding BED may not provide extra benefits for predicting this specific side effect, helping researchers focus on more effective prediction methods in the future.

**Assessment Category**	**Score**	**Assessment Category**	**Score**	**Assessment Category**	**Score**
Comprehension Score Rater 1	5	Gunning Fog Score	18.2	Words	145
Comprehension Score Rater 2	5	SMOG Index	13.3	Complex words	32
Reading Age (years)	22.5	Coleman Liau Index	18.1	Percentage of complex words	22.07
Flesch-Kincaid Reading Ease	21.3	Automated Readability Index	17.7	Average words per sentence	24.17
Flesch-Kincaid Grade Level	16.3	Sentences	6	Average syllables per word	1.9

<u>Lay Summary (Extended Prompt)</u>

Radiotherapy is a common treatment for prostate cancer, but it can sometimes cause long-term side effects like rectal bleeding. To better predict who might experience this side effect, researchers use advanced computer models that analyze how radiation is distributed in the body—a method called dosiomics. This study explored whether including a special calculation called the biological effective dose (BED), which adjusts for differences in treatment schedules, could improve these predictions. We analyzed data from 656 patients treated with different radiation approaches to see if BED-based models worked better than models using standard radiation dose data. Our results showed that adding BED did not significantly improve the accuracy of predicting rectal bleeding. This finding suggests that simpler models without BED may be just as effective, helping doctors focus on more straightforward ways to predict and manage side effects in prostate cancer patients.

**Assessment Category**	**Score**	**Assessment Category**	**Score**	**Assessment Category**	**Score**
Comprehension Score Rater 1	5	Gunning Fog Score	16.0	Words	143
Comprehension Score Rater 2	5	SMOG Index	11.6	Complex words	24
Reading Age (years)	20.5	Coleman Liau Index	17.6	Percentage of complex words	16.78
Flesch-Kincaid Reading Ease	32.4	Automated Readability Index	17.2	Average words per sentence	23.83
Flesch-Kincaid Grade Level	14.7	Sentences	6	Average syllables per word	1.78

Manuscript 16

Chhatre S, Malkowicz SB, Vapiwala N, Guzzo TJ, Jayadevappa R. Association Between Hospital Efficiency and Quality of Care Among Fee-for-Service Medicare Beneficiaries with Prostate Cancer: A Retrospective Cohort Study. Cancers [Internet]. 2024 Dec 13;16(24):4154. Available from: http://dx.doi.org/10.3390/cancers16244154

Abstract

Background: Technical efficiency evaluates a hospital's economic performance and plays an important role in variations in quality of care and outcomes. The study objective was to examine the association between hospital efficiency and quality of care among fee-for-service Medicare beneficiaries with prostate cancer and to assess if race moderates this association.

Design: Retrospective study using Surveillance, Epidemiological, and End Results-Medicare (SEER-Medicare) data from 1998 to 2016 for prostate cancer patients aged ≥ 66. We computed hospital technical efficiency using a data envelopment analysis. Outcomes were emergency room visits, hospitalizations, cost, and mortality (all-cause and prostate cancer-specific). We used competing risk regression for survival, log-link GLM models for cost, and Poisson models for count data. The propensity score approach was used to minimize selection bias.

Results: The cohort consisted of 323,325 patients. Compared to higher efficiency hospitals (upper quartile), low hospital efficiency (i.e., lower quartile) was associated with a higher hazard of long-term mortality (Hazard ratio (HR) = 1.06, 95% CI = 1.05, 1.08) and long-term prostate cancer-specific mortality (HR = 1.14, 95% CI = 1.11, 1.17). Compared to higher efficiency levels, lower levels were associated with impaired emergency room visits, hospitalizations, and costs. A one unit increase in the efficiency score was associated with greater benefits for cost and mortality for

African American and white patients. The benefit was larger for African American patients compared to white patients.

Conclusions: Increasing hospital efficiency may help improve outcomes among Medicare prostate cancer patients. Policies to redirect patients to hospitals with higher efficiency can enhance the quality of care and outcomes.

<u>Keywords:</u>

health service use, hospital technical efficiency, mortality, prostate cancer, racial disparity

Original Lay Summary

Hospitals are an important sector of the U.S. economy. Hospital characteristics such as efficiency play an important role in the quality and cost of care. The impact of differences in hospital efficiency on quality can go either way: efficiency forces hospitals to cut prices and quality or to improve quality. Race and ethnicity may affect the association between hospital efficiency and outcomes. In this study, we examined the association between hospital efficiency and quality of care in fee-for-service Medicare beneficiaries with prostate cancer. We also assessed if race moderates the observed association. We used Surveillance, Epidemiological, and End Results—Medicare (SEER-Medicare) data from 1998 to 2016 for prostate cancer patients aged 66 or older. SEER-Medicare data were linked to American Hospital Association data. We concluded that increasing hospital efficiency may help improve outcomes of prostate cancer care in Medicare fee-for-service patients and provide larger benefits to African American patients compared to white patients. Policies to redirect patients to hospitals with higher efficiency can thus improve quality of care and outcomes.

**Assessment Category**	**Score**	**Assessment Category**	**Score**	**Assessment Category**	**Score**
Comprehension Score Rater 1	5	Gunning Fog Score	14.3	Words	176
Comprehension Score Rater 2	5	SMOG Index	11.8	Complex words	50
Reading Age (years)	18.5	Coleman Liau Index	16.5	Percentage of complex words	28.41
Flesch-Kincaid Reading Ease	27.6	Automated Readability Index	11.7	Average words per sentence	14.67

Flesch-Kincaid Grade Level	13.1	Sentences	12	Average syllables per word	1.94

<u>Lay Summary (Simple Prompt)</u>

This study looked at how efficiently hospitals operate and whether that affects the quality of care for older men with prostate cancer who are covered by Medicare. The researchers analyzed data from over 323,000 patients treated between 1998 and 2016 to see if hospital efficiency influenced important outcomes like emergency room visits, hospital stays, healthcare costs, and survival rates. They found that patients treated in less efficient hospitals had higher risks of dying from prostate cancer and other causes, more hospital visits, and higher medical costs. Interestingly, improving hospital efficiency seemed to benefit African American patients even more than white patients. These results suggest that sending patients to more efficient hospitals could lead to better care, lower costs, and improved survival, especially for groups that often face health disparities. This information could help guide healthcare policies to improve outcomes for prostate cancer patients.

**Assessment Category**	**Score**	**Assessment Category**	**Score**	**Assessment Category**	**Score**
Comprehension Score Rater 1	5	Gunning Fog Score	17.4	Words	144
Comprehension Score Rater 2	5	SMOG Index	13.1	Complex words	31
Reading Age (years)	21.5	Coleman Liau Index	17.4	Percentage of complex words	21.53
Flesch-Kincaid Reading Ease	31.5	Automated Readability Index	17.1	Average words per sentence	24.0
Flesch-Kincaid Grade Level	14.8	Sentences	6	Average syllables per word	1.78

Lay Summary (Extended Prompt)

This study looked at how well hospitals use their resources (called hospital efficiency) and how this affects the quality of care for older men with prostate cancer who have Medicare. The researchers wanted to find out if patients treated at more efficient hospitals had better health outcomes, like lower chances of dying from prostate cancer, fewer hospital visits, and lower medical costs. They studied data from over 323,000 patients treated between 1998 and 2016. The results showed that patients in less efficient hospitals were more likely to die from prostate cancer and had higher medical costs and more hospital visits. Interestingly, the benefits of being treated at more efficient hospitals were even greater for African American patients compared to white patients. This study suggests that improving hospital efficiency can lead to better care and outcomes for prostate cancer patients and may help reduce racial differences in health outcomes.

**Assessment Category**	**Score**	**Assessment Category**	**Score**	**Assessment Category**	**Score**
Comprehension Score Rater 1	5	Gunning Fog Score	15.6	Words	149
Comprehension Score Rater 2	5	SMOG Index	11.6	Complex words	24
Reading Age (years)	19.5	Coleman Liau Index	15.2	Percentage of complex words	16.11
Flesch-Kincaid Reading Ease	40.3	Automated Readability Index	15.8	Average words per sentence	24.83
Flesch-Kincaid Grade Level	13.8	Sentences	6	Average syllables per word	1.67

Manuscript 17

Adekoya TO, Smith N, Kothari P, Dacanay MA, Li Y, Richardson RM. CXCR1 Expression in MDA-PCa-2b Cell Upregulates ITM2A to Inhibit Tumor Growth. Cancers [Internet]. 2024 Dec 11;16(24):4138. Available from: http://dx.doi.org/10.3390/cancers16244138

Abstract

Background: Chemokines, along with their receptors, exert critical roles in tumor development and progression. In prostate cancer (PCa), interleukin-8 (IL-8/CXCL8) was shown to enhance angiogenesis, proliferation, and metastasis. CXCL8 activates two receptors, CXCR1 and CXCR2. While CXCR2 expression was shown to promote PCa growth and metastasis, the role of CXCR1 remains unclear.

Methods: In this study, we stably expressed CXCR1 and, as control, CXCR2 in the androgen-dependent PCa cell line MDA-PCa-2b to evaluate the effect of CXCR1 in tumor development.

Results: MDA-PCa-2b-CXCR1 cells showed decreased cell migration, protein kinase-B (AKT) activation, prostate-specific antigen (PSA) expression, cell proliferation, and tumor development in nude mice, relative to MDA-PCa-2b-Vec and MDA-PCa-2b-CXCR2 cells. MDA-PCa-2b-CXCR1 cells also displayed a significant transition to mesenchymal phenotypes as characterized by decreased E-cadherin expression and a corresponding increased level of N-cadherin and vimentin expression. RNA-seq and Western blot analysis revealed a significant increase in the tumor suppressor integral membrane protein 2A (ITM2A) expression in MDA-PCa-2b-CXCR1 compared to control cells. In prostate adenocarcinoma tissue, ITM2A expression was also shown to be downregulated relative to a normal prostate. Interestingly, the overexpression of ITM2A in MDA-PCa-2b cells (MDA-PCa-2b-ITM2A-GFP) inhibited tumor growth similar to that of MDA-PCa-2b-CXCR1.

Conclusions: Taken together, the data suggest that CXCR1 expression in MDA-PCa-2b cells may upregulate ITM2A to abrogate tumor development.

Keywords:

CXCR1, CXCR2, ITM2A, androgen receptor, interleukin-8 receptors, prostate cancer, prostate-specific antigen

Original Lay Summary

Prostate cancer constitutes a large percentage of cancer-related deaths among men. Due to the complexity of the disease process and resistance to androgen deprivation therapy, a lot remains unknown about prostate tumorigenesis. Recently, several chemokines, including CXCL8 and its receptors (CXCR1 and CXCR2), have been shown to impact the development and progression of prostate cancer. In this study, we used various assays and animal models to better understand the role of CXCL8 receptors in prostate cancer incidence. The results herein indicate that the receptors may have opposing effects in the onset of prostate cancer. CXCR1 expression, not CXCR2, upregulates the tumor suppressor ITM2A to inhibit prostate tumor growth.

**Assessment Category**	**Score**	**Assessment Category**	**Score**	**Assessment Category**	**Score**
Comprehension Score Rater 1	5	Gunning Fog Score	17.2	Words	109
Comprehension Score Rater 2	5	SMOG Index	12.3	Complex words	27
Reading Age (years)	20.5	Coleman Liau Index	16.7	Percentage of complex words	24.77
Flesch-Kincaid Reading Ease	34.7	Automated Readability Index	13.6	Average words per sentence	18.17
Flesch-Kincaid Grade Level	12.9	Sentences	6	Average syllables per word	1.82

Lay Summary (Simple Prompt)

Prostate cancer is one of the most common cancers in men, and understanding how it grows and spreads is crucial for finding better treatments. In this study, researchers explored the role of a protein called CXCR1, which interacts with chemical signals in the body. They introduced CXCR1 into prostate cancer cells to see how it affects tumor growth. Surprisingly, they found that CXCR1 reduced the ability of these cancer cells to grow, spread, and form tumors. This effect was linked to an increase in another protein called ITM2A, known for its tumor-suppressing properties. When ITM2A was increased in cancer cells, it also helped stop tumor growth. These findings suggest that CXCR1 may play a protective role against prostate cancer by boosting ITM2A, offering new insights that could lead to improved therapies in the future.

**Assessment Category**	**Score**	**Assessment Category**	**Score**	**Assessment Category**	**Score**
Comprehension Score Rater 1	5	Gunning Fog Score	11.3	Words	135
Comprehension Score Rater 2	5	SMOG Index	7.7	Complex words	12
Reading Age (years)	16.5	Coleman Liau Index	13.6	Percentage of complex words	8.89
Flesch-Kincaid Reading Ease	60.0	Automated Readability Index	11.8	Average words per sentence	19.29
Flesch-Kincaid Grade Level	9.7	Sentences	7	Average syllables per word	1.5

Lay Summary (Extended Prompt)

Prostate cancer is a common type of cancer in men, and understanding how it grows can help find better ways to treat it. This study looked at a protein called CXCR1, which is found on the surface of certain prostate cancer cells. Researchers wanted to see if CXCR1 affects how cancer cells grow and spread. They compared prostate cancer cells with extra CXCR1 to cells without it. They found that the cells with more CXCR1 grew more slowly, spread less, and formed smaller tumors in mice. This effect seemed to happen because CXCR1 increased the levels of another protein called ITM2A, which helps stop tumor growth. Interestingly, ITM2A is found at lower levels in prostate cancer tissue compared to normal prostate tissue. These results suggest that increasing CXCR1 or ITM2A in prostate cancer cells might be a new way to slow down or stop cancer growth.

**Assessment Category**	**Score**	**Assessment Category**	**Score**	**Assessment Category**	**Score**
Comprehension Score Rater 1	5	Gunning Fog Score	8.7	Words	146
Comprehension Score Rater 2	5	SMOG Index	4.9	Complex words	5
Reading Age (years)	14.5	Coleman Liau Index	12.0	Percentage of complex words	3.42
Flesch-Kincaid Reading Ease	70.1	Automated Readability Index	9.9	Average words per sentence	18.25
Flesch-Kincaid Grade Level	8.0	Sentences	8	Average syllables per word	1.4

Manuscript 18

Almanza-Aguilera E, Martínez-Huélamo M, López-Hernández Y, Guiñón-Fort D, Guadall A, Cruz M, et al. Prediagnostic Plasma Nutrimetabolomics and Prostate Cancer Risk: A Nested Case–Control Analysis Within the EPIC Study. Cancers [Internet]. 2024 Dec 8;16(23):4116. Available from: http://dx.doi.org/10.3390/cancers16234116

Abstract

Background and Objective: Nutrimetabolomics may reveal novel insights into early metabolic alterations and the role of dietary exposures on prostate cancer (PCa) risk. We aimed to prospectively investigate the associations between plasma metabolite concentrations and PCa risk, including clinically relevant tumor subtypes. Methods: We used a targeted and large-scale metabolomics approach to analyze plasma samples of 851 matched PCa case-control pairs from the European Prospective Investigation into Cancer and Nutrition (EPIC) cohort. Associations between metabolite concentrations and PCa risk were estimated by multivariate conditional logistic regression analysis. False discovery rate (FDR) was used to control for multiple testing correction. Results: Thirty-one metabolites (predominately derivatives of food intake and microbial metabolism) were associated with overall PCa risk and its clinical subtypes (p Conclusions: The results from the current nutrimetabolomics study suggest that apart from early metabolic deregulations, some biomarkers of food intake might be related to PCa risk, especially advanced and fatal PCa. Further independent and larger studies are needed to validate our results.

Keywords:

EPIC, nested case–control, nutrimetabolomics, prostate cancer

<u>Original Lay Summary</u>

Previous studies using a metabolomics approach in relation to prostate cancer (PCa) risk assessment have focused on endogenous metabolism. As far as we know, the associations between circulating metabolites derived from exogenous sources, such as diet or lifestyle, and PCa risk have not been widely studied. In our work, we examined the associations between endogenous and exogenous plasma metabolites and PCa risk in a case–control study conducted in the frame of the EPIC study. The main findings of our study include novel negative and positive associations of food metabolites, such as cyclamate and biomarkers of plant-based foods, respectively, with PCa cancer risk. Furthermore, a few microbial metabolites were associated with PCa risk, suggesting the potential role of microbiota in prostate cancer development. Our results reinforce the use of nutritional metabolomics to deepen the understanding of the complex role of diet and its potential mechanisms of action in prostate carcinogenesis.

Assessment Category	Score	Assessment Category	Score	Assessment Category	Score
Comprehension Score Rater 1	5	Gunning Fog Score	20.1	Words	150
Comprehension Score Rater 2	4	SMOG Index	14.7	Complex words	39
Reading Age (years)	22.5	Coleman Liau Index	17.0	Percentage of complex words	26.0
Flesch-Kincaid Reading Ease	17.3	Automated Readability Index	17.3	Average words per sentence	25.0
Flesch-Kincaid Grade Level	17.1	Sentences	6	Average syllables per word	1.94

Lay Summary (Simple Prompt)

Prostate cancer is one of the most common cancers in men, and understanding how diet and metabolism influence its development could help improve prevention strategies. In this study, researchers analyzed blood samples from men collected years before they were diagnosed with prostate cancer. By comparing these samples to those from men without cancer, they identified specific substances in the blood, many linked to diet and gut bacteria, that were associated with a higher or lower risk of developing prostate cancer, including its more aggressive forms. These findings suggest that certain metabolic changes and dietary factors may play a role in prostate cancer risk even before the disease appears. Identifying these early markers could help in developing new ways to detect prostate cancer risk sooner and guide future research on how diet and metabolism influence cancer development.

**Assessment Category**	**Score**	**Assessment Category**	**Score**	**Assessment Category**	**Score**
Comprehension Score Rater 1	5	Gunning Fog Score	17.6	Words	136
Comprehension Score Rater 2	4	SMOG Index	12.4	Complex words	23
Reading Age (years)	21.5	Coleman Liau Index	16.5	Percentage of complex words	16.91
Flesch-Kincaid Reading Ease	31.8	Automated Readability Index	18.0	Average words per sentence	27.2
Flesch-Kincaid Grade Level	15.6	Sentences	5	Average syllables per word	1.74

Lay Summary (Extended Prompt)

This study looked at whether certain substances in the blood, influenced by diet, are linked to the risk of developing prostate cancer. Researchers analyzed blood samples from men in a large European health study to compare those who later developed prostate cancer with those who did not. They found 31 specific substances, many related to food and how the body processes it, that were connected to the risk of prostate cancer, including more serious types of the disease. These findings suggest that what we eat may affect our chances of getting prostate cancer, possibly through early changes in the body's metabolism. Understanding these connections could help identify people at higher risk and guide future research on how diet influences cancer development. More studies are needed to confirm these results and explore how this knowledge can be used to improve prostate cancer prevention.

**Assessment Category**	**Score**	**Assessment Category**	**Score**	**Assessment Category**	**Score**
Comprehension Score Rater 1	5	Gunning Fog Score	14.3	Words	142
Comprehension Score Rater 2	5	SMOG Index	10.1	Complex words	18
Reading Age (years)	18.5	Coleman Liau Index	14.8	Percentage of complex words	12.68
Flesch-Kincaid Reading Ease	49.4	Automated Readability Index	14.9	Average words per sentence	23.67
Flesch-Kincaid Grade Level	12.3	Sentences	6	Average syllables per word	1.58

Manuscript 19

Ghezzo S, Bharathi PG, Duan H, Mapelli P, Sorgo P, Davidzon GA, et al. The Challenge of External Generalisability: Insights from the Bicentric Validation of a [68Ga]Ga-PSMA-11 PET Based Radiomics Signature for Primary Prostate Cancer Characterisation Using Histopathology as Reference. Cancers [Internet]. 2024 Dec 7;16(23):4103. Available from: http://dx.doi.org/10.3390/cancers16234103

Abstract

Background: PSMA PET radiomics is a promising tool for primary prostate cancer (PCa) characterisation. However, small single-centre studies and lack of external validation hinder definitive conclusions on the potential of PSMA PET radiomics in the initial workup of PCa. We aimed to validate a radiomics signature in a larger internal cohort and in an external cohort from a separate centre. Methods: One hundred and twenty-seven PCa patients were retrospectively enrolled across two independent hospitals. The first centre (IRCCS San Raffaele Scientific Institute, Centre 1) contributed 62 [68Ga]Ga-PSMA-11 PET scans, 20 patients classified as low-grade (ISUP grade 68Ga]Ga-PSMA-11 PET scans, and 49 low-grade and 16 high-grade patients. A radiomics model previously generated in Centre 1 was tested on the two cohorts separately and afterward on the entire dataset. Then, we evaluated whether the radiomics features selected in the previous investigation could generalise to new data. Several machine learning (ML) models underwent training and testing using 100-fold Monte Carlo cross-validation, independently at both Centre 1 and Centre 2, with a 70-30% train-test split. Additionally, models were trained in one centre and tested in the other, and vice versa. Furthermore, data from both centres were combined for training and testing using Monte Carlo cross-validation. Finally, a new radiomics signature built on this bicentric dataset was proposed. Several performance metrics were computed. Results: The previously generated radiomics signature resulted in an area under the receiver operating characteristic curve (AUC) of 80.4% when tested on Centre 1, while it generalised poorly to Centre 2, where it reached an AUC of 62.7%. When the whole cohort was considered, AUC was 72.5%.

Similarly, new ML models trained on the previously selected features yielded, at best, an AUC of 80.9% for Centre 1 and performed at chance for Centre 2 (AUC of 49.3%). A new signature built on this bicentric dataset reached, at best, an average AUC of 91.4% in the test set. Conclusions: The satisfying performance of radiomics models when used in the original development settings, paired with the poor performance otherwise observed, emphasises the need to consider centre-specific factors and dataset characteristics when developing radiomics models. Combining radiomics datasets is a viable strategy to reduce such centre-specific biases, but external validation is still needed.

Keywords:

PSMA, external validation, machine learning, prostate cancer, radiomics

<u>Original Lay Summary</u>

Despite the promising results of radiomics for the characterisation of primary prostate cancer, its use in clinical practice is still limited due to a lack of results validation. In this study, we validated a previously published radiomics signature in a bicentric setting. Then, we also explored the role of the previously identified radiomics features if models are trained on new data. Furthermore, we investigated the performance of radiomics analysis if feature selection and model training are performed in the bicentric cohort. Results show that radiomics models work well when tested in their development settings, but generalise poorly to new datasets, thus warning about the need to interpret with caution results from single-centre studies or studies lacking proper external validation.

**Assessment Category**	**Score**	**Assessment Category**	**Score**	**Assessment Category**	**Score**
Comprehension Score Rater 1	4	Gunning Fog Score	19.3	Words	120
Comprehension Score Rater 2	4	SMOG Index	13.9	Complex words	29
Reading Age (years)	22.5	Coleman Liau Index	16.9	Percentage of complex words	24.17
Flesch-Kincaid Reading Ease	20.3	Automated Readability Index	16.7	Average words per sentence	24.0
Flesch-Kincaid Grade Level	16.4	Sentences	5	Average syllables per word	1.92

Lay Summary (Simple Prompt)

Prostate cancer is a common disease, and accurately identifying its severity is crucial for choosing the right treatment. A new imaging method called PSMA PET, combined with advanced computer analysis known as radiomics, shows promise in helping doctors better understand prostate cancer. However, tools like this often work well in the hospital where they were developed but may not perform as well elsewhere. In this study, we tested a radiomics model using prostate cancer scans from two different hospitals to see if it could deliver reliable results in both settings. While the model performed well at the original hospital, it was less accurate at the other. By combining data from both hospitals, we created a new model that showed much better accuracy. This research highlights the importance of using diverse data when developing diagnostic tools to ensure they work well for patients everywhere.

**Assessment Category**	**Score**	**Assessment Category**	**Score**	**Assessment Category**	**Score**
Comprehension Score Rater 1	5	Gunning Fog Score	15.7	Words	143
Comprehension Score Rater 2	5	SMOG Index	11.4	Complex words	27
Reading Age (years)	19.5	Coleman Liau Index	15.0	Percentage of complex words	18.88
Flesch-Kincaid Reading Ease	39.4	Automated Readability Index	13.5	Average words per sentence	20.43
Flesch-Kincaid Grade Level	12.8	Sentences	7	Average syllables per word	1.73

Lay Summary (Extended Prompt)

Prostate cancer is a common disease in men, and doctors are looking for better ways to understand how aggressive it is. This study tested a new method called radiomics, which uses advanced computer programs to analyze medical images from PET scans. We wanted to see if a radiomics model that worked well in one hospital could also work in another hospital with different patients. We found that while the model performed well in the hospital where it was created, it didn't work as well in the other hospital. However, when we combined data from both hospitals, the new model performed much better. This shows that using data from different places can help create more reliable tools for diagnosing and treating prostate cancer. In the future, this approach could help doctors make more accurate decisions, leading to better care for patients.

**Assessment Category**	**Score**	**Assessment Category**	**Score**	**Assessment Category**	**Score**
Comprehension Score Rater 1	4	Gunning Fog Score	13.7	Words	140
Comprehension Score Rater 2	5	SMOG Index	9.8	Complex words	20
Reading Age (years)	17.5	Coleman Liau Index	13.2	Percentage of complex words	14.29
Flesch-Kincaid Reading Ease	54.2	Automated Readability Index	11.7	Average words per sentence	20.0
Flesch-Kincaid Grade Level	10.7	Sentences	7	Average syllables per word	1.56

Manuscript 20

Ueki H, Terakawa T, Hara T, Uemura M, Okamura Y, Suzuki K, et al. Utility of Machine Learning Models to Predict Lymph Node Metastasis of Japanese Localized Prostate Cancer. Cancers [Internet]. 2024 Dec 5;16(23):4073. Available from: http://dx.doi.org/10.3390/cancers16234073

Abstract

Background/objectives: Extended pelvic lymph node dissection is a crucial surgical technique for managing intermediate to high-risk prostate cancer. Accurately predicting lymph node metastasis before surgery can minimize unnecessary lymph node dissections and their associated complications. This study assessed the efficacy of various machine learning models for predicting lymph node metastasis in a cohort of Japanese patients who underwent robot-assisted laparoscopic radical prostatectomy.

Methods: Data from 625 patients who underwent extended pelvic lymph node dissection or standard dissection with lymph node metastasis between October 2010 and February 2023 were analyzed. Four machine learning models-Random Forest, Light Gradient-Boosting Machine, Logistic Regression, and Support Vector Machine-were used to predict lymph node metastasis. Their performance was assessed using receiver operating characteristic curves, a decision curve analysis, and predictive values at different thresholds.

Results: Lymph node metastasis was observed in 34 patients (5.4%). The Light Gradient-Boosting Machine had the highest AUC of 0.924, followed by the Random Forest model with an AUC of 0.894. The decision curve analysis indicated substantial net benefits for both models, particularly at low threshold probabilities. The Light Gradient-Boosting Machine demonstrated superior accuracy, achieving 95.6% at the 0.05 threshold and 96.7% at the 0.10 threshold, outperforming other models and conventional nomograms in the validation dataset.

Conclusion: Machine learning models, especially Light Gradient-Boosting Machine and Random Forest, show significant potential for predicting lymph node metastasis in prostate cancer, thereby aiding in reducing unnecessary surgical interventions.

Keywords:

lymph node metastasis, machine learning, prostate cancer

<u>Original Lay Summary</u>

In this study, machine learning models were developed to predict lymph node invasion in Japanese prostate cancer patients, using a structured approach to improve predictive accuracy. Traditional methods, such as the Briganti nomogram, have been widely used but may lack flexibility and adaptability to diverse clinical datasets. Our machine learning models, especially Light Gradient-Boosting Machine and Random Forest, showed higher flexibility and accuracy compared to conventional models. Through a thorough data analysis and feature engineering, including the use of predictive features such as PSA levels and tumor stage, we enhanced model performance and interpretability. A decision curve analysis indicated significant clinical benefits, suggesting that these models can better identify candidates for lymph node dissection, thereby minimizing unnecessary procedures. However, validation with larger and multi-institutional datasets is needed to confirm the utility of these models in broader clinical settings.

**Assessment Category**	**Score**	**Assessment Category**	**Score**	**Assessment Category**	**Score**
Comprehension Score Rater 1	5	Gunning Fog Score	20.8	Words	140
Comprehension Score Rater 2	5	SMOG Index	15.4	Complex words	43
Reading Age (years)	24.0	Coleman Liau Index	20.5	Percentage of complex words	30.71
Flesch-Kincaid Reading Ease	4.9	Automated Readability Index	19.3	Average words per sentence	23.33
Flesch-Kincaid Grade Level	18.4	Sentences	6	Average syllables per word	2.11

Lay Summary (Simple Prompt)

Prostate cancer can spread to nearby lymph nodes, making it important to identify which patients are at risk before surgery. This helps doctors decide whether to remove these lymph nodes, a procedure that can cause complications if done unnecessarily. In this study, we explored how machine learning, a type of advanced computer technology, can help predict the spread of prostate cancer to lymph nodes in Japanese patients. We analyzed data from 625 men who had prostate cancer surgery and tested different machine learning models to see which one worked best. The Light Gradient-Boosting Machine model showed the highest accuracy, correctly identifying patients at risk over 95% of the time. Our findings suggest that using these machine learning models can help doctors make better decisions about surgery, potentially reducing the need for unnecessary procedures and improving patient care.

**Assessment Category**	**Score**	**Assessment Category**	**Score**	**Assessment Category**	**Score**
Comprehension Score Rater 1	5	Gunning Fog Score	15.3	Words	138
Comprehension Score Rater 2	5	SMOG Index	11.3	Complex words	23
Reading Age (years)	19.5	Coleman Liau Index	15.7	Percentage of complex words	16.67
Flesch-Kincaid Reading Ease	38.2	Automated Readability Index	15.3	Average words per sentence	23.0
Flesch-Kincaid Grade Level	13.6	Sentences	6	Average syllables per word	1.72

<u>Lay Summary (Extended Prompt)</u>

Prostate cancer can spread to nearby lymph nodes, which often requires surgeons to remove these nodes during surgery. However, removing lymph nodes when it's not necessary can cause complications. This study aimed to find a better way to predict if prostate cancer has spread to the lymph nodes before surgery. Researchers analyzed data from 625 Japanese men with prostate cancer who had surgery to remove their prostate and lymph nodes. They used different computer-based tools, called machine learning models, to see which could best predict the spread of cancer. The Light Gradient-Boosting Machine and Random Forest models were the most accurate, correctly identifying cases of cancer spread in over 95% of patients. These findings suggest that using machine learning can help doctors make better decisions about surgery, reducing the need for unnecessary procedures and improving patient care.

**Assessment Category**	**Score**	**Assessment Category**	**Score**	**Assessment Category**	**Score**
Comprehension Score Rater 1	5	Gunning Fog Score	13.7	Words	139
Comprehension Score Rater 2	5	SMOG Index	10.3	Complex words	22
Reading Age (years)	18.5	Coleman Liau Index	15.5	Percentage of complex words	15.83
Flesch-Kincaid Reading Ease	47.9	Automated Readability Index	13.6	Average words per sentence	19.86
Flesch-Kincaid Grade Level	11.5	Sentences	7	Average syllables per word	1.64

Manuscript 21

Drab A, Wdowiak K, Kanadys W, Zajączkowski K, Koczkodaj P, Religioni U, et al. Diabetes Mellitus and Prostate Cancer Risk—A Systematic Review and Meta-Analysis. Cancers [Internet]. 2024 Nov 29;16(23):4010. Available from: http://dx.doi.org/10.3390/cancers16234010

Abstract

Introduction: Prostate cancer is the second most commonly diagnosed malignant tumor worldwide and poses a significant challenge to public health. This systematic review and meta-analysis aims to investigate the association between diabetes mellitus and the risk of developing prostate cancer.

Methods: We conducted a search of PubMed, Embase, and the Cochrane Library from 1998 to 2024. The risk of bias within the included studies was assessed using the Newcastle-Ottawa Scale. The DerSimonian-Laird random-effect model was employed for the meta-analysis. Heterogeneity was evaluated using a forest plot and statistically assessed via the Q test, I2 index, and p-values.

Results: Forty-three studies involving a total of 3,746,769 patients were included. Both case-control (pOR = 0.68, 95% CI: 0.61-0.97; I2 = 92.24%) and cohort studies (pRR = 0.71, 95% CI: 0.59-0.99; I2 = 85.41%) suggest that diabetes mellitus is associated with a reduced risk of prostate cancer, though with significant heterogeneity ($p < 0.05$). Subgroup analysis revealed that the risk of developing prostate cancer was significantly higher in patients with a family history of prostate cancer (pRR = 1.25, 95% CI: 1.16-1.35; I2 = 69.51%).

Conclusions: Our meta-analysis of recent observational studies indicates that diabetes mellitus is associated with a reduced risk of developing prostate cancer.

Keywords:

diabetes mellitus, meta-analysis, prostate cancer

<u>Original Lay Summary</u>

The incidence of prostate cancer is increasing globally. The aim of this meta-analysis was examine the association between diabetes and risk of prostate cancer. We systematically searched PubMed, EMBASE, and the Cochrane Library databases to identify eligible articles. A total of 41 articles were included in this study. The results showed that risk of prostate cancer decreased in diabetes patients (case–control studies: pOR = 0.68, 95% CI: 0.61–0.97; I2 = 92.24%; cohort studies: pRR = 0.71, 95% CI: 0.59–0.99; I2 = 85.41%). Our meta-analysis suggests a protective effect against prostate cancer risk.

**Assessment Category**	**Score**	**Assessment Category**	**Score**	**Assessment Category**	**Score**
Comprehension Score Rater 1	5	Gunning Fog Score	10.0	Words	102
Comprehension Score Rater 2	3	SMOG Index	6.9	Complex words	19
Reading Age (years)	12.5	Coleman Liau Index	9.6	Percentage of complex words	18.63
Flesch-Kincaid Reading Ease	56.0	Automated Readability Index	2.5	Average words per sentence	7.29
Flesch-Kincaid Grade Level	7.3	Sentences	14	Average syllables per word	1.7

<u>Lay Summary (Simple Prompt)</u>

Prostate cancer is one of the most common cancers in men worldwide. Some studies have suggested that diabetes might affect the risk of developing prostate cancer, but the results have been mixed. To better understand this connection, we reviewed and analyzed data from 43 studies involving over 3.7 million people. Our findings showed that men with diabetes seem to have a lower risk of developing prostate cancer compared to those without diabetes. However, this relationship varied between different studies, and men with a family history of prostate cancer were found to be at higher risk regardless of their diabetes status. These results suggest that diabetes may play a role in prostate cancer risk, but more research is needed to understand why this is the case. Understanding these links could help improve cancer prevention strategies and guide future research on prostate health.

**Assessment Category**	**Score**	**Assessment Category**	**Score**	**Assessment Category**	**Score**
Comprehension Score Rater 1	5	Gunning Fog Score	13.3	Words	142
Comprehension Score Rater 2	5	SMOG Index	9.7	Complex words	22
Reading Age (years)	17.5	Coleman Liau Index	14.5	Percentage of complex words	15.49
Flesch-Kincaid Reading Ease	51.2	Automated Readability Index	11.7	Average words per sentence	17.75
Flesch-Kincaid Grade Level	10.5	Sentences	8	Average syllables per word	1.63

<u>Lay Summary (Extended Prompt)</u>

People with diabetes may have a lower chance of getting prostate cancer, but the reasons for this are not fully understood. To find out more, we reviewed 43 studies that included nearly 3.8 million men. We carefully analyzed the data to see if there is a clear link between diabetes and prostate cancer risk. Our results showed that men with diabetes seem to have a lower risk of developing prostate cancer compared to those without diabetes. However, men who have a family history of prostate cancer still face a higher risk, even if they have diabetes. These findings are important because they help doctors and researchers better understand how diabetes might affect prostate cancer risk. This knowledge could lead to improved ways of preventing and detecting prostate cancer, especially for men with different health backgrounds.

**Assessment Category**	**Score**	**Assessment Category**	**Score**	**Assessment Category**	**Score**
Comprehension Score Rater 1	5	Gunning Fog Score	13.0	Words	136
Comprehension Score Rater 2	5	SMOG Index	9.4	Complex words	21
Reading Age (years)	16.5	Coleman Liau Index	13.7	Percentage of complex words	15.44
Flesch-Kincaid Reading Ease	54.6	Automated Readability Index	10.7	Average words per sentence	17.0
Flesch-Kincaid Grade Level	9.9	Sentences	8	Average syllables per word	1.6

Manuscript 22

Alghamdi D, Kernohan N, Li C, Nabi G. Survival Outcomes for Men over 80 Years Undergoing Transrectal Ultrasound-Guided Prostate Biopsy: A Prospective Analysis. Cancers [Internet]. 2024 Nov 28;16(23):3995. Available from: http://dx.doi.org/10.3390/cancers16233995

Abstract

Introduction: Prostate cancer is the second most prevalent cancer among elderly males in Western countries. TRUS biopsy remains a standard diagnosing approach for prostate cancer but poses notable risks, particularly in older men, including complications such as sepsis, acute retention, and rectal bleeding, which can lead to substantial morbidity and mortality. This study aimed to evaluate cancer-specific survival outcomes in men aged over 80 years and whether there is any cancer-specific survival advantage for TRUS biopsy procedure. Methods: Between January 2005 and December 2015, we studied outcomes of 200 patients (median age, 82 years) with elevated prostate-specific antigen (PSA) levels (>4.0 ng/mL) and/or abnormal digital rectal examination (DRE) who underwent TRUS biopsy. Each participant was followed up until death using an electronic system and a unique identifier in a defined geographical area. Cancer-specific and overall survival analyses were carried out utilising SPSS, while R Project was employed to construct and evaluate two nomograms survival duration and predict the risk of death post-biopsy. All statistical tests were two-tailed, with significance set at p Results: Amongst the participants, only 24 patients were alive at the end of follow-up (median age, 91 years). The PSA levels ranged from 4.88 to 102.7 ng/mL. Log-rank and Breslow tests indicated that higher PSA levels, the development of metastases, and ISUP grade group 8-10 were associated with shorter survival times. Age, co-morbid conditions, and tumour type were incorporated into the nomogram due to their clinical significance. Patients aged 88 years faced higher mortality risks. Complications from the biopsy increased mortality risks in both cancerous and benign cases, and metastasis significantly heightened the likelihood of death.

However, co-morbid conditions did not influence survival probability. Conclusions: Our findings underscore that older age (specifically 80 years and above), high Gleason score, metastasis, and elevated PSA levels are predictive of poorer survival outcomes in elderly men following TRUS biopsy.

Keywords:

PSA, nomogram, prognosis, prostate cancer, transrectal ultrasound biopsy (TRUS)

<u>Original Lay Summary</u>

Prostate cancer is common among older men, and its diagnosis often involves a procedure called transrectal ultrasound (TRUS) biopsy, which helps detect cancer but also carries risks like infection and bleeding, particularly for elderly patients. This study evaluated the survival of men over 80 years of age to determine if undergoing a TRUS biopsy might improve their chances of surviving prostate cancer. By examining the records of 200 patients who had this biopsy, the study identified factors linked to poorer survival, including high levels of prostate-specific antigen, advanced cancer grade, and metastases. These findings can assist in a more accurate evaluation of the risks associated with TRUS biopsy in elderly patients and may guide future research toward developing safer or alternative strategies for diagnosing and managing prostate cancer in this population.

**Assessment Category**	**Score**	**Assessment Category**	**Score**	**Assessment Category**	**Score**
Comprehension Score Rater 1	3	Gunning Fog Score	23.5	Words	132
Comprehension Score Rater 2	3	SMOG Index	16.8	Complex words	34
Reading Age (years)	24.0	Coleman Liau Index	16.6	Percentage of complex words	25.76
Flesch-Kincaid Reading Ease	13.8	Automated Readability Index	21.0	Average words per sentence	33.0
Flesch-Kincaid Grade Level	19.5	Sentences	4	Average syllables per word	1.89

<u>Lay Summary (Simple Prompt)</u>

Prostate cancer is common in older men, and diagnosing it often involves a procedure called a transrectal ultrasound-guided biopsy (TRUS biopsy). However, this procedure can carry serious risks, especially for men over 80, including infections, bleeding, and other complications. Our study followed 200 men aged 80 and above who underwent this biopsy to understand how it affected their survival. We found that men with higher prostate-specific antigen (PSA) levels, aggressive cancer types, and cancer that had spread to other parts of the body had shorter survival times. Interestingly, the presence of other health conditions did not significantly affect survival, but complications from the biopsy itself increased the risk of death. These results suggest that for very elderly men, careful consideration is needed before performing a TRUS biopsy, as the risks may outweigh the benefits depending on individual health factors and cancer severity.

**Assessment Category**	**Score**	**Assessment Category**	**Score**	**Assessment Category**	**Score**
Comprehension Score Rater 1	5	Gunning Fog Score	19.9	Words	144
Comprehension Score Rater 2	5	SMOG Index	14.3	Complex words	37
Reading Age (years)	21.5	Coleman Liau Index	16.0	Percentage of complex words	25.69
Flesch-Kincaid Reading Ease	26.2	Automated Readability Index	16.0	Average words per sentence	24.0
Flesch-Kincaid Grade Level	15.6	Sentences	6	Average syllables per word	1.85

Lay Summary (Extended Prompt)

Prostate cancer is common in older men, and diagnosing it often requires a procedure called a transrectal ultrasound-guided biopsy (TRUS biopsy). However, this procedure can be risky for men over 80, leading to complications like infections or bleeding. Our study looked at 200 men aged 80 and above who had this biopsy to see how it affected their survival. We found that men with high prostate-specific antigen (PSA) levels, cancer that had spread (metastasis), or aggressive tumors had shorter survival times. Older age and biopsy-related complications also increased the risk of death, even in men without cancer. Interestingly, other health problems (co-morbid conditions) did not seem to affect survival. These findings can help doctors and patients make better decisions about whether to perform a biopsy in very elderly men, balancing the potential benefits of detecting cancer with the risks involved.

Assessment Category	Score	Assessment Category	Score	Assessment Category	Score
Comprehension Score Rater 1	5	Gunning Fog Score	16.8	Words	144
Comprehension Score Rater 2	5	SMOG Index	12.2	Complex words	31
Reading Age (years)	19.5	Coleman Liau Index	14.3	Percentage of complex words	21.53
Flesch-Kincaid Reading Ease	39.7	Automated Readability Index	13.0	Average words per sentence	20.57
Flesch-Kincaid Grade Level	12.8	Sentences	7	Average syllables per word	1.73

Manuscript 23

Fichtel-Epstein C, Huang J, Rich BJ, Taswell CS, Isrow D, Jin W. Ultra-Processed Food and Prostate Cancer Risk: A Systemic Review and Meta-Analysis. Cancers [Internet]. 2024 Nov 26;16(23):3953. Available from: http://dx.doi.org/10.3390/cancers16233953

Abstract

Background/Objectives: Prostate cancer is the second leading cause of cancer death among American men, following lung cancer. While diet and exercise have been extensively studied in relation to prostate cancer prevention, the evidence remains inconclusive. Methods: A comprehensive literature search was performed to identify observational studies investigating the association between ultra-processed food (UPF) consumption and prostate cancer risk and mortality, determined by the NOVA classification system. In addition, we conducted subgroup analyses to assess the association based on study design, age, and data collection methods. Results: Six studies were identified, including four cohort studies and two case-control studies. No significant association was found between high UPF consumption and increased risk of prostate cancer [RR = 1.02, 95% confidence interval (CI) = 0.96-1.08, n = 5]. However, there was a slight increase in mortality (RR = 1.15, 95% CI = 0.99-1.35, n = 2). A subgroup analysis by the dietary assessment method revealed an RR of 1.01 (95% CI = 0.93-1.09) for studies using the food frequency questionnaire (FFQ) and 1.04 (95% CI = 0.93-1.16) for studies using 24-h recalls. There was no significant heterogeneity among the studies (I2 = 0, p = 0.82). Conclusions: This meta-analysis suggests no significant association between high UPF consumption and prostate cancer risk. Given the known associations with other chronic diseases, the potential public health implications of reducing UPF consumption remain important. Further research with the use of more robust food assignment systems and more precise dietary assessments is needed to clarify the role of UPF in prostate cancer development.

Keywords:

chronic inflammation, dietary pattern, prostate cancer, ultra-processed food

Original Lay Summary

Prostate cancer is the second leading cause of cancer-related deaths among men in the United States. While genetic and environmental factors have been extensively studied, the role of modifiable lifestyle factors, particularly diet, is less understood. Ultra-processed foods, known for their high content of additives and preservatives and low levels of whole-food ingredients, have been associated with various cancers. This study aimed to determine whether ultra-processed food consumption is linked to increased prostate cancer incidence or mortality. After reviewing data from six studies, we found no significant association between ultra-processed food intake and prostate cancer risk. However, higher consumption was correlated with a slight increase in mortality. Further research is needed to clarify these associations and improve dietary assessment methods in future studies.

**Assessment Category**	**Score**	**Assessment Category**	**Score**	**Assessment Category**	**Score**
Comprehension Score Rater 1	5	Gunning Fog Score	16.1	Words	128
Comprehension Score Rater 2	5	SMOG Index	11.8	Complex words	29
Reading Age (years)	20.5	Coleman Liau Index	18.5	Percentage of complex words	22.66
Flesch-Kincaid Reading Ease	27.0	Automated Readability Index	15.1	Average words per sentence	18.29
Flesch-Kincaid Grade Level	14.0	Sentences	7	Average syllables per word	1.91

Lay Summary (Simple Prompt)

Prostate cancer is a major health concern, being the second leading cause of cancer-related deaths among men in the United States. Some studies suggest that what we eat might influence the risk of developing prostate cancer, but the role of ultra-processed foods—like packaged snacks, sugary drinks, and ready-to-eat meals—is still unclear. To better understand this, we reviewed and analyzed data from six studies that explored the link between ultra-processed food consumption and prostate cancer risk. Our findings showed no clear connection between eating large amounts of these foods and an increased risk of developing prostate cancer. However, there was a slight indication that higher intake might be linked to an increased risk of dying from prostate cancer. While this research suggests no strong link between ultra-processed foods and prostate cancer risk, reducing their consumption remains important for overall health. Further studies are needed to confirm these findings.

**Assessment Category**	**Score**	**Assessment Category**	**Score**	**Assessment Category**	**Score**
Comprehension Score Rater 1	5	Gunning Fog Score	12.4	Words	153
Comprehension Score Rater 2	5	SMOG Index	8.6	Complex words	15
Reading Age (years)	18.5	Coleman Liau Index	15.7	Percentage of complex words	9.8
Flesch-Kincaid Reading Ease	52.5	Automated Readability Index	14.7	Average words per sentence	21.86
Flesch-Kincaid Grade Level	11.4	Sentences	7	Average syllables per word	1.56

<u>Lay Summary (Extended Prompt)</u>

Prostate cancer is one of the leading causes of cancer deaths in men. Some studies suggest that what we eat might affect the risk of getting this disease. This study looked at whether eating a lot of ultra-processed foods—like packaged snacks, sugary drinks, and ready-to-eat meals—might increase the risk of prostate cancer. Researchers reviewed data from six studies involving different groups of men to find patterns. The results showed no clear link between eating more ultra-processed foods and a higher chance of getting prostate cancer. However, there was a small sign that eating these foods might slightly raise the risk of dying from prostate cancer. Even though this connection was not strong, eating fewer ultra-processed foods is still important for overall health. More research is needed to better understand how these foods may affect prostate cancer risk and to help guide healthier eating habits.

**Assessment Category**	**Score**	**Assessment Category**	**Score**	**Assessment Category**	**Score**
Comprehension Score Rater 1	5	Gunning Fog Score	10.1	Words	149
Comprehension Score Rater 2	5	SMOG Index	6.6	Complex words	10
Reading Age (years)	15.5	Coleman Liau Index	13.9	Percentage of complex words	6.71
Flesch-Kincaid Reading Ease	65.3	Automated Readability Index	11.7	Average words per sentence	18.63
Flesch-Kincaid Grade Level	8.8	Sentences	8	Average syllables per word	1.45

Manuscript 24

Fonseca LRS, Carreira RJP, Feijó M, Cavaco JEB, Cardoso HJ, Vaz CV, et al. Downregulated Regucalcin Expression Induces a Cancer-like Phenotype in Non-Neoplastic Prostate Cells and Augments the Aggressiveness of Prostate Cancer Cells: Interplay with the G Protein-Coupled Oestrogen Receptor? Cancers [Internet]. 2024 Nov 24;16(23):3932. Available from: http://dx.doi.org/10.3390/cancers16233932

Abstract

Background/objectives: Regucalcin (RGN) is a calcium-binding protein and an oestrogen target gene, which has been shown to play essential roles beyond calcium homeostasis. Decreased RGN expression was identified in several cancers, including prostate cancer (PCa). However, it is unknown if the loss of RGN is a cause or a consequence of malignancy. Also, it needs confirmation if RGN oestrogenic regulation occurs through the G-protein-coupled oestrogen receptor (GPER). This study investigates how RGN knockdown affects prostate cell fate and metabolism and highlights the GPER/RGN interplay in PCa.

Methods: Bioinformatic analysis assessed the relationship between RGN expression levels and patients' outcomes. RGN knockdown (siRNA) was performed in non-neoplastic prostate and castration-resistant PCa. Wild-type and RGN knockdown PCa cells were treated with the GPER agonist G1. Viability (MTT), proliferation (Ki-67 immunocytochemistry), apoptosis (caspase-3-like activity) and migration (Transwell assays) were evaluated. Spectrophotometric analysis was used to determine glucose consumption, lactate production and lactate dehydrogenase activity. Lipid content was assessed using the Oil Red assay.

Results/conclusions: Bioinformatic analysis showed that the loss of RGN correlates with the development of metastatic PCa and poor survival outcomes. RGN knockdown induced a cancer-like phenotype in PNT1A cells, indicated by increased

cell viability and proliferation and reduced apoptosis. In DU145 PCa cells, RGN knockdown augmented migration and enhanced the glycolytic profile, which indicates increased aggressiveness, in line with patients' data. GPER activation modulated RGN expression in PCa cells and RGN knockdown in DU145 cells influenced GPER actions, which highlighted an interplay between these molecular players with relevance for their potential use as biomarkers or therapeutic targets.

Keywords:

G-protein-coupled oestrogen receptor, metabolism, metastisation, prostate cancer, regucalcin, survival

<u>Original Lay Summary</u>

Regucalcin (RGN) is a calcium-binding protein and an oestrogen target gene. Decreased RGN expression was identified in prostate cancer (PCa). However, it is unknown if the loss of RGN is a cause or a consequence of malignancy. Also, it needs confirmation if RGN oestrogenic regulation occurs through the G-protein-coupled oestrogen receptor (GPER). This study investigates how RGN knockdown affects prostate cell fate and metabolism and highlights the GPER/RGN interplay in PCa. Bioinformatic analysis of patients' data demonstrated that the loss of RGN correlates with the development of metastatic PCa and poor survival outcomes. RGN knockdown in an in vitro approach induced a cancer-like phenotype in non-neoplastic prostate cells and increased the aggressiveness features of PCa cells. GPER activation modulated RGN expression in PCa cells and RGN knockdown influenced GPER actions, which highlighted an interplay between these molecular players with relevance for their potential use as biomarkers or therapeutic targets.

**Assessment Category**	**Score**	**Assessment Category**	**Score**	**Assessment Category**	**Score**
Comprehension Score Rater 1	5	Gunning Fog Score	16.8	Words	154
Comprehension Score Rater 2	5	SMOG Index	12.3	Complex words	36
Reading Age (years)	20.5	Coleman Liau Index	16.9	Percentage of complex words	23.38
Flesch-Kincaid Reading Ease	34.0	Automated Readability Index	14.3	Average words per sentence	19.25
Flesch-Kincaid Grade Level	13.3	Sentences	8	Average syllables per word	1.81

<u>Lay Summary (Simple Prompt)</u>

Prostate cancer is a common and serious disease, and understanding how it develops can help improve treatments. This study focuses on a protein called regucalcin, which helps regulate important cell functions. Researchers found that lower levels of regucalcin are linked to more aggressive prostate cancer and poorer patient outcomes. To understand why this happens, they reduced regucalcin levels in both healthy prostate cells and prostate cancer cells. This caused the healthy cells to behave like cancer cells and made cancer cells grow and spread more aggressively. The study also explored how regucalcin interacts with another molecule called the G-protein-coupled oestrogen receptor, which may play a role in cancer progression. These findings suggest that regucalcin could be a valuable marker to identify aggressive prostate cancer and might even serve as a target for new treatments, potentially improving outcomes for patients.

**Assessment Category**	**Score**	**Assessment Category**	**Score**	**Assessment Category**	**Score**
Comprehension Score Rater 1	5	Gunning Fog Score	15.1	Words	141
Comprehension Score Rater 2	5	SMOG Index	11.0	Complex words	25
Reading Age (years)	19.5	Coleman Liau Index	17.0	Percentage of complex words	17.73
Flesch-Kincaid Reading Ease	40.6	Automated Readability Index	14.9	Average words per sentence	20.14
Flesch-Kincaid Grade Level	12.6	Sentences	7	Average syllables per word	1.72

<u>Lay Summary (Extended Prompt)</u>

Prostate cancer can become very aggressive, but the reasons behind this are not fully understood. This study looked at a protein called regucalcin, which helps control important cell functions. We found that when prostate cells lose regucalcin, they start behaving more like cancer cells—growing faster, surviving longer, and spreading more easily. In prostate cancer cells, losing regucalcin made them even more aggressive. We also discovered that regucalcin is linked to another protein called the G-protein-coupled oestrogen receptor, which helps control cell growth. Changes in regucalcin levels affected how this receptor works, suggesting they interact closely. These findings are important because they show that regucalcin may play a key role in how prostate cancer develops and spreads. Understanding this connection could help doctors find new ways to detect aggressive prostate cancer early or develop better treatments to stop it from getting worse.

**Assessment Category**	**Score**	**Assessment Category**	**Score**	**Assessment Category**	**Score**
Comprehension Score Rater 1	5	Gunning Fog Score	15.0	Words	143
Comprehension Score Rater 2	5	SMOG Index	10.8	Complex words	28
Reading Age (years)	19.5	Coleman Liau Index	17.2	Percentage of complex words	19.58
Flesch-Kincaid Reading Ease	44.3	Automated Readability Index	13.9	Average words per sentence	17.88
Flesch-Kincaid Grade Level	11.5	Sentences	8	Average syllables per word	1.71

Manuscript 25

Zhang H, Patkar S, Lis R, Merino MJ, Pinto PA, Choyke PL, et al. Masked Image Modeling Meets Self-Distillation: A Transformer-Based Prostate Gland Segmentation Framework for Pathology Slides. Cancers [Internet]. 2024 Nov 21;16(23):3897. Available from: http://dx.doi.org/10.3390/cancers16233897

Abstract

Detailed evaluation of prostate cancer glands is an essential yet labor-intensive step in grading prostate cancer. Gland segmentation can serve as a valuable preliminary step for machine-learning-based downstream tasks, such as Gleason grading, patient classification, cancer biomarker building, and survival analysis. Despite its importance, there is currently a lack of a reliable gland segmentation model for prostate cancer. Without accurate gland segmentation, researchers rely on cell-level or human-annotated regions of interest for pathomic and deep feature extraction. This approach is sub-optimal, as the extracted features are not explicitly tailored to gland information. Although foundational segmentation models have gained a lot of interest, we demonstrated the limitations of this approach. This work proposes a prostate gland segmentation framework that utilizes a dual-path Swin Transformer UNet structure and leverages Masked Image Modeling for large-scale self-supervised pretaining. A tumor-guided self-distillation step further fused the binary tumor labels of each patch to the encoder to ensure the encoders are suitable for the gland segmentation step. We united heterogeneous data sources for self-supervised training, including biopsy and surgical specimens, to reflect the diversity of benign and cancerous pathology features. We evaluated the segmentation performance on two publicly available prostate cancer datasets. We achieved state-of-the-art segmentation performance with a test mDice of 0.947 on the PANDA dataset and a test mDice of 0.664 on the SICAPv2 dataset.

Keywords:

Deep Learning, digital pathology, gland segmentation, prostate cancer, whole slide image

<u>Original Lay Summary</u>

The accurate characterization of prostate cancer glands is vital for diagnosis and grading, and the segmentation of these glands is critical for quantitative analysis and machine learning models. Human annotation is unrealistic given the size of whole-slide images, yet there lacks a reliable fully automated segmentation framework. This study addressed the limitations of previous state-of-the-art approaches reported on this task, popular segmentation frameworks, and foundational models. Our proposed framework leverages the strength of self-supervised and self-distillation to achieve a high segmentation performance, offering a reliable prostate gland segmentation to pave the way for better downstream analysis of whole-slide images.

**Assessment Category**	**Score**	**Assessment Category**	**Score**	**Assessment Category**	**Score**
Comprehension Score Rater 1	4	Gunning Fog Score	21.5	Words	106
Comprehension Score Rater 2	4	SMOG Index	15.5	Complex words	29
Reading Age (years)	24.0	Coleman Liau Index	18.9	Percentage of complex words	27.36
Flesch-Kincaid Reading Ease	17.1	Automated Readability Index	19.6	Average words per sentence	26.5
Flesch-Kincaid Grade Level	17.5	Sentences	4	Average syllables per word	1.92

<u>Lay Summary (Simple Prompt)</u>

Prostate cancer is one of the most common cancers in men, and accurately identifying cancerous glands in tissue samples is crucial for proper diagnosis and treatment planning. However, this process is time-consuming and often relies on manual work, which can lead to inconsistent results. In this study, we developed an advanced computer model that uses artificial intelligence to automatically detect and outline prostate glands in digital tissue slides. Our model combines cutting-edge techniques, including a special type of deep learning called a Transformer, to improve accuracy and efficiency. We trained the model using a wide range of prostate tissue samples to ensure it can handle both benign and cancerous cases. When tested on large prostate cancer datasets, our model outperformed existing methods, showing exceptional precision. This approach could support pathologists by speeding up the diagnostic process and making prostate cancer assessments more reliable.

**Assessment Category**	**Score**	**Assessment Category**	**Score**	**Assessment Category**	**Score**
Comprehension Score Rater 1	5	Gunning Fog Score	16.0	Words	145
Comprehension Score Rater 2	4	SMOG Index	11.8	Complex words	29
Reading Age (years)	20.5	Coleman Liau Index	17.3	Percentage of complex words	20.0
Flesch-Kincaid Reading Ease	30.6	Automated Readability Index	15.4	Average words per sentence	20.71
Flesch-Kincaid Grade Level	14.1	Sentences	7	Average syllables per word	1.83

Lay Summary (Extended Prompt)

Prostate cancer is one of the most common cancers in men, and accurately identifying prostate glands in tissue samples is important for diagnosing and predicting how the cancer will behave. However, this process is time-consuming and often relies on human experts, which can lead to errors. In this study, we developed a new computer-based method to automatically detect and outline prostate glands in tissue slides. Our approach uses advanced artificial intelligence techniques, including a special type of deep learning model called a Transformer, to improve accuracy. We also combined different types of tissue samples to make the model more reliable in identifying both healthy and cancerous glands. When tested on large public datasets, our method showed excellent performance, even better than existing tools. This could help doctors and researchers analyze prostate cancer more quickly and accurately, leading to better patient care and more reliable research results.

**Assessment Category**	**Score**	**Assessment Category**	**Score**	**Assessment Category**	**Score**
Comprehension Score Rater 1	5	Gunning Fog Score	15.8	Words	148
Comprehension Score Rater 2	4	SMOG Index	11.6	Complex words	28
Reading Age (years)	20.5	Coleman Liau Index	16.5	Percentage of complex words	18.92
Flesch-Kincaid Reading Ease	33.9	Automated Readability Index	15.0	Average words per sentence	21.14
Flesch-Kincaid Grade Level	13.8	Sentences	7	Average syllables per word	1.79

Manuscript 26

Han S, Park S, Kim S, Kwon S, Ko J. Small Leucine Zipper Protein Regulates Glucose Metabolism of Prostate Cancer Cells via Induction of Phosphoglycerate Kinase 1. Cancers [Internet]. 2024 Nov 18;16(22):3861. Available from: http://dx.doi.org/10.3390/cancers16223861

Abstract

Background: Cancer cells exhibit altered metabolism whereby glucose is preferentially utilized to produce lactate through aerobic glycolysis. The increase in lactate production creates an acidic microenvironment that supports tumor progression and metastasis. Human small leucine zipper protein (sLZIP) is involved in the transcriptional regulation of genes related to migration and invasion of prostate cancer. However, the role of sLZIP in modulating glucose metabolism in prostate cancer remains unknown. This study investigates whether sLZIP regulates the transcription of glycolysis-related genes to promote metabolic reprogramming in prostate cancer.

Methods: Depletion of sLZIP resulted in the downregulation of several glycolytic genes, including glucose transporter 1, phosphofructokinase liver type, phosphoglycerate kinase 1 (PGK1), and lactate dehydrogenase. Among these, only PGK1 showed a prominent dose-dependent decrease in mRNA and protein expression after sLZIP silencing.

Results: Mechanistically, increasing or decreasing sLZIP affected the promoter activity of PGK1 in a similar manner. Moreover, the absence of sLZIP attenuated the maximum glycolytic rate in prostate cancer cells. These results were further supported by a reduction in lactate secretion, glucose uptake, and ATP production in sLZIP-knockout prostate cancer cells. sLZIP deficiency hindered cancer growth, as demonstrated by proliferation assays. However, overexpression of PGK1 in sLZIP knockout cells resulted in recovery of aerobic glycolysis. Results of the

xenograft experiment revealed that mice injected with sLZIP knockout cells exhibited a decrease in tumor mass compared to those injected with control cells.

Conclusion: These findings suggest that sLZIP contributes to the metabolic reprogramming of prostate cancer cells via the transcriptional regulation of PGK1.

Keywords:

aerobic glycolysis, metabolic reprogramming, phosphoglycerate kinase 1, prostate cancer, small leucine zipper protein

Original Lay Summary

Prostate cancer is the second leading cause of cancer-related deaths among men, and its recurrence rate is notably high. Depending on the initial treatment, up to 60% of men may experience recurrence within 5 to 10 years after first-line therapy. Our study aims to highlight a novel mechanism for prostate cancer therapy. Human small leucine zipper protein (sLZIP) regulates metabolic reprogramming in prostate cancer, which plays a crucial role in the growth of recurrent prostate cancer. In this study, we found that sLZIP contributes to the metabolic reprogramming of prostate cancer cells via transcriptional regulation of phosphoglycerate kinase 1. The absence of sLZIP attenuated the maximum glycolytic rate and reduced lactate secretion, glucose uptake, and ATP production in prostate cancer. However, overexpression of PGK1 in sLZIP knockout cells resulted in recovery of aerobic glycolysis. sLZIP demonstrates the potential for the development of novel therapeutic approaches and prognostic biomarkers for castration-resistant prostate cancer.

**Assessment Category**	**Score**	**Assessment Category**	**Score**	**Assessment Category**	**Score**
Comprehension Score Rater 1	5	Gunning Fog Score	17.8	Words	155
Comprehension Score Rater 2	5	SMOG Index	12.7	Complex words	39
Reading Age (years)	20.5	Coleman Liau Index	17.4	Percentage of complex words	25.16
Flesch-Kincaid Reading Ease	30.0	Automated Readability Index	14.8	Average words per sentence	19.38
Flesch-Kincaid Grade Level	13.9	Sentences	8	Average syllables per word	1.86

Lay Summary (Simple Prompt)

Prostate cancer cells grow and spread partly because they process sugar (glucose) differently than normal cells, creating an environment that helps tumors thrive. This study focused on a protein called small leucine zipper protein (sLZIP), which was already known to affect how cancer cells move and invade other tissues. However, its role in how prostate cancer cells use glucose was unclear. Researchers found that sLZIP controls the activity of a key enzyme called phosphoglycerate kinase 1 (PGK1), which plays an important role in how cells break down glucose for energy. When sLZIP was removed from prostate cancer cells, the cells produced less energy, grew more slowly, and formed smaller tumors in mice. Interestingly, reintroducing PGK1 reversed these effects. These findings suggest that targeting sLZIP or PGK1 could be a new way to slow down prostate cancer growth by disrupting the cancer cells' energy supply.

**Assessment Category**	**Score**	**Assessment Category**	**Score**	**Assessment Category**	**Score**
Comprehension Score Rater 1	5	Gunning Fog Score	12.4	Words	144
Comprehension Score Rater 2	5	SMOG Index	8.6	Complex words	15
Reading Age (years)	17.5	Coleman Liau Index	15.0	Percentage of complex words	10.42
Flesch-Kincaid Reading Ease	53.8	Automated Readability Index	13.5	Average words per sentence	20.57
Flesch-Kincaid Grade Level	10.9	Sentences	7	Average syllables per word	1.56

Lay Summary (Extended Prompt)

Prostate cancer cells need energy to grow and spread, and they get this energy mainly from sugar (glucose). In this study, we looked at a protein called small leucine zipper protein (sLZIP) to see how it affects how prostate cancer cells use glucose. We found that sLZIP helps turn on a gene called phosphoglycerate kinase 1 (PGK1), which plays an important role in breaking down glucose for energy. When we removed sLZIP from prostate cancer cells, the cells produced less energy, grew more slowly, and formed smaller tumors in mice. However, when we added PGK1 back to these cells, they regained their ability to make energy and grow. This research shows that sLZIP helps prostate cancer cells get the energy they need to grow, and targeting this protein might be a new way to slow down or treat prostate cancer.

**Assessment Category**	**Score**	**Assessment Category**	**Score**	**Assessment Category**	**Score**
Comprehension Score Rater 1	5	Gunning Fog Score	12.5	Words	140
Comprehension Score Rater 2	5	SMOG Index	8.0	Complex words	11
Reading Age (years)	16.5	Coleman Liau Index	11.6	Percentage of complex words	7.86
Flesch-Kincaid Reading Ease	65.9	Automated Readability Index	12.1	Average words per sentence	23.33
Flesch-Kincaid Grade Level	9.9	Sentences	6	Average syllables per word	1.39

Manuscript 27

Washington CG, Deville C. Health Disparities and Inequities in the Utilization of Proton Therapy for Prostate Cancer. Cancers [Internet]. 2024 Nov 15;16(22):3837. Available from: http://dx.doi.org/10.3390/cancers16223837

Abstract

Our study sought to review and summarize the reported health disparities and inequities in the utilization of proton beam therapy (PBT) for prostate cancer. We queried the PubMed search engine through 12/2023 for original publications examining disparate utilization of PBT for prostate cancer. The query terms included the following: prostate cancer AND proton AND (disparities OR IMRT OR race OR insurance OR socioeconomic OR inequities)". Studies were included if they involved United States patients, examined PBT in prostate cancer, and addressed health inequities. From this query, 22 studies met the inclusion criteria, comprising 13 population-based analyses, 5 single-institutional analyses, 3 cost/modeling investigations, and 1 survey-based study. The analyses revealed that in addition to age-related and insurance-related disparities, race and socioeconomic status played significant roles in the receipt of PBT. The likelihood of receiving PBT was lower for non-White patients in population-based and single-institution analyses. Socioeconomic metrics, such as higher median income and higher education level, portended an increased likelihood of receiving PBT. Conclusively, substantial age-based, racial, socioeconomic/insurance-related, and facility-associated disparities and inequities existed for PBT utilization in prostate cancer. The identification of these disparities provides a framework to better address these as the utility of PBT continues to expand across the US and globally.

Keywords:

IMRT, disparities, inequities, insurance, prostate cancer, proton, race, socioeconomic

Original Lay Summary

As technology progresses, the scope and practice of medicine adapts, resulting in treatment applications that improve the precise and potentially current standard of care. The advent and continued use of proton beam therapy for use in patients with prostate cancer is a slowly evolving topic due to the lack of level 1 evidence demonstrating improved efficacy with its use. Despite this, not all patients are provided with equal access to this treatment modality. While there have been several publications utilizing either single-institutional analyses or national medical databases, to date, a comprehensive review summarizing the disparities and inequities in proton beam therapy use in prostate cancer does not exist. Our research sought to elucidate trends in this topic and create a compendium that can be used to inform future study and equitable advances in the field.

**Assessment Category**	**Score**	**Assessment Category**	**Score**	**Assessment Category**	**Score**
Comprehension Score Rater 1	3	Gunning Fog Score	19.7	Words	136
Comprehension Score Rater 2	3	SMOG Index	14.1	Complex words	30
Reading Age (years)	22.5	Coleman Liau Index	15.8	Percentage of complex words	22.06
Flesch-Kincaid Reading Ease	26.2	Automated Readability Index	17.5	Average words per sentence	27.2
Flesch-Kincaid Grade Level	16.4	Sentences	5	Average syllables per word	1.81

Lay Summary (Simple Prompt)

Proton beam therapy is an advanced treatment for prostate cancer, but not everyone has the same access to it. This study reviewed research to understand if factors like age, race, income, education, and health insurance affect who receives this therapy in the United States. By analyzing 22 studies, we found that older individuals, people from minority racial groups, and those with lower income or education levels are less likely to receive proton therapy compared to others. Health insurance coverage and the type of medical facility also play a role. These differences highlight significant health disparities, meaning that not all patients have equal opportunities to benefit from the best available treatments. Recognizing these issues is the first step toward creating fairer healthcare systems, ensuring that more people can access advanced cancer therapies, regardless of their background.

**Assessment Category**	**Score**	**Assessment Category**	**Score**	**Assessment Category**	**Score**
Comprehension Score Rater 1	4	Gunning Fog Score	16.1	Words	135
Comprehension Score Rater 2	4	SMOG Index	11.8	Complex words	25
Reading Age (years)	20.5	Coleman Liau Index	16.7	Percentage of complex words	18.52
Flesch-Kincaid Reading Ease	34.2	Automated Readability Index	15.8	Average words per sentence	22.5
Flesch-Kincaid Grade Level	14.1	Sentences	6	Average syllables per word	1.77

<u>Lay Summary (Extended Prompt)</u>

This study looks at who gets access to proton therapy, a special type of radiation treatment for prostate cancer, and whether some groups of people face unfair barriers. Researchers reviewed 22 studies from the United States to find patterns in how this therapy is used. They discovered that factors like race, age, income, education, and insurance coverage can affect whether someone receives proton therapy. For example, non-White patients and those with lower incomes or less education are less likely to get this treatment compared to White patients or those from wealthier backgrounds. These differences, called health disparities, show that not everyone has the same chance to receive the best care. Understanding these gaps can help doctors, healthcare leaders, and policymakers find ways to make proton therapy available to more people, regardless of their background, as its use continues to grow in the U.S. and around the world.

**Assessment Category**	**Score**	**Assessment Category**	**Score**	**Assessment Category**	**Score**
Comprehension Score Rater 1	5	Gunning Fog Score	12.8	Words	149
Comprehension Score Rater 2	5	SMOG Index	9.4	Complex words	21
Reading Age (years)	17.5	Coleman Liau Index	14.6	Percentage of complex words	14.09
Flesch-Kincaid Reading Ease	53.4	Automated Readability Index	12.2	Average words per sentence	18.63
Flesch-Kincaid Grade Level	10.4	Sentences	8	Average syllables per word	1.59

Manuscript 28

Pascual G, Sabater A, Bizzotto J, Seniuk R, Sanchis P, Ledesma-Bazan S, et al. AR (CAG)n Microsatellite and APEX1 c.444T>G (p.Asp148Glu) Polymorphisms as Independent Prognostic Biomarkers in Prostate Cancer: Insights from an Argentinian Cohort. Cancers [Internet]. 2024 Nov 13;16(22):3815. Available from: http://dx.doi.org/10.3390/cancers16223815

Abstract

Background/objectives: Prostate cancer (PCa) is the leading malignancy and the third most common cause of cancer-related death in Argentinian men. Predicting outcomes in localized PCa remains difficult due to tumor heterogeneity. In this study, we assessed the impact of AR (CAG)n and APEX1 c.444T>G polymorphisms on biochemical relapse in Argentine patients with localized PCa.

Methods: We genotyped blood samples from 123 PCa patients for AR (CAG)n and APEX1 p.Asp148Glu (c.444T>G) polymorphisms. Associations with clinicopathological parameters and biochemical relapse-free survival (BRFS) were assessed.

Results: AR (CAG)20-23 was associated with a family history of breast/ovarian cancer (p = 0.0469). The combination of AR (CAG)20-23 and APEX1 c.444TT/GG correlated with a 2.89 times higher risk of biochemical relapse (log-rank p = 0.006). Multivariable analysis confirmed AR and APEX1 polymorphisms as independent predictors of biochemical relapse (HR = 3.95, p = 0.002). In patients with PSA levels <10 ng/mL, combined AR (CAG)20-23 and APEX1 c.444TT/GG genotypes were significantly associated with an increased risk of biochemical relapse (HR = 2.61, p = 0.044). Multivariable analysis confirmed the prognostic significance of these genotypes (HR = 3.44, p = 0.02).

Conclusions: This study has identified AR (CAG)n and APEX1 c.444T>G polymorphisms as independent predictors of PCa relapse in Argentinian patients, suggesting their potential use in improving prognostic models.

Keywords:

APEX1, androgen receptor, biochemical relapse, genetic biomarkers, polymorphisms, prostate cancer

<u>Original Lay Summary</u>

This study explores the association of two gene polymorphisms (AR (CAG)n and APEX1 c.444T>G (p.Asp148Glu)) with biochemical recurrence in Argentinian patients with prostate cancer. The findings suggest that patients with certain alleles (AR (CAG)M and the heterozygous APEX1 c.444TG genotype) are linked to an increased risk of prostate cancer relapse, even when accounting for conventional clinicopathological variables. Notably, patients with a combined AR (CAG)M and APEX1 c.444TT/GG genotype had the highest risk of relapse, indicating the potential of these polymorphisms as independent predictors. These results are particularly relevant for patients with prostate specific antigen (PSA) serum levels <10 ng/mL, where traditional markers may not predict outcomes. By incorporating these genetic markers into clinical models, this research offers a novel approach to enhance the precision of prostate cancer prognosis and improve patient stratification, ultimately guiding more personalized treatment strategies for high-risk individuals.

**Assessment Category**	**Score**	**Assessment Category**	**Score**	**Assessment Category**	**Score**
Comprehension Score Rater 1	5	Gunning Fog Score	14.0	Words	104
Comprehension Score Rater 2	5	SMOG Index	10.1	Complex words	24
Reading Age (years)	18.5	Coleman Liau Index	16.4	Percentage of complex words	23.08
Flesch-Kincaid Reading Ease	32.6	Automated Readability Index	10.8	Average words per sentence	13.0
Flesch-Kincaid Grade Level	11.9	Sentences	8	Average syllables per word	1.9

Lay Summary (Simple Prompt)

Prostate cancer is the most common cancer in Argentinian men and a leading cause of cancer-related deaths. Predicting which patients will experience a cancer relapse after treatment is challenging because the disease can behave differently in each person. This study investigated two specific genetic variations—one in the androgen receptor (AR) gene and another in the APEX1 gene—to see if they could help predict the risk of cancer returning. By analyzing blood samples from 123 men with localized prostate cancer, the researchers found that certain combinations of these genetic variations were linked to a higher chance of cancer relapse, even in patients with low PSA levels. These findings suggest that testing for these genetic markers could help doctors identify patients at greater risk of relapse, leading to more personalized follow-up care and treatment decisions.

**Assessment Category**	**Score**	**Assessment Category**	**Score**	**Assessment Category**	**Score**
Comprehension Score Rater 1	5	Gunning Fog Score	18.8	Words	135
Comprehension Score Rater 2	5	SMOG Index	13.6	Complex words	28
Reading Age (years)	21.5	Coleman Liau Index	15.9	Percentage of complex words	20.74
Flesch-Kincaid Reading Ease	29.0	Automated Readability Index	17.4	Average words per sentence	27.0
Flesch-Kincaid Grade Level	15.9	Sentences	5	Average syllables per word	1.78

Lay Summary (Extended Prompt)

Prostate cancer is the most common cancer in men in Argentina and a major cause of cancer-related deaths. Predicting which patients will experience a return of cancer after treatment is challenging. This study looked at specific changes in two genes, called AR and APEX1, in the blood of 123 men with prostate cancer. The researchers found that certain versions of these genes were linked to a higher chance of the cancer coming back, even in men with low PSA levels, which are usually considered less risky. These gene changes were strong predictors of cancer relapse, independent of other risk factors. Understanding these genetic markers may help doctors identify patients at higher risk of relapse earlier, allowing for more personalized care and better treatment planning. This could improve outcomes for men with prostate cancer in the future.

**Assessment Category**	**Score**	**Assessment Category**	**Score**	**Assessment Category**	**Score**
Comprehension Score Rater 1	4	Gunning Fog Score	13.1	Words	137
Comprehension Score Rater 2	4	SMOG Index	9.6	Complex words	19
Reading Age (years)	17.5	Coleman Liau Index	14.1	Percentage of complex words	13.87
Flesch-Kincaid Reading Ease	52.4	Automated Readability Index	12.3	Average words per sentence	19.57
Flesch-Kincaid Grade Level	10.8	Sentences	7	Average syllables per word	1.59

Manuscript 29

Bertok T, Jane E, Hires M, Tkac J. N-Acetylated Monosaccharides and Derived Glycan Structures Occurring in N- and O-Glycans During Prostate Cancer Development. Cancers [Internet]. 2024 Nov 10;16(22):3786. Available from: http://dx.doi.org/10.3390/cancers16223786

Abstract

Post-translational modifications of proteins play an important role in their stability, solubility and in vivo function. Also, for several reasons, such as the Golgi fragmentation during cancerogenesis, glycosylation as the most common modification is especially promising in offering high cancer specificity which, in combination with tissue-specific biomarkers available in the case of prostate diseases (PSA, PSMA, PAP), may lead to the development of novel oncodiagnostic approaches. In this review, we present the importance of subterminal glycan structures based on the N-acetylated monosaccharides GlcNAc and GalNAc in N- and also O-glycans, structures of which they are a component (LacNAc, LacdiNAc, branched structures). We also discuss the importance and clinical performance of these structures in cases of prostate cancer diagnostics using lectin-based affinity methods, which could be implemented in clinical laboratory practice in the future.

Keywords:

LacdiNAc, PSA, glycan, glycosylation, lectin, prostate cancer

<u>Original Lay Summary</u>

Prostate cancer is globally one of the commonest cancer types with hundreds of thousands of deaths annually. Early diagnostics and the elimination of overtreatment lead to higher survival rates and increase the overall quality of life. Novel diagnostic markers are continually being introduced, but glycans, as one of the most promising cancer-related biomarkers, are excluded due to technological requirements, despite the fact that several approaches compatible with clinical practice have emerged in recent years.

**Assessment Category**	**Score**	**Assessment Category**	**Score**	**Assessment Category**	**Score**
Comprehension Score Rater 1	4	Gunning Fog Score	20.7	Words	75
Comprehension Score Rater 2	4	SMOG Index	14.9	Complex words	20
Reading Age (years)	23.5	Coleman Liau Index	18.4	Percentage of complex words	26.67
Flesch-Kincaid Reading Ease	17.9	Automated Readability Index	18.4	Average words per sentence	25.0
Flesch-Kincaid Grade Level	17.0	Sentences	3	Average syllables per word	1.93

<u>Lay Summary (Simple Prompt)</u>

Prostate cancer is one of the most common cancers in men, and finding better ways to detect it early can save lives. This study focuses on special sugar molecules, called glycans, which attach to proteins in the body and can change when cancer develops. We specifically looked at certain glycans containing N-acetylated sugars, which may play an important role in prostate cancer. By understanding how these sugars change during cancer development, we can improve diagnostic tests. The study also explores how these sugar structures can be detected using a method called lectin-based affinity, which could help identify prostate cancer more accurately when combined with current markers like PSA. This research could lead to more reliable, early detection methods for prostate cancer, ultimately helping doctors diagnose and treat patients more effectively.

**Assessment Category**	**Score**	**Assessment Category**	**Score**	**Assessment Category**	**Score**
Comprehension Score Rater 1	4	Gunning Fog Score	14.3	Words	132
Comprehension Score Rater 2	4	SMOG Index	10.1	Complex words	18
Reading Age (years)	19.5	Coleman Liau Index	16.0	Percentage of complex words	13.64
Flesch-Kincaid Reading Ease	39.0	Automated Readability Index	15.0	Average words per sentence	22.0
Flesch-Kincaid Grade Level	13.3	Sentences	6	Average syllables per word	1.72

<u>Lay Summary (Extended Prompt)</u>

Prostate cancer is one of the most common cancers in men, and finding better ways to detect it early can save lives. This study looks at special sugar molecules called glycans that attach to proteins in the body. When cancer develops, these sugars can change in unique ways, making them useful for spotting the disease. We focused on specific sugar building blocks, called N-acetylated monosaccharides, and the structures they form in prostate cancer. By studying how these sugars change, we aim to improve current tests like PSA, which sometimes give unclear results. Our research suggests that using special tools called lectins to detect these sugar changes could help doctors diagnose prostate cancer more accurately in the future. This approach might lead to better, more reliable tests, helping to catch prostate cancer earlier and guide treatment decisions.

**Assessment Category**	**Score**	**Assessment Category**	**Score**	**Assessment Category**	**Score**
Comprehension Score Rater 1	4	Gunning Fog Score	11.6	Words	137
Comprehension Score Rater 2	4	SMOG Index	8.0	Complex words	13
Reading Age (years)	17.5	Coleman Liau Index	14.8	Percentage of complex words	9.49
Flesch-Kincaid Reading Ease	53.0	Automated Readability Index	12.8	Average words per sentence	19.57
Flesch-Kincaid Grade Level	10.7	Sentences	7	Average syllables per word	1.58

Manuscript 30

Prunaretty J, Ungun B, Vauclin R, Costea M, Bus N, Paragios N, et al. Quantitative Evaluation of a Fully Automated Planning Solution for Prostate-Only and Whole-Pelvic Radiotherapy. Cancers [Internet]. 2024 Nov 5;16(22):3735. Available from: http://dx.doi.org/10.3390/cancers16223735

Abstract

Background/Objectives: To evaluate an end-to-end pipeline for normo-fractionated prostate-only and whole-pelvic cancer treatments that requires minimal human input and generates a machine-deliverable plan as an output. Methods: In collaboration with TheraPanacea, a treatment planning pipeline was developed that takes as its input a planning CT with organs-at-risk (OARs) and planning target volume (PTV) contours, the targeted linac machine, and the prescription dose. The primary components are (i) dose prediction by a single deep learning model for both localizations and (ii) a direct aperture VMAT plan optimization that seeks to mimic the predicted dose. The deep learning model was trained on 238 cases, and a held-out set of 86 cases was used for model validation. An end-to-end clinical evaluation study was performed on another 40 cases (20 prostate-only, 20 whole-pelvic). First, a quantitative evaluation was performed based on dose-volume histogram (DVH) points and plan parameter metrics. Then, the plan deliverability was assessed via portal dosimetry using the global gamma index. Additionally, the reference clinical manual plans were compared with the automated plans in terms of monitor unit (MU) numbers and modulation complexity scores (MCSv). Results: The automated plans provided adequate treatment plans (or minor deviations) with respect to the dose constraints, and the quality of the plans was similar to the manual plans for both localizations. Moreover, the automated plans showed successful deliverability and passed the portal dose verification. Despite higher median total MUs, no statistically significant correlation was observed between any of the gamma criteria tested and the number of MUs or MCSv. Conclusions: This study shows the feasibility of a deep learning-based fully automated treatment planning pipeline that generates high-

quality plans that are competitive with manually made plans and are clinically approved in terms of dosimetry and machine deliverability.

Keywords:

VMAT, artificial intelligence, auto-planning, deep learning, prostate cancer

Original Lay Summary

In recent years, advanced radiotherapy techniques such as intensity-modulated radiation therapy (IMRT) and simultaneous integrated boost (SIB) irradiation have played a key role in enhancing the precision of radiation delivery to tumors. However, these advancements have also increased the complexity of treatment planning by involving a trial-and-error approach, resulting in greater variability between operators and longer planning times. The automation of planning processes has shown promise in standardizing treatment plans while maintaining their quality and reducing workload. Additionally, deep learning-based fully automated planning solutions have become a significant focus of research in radiation oncology. In this study, we propose a single, end-to-end pipeline for normo-fractionated prostate-only and whole-pelvic cancer treatments, requiring minimal human input and producing a machine-deliverable volumetric modulated arc therapy (VMAT) plan. A comprehensive clinical evaluation was performed, incorporating both dosimetric analysis and plan deliverability assessment.

**Assessment Category**	**Score**	**Assessment Category**	**Score**	**Assessment Category**	**Score**
Comprehension Score Rater 1	5	Gunning Fog Score	23.1	Words	148
Comprehension Score Rater 2	5	SMOG Index	16.4	Complex words	49
Reading Age (years)	24.0	Coleman Liau Index	20.8	Percentage of complex words	33.11
Flesch-Kincaid Reading Ease	-4.0	Automated Readability Index	20.2	Average words per sentence	24.67
Flesch-Kincaid Grade Level	19.9	Sentences	6	Average syllables per word	2.2

Lay Summary (Simple Prompt)

This study explored a new fully automated system designed to create radiation treatment plans for prostate cancer patients, both targeting the prostate only and the entire pelvic area. The goal was to reduce the need for human input while still producing high-quality, safe, and effective treatment plans. The system uses advanced artificial intelligence, specifically deep learning, to predict the best radiation dose and then optimize the plan to match that prediction. Researchers tested the system on 40 patient cases and compared the automated plans to traditional, manually created ones. The results showed that the automated plans met clinical safety standards, matched the quality of manual plans, and could be delivered accurately by radiation machines. This approach could save time for healthcare professionals, reduce the chance of human error, and make radiation treatment planning more efficient, potentially improving care for prostate cancer patients.

**Assessment Category**	**Score**	**Assessment Category**	**Score**	**Assessment Category**	**Score**
Comprehension Score Rater 1	5	Gunning Fog Score	17.6	Words	143
Comprehension Score Rater 2	5	SMOG Index	12.7	Complex words	29
Reading Age (years)	21.5	Coleman Liau Index	17.4	Percentage of complex words	20.28
Flesch-Kincaid Reading Ease	32.4	Automated Readability Index	17.0	Average words per sentence	23.83
Flesch-Kincaid Grade Level	14.7	Sentences	6	Average syllables per word	1.78

Lay Summary (Extended Prompt)

This study looks at a new computer-based system designed to create radiation treatment plans for prostate cancer with little human involvement. Normally, planning these treatments takes a lot of time and expert work. Our new system uses artificial intelligence to automatically design treatment plans for both prostate-only and whole-pelvic radiotherapy. We tested this system on 40 patients and compared the results to traditional, manually made plans. The automated plans were just as effective and safe, meeting the required dose levels needed for successful treatment. They were also successfully tested on radiation machines to ensure they work in real clinical settings. This research shows that using artificial intelligence can save time, reduce the need for manual work, and still provide high-quality cancer treatment plans. In the future, this could make prostate cancer care faster and more accessible for patients.

**Assessment Category**	**Score**	**Assessment Category**	**Score**	**Assessment Category**	**Score**
Comprehension Score Rater 1	5	Gunning Fog Score	13.0	Words	142
Comprehension Score Rater 2	5	SMOG Index	9.4	Complex words	21
Reading Age (years)	18.5	Coleman Liau Index	16.1	Percentage of complex words	14.79
Flesch-Kincaid Reading Ease	43.4	Automated Readability Index	13.0	Average words per sentence	17.75
Flesch-Kincaid Grade Level	11.6	Sentences	8	Average syllables per word	1.72

Manuscript 31

Pakula H, Pederzoli F, Fanelli GN, Nuzzo PV, Rodrigues S, Loda M. Deciphering the Tumor Microenvironment in Prostate Cancer: A Focus on the Stromal Component. Cancers [Internet]. 2024 Oct 31;16(21):3685. Available from: http://dx.doi.org/10.3390/cancers16213685

Abstract

Prostate cancer progression is significantly affected by its tumor microenvironment, in which mesenchymal cells play a crucial role. Stromal cells are modified by cancer mutations, response to androgens, and lineage plasticity, and in turn, engage with epithelial tumor cells via a complex array of signaling pathways and ligand-receptor interactions, ultimately affecting tumor growth, immune interaction, and response to therapy. The metabolic rewiring and interplay in the microenvironment play an additional role in affecting the growth and progression of prostate cancer. Finally, therapeutic strategies and novel clinical trials with agents that target the stromal microenvironment or disrupt the interaction between cellular compartments are described. This review underscores cancer-associated fibroblasts as essential contributors to prostate cancer biology, emphasizing their potential as prognostic indicators and therapeutic targets.

Keywords:

cancer-associated fibroblasts (CAFs), castration-resistant prostate cancer (CRPC), mesenchymal cells, prostate cancer (PCa), tumor microenvironment (TME)

<u>Original Lay Summary</u>

Prostate cancer is one of the most common cancers in men, and its progression is strongly influenced by the surrounding environment, known as the tumor microenvironment. This is comprised of a variety of cells, including cancer-associated fibroblasts (CAFs), that modulate tumor initiation and progression and may affect trea ment strategies. This review focuses on the interactions between fibroblasts and eithelial cancer cells and how these interactions contribute to cancer progression. This knowledge may unveil novel therapeutic approaches that could mitigate

aggressiveness and/or render prostate cancer more responsive to current treaments, thus improving patient outcomes.

**Assessment Category**	**Score**	**Assessment Category**	**Score**	**Assessment Category**	**Score**
Comprehension Score Rater 1	5	Gunning Fog Score	19.2	Words	94
Comprehension Score Rater 2	5	SMOG Index	13.8	Complex words	23
Reading Age (years)	23.5	Coleman Liau Index	20.4	Percentage of complex words	24.47
Flesch-Kincaid Reading Ease	19.2	Automated Readability Index	19.3	Average words per sentence	23.5
Flesch-Kincaid Grade Level	16.4	Sentences	4	Average syllables per word	1.94

<u>Lay Summary (Simple Prompt)</u>

Prostate cancer is influenced not only by genetic mutations but also by its surrounding environment, which includes various non-cancerous cells. Among these, stromal cells play a key role in helping or hindering tumor growth. These cells interact with cancer cells through complex signals, affecting how the disease progresses and responds to treatment. This study explores how changes in these supportive cells impact prostate cancer and how they could be targeted for therapy. Understanding these interactions may lead to better treatment strategies and improved patient outcomes. Scientists are now investigating new therapies designed to disrupt these interactions, potentially slowing cancer growth and making treatments more effective. By focusing on the role of the tumor's environment, this research may help identify new ways to predict how prostate cancer will behave and respond to treatment, ultimately guiding the development of more personalized and effective therapies.

**Assessment Category**	**Score**	**Assessment Category**	**Score**	**Assessment Category**	**Score**
Comprehension Score Rater 1	5	Gunning Fog Score	16.3	Words	143
Comprehension Score Rater 2	5	SMOG Index	11.8	Complex words	29
Reading Age (years)	20.5	Coleman Liau Index	18.3	Percentage of complex words	20.28
Flesch-Kincaid Reading Ease	35.8	Automated Readability Index	16.1	Average words per sentence	20.43
Flesch-Kincaid Grade Level	13.3	Sentences	7	Average syllables per word	1.78

<u>Lay Summary (Extended Prompt)</u>

Prostate cancer is not just about the tumor itself—it also depends on the environment around it, called the tumor microenvironment. This includes special support cells, known as stromal cells, which can influence how the cancer grows, spreads, and responds to treatment. These stromal cells interact with cancer cells through signals and metabolic changes, sometimes making the cancer more aggressive or resistant to therapy. Scientists are now studying ways to block these interactions to slow cancer growth and improve treatment outcomes. This review explains how these support cells contribute to prostate cancer, why they could serve as important markers for predicting cancer progression, and how new drugs are being developed to target these interactions. Understanding the role of these cells could lead to better treatments and improved survival for patients with prostate cancer.

**Assessment Category**	**Score**	**Assessment Category**	**Score**	**Assessment Category**	**Score**
Comprehension Score Rater 1	5	Gunning Fog Score	14.6	Words	132
Comprehension Score Rater 2	5	SMOG Index	10.3	Complex words	19
Reading Age (years)	19.5	Coleman Liau Index	17.3	Percentage of complex words	14.39
Flesch-Kincaid Reading Ease	45.4	Automated Readability Index	16.0	Average words per sentence	22.0
Flesch-Kincaid Grade Level	12.4	Sentences	6	Average syllables per word	1.64

Manuscript 32

Jimenez Rios MA, Scavuzzo A, Noverón NR, García Arango C, Calvo Vazquez I, Hurtado Vázquez A, et al. Lethal Prostate Cancer in Mexico: Data from the Can.Prost Mexican Registry and a Project for Early Detection. Cancers [Internet]. 2024 Oct 30;16(21):3675. Available from: http://dx.doi.org/10.3390/cancers16213675

Abstract

Introduction: Epidemiological data are crucial for adopting primary and secondary prevention strategies and to develop screening protocols against prostate cancer (PCa). Despite the comprehensive characterization of PCa across White and Black men, there is a lack of data from the Mexican population. This manuscript presents data from the Can.Prost registry that captures PCa trends over the past two decades in Mexico City; furthermore, we aimed to compare clinical differences and oncological outcomes before and after the promotion of early detection actions through a campaign against PCa that occurred in 2014.

Methods: A retrospective observational study on newly diagnosed Mexican PCa patients was carried out at the Instituto Nacional de Cancerología (INCan) in Mexico City. During 2014 and 2015, a project for the early diagnosis of PCa ("OPUS program") was launched in the aforementioned tertiary hospital. Starting at the age of 45 years, all men were invited for a PSA measurement and a specialist urologist consultation. All individuals with clinical or biochemical suspicion of PCa (PSA > 4 ng/mL), in the context of age and prostate volume, underwent ultrasound-guided transrectal prostate biopsy. Then, patients with pathologically confirmed prostate cancer were stratified according to the year of diagnosis: Group A accounted for those diagnosed between 2000 and 2014 and Group B for those patients diagnosed in the timeframe of 2015-2021. Comparisons of PCa characteristics, treatment modalities and oncologic outcomes between Group A and B were performed.

Results: Overall, we collected data from 2759 PCa patients from 2000 to 2021. The median PSA at baseline was 32 ng/mL, and 25% had a family history of PCa. Overall, 25.8% were asymptomatic and 46% had a non-metastatic presentation. After the OPUS campaign, PSA at diagnosis was significantly lower across all age groups. The incidence of PCa diagnosis in asymptomatic men was higher (31.4% vs. 19.9%) and a higher proportion of men were diagnosed with organ-confined, palpable disease (46% vs. 28%) ($p < 0.001$). The rate of patients eligible for active/radical treatment was higher after the OPUS campaign (patients who received surgery increased from 12.78% to 32.41%; patients who underwent radiation increased from 28.38% to 49.61%). The proportion of patients diagnosed with non-clinically significant disease was negligible and remained stable across time.

Conclusions: PCa in Mexican patients displays aggressive features at diagnosis, whereas the rate of non-significant disease is negligible. The introduction of early detection strategies may lead to lower symptomatic and metastatic PCa and higher opportunities for radical treatment. This emphasizes the need for public awareness and for adjustment of screening strategies to the peculiarities of the Mexican population.

Keywords:

aggressive features, early diagnosis, lethal cancer, prostate cancer

<u>Original Lay Summary</u>

Prostate cancer (PCa) is a heterogeneous disease and the second most diagnosticated cancer in men. Epidemiological information is essential to adopt the strategy of early detection. In Mexico there is paucity of epidemiological data. We observed that Mexican men present aggressive disease at diagnosis with metastatic symptoms. Seems the Mexican race is a risk factor for PCa, as African men. “OPUS program “ is the actual screening protocol for Mexican men. We describe the concernment of early detection of PCa in the Mexican population to reduce the rate of metastases.

**Assessment Category**	**Score**	**Assessment Category**	**Score**	**Assessment Category**	**Score**
Comprehension Score Rater 1	5	Gunning Fog Score	13.1	Words	90
Comprehension Score Rater 2	5	SMOG Index	10.7	Complex words	24
Reading Age (years)	17.5	Coleman Liau Index	15.1	Percentage of complex words	26.67
Flesch-Kincaid Reading Ease	29.3	Automated Readability Index	9.7	Average words per sentence	12.86
Flesch-Kincaid Grade Level	12.4	Sentences	7	Average syllables per word	1.94

<u>Lay Summary (Simple Prompt)</u>

Prostate cancer is a major health concern worldwide, but little is known about its characteristics in Mexican men. This study analyzed data from over 2,700 patients diagnosed with prostate cancer in Mexico City between 2000 and 2021 to understand how the disease presents and whether early detection efforts improve outcomes. A screening program launched in 2014 encouraged men aged 45 and older to get tested. After this campaign, more men were diagnosed at an earlier stage, when the cancer was still confined to the prostate, and had better chances of receiving curative treatment. The findings suggest that prostate cancer in Mexican men is often aggressive at diagnosis, highlighting the need for increased awareness and targeted screening strategies. Early detection programs may help reduce advanced cases and improve survival, emphasizing the importance of proactive healthcare measures tailored to the Mexican population.

**Assessment Category**	**Score**	**Assessment Category**	**Score**	**Assessment Category**	**Score**
Comprehension Score Rater 1	5	Gunning Fog Score	15.1	Words	141
Comprehension Score Rater 2	5	SMOG Index	11.6	Complex words	24
Reading Age (years)	19.5	Coleman Liau Index	15.9	Percentage of complex words	17.02
Flesch-Kincaid Reading Ease	37.2	Automated Readability Index	15.7	Average words per sentence	23.5
Flesch-Kincaid Grade Level	13.9	Sentences	6	Average syllables per word	1.72

<u>Lay Summary (Extended Prompt)</u>

Prostate cancer is a serious health issue worldwide, but there is little data on how it affects men in Mexico. This study analyzed information from nearly 2,800 Mexican men diagnosed with prostate cancer over two decades. Researchers compared cases before and after a campaign in 2014 that encouraged early detection through PSA testing and specialist consultations. They found that after this campaign, more men were diagnosed earlier, when the cancer was less advanced and easier to treat. This led to more men receiving surgery or radiation, which can improve survival. However, prostate cancer in Mexico still tends to be aggressive when diagnosed, highlighting the need for better awareness and screening strategies tailored to Mexican men. These findings suggest that early detection programs can make a difference and should be expanded to help reduce deaths from prostate cancer in Mexico.

**Assessment Category**	**Score**	**Assessment Category**	**Score**	**Assessment Category**	**Score**
Comprehension Score Rater 1	5	Gunning Fog Score	14.6	Words	140
Comprehension Score Rater 2	5	SMOG Index	11.6	Complex words	28
Reading Age (years)	18.5	Coleman Liau Index	15.2	Percentage of complex words	20.0
Flesch-Kincaid Reading Ease	43.9	Automated Readability Index	13.4	Average words per sentence	20.0
Flesch-Kincaid Grade Level	12.1	Sentences	7	Average syllables per word	1.69

Manuscript 33

Torres-Luna C, Wei S, Bhattiprolu S, Tuszynski G, Rothman VL, McNulty D, et al. G-Protein-Coupled Receptor-Associated Sorting Protein 1 Overexpression Is Involved in the Progression of Benign Prostatic Hyperplasia, Early-Stage Prostatic Malignant Diseases, and Prostate Cancer. Cancers [Internet]. 2024 Oct 30;16(21):3659. Available from: http://dx.doi.org/10.3390/cancers16213659

Abstract

Background/Objectives: Prostate cancer (PCa) is a prevalent malignancy, necessitating accurate diagnostic methods to distinguish it from benign conditions such as benign prostatic hyperplasia (BPH). Current diagnostic tools, relying primarily on serum prostate-specific antigen (PSA) levels, lack specificity, leading to an over-diagnosis and unnecessary treatment of patients with benign conditions. This study explores G-protein-coupled receptor-associated sorting protein 1 (GASP-1) as a more sensitive biomarker for PCa detection. Methods: Prostate tissue microarrays of healthy, BPH, and prostate cancer patients with different Gleason scores were studied. Polyclonal antibodies targeted against GASP-1 were used for routine immunohistochemistry (IHC) and enzyme-linked immunosorbent assay (ELISA) analyses. Results: The results indicated a 5-fold difference in serum GASP-1 levels between BPH and PCa, which was validated through GASP-1 IHC. Furthermore, a novel scoring system, the H-score, assesses GASP-1 granules' intensity and size, revealing a clear distinction between BPH and PCa. An additional analysis of GASP-1 expression between PCa cases with different Gleason scores reveals that GASP-1 overexpression correlates with PCa severity, providing insights into disease progression. Conclusions: The study supports GASP-1's role as a promising diagnostic marker, supplementing PSA testing, and offering improved risk stratification for PCa. Additionally, an open-source software system is introduced for an efficient GASP-1 granule color analysis, enhancing diagnostic accuracy.

Keywords:

2D HPLE, ELISA, GASP-1, benign prostatic hyperplasia, cancer diagnostic, cancer prevention, immunohistochemistry, prostate cancer biomarker

<u>Original Lay Summary</u>

Prostate cancer is the second most common cancer diagnosed in men and yet is the second leading cause of cancer-related deaths worldwide. This is often due to the cancer being misdiagnosed as benign prostatic hyperplasia or missed entirely due to the limited nature of the most popular detection method, prostate-specific antigen levels. The aim of this study was to assess the potential of G-protein-coupled receptor-associated sorting protein 1 (GASP-1) as a valid prostate cancer biomarker. Prostate tissue samples from healthy, benign prostatic hyperplasia, and prostate cancer patients were subjected to anti-GASP-1 polyclonal antibodies in immunohistochemical staining and enzyme-linked immunosorbent assay analyses, showing that GASP-1 levels were significantly greater in prostate cancer patients when compared to benign prostatic hyperplasia and healthy patients. Specifically in prostate cancer patients, there was a positive correlation between GASP-1 overexpression and the severity of the prostate cancer.

**Assessment Category**	**Score**	**Assessment Category**	**Score**	**Assessment Category**	**Score**
Comprehension Score Rater 1	5	Gunning Fog Score	20.3	Words	132
Comprehension Score Rater 2	5	SMOG Index	14.3	Complex words	31
Reading Age (years)	23.5	Coleman Liau Index	17.1	Percentage of complex words	20.39
Flesch-Kincaid Reading Ease	14.6	Automated Readability Index	20.1	Average words per sentence	30.4
Flesch-Kincaid Grade Level	18.8	Sentences	5	Average syllables per word	1.91

Lay Summary (Simple Prompt)

Prostate cancer is one of the most common cancers in men, but diagnosing it accurately remains challenging. Many current tests, such as measuring prostate-specific antigen (PSA) levels in the blood, lack precision and can lead to unnecessary treatments for non-cancerous conditions like benign prostatic hyperplasia (BPH). This study investigates a new potential biomarker called GASP-1, which may help distinguish between BPH and prostate cancer more effectively. By analyzing prostate tissue and blood samples, researchers found that GASP-1 levels were significantly higher in cancer patients and increased with disease severity. They also developed a new scoring system to assess GASP-1 levels more accurately. These findings suggest that GASP-1 could be a valuable tool to improve prostate cancer diagnosis, reducing the risk of unnecessary treatments and helping doctors make better-informed decisions. Additionally, the study introduces software to enhance the accuracy of GASP-1 analysis, making future testing more reliable.

**Assessment Category**	**Score**	**Assessment Category**	**Score**	**Assessment Category**	**Score**
Comprehension Score Rater 1	5	Gunning Fog Score	17.1	Words	154
Comprehension Score Rater 2	5	SMOG Index	12.4	Complex words	32
Reading Age (years)	20.1	Coleman Liau Index	16.9	Percentage of complex words	20.78
Flesch-Kincaid Reading Ease	27.4	Automated Readability Index	15.7	Average words per sentence	22.0
Flesch-Kincaid Grade Level	14.9	Sentences	7	Average syllables per word	1.86

Lay Summary (Extended Prompt)

Prostate cancer is one of the most common cancers in men, but diagnosing it early can be challenging. Many tests rely on measuring a protein called PSA in the blood, but this method is not always accurate and can lead to unnecessary treatments. This study explores a new potential biomarker, GASP-1, to improve prostate cancer detection. Researchers examined prostate tissue and blood samples from healthy individuals, patients with benign prostatic hyperplasia (a non-cancerous prostate condition), and prostate cancer patients. The findings show that GASP-1 levels are much higher in prostate cancer cases than in benign conditions, making it a promising diagnostic tool. Additionally, a new scoring system was developed to measure GASP-1 more precisely, which may help doctors assess disease severity. These findings could lead to better, more reliable tests for prostate cancer, reducing unnecessary treatments and improving patient care.

**Assessment Category**	**Score**	**Assessment Category**	**Score**	**Assessment Category**	**Score**
Comprehension Score Rater 1	5	Gunning Fog Score	15.5	Words	144
Comprehension Score Rater 2	5	SMOG Index	11.2	Complex words	26
Reading Age (years)	19.5	Coleman Liau Index	15.9	Percentage of complex words	18.06
Flesch-Kincaid Reading Ease	36.1	Automated Readability Index	14.2	Average words per sentence	20.57
Flesch-Kincaid Grade Level	13.3	Sentences	7	Average syllables per word	1.77

Manuscript 34

Siech C, Wenzel M, Grosshans N, Cano Garcia C, Humke C, Koll FJ, et al. The Association Between Lymphovascular or Perineural Invasion in Radical Prostatectomy Specimen and Biochemical Recurrence. Cancers [Internet]. 2024 Oct 29;16(21):3648. Available from: http://dx.doi.org/10.3390/cancers16213648

Abstract

Objective: The aim of this study was to test for the association between lymphovascular invasion or perineural invasion in radical prostatectomy (RP) specimens and biochemical recurrence (BCR).

Methods: Relying on a tertiary-care database, we identified prostate cancer patients treated with RP between January 2014 and June 2023. Of these, the majority underwent robotic-assisted RP (81%). Kaplan-Meier survival analyses and Cox regression models addressed BCR according to either lymphovascular invasion or perineural invasion in RP specimens. Additionally, the linear trend test assessed the association between the Gleason Grade Group or pathologic tumor stage and lymphovascular or perineural invasion.

Results: Of 822 patients, 78 (9%) exhibited lymphovascular invasion and 633 (77%) exhibited perineural invasion in RP specimens. In survival analyses, the five-year BCR-free survival rates were 62% in patients with lymphovascular invasion vs. 70% in patients without lymphovascular invasion (p = 0.04) and 64% in patients with perineural invasion vs. 82% in patients without perineural invasion (p = 0.01). In univariable Cox regression models, lymphovascular invasion (hazard ratio 1.58, 95% confidence interval 1.01-2.47; p = 0.045) and perineural invasion (hazard ratio 1.77, 95% confidence interval 1.13-2.77; p = 0.013) were both associated with a higher BCR rate. After accounting for age at surgery, PSA value, pathologic tumor stage, Gleason Grade Group, lymph node invasion, positive surgical margin, surgical approach, and adjuvant radiation therapy, lymphovascular (p = 0.740) or perineural invasion (p =

0.341) were not significantly associated with a higher BCR since the Gleason Grade Group and pathologic tumor stage highly correlated with lymphovascular as well as perineural invasion.

Conclusions: In univariable models, lymphovascular or perineural invasion is associated with BCR. After adjustment for standard pathologic tumor characteristics, lymphovascular or perineural invasion is not an independent predictor for BCR.

Keywords:

BCR, lymphovascular invasion, perineural invasion, prostate cancer, radical prostatectomy

<u>Original Lay Summary</u>

The prognostic value of lymphovascular or perineural invasion in prostate cancer specimens regarding oncological outcomes after radical prostatectomy is unclear. Within a contemporary study cohort of 822 prostate cancer patients, 78 (9%) exhibited lymphovascular invasion and 633 (77%) exhibited perineural invasion in RP specimens. In univariable Cox regression models, lymphovascular invasion and perineural invasion were both associated with higher rates of biochemical recurrence (BCR). However, after multivariable adjustment for standard pathologic tumor characteristics, lymphovascular or perineural invasion was not found to be an independent predictor for BCR. These phenomes may be explained by the strong association between the Gleason Grade Group and pathologic tumor stage with lymphovascular as well as perineural invasion.

**Assessment Category**	**Score**	**Assessment Category**	**Score**	**Assessment Category**	**Score**
Comprehension Score Rater 1	5	Gunning Fog Score	23.6	Words	112
Comprehension Score Rater 2	4	SMOG Index	16.5	Complex words	41
Reading Age (years)	24.0	Coleman Liau Index	20.9	Percentage of complex words	36.61
Flesch-Kincaid Reading Ease	-9.3	Automated Readability Index	19.2	Average words per sentence	22.4
Flesch-Kincaid Grade Level	20.1	Sentences	5	Average syllables per word	2.29

Lay Summary (Simple Prompt)

When prostate cancer is surgically removed, doctors examine the tissue to check for signs that the cancer might return. Two such signs are lymphovascular invasion (cancer cells spreading into blood vessels or lymph nodes) and perineural invasion (cancer spreading along nerves). This study analyzed data from 822 patients who had undergone prostate cancer surgery to determine whether these two factors are linked to a higher risk of cancer recurrence, measured by rising PSA levels. Initially, both lymphovascular and perineural invasion were associated with a greater chance of recurrence. However, after adjusting for other cancer severity indicators, these factors did not independently predict recurrence risk. This suggests that while these findings may indicate aggressive cancer, they do not provide additional predictive value beyond other well-established tumor characteristics. Understanding these relationships helps refine how doctors assess cancer prognosis and decide on post-surgery treatments for patients.

**Assessment Category**	**Score**	**Assessment Category**	**Score**	**Assessment Category**	**Score**
Comprehension Score Rater 1	5	Gunning Fog Score	17.4	Words	145
Comprehension Score Rater 2	5	SMOG Index	12.5	Complex words	33
Reading Age (years)	21.5	Coleman Liau Index	19.1	Percentage of complex words	22.76
Flesch-Kincaid Reading Ease	24.2	Automated Readability Index	16.8	Average words per sentence	20.71
Flesch-Kincaid Grade Level	15.0	Sentences	7	Average syllables per word	1.91

Lay Summary (Extended Prompt)

Prostate cancer is one of the most common cancers in men, and doctors use several factors to predict whether the disease might return after surgery. This study looked at two specific features in prostate cancer tissue—lymphovascular invasion (when cancer spreads into blood or lymph vessels) and perineural invasion (when cancer spreads around nerves)—to see if they are linked to a higher risk of cancer returning after surgery. Researchers analyzed data from 822 patients who had prostate surgery, using statistical methods to compare their outcomes. The results showed that both types of invasion were associated with a higher risk of cancer recurrence at first. However, when other important cancer characteristics were considered, these factors were not independent predictors. This means that while they may indicate aggressive cancer, they do not provide unique predictive value beyond standard tumor grading. These findings help refine how doctors assess prostate cancer risk.

**Assessment Category**	**Score**	**Assessment Category**	**Score**	**Assessment Category**	**Score**
Comprehension Score Rater 1	5	Gunning Fog Score	15.5	Words	148
Comprehension Score Rater 2	5	SMOG Index	11.2	Complex words	26
Reading Age (years)	19.5	Coleman Liau Index	16.7	Percentage of complex words	17.57
Flesch-Kincaid Reading Ease	36.8	Automated Readability Index	15.1	Average words per sentence	21.14
Flesch-Kincaid Grade Level	13.4	Sentences	7	Average syllables per word	1.76

Manuscript 35

Van der Eecken H, De Cock D, Roussel E, Giesen A, Vansevenant B, Goeman L, et al. Nutritional Supplement with Fermented Soy in Patients Under Active Surveillance for Low-Risk or Intermediate-Risk Prostate Cancer: Results from the PRAEMUNE Trial. Cancers [Internet]. 2024 Oct 28;16(21):3634. Available from: http://dx.doi.org/10.3390/cancers16213634

Abstract

Background/Objectives: To investigate the effect of a fermented soy supplement during 18 months in patients under active surveillance (AS) for low-risk and selected favorable intermediate-risk prostate cancer (PCa), with an emphasis on PSA modulation. Methods: Low-risk patients with ISUP grade 1, clinical stage cT1 or cT2a, PSA Results: Overall, 92 (61.3%) of 150 patients showed a PSA level response. This was more evident in ISUP 1 patients and resulted in fewer follow-up MRIs and fewer control biopsies, as well as a fewer number of positive control biopsies with statistical significance in the imputed dataset. Obtaining a PSA response was numerically associated with less initiated therapy. Conclusions: a fermented soy supplement in patients under AS for low-risk and selected favorable intermediate-risk PCa could be useful in selecting patients who may remain under AS or who may need to switch to active therapy.

Keywords:

PSA, active surveillance, fermented soy, prostate cancer

Original Lay Summary

Many beneficial mechanisms of action are known from soy isoflavones, including in prostate and prostate cancer. Previous research was able to demonstrate a clear effect of PSA modulation, and this could potentially lead to better selection of patients at increased risk of occult prostate carcinoma. In low-grade localized prostate cancer, more and more active surveillance is being proposed and a lot of research is being carried out to see if certain factors or treatments may also act in this regard. This formed the basis to study the effect of soy isoflavones in patients under active surveillance. In this study, we were able to observe PSA modulation in a large proportion of patients, resulting in fewer follow-up examinations and fewer positive control biopsies. Potentially, in active surveillance, a soy supplement could be useful to help select patients who could continue to be followed up or who should be treated earlier.

**Assessment Category**	**Score**	**Assessment Category**	**Score**	**Assessment Category**	**Score**
Comprehension Score Rater 1	5	Gunning Fog Score	16.7	Words	151
Comprehension Score Rater 2	5	SMOG Index	11.8	Complex words	25
Reading Age (years)	19.5	Coleman Liau Index	14.3	Percentage of complex words	16.56
Flesch-Kincaid Reading Ease	37.9	Automated Readability Index	15.3	Average words per sentence	25.17
Flesch-Kincaid Grade Level	14.2	Sentences	6	Average syllables per word	1.7

<u>Lay Summary (Simple Prompt)</u>

Prostate cancer is often slow-growing, and some patients with low-risk or favorable intermediate-risk disease can delay treatment while carefully monitoring their condition, a strategy called active surveillance. This study explored whether a nutritional supplement containing fermented soy could help these patients by stabilizing their prostate-specific antigen (PSA) levels, a key marker for cancer progression. Over 18 months, 150 patients took the supplement, and more than 60% experienced a positive PSA response. Those with the lowest-risk cancer showed the most benefit, requiring fewer follow-up MRIs and biopsies. Patients who responded well to the supplement were also less likely to need active treatment. These findings suggest that fermented soy may support men on active surveillance by helping identify who can safely continue monitoring their cancer and who may require treatment, potentially reducing unnecessary procedures and improving quality of life.

**Assessment Category**	**Score**	**Assessment Category**	**Score**	**Assessment Category**	**Score**
Comprehension Score Rater 1	5	Gunning Fog Score	18.8	Words	143
Comprehension Score Rater 2	5	SMOG Index	13.5	Complex words	33
Reading Age (years)	21.5	Coleman Liau Index	17.4	Percentage of complex words	23.08
Flesch-Kincaid Reading Ease	29.4	Automated Readability Index	17.1	Average words per sentence	23.83
Flesch-Kincaid Grade Level	15.1	Sentences	6	Average syllables per word	1.81

<u>Lay Summary (Extended Prompt)</u>

Prostate cancer is often slow-growing, and many men with low-risk prostate cancer choose active surveillance, meaning they delay treatment while closely monitoring the disease. This study examined whether a nutritional supplement made from fermented soy could help manage prostate cancer in these patients. Over 18 months, 150 men took this supplement, and researchers tracked changes in their PSA levels, a common marker for prostate cancer progression. More than half of the patients showed a beneficial PSA response, especially those with the lowest-risk cancer. This led to fewer follow-up biopsies and MRI scans, reducing the need for additional medical procedures. The results suggest that fermented soy may help doctors better identify which patients can safely stay on active surveillance and which may need treatment. While more research is needed, these findings offer a promising, natural approach to managing prostate cancer with fewer interventions.

**Assessment Category**	**Score**	**Assessment Category**	**Score**	**Assessment Category**	**Score**
Comprehension Score Rater 1	5	Gunning Fog Score	14.9	Words	146
Comprehension Score Rater 2	5	SMOG Index	10.7	Complex words	24
Reading Age (years)	19.5	Coleman Liau Index	16.3	Percentage of complex words	16.44
Flesch-Kincaid Reading Ease	43.7	Automated Readability Index	14.7	Average words per sentence	20.86
Flesch-Kincaid Grade Level	12.3	Sentences	7	Average syllables per word	1.68

Manuscript 36

Salciccia S, Santarelli V, Di Pierro GB, Del Giudice F, Bevilacqua G, Di Lascio G, et al. Real-Life Comparative Analysis of Robotic-Assisted Versus Laparoscopic Radical Prostatectomy in a Single Centre Experience. Cancers [Internet]. 2024 Oct 25;16(21):3604. Available from: http://dx.doi.org/10.3390/cancers16213604

Abstract

Background: The advantage of a robotic-assisted (RARP) over a laparoscopic (LRP) approach in radical prostatectomy (RP) remains to be demonstrated. Aim: The aim of the study is to use a homogeneous population in real life and single primary surgeon surgery to analyze the oncological and functional results based on the type of surgical approach and pathological features. Methods: This is a prospective trial on non-metastatic prostate cancer (PCa) patients considered after a multidisciplinary decision to conduct a RP, using a RARP or LRP approach. A real-life setting was analyzed at our Urological Departments using homogeneous criteria for the management of PCa cases and a single surgeon experience on 444 cases (284 LRP and 160 RARP). Results: Mean operating time was significantly lower in RARP (153.21 ± 25.1 min) than in LRP (173.33 ± 44.3 min) (p p p = 0.430). Surgical margins (SM) positivity was not significantly higher in the RARP group (20.0%) when compared to the LRP group (15.9%) (p = 0.145). During the postoperative follow-up, a biochemical recurrence (BCR) was detected in 14.4% and 7.5% of cases in the LRP and RARP group, respectively, (p = 0.014). Better results of PAD tests at 3-month intervals using the RARP approach (mean pad weight 75.57 ± 122 g and 14 ± 42 g, respectively, in LRP and RARP (p < 0.01)) were described. Conclusions: In the comparison between the RARP and LRP approach, a clear advantage of the robotic approach is a significant reduction in operating times, days of hospitalization, and postoperative catheterization compared to laparoscopic surgery. It is not possible to describe any certain oncological advantage both in terms of surgical margins and pathological lymph nodes removed. In RARP cases a reduction to the limit of significance is described in terms of biochemical recurrence. RARP produces a more rapid recovery

of urinary continence at 3 months postoperatively without significant advantages in terms of erective potency recovery.

Keywords:

laparoscopic surgery, prostatic neoplasm, radical prostatectomy, robotic surgery

<u>Original Lay Summary</u>

In the comparison between the RARP and LRP approach, a clear advantage of the robotic approach is a significant reduction in operating time, days of hospitalization, and duration of postoperative catheterization compared to laparoscopic surgery. It is not possible to describe any certain oncological advantages, both in terms of surgical margins and pathological lymph nodes removed. In RARP cases a reduction to the limit of significance is described in terms of biochemical recurrence. RARP produces a more rapid recovery of urinary continence at 3 months postoperatively without significant advantages in terms of erective potency recovery.

**Assessment Category**	**Score**	**Assessment Category**	**Score**	**Assessment Category**	**Score**
Comprehension Score Rater 1	4	Gunning Fog Score	20.9	Words	95
Comprehension Score Rater 2	4	SMOG Index	15.0	Complex words	27
Reading Age (years)	23.5	Coleman Liau Index	17.7	Percentage of complex words	18.42
Flesch-Kincaid Reading Ease	11.7	Automated Readability Index	17.3	Average words per sentence	23.75
Flesch-Kincaid Grade Level	17.5	Sentences	4	Average syllables per word	2.02

Lay Summary (Simple Prompt)

Prostate cancer is one of the most common cancers in men, and surgery is often needed to remove the prostate. This study compares two surgical techniques for prostate removal: robotic-assisted surgery and traditional keyhole (laparoscopic) surgery. Researchers analyzed 444 patients treated at a single hospital by the same experienced surgeon to ensure fair comparisons. The findings show that robotic-assisted surgery leads to shorter operating times, faster recovery, and earlier return of bladder control compared to laparoscopic surgery. However, both techniques were similar in terms of cancer control and surgical precision. Although robotic-assisted surgery showed a slight reduction in cancer recurrence, the difference was not strong enough to be definitive. These results provide valuable insights for doctors and patients when choosing the best surgical approach for prostate cancer treatment, balancing recovery benefits with long-term cancer outcomes.

**Assessment Category**	**Score**	**Assessment Category**	**Score**	**Assessment Category**	**Score**
Comprehension Score Rater 1	5	Gunning Fog Score	19.2	Words	139
Comprehension Score Rater 2	5	SMOG Index	13.6	Complex words	39
Reading Age (years)	21.5	Coleman Liau Index	18.4	Percentage of complex words	28.06
Flesch-Kincaid Reading Ease	22.3	Automated Readability Index	15.9	Average words per sentence	19.86
Flesch-Kincaid Grade Level	15.1	Sentences	7	Average syllables per word	1.94

Lay Summary (Extended Prompt)

Prostate cancer is one of the most common cancers in men, and surgery is a key treatment option. This study compares two types of surgery used to remove the prostate: robotic-assisted surgery and traditional laparoscopic surgery. Researchers studied 444 patients to see which method leads to better outcomes. They found that robotic-assisted surgery had shorter operation times, shorter hospital stays, and faster recovery of urinary control within three months. However, both methods were equally effective in removing cancer, and there was no clear difference in cancer recurrence rates. While robotic-assisted surgery may offer certain advantages in recovery, it is not yet proven to be superior in cancer control. These findings help doctors and patients understand the benefits and limitations of each approach, allowing them to make informed decisions about prostate cancer treatment.

**Assessment Category**	**Score**	**Assessment Category**	**Score**	**Assessment Category**	**Score**
Comprehension Score Rater 1	5	Gunning Fog Score	17.2	Words	135
Comprehension Score Rater 2	5	SMOG Index	12.4	Complex words	32
Reading Age (years)	20.5	Coleman Liau Index	16.4	Percentage of complex words	23.7
Flesch-Kincaid Reading Ease	33.1	Automated Readability Index	14.0	Average words per sentence	19.29
Flesch-Kincaid Grade Level	13.4	Sentences	7	Average syllables per word	1.82

Manuscript 37

Liu J, Zhang H, Woon DTS, Perera M, Lawrentschuk N. Predicting Biochemical Recurrence of Prostate Cancer Post-Prostatectomy Using Artificial Intelligence: A Systematic Review. Cancers [Internet]. 2024 Oct 25;16(21):3596. Available from: http://dx.doi.org/10.3390/cancers16213596

Abstract

Background/Objectives: Biochemical recurrence (BCR) after radical prostatectomy (RP) is a significant predictor of distal metastases and mortality in prostate cancer (PCa) patients. This systematic review aims to evaluate the accuracy of artificial intelligence (AI) in predicting BCR post-RP. Methods: Adhering to PRISMA guidelines, a comprehensive literature search was conducted across Medline, Embase, Web of Science, and IEEE Xplore. Studies were included if they utilised AI to predict BCR in patients post-RP. Studies involving patients who underwent radiotherapy or salvage RP were excluded. This systematic review was registered on PROSPERO (International prospective register of systematic reviews) under the ID CRD42023482392. Results: After screening 9764 articles, 24 met the inclusion criteria. The included studies involved 27,216 patients, of whom 7267 developed BCR. AI algorithms developed using radiological parameters demonstrated higher predictive accuracy (median AUROC of 0.90) compared to algorithms based solely on pathological variables (median AUROC of 0.74) or clinicopathological variables (median AUROC of 0.81). According to the Prediction Model Risk of Bias Assessment Tool (PROBAST), the overall risk of bias was unclear in three studies due to ambiguous inclusion criteria and the exclusion of many patients because of missing follow-up data. In seven studies, the developed AI outperformed or was at least equivocal to traditional methods of BCR prediction. Conclusions: AI shows promise in predicting BCR post-RP, particularly when radiological data were used in its development. However, the significant variability in AI performance and study methodologies highlights the need for larger, standardised prospective studies with external validation prior to clinical application.

Keywords:

artificial intelligence, biochemical recurrence, convolutional neural network, deep learning, machine learning, prostate cancer, prostatic neoplasm

<u>Original Lay Summary</u>

Biochemical recurrence (BCR) of prostate cancer (PCa) after surgery is marked by an increase in prostate-specific antigen. BCR is associated with the risk of metastatic disease and/or death. This review evaluates the ability of artificial intelligence (AI) to predict BCR in PCa post-operatively. AI demonstrated high accuracy, especially when radiological features are utilised in its development. AI occasionally outperforms traditional methods of BCR prediction. However, due to the limited number of high-quality studies and insufficient external validation, further research is necessary to confirm the reliability and effectiveness of these AI techniques before they can be widely used in clinical practice.

**Assessment Category**	**Score**	**Assessment Category**	**Score**	**Assessment Category**	**Score**
Comprehension Score Rater 1	5	Gunning Fog Score	19.3	Words	103
Comprehension Score Rater 2	5	SMOG Index	13.5	Complex words	33
Reading Age (years)	21.5	Coleman Liau Index	18.2	Percentage of complex words	32.04
Flesch-Kincaid Reading Ease	12.0	Automated Readability Index	14.4	Average words per sentence	17.17
Flesch-Kincaid Grade Level	15.9	Sentences	6	Average syllables per word	2.1

Lay Summary (Simple Prompt)

Prostate cancer is one of the most common cancers in men, and after surgery to remove the prostate (radical prostatectomy), some patients experience a return of the disease, known as biochemical recurrence. Detecting this early is crucial to guide further treatment. This study reviews existing research on how artificial intelligence (AI) can help predict which patients are at higher risk of recurrence. By analyzing data from 27,216 patients, researchers found that AI models using medical imaging were more accurate in predicting recurrence than those relying only on lab results or pathology reports. Some AI models performed as well as, or even better than, traditional prediction methods. However, the study also found inconsistencies in how AI models were developed and tested. To ensure AI can be used reliably in clinical practice, larger studies with standardized methods are needed to confirm its accuracy and improve early detection for better patient outcomes.

**Assessment Category**	**Score**	**Assessment Category**	**Score**	**Assessment Category**	**Score**
Comprehension Score Rater 1	5	Gunning Fog Score	16.3	Words	150
Comprehension Score Rater 2	5	SMOG Index	11.8	Complex words	29
Reading Age (years)	19.5	Coleman Liau Index	15.6	Percentage of complex words	19.33
Flesch-Kincaid Reading Ease	33.4	Automated Readability Index	14.4	Average words per sentence	21.43
Flesch-Kincaid Grade Level	13.9	Sentences	7	Average syllables per word	1.79

<u>Lay Summary (Extended Prompt)</u>

Prostate cancer can come back even after surgery, a condition known as biochemical recurrence. Doctors want to predict which patients are at higher risk of this happening, as early detection can help guide treatment. This study reviews research on how artificial intelligence (AI) can be used to make these predictions. By analyzing data from 27,216 patients, researchers found that AI models using imaging scans were the most accurate, outperforming those based only on lab results or doctor's notes. However, some studies had weaknesses, such as missing data or unclear patient selection. While AI shows great promise in improving prostate cancer care, more large-scale studies are needed to confirm its reliability before it can be widely used in hospitals. This research highlights how AI could help doctors make better decisions, leading to earlier interventions and improved patient outcomes.

**Assessment Category**	**Score**	**Assessment Category**	**Score**	**Assessment Category**	**Score**
Comprehension Score Rater 1	5	Gunning Fog Score	14.6	Words	139
Comprehension Score Rater 2	5	SMOG Index	10.5	Complex words	23
Reading Age (years)	18.5	Coleman Liau Index	15.4	Percentage of complex words	16.55
Flesch-Kincaid Reading Ease	43.0	Automated Readability Index	13.5	Average words per sentence	19.86
Flesch-Kincaid Grade Level	12.2	Sentences	7	Average syllables per word	1.7

Manuscript 38

Puri D, Pandit K, Choi N, Rose BS, McKay RR, Bagrodia A. Striving for Equity: Examining Health Disparities in Urologic Oncology. Cancers [Internet]. 2024 Oct 22;16(21):3559. Available from: http://dx.doi.org/10.3390/cancers16213559

Abstract

Health disparities in urologic oncology, particularly in prostate, bladder, kidney, and testicular cancers, significantly impact patient outcomes across different demographic groups. This narrative review aims to investigate the extent and drivers of these disparities, focusing on the influence of race, socioeconomic status, and geographic location on diagnosis, treatment, and survival outcomes. We conducted a comprehensive review of the existing literature and analyzed data from national cancer databases to identify patterns of inequity. Our findings reveal that minority populations, individuals with lower socioeconomic status, and those residing in underserved areas are less likely to receive timely and guideline-based care, leading to worse outcomes. This review underscores the urgent need for targeted interventions, including policy reforms, health system restructuring, enhanced community outreach, and increased funding for disparity-focused research, to ensure equitable access to high-quality oncologic care. Addressing these disparities is crucial for improving cancer outcomes and achieving health equity in urologic oncology.

Keywords:

bladder cancer, cancer treatment equity, clinical decision making, health disparities, prostate cancer, racial disparities, socioeconomic status, urologic oncology

<u>Original Lay Summary</u>

This review explores the significant differences in the diagnosis, treatment, and outcomes of urologic cancers, including prostate, bladder, kidney, and testicular cancers, among different population groups. By examining factors like race, socioeconomic status, and access to specialized care, we aim to highlight the disparities that exist in urologic oncology and suggest strategies to ensure that all patients, regardless of their background, receive equitable treatment. The findings from this study are crucial for clinicians, policymakers, and researchers to understand the barriers faced by underserved populations and to develop interventions that can improve cancer care for everyone.

Assessment Category	Score	Assessment Category	Score	Assessment Category	Score
Comprehension Score Rater 1	4	Gunning Fog Score	23.6	Words	95
Comprehension Score Rater 2	4	SMOG Index	16.9	Complex words	26
Reading Age (years)	24.0	Coleman Liau Index	20.3	Percentage of complex words	27.4
Flesch-Kincaid Reading Ease	2.8	Automated Readability Index	23.3	Average words per sentence	31.7
Flesch-Kincaid Grade Level	20.7	Sentences	3	Average syllables per word	2.0

<u>Lay Summary (Simple Prompt)</u>

Health disparities in urologic cancers, such as prostate, bladder, kidney, and testicular cancer, can lead to worse outcomes for certain groups of people. This study explores how factors like race, income, and where a person lives affect their chances of being diagnosed early, receiving proper treatment, and surviving these cancers. By reviewing existing research and analyzing national cancer data, the authors found that minority groups, lower-income individuals, and those in underserved areas often face barriers to timely and effective care. As a result, their health outcomes are poorer compared to others. The study highlights the urgent need for policy changes, better healthcare access, and increased support for communities facing these challenges. Addressing these disparities can improve cancer treatment for all and move the healthcare system toward greater fairness and equity in urologic oncology.

**Assessment Category**	**Score**	**Assessment Category**	**Score**	**Assessment Category**	**Score**
Comprehension Score Rater 1	5	Gunning Fog Score	15.2	Words	134
Comprehension Score Rater 2	5	SMOG Index	10.8	Complex words	21
Reading Age (years)	20.5	Coleman Liau Index	12.6	Percentage of complex words	15.7
Flesch-Kincaid Reading Ease	32.6	Automated Readability Index	16.5	Average words per sentence	22.3
Flesch-Kincaid Grade Level	14.3	Sentences	6	Average syllables per word	1.8

Lay Summary (Extended Prompt)

Cancer in the urinary system, like prostate, bladder, kidney, and testicular cancer, does not affect everyone equally. Some groups of people—especially those from minority backgrounds, lower-income households, or underserved areas—often face delays in diagnosis and treatment, leading to worse health outcomes. This study explores why these differences exist and how they impact patient care. By reviewing past research and analyzing large cancer databases, the authors found that certain groups receive less access to high-quality treatment. The study highlights the need for changes in healthcare policies, better community support, and increased funding for research focused on reducing these health gaps. By addressing these issues, doctors and policymakers can work toward fair and equal cancer care for all patients, improving survival rates and overall well-being. Ensuring fairness in cancer treatment is a crucial step toward better health for everyone.

**Assessment Category**	**Score**	**Assessment Category**	**Score**	**Assessment Category**	**Score**
Comprehension Score Rater 1	5	Gunning Fog Score	14.9	Words	140
Comprehension Score Rater 2	5	SMOG Index	10.7	Complex words	24
Reading Age (years)	19.5	Coleman Liau Index	17.9	Percentage of complex words	17.1
Flesch-Kincaid Reading Ease	37.3	Automated Readability Index	15.5	Average words per sentence	20.0
Flesch-Kincaid Grade Level	13.0	Sentences	7	Average syllables per word	1.8

Manuscript 39

Pisotskyi O, Petrasz P, Zorga P, Gałęski M, Szponar P, Brzeźniakiewicz-Janus K, et al. Enhancing Prostate Cancer Staging: Association of 68Ga-PSMA PET/CT Imaging with Histopathological Grading in Treatment-Naive Patients. Cancers [Internet]. 2024 Oct 18;16(20):3526. Available from: http://dx.doi.org/10.3390/cancers16203526

Abstract

Objective: This study aimed to evaluate the correlation between 68Ga-PSMA uptake in PSMA PET/CT in primary prostate cancer (PC) and its histopathological grading (Gleason score and ISUP grade). Additionally, we compared preoperative biopsy histopathological findings with definitive pathology results in radical prostatectomy (RP) specimens.

Methods: We retrospectively analyzed 86 patients who underwent 68Ga-PSMA PET/CT for primary PC staging, of which 40 patients later underwent radical prostatectomy. PET/CT results, including SUVmax values, were correlated with GS and PSA concentrations. Histopathology reports were analyzed and compared between biopsy and final pathology results following RP.

Results: A significant positive correlation was observed between SUVmax and ISUP grades (Pearson's $\rho = 0.34$, $p < 0.001$), with higher SUVmax values associated with more advanced grades. A cut-off SUVmax value of 5.64 was determined to predict upstaging in patients, yielding a sensitivity of 76% and a specificity of 60% (AUC = 0.82, 95% CI: 0.70-0.94). Additionally, 57.5% of patients experienced a grade shift following RP, with a 35% upgrade and 22.5% downgrade in ISUP grades.

Conclusion: 68Ga-PSMA PET/CT demonstrated high sensitivity in detecting high-risk prostate cancer, particularly in patients with GS > 7 or PSA levels ≥ 10 ng/mL. The findings suggest that this imaging modality may be less effective for the staging of

patients with lower GS or PSA values, that is, low-risk PCa. Further prospective studies are necessary to validate these results.

Keywords:

68Ga-PSMA PET/CT, PSMA expression, SUVmax correlation, biopsy vs. final pathology, histopathological grading, prostate cancer staging

Original Lay Summary

This study investigates the correlation between 68Ga-PSMA PET/CT imaging and histopathological grading in treatment-naive prostate cancer patients. We retrospectively analyzed 86 patients who underwent 68Ga-PSMA PET/CT scans for prostate cancer staging, with a subset of 40 patients also undergoing radical prostatectomy. The PET/CT results, specifically SUVmax values, were compared with Gleason scores and PSA levels to assess their association with cancer grades. Results indicated a significant positive correlation between SUVmax and ISUP grades, suggesting that higher SUVmax values correspond to more advanced prostate cancer stages. Furthermore, the analysis of biopsy and radical prostatectomy pathology reports revealed a substantial rate of grading changes, underscoring the limitations of biopsy alone for accurate staging. These findings highlight the potential utility of 68Ga-PSMA PET/CT as a diagnostic tool in stratifying prostate cancer risk and guiding treatment decisions. Future studies are recommended to validate these observations and explore further applications of this imaging modality.

**Assessment Category**	**Score**	**Assessment Category**	**Score**	**Assessment Category**	**Score**
Comprehension Score Rater 1	5	Gunning Fog Score	19.2	Words	153
Comprehension Score Rater 2	5	SMOG Index	13.8	Complex words	40
Reading Age (years)	23.5	Coleman Liau Index	20.1	Percentage of complex words	26.1
Flesch-Kincaid Reading Ease	13.8	Automated Readability Index	18.3	Average words per sentence	21.9

Flesch-Kincaid Grade Level	16.8	Sentences	7	Average syllables per word	2.0

Lay Summary (Simple Prompt)

Prostate cancer staging helps doctors determine the best treatment options for patients. This study explored whether a specialized imaging technique, called 68Ga-PSMA PET/CT, can accurately predict the severity of prostate cancer before surgery. Researchers analyzed scans from 86 patients and compared imaging results with tissue samples collected during surgery. They found that higher tumor activity on the scan was linked to more aggressive cancer. However, in over half of the cases, the initial biopsy results differed from the final diagnosis after surgery, showing that early assessments may sometimes be inaccurate. The study identified a specific scan value that may help predict when a patient's cancer stage will change after surgery. These findings suggest that 68Ga-PSMA PET/CT could improve prostate cancer staging, leading to better treatment decisions. Further research is needed to confirm its full potential in routine clinical practice.

**Assessment Category**	**Score**	**Assessment Category**	**Score**	**Assessment Category**	**Score**
Comprehension Score Rater 1	5	Gunning Fog Score	15.0	Words	141
Comprehension Score Rater 2	5	SMOG Index	10.8	Complex words	28
Reading Age (years)	19.5	Coleman Liau Index	17.3	Percentage of complex words	19.9
Flesch-Kincaid Reading Ease	37.7	Automated Readability Index	13.9	Average words per sentence	17.6
Flesch-Kincaid Grade Level	12.4	Sentences	8	Average syllables per word	1.8

Lay Summary (Extended Prompt)

Prostate cancer is a common disease, and accurate staging is crucial for choosing the best treatment. This study explores how a special imaging technique, called 68Ga-PSMA PET/CT, helps predict the severity of prostate cancer before surgery. By analyzing scans from patients with untreated prostate cancer, researchers found that higher scan values were linked to more aggressive disease. The study also showed that initial biopsy results often differed from final surgical findings, meaning some cancers were actually more or less severe than first thought. These results suggest that 68Ga-PSMA PET/CT could improve how doctors assess prostate cancer, especially in higher-risk cases. However, the scan may be less useful for patients with less aggressive cancer. Future studies are needed to confirm these findings, which could lead to better treatment decisions and improved outcomes for patients with prostate cancer.

**Assessment Category**	**Score**	**Assessment Category**	**Score**	**Assessment Category**	**Score**
Comprehension Score Rater 1	5	Gunning Fog Score	12.8	Words	139
Comprehension Score Rater 2	5	SMOG Index	9.1	Complex words	17
Reading Age (years)	18.5	Coleman Liau Index	16.5	Percentage of complex words	12.2
Flesch-Kincaid Reading Ease	47.3	Automated Readability Index	14.3	Average words per sentence	19.9
Flesch-Kincaid Grade Level	11.6	Sentences	7	Average syllables per word	1.7

Manuscript 40

Wenzel M, Hoeh B, Humke C, Siech C, Cano Garcia C, Salomon G, et al. Assessment of Different Castration Resistance Definitions and Staging Modalities in Metastatic Castration-Resistant Prostate Cancer. Cancers [Internet]. 2024 Oct 17;16(20):3506. Available from: http://dx.doi.org/10.3390/cancers16203506

Abstract

Background/Objectives: Progression to metastatic castration-resistant prostate cancer (mCRPC) is defined either biochemically, radiographically or both. Moreover, staging for mCRPC can be performed either conventionally or with molecular imaging such as prostate-specific membrane antigen computer tomography (PSMA-PET/CT). Methods: We relied on the Frankfurt Metastatic Cancer Database of the Prostate (FRAMCAP) database to compare progression-free (PFS) and overall survival (OS) outcomes regarding the cause of castration resistance and the staging modality used. Results: Overall, 35% progressed to mCRPC biochemically vs. 23% radiographically vs. 42% biochemically + radiographically. The PSA nadir in mHSPC (1.4 vs. 0.4 vs. 0.8 ng/mL) and PSA level at mCRPC progression (15 vs. 2 vs. 21 ng/mL, both $p \leq 0.01$) were significantly higher for biochemical vs. radiographic vs. both progressed patients. In PFS and OS analyses, no significant differences were observed among all three compared groups. In the comparison of the staging used for progression to mCRPC, 67% received conventional vs. 33% PSMA-PET/CT, with higher metastatic burden in mHSPC and osseous lesions in mCRPC for conventionally staged patients (both p p Conclusions: The cause of progression to mCRPC seems not to influence cancer-control outcomes, despite important baseline tumor characteristic differences. The PSMA-PET/CT staging modality might be associated with better PFS and OS outcomes, possibly due to its more sensitive detection of progression or new metastatic lesions.

Keywords:

PET, PFS, PSMA, mCRPC, survival

<u>Original Lay Summary</u>

The current real-world study suggests that important differences exist in patients progressing to mCRPC. Specifically, we found that a proportion of over 20% of patients progress without a PSA level rise, emphasizing the importance of regular staging intervals. Moreover, we found that patients with worse PSA responses are at higher risk for biochemical progression, emphasizing the need for regular PSA check-ups and kinetic measurements. However, no differences in PFS and OS outcomes between these patients were observed. Finally, staging with PSMA-PET/CT might provide better PFS and OS, relative to conventionally staged mCRPC patients. However, this observation may be substantially influenced by the detection of earlier disease progression and the possibility of treatment in patients with a lower metastatic burden, as well as more contemporary patients with better treatment options.

**Assessment Category**	**Score**	**Assessment Category**	**Score**	**Assessment Category**	**Score**
Comprehension Score Rater 1	5	Gunning Fog Score	18.2	Words	132
Comprehension Score Rater 2	4	SMOG Index	13.1	Complex words	31
Reading Age (years)	21.5	Coleman Liau Index	17.4	Percentage of complex words	23.5
Flesch-Kincaid Reading Ease	31.3	Automated Readability Index	16.1	Average words per sentence	22.0
Flesch-Kincaid Grade Level	14.4	Sentences	6	Average syllables per word	1.8

<u>Lay Summary (Simple Prompt)</u>

Prostate cancer can become resistant to hormone therapy, leading to a stage called metastatic castration-resistant prostate cancer (mCRPC). Doctors use different methods to detect this progression, including blood tests (biochemical progression), imaging scans (radiographic progression), or both. This study compared how these different definitions of disease progression and the imaging techniques used for staging impact patient outcomes. Researchers analyzed data from a cancer database and found that while patients had different tumor characteristics depending on how their disease was classified, there was no major difference in overall survival between groups. However, a newer imaging method called PSMA-PET/CT may improve patient outcomes by detecting cancer spread more accurately. These findings suggest that refining how doctors diagnose and monitor mCRPC could lead to better treatment strategies, helping patients receive the most effective care based on their disease characteristics.

**Assessment Category**	**Score**	**Assessment Category**	**Score**	**Assessment Category**	**Score**
Comprehension Score Rater 1	5	Gunning Fog Score	20.2	Words	138
Comprehension Score Rater 2	5	SMOG Index	14.5	Complex words	38
Reading Age (years)	23.5	Coleman Liau Index	20.2	Percentage of complex words	27.5
Flesch-Kincaid Reading Ease	21.6	Automated Readability Index	18.9	Average words per sentence	23.0
Flesch-Kincaid Grade Level	16.0	Sentences	6	Average syllables per word	1.9

Lay Summary (Extended Prompt)

Prostate cancer can become more advanced when it stops responding to hormone therapy, a stage called metastatic castration-resistant prostate cancer (mCRPC). Doctors use different ways to determine when this happens—by measuring changes in blood tests, imaging scans, or both. This study examined how these different methods impact patient outcomes using data from a cancer database. Researchers found that whether the disease progressed based on blood tests, imaging, or both did not change overall survival rates. However, a newer imaging method called PSMA-PET/CT detected more cancer spread than traditional scans and was linked to better outcomes. This suggests that using more advanced imaging may help doctors detect cancer earlier and make better treatment decisions. These findings could improve how doctors monitor and treat patients with advanced prostate cancer, potentially leading to better survival and care strategies in the future.

**Assessment Category**	**Score**	**Assessment Category**	**Score**	**Assessment Category**	**Score**
Comprehension Score Rater 1	5	Gunning Fog Score	16.0	Words	140
Comprehension Score Rater 2	5	SMOG Index	11.6	Complex words	28
Reading Age (years)	20.5	Coleman Liau Index	17.5	Percentage of complex words	20.0
Flesch-Kincaid Reading Ease	42.7	Automated Readability Index	15.2	Average words per sentence	20.0
Flesch-Kincaid Grade Level	12.3	Sentences	7	Average syllables per word	1.7

Manuscript 41

Chatterjee A, Fan X, Slear J, Asare G, Yousuf AN, Medved M, et al. Quantitative Multi-Parametric MRI of the Prostate Reveals Racial Differences. Cancers [Internet]. 2024 Oct 16;16(20):3499. Available from: http://dx.doi.org/10.3390/cancers16203499

Abstract

Purpose: This study investigates whether quantitative MRI and histology of the prostate reveal differences between races, specifically African Americans (AAs) and Caucasian Americans (CAs), that can affect diagnosis. Materials and Methods: Patients (98 CAs, 47 AAs) with known or suspected prostate cancer (PCa) underwent 3T MRI (T2W, DWI, and DCE-MRI) prior to biopsy or prostatectomy. Quantitative mpMRI metrics: ADC, T2, and DCE empirical mathematical model parameters were calculated. Results: AAs had a greater percentage of higher Gleason-grade lesions compared to CAs. There were no significant differences in the quantitative ADC and T2 values between AAs and CAs. The cancer signal enhancement rate (α) on DCE-MRI was significantly higher for AAs compared to CAs (AAs: 13.3 ± 9.3 vs. CAs: 6.1 ± 4.7 s-1, p -1 vs. CAs: 0.07 ± 0.07 s-1, p -1 vs. CAs: 0.07 ± 0.08 s-1, p = 0.02). DCE significantly improves the differentiation of PCa from benign in AAs (α: 52%, β: 62% more effective in AAs compared to CAs). Histologic analysis showed cancers have a greater proportion (p = 0.04) of epithelium (50.9 ± 12.3 vs. 44.7 ± 12.8%) and lower lumen (10.5 ± 6.9 vs. 16.2 ± 6.8%) in CAs compared to AAs. Conclusions: This study shows that AAs have different quantitative DCE-MRI values for benign prostate and prostate cancer and different histologic makeup in PCa compared to CAs. Quantitative DCE-MRI can significantly improve the performance of MRI for PCa diagnosis in African Americans but is much less effective for Caucasian Americans.

Keywords:

African Americans, Caucasian Americans, DCE-MRI, MRI, prostate cancer, quantitative, racial differences

Original Lay Summary

This study investigated whether quantitative MRI and histology of the prostate reveal differences between races that can affect diagnosis. The cancer signal enhancement rate (α) on dynamic contrast-enhanced MRI (DCE-MRI) was significantly higher for African Americans (AAs) compared to Caucasian Americans (CAs). The signal washout rate (β) was significantly lower in benign tissue in AAs and significantly elevated in cancers in AAs. However, no significant differences were found for ADC and T2. The ROC analysis showed that the apparent diffusion coefficient (ADC) and T2 are slightly less effective in AAs compared to CAs. DCE significantly improves the differentiation of PCa from benign in AAs (α: 52%, β: 62% more effective in AAs compared to CAs). Histologic analysis showed that cancers have a greater proportion of epithelium and lower lumen in AAs compared to CAs. This study shows that the different races have different quantitative MRI values and histologic makeup. Quantitative DCE-MRI is highly effective and improves PCa diagnosis in African Americans.

**Assessment Category**	**Score**	**Assessment Category**	**Score**	**Assessment Category**	**Score**
Comprehension Score Rater 1	5	Gunning Fog Score	15.3	Words	164
Comprehension Score Rater 2	5	SMOG Index	12.0	Complex words	39
Reading Age (years)	19.5	Coleman Liau Index	15.3	Percentage of complex words	23.8
Flesch-Kincaid Reading Ease	31.0	Automated Readability Index	12.6	Average words per sentence	18.2
Flesch-Kincaid Grade Level	13.5	Sentences	9	Average syllables per word	1.9

Lay Summary (Simple Prompt)

Prostate cancer can affect people differently based on their racial background, which may impact how doctors diagnose and treat the disease. This study examined whether prostate cancer appears differently in African American and Caucasian American men using a special type of MRI scan before biopsy or surgery. Researchers analyzed imaging results and tissue samples from both groups. They found that African American men were more likely to have aggressive prostate cancer and showed unique patterns in MRI scans compared to Caucasian American men. A specific MRI technique, called contrast-enhanced imaging, was much better at detecting cancer in African American men but less effective for Caucasian American men. These findings suggest that using personalized imaging approaches based on racial differences could improve prostate cancer detection and treatment. This research may help doctors develop better screening and diagnostic tools, leading to more accurate and effective care for all patients.

**Assessment Category**	**Score**	**Assessment Category**	**Score**	**Assessment Category**	**Score**
Comprehension Score Rater 1	5	Gunning Fog Score	14.9	Words	148
Comprehension Score Rater 2	4	SMOG Index	13.1	Complex words	36
Reading Age (years)	20.5	Coleman Liau Index	17.9	Percentage of complex words	24.3
Flesch-Kincaid Reading Ease	27.6	Automated Readability Index	16.1	Average words per sentence	21.1
Flesch-Kincaid Grade Level	14.7	Sentences	7	Average syllables per word	1.9

Lay Summary (Extended Prompt)

Prostate cancer affects men of all races, but differences in how it appears on medical scans could impact diagnosis. This study compared prostate MRI scans from African American and Caucasian American men to see if there were differences in how cancer and normal tissue look. Researchers analyzed images from 145 men using a special MRI technique that measures changes in the prostate. They found that African American men had more aggressive cancer and unique patterns in their prostate tissue. A specific MRI method, called DCE-MRI, was much better at detecting cancer in African American men than in Caucasian men. These findings suggest that prostate cancer screening and diagnosis may need to be adjusted based on a person's race to improve accuracy. Understanding these differences could help doctors make better decisions when diagnosing and treating prostate cancer, leading to improved care for all patients.

**Assessment Category**	**Score**	**Assessment Category**	**Score**	**Assessment Category**	**Score**
Comprehension Score Rater 1	5	Gunning Fog Score	13.2	Words	144
Comprehension Score Rater 2	5	SMOG Index	11.4	Complex words	27
Reading Age (years)	18.5	Coleman Liau Index	15.2	Percentage of complex words	18.8
Flesch-Kincaid Reading Ease	41.4	Automated Readability Index	13.7	Average words per sentence	20.6
Flesch-Kincaid Grade Level	12.6	Sentences	7	Average syllables per word	1.7

Manuscript 42

Kim SH, Kim JY, Hwang MJ. Magnetic Resonance Elastography for the Detection and Classification of Prostate Cancer. Cancers [Internet]. 2024 Oct 15;16(20):3494. Available from: http://dx.doi.org/10.3390/cancers16203494

Abstract

We investigated the feasibility of magnetic resonance elastography (MRE) using a pelvic acoustic driver for the detection and classification of prostate cancer (PCa). A total of 75 consecutive patients (mean age, 70; range, 56-86) suspected of having PCa and who underwent multi-parametric MRI including MRE and subsequent surgical resection were included. The analyzed regions consisted of cancer (n = 69), benign prostatic hyperplasia (BPH) (n = 70), and normal parenchyma (n = 70). A histopathologic topographic map served as the reference standard for each region. One radiologist and one pathologist performed radiologic-pathologic correlation, and the radiologist measured stiffness values in each region of interest on elastograms automatically generated by dedicated software. Paired t-tests were used to compare stiffness values between two regions. ROC curve analysis was also used to extract a cutoff value between two regions. The stiffness value of PCa (unit, kilopascal (kPa); 4.9 ± 1.1) was significantly different to that of normal parenchyma (3.6 ± 0.3, p p = 0.0454). Under a cutoff value of 4.2 kPa, a maximum accuracy of 87% was estimated, with a sensitivity of 73%, a specificity of 99%, and an AUC of 0.839 for discriminating PCa from normal parenchyma. Between PCa and BPH, a maximum accuracy of 62%, a sensitivity of 70%, a specificity of 56%, and an AUC of 0.598 were estimated at a 4.5 kPa cutoff. The stiffness values tended to increase as the ISUP grade increased. In conclusion, it is feasible to detect and classify PCa using pelvic MRE. Our observations suggest that MRE could be a supplement to multi-parametric MRI for PCa detection.

Keywords:

magnetic resonance elastography (MRE), magnetic resonance imaging (MRI), prostate cancer, prostate gland

<u>Original Lay Summary</u>

Multi-parametric MRI is a first-line imaging modality for prostate cancer detection. Magnetic resonance elastography (MRE) is a useful tool for measuring parenchymal stiffness to diagnose liver fibrosis. A known physical characteristic of prostate cancer is that it is harder than a normal prostate gland. We hypothesized that the quantitative stiffness value derived from MRE could be used to discriminate prostate cancer from benign prostate hyperplasia and normal parenchyma. Until now, the application of MRE to prostate cancer has been sporadic; it has only been used for investigational purposes due to the uncomfortable perineal placement of the acoustic device and inconvenience to patients during preparation. Therefore, we attempted to place an acoustic driver on the lower abdominal wall and determined that detecting and classifying prostate cancer via MRE is feasible. Our observation of 75 consecutive patients revealed that the stiffness value of prostate cancer was significantly different from normal parenchyma, and an accuracy of 87% was estimated at a cutoff value of 4.2 kPa in discriminating prostate cancer from normal parenchyma. In terms of differentiating prostate cancer from benign prostate hyperplasia, an accuracy of 62% was estimated. Additionally, the stiffness values tended to increase as the ISUP grade increased. This observation suggests that the stiffness value derived from pelvic MRE is helpful for detecting and classifying prostate cancer and could be an adjunct to multi-parametric MRI.

**Assessment Category**	**Score**	**Assessment Category**	**Score**	**Assessment Category**	**Score**
Comprehension Score Rater 1	5	Gunning Fog Score	17.6	Words	229
Comprehension Score Rater 2	5	SMOG Index	12.7	Complex words	53
Reading Age (years)	21.5	Coleman Liau Index	16.9	Percentage of complex words	23.1

Flesch-Kincaid Reading Ease	23.5	Automated Readability Index	15.2	Average words per sentence	20.8
Flesch-Kincaid Grade Level	15.2	Sentences	11	Average syllables per word	1.9

Lay Summary (Simple Prompt)

Magnetic resonance elastography (MRE) is a specialized imaging technique that measures tissue stiffness. This study explored whether MRE can help detect and classify prostate cancer by comparing the stiffness of cancerous, non-cancerous, and enlarged prostate tissues. Researchers analyzed MRE scans of 75 patients suspected of having prostate cancer, using surgical results as a reference. The findings showed that prostate cancer tissue is significantly stiffer than normal tissue, and MRE could distinguish between them with high accuracy. Stiffness values also tended to increase with cancer severity. However, differentiating cancer from benign enlargement of the prostate was more challenging. These results suggest that MRE could be a valuable addition to standard MRI scans, helping doctors improve prostate cancer detection and possibly assess its severity. This could lead to earlier and more precise diagnoses, benefiting patients and advancing prostate cancer imaging techniques.

**Assessment Category**	**Score**	**Assessment Category**	**Score**	**Assessment Category**	**Score**
Comprehension Score Rater 1	5	Gunning Fog Score	16.4	Words	140
Comprehension Score Rater 2	5	SMOG Index	11.9	Complex words	34
Reading Age (years)	20.5	Coleman Liau Index	19.2	Percentage of complex words	24.3
Flesch-Kincaid Reading Ease	27.7	Automated Readability Index	15.3	Average words per sentence	17.5
Flesch-Kincaid Grade Level	13.7	Sentences	8	Average syllables per word	1.9

Lay Summary (Extended Prompt)

Prostate cancer is a common disease in men, and early detection is important for better treatment. This study tested a special MRI technique called magnetic resonance elastography (MRE) to see if it can help find and classify prostate cancer. Researchers examined 75 patients suspected of having prostate cancer. They compared the stiffness of cancerous, normal, and enlarged prostate tissue using MRE. The results showed that prostate cancer tissue is stiffer than normal tissue and slightly stiffer than benign enlarged tissue. Using a specific stiffness value, MRE was able to detect cancer with high accuracy. These findings suggest that MRE could be a useful tool alongside standard MRI to improve prostate cancer detection. If confirmed in larger studies, this method could help doctors diagnose prostate cancer more accurately, leading to better treatment decisions and outcomes for patients.

**Assessment Category**	**Score**	**Assessment Category**	**Score**	**Assessment Category**	**Score**
Comprehension Score Rater 1	5	Gunning Fog Score	12.1	Words	136
Comprehension Score Rater 2	5	SMOG Index	8.8	Complex words	18
Reading Age (years)	17.5	Coleman Liau Index	16.5	Percentage of complex words	13.2
Flesch-Kincaid Reading Ease	45.3	Automated Readability Index	12.9	Average words per sentence	17.0
Flesch-Kincaid Grade Level	11.2	Sentences	8	Average syllables per word	1.7

Manuscript 43

Albers P, Kinnaird A. Advanced Imaging for Localized Prostate Cancer. Cancers [Internet]. 2024 Oct 15;16(20):3490. Available from: http://dx.doi.org/10.3390/cancers16203490

Abstract

Background/objectives: Prostate cancer is a prevalent malignancy often presenting without early symptoms. Advanced imaging technologies have revolutionized its diagnosis and management. This review discusses the principles, benefits, and clinical applications of multiparametric magnetic resonance imaging (mpMRI), micro-ultrasound (microUS), and prostate-specific membrane antigen positron emission tomography-computed tomography (PSMA PET/CT) in localized prostate cancer.

Methods: We conducted a comprehensive literature review of recent studies and guidelines on mpMRI, microUS, and PSMA PET/CT in prostate cancer diagnosis, focusing on their applications in biopsy-naïve patients, those with previous negative biopsies, and patients under active surveillance.

Results: MpMRI has demonstrated high sensitivity and negative predictive value in detecting clinically significant prostate cancer (csPCa). MicroUS, a newer technology, has shown promising results in early studies, with sensitivity and specificity comparable to mpMRI. PSMA PET/CT has emerged as a highly sensitive and specific imaging modality, particularly valuable for staging and detecting metastatic disease. All three technologies have been incorporated into urologic practice for prostate cancer diagnosis and management, with each offering unique advantages in different clinical scenarios.

Conclusions: Advanced imaging techniques, including mpMRI, microUS, and PSMA PET/CT, have significantly improved the accuracy of prostate cancer diagnosis,

staging, and management. These technologies enable more precise targeting of suspicious lesions during biopsy and therapy planning. However, further research, especially randomized controlled trials, is needed to fully establish the optimal use and inclusion of these imaging modalities in various stages of prostate cancer care.

Keywords:

MRI, PSMA PET, diagnosis, imaging, localized, micro-ultrasound, prostate biopsy, prostate cancer, targeted prostate biopsy

<u>Original Lay Summary</u>

This review paper explores advanced imaging technologies for prostate cancer because traditional methods often miss or misclassify cancers. The aim of this study is to provide a comprehensive overview of three key technologies, MRI, micro-ultrasound, and prostate-specific membrane antigen (PSMA) PET scans, and how they can improve prostate cancer detection and management. The findings showed that these techniques have improved our ability to detect prostate cancer; however, further studies are needed to determine each imaging technique's specific role in the diagnosis of prostate cancer.

**Assessment Category**	**Score**	**Assessment Category**	**Score**	**Assessment Category**	**Score**
Comprehension Score Rater 1	4	Gunning Fog Score	20.8	Words	86
Comprehension Score Rater 2	3	SMOG Index	14.9	Complex words	20
Reading Age (years)	23.5	Coleman Liau Index	18.2	Percentage of complex words	23.2
Flesch-Kincaid Reading Ease	20.3	Automated Readability Index	20.1	Average words per sentence	28.7
Flesch-Kincaid Grade Level	17.5	Sentences	3	Average syllables per word	1.9

Lay Summary (Simple Prompt)

Prostate cancer is one of the most common cancers in men, often showing no symptoms in its early stages. Detecting it accurately is crucial for proper treatment. This study reviews advanced imaging techniques—multiparametric MRI (mpMRI), micro-ultrasound (microUS), and PSMA PET/CT—that help improve diagnosis and treatment planning for prostate cancer. Researchers analyzed recent studies to compare how these technologies work in different patient groups, such as those undergoing their first biopsy or being monitored for cancer progression. The findings show that these imaging tools can detect significant prostate cancer more accurately, reducing the need for unnecessary biopsies and improving treatment decisions. While these technologies are becoming more common in medical practice, further research is needed to determine the best ways to use them in different stages of prostate cancer care. This study helps guide future research and clinical decisions, ultimately benefiting patients.

**Assessment Category**	**Score**	**Assessment Category**	**Score**	**Assessment Category**	**Score**
Comprehension Score Rater 1	5	Gunning Fog Score	16.8	Words	142
Comprehension Score Rater 2	5	SMOG Index	12.2	Complex words	31
Reading Age (years)	21.5	Coleman Liau Index	18.9	Percentage of complex words	21.8
Flesch-Kincaid Reading Ease	30.2	Automated Readability Index	16.4	Average words per sentence	20.3
Flesch-Kincaid Grade Level	14.1	Sentences	7	Average syllables per word	1.9

Lay Summary (Extended Prompt)

Prostate cancer is a common disease that often has no early symptoms, making early and accurate detection very important. This study looks at three advanced imaging techniques—multiparametric MRI (mpMRI), micro-ultrasound (microUS), and PSMA PET/CT—to see how well they help doctors find and manage prostate cancer. The researchers reviewed recent studies to understand how these imaging methods work in different situations, such as when a patient has never had a biopsy, had a negative biopsy before, or is being monitored for changes. The results show that all three methods improve cancer detection, with mpMRI and microUS being especially helpful for spotting significant cancers, while PSMA PET/CT is highly effective for identifying cancer spread. These imaging tools help doctors diagnose prostate cancer more accurately and plan better treatments, but more research is needed to determine the best way to use them in patient care.

**Assessment Category**	**Score**	**Assessment Category**	**Score**	**Assessment Category**	**Score**
Comprehension Score Rater 1	5	Gunning Fog Score	18.2	Words	143
Comprehension Score Rater 2	5	SMOG Index	12.7	Complex words	24
Reading Age (years)	21.5	Coleman Liau Index	16.1	Percentage of complex words	16.8
Flesch-Kincaid Reading Ease	32.3	Automated Readability Index	18.4	Average words per sentence	28.6
Flesch-Kincaid Grade Level	15.9	Sentences	5	Average syllables per word	1.7

Manuscript 44

Kaltsas A, Chrisofos M, Symeonidis EN, Zachariou A, Stavropoulos M, Kratiras Z, et al. To Drink or Not to Drink? Investigating Alcohol's Impact on Prostate Cancer Risk. Cancers [Internet]. 2024 Oct 12;16(20):3453. Available from: http://dx.doi.org/10.3390/cancers16203453

Abstract

Background/objectives: Prostate cancer (PCa) is a significant global health issue. The relationship between alcohol consumption and PCa risk has been the subject of extensive research, yet findings remain inconsistent. This review aims to clarify the association between alcohol intake and PCa risk, its aggressiveness, and the potential metabolic pathways involved in PCa onset.

Methods: A comprehensive literature search was conducted across multiple databases, including PubMed and MEDLINE, focusing on epidemiological studies, meta-analyses, cohort studies, and case-control studies. Studies evaluating alcohol consumption, prostate-specific antigen (PSA) levels, and PCa risk were included. The review also explored the roles of alcohol dehydrogenase (ADH) and aldehyde dehydrogenase (ALDH) in alcohol metabolism.

Results: The analysis reveals a complex relationship between alcohol consumption and PCa. Heavy alcohol intake is associated with an increased risk of PCa, particularly more aggressive forms, and higher mortality rates. However, studies also show weak or no association between moderate alcohol consumption and PCa. The variability in findings may be attributed to differences in alcohol types, regional factors, and study methodologies.

Conclusions: The link between alcohol consumption and PCa risk is multifaceted. While heavy drinking appears to increase the risk of aggressive PCa, the overall relationship remains unclear. Further research is needed to better understand these

associations and inform public health recommendations and cancer prevention strategies.

Keywords:

PSA levels, aggressive prostate cancer, alcohol consumption, cancer risk factors, carcinogenesis, chronic alcohol consumption, prostate cancer

<u>Original Lay Summary</u>

Prostate cancer is one of the most common cancers in men worldwide, and its causes are influenced by both genetic and lifestyle factors. One lifestyle factor that has shown mixed evidence is alcohol consumption. While some studies suggest that heavy drinking increases prostate cancer risk, others show little to no connection. This research aims to clarify the relationship between alcohol consumption and prostate cancer by reviewing and analyzing global studies. By exploring how different types of alcohol and drinking patterns may influence cancer risk, the goal is to provide more precise guidance for healthcare providers and patients. These findings can help inform public health recommendations and future research on cancer prevention strategies, especially for higher-risk populations.

**Assessment Category**	**Score**	**Assessment Category**	**Score**	**Assessment Category**	**Score**
Comprehension Score Rater 1	4	Gunning Fog Score	15.3	Words	117
Comprehension Score Rater 2	4	SMOG Index	11.1	Complex words	22
Reading Age (years)	19.5	Coleman Liau Index	17.9	Percentage of complex words	18.8
Flesch-Kincaid Reading Ease	38.8	Automated Readability Index	15.3	Average words per sentence	19.5
Flesch-Kincaid Grade Level	12.7	Sentences	6	Average syllables per word	1.8

Lay Summary (Simple Prompt)

Prostate cancer is a major health concern worldwide, and scientists are still trying to understand whether drinking alcohol affects the risk of developing this disease. This study reviews past research to explore the connection between alcohol consumption and prostate cancer risk, focusing on how different drinking patterns may influence the likelihood of developing aggressive forms of the disease. By analyzing multiple studies, researchers found that heavy drinking may increase the risk of aggressive prostate cancer and higher death rates, while moderate alcohol intake shows little or no clear effect. However, results vary depending on factors like alcohol type and study methods. These findings highlight the need for further research to clarify how alcohol influences prostate cancer risk. A better understanding of this link could help shape public health recommendations and cancer prevention strategies.

**Assessment Category**	**Score**	**Assessment Category**	**Score**	**Assessment Category**	**Score**
Comprehension Score Rater 1	5	Gunning Fog Score	16.7	Words	133
Comprehension Score Rater 2	5	SMOG Index	12.0	Complex words	26
Reading Age (years)	21.5	Coleman Liau Index	18.5	Percentage of complex words	19.6
Flesch-Kincaid Reading Ease	32.3	Automated Readability Index	17.1	Average words per sentence	22.2
Flesch-Kincaid Grade Level	14.3	Sentences	6	Average syllables per word	1.8

Lay Summary (Extended Prompt)

Prostate cancer is a major health concern, and scientists are still trying to understand whether drinking alcohol affects the risk of developing it. Some studies suggest that drinking large amounts of alcohol may increase the risk of aggressive prostate cancer, while others find little to no connection between moderate drinking and the disease. This study reviews many research articles to better understand how alcohol might play a role in prostate cancer risk. The researchers examined different types of studies and explored how the body processes alcohol. They found that heavy drinking may raise the risk of more dangerous prostate cancer, but the overall relationship remains unclear. These findings highlight the need for more research to guide public health advice. Understanding how alcohol affects prostate cancer risk could help people make healthier choices and improve cancer prevention strategies.

**Assessment Category**	**Score**	**Assessment Category**	**Score**	**Assessment Category**	**Score**
Comprehension Score Rater 1	5	Gunning Fog Score	14.5	Words	137
Comprehension Score Rater 2	5	SMOG Index	10.5	Complex words	23
Reading Age (years)	19.5	Coleman Liau Index	17.2	Percentage of complex words	16.8
Flesch-Kincaid Reading Ease	43.1	Automated Readability Index	14.7	Average words per sentence	19.6
Flesch-Kincaid Grade Level	12.1	Sentences	7	Average syllables per word	1.7

Manuscript 45

Gonnet P, Marinari E, Achard V, Schaffar R, Neyroud-Caspar I, May A, et al. Brain Metastases from Genito-Urinary Cancers in the Canton of Geneva (Switzerland): Study of Incidence, Management and Outcomes. Cancers [Internet]. 2024 Oct 10;16(20):3437. Available from: http://dx.doi.org/10.3390/cancers16203437

Abstract

Background: Incidence of brain metastases is precisely unknown and there is no clear consensus on their management. We aimed to determine the incidence of brain metastases among patients with genito-urinary primaries, present patients' characteristics and identify prognostic factors.

Method: We identified 51 patients treated in Geneva University Hospitals between January 1992 and December 2019. We retrospectively correlated their overall survival with 23 variables. We repeated a multivariate analysis with significant variables.

Results: Overall incidence of Brain Metastases (BMs) among Genito-Urinary (GU) patients is estimated to be 1.76% (range per primary GU tumour type: 0.00-6.65%). BMs originate from germ cell tumours in two cases (3.92%), from urothelial cell carcinoma in 15 cases (29.41%), from prostate cancer in 13 cases (25.49%), and from renal cell carcinoma in 21 cases (41.18%); there are no BMs from penile cancer in our cohort. The median age at BM diagnosis is 67 years old (range: 25-92). Most patients (54%) have a stage IV disease at initial diagnosis and 11 patients (22%) have BM at initial diagnosis. Only six patients (12%) are asymptomatic at BM diagnosis. The median Overall Survival (OS) from BM diagnosis is 3 months (range: 0-127). Five patients (10%) are long survivors (OS > 24 months). OS is significantly influenced by patient performance status and administration of systemic treatment. In the absence of meningeal carcinomatosis, OS is influenced by systemic treatment and

stereotactic radiosurgery. We also apply the Graded Prognostic Assessment (GPA) score to our cohort and note significant differences between groups.

Conclusion: Brain metastases from solid tumours is not a uniform disease, with a prognosis varying a lot among patients. The optimal management for patients with genito-urinary malignancies with brain metastases remain unclear and further research is needed.

Keywords:

brain metastases, chemotherapy, genito-urinary tumours, incidence, prognostic factors, survival

<u>Original Lay Summary</u>

Incidence of brain metastases from genito-urinary cancers has increased over the last years due to improved imaging techniques and better survival outcomes for patients even at an advanced metastatic stage thanks to therapeutic advances. However, no clear consensus exists on their management, and every case ought to be discussed in a multidisciplinary panel. We perform a single-centre retrospective study to report on incidence, patient demographics, clinicopathological characteristics, and treatment modalities. We also manage to identify predictive factors of outcome.

**Assessment Category**	**Score**	**Assessment Category**	**Score**	**Assessment Category**	**Score**
Comprehension Score Rater 1	4	Gunning Fog Score	18.5	Words	81
Comprehension Score Rater 2	3	SMOG Index	13.5	Complex words	22
Reading Age (years)	22.5	Coleman Liau Index	19.5	Percentage of complex words	27.2
Flesch-Kincaid Reading Ease	12.9	Automated Readability Index	16.9	Average words per sentence	20.3
Flesch-Kincaid Grade Level	16.5	Sentences	4	Average syllables per word	2.1

<u>Lay Summary (Simple Prompt)</u>

Brain metastases—cancer that spreads to the brain—can develop from genito-urinary cancers, such as kidney, bladder, and prostate cancer, but their exact frequency and best treatment approaches remain unclear. This study analyzed data from 51 patients treated in Geneva over nearly three decades to determine how often brain metastases occur, patient characteristics, and factors influencing survival. The results show that brain metastases are rare in these cancers, with survival varying widely among patients. While some patients live for years, the median survival after diagnosis is only three months. Factors such as overall health, systemic treatments, and targeted brain therapies influence survival outcomes. These findings highlight the need for better treatment strategies and further research to improve care for patients with brain metastases from genito-urinary cancers. Understanding these factors may help doctors provide more personalized treatment and improve patient outcomes.

**Assessment Category**	**Score**	**Assessment Category**	**Score**	**Assessment Category**	**Score**
Comprehension Score Rater 1	5	Gunning Fog Score	16.0	Words	140
Comprehension Score Rater 2	5	SMOG Index	11.8	Complex words	29
Reading Age (years)	21.5	Coleman Liau Index	19.3	Percentage of complex words	20.7
Flesch-Kincaid Reading Ease	30.6	Automated Readability Index	16.6	Average words per sentence	20.0
Flesch-Kincaid Grade Level	14.0	Sentences	7	Average syllables per word	1.8

Lay Summary (Extended Prompt)

Brain metastases, or cancer spread to the brain, are rare in patients with genito-urinary cancers, but their impact can be severe. This study examines how often brain metastases occur in these cancers, how they are treated, and what factors influence survival. Researchers analyzed data from 51 patients treated over nearly three decades to identify patterns in disease progression and treatment outcomes. The findings suggest that survival varies widely based on a patient's overall health and the treatments they receive, such as systemic therapy and targeted radiation. However, there is no clear standard for managing these cases. Understanding these patterns can help doctors make better treatment decisions and improve patient care. This study highlights the need for further research to develop more effective treatment strategies for patients with genito-urinary cancers that spread to the brain.

**Assessment Category**	**Score**	**Assessment Category**	**Score**	**Assessment Category**	**Score**
Comprehension Score Rater 1	5	Gunning Fog Score	15.1	Words	136
Comprehension Score Rater 2	5	SMOG Index	11.0	Complex words	25
Reading Age (years)	19.5	Coleman Liau Index	16.8	Percentage of complex words	18.4
Flesch-Kincaid Reading Ease	41.6	Automated Readability Index	14.3	Average words per sentence	19.4
Flesch-Kincaid Grade Level	12.3	Sentences	7	Average syllables per word	1.7

Manuscript 46

Steike DR, Troschel FM, Roers J, Siats JJ, Kittel C, Pepper NB, et al. Effect of Prior Transurethral Prostate Resection (TURP) or Laser Enucleation (ThuLEP) on Radiotherapy-Induced Toxicity and Quality of Life in Prostate Cancer Patients Undergoing Definitive Radiotherapy. Cancers [Internet]. 2024 Oct 6;16(19):3403. Available from: http://dx.doi.org/10.3390/cancers16193403

Abstract

In our study, the post-radiotherapy quality of life of prostate cancer patients who previously underwent transurethral resection of the prostate (TURP) is compared to those who had thulium laser enucleation of the prostate (ThuLEP) and those who had no prior surgery. It also aims to identify and assess risk factors affecting therapy tolerance in this patient group. We analyzed 132 patients with localized prostate cancer treated with definitive radiotherapy (RT), including 23 who had prior TURP and 19 who previously underwent ThuLEP. A total of 62% of patients underwent irradiation within 12 months after surgery. We included only patients treated with radiotherapy using the IMRT technique. Changes in patient-reported urinary toxicity were evaluated using the International Prostate Syndrome Score (IPSS) and the quality of life index of the World Health Organization (QoL/WHO-PSS) over a three-year post-radiotherapy period. Patients with prior TURP experienced significant deterioration in QoL and IPSS immediately after irradiation ($p < 0.001$), whereas those without previous surgery showed both less significant differences in IPSS and QoL scores. In conclusion, patients with previous TURP/ThuLEP differ from those without previous surgery in urinary quality of life and acute and chronic urinary symptom profiles after RT. The surgical technique (ThuLEP vs. TURP) and the time interval to irradiation are crucial factors affecting RT tolerance in acute and late settings. The previously operated patient group reported a significantly longer period of increased symptom burden.

Keywords:

IMRT, IPSS, TURP, ThuLEP, prostate cancer, quality of life, radio-oncology, radiotherapy of the prostate, thulium laser enucleation of the prostate, transurethral resection of the prostate

Original Lay Summary

In the radio-oncological treatment of patients with prostate cancer, there are significant differences in therapy tolerance due to risk factors, previous illnesses, and pre-treatment of the prostate. Patients with previous prostate surgery (TURP or ThuLEP) are a significant risk group in prostate cancer treatment and differ from non-operated patients in terms of quality of life. Patients who underwent transurethral prostate resection (TURP) exhibited greater overall quality of life impairment immediately after radiotherapy compared to those who had transurethral laser enucleation (ThuLEP) or no surgery. Operation time and radiation technique are important factors regarding therapy tolerance.

**Assessment Category**	**Score**	**Assessment Category**	**Score**	**Assessment Category**	**Score**
Comprehension Score Rater 1	5	Gunning Fog Score	22.9	Words	98
Comprehension Score Rater 2	5	SMOG Index	16.3	Complex words	32
Reading Age (years)	24.0	Coleman Liau Index	19.6	Percentage of complex words	32.7
Flesch-Kincaid Reading Ease	8.5	Automated Readability Index	19.2	Average words per sentence	24.5
Flesch-Kincaid Grade Level	18.2	Sentences	4	Average syllables per word	2.1

<u>Lay Summary (Simple Prompt)</u>

Prostate cancer patients sometimes undergo surgery to relieve urinary symptoms before receiving radiation therapy. This study examines how two common procedures—transurethral resection of the prostate (TURP) and thulium laser enucleation (ThuLEP)—affect side effects and quality of life after radiation therapy. Researchers analyzed 132 patients, comparing those who had prior surgery to those who did not. They found that patients with previous TURP experienced more severe urinary symptoms and a decline in quality of life after radiation, especially in the short term. The type of surgery and the time between surgery and radiation also influenced how well patients tolerated treatment. These findings help doctors better predict side effects and guide treatment decisions, ensuring that patients receive the best possible care while minimizing discomfort. Understanding these risks can improve patient counseling and lead to more personalized treatment strategies.

**Assessment Category**	**Score**	**Assessment Category**	**Score**	**Assessment Category**	**Score**
Comprehension Score Rater 1	4	Gunning Fog Score	18.6	Words	137
Comprehension Score Rater 2	4	SMOG Index	13.3	Complex words	37
Reading Age (years)	21.5	Coleman Liau Index	19.2	Percentage of complex words	27.0
Flesch-Kincaid Reading Ease	23.3	Automated Readability Index	16.3	Average words per sentence	19.6
Flesch-Kincaid Grade Level	14.9	Sentences	7	Average syllables per word	1.9

<u>Lay Summary (Extended Prompt)</u>

Prostate cancer is often treated with radiotherapy, but some men have already had surgery to ease urination problems before their cancer treatment. This study looked at how two types of prostate surgeries—Transurethral Resection of the Prostate (TURP) and Thulium Laser Enucleation of the Prostate (ThuLEP)—affect side effects and quality of life after radiotherapy. Researchers followed 132 patients, comparing those who had prior surgery to those who did not. They found that men who had TURP experienced more urinary problems and a drop in quality of life after radiotherapy, while those with no surgery had fewer issues. The time between surgery and radiotherapy also played a role in side effects. These findings help doctors better understand how past surgeries impact cancer treatment, allowing for better patient care and decision-making when planning radiotherapy for prostate cancer.

**Assessment Category**	**Score**	**Assessment Category**	**Score**	**Assessment Category**	**Score**
Comprehension Score Rater 1	4	Gunning Fog Score	15.2	Words	136
Comprehension Score Rater 2	4	SMOG Index	11.6	Complex words	24
Reading Age (years)	20.5	Coleman Liau Index	16.2	Percentage of complex words	17.7
Flesch-Kincaid Reading Ease	31.4	Automated Readability Index	15.5	Average words per sentence	22.7
Flesch-Kincaid Grade Level	14.5	Sentences	6	Average syllables per word	1.8

Manuscript 47

Ilie G, Knapp G, Davidson A, Snow S, Dahn HM, MacDonald C, et al. The Cancer Patient Empowerment Program: A Comprehensive Approach to Reducing Psychological Distress in Cancer Survivors, with Insights from a Mixed-Model Analysis, Including Implications for Breast Cancer Patients. Cancers [Internet]. 2024 Oct 2;16(19):3373. Available from: http://dx.doi.org/10.3390/cancers16193373

Abstract

Background/objectives: Psychological distress is a significant concern among cancer patients, negatively affecting their quality of life and adherence to treatment. The Cancer Patient Empowerment Program (CancerPEP) was developed as a comprehensive, home-based intervention aimed at reducing psychological distress by incorporating physical activity, dietary guidance, and social support. This study aimed to evaluate the feasibility, accrual and attrition rates, safety, and effectiveness of the CancerPEP intervention, with and without the biofeedback device, on psychological distress from baseline to 6 months, specifically focusing on the effects of group randomization and the difference between pre- and post-intervention results.

Methods: This single-site, crossover randomized clinical trial included 104 cancer patients who were randomized to receive the CancerPEP intervention, with or without a Heart Rate Variability (HRV) biofeedback monitor. At 6 months, participants who did not receive the device were allowed to use one until the end of the year, while those who did receive the device were followed up to 12 months. Randomization was stratified by the presence or absence of clinically significant psychological distress and metastatic status. Psychological distress was assessed using the Kessler Psychological Distress Scale (K10) at baseline, 6 months, and 12 months. The primary endpoint was the presence of nonspecific psychological distress, as measured by the K10 scale at 6 months from the trial start, based on group randomization. A secondary exploratory analysis assessed psychological distress at

baseline, 6 months, and 12 months for both groups, while controlling for group randomization and prognostic covariates. Prognostic covariates included age; comorbidities; time between diagnosis and randomization; treatment modality; relationship status; and use of prescribed medications for anxiety, depression, or both. An exploratory sub-analysis was conducted for the breast cancer subgroup, based on the sample size available after recruitment. The trial is registered at ClinicalTrials.gov (NCT05508412).

Results: The provision of the HRV biofeedback monitor in conjunction with the CancerPEP intervention did not significantly affect the primary outcome in either the full sample or the breast cancer subgroup, indicating that the HRV biofeedback provision was not beneficial in this trial. No self-reported or otherwise discovered adverse events at the 6-month mark were observed. About 10% of participants were lost to follow-up in both the early and late HRV monitor provision groups. Participation in the CancerPEP program led to a significant reduction in psychological distress over time. The odds of psychological distress were significantly higher at the start of the trial than at the end of the intervention (aOR = 2.64, 95% CI: 1.53-4.56) or 6 months after the intervention (aOR = 2.94, 95% CI: 1.62-5.30). Similarly, in the breast cancer subgroup, distress was higher at the trial's start than at 6 months, i.e., after the intervention (aOR = 2.25, 95% CI: 1.24-4.08), or at the end of the trial at 12 months (aOR = 2.73, 95% CI: 1.35-5.52).

Conclusions: CancerPEP significantly reduces psychological distress in cancer patients, with consistent improvements noted across various cancer types and stages, including benefits specifically for breast cancer patients. These findings build upon the success of the Prostate Cancer Patient Empowerment Program (PC-PEP), indicating that a similar comprehensive intervention can be advantageous for all cancer patients and may be further tailored to address specific needs. With its holistic approach-encompassing physical, dietary, and psychosocial support-

CancerPEP shows promise as a vital component of survivorship care. Ongoing 24-month evaluations will yield critical data on its long-term benefits. Additionally, a randomized trial with a control group (usual care without intervention) for breast cancer patients is currently under way and could potentially guide the integration of CancerPEP into standard oncology care to enhance patient outcomes and quality of life.

Keywords:

CancerPEP, breast cancer, cancer survivorship, mental health, multifaceted intervention, patient empowerment, psychological distress, randomized clinical trial

Original Lay Summary

Cancer patients frequently encounter significant emotional and psychological challenges that can adversely affect their overall well-being and treatment outcomes. To address these challenges, we developed the Cancer Patient Empowerment Program (CancerPEP), a comprehensive, home-based intervention that incorporates physical exercise, nutritional guidance, and social support. CancerPEP builds on the success of the Prostate Cancer Patient Empowerment Program (PC-PEP), which has demonstrated positive effects on psychological well-being in prostate cancer patients. In this study, we also evaluated whether adding a Heart Rate Variability (HRV) biofeedback device could enhance the effectiveness of CancerPEP. Our findings indicate that while CancerPEP significantly reduced psychological distress and improved emotional well-being across cancer patients—including a breast cancer subgroup—at the end of the intervention (the 6-month point), the addition or lack of an HRV device did not significantly contribute to these improvements for the full sample. These results suggest that CancerPEP, similar to PC-PEP, is a valuable addition to standard cancer care, providing a holistic approach to support patients both mentally and emotionally. However, the inclusion of the HRV device may not be necessary to achieve these benefits.

**Assessment Category**	**Score**	**Assessment Category**	**Score**	**Assessment Category**	**Score**
Comprehension Score Rater 1	5	Gunning Fog Score	19.9	Words	187
Comprehension Score Rater 2	5	SMOG Index	15.4	Complex words	50
Reading Age (years)	24.0	Coleman Liau Index	19.3	Percentage of complex words	26.7
Flesch-Kincaid Reading Ease	12.8	Automated Readability Index	20.0	Average words per sentence	26.7

Flesch-Kincaid Grade Level	18.1	Sentences	7	Average syllables per word	2.0

Lay Summary (Simple Prompt)

The Cancer Patient Empowerment Program (CancerPEP) was designed to help cancer survivors manage psychological distress by combining physical activity, healthy eating, and social support. This study tested the program's effectiveness in reducing distress over six months, with some participants also using a heart rate variability (HRV) biofeedback device. A total of 104 cancer patients participated, including a subgroup of breast cancer patients. The results showed that while the HRV device did not provide additional benefits, the CancerPEP program itself significantly reduced psychological distress over time. Improvements were consistent across different cancer types and stages. These findings suggest that CancerPEP could be a valuable tool in survivorship care, helping patients cope with emotional challenges after treatment. Future research will assess the program's long-term benefits and explore its potential integration into routine cancer care to improve patient well-being and quality of life.

**Assessment Category**	**Score**	**Assessment Category**	**Score**	**Assessment Category**	**Score**
Comprehension Score Rater 1	5	Gunning Fog Score	15.4	Words	142
Comprehension Score Rater 2	5	SMOG Index	12.0	Complex words	30
Reading Age (years)	21.5	Coleman Liau Index	19.4	Percentage of complex words	21.1
Flesch-Kincaid Reading Ease	23.0	Automated Readability Index	16.8	Average words per sentence	20.3
Flesch-Kincaid Grade Level	15.1	Sentences	7	Average syllables per word	1.9

Lay Summary (Extended Prompt)

The Cancer Patient Empowerment Program (CancerPEP) was created to help cancer survivors feel better emotionally by combining exercise, healthy eating, and social support. This study tested how well CancerPEP worked in reducing stress and anxiety in 104 cancer patients, including those with breast cancer. Some participants also used a heart rate monitor to see if it made a difference. After six months, results showed that the program helped lower psychological distress, but the heart rate monitor did not add extra benefits. No serious side effects were reported, and most participants stayed in the study. These findings suggest that CancerPEP can be a valuable tool to support cancer patients, helping them cope better with emotional challenges. More research is ongoing to see its long-term effects. If proven effective, CancerPEP could become a key part of cancer care, improving the quality of life for many survivors.

**Assessment Category**	**Score**	**Assessment Category**	**Score**	**Assessment Category**	**Score**
Comprehension Score Rater 1	5	Gunning Fog Score	13.6	Words	145
Comprehension Score Rater 2	5	SMOG Index	10.8	Complex words	28
Reading Age (years)	18.5	Coleman Liau Index	15.1	Percentage of complex words	19.3
Flesch-Kincaid Reading Ease	44.3	Automated Readability Index	12.4	Average words per sentence	18.1
Flesch-Kincaid Grade Level	11.6	Sentences	8	Average syllables per word	1.7

Manuscript 48

Maes J, Gesquière S, Maes A, Sathekge M, Van de Wiele C. Prostate-Specific Membrane Antigen-Positron Emission Tomography-Guided Radiomics and Machine Learning in Prostate Carcinoma. Cancers [Internet]. 2024 Oct 1;16(19):3369. Available from: http://dx.doi.org/10.3390/cancers16193369

Abstract

Positron emission tomography (PET) using radiolabeled prostate-specific membrane antigen targeting PET-imaging agents has been increasingly used over the past decade for imaging and directing prostate carcinoma treatment. Here, we summarize the available literature data on radiomics and machine learning using these imaging agents in prostate carcinoma. Gleason scores derived from biopsy and after resection are discordant in a large number of prostate carcinoma patients. Available studies suggest that radiomics and machine learning applied to PSMA-radioligand avid primary prostate carcinoma might be better performing than biopsy-based Gleason-scoring and could serve as an alternative for non-invasive GS characterization. Furthermore, it may allow for the prediction of biochemical recurrence with a net benefit for clinical utilization. Machine learning based on PET/CT radiomics features was also shown to be able to differentiate benign from malignant increased tracer uptake on PSMA-targeting radioligand PET/CT examinations, thus paving the way for a fully automated image reading in nuclear medicine. As for prediction to treatment outcome following 177Lu-PSMA therapy and overall survival, a limited number of studies have reported promising results on radiomics and machine learning applied to PSMA-targeting radioligand PET/CT images for this purpose. Its added value to clinical parameters warrants further exploration in larger datasets of patients.

Keywords:

PSMA, prostate carcinoma, radiomics

<u>Original Lay Summary</u>

Available studies suggest that radiomics and machine learning applied to PSMA-radioligand avid primary prostate carcinoma have potential to serve as an alternative for non-invasive Gleason score characterization, for the prediction of biochemical recurrence and to differentiate benign from malignant increased tracer uptake. However, prior to their implementation in clinical practice, additional, clinically relevant studies performed according to recently published guidelines and checklists, offering full transparency, including large enough datasets as well as external validation, are mandatory.

**Assessment Category**	**Score**	**Assessment Category**	**Score**	**Assessment Category**	**Score**
Comprehension Score Rater 1	4	Gunning Fog Score	30.5	Words	80
Comprehension Score Rater 2	4	SMOG Index	22.6	Complex words	31
Reading Age (years)	24.0	Coleman Liau Index	23.1	Percentage of complex words	38.8
Flesch-Kincaid Reading Ease	-24.1	Automated Readability Index	29.7	Average words per sentence	40.0
Flesch-Kincaid Grade Level	26.6	Sentences	2	Average syllables per word	2.3

Lay Summary (Simple Prompt)

Prostate cancer is one of the most common cancers in men, and accurate diagnosis is essential for effective treatment. This study explores how advanced imaging techniques, combined with artificial intelligence, can improve the detection and assessment of prostate cancer. Using positron emission tomography (PET) scans that target a specific protein found in prostate cancer cells, researchers analyze imaging data with machine learning to predict cancer severity and treatment outcomes. Findings suggest that this approach may be more reliable than traditional biopsy-based methods, offering a non-invasive way to assess cancer aggressiveness and predict recurrence. Additionally, artificial intelligence could help distinguish between cancerous and non-cancerous tissue, leading to faster and more accurate diagnoses. These advancements have the potential to improve patient care by reducing the need for invasive procedures and enhancing treatment decisions, but further research with larger patient groups is needed to confirm these benefits.

**Assessment Category**	**Score**	**Assessment Category**	**Score**	**Assessment Category**	**Score**
Comprehension Score Rater 1	5	Gunning Fog Score	21.0	Words	147
Comprehension Score Rater 2	5	SMOG Index	15.0	Complex words	41
Reading Age (years)	23.5	Coleman Liau Index	19.6	Percentage of complex words	27.9
Flesch-Kincaid Reading Ease	17.9	Automated Readability Index	19.1	Average words per sentence	24.5
Flesch-Kincaid Grade Level	16.8	Sentences	6	Average syllables per word	1.9

Lay Summary (Extended Prompt)

Prostate cancer is a common disease, and doctors use imaging scans to help diagnose and treat it. A special type of scan called PSMA-PET helps detect prostate cancer more accurately. This study looks at how advanced computer techniques, like machine learning and radiomics, can improve the way doctors understand these scans. Researchers found that these methods may predict cancer severity better than traditional biopsies, which involve removing a small tissue sample. They may also help doctors predict if the cancer will return and even assist in automatically reading scans to tell cancerous and non-cancerous areas apart. If proven reliable in larger studies, these techniques could make diagnosing and treating prostate cancer faster, easier, and more accurate. This could lead to better care for patients while reducing the need for invasive procedures. Scientists continue to explore these tools to improve prostate cancer detection and treatment.

**Assessment Category**	**Score**	**Assessment Category**	**Score**	**Assessment Category**	**Score**
Comprehension Score Rater 1	5	Gunning Fog Score	14.4	Words	146
Comprehension Score Rater 2	5	SMOG Index	10.5	Complex words	26
Reading Age (years)	18.5	Coleman Liau Index	16.3	Percentage of complex words	17.8
Flesch-Kincaid Reading Ease	42.9	Automated Readability Index	13.4	Average words per sentence	18.3
Flesch-Kincaid Grade Level	11.8	Sentences	8	Average syllables per word	1.7

Manuscript 49

Leblanc K, Edwards SJ, Dranitsaris G, Leong DP, Carrier M, Malone S, et al. Drug Interactions between Androgen Receptor Axis-Targeted Therapies and Antithrombotic Therapies in Prostate Cancer: Delphi Consensus. Cancers [Internet]. 2024 Sep 29;16(19):3336. Available from: http://dx.doi.org/10.3390/cancers16193336

Abstract

Background/Objectives: Abiraterone acetate, apalutamide, darolutamide, and enzalutamide, which make up the androgen receptor axis-targeted therapies (ARATs) drug class, are commonly used in the management of prostate cancer. Many patients on ARATs also receive oral antithrombotic therapy (i.e., anticoagulants or antiplatelets). The concomitant use of ARATs and antithrombotic therapies creates the potential for clinically relevant drug-drug interactions, but the literature regarding the actual consequences of these interactions, and guidance for co-prescribing, is limited. We assembled a multidisciplinary panel of experts and provided them with clinical information derived from a comprehensive literature review regarding the drug-drug interactions between ARATs and antithrombotic therapies. Methods: A three-stage modified electronic Delphi process was used to gather and consolidate opinions from the panel. Each stage consisted of up to three rounds of voting to achieve consensus on which ARAT/antithrombotic therapy drug pairs warrant attention, the possible clinical consequences of drug-drug interactions, and suggested actions for management. Results: The panel achieved consensus to avoid 11 ARAT/antithrombotic therapy drug pairs and modify therapy for eight pairs. Assessments relied heavily on pharmacokinetic data and extrapolation from drug-drug interaction studies of similarly metabolized drugs. Conclusions: This e-Delphi process highlights the need for further research into the clinical impact of ARAT/antithrombotic drug interactions. Nonetheless, the suggested actions aim to provide clinicians with a practical framework for therapeutic decision making.

Keywords:

androgen receptor axis-target therapy, anticoagulant, drug–drug interactions, prostate cancer, thrombosis

<u>Original Lay Summary</u>

Prostate cancer is most commonly diagnosed in males after the age of 55 years. These patients are also at risk for cardiovascular disease and venous thromboembolism requiring antithrombotic therapy. Prostate cancer treatments, such as androgen receptor axis-targeted therapies (ARATs, i.e., abiraterone acetate, apalutamide, darolutamide, and enzalutamide), may interact with common antithrombotic medications like warfarin, clopidogrel, and the direct oral anticoagulants. However, the data detailing the clinical outcomes of patients treated with these combinations are limited. We undertook a comprehensive review of the literature and modified Delphi process to enable development of an evidence-based consensus document for the co-prescribing of ARATs with antithrombotic medications. Our assessments relied heavily on pharmacokinetic data and extrapolation from drug interaction studies of similarly metabolized drugs, highlighting the need for more research into the clinical impact of drug interactions in prostate cancer patients. Nonetheless, we provide a practical framework to support clinicians in day-to-day therapeutic decision making.

**Assessment Category**	**Score**	**Assessment Category**	**Score**	**Assessment Category**	**Score**
Comprehension Score Rater 1	5	Gunning Fog Score	21.0	Words	157
Comprehension Score Rater 2	5	SMOG Index	14.2	Complex words	55
Reading Age (years)	23.5	Coleman Liau Index	20.6	Percentage of complex words	35.0
Flesch-Kincaid Reading Ease	4.8	Automated Readability Index	16.4	Average words per sentence	17.4

Flesch-Kincaid Grade Level	16.9	Sentences	9	Average syllables per word	2.2

<u>Lay Summary (Simple Prompt)</u>

Prostate cancer is often treated with a group of medications called androgen receptor axis-targeted therapies (ARATs). Many patients receiving these treatments also take blood thinners (antithrombotic therapies) to prevent blood clots. However, using these medications together may lead to harmful drug interactions, but there is little guidance on how to manage them. To address this, a team of experts reviewed available research and reached a consensus on which drug combinations should be avoided or adjusted. Through a structured process, they identified 11 ARAT and blood thinner combinations to avoid and recommended modifications for eight others. While more research is needed to understand the real-world effects of these interactions, the study provides valuable guidance for doctors to make safer treatment decisions for patients with prostate cancer who require both types of medication.

**Assessment Category**	**Score**	**Assessment Category**	**Score**	**Assessment Category**	**Score**
Comprehension Score Rater 1	5	Gunning Fog Score	16.7	Words	133
Comprehension Score Rater 2	5	SMOG Index	12.0	Complex words	26
Reading Age (years)	20.5	Coleman Liau Index	17.4	Percentage of complex words	19.6
Flesch-Kincaid Reading Ease	32.9	Automated Readability Index	16.2	Average words per sentence	22.2
Flesch-Kincaid Grade Level	14.2	Sentences	6	Average syllables per word	1.8

<u>Lay Summary (Extended Prompt)</u>

Prostate cancer is often treated with medications called androgen receptor axis-targeted therapies (ARATs). Many patients taking these drugs also need blood thinners, like anticoagulants or antiplatelets, to prevent blood clots. However, these medications can interact in ways that may cause serious health risks, but there is little clear guidance on how to manage this. To address this issue, a group of medical experts reviewed existing research and reached a consensus using a structured voting process. They identified which drug combinations should be avoided and which require adjustments. Their findings highlight the need for more studies but offer practical advice to help doctors safely prescribe these medications together. This research aims to improve treatment decisions for prostate cancer patients, reducing risks while ensuring they receive the best possible care.

**Assessment Category**	**Score**	**Assessment Category**	**Score**	**Assessment Category**	**Score**
Comprehension Score Rater 1	5	Gunning Fog Score	15.1	Words	129
Comprehension Score Rater 2	5	SMOG Index	11.0	Complex words	25
Reading Age (years)	19.5	Coleman Liau Index	17.9	Percentage of complex words	19.4
Flesch-Kincaid Reading Ease	39.9	Automated Readability Index	14.8	Average words per sentence	18.4
Flesch-Kincaid Grade Level	12.3	Sentences	7	Average syllables per word	1.8

Manuscript 50

Mavingire N, Moore JC, Johnson JR, Dwead AM, Cropp CD, Mechref Y, et al. Revisiting HER2 in Prostate Cancer from an Inclusive Perspective: From Biomarkers to Omics. Cancers [Internet]. 2024 Sep 25;16(19):3262. Available from: http://dx.doi.org/10.3390/cancers16193262

Abstract

Human epidermal growth factor receptor 2 (HER2) is a major driver of disease progression, treatment resistance, and worse survival for patients with various types of cancers, including prostate cancer. However, key bench studies and clinical trials have failed to evaluate the role of HER2 in prostate cancer using racially diverse experimental designs and protocols. This lack of diversity represents what has been the status quo of cancer research in the United States for decades. In the case of prostate cancer, homogenic study designs are problematic as Black men are much more likely to be diagnosed and die from aggressive and incurable forms of the disease. Therefore, the strategic inclusion of biospecimens collected from Black patients as well as the recruitment and enrollment of Black men into prostate cancer clinical trials is necessary to comprehensively evaluate genetic and molecular factors that contribute to variable outcomes in this high-risk population. Additionally, a higher prevalence of HER2 expression in Black men was recently reported in a small cohort of prostate cancer patients and may contribute to worsened prognosis. In this review, we carefully consider the role of HER2 in prostate cancer while, for the first time, taking into account the influences of race and genetic ancestry.

Keywords:

Black men, ERBB2, HER2, genetic ancestry, omics, prostate cancer

<u>Original Lay Summary</u>

HER2 is a well-known driver of worse outcomes for many types of cancer, including prostate cancer. While HER2 has been previously evaluated in prostate cancer bench studies and clinical trials, there has been a lack of diversity in the experimental designs and protocols. For this reason, it has only recently been reported that Black men with prostate cancer may have a higher prevalence of HER2 overexpression. To thoroughly address health inequities that exist for Black men with prostate cancer, it is critical to utilize diverse biospecimens and enroll diverse study participants into studies that evaluate genetic and molecular contributors to worse prognosis for high-risk populations. In this review, we reconsider the role of HER2 in prostate cancer with an approach that incorporates the effects of race and genetic ancestry.

**Assessment Category**	**Score**	**Assessment Category**	**Score**	**Assessment Category**	**Score**
Comprehension Score Rater 1	4	Gunning Fog Score	18.7	Words	131
Comprehension Score Rater 2	4	SMOG Index	13.4	Complex words	27
Reading Age (years)	21.5	Coleman Liau Index	15.3	Percentage of complex words	20.6
Flesch-Kincaid Reading Ease	29.8	Automated Readability Index	16.5	Average words per sentence	26.2
Flesch-Kincaid Grade Level	15.6	Sentences	5	Average syllables per word	1.8

<u>Lay Summary (Simple Prompt)</u>

Prostate cancer is a serious disease that affects men worldwide, but Black men are more likely to develop aggressive forms and have worse survival rates. A protein called HER2 has been linked to cancer growth and treatment resistance, but most studies on HER2 in prostate cancer have not included diverse patient groups. This review highlights the importance of studying HER2 in Black men with prostate cancer, as recent evidence suggests they may have higher levels of this protein, which could contribute to poor outcomes. By analyzing genetic and molecular factors using a more inclusive research approach, scientists can better understand how HER2 affects different populations. These findings could lead to improved treatments and more personalized care for high-risk patients, ultimately reducing racial disparities in prostate cancer outcomes.

**Assessment Category**	**Score**	**Assessment Category**	**Score**	**Assessment Category**	**Score**
Comprehension Score Rater 1	4	Gunning Fog Score	16.8	Words	128
Comprehension Score Rater 2	4	SMOG Index	11.9	Complex words	21
Reading Age (years)	20.5	Coleman Liau Index	16.8	Percentage of complex words	16.4
Flesch-Kincaid Reading Ease	38.7	Automated Readability Index	17.4	Average words per sentence	25.6
Flesch-Kincaid Grade Level	14.2	Sentences	5	Average syllables per word	1.7

Lay Summary (Extended Prompt)

Prostate cancer is a serious disease that affects many men, but Black men are more likely to develop aggressive forms and have worse outcomes. A protein called HER2 has been linked to cancer growth and treatment resistance, but most studies on prostate cancer have not considered racial differences. This study reviews what is known about HER2 in prostate cancer while highlighting the importance of including Black patients in research. Scientists recently found that Black men may have higher levels of HER2, which could explain some of the differences in survival rates. By focusing on HER2 and genetic factors, this research aims to improve our understanding of prostate cancer and help develop better treatments for all patients. Including diverse populations in studies is critical for making sure new treatments work for everyone, not just a select few.

**Assessment Category**	**Score**	**Assessment Category**	**Score**	**Assessment Category**	**Score**
Comprehension Score Rater 1	5	Gunning Fog Score	14.9	Words	136
Comprehension Score Rater 2	5	SMOG Index	10.6	Complex words	20
Reading Age (years)	18.5	Coleman Liau Index	14.9	Percentage of complex words	14.7
Flesch-Kincaid Reading Ease	49.5	Automated Readability Index	14.5	Average words per sentence	22.7
Flesch-Kincaid Grade Level	12.0	Sentences	6	Average syllables per word	1.6

Manuscript 51

Salemi S, Kranzbühler B, Baumgartner V, Breitenmoser L, Kuzmanov A, Lehner F, et al. Simultaneous Autophagy and Androgen Receptor Inhibition in a Prostate Cancer Xenograft Model. Cancers [Internet]. 2024 Sep 25;16(19):3261. Available from: http://dx.doi.org/10.3390/cancers16193261

Abstract

Objective: Abi, when used in conjunction with prednisone, is an established treatment for advanced PCa. Our goal was to explore the level of autophagy induced by Abi treatment, both alone and in combination with the autophagy inhibitor Chl, in a castrated mouse xenograft model.

Methods: LNCaP cells were injected into the left and right sides of the back of nude mice that had been previously castrated. Mice were divided into four groups and treated daily with intraperitoneal injections of vehicle (control), Abi (10 mg/kg), Abi (10 mg/kg) combined with Chl (10 mg/kg), or Chl (10 mg/kg), and were monitored for periods of 2 and 3 weeks.

Results: A significant reduction in tumor weight was observed in mice treated with the combination therapy, as opposed to those receiving vehicle control, Abi, or Chl alone. Mice receiving Abi + Chl exhibited reduced expression of ATG5, Beclin 1, and LC3 punctuations, along with an increase in P62, as determined by immunofluorescence and WES analysis. AR expression decreased significantly in all treatment groups compared to the control. PSMA expression was highest in the vehicle and combined treatment groups after 3 weeks, with a significant reduction observed with Chl treatment.

Conclusions: These findings demonstrate that Abi + Chl treatment lowers autophagy levels and suppresses tumors more effectively than Abi alone.

Keywords:

PSMA, abiraterone, autophagy, in vivo, prostate cancer

<u>Original Lay Summary</u>

Recent advancements in anticancer drug treatments face significant challenges due to drug resistance, often leading to only partial or short-term responses in patients. Our previous in vitro studies indicated that autophagy is linked to drug resistance in prostate cancer (PCa). Consequently, combination therapies targeting multiple pathways could help overcome resistance and extend treatment efficacy. To validate our earlier findings, we conducted in vivo experiments. Our data demonstrated that combining Abiraterone (Abi) with an autophagy inhibitor such as Chloroquine (Chl) not only reduces autophagy but also suppresses tumors more effectively than Abi alone. Thus, the combination of Abi with autophagy inhibitors represents a promising therapeutic strategy that could potentially offer a novel approach for patients.

**Assessment Category**	**Score**	**Assessment Category**	**Score**	**Assessment Category**	**Score**
Comprehension Score Rater 1	5	Gunning Fog Score	20.2	Words	115
Comprehension Score Rater 2	5	SMOG Index	14.5	Complex words	38
Reading Age (years)	23.5	Coleman Liau Index	19.9	Percentage of complex words	33.0
Flesch-Kincaid Reading Ease	10.1	Automated Readability Index	16.7	Average words per sentence	19.2
Flesch-Kincaid Grade Level	16.6	Sentences	6	Average syllables per word	2.1

<u>Lay Summary (Simple Prompt)</u>

Prostate cancer is often treated with a drug called abiraterone, but cancer cells can become resistant over time. One way they survive treatment is through a process called autophagy, where they recycle their own components to stay alive. This study explored whether blocking autophagy with a drug called chloroquine, alongside abiraterone, could make treatment more effective. Researchers tested this approach in mice with prostate cancer and found that the combination therapy significantly reduced tumor size compared to either drug alone. The treatment also lowered levels of key proteins linked to autophagy, suggesting that blocking this process may enhance the effectiveness of prostate cancer treatment. These findings could help develop better treatment strategies for advanced prostate cancer, potentially improving patient outcomes by preventing resistance to therapy.

**Assessment Category**	**Score**	**Assessment Category**	**Score**	**Assessment Category**	**Score**
Comprehension Score Rater 1	5	Gunning Fog Score	16.0	Words	125
Comprehension Score Rater 2	5	SMOG Index	11.6	Complex words	24
Reading Age (years)	21.5	Coleman Liau Index	19.2	Percentage of complex words	19.2
Flesch-Kincaid Reading Ease	31.4	Automated Readability Index	17.0	Average words per sentence	20.8
Flesch-Kincaid Grade Level	14.1	Sentences	6	Average syllables per word	1.8

Lay Summary (Extended Prompt)

Prostate cancer is a serious disease that affects many men worldwide. A common treatment, abiraterone (Abi), helps slow cancer growth, but researchers wanted to see if blocking a process called autophagy—where cancer cells recycle their parts to survive—could make the treatment work better. In this study, scientists used mice with prostate cancer and tested different treatments: Abi alone, an autophagy blocker (Chl) alone, both together, or no treatment. They found that the combination of Abi and Chl reduced tumor size more effectively than either treatment alone. The cancer cells also showed changes in important proteins related to tumor growth and survival. These findings suggest that stopping autophagy might make current prostate cancer treatments stronger. This research could help scientists develop better therapies for patients, potentially improving outcomes for those with advanced prostate cancer.

**Assessment Category**	**Score**	**Assessment Category**	**Score**	**Assessment Category**	**Score**
Comprehension Score Rater 1	5	Gunning Fog Score	13.3	Words	133
Comprehension Score Rater 2	5	SMOG Index	9.6	Complex words	19
Reading Age (years)	19.5	Coleman Liau Index	18.0	Percentage of complex words	14.3
Flesch-Kincaid Reading Ease	41.2	Automated Readability Index	15.1	Average words per sentence	19.0
Flesch-Kincaid Grade Level	12.2	Sentences	7	Average syllables per word	1.7

Manuscript 52

Pio F, Murdock A, Fuller RE, Whalen MJ. The Role of Whole-Gland and Focal Cryotherapy in Recurrent Prostate Cancer. Cancers [Internet]. 2024 Sep 22;16(18):3225. Available from: http://dx.doi.org/10.3390/cancers16183225

Abstract

Prostate cancer is the most common non-cutaneous malignancy in men, with the majority of newly diagnosed patients eligible for active surveillance. Despite definitive treatment, a considerable percentage of men will experience biochemical recurrence and even regional and distant metastatic recurrence after radiation therapy or radical prostatectomy. Salvage prostatectomy, while oncologically effective, poses significant morbidity with poor functional outcomes. Salvage cryotherapy has emerged as a promising alternative for localized recurrence, demonstrating safety and efficacy. This review examines the oncologic and functional outcomes of whole-gland and focal salvage cryotherapy, including disease-free survival, cancer-specific survival, and overall survival. The crucial role of multiparametric prostate MRI and evolving role of next-generation PSMA-targeted PET imaging are also examined. The comparison of outcomes of cryotherapy to other salvage ablation modalities, such as high-intensity focused ultrasound (HIFU), is also explored.

Keywords:

biochemical recurrence, cryoablation, prostate cancer, radiation therapy failure, salvage HIFU, salvage cryotherapy, salvage therapy

<u>Original Lay Summary</u>

The majority of newly diagnosed prostate cancer patients are eligible for active surveillance, and those at moderate to high risk frequently receive definitive treatments such as radiotherapy or radical prostatectomy. A considerable proportion of patients will experience biochemical recurrence, as well as localized and distant metastatic recurrence following definitive treatment. Salvage cryotherapy has emerged as a safe and effective alternative for treating localized recurrence. This review examines the oncologic and functional outcomes of whole-gland and focal salvage cryotherapy, as well as the crucial role of multiparametric prostate MRI and PSMA-targeted PET imaging. The outcomes of cryotherapy compared to those of other salvage ablation techniques like high-intensity focused ultrasound (HIFU) are also explored.

**Assessment Category**	**Score**	**Assessment Category**	**Score**	**Assessment Category**	**Score**
Comprehension Score Rater 1	4	Gunning Fog Score	22.1	Words	115
Comprehension Score Rater 2	3	SMOG Index	15.6	Complex words	37
Reading Age (years)	24.0	Coleman Liau Index	20.3	Percentage of complex words	32.2
Flesch-Kincaid Reading Ease	10.6	Automated Readability Index	18.9	Average words per sentence	23.0
Flesch-Kincaid Grade Level	17.5	Sentences	5	Average syllables per word	2.0

Lay Summary (Simple Prompt)

Prostate cancer is a common disease in men, and while many cases can be monitored without immediate treatment, some patients will experience cancer recurrence even after initial treatments like surgery or radiation. Traditional treatment options for recurrent prostate cancer, such as salvage prostatectomy, can be effective but often lead to serious side effects. This study explores an alternative approach—salvage cryotherapy—which uses extreme cold to destroy cancerous tissue while preserving as much healthy tissue as possible. Researchers compare the effectiveness and safety of whole-gland and focal cryotherapy in treating recurrent prostate cancer and assess their impact on survival and disease control. Advanced imaging techniques, such as multiparametric MRI and PSMA-targeted PET scans, are also examined for their role in guiding treatment decisions. By evaluating cryotherapy against other salvage treatments like high-intensity focused ultrasound (HIFU), this study aims to improve treatment options and outcomes for patients facing recurrent prostate cancer.

**Assessment Category**	**Score**	**Assessment Category**	**Score**	**Assessment Category**	**Score**
Comprehension Score Rater 1	5	Gunning Fog Score	19.3	Words	151
Comprehension Score Rater 2	4	SMOG Index	13.9	Complex words	35
Reading Age (years)	23.5	Coleman Liau Index	19.6	Percentage of complex words	23.2
Flesch-Kincaid Reading Ease	19.4	Automated Readability Index	19.5	Average words per sentence	25.2
Flesch-Kincaid Grade Level	16.8	Sentences	6	Average syllables per word	1.9

<u>Lay Summary (Extended Prompt)</u>

While many cases can be monitored without immediate treatment, some men need surgery or radiation. Unfortunately, cancer can return even after these treatments. Traditional surgery to remove the prostate again can be risky and cause serious side effects. This study looks at a less invasive option called cryotherapy, which freezes and destroys cancer cells. Researchers explore two types: whole-gland cryotherapy, which treats the entire prostate, and focal cryotherapy, which targets only the affected area. They also examine how imaging techniques, such as MRI and new PET scans, help improve treatment. By comparing cryotherapy to other approaches, like high-intensity focused ultrasound (HIFU), this study aims to find the best way to treat recurrent prostate cancer with fewer risks. These findings could help doctors choose safer, more effective treatments for patients whose cancer has returned.

**Assessment Category**	**Score**	**Assessment Category**	**Score**	**Assessment Category**	**Score**
Comprehension Score Rater 1	5	Gunning Fog Score	13.6	Words	135
Comprehension Score Rater 2	4	SMOG Index	9.9	Complex words	23
Reading Age (years)	18.5	Coleman Liau Index	16.8	Percentage of complex words	17.0
Flesch-Kincaid Reading Ease	43.7	Automated Readability Index	13.1	Average words per sentence	16.9
Flesch-Kincaid Grade Level	11.4	Sentences	8	Average syllables per word	1.7

Manuscript 53

Vecchiotti D, Clementi L, Cornacchia E, Di Vito Nolfi M, Verzella D, Capece D, et al. Evidence of the Link between Stroma Remodeling and Prostate Cancer Prognosis. Cancers [Internet]. 2024 Sep 21;16(18):3215. Available from: http://dx.doi.org/10.3390/cancers16183215

Abstract

Prostate cancer (PCa), the most commonly diagnosed cancer in men worldwide, is particularly challenging for oncologists when a precise prognosis needs to be established. Indeed, the entire clinical management in PCa has important drawbacks, generating an intense debate concerning the possibility to individuate molecular biomarkers able to avoid overtreatment in patients with pathological indolent cancers. To date, the paradigmatic change in the view of cancer pathogenesis prompts to look for prognostic biomarkers not only in cancer epithelial cells but also in the tumor microenvironment. PCa ecology has been defined with increasing details in the last few years, and a number of promising key markers associated with the reactive stroma are now available. Here, we provide an updated description of the most biologically significant and cited prognosis-oriented microenvironment biomarkers derived from the main reactive processes during PCa pathogenesis: tissue adaptations, inflammatory response and metabolic reprogramming. Proposed biomarkers include factors involved in stromal cell differentiation, cancer-normal cell crosstalk, angiogenesis, extracellular matrix remodeling and energy metabolism.

Keywords:

cancer-associated fibroblasts, energy metabolism, extracellular matrix, inflammation, myofibroblasts, reactive stroma

<u>Original Lay Summary</u>

Prostate cancer (PCa) is among the most common cancers. While PCa is frequently diagnosed in elderly men as a slow-growing, low-risk disease, in about 10–15% of cases, it can become a life-threatening danger. Unfortunately, biomarkers able to discriminate indolent prostatic tumors from aggressive forms are lacking, and watchful waiting remains one of the best options. However, available data support the hypothesis that distinctive biological characteristics of PCa stroma can contribute to cancer progression in a clinically relevant way. According to the current view, tissue alterations induced by tumor growth affect both metabolism of resident normal cells and composition of the extracellular matrix and are able also to recruit cells from circulation. Here, we seek to respond to the challenge of identifying stroma-associated biomarkers that may be relevant to assist prognostic decisions in PCa patients.

**Assessment Category**	**Score**	**Assessment Category**	**Score**	**Assessment Category**	**Score**
Comprehension Score Rater 1	5	Gunning Fog Score	18.8	Words	138
Comprehension Score Rater 2	5	SMOG Index	13.5	Complex words	33
Reading Age (years)	21.5	Coleman Liau Index	16.5	Percentage of complex words	23.9
Flesch-Kincaid Reading Ease	23.5	Automated Readability Index	15.9	Average words per sentence	23.0
Flesch-Kincaid Grade Level	15.7	Sentences	6	Average syllables per word	1.9

<u>Lay Summary (Simple Prompt)</u>

Prostate cancer is one of the most common cancers in men, but predicting how it will progress remains a challenge. Many patients receive unnecessary treatments, which can have serious side effects. Scientists are now looking beyond cancer cells and focusing on the surrounding tissue, known as the tumor microenvironment, to find better ways to assess prognosis. This study reviews key biological markers in the tumor's supporting structure, particularly in areas affected by inflammation, tissue changes, and energy use. These markers help scientists understand how cancer interacts with its surroundings and may improve predictions about which tumors are likely to grow aggressively. By identifying these biomarkers, researchers hope to develop more precise tools for distinguishing high-risk patients from those with slow-growing tumors, reducing overtreatment and improving patient care. This research could lead to more effective and personalized approaches to managing prostate cancer.

**Assessment Category**	**Score**	**Assessment Category**	**Score**	**Assessment Category**	**Score**
Comprehension Score Rater 1	5	Gunning Fog Score	16.3	Words	143
Comprehension Score Rater 2	5	SMOG Index	11.8	Complex words	29
Reading Age (years)	20.5	Coleman Liau Index	18.5	Percentage of complex words	20.3
Flesch-Kincaid Reading Ease	33.5	Automated Readability Index	16.2	Average words per sentence	20.4
Flesch-Kincaid Grade Level	13.7	Sentences	7	Average syllables per word	1.8

Lay Summary (Extended Prompt)

Prostate cancer is the most common cancer in men, but predicting its progression remains a major challenge. Some patients have slow-growing tumors that do not require aggressive treatment, while others have more aggressive forms that need early intervention. This study explores how changes in the tumor's surrounding tissue, known as the stroma, could help predict prostate cancer outcomes. Researchers are focusing on key biological markers related to tissue changes, inflammation, and metabolism, which may improve the accuracy of prognosis. By identifying these markers, doctors may be able to distinguish between aggressive and non-aggressive cases, reducing unnecessary treatments and improving patient care. These findings could lead to a new approach in prostate cancer research, shifting the focus from cancer cells alone to the broader tumor environment, ultimately enhancing how this disease is diagnosed and managed.

**Assessment Category**	**Score**	**Assessment Category**	**Score**	**Assessment Category**	**Score**
Comprehension Score Rater 1	5	Gunning Fog Score	16.4	Words	136
Comprehension Score Rater 2	5	SMOG Index	11.8	Complex words	25
Reading Age (years)	20.5	Coleman Liau Index	17.8	Percentage of complex words	18.4
Flesch-Kincaid Reading Ease	32.7	Automated Readability Index	16.7	Average words per sentence	22.7
Flesch-Kincaid Grade Level	14.3	Sentences	6	Average syllables per word	1.8

Manuscript 54

Iwamoto H, Hori T, Nakagawa R, Kano H, Makino T, Naito R, et al. Novel Treatment Strategies for Low-Risk Metastatic Castration-Sensitive Prostate Cancer. Cancers [Internet]. 2024 Sep 19;16(18):3198. Available from: http://dx.doi.org/10.3390/cancers16183198

Abstract

Background: The treatment strategy for metastatic castration-sensitive prostate cancer (mCSPC) has changed significantly in recent years. Based on various guidelines, an upfront androgen receptor signaling inhibitor (ARSI) is the first choice, but in patients of Asian descent, including Japanese patients, there are a certain number of cases in which androgen deprivation therapy (ADT) and CAB are more effective. If patients can be identified who show a marked response to ADT within 12 weeks after the initiation of ADT, which is the inclusion criterion for ARSI clinical trials targeting mCSPC, it would be valuable from an economic standpoint.

Methods: A total of 218 patients with pure prostate adenocarcinoma and treated with ADT at the Kanazawa University Hospital between January 2000 and December 2020 were included in this study. As a risk classification for mCSPC, in addition to the LATITUDE and CHAARTED criteria, we used the castration-sensitive prostate cancer classification proposed by Kanazawa University (Canazawa), developed by the Department of Urology of Kanazawa University. The Canazawa classification was based on three factors: Gleason pattern 5, bone scan index (BSI) ≥ 1.5, and lactate dehydrogenase (LDH) ≥ 300 IU/L. It defined patients with one factor or less as low-risk and patients with two or three factors as high-risk. The overall survival (OS) and time to castration resistance (TTCR) were estimated retrospectively using the Kaplan-Meier method, and factors associated with TTCR were identified using univariate and multivariate analyses.

Results: The median follow-up period was 40.4 months, the median OS period was 85.2 months, and the median TTCR period was 16.4 months. The Canazawa risk classification provided the clearest distinction between the OS and TTCR in mCSPC patients. Multivariate analysis revealed a decrease in PSA levels of <95% at 12 weeks after ADT initiation and was a predictor of short TTCR in low-risk, low-volume patients across all risk classifications.

Conclusion: The Canazawa classification differentiated the prognosis of mCSPC patients more clearly. A PSA reduction rate of <95% at 12 w after starting ADT in low-risk, low-volume patients of all risk classifications was significantly shorter than the TTCR. We propose a new treatment strategy, in which patients with low-risk mCSPC are treated with ADT and switched to ARSIs based on the rate of PSA reduction at 12 w.

<u>Keywords:</u>

PSA reduction rate, androgen deprivation therapy, combined androgen blockade, metastatic castration-sensitive prostate cancer, novel androgen receptor signaling inhibitor, prostate-specific antigen

Original Lay Summary

Upfront novel androgen receptor signaling inhibitors (ARSIs) are the first-line treatment for metastatic castration-sensitive prostate cancer (mCSPC). However, there are a certain number of cases in which androgen deprivation therapy (ADT) is more effective in patients of Asian descent. If we can identify patients who show a marked response to ADT within 12 weeks after ADT, which is the inclusion criterion for upfront ARSI clinical trials, it would be valuable from an economic standpoint. A total of 218 patients who received ADT treatment at Kanazawa University Hospital between 2000 and 2020 were included in this study. Multivariate analysis revealed that a decrease in PSA levels of <95% at 12 weeks after ADT initiation was a predictor of short time to castration resistance (TTCR) in low-risk patients. We propose a new treatment strategy, in which patients with low-risk mCSPC are treated with ADT and switched to ARSIs, based on the rate of PSA reduction at 12 weeks.

**Assessment Category**	**Score**	**Assessment Category**	**Score**	**Assessment Category**	**Score**
Comprehension Score Rater 1	5	Gunning Fog Score	16.8	Words	108
Comprehension Score Rater 2	4	SMOG Index	13.2	Complex words	26
Reading Age (years)	20.5	Coleman Liau Index	15.6	Percentage of complex words	24.1
Flesch-Kincaid Reading Ease	29.8	Automated Readability Index	13.7	Average words per sentence	21.6
Flesch-Kincaid Grade Level	14.5	Sentences	5	Average syllables per word	1.8

<u>Lay Summary (Simple Prompt)</u>

Prostate cancer that has spread but still responds to hormone therapy can be treated in different ways. While advanced hormone-blocking drugs are often recommended, some patients, especially those of Asian descent, respond well to standard hormone therapy alone. This study examined whether a patient's early response to treatment could help doctors decide the best approach. Researchers analyzed data from 218 patients and found that those whose prostate-specific antigen (PSA) levels dropped by less than 95% within 12 weeks were more likely to develop treatment resistance sooner. Using a new risk classification system, they suggest that low-risk patients start with standard hormone therapy and switch to advanced drugs only if their PSA response is slow. This strategy could improve patient outcomes while reducing unnecessary treatments and costs. These findings may help personalize prostate cancer treatment, making it more effective and affordable for patients worldwide.

**Assessment Category**	**Score**	**Assessment Category**	**Score**	**Assessment Category**	**Score**
Comprehension Score Rater 1	5	Gunning Fog Score	14.4	Words	146
Comprehension Score Rater 2	5	SMOG Index	10.5	Complex words	23
Reading Age (years)	19.5	Coleman Liau Index	17.0	Percentage of complex words	15.8
Flesch-Kincaid Reading Ease	43.1	Automated Readability Index	15.2	Average words per sentence	20.9
Flesch-Kincaid Grade Level	12.4	Sentences	7	Average syllables per word	1.7

Lay Summary (Extended Prompt)

Prostate cancer that has spread but still responds to hormone therapy can be treated in different ways. While advanced hormone-blocking drugs are often recommended, some patients, especially those of Asian descent, respond well to traditional hormone therapy alone. This study looked at whether doctors could predict which patients might do well with standard treatment by checking how much their prostate-specific antigen (PSA) levels dropped in the first 12 weeks. Researchers studied 218 patients and found that those whose PSA levels did not drop by at least 95% were more likely to develop treatment resistance sooner. Using a new risk classification system, they suggest that low-risk patients begin with standard hormone therapy and switch to stronger drugs only if their PSA response is not strong enough. This approach could help doctors provide better, more affordable treatment, reducing unnecessary medication while still controlling cancer effectively.

**Assessment Category**	**Score**	**Assessment Category**	**Score**	**Assessment Category**	**Score**
Comprehension Score Rater 1	5	Gunning Fog Score	15.2	Words	146
Comprehension Score Rater 2	5	SMOG Index	10.8	Complex words	21
Reading Age (years)	19.5	Coleman Liau Index	16.4	Percentage of complex words	14.4
Flesch-Kincaid Reading Ease	42.5	Automated Readability Index	16.5	Average words per sentence	24.3
Flesch-Kincaid Grade Level	13.4	Sentences	6	Average syllables per word	1.7

Manuscript 55

Kwon WA, Song Y, Lee MK. Strategic Advances in Combination Therapy for Metastatic Castration-Sensitive Prostate Cancer: Current Insights and Future Perspectives. Cancers [Internet]. 2024 Sep 18;16(18):3187. Available from: http://dx.doi.org/10.3390/cancers16183187

Abstract

The contemporary treatment for metastatic castration-sensitive prostate cancer (mCSPC) has evolved significantly, building on successes in managing metastatic castration-resistant prostate cancer (mCRPC). Although androgen deprivation therapy (ADT) alone has long been the cornerstone of mCSPC treatment, combination therapies have emerged as the new standard of care based on recent advances, offering improved survival outcomes. Landmark phase 3 trials demonstrated that adding chemotherapy (docetaxel) and androgen receptor pathway inhibitors to ADT significantly enhances overall survival, particularly for patients with high-volume, high-risk, or de novo metastatic disease. Despite these advancements, a concerning gap between evidence-based guidelines and real-world practice remains, with many patients not receiving recommended combination therapies. The challenge in optimizing therapy sequences, considering both disease control and treatment burdens, and identifying clinical and biological subgroups that could benefit from personalized treatment strategies persists. The advent of triplet therapy has shown promise in extending survival, but the uro-oncology community must narrow the gap between evidence and practice to deliver the most effective care. Current research is focused on refining treatment approaches and utilizing biomarkers to guide therapy selection, aiming to offer more personalized and adaptive strategies for mCSPC management. Thus, aligning clinical practices with the evolving evidence is urgently needed to improve outcomes for patients facing this incurable disease.

Keywords:

combination therapy, doublet therapy, metastatic castrate sensitive prostate cancer, prostate cancer, systemic treatment, triplet therapy

Original Lay Summary

This review explores the evolution of treatment strategies for metastatic castration-sensitive prostate cancer, emphasizing the benefits of early treatment intensification with androgen deprivation therapy, androgen receptor pathway inhibitors, and chemotherapy. Despite robust evidence and guideline recommendations, their real-world adoption remains low. The review discusses clinical trial findings, real-world data, and ongoing challenges, advocating for a personalized treatment approach based on disease characteristics and patient fitness to optimize outcomes and narrow the gap between clinical evidence and practice.

**Assessment Category**	**Score**	**Assessment Category**	**Score**	**Assessment Category**	**Score**
Comprehension Score Rater 1	4	Gunning Fog Score	24.7	Words	80
Comprehension Score Rater 2	4	SMOG Index	17.5	Complex words	28
Reading Age (years)	24.0	Coleman Liau Index	23.7	Percentage of complex words	35.0
Flesch-Kincaid Reading Ease	-6.4	Automated Readability Index	23.6	Average words per sentence	26.7
Flesch-Kincaid Grade Level	20.8	Sentences	3	Average syllables per word	2.2

<u>Lay Summary (Simple Prompt)</u>

Prostate cancer that has spread to other parts of the body is often treated with hormone therapy to slow its growth. However, research shows that using a combination of treatments, such as chemotherapy and targeted hormone therapies, can improve survival for many patients. While these combination therapies are now recommended, many patients are not receiving them due to differences between research findings and real-world practice. This study explores the latest advancements in combination treatments and highlights the need for more personalized approaches to improve outcomes. Researchers are also investigating biomarkers—biological indicators that can help doctors choose the best treatments for individual patients. By refining treatment strategies and ensuring patients receive the most effective care, this research aims to bridge the gap between scientific evidence and clinical practice, ultimately helping more people with advanced prostate cancer live longer and better lives.

**Assessment Category**	**Score**	**Assessment Category**	**Score**	**Assessment Category**	**Score**
Comprehension Score Rater 1	4	Gunning Fog Score	17.3	Words	141
Comprehension Score Rater 2	4	SMOG Index	12.5	Complex words	28
Reading Age (years)	21.5	Coleman Liau Index	19.2	Percentage of complex words	19.9
Flesch-Kincaid Reading Ease	28.8	Automated Readability Index	18.3	Average words per sentence	23.5
Flesch-Kincaid Grade Level	15.1	Sentences	6	Average syllables per word	1.8

Lay Summary (Extended Prompt)

Prostate cancer that has spread to other parts of the body is usually treated with hormone therapy, but new research shows that using a combination of treatments can help patients live longer. Scientists have tested adding chemotherapy or other hormone-blocking medicines to standard therapy, and the results are promising, especially for patients with aggressive cancer. However, many patients are not receiving these newer treatments, even though they have been proven to work better. Researchers are now trying to figure out the best ways to use these combination treatments and whether certain groups of patients might benefit more from specific therapies. They are also exploring the use of biomarkers—special signs in the body—to guide treatment choices. This study highlights the need to improve how doctors use the latest research to help patients receive the most effective care for advanced prostate cancer.

**Assessment Category**	**Score**	**Assessment Category**	**Score**	**Assessment Category**	**Score**
Comprehension Score Rater 1	4	Gunning Fog Score	15.1	Words	141
Comprehension Score Rater 2	4	SMOG Index	10.6	Complex words	20
Reading Age (years)	19.5	Coleman Liau Index	16.1	Percentage of complex words	14.2
Flesch-Kincaid Reading Ease	43.2	Automated Readability Index	15.8	Average words per sentence	23.5
Flesch-Kincaid Grade Level	13.1	Sentences	6	Average syllables per word	1.7

Manuscript 56

Choi SH, Beom SH, Choi YD, Ham WS, Han H, Han WK, et al. The Role of Local Prostate and Metastasis-Directed Radiotherapy in the Treatment of Oligometastatic Prostate Cancer. Cancers [Internet]. 2024 Sep 14;16(18):3159. Available from: http://dx.doi.org/10.3390/cancers16183159

<u>Abstract</u>

Background/Objectives: Oligometastatic prostate cancer (OMPC) represents an early stage of metastatic disease characterized by a limited number of lesions. Recent advancements in imaging and treatment have revived interest in personalized therapies, including metastasis-directed radiotherapy (OMDRT) and primary prostate radiotherapy (PPR). This study evaluates the impact of OMDRT timing and the role of PPR on survival outcomes in OMPC patients; Methods: In this retrospective cohort study, 82 patients with OMPC who underwent OMDRT between 2010 and 2019 were analyzed. Patients were classified based on OMDRT timing (early vs. late) and disease type (synchronous vs. metachronous). Progression-free survival (PFS) and overall survival (OS) were the primary endpoints, assessed via Kaplan-Meier analysis and Cox proportional hazards models; Results: Among the patients, 36 (43.9%) had synchronous and 46 (56.1%) had metachronous OMD. With a median follow-up of 32 months, the 5-year PFS and OS rates were 77.5% and 88.5%, respectively. Early OMDRT significantly improved PFS (HR 0.461, 95% CI: 0.257-0.826, p = 0.009) and OS (HR 0.219, 95% CI: 0.080-0.603, p = 0.003). Subgroup analysis showed the most favorable outcomes for synchronous OMD patients receiving early OMDRT, with a median PFS of 22.2 months and a 5-year survival rate of 42.1%. The treatment of the primary prostate provided a survival benefit in the OS of synchronous OMD patients (5-year 83.1% vs. 50%, p = 0.025), and there was a further improvement in OS after PPR (5-year 87.7% vs. 50%, p = 0.015). Conclusions: Early OMDRT significantly enhances survival outcomes in OMPC, in both synchronous and metachronous cases. The integration of PPR can further improve results, emphasizing the importance of early intervention and personalized

treatment strategies. To more definitively clarify our findings across various clinical situations, further studies with larger cohorts or prospective designs are necessary.

Keywords:

metastasis-directed radiotherapy, oligometastasis, prostate cancer, radiotherapy, survival outcomes

Original Lay Summary

This study investigates the optimal timing and effectiveness of radiotherapy in treating oligometastatic prostate cancer (OMPC), which is characterized by limited metastatic spread. We evaluated the impact of early intervention with radiotherapy, targeting both the primary tumor and metastatic sites, on patient survival. Our results show that early radiotherapy to oligometastatic sites, particularly when combined with primary tumor treatment, significantly enhances survival outcomes. These findings underscore the critical importance of timely and personalized treatment strategies in improving the management and care of patients with OMPC.

**Assessment Category**	**Score**	**Assessment Category**	**Score**	**Assessment Category**	**Score**
Comprehension Score Rater 1	4	Gunning Fog Score	21.7	Words	85
Comprehension Score Rater 2	4	SMOG Index	15.2	Complex words	28
Reading Age (years)	24.0	Coleman Liau Index	22.4	Percentage of complex words	32.9
Flesch-Kincaid Reading Ease	-6.8	Automated Readability Index	19.7	Average words per sentence	21.3
Flesch-Kincaid Grade Level	19.5	Sentences	4	Average syllables per word	2.3

<u>Lay Summary (Simple Prompt)</u>

Prostate cancer can spread to other parts of the body, but in some cases, the spread is limited, known as oligometastatic prostate cancer (OMPC). This study explores whether treating both the main tumor in the prostate and small metastatic sites with radiation therapy can improve survival. Researchers analyzed 82 patients who received targeted radiation and found that treating metastases early led to better long-term outcomes. Additionally, radiation to the prostate itself further improved survival, particularly for patients whose cancer had already spread at diagnosis. These findings suggest that early, personalized radiation therapy may help patients live longer and delay disease progression. More research is needed to confirm these benefits in larger groups, but this study highlights the potential of combining local and metastasis-directed treatment to improve care for men with OMPC.

**Assessment Category**	**Score**	**Assessment Category**	**Score**	**Assessment Category**	**Score**
Comprehension Score Rater 1	4	Gunning Fog Score	16.7	Words	133
Comprehension Score Rater 2	4	SMOG Index	12.0	Complex words	26
Reading Age (years)	20.5	Coleman Liau Index	17.4	Percentage of complex words	19.6
Flesch-Kincaid Reading Ease	30.4	Automated Readability Index	16.2	Average words per sentence	22.2
Flesch-Kincaid Grade Level	14.5	Sentences	6	Average syllables per word	1.8

<u>Lay Summary (Extended Prompt)</u>

Prostate cancer can sometimes spread to a few other areas in the body, a stage called oligometastatic prostate cancer. Researchers are exploring better treatment options for these patients. This study looked at how treating both the prostate and the small areas where cancer has spread with radiation therapy affects survival. The researchers analyzed data from 82 patients treated between 2010 and 2019. They found that starting radiation earlier helped patients live longer without cancer worsening. Patients whose cancer spread at the same time as their diagnosis had the best results when they received early radiation and prostate treatment. These findings suggest that using radiation therapy in a timely and targeted way may improve survival for these patients. More research is needed to confirm these results in larger groups, but this study supports a more personalized approach to treating prostate cancer that has only spread to a few places.

**Assessment Category**	**Score**	**Assessment Category**	**Score**	**Assessment Category**	**Score**
Comprehension Score Rater 1	5	Gunning Fog Score	12.5	Words	148
Comprehension Score Rater 2	5	SMOG Index	9.0	Complex words	19
Reading Age (years)	17.5	Coleman Liau Index	15.5	Percentage of complex words	12.8
Flesch-Kincaid Reading Ease	47.4	Automated Readability Index	12.9	Average words per sentence	18.5
Flesch-Kincaid Grade Level	11.2	Sentences	8	Average syllables per word	1.7

Manuscript 57

Abdollahi H, Fele-Paranj A, Rahmim A. Model-Informed Radiopharmaceutical Therapy Optimization: A Study on the Impact of PBPK Model Parameters on Physical, Biological, and Statistical Measures in 177Lu-PSMA Therapy. Cancers [Internet]. 2024 Sep 10;16(18):3120. Available from: http://dx.doi.org/10.3390/cancers16183120

Abstract

Purpose: To investigate the impact of physiologically based pharmacokinetic (PBPK) parameters on physical, biological, and statistical measures in lutetium-177-labeled radiopharmaceutical therapies (RPTs) targeting the prostate-specific membrane antigen (PSMA). Methods: Using a clinically validated PBPK model, realistic time-activity curves (TACs) for tumors, salivary glands, and kidneys were generated based on various model parameters. These TACs were used to calculate the area-under-the-TAC (AUC), dose, biologically effective dose (BED), and figure-of-merit BED (fBED). The effects of these parameters on radiobiological, pharmacokinetic, time, and statistical features were assessed. Results: Manipulating PBPK parameters significantly influenced AUC, dose, BED, and fBED outcomes across four different BED models. Higher association rates increased AUC, dose, and BED values for tumors, with minimal impact on non-target organs. Increased internalization rates reduced AUC and dose for tumors and kidneys. Higher serum protein-binding rates decreased AUC and dose for all tissues. Elevated tumor receptor density and ligand amounts enhanced uptake and effectiveness in tumors. Larger tumor volumes required dosimetry adjustments to maintain efficacy. Setting the tumor release rate to zero intensified the impact of association and internalization rates, enhancing tumor targeting while minimizing the effects on salivary glands and kidneys. Conclusions: Optimizing PBPK parameters can enhance the efficacy of lutetium-177-labeled RPTs targeting PSMA, providing insights for personalized and effective treatment regimens to minimize toxicity and improve therapeutic outcomes.

Keywords:

biologically effective dose, metastatic castration-resistant prostate cancer, physiologically based pharmacokinetic model, radiobiological response, radiopharmaceutical therapy

Original Lay Summary

This study investigates the influence of various pharmacokinetic parameters on 177Lu-PSMA therapies via physical, biological, and statistical measures. Employing a clinically validated physiologically based pharmacokinetic (PBPK) model, realistic time–activity curves (TACs) in tumors, salivary glands, and kidneys are generated, allowing for the calculation of metrics such as AUC, dose, BED, and fBED. The results demonstrate the significant impact of multiple parameters on the measured outcomes. In addition to the great impact of administered ligand amount, tumor volume, and receptor density, other pharmacokinetic parameters such as rates of radiopharmaceutical association, internalization, and release were identified as key influencers. Notably, alterations in the parameters induced distinct modifications in TAC features, affecting radiobiological, pharmacokinetic, and statistical aspects. These insights contribute to advancing personalized treatment regimens by understanding the key contributions of various parameters towards improving therapeutic efficacy while minimizing radiation-related toxicities.

**Assessment Category**	**Score**	**Assessment Category**	**Score**	**Assessment Category**	**Score**
Comprehension Score Rater 1	5	Gunning Fog Score	25.6	Words	140
Comprehension Score Rater 2	5	SMOG Index	17.7	Complex words	57
Reading Age (years)	24.0	Coleman Liau Index	24.1	Percentage of complex words	40.7
Flesch-Kincaid Reading Ease	-22.9	Automated Readability Index	22.2	Average words per sentence	23.3
Flesch-Kincaid Grade Level	22.3	Sentences	6	Average syllables per word	2.4

Lay Summary (Simple Prompt)

Radiopharmaceutical therapy (RPT) using lutetium-177 (^{77}Lu-PSMA) is a promising treatment for advanced prostate cancer. This study explores how different biological and physiological factors influence the effectiveness of this therapy. Using a computer model, researchers analyzed how changes in these factors affect drug absorption in tumors and healthy tissues, as well as the overall treatment impact. They found that adjusting specific parameters, such as how well the drug binds to cancer cells or how quickly it is taken up, can improve tumor targeting while reducing side effects on healthy organs. These findings provide valuable insights for optimizing RPT, helping doctors tailor treatments to individual patients. By fine-tuning these parameters, researchers aim to enhance treatment effectiveness, minimize harm to healthy tissues, and improve outcomes for patients with prostate cancer. This study contributes to the development of more precise and personalized cancer therapies.

**Assessment Category**	**Score**	**Assessment Category**	**Score**	**Assessment Category**	**Score**
Comprehension Score Rater 1	5	Gunning Fog Score	16.3	Words	143
Comprehension Score Rater 2	4	SMOG Index	12.0	Complex words	30
Reading Age (years)	20.5	Coleman Liau Index	18.2	Percentage of complex words	21.0
Flesch-Kincaid Reading Ease	26.4	Automated Readability Index	16.0	Average words per sentence	20.4
Flesch-Kincaid Grade Level	14.7	Sentences	7	Average syllables per word	1.9

<u>Lay Summary (Extended Prompt)</u>

Radiopharmaceutical therapy (RPT) is a treatment that uses tiny amounts of radioactive medicine to target cancer cells. This study explores how different biological and chemical factors affect the success of a specific RPT for prostate cancer. Using a detailed computer model, researchers tested how changes in these factors impact the treatment's effectiveness and safety. They found that adjusting certain properties, such as how well the medicine binds to cancer cells or how fast it spreads in the body, can improve tumor targeting while reducing harm to healthy tissues. The results suggest that fine-tuning these factors could lead to more personalized treatments, making RPT safer and more effective for patients. This research helps scientists and doctors understand how to optimize cancer therapies, potentially leading to better outcomes for people with advanced prostate cancer.

**Assessment Category**	**Score**	**Assessment Category**	**Score**	**Assessment Category**	**Score**
Comprehension Score Rater 1	5	Gunning Fog Score	15.5	Words	133
Comprehension Score Rater 2	4	SMOG Index	11.3	Complex words	23
Reading Age (years)	20.5	Coleman Liau Index	17.1	Percentage of complex words	17.3
Flesch-Kincaid Reading Ease	35.5	Automated Readability Index	16.0	Average words per sentence	22.2
Flesch-Kincaid Grade Level	13.8	Sentences	6	Average syllables per word	1.8

Manuscript 58

Zhang J, Chadha JS. Developmental Therapeutics in Metastatic Prostate Cancer: New Targets and New Strategies. Cancers [Internet]. 2024 Sep 6;16(17):3098. Available from: http://dx.doi.org/10.3390/cancers16173098

Abstract

There is an unmet need to develop new treatments for metastatic prostate cancer. With the development of targeted radioligand therapies, bispecific T cell engagers, antibody-drug conjugates and chimeric antigen receptor T cell (CAR T) therapies, tumor-associated cell surface antigens have emerged as new therapeutic targets in metastatic prostate cancer. Ongoing and completed clinical trials targeting prostate-specific membrane antigen (PSMA), six transmembrane epithelial antigens of the prostate 1 (STEAP1), kallikrein-related peptidase 2 (KLK2), prostate stem cell antigen (PSCA), and delta-like protein 3 (DLL3) in metastatic prostate cancer were reviewed. Strategies for sequential or combinational therapy were discussed.

Keywords:

CAR T, DLL3, KLK2, PSCA, PSMA, STEAP1, antibody–drug conjugate, bispecific T cell engagers, immunotherapy, prostate cancer

<u>Original Lay Summary</u>

The developments of radioligand therapy, immunotherapy, and targeted chemotherapy have led to dozens of clinical trials targeting tumor-associated surface antigens in metastatic prostate cancer. Emerging clinical data on the development of these new therapies were summarized and discussed.

**Assessment Category**	**Score**	**Assessment Category**	**Score**	**Assessment Category**	**Score**
Comprehension Score Rater 1	3	Gunning Fog Score	24.2	Words	39
Comprehension Score Rater 2	2	SMOG Index	16.3	Complex words	16
Reading Age (years)	24.0	Coleman Liau Index	21.6	Percentage of complex words	41.0
Flesch-Kincaid Reading Ease	-6.0	Automated Readability Index	18.3	Average words per sentence	19.5
Flesch-Kincaid Grade Level	18.9	Sentences	2	Average syllables per word	2.3

<u>Lay Summary (Simple Prompt)</u>

Metastatic prostate cancer remains challenging to treat, and new therapies are urgently needed. Scientists are exploring innovative treatments that target specific markers found on prostate cancer cells. These include advanced immunotherapies like CAR T-cell therapy, bispecific T-cell engagers, antibody-drug conjugates, and targeted radioligand treatments. This study reviews recent and ongoing clinical trials focusing on key prostate cancer targets such as PSMA, STEAP1, KLK2, PSCA, and DLL3. Researchers also discuss the potential benefits of combining different treatments or using them in sequence to improve outcomes. By identifying and evaluating these emerging strategies, this research aims to enhance the effectiveness of prostate cancer treatment. The findings could help guide future therapies, offering new hope to patients and advancing scientific knowledge in the fight against metastatic prostate cancer.

**Assessment Category**	**Score**	**Assessment Category**	**Score**	**Assessment Category**	**Score**
Comprehension Score Rater 1	5	Gunning Fog Score	17.3	Words	128
Comprehension Score Rater 2	5	SMOG Index	12.5	Complex words	33
Reading Age (years)	21.5	Coleman Liau Index	19.8	Percentage of complex words	25.8
Flesch-Kincaid Reading Ease	25.0	Automated Readability Index	16.2	Average words per sentence	18.3
Flesch-Kincaid Grade Level	14.3	Sentences	7	Average syllables per word	1.9

Lay Summary (Extended Prompt)

Metastatic prostate cancer is a serious disease that spreads beyond the prostate and is difficult to treat. Scientists are looking for new and better ways to fight it. This study explores promising new treatments that target specific markers on cancer cells. These treatments include special drugs that deliver radiation directly to cancer, therapies that help the immune system recognize and attack cancer, and advanced cell-based treatments like CAR T therapy. Researchers reviewed past and current clinical trials focusing on several key cancer markers, such as PSMA and DLL3. They also discussed ways to combine different treatments to improve their effectiveness. These new approaches could lead to better options for patients with metastatic prostate cancer, offering more hope for improved survival and quality of life. By advancing our understanding of these innovative therapies, this research helps guide future cancer treatments and improves care for patients facing this challenging disease.

**Assessment Category**	**Score**	**Assessment Category**	**Score**	**Assessment Category**	**Score**
Comprehension Score Rater 1	5	Gunning Fog Score	14.2	Words	149
Comprehension Score Rater 2	5	SMOG Index	10.5	Complex words	26
Reading Age (years)	19.5	Coleman Liau Index	17.4	Percentage of complex words	17.5
Flesch-Kincaid Reading Ease	43.1	Automated Readability Index	14.5	Average words per sentence	18.6
Flesch-Kincaid Grade Level	11.9	Sentences	8	Average syllables per word	1.7

Manuscript 59

Palmisano F, Lorusso V, Legnani R, Martorello V, Nedbal C, Tramanzoli P, et al. Analysis of the Performance and Accuracy of a PSA and PSA Ratio-Based Nomogram to Predict the Probability of Prostate Cancer in a Cohort of Patients with PIRADS 3 Findings at Multiparametric Magnetic Resonance Imaging. Cancers [Internet]. 2024 Sep 5;16(17):3084. Available from: http://dx.doi.org/10.3390/cancers16173084

Abstract

Background: PIRADS score 3 represents a challenge in prostate cancer (PCa) detection with MRI. Our study aimed to evaluate the application of a nomogram on a cohort of patients with PIRADS 3.

Methods: We analyzed 286 patients undergoing fusion prostate biopsy from January 2020 to February 2024. Only PIRADS 3 patients were included. Two nomograms, previously developed and based on clinical variables such as age, total PSA (specifically 2-10 ng/mL) and PSA ratio were applied to estimate the probability (Nomograms A and B) for PCa Grade Group (GG) > 3 and GG < 3.

Results: Out of the 70 patients available for analysis, 14/70 patients (20%) had PCa, 4/14 were GG 1 (28.6%), 1/14 were GG 2 (7.1%), 5/14 were GG 3 (35.8%), 2/14 were GG 4 (14.3%) and 2/14 were GG 5 (14.3%). The median probability of PCa GG > 3 and GG < 3 was 5% and 33%, respectively. A significant difference (p = 0.033) was found between patients with negative versus positive biopsy for Nomogram B. There was a significant difference (p = 0.029) for Nomogram B comparing patients with GG < 3 and GG > 3. Using a cut-off of 40% for Nomogram B, sensitivity and specificity were 70% and 80%, respectively.

Conclusions: This cohort has a low probability of harboring PCa especially ISUP > 3. Nomogram B has good accuracy for discriminating patients with PCa from those with negative biopsy.

Keywords:

MRI, PIRADS 3, nomogram, prostate cancer

<u>Original Lay Summary</u>

Prostate cancer (PCa) detection and risk stratification in patients with PIRADS 3 represents a challenge, since it depicts a clinical scenario in which the chance of unnecessary biopsy procedures and overdiagnosis is still high. For these reasons, in this study, we analyzed and evaluated the performance of a nomogram previously developed by our group and based on age, total PSA and PSA ratio to estimate the probability of harboring PCa and help with discriminating both PCa with ISUP < 3 and ISUP ≥ 3 in patients scheduled for prostate biopsy at our institution and with a recent finding of PIRADS 3 at multiparametric MRI (mpMRI).

**Assessment Category**	**Score**	**Assessment Category**	**Score**	**Assessment Category**	**Score**
Comprehension Score Rater 1	4	Gunning Fog Score	29.8	Words	105
Comprehension Score Rater 2	4	SMOG Index	19.5	Complex words	23
Reading Age (years)	24.0	Coleman Liau Index	13.1	Percentage of complex words	21.9
Flesch-Kincaid Reading Ease	2.9	Automated Readability Index	27.9	Average words per sentence	52.5
Flesch-Kincaid Grade Level	25.9	Sentences	2	Average syllables per word	1.8

<u>Lay Summary (Simple Prompt)</u>

Detecting prostate cancer in patients with PIRADS 3 findings on MRI is challenging because this category has an unclear risk of cancer. This study aimed to evaluate a mathematical tool, called a nomogram, which predicts the likelihood of prostate cancer based on clinical factors like age, PSA levels, and PSA ratio. Researchers analyzed 70 patients who underwent prostate biopsy, finding that 20% had prostate cancer, with varying levels of severity. One of the tested nomograms, Nomogram B, showed good accuracy in identifying patients with cancer and distinguishing between lower and higher-risk cases. With a specific cut-off value, it achieved 70% sensitivity and 80% specificity. These findings suggest that Nomogram B could help doctors better assess the need for biopsy in patients with PIRADS 3, potentially reducing unnecessary procedures while improving cancer detection.

**Assessment Category**	**Score**	**Assessment Category**	**Score**	**Assessment Category**	**Score**
Comprehension Score Rater 1	5	Gunning Fog Score	17.3	Words	134
Comprehension Score Rater 2	5	SMOG Index	13.1	Complex words	31
Reading Age (years)	20.5	Coleman Liau Index	15.8	Percentage of complex words	23.1
Flesch-Kincaid Reading Ease	27.6	Automated Readability Index	15.0	Average words per sentence	22.3
Flesch-Kincaid Grade Level	15.0	Sentences	6	Average syllables per word	1.9

Lay Summary (Extended Prompt)

Detecting prostate cancer can be challenging, especially in patients with a PIRADS 3 score on MRI, which means the risk of cancer is uncertain. This study tested a tool called a nomogram, which uses factors like age, PSA levels, and PSA ratio to predict cancer risk. Researchers analyzed data from 70 patients who had biopsies after their MRI. They found that 20% had prostate cancer, with most cases being low to moderate risk. One version of the nomogram (Nomogram B) showed good accuracy in identifying patients with cancer versus those without. It correctly predicted cancer risk in many cases, with 70% sensitivity and 80% specificity at a certain threshold. These findings suggest that the nomogram could help doctors make better decisions about whether a biopsy is needed, reducing unnecessary procedures for patients with a low risk of aggressive cancer.

**Assessment Category**	**Score**	**Assessment Category**	**Score**	**Assessment Category**	**Score**
Comprehension Score Rater 1	5	Gunning Fog Score	14.6	Words	139
Comprehension Score Rater 2	5	SMOG Index	11.0	Complex words	25
Reading Age (years)	18.5	Coleman Liau Index	13.6	Percentage of complex words	18.0
Flesch-Kincaid Reading Ease	44.9	Automated Readability Index	12.0	Average words per sentence	19.9
Flesch-Kincaid Grade Level	11.9	Sentences	7	Average syllables per word	1.7

Manuscript 60

Hameed MY, Gul M, Chaudhry A, Muzaffar H, Sheikh M, Chee W, et al. From Oncogenesis to Theranostics: The Transformative Role of PSMA in Prostate Cancer. Cancers [Internet]. 2024 Aug 31;16(17):3039. Available from: http://dx.doi.org/10.3390/cancers16173039

Abstract

Prostate cancer, a leading cause of cancer-related mortality among men, is characterized by complex genetic and epigenetic alterations, dysregulation of oncogenic pathways, and a dynamic tumor microenvironment. Advances in molecular diagnostics and targeted therapies have significantly transformed the management of this disease. Prostate-specific membrane antigen (PSMA) has emerged as a critical biomarker, enhancing the precision of prostate cancer diagnosis and treatment. Theranostics, which integrates PSMA-targeted imaging with radioligand therapies, has shown remarkable efficacy in detecting and treating advanced prostate cancer. By leveraging the dual capabilities of PSMA-based diagnostics and therapeutic agents, theranostics offers a personalized approach that improves patient outcomes. This comprehensive review explores the latest developments in PSMA-targeted theranostics and their impact on the future of prostate cancer management, highlighting key clinical trials and emerging therapeutic strategies.

Keywords:

Actinium 225, Lutetium (177Lu) vipivotide tetraxetan, Lutetium 177, Pluvicto, prostate cancer, prostate-specific membrane antigen (PSMA), theranostics

Original Lay Summary

Prostate cancer remainsa leading cause of cancer-related deaths in men worldwide. This review focuses on the transformative role of prostate-specific membrane antigen (PSMA) in the diagnosis and treatment of prostate cancer. PSMA is highly expressed in prostate cancer cells, making it a valuable target for both imaging and therapy. The integration of PSMA-targeted imaging with radioligand therapy, known as theranostics, has significantly improved the detection and management of advanced prostate cancer. Recent clinical trials, including the VISION trial, have demonstrated the effectiveness of PSMA-targeted therapies, such as 177Lu-PSMA-617. These advancements highlight the potential of theranostics in offering personalized and more effective treatment options for prostate cancer patients.

**Assessment Category**	**Score**	**Assessment Category**	**Score**	**Assessment Category**	**Score**
Comprehension Score Rater 1	5	Gunning Fog Score	18.2	Words	113
Comprehension Score Rater 2	5	SMOG Index	12.9	Complex words	30
Reading Age (years)	21.5	Coleman Liau Index	19.0	Percentage of complex words	26.6
Flesch-Kincaid Reading Ease	24.5	Automated Readability Index	15.8	Average words per sentence	18.8
Flesch-Kincaid Grade Level	14.5	Sentences	6	Average syllables per word	1.9

Lay Summary (Simple Prompt)

Prostate cancer is a major health concern for men worldwide, and finding better ways to detect and treat it is crucial. Scientists have discovered that a protein called prostate-specific membrane antigen (PSMA) plays a key role in prostate cancer, making it a valuable target for both diagnosis and treatment. A new approach called theranostics combines PSMA-targeted imaging to detect cancer with specialized treatments that deliver radiation directly to cancer cells. This personalized method helps doctors identify the disease more accurately and treat it more effectively, improving patient outcomes. In this study, the authors review the latest advancements in PSMA-based theranostics, including promising clinical trials and emerging therapies. Understanding these developments may lead to better tools for managing prostate cancer, ultimately improving survival and quality of life for patients.

**Assessment Category**	**Score**	**Assessment Category**	**Score**	**Assessment Category**	**Score**
Comprehension Score Rater 1	5	Gunning Fog Score	18.2	Words	131
Comprehension Score Rater 2	5	SMOG Index	13.1	Complex words	31
Reading Age (years)	21.5	Coleman Liau Index	18.3	Percentage of complex words	23.7
Flesch-Kincaid Reading Ease	27.1	Automated Readability Index	16.7	Average words per sentence	21.8
Flesch-Kincaid Grade Level	14.9	Sentences	6	Average syllables per word	1.9

Lay Summary (Extended Prompt)

Prostate cancer is a major health concern for men, and doctors are always looking for better ways to detect and treat it. One important discovery is a protein called prostate-specific membrane antigen (PSMA), which is found in high levels on prostate cancer cells. Scientists have developed special imaging tools and treatments that target PSMA, helping doctors see cancer more clearly and deliver precise therapies. This approach, called theranostics, combines diagnosis and treatment to improve patient care. By using radioactive drugs that attach to PSMA, doctors can find and treat cancer more effectively, even in advanced stages. This study explores how PSMA-based theranostics is changing prostate cancer treatment, leading to more personalized and effective care. Understanding these advances can help improve survival rates and quality of life for patients while shaping the future of cancer treatment.

**Assessment Category**	**Score**	**Assessment Category**	**Score**	**Assessment Category**	**Score**
Comprehension Score Rater 1	5	Gunning Fog Score	13.1	Words	137
Comprehension Score Rater 2	5	SMOG Index	9.3	Complex words	18
Reading Age (years)	18.5	Coleman Liau Index	16.8	Percentage of complex words	13.1
Flesch-Kincaid Reading Ease	44.9	Automated Readability Index	14.4	Average words per sentence	19.6
Flesch-Kincaid Grade Level	11.9	Sentences	7	Average syllables per word	1.7

Manuscript 61

Ladner LR, Adhikari S, Bhutada AS, Cuoco JA, Patel VM, Entwistle JJ, et al. Traditional Prostate Cancer Risk Assessment Scales Do Not Predict Outcomes from Brain Metastases: A Population-Based Predictive Nomogram. Cancers [Internet]. 2024 Aug 30;16(17):3029. Available from: http://dx.doi.org/10.3390/cancers16173029

Abstract

Brain metastases are an uncommon yet life-limiting manifestation of prostate cancer. However, there is limited insight into the natural progression, therapeutics, and patient outcomes for prostate cancer once metastasized to the brain. This is a retrospective study of 461 patients with metastatic prostate cancer to the brain with a primary outcome of median overall survival (OS). The Surveillance, Epidemiology, and End Results (SEER) database was examined using Cox regression univariate and multivariable analyses, and a corresponding nomogram was developed. The median overall survival was 15 months. In the multivariable analysis, Hispanic patients had significantly increased OS (median OS 17 months, p = 0.005). Patients with tumor sizes greater than three centimeters exhibited significantly reduced OS (median OS 19 months, p = 0.014). Patients with additional metastases to the liver exhibited significantly reduced OS (median OS 3.5 months, p p = 0.039), in addition to radiation and chemotherapy (median OS 25 months, p = 0.002). The nomogram had a C-index of 0.641. For patients with prostate metastases to the brain, median OS is influenced by race, tumor size, presence of additional metastases, and treatment. The lack of an association between traditional prostate cancer prognosis metrics, including Gleason and ISUP grading, and mortality highlights the need for individualized, metastasis-specific prognosis metrics. This prognostic nomogram for prostate metastases to the brain can be used to guide the management of affected patients.

Keywords:

brain metastases, nomogram, prognosis, prostate cancer

<u>Original Lay Summary</u>

Brain metastases from systemic cancer are the most common tumors of the central nervous system. For prostate metastases to the brain, the clinical progression is poorly understood. This retrospective study aims to elucidate clinical risk factors associated with overall survival (OS; months post-diagnosis) in prostate metastases to the brain, and then develop a nomogram to aid in clinical decision making for this vulnerable population. We identified several factors associated with survival, including race, tumor size, and the presence of additional metastases. This study should serve as a clinical framework for prognostication in metastatic prostate cancer to the brain.

**Assessment Category**	**Score**	**Assessment Category**	**Score**	**Assessment Category**	**Score**
Comprehension Score Rater 1	4	Gunning Fog Score	20.4	Words	99
Comprehension Score Rater 2	4	SMOG Index	14.3	Complex words	31
Reading Age (years)	22.5	Coleman Liau Index	17.9	Percentage of complex words	31.3
Flesch-Kincaid Reading Ease	21.8	Automated Readability Index	15.4	Average words per sentence	19.8
Flesch-Kincaid Grade Level	15.1	Sentences	5	Average syllables per word	2.0

Lay Summary (Simple Prompt)

Prostate cancer can sometimes spread to the brain, but little is known about how this affects patients' survival and treatment outcomes. This study analyzed data from 461 patients with brain metastases from prostate cancer to better understand survival patterns and key risk factors. Researchers developed a prediction tool (a nomogram) using various patient characteristics, such as race, tumor size, and additional metastases. They found that larger tumors and liver metastases were linked to shorter survival, while patients receiving radiation and chemotherapy lived longer. Surprisingly, traditional prostate cancer grading systems did not predict survival, suggesting that a new, metastasis-specific approach is needed. This study provides a tool that may help doctors estimate survival for these patients and make better treatment decisions. By improving our understanding of brain metastases in prostate cancer, this research may lead to more personalized care and better outcomes.

**Assessment Category**	**Score**	**Assessment Category**	**Score**	**Assessment Category**	**Score**
Comprehension Score Rater 1	4	Gunning Fog Score	17.1	Words	142
Comprehension Score Rater 2	4	SMOG Index	12.4	Complex words	32
Reading Age (years)	21.5	Coleman Liau Index	18.4	Percentage of complex words	22.5
Flesch-Kincaid Reading Ease	31.9	Automated Readability Index	16.1	Average words per sentence	20.3
Flesch-Kincaid Grade Level	13.8	Sentences	7	Average syllables per word	1.8

Lay Summary (Extended Prompt)

Prostate cancer can sometimes spread to the brain, but doctors do not fully understand how this affects patients or what treatments work best. This study looked at 461 patients with prostate cancer that had spread to the brain to find patterns in survival rates. Researchers used a large health database to study factors like tumor size, race, and additional cancer spread. They found that larger tumors and cancer spreading to the liver led to shorter survival, while treatments like chemotherapy and radiation helped patients live longer. Surprisingly, common prostate cancer grading systems did not predict survival in these cases. The researchers created a new tool, called a nomogram, to help doctors estimate survival for these patients. This study highlights the need for better ways to predict outcomes and guide treatment for prostate cancer that spreads to the brain, helping doctors make more informed decisions for their patients.

**Assessment Category**	**Score**	**Assessment Category**	**Score**	**Assessment Category**	**Score**
Comprehension Score Rater 1	4	Gunning Fog Score	12.8	Words	147
Comprehension Score Rater 2	4	SMOG Index	8.8	Complex words	16
Reading Age (years)	17.5	Coleman Liau Index	15.4	Percentage of complex words	10.9
Flesch-Kincaid Reading Ease	53.7	Automated Readability Index	14.0	Average words per sentence	21.0
Flesch-Kincaid Grade Level	11.0	Sentences	7	Average syllables per word	1.6

Manuscript 62

Leitão C, Estrela M, Monteiro L, Fardilha M, Herdeiro MT, Roque F. Health Professionals' Perceptions about Prostate Cancer—A Focus Group Study. Cancers [Internet]. 2024 Aug 29;16(17):3005. Available from: http://dx.doi.org/10.3390/cancers16173005

Abstract

Prostate cancer (PCa) accounts for 20% of new cancer cases and 10.5% of cancer-associated mortality in Portugal. Associated risk factors include advanced age, family history, genetic alterations, and race/ethnicity. However, the role of lifestyle factors is often underestimated. To explore health professionals' perceptions of PCa risk factors, a qualitative study with three focus groups (FG), with a total of twenty-one general practitioners and urologists, was conducted via videoconference between February and April 2023. Seven themes emerged, including general perceptions of PCa; PCa risk factors; nutritional impact; the role of physical activity; alcohol consumption and smoking; sexual activity and sexually transmitted diseases roles in PCa; and screening, diagnosis, and treatment methods. Despite agreeing that healthy lifestyles could promote better PCa outcomes and quality of life, participants did not specify any lifestyle factors that could promote or prevent this disease, posing challenges to lifestyle changes, particularly among older adults. Non-invasive screening methods, such as biomarkers and alternative treatments, are crucial for future research. This study underscores the need for further investigation into the correlation of lifestyle factors with PCa and highlights the necessity of health professionals in encouraging their patients to adopt healthier lifestyles, while offering important insights into awareness, prevention, and alternative screening, diagnosis, and treatment methods, which could help reduce false positives and treatment side effects.

Keywords:

attitudes, health professionals, knowledge, lifestyle, perceptions, prostate cancer, risk factors, screening/diagnosis, treatment

<u>Original Lay Summary</u>

Prostate cancer (PCa) is a major health issue among men, with risk factors such as age, family history, genetics, and race being well known. However, the impact of lifestyle choices has often been overlooked. This study aimed to understand health professionals' perceptions of PCa risk factors to design a questionnaire applicable to the male population. Three focus groups were conducted to discuss topics, such as the impact of diet, physical activity, alcohol consumption, smoking, sexual health, and screening and treatment methods. Although healthy lifestyles can generally improve PCa outcomes, no specific lifestyle change has been identified as a preventive measure, particularly for older adults. Lycopene, present in tomatoes, was highlighted for its antioxidant benefits. This study demonstrates the necessity for additional research on how lifestyle affects PCa and emphasizes the importance of health professionals in encouraging the adoption of healthier habits. It also suggests exploring non-invasive screening through urine and innovative treatment methods to reduce false positives and side effects, respectively.

**Assessment Category**	**Score**	**Assessment Category**	**Score**	**Assessment Category**	**Score**
Comprehension Score Rater 1	5	Gunning Fog Score	19.2	Words	162
Comprehension Score Rater 2	5	SMOG Index	13.7	Complex words	45
Reading Age (years)	21.5	Coleman Liau Index	18.5	Percentage of complex words	27.8
Flesch-Kincaid Reading Ease	25.4	Automated Readability Index	16.1	Average words per sentence	20.3
Flesch-Kincaid Grade Level	14.7	Sentences	8	Average syllables per word	1.9

<u>Lay Summary (Simple Prompt)</u>

Prostate cancer is a major health concern in Portugal, yet the role of lifestyle factors in its prevention and progression is often overlooked. This study explored the views of general practitioners and urologists on prostate cancer risk factors through online focus group discussions. Seven key themes emerged, including perceptions of risk factors, the impact of diet and exercise, alcohol and smoking, sexual health, and screening methods. While participants agreed that a healthy lifestyle could improve outcomes, they did not specify which lifestyle changes might help prevent prostate cancer. This gap highlights the need for more research on how daily habits influence the disease. Additionally, the study emphasizes the importance of non-invasive screening methods and alternative treatments to reduce false positives and treatment side effects. Encouraging health professionals to promote healthy lifestyles could play a crucial role in prevention and patient well-being.

**Assessment Category**	**Score**	**Assessment Category**	**Score**	**Assessment Category**	**Score**
Comprehension Score Rater 1	5	Gunning Fog Score	15.7	Words	143
Comprehension Score Rater 2	4	SMOG Index	11.6	Complex words	28
Reading Age (years)	20.5	Coleman Liau Index	18.1	Percentage of complex words	19.6
Flesch-Kincaid Reading Ease	38.8	Automated Readability Index	15.9	Average words per sentence	20.4
Flesch-Kincaid Grade Level	12.9	Sentences	7	Average syllables per word	1.7

<u>Lay Summary (Extended Prompt)</u>

Prostate cancer is a common disease that affects many men, but there is still uncertainty about how lifestyle choices influence its development. This study explored the views of doctors and specialists on prostate cancer risk factors through online group discussions. The researchers found that while health professionals agreed that a healthy lifestyle might improve outcomes, they did not identify specific habits that could help prevent the disease. The study also highlighted the need for better screening tests that are less invasive and more accurate. Understanding what doctors know and believe about prostate cancer can help improve awareness, encourage healthier lifestyles, and promote better screening and treatment options. These findings emphasize the importance of further research on lifestyle factors and prostate cancer, as well as the role of healthcare professionals in guiding their patients toward healthier choices.

**Assessment Category**	**Score**	**Assessment Category**	**Score**	**Assessment Category**	**Score**
Comprehension Score Rater 1	5	Gunning Fog Score	15.5	Words	136
Comprehension Score Rater 2	5	SMOG Index	11.1	Complex words	22
Reading Age (years)	20.5	Coleman Liau Index	18.4	Percentage of complex words	16.2
Flesch-Kincaid Reading Ease	37.6	Automated Readability Index	17.3	Average words per sentence	22.7
Flesch-Kincaid Grade Level	13.6	Sentences	6	Average syllables per word	1.7

Manuscript 63

Chavali JS, Pedraza AM, Soputro NA, Ramos-Carpinteyro R, Mikesell CD, Kaouk J. Single-Port Extraperitoneal vs. Multiport Transperitoneal Robot-Assisted Radical Prostatectomy: A Propensity Score-Matched Analysis. Cancers [Internet]. 2024 Aug 28;16(17):2994. Available from: http://dx.doi.org/10.3390/cancers16172994

Abstract

(1) Background: Since the introduction of the purpose-built Single Port (SP) robotic platform, there has been an ongoing debate regarding its advantages compared to the established multi-port (MP) system. The goal of this present study is to compare the perioperative, oncological, and functional outcomes of SP Extraperitoneal robotic radical prostatectomy (RARP) versus that of MP Transperitoneal RARP approach at a high-volume tertiary center. (2) Methods: Based on a retrospective review of the prospectively maintained IRB-approved database, 925 patients successfully underwent RARP by a single experienced robotic surgeon. A 4:1 propensity-matched analysis based on the baseline prostate cancer International Society of Urological Pathology (ISUP) Grade Group, clinical stage, and preoperative Prostate Specific Antigen (PSA) was performed, which yielded a cohort of 606 patients-485 in the SP EP and 121 in the MP TP approaches. Of note, the SP EP approach also included the traditional Extraperitoneal (n = 259, 53.4%) and the novel Transvesical (TV) approaches (n = 226, 46.6%). (3) Results: The overall operative time was slightly longer in the SP cohort, with a mean of 198.9 min compared to 181.5 min for the MP group (p p = 0.84). Regarding functional outcomes, the mean continence rates and Sexual Health Inventory for Men (SHIM) scores were identical between the two groups at 6 weeks, 3 months, and 6 months respectively. (4) Conclusion: SP EP RARP demonstrates similar performance to MP TP RARP in terms of oncologic and functional outcomes. However, SP EP RARP offers several advantages in reducing the overall hospital stay, decreasing postoperative pain and hence the overall opioid use, as well as shortening the time to catheter removal, all of which translates to reduced morbidity and facilitates the transition to outpatient surgery.

Keywords:

multi-port, prostate cancer, radical prostatectomy, robotic surgery, single-port

<u>Original Lay Summary</u>

Following its introduction, the innovative purpose-built Single Port (SP) robotic platform has been utilized for various urological surgical procedures, including for the management of clinically significant prostate cancer. This study sought to compare the different cancer-related and postoperative functional outcomes between the novel SP Extraperitoneal robotic radical prostatectomy (RARP) and the gold-standard Transperitoneal multi-port RARP approaches. The findings from this study can be beneficial to assist clinicians to determine best surgical approach based on the individual patient's characteristics to ensure the favorable outcomes following RARP.

**Assessment Category**	**Score**	**Assessment Category**	**Score**	**Assessment Category**	**Score**
Comprehension Score Rater 1	4	Gunning Fog Score	24.0	Words	89
Comprehension Score Rater 2	3	SMOG Index	18.2	Complex words	30
Reading Age (years)	24.0	Coleman Liau Index	21.6	Percentage of complex words	33.7
Flesch-Kincaid Reading Ease	-5.8	Automated Readability Index	23.3	Average words per sentence	29.7
Flesch-Kincaid Grade Level	21.4	Sentences	3	Average syllables per word	2.2

Lay Summary (Simple Prompt)

Prostate cancer is commonly treated with robotic-assisted surgery, but there is ongoing debate about the best approach. This study compares two techniques: a single-port (SP) extraperitoneal method, which uses a single small incision, and a traditional multi-port (MP) transperitoneal method, which requires multiple incisions. Researchers analyzed data from 606 patients who underwent surgery at a specialized center to compare outcomes such as cancer control, recovery time, and post-surgical function. The results showed that both techniques achieved similar cancer control and functional recovery. However, the SP approach led to shorter hospital stays, less pain, lower opioid use, and quicker catheter removal, making it a promising option for minimally invasive prostate cancer surgery. These findings suggest that SP surgery may improve patient recovery and support a shift toward outpatient prostate cancer treatment while maintaining effective outcomes.

**Assessment Category**	**Score**	**Assessment Category**	**Score**	**Assessment Category**	**Score**
Comprehension Score Rater 1	5	Gunning Fog Score	19.9	Words	138
Comprehension Score Rater 2	5	SMOG Index	14.3	Complex words	37
Reading Age (years)	22.5	Coleman Liau Index	18.4	Percentage of complex words	26.8
Flesch-Kincaid Reading Ease	19.8	Automated Readability Index	17.4	Average words per sentence	23.0
Flesch-Kincaid Grade Level	16.2	Sentences	6	Average syllables per word	1.9

Lay Summary (Extended Prompt)

Prostate cancer is commonly treated with robotic surgery to remove the prostate, but there are different surgical approaches. This study compares two methods: single-port (SP) surgery, which uses one small incision, and multi-port (MP) surgery, which requires multiple incisions. Researchers analyzed the outcomes of 606 patients who had either SP or MP surgery. They found that both methods were equally effective in removing cancer and restoring function. However, the SP approach had benefits such as shorter hospital stays, less pain, reduced need for pain medication, and faster recovery. These findings suggest that the SP method may improve patient comfort and allow for quicker return to normal activities. By providing evidence that SP surgery is as safe and effective as MP surgery while offering additional recovery benefits, this research can help guide doctors and patients in choosing the best surgical option for prostate cancer treatment.

**Assessment Category**	**Score**	**Assessment Category**	**Score**	**Assessment Category**	**Score**
Comprehension Score Rater 1	5	Gunning Fog Score	17.4	Words	146
Comprehension Score Rater 2	5	SMOG Index	12.5	Complex words	33
Reading Age (years)	20.5	Coleman Liau Index	15.9	Percentage of complex words	22.6
Flesch-Kincaid Reading Ease	37.3	Automated Readability Index	14.3	Average words per sentence	20.9
Flesch-Kincaid Grade Level	13.2	Sentences	7	Average syllables per word	1.8

Manuscript 64

Zhao K, Pang K, Hung AL, Zheng H, Yan R, Sung K. A Deep Learning-Based Framework for Highly Accelerated Prostate MR Dispersion Imaging. Cancers [Internet]. 2024 Aug 27;16(17):2983. Available from: http://dx.doi.org/10.3390/cancers16172983

Abstract

Dynamic contrast-enhanced magnetic resonance imaging (DCE-MRI) measures microvascular perfusion by capturing the temporal changes of an MRI contrast agent in a target tissue, and it provides valuable information for the diagnosis and prognosis of a wide range of tumors. Quantitative DCE-MRI analysis commonly relies on the nonlinear least square (NLLS) fitting of a pharmacokinetic (PK) model to concentration curves. However, the voxel-wise application of such nonlinear curve fitting is highly time-consuming. The arterial input function (AIF) needs to be utilized in quantitative DCE-MRI analysis. and in practice, a population-based arterial AIF is often used in PK modeling. The contribution of intravascular dispersion to the measured signal enhancement is assumed to be negligible. The MR dispersion imaging (MRDI) model was recently proposed to account for intravascular dispersion, enabling more accurate PK modeling. However, the complexity of the MRDI hinders its practical usability and makes quantitative PK modeling even more time-consuming. In this paper, we propose fast MR dispersion imaging (fMRDI) to effectively represent the intravascular dispersion and highly accelerated PK parameter estimation. We also propose a deep learning-based, two-stage framework to accelerate PK parameter estimation. We used a deep neural network (NN) to estimate PK parameters directly from enhancement curves. The estimation from NN was further refined using several steps of NLLS, which is significantly faster than performing NLLS from random initializations. A data synthesis module is proposed to generate synthetic training data for the NN. Two data-processing modules were introduced to improve the model's stability against noise and variations. Experiments on our in-house clinical prostate MRI dataset demonstrated that our method significantly reduces the processing time, produces a better

distinction between normal and clinically significant prostate cancer (csPCa) lesions, and is more robust against noise than conventional DCE-MRI analysis methods.

Keywords:

DCE-MRI, MRI, deep learning, dispersion imaging, prostate cancer, transformer

Original Lay Summary

Nonlinear curve fitting of the pharmacokinetic model to DCE-MRI concentration curves is highly time-consuming. The estimation of highly non-linear dispersion-related parameter in MR dispersion imaging (MRDI) makes the process even more tedious. The fast MRDI (fMRDI) model is proposed to simplify and accelerate the MRDI model by representing the dispersion-applied arterial input function (AIF) as the weighted-sum of a fast and a slow population-based AIFs. A deep learning-based two-stage inference method is proposed to accelerate quantitative MRDI. The deep learning model makes a initial estimation of the parameters directly from the concentration curves and the parameters is then refined by a number of iterative optimization.

**Assessment Category**	**Score**	**Assessment Category**	**Score**	**Assessment Category**	**Score**
Comprehension Score Rater 1	4	Gunning Fog Score	18.6	Words	114
Comprehension Score Rater 2	4	SMOG Index	13.6	Complex words	28
Reading Age (years)	21.5	Coleman Liau Index	15.8	Percentage of complex words	24.6
Flesch-Kincaid Reading Ease	26.4	Automated Readability Index	15.2	Average words per sentence	22.8
Flesch-Kincaid Grade Level	15.2	Sentences	5	Average syllables per word	1.9

Lay Summary (Simple Prompt)

Prostate cancer diagnosis often relies on MRI scans that track how a contrast agent moves through blood vessels. However, current methods for analyzing these images are slow and may overlook important details about how the contrast spreads. This study introduces a new deep learning-based approach to speed up the process while improving accuracy. The researchers developed a method called fast MR dispersion imaging (fMRDI) that accounts for how contrast disperses in blood vessels, leading to more precise tumor detection. By using artificial intelligence to estimate key parameters, their approach significantly reduces processing time and improves the distinction between normal and aggressive prostate cancer. This advancement could help doctors diagnose prostate cancer more quickly and accurately, leading to better treatment decisions. The research also provides a foundation for improving MRI analysis in other medical applications, making advanced imaging techniques more accessible and efficient.

**Assessment Category**	**Score**	**Assessment Category**	**Score**	**Assessment Category**	**Score**
Comprehension Score Rater 1	4	Gunning Fog Score	17.7	Words	143
Comprehension Score Rater 2	4	SMOG Index	12.7	Complex words	34
Reading Age (years)	21.5	Coleman Liau Index	19.2	Percentage of complex words	23.8
Flesch-Kincaid Reading Ease	28.7	Automated Readability Index	16.8	Average words per sentence	20.4
Flesch-Kincaid Grade Level	14.3	Sentences	7	Average syllables per word	1.9

<u>Lay Summary (Extended Prompt)</u>

Doctors use a special type of MRI scan called dynamic contrast-enhanced MRI (DCE-MRI) to help detect and understand prostate cancer. However, analyzing these scans takes a long time because it requires complex calculations. This study aims to speed up the process using artificial intelligence. The researchers created a deep learning-based method to quickly analyze MRI data while improving accuracy. Their approach also accounts for how the contrast agent moves through the blood, making the results more reliable. By combining a neural network with a faster way to refine the results, the new method provides better cancer detection while drastically reducing processing time. Tests on real prostate MRI scans showed that this technique helps doctors better identify dangerous tumors and works well even with noisy data. This breakthrough could make prostate cancer diagnosis faster and more precise, helping doctors and patients get better results.

**Assessment Category**	**Score**	**Assessment Category**	**Score**	**Assessment Category**	**Score**
Comprehension Score Rater 1	5	Gunning Fog Score	13.1	Words	146
Comprehension Score Rater 2	5	SMOG Index	9.4	Complex words	21
Reading Age (years)	18.5	Coleman Liau Index	16.2	Percentage of complex words	14.4
Flesch-Kincaid Reading Ease	45.8	Automated Readability Index	13.3	Average words per sentence	18.3
Flesch-Kincaid Grade Level	11.4	Sentences	8	Average syllables per word	1.7

Manuscript 65

Gottlieb J, Hanes DA, Bustos MA, Choe J, Luu A, Seizer D, et al. Impact of Cryopreserved Placental Allografts on Biochemical Recurrence in Prostate Cancer. Cancers [Internet]. 2024 Aug 26;16(17):2973. Available from: http://dx.doi.org/10.3390/cancers16172973

Abstract

Background: Human placental allografts are widely used to promote wound healing. Placental (or amniotic membrane/umbilical cord) allografts are placed along the neurovascular bundles during radical prostatectomy to improve continence and erectile function recovery. It is unknown whether placental allografts impact biochemical recurrence (BCR).

Methods: This was a single-surgeon retrospective study of 566 robotic radical prostatectomies performed from April 2015 to March 2021. The patients were divided into three groups: the negative control, Brand A, and Brand B. Brand A and Brand B were both cryopreserved amniotic membrane (CAM) allografts. A total of 324 cases were included for BCR Kaplan-Meier and risk-adjusted multivariate analyses (362 for continence analysis). In vitro analyses were performed to determine the effect of CAM allografts on prostate cancer (PCa) cell line growth.

Results: For propensity score-matched analysis (adjusting for pre-operative PSA, tumor stage, Gleason Grade, and margin status), (1) the allograft groups did not show differences in time to BCR vs. the negative control group (p = 0.7), and (2) combined allograft treatment groups showed better continence recovery vs. the negative controls (p = 0.01). In vitro, placental allografts reduced PCa cell line growth in co-culture assays.

Conclusions: cryopreserved AM allografts (combined or individual brands) did not show a significant effect on BCR but improved continence recovery for PCa patients.

Keywords:

BCR, biochemical recurrence-free survival, placental allograft, prostate cancer, prostatectomy

<u>Original Lay Summary</u>

Placental allograft tissue has been extensively used in wound healing. It can also be placed along the neurovascular bundles during radical prostatectomy to improve post-operative continence and erectile function recovery. The existing literature shows how placental allografts lead to improved functional recovery after radical prostatectomy. It is unknown whether these allografts have an impact on biochemical recurrence (BCR). Our study was a single surgeon retrospective review of 362 radical prostatectomy cases. The subgroups were the negative control group and two brands of cryopreserved amniotic membrane (CAM). Our data show improved continence recovery using CAM allografts. At a median follow-up of 41 months, there was no observed impact of BCR. Interestingly, in vitro analysis also revealed decreased cell viability of prostate cancer cell lines when incubated with the CAM allograft. Although further investigation is required, our data do not support a need for oncologic concern while using CAM allografts during radical prostatectomy.

**Assessment Category**	**Score**	**Assessment Category**	**Score**	**Assessment Category**	**Score**
Comprehension Score Rater 1	5	Gunning Fog Score	16.3	Words	153
Comprehension Score Rater 2	5	SMOG Index	11.6	Complex words	40
Reading Age (years)	20.5	Coleman Liau Index	18.1	Percentage of complex words	26.14
Flesch-Kincaid Reading Ease	24.3	Automated Readability Index	13.3	Average words per sentence	15.3
Flesch-Kincaid Grade Level	13.7	Sentences	10	Average syllables per word	1.97

Lay Summary (Simple Prompt)

Prostate cancer is often treated with surgery to remove the prostate, which can affect a man's ability to control urination and maintain sexual function. To help with recovery, doctors sometimes place tissue from donated human placentas, known as placental allografts, near the nerves during surgery. While this tissue helps with healing, it was unclear if it could affect the chance of cancer coming back. In this study, researchers looked at the records of 566 men who had prostate surgery, comparing those who received placental tissue with those who did not. They found that using this tissue did not increase the risk of the cancer returning. Interestingly, men who received the tissue recovered bladder control faster than those who did not. This means placental tissue can safely support better recovery after prostate surgery without raising the risk of cancer returning.

**Assessment Category**	**Score**	**Assessment Category**	**Score**	**Assessment Category**	**Score**
Comprehension Score Rater 1	5	Gunning Fog Score	14.3	Words	139
Comprehension Score Rater 2	5	SMOG Index	10.3	Complex words	22
Reading Age (years)	18.5	Coleman Liau Index	14.8	Percentage of complex words	15.83
Flesch-Kincaid Reading Ease	44.9	Automated Readability Index	13.0	Average words per sentence	19.86
Flesch-Kincaid Grade Level	11.9	Sentences	7	Average syllables per word	1.68

Lay Summary (Extended Prompt)

This study looked at whether using special tissue from the human placenta, called cryopreserved placental allografts, affects the chances of prostate cancer coming back after surgery. These allografts are often placed near important nerves during prostate removal surgery to help men recover bladder control and sexual function. We reviewed the medical records of 566 men who had robotic prostate surgery between 2015 and 2021, comparing those who received the allografts with those who did not. We also tested how these tissues affect prostate cancer cells in the lab. Our results showed that the placental allografts did not change the risk of the cancer returning. However, men who received the allografts recovered bladder control faster than those who didn't. Lab tests also showed that these allografts might slow down the growth of prostate cancer cells. This suggests that placental allografts can help improve recovery without increasing cancer risk.

**Assessment Category**	**Score**	**Assessment Category**	**Score**	**Assessment Category**	**Score**
Comprehension Score Rater 1	5	Gunning Fog Score	14.4	Words	147
Comprehension Score Rater 2	5	SMOG Index	10.5	Complex words	26
Reading Age (years)	18.5	Coleman Liau Index	15.8	Percentage of complex words	17.69
Flesch-Kincaid Reading Ease	46.8	Automated Readability Index	13.0	Average words per sentence	18.38
Flesch-Kincaid Grade Level	11.2	Sentences	8	Average syllables per word	1.66

Manuscript 66

Du Q, Chan K, Kam MTY, Zheng KYC, Hung RHM, Wu PY. Volumetric Modulated Arc Therapy for High-Risk and Very High-Risk Locoregional Prostate Cancer in the Modern Era: Real-World Experience from an Asian Cohort. Cancers [Internet]. 2024 Aug 25;16(17):2964. Available from: http://dx.doi.org/10.3390/cancers16172964

Abstract

This study retrospectively evaluates the clinical outcomes of definitive volumetric modulated arc therapy (VMAT) for high-risk or very high-risk locoregional prostate cancer patients from an Asian institution. Consecutive patients who received VMAT (76 Gy in 38 fractions) between January 2017 and June 2022 were included. Whole pelvic radiotherapy (WPRT) (46 Gy in 23 fractions) was employed for clinically node-negative disease (cN0) and a Roach estimated risk of ≥15%, as well as simultaneous integrated boost (SIB) of 55-57.5 Gy to node-positive (cN1) disease. The primary endpoint was biochemical relapse-free survival (BRFS). Secondary endpoints included radiographic relapse-free survival (RRFS), metastasis-free survival (MFS) and prostate cancer-specific survival (PCSS). A total of 209 patients were identified. After a median follow-up of 47.5 months, the 4-year actuarial BRFS, RRFS, MFS and PCSS were 85.2%, 96.8%, 96.8% and 100%, respectively. The incidence of late grade ≥ 2 genitourinary (GU) and gastrointestinal (GI) toxicity were 15.8% and 11.0%, respectively. No significant difference in cancer outcomes or toxicity was observed between WPRT and prostate-only radiotherapy for cN0 patients. SIB to the involved nodes did not result in increased toxicity. International Society of Urological Pathology (ISUP) group 5 and cN1 stage were associated with worse RRFS (p p = 0.039). Five-year local experience demonstrates excellent clinical outcomes. PSMA PET-CT staging for high-grade disease and tailored pelvic irradiation based on nodal risk should be considered to maximize clinical benefit.

Keywords:

PSMA PET-CT, high-risk locoregional prostate cancer, simultaneous integrated boost, volumetric modulated arc therapy, whole pelvic radiotherapy

<u>Original Lay Summary</u>

Definitive radiotherapy (RT) with androgen deprivation therapy is the standard of care for high- or very high-risk locoregional prostate cancer. Despite various randomized trials, the optimal RT strategy is undefined. Practices in the selection for whole pelvic radiotherapy (WPRT), RT doses and techniques are considerably heterogeneous. Image-guided radiotherapy (IGRT) techniques have emerged to allow for precise dose prescription. The current study reports on the 5-year institutional experience of definitive volumetric modulated arc therapy using a standardized strategy of WPRT based on the estimated nodal risk, as well as simultaneous integrated boost to the involved nodes in the context of contemporary staging and RT techniques. An analysis of 209 patients demonstrated excellent clinical outcomes. Initial PSMA PET-CT compared to conventional imaging staging was associated with better biochemical relapse-free survival in patients with ISUP grade group 5 disease. These results suggest the approach of modern IGRT and the risk-adapted use of PSMA PET-CT staging and nodal irradiation in real-world clinical practice.

**Assessment Category**	**Score**	**Assessment Category**	**Score**	**Assessment Category**	**Score**
Comprehension Score Rater 1	5	Gunning Fog Score	19.1	Words	167
Comprehension Score Rater 2	4	SMOG Index	13.8	Complex words	46
Reading Age (years)	21.5	Coleman Liau Index	17.4	Percentage of complex words	27.54
Flesch-Kincaid Reading Ease	19.5	Automated Readability Index	15.6	Average words per sentence	20.88
Flesch-Kincaid Grade Level	15.7	Sentences	8	Average syllables per word	1.96

Lay Summary (Simple Prompt)

Prostate cancer that has spread to nearby tissues or lymph nodes can be challenging to treat, especially in high-risk cases. This study looked at the real-world outcomes of an advanced radiation technique called volumetric modulated arc therapy, used to precisely target cancer while minimizing damage to healthy tissue. Researchers analyzed data from 209 men treated between 2017 and 2022, some of whom also received radiation to the pelvic area or additional focused treatment to affected lymph nodes. After nearly four years of follow-up, most patients remained free from cancer recurrence, with excellent survival rates and manageable side effects. Treating the pelvic area did not increase side effects, and focused treatment of lymph nodes was safe. These results suggest that this modern radiation approach is highly effective and safe for men with high-risk prostate cancer, providing valuable guidance for improving treatment strategies in similar patient populations.

**Assessment Category**	**Score**	**Assessment Category**	**Score**	**Assessment Category**	**Score**
Comprehension Score Rater 1	4	Gunning Fog Score	16.9	Words	149
Comprehension Score Rater 2	4	SMOG Index	12.0	Complex words	26
Reading Age (years)	20.5	Coleman Liau Index	16.0	Percentage of complex words	17.45
Flesch-Kincaid Reading Ease	36.8	Automated Readability Index	16.4	Average words per sentence	24.83
Flesch-Kincaid Grade Level	14.3	Sentences	6	Average syllables per word	1.71

<u>Lay Summary (Extended Prompt)</u>

This study looked at how well a modern type of radiation therapy, called volumetric modulated arc therapy (VMAT), works for men with high-risk or very high-risk prostate cancer in an Asian population. We reviewed the medical records of 209 men treated between 2017 and 2022. Some patients received radiation to the whole pelvic area, while others had radiation focused only on the prostate. We also gave higher doses to areas where cancer had spread to nearby lymph nodes. After about four years of follow-up, we found that most patients had good outcomes, with high survival rates and low rates of cancer returning. Side effects related to the bladder and intestines were manageable. Treating the pelvic area or lymph nodes did not cause more serious side effects. These findings suggest that VMAT is an effective and safe treatment option for men with aggressive prostate cancer, offering strong cancer control with acceptable side effects.

**Assessment Category**	**Score**	**Assessment Category**	**Score**	**Assessment Category**	**Score**
Comprehension Score Rater 1	4	Gunning Fog Score	12.7	Words	155
Comprehension Score Rater 2	5	SMOG Index	9.2	Complex words	20
Reading Age (years)	16.5	Coleman Liau Index	13.1	Percentage of complex words	12.9
Flesch-Kincaid Reading Ease	52.9	Automated Readability Index	11.4	Average words per sentence	19.38
Flesch-Kincaid Grade Level	10.7	Sentences	8	Average syllables per word	1.59

Manuscript 67

Goode EA, Orozco-Moreno M, Hodgson K, Nabilah A, Murali M, Peng Z, et al. Sialylation Inhibition Can Partially Revert Acquired Resistance to Enzalutamide in Prostate Cancer Cells. Cancers [Internet]. 2024 Aug 24;16(17):2953. Available from: http://dx.doi.org/10.3390/cancers16172953

Abstract

Prostate cancer is a lethal solid malignancy and a leading cause of cancer-related deaths in males worldwide. Treatments, including radical prostatectomy, radiotherapy, and hormone therapy, are available and have improved patient survival; however, recurrence remains a huge clinical challenge. Enzalutamide is a second-generation androgen receptor antagonist that is used to treat castrate-resistant prostate cancer. Among patients who initially respond to enzalutamide, virtually all acquire secondary resistance, and an improved understanding of the mechanisms involved is urgently needed. Aberrant glycosylation, and, in particular, alterations to sialylated glycans, have been reported as mediators of therapy resistance in cancer, but a link between tumour-associated glycans and resistance to therapy in prostate cancer has not yet been investigated. Here, using cell line models, we show that prostate cancer cells with acquired resistance to enzalutamide therapy have an upregulation of the sialyltransferase ST6 beta-galactoside alpha-2,6-sialyltransferase 1 (ST6GAL1) and increased levels of α2,6-sialylated N-glycans. Furthermore, using the sialyltransferase inhibitor P-SiaFNEtoc, we discover that acquired resistance to enzalutamide can be partially reversed by combining enzalutamide therapy with sialic acid blockade. Our findings identify a potential role for ST6GAL1-mediated aberrant sialylation in acquired resistance to enzalutamide therapy for prostate cancer and suggest that sialic acid blockade in combination with enzalutamide may represent a novel therapeutic approach in patients with advanced disease. Our study also highlights the potential to bridge the fields of cancer biology and glycobiology to develop novel combination therapies for prostate cancer.

Keywords:

castrate resistance, combination therapies, enzalutamide, prostate cancer, sialic acid, sialyltransferase inhibitor

Original Lay Summary

Prostate cancer is the most common cancer in men and a major cause of cancer-related deaths around the world. Prostate cancer that has spread to other parts of the body (advanced prostate cancer) is often treated with a drug called enzalutamide, which is a type of hormone therapy. Enzalutamide works by blocking the effect of the testosterone hormone on prostate cancer cells to stop them from growing. While this can be effective for several years, unfortunately, many patients being treated with enzalutamide eventually go on to become resistant to treatment, and the therapy stops working. Here, we show that prostate cancer cells that have become resistant to enzalutamide have increased levels of a type of sugar (known as sialic acid) on their surfaces. We set out to test whether stripping sialic acid from the surface of prostate cancer cells could help keep enzalutamide working for longer. Excitingly, our experiments show that treating prostate cancer cells with a drug to block sialic acid can partially reverse enzalutamide resistance. These findings suggest that drugs targeting sialic acid could be used in combination with enzalutamide therapy to disarm drug resistance and provide urgently needed new treatment options for men with prostate cancer.

**Assessment Category**	**Score**	**Assessment Category**	**Score**	**Assessment Category**	**Score**
Comprehension Score Rater 1	5	Gunning Fog Score	16.0	Words	200
Comprehension Score Rater 2	5	SMOG Index	11.2	Complex words	30
Reading Age (years)	19.5	Coleman Liau Index	15.1	Percentage of complex words	15.0
Flesch-Kincaid Reading Ease	38.5	Automated Readability Index	15.8	Average words per sentence	25.0

Flesch-Kincaid Grade Level	14.1	Sentences	8	Average syllables per word	1.69

Lay Summary (Simple Prompt)

Prostate cancer is a common and serious disease in men, and while treatments like surgery, radiation, and hormone therapy help, the cancer often returns. Enzalutamide is a drug used to treat advanced prostate cancer, but over time, many patients develop resistance, making the drug less effective. This study explored why this resistance happens and how it might be overcome. Researchers found that resistant prostate cancer cells produce more of a specific sugar molecule called sialic acid, which may help the cancer survive despite treatment. By using a special inhibitor to block the production of this sugar, they were able to partially restore the effectiveness of enzalutamide in laboratory-grown cancer cells. This discovery suggests that combining enzalutamide with treatments that target sialic acid could be a promising new approach for men with advanced prostate cancer, offering hope for more effective therapies in the future.

**Assessment Category**	**Score**	**Assessment Category**	**Score**	**Assessment Category**	**Score**
Comprehension Score Rater 1	5	Gunning Fog Score	17.7	Words	144
Comprehension Score Rater 2	5	SMOG Index	12.7	Complex words	29
Reading Age (years)	20.5	Coleman Liau Index	16.1	Percentage of complex words	20.14
Flesch-Kincaid Reading Ease	32.7	Automated Readability Index	16.1	Average words per sentence	24.0

Flesch-Kincaid Grade Level	14.7	Sentences	6	Average syllables per word	1.77

Lay Summary (Extended Prompt)

Prostate cancer is a common and serious disease in men. One of the treatments for advanced prostate cancer is a drug called enzalutamide, which helps slow the growth of cancer. Unfortunately, many patients eventually stop responding to this drug, and scientists are trying to understand why. In this study, researchers discovered that prostate cancer cells that become resistant to enzalutamide have higher levels of certain sugar molecules on their surface, controlled by an enzyme called ST6GAL1. They tested a special drug that blocks these sugar molecules and found that it could partially restore the cancer cells' sensitivity to enzalutamide. This means that combining enzalutamide with a drug that blocks these sugars might be a new way to treat prostate cancer when it becomes resistant to current therapies. This research could help develop better treatment options for patients with advanced prostate cancer in the future.

**Assessment Category**	**Score**	**Assessment Category**	**Score**	**Assessment Category**	**Score**
Comprehension Score Rater 1	5	Gunning Fog Score	13.8	Words	144
Comprehension Score Rater 2	5	SMOG Index	9.8	Complex words	20
Reading Age (years)	18.5	Coleman Liau Index	15.8	Percentage of complex words	13.89
Flesch-Kincaid Reading Ease	46.7	Automated Readability Index	14.2	Average words per sentence	20.57
Flesch-Kincaid Grade Level	11.9	Sentences	7	Average syllables per word	1.65

Manuscript 68

Antolin A, Roson N, Mast R, Arce J, Almodovar R, Cortada R, et al. The Role of Radiomics in the Prediction of Clinically Significant Prostate Cancer in the PI-RADS v2 and v2.1 Era: A Systematic Review. Cancers [Internet]. 2024 Aug 24;16(17):2951. Available from: http://dx.doi.org/10.3390/cancers16172951

Abstract

Early detection of clinically significant prostate cancer (csPCa) has substantially improved with the latest PI-RADS versions. However, there is still an overdiagnosis of indolent lesions (iPCa), and radiomics has emerged as a potential solution. The aim of this systematic review is to evaluate the role of handcrafted and deep radiomics in differentiating lesions with csPCa from those with iPCa and benign lesions on prostate MRI assessed with PI-RADS v2 and/or 2.1. The literature search was conducted in PubMed, Cochrane, and Web of Science databases to select relevant studies. Quality assessment was carried out with Quality Assessment of Diagnostic Accuracy Studies 2 (QUADAS-2), Radiomic Quality Score (RQS), and Checklist for Artificial Intelligence in Medical Imaging (CLAIM) tools. A total of 14 studies were deemed as relevant from 411 publications. The results highlighted a good performance of handcrafted and deep radiomics methods for csPCa detection, but without significant differences compared to radiologists (PI-RADS) in the few studies in which it was assessed. Moreover, heterogeneity and restrictions were found in the studies and quality analysis, which might induce bias. Future studies should tackle these problems to encourage clinical applicability. Prospective studies and comparison with radiologists (PI-RADS) are needed to better understand its potential.

Keywords:

PI-RADS, clinically significant prostate cancer, deep learning, machine learning, magnetic resonance imaging, prediction, radiomics, systematic review

Original Lay Summary

There is still an overdiagnosis of indolent prostate cancer (iPCa) lesions using the Prostate Imaging-Reporting and Data System (PI-RADS), and radiomics has emerged as a promising tool to improve the diagnosis of clinically significant prostate cancer (csPCa) lesions. However, the current state and applicability of radiomics remains a challenge. This systematic review aims at evaluating the evidence of handcrafted and deep radiomics in differentiating lesions at risk of having csPCa from those with iPCa and benign pathology. The review highlighted a good performance of radiomics but without significant differences with radiologist assessment (PI-RADS), as well as several methodological limitations in the reported studies, which might induce bias. Future studies should improve methodological aspects to ensure the clinical applicability of radiomics, especially the need for clinical prospective studies and the comparison with PI-RADS.

**Assessment Category**	**Score**	**Assessment Category**	**Score**	**Assessment Category**	**Score**
Comprehension Score Rater 1	5	Gunning Fog Score	21.5	Words	136
Comprehension Score Rater 2	4	SMOG Index	15.9	Complex words	38
Reading Age (years)	24.0	Coleman Liau Index	18.5	Percentage of complex words	27.94
Flesch-Kincaid Reading Ease	6.9	Automated Readability Index	19.6	Average words per sentence	27.2
Flesch-Kincaid Grade Level	19.1	Sentences	5	Average syllables per word	2.04

<u>Lay Summary (Simple Prompt)</u>

Prostate cancer can range from harmless to life-threatening, and it's important to tell the difference early to avoid unnecessary treatments. Doctors use MRI scans with a system called PI-RADS to identify serious cases, but this method isn't perfect and sometimes leads to overdiagnosis of harmless cancers. This study reviewed research on "radiomics," a technique that uses advanced computer analysis to find patterns in MRI images that the human eye might miss. By analyzing 14 relevant studies, the review found that radiomics can help detect significant prostate cancers with good accuracy, similar to experienced doctors using PI-RADS. However, the quality and methods of these studies varied, which could affect the reliability of the results. More research is needed to confirm how well radiomics works in real clinical settings, but it shows promise as a helpful tool alongside traditional diagnostic methods.

**Assessment Category**	**Score**	**Assessment Category**	**Score**	**Assessment Category**	**Score**
Comprehension Score Rater 1	5	Gunning Fog Score	16.5	Words	143
Comprehension Score Rater 2	5	SMOG Index	12.0	Complex words	26
Reading Age (years)	20.5	Coleman Liau Index	15.3	Percentage of complex words	18.18
Flesch-Kincaid Reading Ease	34.7	Automated Readability Index	15.4	Average words per sentence	23.83
Flesch-Kincaid Grade Level	14.3	Sentences	6	Average syllables per word	1.75

Lay Summary (Extended Prompt)

Prostate cancer can be serious or slow-growing, and it's important to tell the difference early to avoid unnecessary treatments. Doctors use MRI scans with a system called PI-RADS to help find aggressive prostate cancers. However, this method sometimes detects harmless cancers, leading to overdiagnosis. This study reviews research on a new approach called radiomics, which uses computer tools to analyze MRI images in more detail. By examining 14 studies from hundreds of scientific papers, the review found that radiomics can help identify aggressive prostate cancers, performing well in many cases. However, it did not consistently outperform experienced doctors using PI-RADS. Also, the quality of the studies varied, which may affect the results. More research is needed to confirm how useful radiomics can be in real medical practice. If improved, radiomics could support doctors in making better decisions about prostate cancer diagnosis.

**Assessment Category**	**Score**	**Assessment Category**	**Score**	**Assessment Category**	**Score**
Comprehension Score Rater 1	5	Gunning Fog Score	13.6	Words	144
Comprehension Score Rater 2	5	SMOG Index	9.9	Complex words	26
Reading Age (years)	18.5	Coleman Liau Index	16.1	Percentage of complex words	18.06
Flesch-Kincaid Reading Ease	40.2	Automated Readability Index	12.1	Average words per sentence	16.0
Flesch-Kincaid Grade Level	11.6	Sentences	9	Average syllables per word	1.78

Manuscript 69

Liu JC, Ruan XH, Chun TT, Yao C, Huang D, Wong HL, et al. MRI T2w Radiomics-Based Machine Learning Models in Imaging Simulated Biopsy Add Diagnostic Value to PI-RADS in Predicting Prostate Cancer: A Retrospective Diagnostic Study. Cancers [Internet]. 2024 Aug 23;16(17):2944. Available from: http://dx.doi.org/10.3390/cancers16172944

Abstract

Background: Currently, prostate cancer (PCa) prebiopsy medical image diagnosis mainly relies on mpMRI and PI-RADS scores. However, PI-RADS has its limitations, such as inter- and intra-radiologist variability and the potential for imperceptible features. The primary objective of this study is to evaluate the effectiveness of a machine learning model based on radiomics analysis of MRI T2-weighted (T2w) images for predicting PCa in prebiopsy cases.

Method: A retrospective analysis was conducted using 820 lesions (363 cases, 457 controls) from The Cancer Imaging Archive (TCIA) Database for model development and validation. An additional 83 lesions (30 cases, 53 controls) from Hong Kong Queen Mary Hospital were used for independent external validation. The MRI T2w images were preprocessed, and radiomic features were extracted. Feature selection was performed using Cross Validation Least Angle Regression (CV-LARS). Using three different machine learning algorithms, a total of 18 prediction models and 3 shape control models were developed. The performance of the models, including the area under the curve (AUC) and diagnostic values such as sensitivity, specificity, positive predictive value (PPV), and negative predictive value (NPV), were compared to the PI-RADS scoring system for both internal and external validation.

Results: All the models showed significant differences compared to the shape control model (all $p < 0.001$, except SVM model PI-RADS+2 Features $p = 0.004$, SVM model PI-RADS+3 Features $p = 0.002$). In internal validation, the best model, based

on the LR algorithm, incorporated 3 radiomic features (AUC = 0.838, sensitivity = 76.85%, specificity = 77.36%). In external validation, the LR (3 features) model outperformed PI-RADS in predictive value with AUC 0.870 vs. 0.658, sensitivity 56.67% vs. 46.67%, specificity 92.45% vs. 84.91%, PPV 80.95% vs. 63.64%, and NPV 79.03% vs. 73.77%.

Conclusions: The machine learning model based on radiomics analysis of MRI T2w images, along with simulated biopsy, provides additional diagnostic value to the PI-RADS scoring system in predicting PCa.

Keywords:

biopsy, machine learning, multi-parametric magnetic resonance imaging, prostate cancer, prostate imaging reporting and data system, radiomics

<u>Original Lay Summary</u>

Prostate mpMRI is currently the most widely used image diagnosis approach to detect prostate cancer, while the PI-RADS system was developed to standardize and improve the accuracy of suspicious lesion identification on MRI. However, there still remain several limitations including inter-individual inconsistencies and naked-eye insufficiency. This study aims to apply AI technology to image interpretation to enhance diagnostic efficiency and explore the use of T2-weighted image-based stimulated biopsy in predicting prostate cancer (PCa). Using 820 lesions from The Cancer Imaging Archive database and 83 lesions from Hong Kong Queen Mary Hospital, we constructed 18 machine-learning models based on three algorithms and conducted both internal and external validation. We found that the logistic regression-based model provides additional diagnostic value to the PI-RADS in predicting PCa.

**Assessment Category**	**Score**	**Assessment Category**	**Score**	**Assessment Category**	**Score**
Comprehension Score Rater 1	5	Gunning Fog Score	10.6	Words	132
Comprehension Score Rater 2	4	SMOG Index	15.2	Complex words	35
Reading Age (years)	23.5	Coleman Liau Index	17.4	Percentage of complex words	26.52
Flesch-Kincaid Reading Ease	17.2	Automated Readability Index	18.3	Average words per sentence	26.4
Flesch-Kincaid Grade Level	17.4	Sentences	5	Average syllables per word	1.92

<u>Lay Summary (Simple Prompt)</u>

Prostate cancer is commonly diagnosed using MRI scans and a scoring system called PI-RADS, which helps doctors identify suspicious areas. However, this system can sometimes miss important details and varies between different doctors. Our study explored whether adding advanced computer techniques, known as machine learning, to analyze specific features from MRI images could improve prostate cancer detection. We reviewed MRI data from hundreds of patients and developed several computer models to predict the presence of cancer. Our best model was more accurate than the traditional PI-RADS system, identifying cancer more reliably while reducing false alarms. This approach could help doctors make better decisions before performing a biopsy, potentially leading to earlier and more accurate diagnoses of prostate cancer.

**Assessment Category**	**Score**	**Assessment Category**	**Score**	**Assessment Category**	**Score**
Comprehension Score Rater 1	4	Gunning Fog Score	17.3	Words	120
Comprehension Score Rater 2	4	SMOG Index	12.5	Complex words	28
Reading Age (years)	21.5	Coleman Liau Index	18.2	Percentage of complex words	23.33
Flesch-Kincaid Reading Ease	30.0	Automated Readability Index	15.8	Average words per sentence	20.0
Flesch-Kincaid Grade Level	14.0	Sentences	6	Average syllables per word	1.85

Lay Summary (Extended Prompt)

Prostate cancer is often detected using special MRI scans and a scoring system called PI-RADS. However, this system can sometimes miss important details, leading to different results depending on the doctor reviewing the images. In this study, we explored whether adding information from a computer-based method called machine learning, which analyzes MRI images in detail (radiomics), could improve prostate cancer detection. We used MRI data from hundreds of patients to create and test models that predict the presence of cancer. Our best model was more accurate than PI-RADS alone, correctly identifying cancer in more cases while reducing false alarms. This means that combining traditional imaging scores with advanced computer analysis could help doctors detect prostate cancer more reliably, leading to better decisions about who needs a biopsy and who does not. This approach could make prostate cancer diagnosis more accurate and consistent for patients.

**Assessment Category**	**Score**	**Assessment Category**	**Score**	**Assessment Category**	**Score**
Comprehension Score Rater 1	5	Gunning Fog Score	15.7	Words	147
Comprehension Score Rater 2	5	SMOG Index	11.4	Complex words	27
Reading Age (years)	19.5	Coleman Liau Index	16.3	Percentage of complex words	18.37
Flesch-Kincaid Reading Ease	39.3	Automated Readability Index	14.8	Average words per sentence	21.0
Flesch-Kincaid Grade Level	13.0	Sentences	7	Average syllables per word	1.73

Manuscript 70

Olatunde D, De Benedetti A. TLK1>Nek1 Axis Promotes Nuclear Retention and Activation of YAP with Implications for Castration-Resistant Prostate Cancer. Cancers [Internet]. 2024 Aug 22;16(16):2918. Available from: http://dx.doi.org/10.3390/cancers16162918

Abstract

Despite some advances in controlling the progression of prostate cancer (PCa) that is refractory to the use of ADT/ARSI, most patients eventually succumb to the disease, and there is a pressing need to understand the mechanisms that lead to the development of CRPC. A common mechanism is the ability to integrate AR signals from vanishing levels of testosterone, with the frequent participation of YAP as a co-activator, and pointing to the deregulation of the Hippo pathway as a major determinant. We have recently shown that YAP is post-transcriptionally activated via the TLK1>NEK1 axis by stabilizing phosphorylation at Y407. We are now solidifying this work by showing the following: (1) The phosphorylation of Y407 is critical for YAP retention/partition in the nuclei, and J54 (TLK1i) reverses this along with YAP-Y407 dephosphorylation. (2) The enhanced degradation of (cytoplasmic) YAP is increased by J54 counteracting its Enzalutamide-induced accumulation. (3) The basis for all these effects, including YAP nuclear retention, can be explained by the stronger association of pYAP-Y407 with its transcriptional co-activators, AR and TEAD1. (4) We demonstrate that ChIP for GFP-YAP-wt, but hardly for the GFP-YAP-Y407F mutant, at the promoters of typical ARE- and TEAD1-driven genes is readily detected but becomes displaced after treatment with J54. (5) While xenografts of LNCaP cells show rapid regression following treatment with ARSI+J54, in the VCaP model, driven by the TMPRSS2-ERG oncogenic translocation, tumors initially respond well to the combination but subsequently recur, despite the continuous suppression of pNek1-T141 and pYAP-Y407. This suggests an alternative parallel pathway for CRPC progression for VCaP tumors in the long term, which may be separate from the observed ENZ-driven YAP deregulation, although clearly some YAP gene targets like

PD-L1, that are found to accumulate following prolonged ENZ treatment, are still suppressed by the concomitant addition of J54.

Keywords:

CRPC progression, NEK1, PCA models, TLK1, YAP regulation, tumors

Original Lay Summary

Treatment with ARSI results in an increased expression of the TLK1B splice variant and consequent activation of the TLK1>NEK1>YAP axis. We now provide important details of the significance of the NEK1-mediated phosphorylation of the YAP-Y407F residue for its nuclear localization and transcriptional output. Furthermore, we study the effect of the pharmacologic inhibition of this pathway in a xenograft model that readily converts from androgen-dependent to castration-resistant (CRPC) prostate cancer, as a first approach to translating our work toward a clinical investigation.

**Assessment Category**	**Score**	**Assessment Category**	**Score**	**Assessment Category**	**Score**
Comprehension Score Rater 1	4	Gunning Fog Score	22.2	Words	85
Comprehension Score Rater 2	4	SMOG Index	15.9	Complex words	23
Reading Age (years)	24.0	Coleman Liau Index	17.5	Percentage of complex words	27.06
Flesch-Kincaid Reading Ease	15.8	Automated Readability Index	19.3	Average words per sentence	28.33
Flesch-Kincaid Grade Level	18.1	Sentences	3	Average syllables per word	1.92

Lay Summary (Simple Prompt)

Prostate cancer that no longer responds to hormone therapy, known as castration-resistant prostate cancer, is challenging to treat and often leads to poor outcomes. This study investigates a specific pathway in cancer cells, called the TLK1>Nek1 axis, which helps a protein named YAP stay active inside the cell nucleus, promoting cancer growth. We discovered that blocking this pathway with a drug called J54 reduces YAP's activity, making cancer cells more vulnerable to treatment. In laboratory models, combining J54 with existing prostate cancer drugs led to significant tumor shrinkage. However, some tumors eventually found new ways to grow, suggesting that additional factors may contribute to disease progression. Our findings highlight a potential new approach to treating resistant prostate cancer by targeting the TLK1>Nek1-YAP pathway, which could improve the effectiveness of current therapies and help delay cancer recurrence.

**Assessment Category**	**Score**	**Assessment Category**	**Score**	**Assessment Category**	**Score**
Comprehension Score Rater 1	5	Gunning Fog Score	16.7	Words	138
Comprehension Score Rater 2	4	SMOG Index	12.0	Complex words	26
Reading Age (years)	20.5	Coleman Liau Index	17.3	Percentage of complex words	18.84
Flesch-Kincaid Reading Ease	35.1	Automated Readability Index	16.6	Average words per sentence	23.0
Flesch-Kincaid Grade Level	14.1	Sentences	6	Average syllables per word	1.75

Lay Summary (Extended Prompt)

Prostate cancer can become resistant to treatments that lower male hormones, leading to a more aggressive form called castration-resistant prostate cancer (CRPC). This study explores how a protein called YAP helps cancer cells survive in this resistant stage. The researchers discovered that another protein, TLK1, activates YAP, allowing it to stay inside the cell's nucleus where it supports cancer growth. They tested a drug called J54, which blocks TLK1, and found that it reduces YAP's activity and slows tumor growth in laboratory models. However, some tumors eventually found ways to resist this treatment. These findings are important because they reveal new details about how prostate cancer becomes resistant to therapy, which could help develop better treatments to control the disease and improve patient outcomes.

**Assessment Category**	**Score**	**Assessment Category**	**Score**	**Assessment Category**	**Score**
Comprehension Score Rater 1	5	Gunning Fog Score	14.1	Words	125
Comprehension Score Rater 2	4	SMOG Index	10.1	Complex words	18
Reading Age (years)	18.5	Coleman Liau Index	16.2	Percentage of complex words	14.4
Flesch-Kincaid Reading Ease	46.3	Automated Readability Index	14.6	Average words per sentence	20.83
Flesch-Kincaid Grade Level	12.0	Sentences	6	Average syllables per word	1.65

Manuscript 71

Vera G, Rojas PA, Black JB, San Francisco IF. Usefulness of Tissue Biomarkers versus Prostate-Specific Membrane Antigen-Positron Emission Tomography for Prostate Cancer Biochemical Recurrence after Radical Prostatectomy. Cancers [Internet]. 2024 Aug 19;16(16):2879. Available from: http://dx.doi.org/10.3390/cancers16162879

Abstract

Despite curative-intent local therapy, approximately 27% to 53% of prostate cancer (PCa) patients experience prostate-specific antigen (PSA) recurrence, known as biochemical recurrence (BCR). BCR significantly raises the risk of PCa-related morbidity and mortality, yet there is no consensus on optimal management. Prostate-specific membrane antigen-positron emission tomography (PSMA PET) has emerged as highly sensitive imaging, distinguishing local recurrences from distant metastases, crucially influencing treatment decisions. Genomic biomarkers such as Decipher, Prolaris, and Oncotype DX contribute to refining recurrence risk profiles, guiding decisions on intensifying adjuvant therapies, like radiotherapy and androgen deprivation therapy (ADT). This review assesses PSMA PET and biomarker utility in post-radical prostatectomy BCR scenarios, highlighting their impact on clinical decision-making. Despite their promising roles, the routine integration of biomarkers is limited by availability and cost, requiring further evidence. PSMA PET remains indispensable for restaging and treatment evaluation in these patients. Integrating biomarkers and PSMA PET promises to optimize personalized management strategies for BCR, though more comprehensive consensus-building studies are needed to define their standardized utility in clinical practice.

Keywords:

biomarkers, novel images, prostate cancer recurrence

<u>Original Lay Summary</u>

Patients undergoing definitive prostate cancer therapy experience recurrence despite standard-of-care interventions. Biochemical recurrence is detected initially via prostate-specific antigen (PSA) monitoring and is defined as biochemical recurrence. The clinical importance of the early detection of biochemical recurrence is underscored in this study. This review seeks to further elucidate modalities used for earlier identification of biochemical recurrence. Specifically, we highlight in this review the clinical utility of both tissue biomarkers and prostate-specific membrane antigen-positron emission tomography (PSMA PET) in monitoring patients for recurrence. The summarization provided in this article can help guide clinicians in understanding the recent developments in this field.

**Assessment Category**	**Score**	**Assessment Category**	**Score**	**Assessment Category**	**Score**
Comprehension Score Rater 1	5	Gunning Fog Score	23.8	Words	105
Comprehension Score Rater 2	3	SMOG Index	15.6	Complex words	44
Reading Age (years)	24.0	Coleman Liau Index	22.3	Percentage of complex words	41.9
Flesch-Kincaid Reading Ease	-5.1	Automated Readability Index	17.8	Average words per sentence	17.5
Flesch-Kincaid Grade Level	18.3	Sentences	6	Average syllables per word	2.3

<u>Lay Summary (Simple Prompt)</u>

After prostate cancer surgery, many patients experience a rise in prostate-specific antigen (PSA) levels, known as biochemical recurrence, which can signal the return of cancer. Deciding the best treatment in these cases is challenging. This study compares two important tools that help guide these decisions: advanced imaging with prostate-specific membrane antigen positron emission tomography (PSMA PET) and genetic tests called tissue biomarkers. PSMA PET is highly effective in detecting whether the cancer has come back locally or spread to other parts of the body, which is crucial for planning treatment. Tissue biomarkers, on the other hand, help predict the likelihood of cancer returning and whether additional therapies like radiation or hormone treatment might be needed. While PSMA PET is widely used, the routine use of biomarkers is limited due to cost and availability. Combining both tools could lead to more personalized, effective treatment plans, improving outcomes for patients with recurring prostate cancer.

**Assessment Category**	**Score**	**Assessment Category**	**Score**	**Assessment Category**	**Score**
Comprehension Score Rater 1	5	Gunning Fog Score	17.9	Words	154
Comprehension Score Rater 2	4	SMOG Index	12.9	Complex words	35
Reading Age (years)	21.5	Coleman Liau Index	17.0	Percentage of complex words	22.73
Flesch-Kincaid Reading Ease	34.0	Automated Readability Index	15.8	Average words per sentence	22.0
Flesch-Kincaid Grade Level	14.0	Sentences	7	Average syllables per word	1.78

<u>Lay Summary (Extended Prompt)</u>

After surgery to remove the prostate due to cancer, many men face the risk of the cancer coming back, which is detected by rising levels of a protein called PSA in the blood. This is known as biochemical recurrence. Doctors use advanced imaging tests, like prostate-specific membrane antigen (PSMA) PET scans, to find out if the cancer has returned and where it might be—either near the original site or spread to other parts of the body. In addition, tissue biomarkers, which are special tests done on cancer cells, can help predict how likely the cancer is to come back and guide decisions about further treatments, such as radiation or hormone therapy. This study compares the usefulness of PSMA PET scans and tissue biomarkers to see which provides better information for managing prostate cancer after surgery. Understanding how to combine these tools can help doctors create more personalized and effective treatment plans.

**Assessment Category**	**Score**	**Assessment Category**	**Score**	**Assessment Category**	**Score**
Comprehension Score Rater 1	5	Gunning Fog Score	15.9	Words	152
Comprehension Score Rater 2	4	SMOG Index	11.1	Complex words	22
Reading Age (years)	19.5	Coleman Liau Index	13.7	Percentage of complex words	14.47
Flesch-Kincaid Reading Ease	45.3	Automated Readability Index	14.8	Average words per sentence	25.33
Flesch-Kincaid Grade Level	13.2	Sentences	6	Average syllables per word	1.61

Manuscript 72

Erb HHH, Polishchuk N, Stasyk O, Kahya U, Weigel MM, Dubrovska A. Glutamine Metabolism and Prostate Cancer. Cancers [Internet]. 2024 Aug 18;16(16):2871. Available from: http://dx.doi.org/10.3390/cancers16162871

Abstract

Glutamine (Gln) is a non-essential amino acid that is involved in the development and progression of several malignancies, including prostate cancer (PCa). While Gln is non-essential for non-malignant prostate epithelial cells, PCa cells become highly dependent on an exogenous source of Gln. The Gln metabolism in PCa is tightly controlled by well-described oncogenes such as MYC, AR, and mTOR. These oncogenes contribute to therapy resistance and progression to the aggressive castration-resistant PCa. Inhibition of Gln catabolism impedes PCa growth, survival, and tumor-initiating potential while sensitizing the cells to radiotherapy. Therefore, given its significant role in tumor growth, targeting Gln metabolism is a promising approach for developing new therapeutic strategies. Ongoing clinical trials evaluate the safety and efficacy of Gln catabolism inhibitors in combination with conventional and targeted therapies in patients with various solid tumors, including PCa. Further understanding of how PCa cells metabolically interact with their microenvironment will facilitate the clinical translation of Gln inhibitors and help improve therapeutic outcomes. This review focuses on the role of Gln in PCa progression and therapy resistance and provides insights into current clinical trials.

Keywords:

androgen receptor, chemotherapy, glutamine, prostate cancer, radiation therapy, therapy resistance

Original Lay Summary

Glutamine (Gln) plays a critical role in the development and progression of prostate cancer (PCa). Non-malignant prostate cells are not addicted to Gln, but PCa cells become Gln-dependent due to the low levels of the Gln-producing enzyme glutamine synthetase. Gln metabolism in PCa is controlled by oncogenes such as MYC, AR, and mTOR, which contribute to therapy resistance and more aggressive forms of the disease. Studies have shown that depriving PCa cells of Gln can inhibit their growth and survival and make them more sensitive to radiotherapy. Targeting Gln metabolism is, therefore, a promising approach for PCa treatment. Clinical trials are currently testing the safety and efficacy of drugs that inhibit Gln metabolism in combination with other therapies for different types of cancer, including PCa. Understanding of how tumor cells metabolically interact with their environment may help improve these treatments and patient outcomes. In this review, we present the latest advances in Gln research in PCa and insights into the current clinical trials.

**Assessment Category**	**Score**	**Assessment Category**	**Score**	**Assessment Category**	**Score**
Comprehension Score Rater 1	5	Gunning Fog Score	17.7	Words	166
Comprehension Score Rater 2	4	SMOG Index	12.9	Complex words	40
Reading Age (years)	20.5	Coleman Liau Index	15.4	Percentage of complex words	24.1
Flesch-Kincaid Reading Ease	37.0	Automated Readability Index	13.9	Average words per sentence	20.75
Flesch-Kincaid Grade Level	13.3	Sentences	8	Average syllables per word	1.76

Lay Summary (Simple Prompt)

Prostate cancer cells rely heavily on a nutrient called glutamine, which helps them grow and survive, unlike normal prostate cells that do not depend on it as much. This study explores how prostate cancer uses glutamine to fuel its progression and resist common treatments like hormone therapy and radiation. Researchers found that blocking glutamine metabolism can slow down cancer growth, reduce its ability to spread, and make cancer cells more sensitive to treatment. This approach is currently being tested in clinical trials to see if it can improve outcomes for patients with advanced prostate cancer. By better understanding how prostate cancer cells interact with their environment and depend on glutamine, scientists hope to develop new, more effective therapies that target this weakness and offer better treatment options for patients.

**Assessment Category**	**Score**	**Assessment Category**	**Score**	**Assessment Category**	**Score**
Comprehension Score Rater 1	5	Gunning Fog Score	16.5	Words	129
Comprehension Score Rater 2	5	SMOG Index	11.6	Complex words	20
Reading Age (years)	20.5	Coleman Liau Index	16.5	Percentage of complex words	15.5
Flesch-Kincaid Reading Ease	39.0	Automated Readability Index	17.3	Average words per sentence	25.8
Flesch-Kincaid Grade Level	14.2	Sentences	5	Average syllables per word	1.67

Lay Summary (Extended Prompt)

Prostate cancer is one of the most common cancers in men, and researchers are working to find better ways to treat it. This study looks at how prostate cancer cells use a nutrient called glutamine, which is important for their growth and survival. While normal prostate cells do not rely heavily on glutamine, prostate cancer cells become highly dependent on it to grow and resist treatments. Certain genes in cancer cells help control how they use glutamine, making the cancer harder to treat. Scientists have found that blocking glutamine metabolism can slow down cancer growth, make the cancer cells weaker, and help existing treatments like radiation therapy work better. This research highlights the potential of targeting glutamine metabolism as a new way to treat prostate cancer. Understanding how cancer cells depend on glutamine could lead to more effective therapies and improve outcomes for patients with advanced prostate cancer.

**Assessment Category**	**Score**	**Assessment Category**	**Score**	**Assessment Category**	**Score**
Comprehension Score Rater 1	5	Gunning Fog Score	14.1	Words	148
Comprehension Score Rater 2	5	SMOG Index	10.1	Complex words	21
Reading Age (years)	18.5	Coleman Liau Index	15.3	Percentage of complex words	14.19
Flesch-Kincaid Reading Ease	49.9	Automated Readability Index	14.0	Average words per sentence	21.14
Flesch-Kincaid Grade Level	11.6	Sentences	7	Average syllables per word	1.6

Manuscript 73

Wang H, Gong L, Huang X, White SD, Chung HT, Vesprini D, et al. Potentiating Salvage Radiotherapy in Radiorecurrent Prostate Cancer Through Anti-CTLA4 Therapy: Implications from a Syngeneic Model. Cancers [Internet]. 2024 Aug 14;16(16):2839. Available from: http://dx.doi.org/10.3390/cancers16162839

Abstract

High-risk prostate cancer (PCa) is a leading cause in cancer death and can elicit significant morbidity and mortality. Currently, the salvage of local disease recurrence after radiation therapy (RT) is a major clinical problem. Immune checkpoint inhibitors (ICIs), which enhance immune activation, have demonstrated clinical therapeutic promise in combination with ionizing radiation (IR) in certain advanced cancers. We generated the TRAMP-C2 HF radiorecurrent syngeneic mouse model to evaluate the therapeutic efficacy of ICIs in combination with RT. The administration of anti-PDL1 and/or anti-CTLA4 did not achieve a significant tumor growth delay compared to the control. The combination of IR and anti-PDL1 did not yield additional a growth delay compared to IR and the isotype control. Strikingly, a significant tumor growth delay and complete cure in one-third of the mice were seen with the combination of IR and anti-CTLA4. Immune cells in tumor-draining lymph nodes and tumor-infiltrating lymphocytes from mice treated with IR and anti-CTLA4 demonstrated an upregulation of genes in T-cell functions and enrichment in both CD4+ and CD8+ T-cell populations compared to mice given IR and the isotype control. Taken together, these results indicate enhancement of T-cell response in radiorecurrent PCa by IR and anti-CTLA4.

Keywords:

anti-CTLA4, brachytherapy, prostate cancer, radiorecurrence, radiotherapy

<u>Original Lay Summary</u>

Advanced prostate cancer (PCa) is a prominent contributor to cancer-related fatalities and is associated with significant morbidity and mortality. Currently, addressing the local recurrence of the disease after radiation therapy (RT) poses a significant clinical challenge. We established the first syngeneic model of radiorecurrent PCa to evaluate the effectiveness of immune checkpoint inhibitors (ICIs) in combination with high-dose ionizing radiation (IR). We observed an enhanced anti-tumor response, which led to a delay in tumor growth and, in some cases, a complete elimination of tumors when combining IR with anti-CTLA4. This improvement was linked to an enhanced activation of total T cells, CD4+ helper T cells, and CD8+ cytotoxic T cells in both the draining lymph node and tumor. These results hold substantial potential for translation into clinical practice, serving as a proof of concept for the application of brachytherapy and immune checkpoint inhibitors (ICIs) in cases of recurrent disease.

**Assessment Category**	**Score**	**Assessment Category**	**Score**	**Assessment Category**	**Score**
Comprehension Score Rater 1	5	Gunning Fog Score	20.1	Words	153
Comprehension Score Rater 2	4	SMOG Index	14.5	Complex words	38
Reading Age (years)	22.5	Coleman Liau Index	16.2	Percentage of complex words	24.84
Flesch-Kincaid Reading Ease	22.8	Automated Readability Index	16.9	Average words per sentence	25.5
Flesch-Kincaid Grade Level	16.4	Sentences	6	Average syllables per word	1.87

Lay Summary (Simple Prompt)

Prostate cancer that returns after radiation therapy is difficult to treat, and finding better treatment options is critical. This study explored whether combining radiation with a type of immunotherapy called anti-CTLA4, which helps the immune system recognize and attack cancer cells, could improve outcomes. Using a specialized mouse model that mimics prostate cancer recurrence after radiation, researchers tested different combinations of treatments. They found that while radiation alone or combined with another immunotherapy (anti-PDL1) had limited effects, the combination of radiation and anti-CTLA4 significantly slowed tumor growth and even cured some cases. This powerful response was linked to a stronger activation of immune cells that target cancer. These findings suggest that adding anti-CTLA4 therapy to radiation could be a promising new approach for treating prostate cancer that comes back after initial treatment, potentially leading to better long-term outcomes for patients.

**Assessment Category**	**Score**	**Assessment Category**	**Score**	**Assessment Category**	**Score**
Comprehension Score Rater 1	5	Gunning Fog Score	17.7	Words	145
Comprehension Score Rater 2	4	SMOG Index	12.7	Complex words	29
Reading Age (years)	21.5	Coleman Liau Index	18.2	Percentage of complex words	20.0
Flesch-Kincaid Reading Ease	24.2	Automated Readability Index	17.8	Average words per sentence	24.17
Flesch-Kincaid Grade Level	15.9	Sentences	6	Average syllables per word	1.87

<u>Lay Summary (Extended Prompt)</u>

Prostate cancer can return after radiation treatment, making it hard to control. This study explored whether combining radiation therapy with a type of immune-boosting drug, called anti-CTLA4, could improve outcomes. Using a special mouse model that mimics prostate cancer recurrence in humans, researchers tested how well this combination worked. They found that radiation therapy alone or with other immune drugs had limited effects. However, when radiation was combined with anti-CTLA4, it significantly slowed tumor growth, and one-third of the mice were completely cured. This strong response was linked to increased activity of important immune cells that help fight cancer. These findings suggest that adding anti-CTLA4 therapy to radiation could be a promising way to treat prostate cancer that comes back after initial treatment, offering hope for better outcomes in the future.

**Assessment Category**	**Score**	**Assessment Category**	**Score**	**Assessment Category**	**Score**
Comprehension Score Rater 1	5	Gunning Fog Score	14.2	Words	136
Comprehension Score Rater 2	5	SMOG Index	10.3	Complex words	22
Reading Age (years)	18.5	Coleman Liau Index	16.0	Percentage of complex words	16.18
Flesch-Kincaid Reading Ease	42.8	Automated Readability Index	13.7	Average words per sentence	19.43
Flesch-Kincaid Grade Level	12.1	Sentences	7	Average syllables per word	1.71

Manuscript 74

McClelland S, Maxwell PJ, Branco C, Barry ST, Eberlein C, LaBonte MJ. Targeting IL-8 and Its Receptors in Prostate Cancer: Inflammation, Stress Response, and Treatment Resistance. Cancers [Internet]. 2024 Aug 8;16(16):2797. Available from: http://dx.doi.org/10.3390/cancers16162797

Abstract

This review delves into the intricate roles of interleukin-8 (IL-8) and its receptors, CXCR1 and CXCR2, in prostate cancer (PCa), particularly in castration-resistant (CRPC) and metastatic CRPC (mCRPC). This review emphasizes the crucial role of the tumour microenvironment (TME) and inflammatory cytokines in promoting tumour progression and response to tumour cell targeting agents. IL-8, acting through C-X-C chemokine receptor type 1 (CXCR1) and type 2 (CXCR2), modulates multiple signalling pathways, enhancing the angiogenesis, proliferation, and migration of cancer cells. This review highlights the shift in PCa research focus from solely tumour cells to the non-cancer-cell components, including vascular endothelial cells, the extracellular matrix, immune cells, and the dynamic interactions within the TME. The immunosuppressive nature of the PCa TME significantly influences tumour progression and resistance to emerging therapies. Current treatment modalities, including androgen deprivation therapy and chemotherapeutics, encounter persistent resistance and are complicated by prostate cancer's notably "immune-cold" nature, which limits immune system response to the tumour. These challenges underscore the critical need for novel approaches that both overcome resistance and enhance immune engagement within the TME. The therapeutic potential of inhibiting IL-8 signalling is explored, with studies showing enhanced sensitivity of PCa cells to treatments, including radiation and androgen receptor inhibitors. Clinical trials, such as the ACE trial, demonstrate the efficacy of combining CXCR2 inhibitors with existing treatments, offering significant benefits, especially for patients with resistant PCa. This review also addresses the challenges in targeting cytokines and chemokines, noting the

complexity of the TME and the need for precision in therapeutic targeting to avoid side effects and optimize outcomes.

Keywords:

CXCR1, CXCR2, PI3K/AKT pathway, PTEN, androgen receptor, chemokines, clinical trials, cytokines, drug resistance, hypoxia, immune response, inflammation, inhibitory combinations, interleukin-8 (IL-8), monoclonal antibodies, prostate cancer, small-molecule inhibitors, tumour microenvironment

<u>Original Lay Summary</u>

Prostate cancer remains a significant health issue, particularly when it progresses to castration-resistant (CRPC) and metastatic CRPC (mCRPC). This review explores the role of interleukin-8 (IL-8) and its receptors, CXCR1 and CXCR2, in prostate cancer. IL-8 remains a key player in inflammation and tumour growth, promoting cancer cell survival, migration, and resistance to therapies. By targeting IL-8 signalling, researchers aim to develop new treatments that can enhance the effectiveness of existing therapies and improve outcomes for patients with resistant forms of prostate cancer. This approach could lead to better management of the disease and provide new hope for patients facing limited treatment options.

**Assessment Category**	**Score**	**Assessment Category**	**Score**	**Assessment Category**	**Score**
Comprehension Score Rater 1	5	Gunning Fog Score	17.5	Words	108
Comprehension Score Rater 2	4	SMOG Index	12.7	Complex words	24
Reading Age (years)	20.5	Coleman Liau Index	16.3	Percentage of complex words	22.22
Flesch-Kincaid Reading Ease	36.1	Automated Readability Index	15.0	Average words per sentence	21.6
Flesch-Kincaid Grade Level	13.6	Sentences	5	Average syllables per word	1.76

Lay Summary (Simple Prompt)

Prostate cancer, especially in its advanced stages, often becomes resistant to standard treatments like hormone therapy and chemotherapy. This study focuses on a protein called interleukin-8 (IL-8) and its receptors, which play a key role in helping cancer grow, spread, and resist treatment by influencing the environment around the tumor. IL-8 promotes inflammation, supports the formation of new blood vessels that feed tumors, and weakens the body's immune response against cancer. Researchers found that blocking IL-8 and its receptors can make prostate cancer cells more sensitive to treatments, including radiation and hormone therapies. Clinical trials have shown promising results when combining IL-8 inhibitors with existing therapies, particularly for patients with treatment-resistant cancer. By targeting IL-8, this approach could improve treatment effectiveness and help overcome resistance, offering new hope for patients with advanced prostate cancer.

**Assessment Category**	**Score**	**Assessment Category**	**Score**	**Assessment Category**	**Score**
Comprehension Score Rater 1	5	Gunning Fog Score	17.9	Words	141
Comprehension Score Rater 2	5	SMOG Index	12.9	Complex words	30
Reading Age (years)	21.5	Coleman Liau Index	17.6	Percentage of complex words	21.28
Flesch-Kincaid Reading Ease	32.4	Automated Readability Index	17.1	Average words per sentence	23.5
Flesch-Kincaid Grade Level	14.6	Sentences	6	Average syllables per word	1.78

Lay Summary (Extended Prompt)

Prostate cancer can become very hard to treat, especially when it spreads or stops responding to standard therapies. This research focuses on a protein called interleukin-8 (IL-8) and its receptors, which play a key role in how prostate cancer grows, spreads, and resists treatment. IL-8 helps cancer cells survive by creating an environment that protects them from the body's immune system and common cancer treatments. Scientists are now looking at ways to block IL-8 and its receptors to make prostate cancer treatments more effective. Studies show that targeting IL-8 can slow down cancer growth, make cancer cells more sensitive to therapies, and improve the immune system's ability to fight the disease. Some clinical trials are already testing new drugs that block IL-8, showing promising results for patients with advanced prostate cancer. This research could lead to better treatments and improved outcomes for people with prostate cancer.

**Assessment Category**	**Score**	**Assessment Category**	**Score**	**Assessment Category**	**Score**
Comprehension Score Rater 1	5	Gunning Fog Score	12.6	Words	152
Comprehension Score Rater 2	5	SMOG Index	8.6	Complex words	15
Reading Age (years)	17.5	Coleman Liau Index	14.1	Percentage of complex words	9.87
Flesch-Kincaid Reading Ease	55.1	Automated Readability Index	13.4	Average words per sentence	21.71
Flesch-Kincaid Grade Level	11.0	Sentences	7	Average syllables per word	1.53

Manuscript 75

Marsland M, Jiang CC, Faulkner S, Steigler A, McEwan K, Jobling P, et al. CCL2/CCR2 Expression in Locally Advanced Prostate Cancer and Patient Long-Term Outcome: 10-Year Results from the TROG 03.04 RADAR Trial. Cancers [Internet]. 2024 Aug 8;16(16):2794. Available from: http://dx.doi.org/10.3390/cancers16162794

Abstract

This study investigated the prognostic value of the chemokine C-C motif ligand 2 (CCL2) and its receptor C-C motif chemokine receptor 2 (CCR2) expression in locally advanced prostate cancer treated with radiotherapy and androgen deprivation using the 10-year outcome data from the TROG 03.04 RADAR clinical trial. CCL2 and CCR2 protein expression in prostate cancer biopsies at the time of diagnosis were quantified by immunohistochemistry and digital quantification. CCR2 protein expression was detected in prostate cancer cells and was associated with prostate-specific antigen serum concentration ($p = 0.045$). However, neither CCL2 nor CCR2 tissue expression could predict prostate cancer progression, or other clinicopathological parameters including perineural invasion and patient outcome. In serum samples, CCL2 concentration at the time of diagnosis, as assayed by enzyme-linked immunosorbent assay, was significantly higher in patients with prostate cancer compared with benign prostatic hyperplasia (median difference 0.22 ng/mL, 95% CI, 0.17-0.30) (p $p < 0.0001$). However, circulating CCL2 was not statistically significant as a predictor of disease progression and patient outcome. In conclusion, this study shows that although CCL2 and CCR2 are expressed in prostate cancer, with an increased level of CCL2 in the serum, neither CCL2 nor CCR2 expression has a clinical prognostic value in locally advanced prostate cancer.

Keywords:

CCL2, CCR2, RADAR trial, cancer biomarker, prostate cancer

<u>Original Lay Summary</u>

Prostate cancer is the second most common cancer among males and is a significant cause of morbidity and mortality. The chemokine C-C motif ligand 2 (CCL2) and its receptor C-C motif chemokine receptor 2 (CCR2) are expressed in prostate cancer but their prognostic value is unclear. This study investigated CCL2 and CCR2 as prognostic biomarkers in locally advanced prostate cancer using the large TROG 03.04 RADAR clinical trial cohort. The results demonstrate that despite being expressed in prostate tumours, CCR2 and CCL2 have no prognostic value in the disease.

**Assessment Category**	**Score**	**Assessment Category**	**Score**	**Assessment Category**	**Score**
Comprehension Score Rater 1	5	Gunning Fog Score	13.9	Words	92
Comprehension Score Rater 2	4	SMOG Index	10.1	Complex words	15
Reading Age (years)	17.5	Coleman Liau Index	13.2	Percentage of complex words	16.3
Flesch-Kincaid Reading Ease	43.8	Automated Readability Index	11.0	Average words per sentence	18.4
Flesch-Kincaid Grade Level	11.7	Sentences	5	Average syllables per word	1.71

Lay Summary (Simple Prompt)

Prostate cancer is one of the most common cancers in men, and finding markers that can help predict how the disease will progress is important for improving treatment decisions. This study looked at two specific proteins, CCL2 and CCR2, which are involved in immune responses, to see if their levels in prostate cancer tissue and blood could predict patient outcomes. Using data from the TROG 03.04 RADAR clinical trial, which followed patients for 10 years, researchers measured these proteins in prostate cancer samples and blood tests. Although higher levels of CCL2 were found in the blood of men with prostate cancer compared to those with non-cancerous conditions, neither CCL2 nor CCR2 levels were linked to cancer progression or long-term outcomes. This means that while these proteins are present in prostate cancer, they are not useful as biomarkers to predict how the disease will behave in the long term.

**Assessment Category**	**Score**	**Assessment Category**	**Score**	**Assessment Category**	**Score**
Comprehension Score Rater 1	5	Gunning Fog Score	11.7	Words	156
Comprehension Score Rater 2	4	SMOG Index	7.4	Complex words	11
Reading Age (years)	16.5	Coleman Liau Index	13.4	Percentage of complex words	7.05
Flesch-Kincaid Reading Ease	58.9	Automated Readability Index	13.1	Average words per sentence	22.29
Flesch-Kincaid Grade Level	10.6	Sentences	7	Average syllables per word	1.48

<u>Lay Summary (Extended Prompt)</u>

Prostate cancer is one of the most common cancers in men, and finding ways to predict how it will progress can help improve treatment. This study looked at two proteins, CCL2 and CCR2, which are present in prostate cancer cells and blood. Researchers wanted to see if the levels of these proteins could help predict how the cancer would develop over time. They analyzed samples from men with locally advanced prostate cancer who were part of a large clinical trial called RADAR. Although the proteins were found in cancer tissues and blood, their levels did not help predict if the cancer would get worse or how patients would respond to treatment. This means that CCL2 and CCR2 are not useful markers for forecasting long-term outcomes in these patients. Understanding what does and doesn't work as a predictor can help guide future research to improve prostate cancer care.

**Assessment Category**	**Score**	**Assessment Category**	**Score**	**Assessment Category**	**Score**
Comprehension Score Rater 1	5	Gunning Fog Score	10.6	Words	148
Comprehension Score Rater 2	5	SMOG Index	6.4	Complex words	8
Reading Age (years)	15.5	Coleman Liau Index	12.5	Percentage of complex words	5.41
Flesch-Kincaid Reading Ease	65.9	Automated Readability Index	11.8	Average words per sentence	21.14
Flesch-Kincaid Grade Level	9.3	Sentences	7	Average syllables per word	1.41

Manuscript 76

Rahman M, Akter K, Ahmed KR, Fahim MdMH, Aktary N, Park MN, et al. Synergistic Strategies for Castration-Resistant Prostate Cancer: Targeting AR-V7, Exploring Natural Compounds, and Optimizing FDA-Approved Therapies. Cancers [Internet]. 2024 Aug 6;16(16):2777. Available from: http://dx.doi.org/10.3390/cancers16162777

Abstract

Castration-resistant prostate cancer (CRPC) remains a significant therapeutic challenge due to its resistance to standard androgen deprivation therapy (ADT). The emergence of androgen receptor splice variant 7 (AR-V7) has been implicated in CRPC progression, contributing to treatment resistance. Current treatments, including first-generation chemotherapy, androgen receptor blockers, radiation therapy, immune therapy, and PARP inhibitors, often come with substantial side effects and limited efficacy. Natural compounds, particularly those derived from herbal medicine, have garnered increasing interest as adjunctive therapeutic agents against CRPC. This review explores the role of AR-V7 in CRPC and highlights the promising benefits of natural compounds as complementary treatments to conventional drugs in reducing CRPC and overcoming therapeutic resistance. We delve into the mechanisms of action underlying the anti-CRPC effects of natural compounds, showcasing their potential to enhance therapeutic outcomes while mitigating the side effects associated with conventional therapies. The exploration of natural compounds offers promising avenues for developing novel treatment strategies that enhance therapeutic outcomes and reduce the adverse effects of conventional CRPC therapies. These compounds provide a safer, more effective approach to managing CRPC, representing a significant advancement in improving patient care.

Keywords:

androgen receptor blockers, androgen receptor splice variant 7, castration-resistant prostate cancer, herbal medicine, natural compounds

<u>Original Lay Summary</u>

Castration-resistant prostate cancer (CRPC) resists standard therapies, partly due to the androgen receptor variant AR-V7. Conventional treatments like chemotherapy and radiation have limited success and significant side effects. Natural compounds from herbal medicine offer promise as complementary therapies. This review examines how these natural compounds can improve treatment outcomes and reduce side effects, presenting a safer and more effective approach to managing CRPC and overcoming resistance to conventional therapies.

**Assessment Category**	**Score**	**Assessment Category**	**Score**	**Assessment Category**	**Score**
Comprehension Score Rater 1	5	Gunning Fog Score	20.1	Words	71
Comprehension Score Rater 2	3	SMOG Index	14.1	Complex words	24
Reading Age (years)	23.5	Coleman Liau Index	21.8	Percentage of complex words	33.8
Flesch-Kincaid Reading Ease	12.5	Automated Readability Index	17.5	Average words per sentence	17.75
Flesch-Kincaid Grade Level	15.9	Sentences	4	Average syllables per word	2.08

Lay Summary (Simple Prompt)

Prostate cancer that no longer responds to standard hormone therapy, known as castration-resistant prostate cancer (CRPC), is difficult to treat. A protein called AR-V7 has been linked to the cancer's ability to resist treatment, making it harder to control. While current therapies like chemotherapy, radiation, and hormone blockers can help, they often cause serious side effects and may lose effectiveness over time. This study explores new ways to manage CRPC by focusing on AR-V7 and investigating natural compounds, such as those found in herbal medicines, as additional treatment options. These natural compounds may work alongside existing drugs to improve treatment results and reduce side effects. By understanding how these compounds affect prostate cancer cells, researchers aim to develop safer, more effective strategies for patients with CRPC. This approach could lead to better outcomes and a higher quality of life for those living with advanced prostate cancer.

**Assessment Category**	**Score**	**Assessment Category**	**Score**	**Assessment Category**	**Score**
Comprehension Score Rater 1	5	Gunning Fog Score	15.0	Words	149
Comprehension Score Rater 2	5	SMOG Index	10.7	Complex words	24
Reading Age (years)	19.5	Coleman Liau Index	16.2	Percentage of complex words	16.11
Flesch-Kincaid Reading Ease	42.7	Automated Readability Index	14.8	Average words per sentence	21.29
Flesch-Kincaid Grade Level	12.6	Sentences	7	Average syllables per word	1.68

<u>Lay Summary (Extended Prompt)</u>

Castration-resistant prostate cancer (CRPC) is a tough form of prostate cancer that doesn't respond well to standard treatments, like hormone therapy. A key reason for this is a protein called AR-V7, which helps the cancer grow even when hormone levels are low. Current treatments, such as chemotherapy and radiation, can have strong side effects and don't always work well. This study looks at new ways to fight CRPC by focusing on AR-V7 and exploring natural compounds, especially from herbal medicine, that might help make treatments more effective. These natural compounds could work alongside regular medicines to reduce cancer growth and help patients feel better by lowering side effects. By combining these strategies, this research aims to find safer, more effective treatments for people with CRPC, offering new hope for better care and longer, healthier lives.

**Assessment Category**	**Score**	**Assessment Category**	**Score**	**Assessment Category**	**Score**
Comprehension Score Rater 1	5	Gunning Fog Score	14.7	Words	138
Comprehension Score Rater 2	5	SMOG Index	10.6	Complex words	20
Reading Age (years)	18.5	Coleman Liau Index	14.6	Percentage of complex words	14.49
Flesch-Kincaid Reading Ease	51.1	Automated Readability Index	14.4	Average words per sentence	23.0
Flesch-Kincaid Grade Level	11.8	Sentences	6	Average syllables per word	1.57

Manuscript 77

Bouchareb E, Dallel S, De Haze A, Damon-Soubeyrand C, Renaud Y, Baabdaty E, et al. Liver X Receptors Enhance Epithelial to Mesenchymal Transition in Metastatic Prostate Cancer Cells. Cancers [Internet]. 2024 Aug 6;16(16):2776. Available from: http://dx.doi.org/10.3390/cancers16162776

Abstract

Prostate cancer (PCa) is one of the most common cancers in men. Metastasis is the leading cause of death in prostate cancer patients. One of the crucial processes involved in metastatic spread is the "epithelial-mesenchymal transition" (EMT), which allows cells to acquire the ability to invade distant organs. Liver X Receptors (LXRs) are nuclear receptors that have been demonstrated to regulate EMT in various cancers, including hepatic cancer. Our study reveals that the LXR pathway can control pro-invasive cell capacities through EMT in prostate cancer, employing ex vivo and in vivo approaches. We characterized the EMT status of the commonly used LNCaP, DU145, and PC3 prostate cancer cell lines through molecular and immunohistochemistry experiments. The impact of LXR activation on EMT function was also assessed by analyzing the migration and invasion of these cell lines in the absence or presence of an LXR agonist. Using in vivo experiments involving NSG-immunodeficient mice xenografted with PC3-GFP cells, we were able to study metastatic spread and the effect of LXRs on this process. LXR activation led to an increase in the accumulation of Vimentin and Amphiregulin in PC3. Furthermore, the migration of PC3 cells significantly increased in the presence of the LXR agonist, correlating with an upregulation of EMT. Interestingly, LXR activation significantly increased metastatic spread in an NSG mouse model. Overall, this work identifies a promoting effect of LXRs on EMT in the PC3 model of advanced prostate cancer.

Keywords:

epithelial–mesenchymal transition, liver X receptors, metastasis, prostate cancer

<u>Original Lay Summary</u>

As many cancers, prostate cancer is lethal when reaching the metastatic state. Starting from prostate to invade secondary sites, tumour cells undergo structural modifications called epithelial-mesenchymal transition. Molecular mechanisms controlling this phenomenon were studied in vitro and in vivo after modification of LXRs transcription factors activity, which regulate cholesterol metabolism. These studies showed that in a metastatic cell line derived from bone, increased activity of LXR stimulated the EMT processes in vitro and in vivo. We then characterized the associated deregulated genes and identified Amphiregulin as a new target regulated by LXR. The relationship between amphiregulin and EMT was here first reported in prostate cancer and as this protein can be secreted, will be further considered as a possible blood maker for monitoring metastatic occurrence.

**Assessment Category**	**Score**	**Assessment Category**	**Score**	**Assessment Category**	**Score**
Comprehension Score Rater 1	5	Gunning Fog Score	19.8	Words	126
Comprehension Score Rater 2	4	SMOG Index	14.3	Complex words	37
Reading Age (years)	22.5	Coleman Liau Index	18.9	Percentage of complex words	29.37
Flesch-Kincaid Reading Ease	12.3	Automated Readability Index	16.8	Average words per sentence	21.0
Flesch-Kincaid Grade Level	16.8	Sentences	6	Average syllables per word	2.05

Lay Summary (Simple Prompt)

Prostate cancer is one of the most common cancers in men, and its spread to other parts of the body (metastasis) is the main cause of death. A key process that helps cancer cells spread is called epithelial-mesenchymal transition (EMT), which allows these cells to become more mobile and invasive. This study focused on liver X receptors (LXRs), proteins that can influence how cells behave. The researchers investigated how activating LXRs affects prostate cancer cells, using laboratory experiments with cancer cell lines and mouse models. They found that turning on LXRs made the cancer cells more invasive and increased their ability to spread in mice. This suggests that LXRs may play a role in promoting prostate cancer metastasis. Understanding this process could help scientists develop new strategies to prevent the spread of prostate cancer, potentially leading to better treatment options in the future.

**Assessment Category**	**Score**	**Assessment Category**	**Score**	**Assessment Category**	**Score**
Comprehension Score Rater 1	5	Gunning Fog Score	13.5	Words	144
Comprehension Score Rater 2	4	SMOG Index	9.6	Complex words	19
Reading Age (years)	18.5	Coleman Liau Index	15.0	Percentage of complex words	13.19
Flesch-Kincaid Reading Ease	47.9	Automated Readability Index	13.5	Average words per sentence	20.57
Flesch-Kincaid Grade Level	11.7	Sentences	7	Average syllables per word	1.63

Lay Summary (Extended Prompt)

Prostate cancer is one of the most common cancers in men, and when it spreads to other parts of the body (metastasis), it becomes much harder to treat. For cancer to spread, cells go through a change called "epithelial-mesenchymal transition" (EMT), which helps them move and invade new areas. Our study looked at special proteins called Liver X Receptors (LXRs) to see how they affect this process in prostate cancer cells. We tested this in lab-grown cells and in mice with prostate cancer. We found that when LXRs were activated, the cancer cells became better at moving and spreading, showing more signs of EMT. This means that LXRs might play an important role in how prostate cancer spreads. Understanding this could help scientists develop new ways to stop cancer from spreading, which might lead to better treatments for patients with advanced prostate cancer.

**Assessment Category**	**Score**	**Assessment Category**	**Score**	**Assessment Category**	**Score**
Comprehension Score Rater 1	5	Gunning Fog Score	10.2	Words	145
Comprehension Score Rater 2	4	SMOG Index	6.7	Complex words	9
Reading Age (years)	15.5	Coleman Liau Index	12.7	Percentage of complex words	6.21
Flesch-Kincaid Reading Ease	66.2	Automated Readability Index	11.7	Average words per sentence	20.71
Flesch-Kincaid Grade Level	9.2	Sentences	7	Average syllables per word	1.41

Manuscript 78

Falkenbach F, Di Bello F, Rodriguez Peñaranda N, Longoni M, Marmiroli A, Le QC, et al. Adverse In-Hospital Outcomes after Radical Prostatectomy in Leukemia History Patients. Cancers [Internet]. 2024 Aug 5;16(15):2764. Available from: http://dx.doi.org/10.3390/cancers16152764

Abstract

Introduction: Leukemia history affects some radical prostatectomy (RP) patients. Although its prevalence and effect as an adverse risk factor are well known in cardiac surgery, the number of RP patients with a leukemia history, as well as their rate of adverse in-hospital outcomes, are unknown.

Methods: We identified RP patients (National Inpatient Sample 2000-2019), stratified according to the presence or absence of a leukemia history. Descriptive analyses, propensity score matching (PSM, ratio 1:10), and multivariable logistic regression models were used.

Results: Of 259,939 RP patients, 416 (0.2%) had a leukemia history. Their proportion increased from 0.1 to 0.2% covering the study span ($p < 0.01$). Leukemia history patients were older (median age, 64 vs. 62 years, $p < 0.001$). After PSM for age, insurance status, ethnicity, pelvic lymph node dissection, and Charlson Comorbidity Index, leukemia history RP patients exhibited higher rates of acute kidney injury (<2.6 vs. 0.9%; Odds Ratio [OR] 2.0, $p = 0.02$), more frequently underwent dialysis (3.6 vs. 1.9%; OR 1.9, $p = 0.03$), and more frequently had a length of stay exceeding one week (4.8 vs. 2.5%; OR 2.0, $p = 0.006$).

Conclusions: Although leukemia history RP patients are rare, their numbers have increased. Renal complications and extended hospital stays are more frequent in those individuals.

Keywords:

CLL, NIS, leukemia, pelvic lymph node dissection, prostate cancer, prostatectomy

<u>Original Lay Summary</u>

Leukemia history affects some radical prostatectomy (RP) patients. While its prevalence and impact as an adverse risk factor are well-known in cardiac surgery, the number of RP patients with a leukemia history and their rate of adverse in-hospital outcomes are unknown. We identified RP patients with a leukemia history in a large-scale database. Patients with a leukemia history had higher rates of acute kidney injury, more frequent dialysis, and extended hospital stays compared to those without a leukemia history.

**Assessment Category**	**Score**	**Assessment Category**	**Score**	**Assessment Category**	**Score**
Comprehension Score Rater 1	5	Gunning Fog Score	18.0	Words	82
Comprehension Score Rater 2	4	SMOG Index	13.2	Complex words	21
Reading Age (years)	20.5	Coleman Liau Index	14.9	Percentage of complex words	25.61
Flesch-Kincaid Reading Ease	29.2	Automated Readability Index	13.4	Average words per sentence	10.5
Flesch-Kincaid Grade Level	14.3	Sentences	4	Average syllables per word	1.85

Lay Summary (Simple Prompt)

Some men with prostate cancer who undergo surgery to remove the prostate (radical prostatectomy) also have a history of leukemia. While the risks associated with leukemia are well known in heart surgery, it is unclear how leukemia affects recovery after prostate surgery. This study analyzed data from nearly 260,000 prostate surgery patients, including 416 with a history of leukemia. The researchers found that the number of such cases has increased over the years. Compared to other patients, those with a history of leukemia were slightly older and more likely to experience complications like acute kidney injury, the need for dialysis, and longer hospital stays. These findings highlight the need for extra care and monitoring of prostate cancer patients with a history of leukemia to improve their outcomes after surgery. Understanding these risks can help doctors make better decisions and provide more tailored care for these patients.

**Assessment Category**	**Score**	**Assessment Category**	**Score**	**Assessment Category**	**Score**
Comprehension Score Rater 1	5	Gunning Fog Score	16.8	Words	147
Comprehension Score Rater 2	5	SMOG Index	12.2	Complex words	31
Reading Age (years)	19.5	Coleman Liau Index	15.2	Percentage of complex words	21.09
Flesch-Kincaid Reading Ease	33.6	Automated Readability Index	13.9	Average words per sentence	21.0
Flesch-Kincaid Grade Level	13.8	Sentences	7	Average syllables per word	1.8

Lay Summary (Extended Prompt)

Patients who have had leukemia and later undergo surgery to remove their prostate (called radical prostatectomy) may face higher risks of complications during their hospital stay. While it is known that leukemia can increase risks in heart surgeries, little was known about its effects in prostate surgeries. In this study, we looked at hospital data from nearly 260,000 men who had prostate surgery between 2000 and 2019. We found that only a small number (0.2%) had a history of leukemia, but this number has been growing over the years. Compared to other patients, those with a leukemia history were more likely to develop kidney problems, need dialysis, and stay in the hospital longer. These results show that doctors should be aware of these risks when treating prostate cancer patients with a history of leukemia, to provide better care and possibly prevent complications.

**Assessment Category**	**Score**	**Assessment Category**	**Score**	**Assessment Category**	**Score**
Comprehension Score Rater 1	5	Gunning Fog Score	14.6	Words	144
Comprehension Score Rater 2	5	SMOG Index	10.5	Complex words	23
Reading Age (years)	17.5	Coleman Liau Index	12.9	Percentage of complex words	15.97
Flesch-Kincaid Reading Ease	46.7	Automated Readability Index	11.8	Average words per sentence	20.57
Flesch-Kincaid Grade Level	11.9	Sentences	7	Average syllables per word	1.65

Manuscript 79

Fekete B, Biró K, Gyergyay F, Polk N, Horváth O, Géczi L, et al. Prediction of PSA Response after Dexamethasone Switch during Abiraterone Acetate + Prednisolone Treatment of Metastatic Castration-Resistant Prostate Cancer Patients. Cancers [Internet]. 2024 Aug 3;16(15):2760. Available from: http://dx.doi.org/10.3390/cancers16152760

Abstract

Background: The aim was to elaborate a predictive model to find responders for the corticosteroid switch (from prednisolone to dexamethasone) at the first prostate-specific antigen (PSA) progression (≥25% increase) during abiraterone acetate (AA) treatment of metastatic castration-resistant prostate cancer (mCRPC) patients.

Methods: If PSA has decreased (≥25%) after switch, patients were considered responders. Logistic regression of 19 dichotomized parameters from routine laboratory and patients' history was used to find the best model in a cohort of 67 patients. The model was validated in another cohort of 42 patients.

Results: The model provided 92.5% and 90.5% accuracy in the testing and the validation cohorts, respectively. Overall the accuracy was 91.7%. The AUC of ROC curve was 0.92 (95% CI 0.85-0.96). After a median follow-up of 27.9 (26.3-84) months, the median AA+dexamethasone treatment duration (TD) in non-responders and responders was 4.7 (3.1-6.5) and 11.1 (8.5-12.9) months and the median overall survival (OS) was 23.2 (15.6-25.8) and 33.5 (26.1-38) months, respectively. Multivariate Cox regression revealed that responsiveness was an independent marker of TD and OS.

Conclusions: A high accuracy model was developed for mCRPC patients in predicting cases which might benefit from the switch. For non-responders, induction of the next systemic treatment is indicated.

Keywords:
corticosteroid switch, overall survival, predictive model, responders, treatment duration

<u>Original Lay Summary</u>

A model predicting responders to corticosteroid switch during abiraterone therapy in patients with metastatic castration-resistant prostate cancer is warranted. The model using logistic regression was developed in a cohort of 67 and validated in another cohort of 42 patients. The use of this model is easy and all needed parameters are available from routine laboratory and history of patients. The overall accuracy was 92%. Multivariate analysis revealed that responsiveness was a marker of longer treatment duration and overall survival. For non-responders a new systemic treatment was indicated.

**Assessment Category**	**Score**	**Assessment Category**	**Score**	**Assessment Category**	**Score**
Comprehension Score Rater 1	4	Gunning Fog Score	19.0	Words	89
Comprehension Score Rater 2	4	SMOG Index	12.7	Complex words	29
Reading Age (years)	20.5	Coleman Liau Index	17.1	Percentage of complex words	32.58
Flesch-Kincaid Reading Ease	16.9	Automated Readability Index	12.3	Average words per sentence	14.83
Flesch-Kincaid Grade Level	14.6	Sentences	6	Average syllables per word	2.07

Lay Summary (Simple Prompt)

In men with advanced prostate cancer that no longer responds to hormone therapy, treatment often includes a drug called abiraterone acetate combined with a steroid called prednisolone. When this treatment stops working, doctors sometimes switch the steroid from prednisolone to dexamethasone, which can lower prostate-specific antigen (PSA) levels in some patients. However, not all patients benefit from this switch. This study aimed to develop a model to predict which patients are likely to respond to the steroid change. Researchers analyzed medical data from 109 patients and identified key factors that could help predict a positive response. The model was highly accurate, correctly identifying responders in over 90% of cases. Patients who responded to the switch lived longer and had better treatment outcomes. This tool could help doctors make more informed decisions, ensuring patients receive the most effective treatment at the right time.

**Assessment Category**	**Score**	**Assessment Category**	**Score**	**Assessment Category**	**Score**
Comprehension Score Rater 1	5	Gunning Fog Score	14.1	Words	143
Comprehension Score Rater 2	5	SMOG Index	10.3	Complex words	25
Reading Age (years)	18.5	Coleman Liau Index	16.6	Percentage of complex words	17.48
Flesch-Kincaid Reading Ease	43.2	Automated Readability Index	13.5	Average words per sentence	17.88
Flesch-Kincaid Grade Level	11.7	Sentences	8	Average syllables per word	1.72

<u>Lay Summary (Extended Prompt)</u>

When treating advanced prostate cancer, some patients stop responding to a common drug combination of abiraterone acetate and prednisolone. In these cases, doctors sometimes switch the steroid from prednisolone to dexamethasone, but it's hard to predict who will benefit from this change. Our study aimed to create a simple model to help doctors identify which patients are likely to respond to this switch. We looked at the medical history and routine lab results of 67 patients to find key factors linked to treatment success. We then tested the model in another group of 42 patients to make sure it worked well. The model was very accurate, correctly predicting outcomes in over 90% of cases. Patients who responded to the switch lived longer and stayed on treatment for more time. This model can help doctors make better treatment decisions, ensuring patients get the most effective care at the right time.

**Assessment Category**	**Score**	**Assessment Category**	**Score**	**Assessment Category**	**Score**
Comprehension Score Rater 1	5	Gunning Fog Score	12.6	Words	149
Comprehension Score Rater 2	5	SMOG Index	9.0	Complex words	19
Reading Age (years)	16.5	Coleman Liau Index	13.5	Percentage of complex words	12.75
Flesch-Kincaid Reading Ease	56.2	Automated Readability Index	11.3	Average words per sentence	18.63
Flesch-Kincaid Grade Level	10.0	Sentences	8	Average syllables per word	1.56

Manuscript 80

Santucci D, Ragone R, Vergantino E, Vaccarino F, Esperto F, Prata F, et al. Comparison between Three Radiomics Models and Clinical Nomograms for Prediction of Lymph Node Involvement in PCa Patients Combining Clinical and Radiomic Features. Cancers [Internet]. 2024 Jul 31;16(15):2731. Available from: http://dx.doi.org/
10.3390/cancers16152731

Abstract

Purpose: We aim to compare the performance of three different radiomics models (logistic regression (LR), random forest (RF), and support vector machine (SVM)) and clinical nomograms (Briganti, MSKCC, Yale, and Roach) for predicting lymph node involvement (LNI) in prostate cancer (PCa) patients.

Materials and methods: The retrospective study includes 95 patients who underwent mp-MRI and radical prostatectomy for PCa with pelvic lymphadenectomy. Imaging data (intensity in T2, DWI, ADC, and PIRADS), clinical data (age and pre-MRI PSA), histological data (Gleason score, TNM staging, histological type, capsule invasion, seminal vesicle invasion, and neurovascular bundle involvement), and clinical nomograms (Yale, Roach, MSKCC, and Briganti) were collected for each patient. Manual segmentation of the index lesions was performed for each patient using an open-source program (3D SLICER). Radiomic features were extracted for each segmentation using the Pyradiomics library for each sequence (T2, DWI, and ADC). The features were then selected and used to train and test three different radiomics models (LR, RF, and SVM) independently using ChatGPT software (v 4o). The coefficient value of each feature was calculated (significant value for coefficient ≥ ±0.5). The predictive performance of the radiomics models and clinical nomograms was assessed using accuracy and area under the curve (AUC) (significant value for $p \leq 0.05$). Thus, the diagnostic accuracy between the radiomics and clinical models were compared.

Results: This study identified 343 features per patient (330 radiomics features and 13 clinical features). The most significant features were T2_nodulofirstordervariance and T2_nodulofirstorderkurtosis. The highest predictive performance was achieved by the RF model with DWI (accuracy 86%, AUC 0.89) and ADC (accuracy 89%, AUC 0.67). Clinical nomograms demonstrated satisfactory but lower predictive performance compared to the RF model in the DWI sequences.

Conclusions: Among the prediction models developed using integrated data (radiomics and semantics), RF shows slightly higher diagnostic accuracy in terms of AUC compared to clinical nomograms in PCa lymph node involvement prediction.
Keywords:

ChatGPT, artificial intelligence, clinical nomogram, logistic regression (LR), multiparametric magnetic resonance imaging (mp-MRI), prostate cancer (PCa), random forest (RF), support vector machine (SVM)

<u>Original Lay Summary</u>

Our study aims to demonstrate and quantify the usefulness of three different AI models for lymph node involvement prediction in prostate cancer patients comparing the performance with current routinely used clinical nomograms. Knowing the lymph node involvement with accuracy and confidence can influence the type of surgical and therapeutic choice.

**Assessment Category**	**Score**	**Assessment Category**	**Score**	**Assessment Category**	**Score**
Comprehension Score Rater 1	4	Gunning Fog Score	22.8	Words	50
Comprehension Score Rater 2	3	SMOG Index	16.3	Complex words	16
Reading Age (years)	24.0	Coleman Liau Index	19.3	Percentage of complex words	32.0
Flesch-Kincaid Reading Ease	20.7	Automated Readability Index	19.1	Average words per sentence	25.0
Flesch-Kincaid Grade Level	16.6	Sentences	2	Average syllables per word	1.9

<u>Lay Summary (Simple Prompt)</u>

Prostate cancer can spread to nearby lymph nodes, which affects how the disease is treated. Accurately predicting whether cancer has reached the lymph nodes is crucial for deciding the best treatment. In this study, we compared advanced computer-based models, called radiomics models, with traditional clinical tools (known as nomograms) to see which predicts lymph node involvement more accurately. We analyzed data from prostate cancer patients, including medical imaging, clinical details, and laboratory results. Among the models tested, the random forest model—an artificial intelligence method—performed better than the traditional tools, offering more precise predictions. This means that using such advanced computer models could help doctors make more informed decisions about prostate cancer treatment, potentially leading to better outcomes for patients.

**Assessment Category**	**Score**	**Assessment Category**	**Score**	**Assessment Category**	**Score**
Comprehension Score Rater 1	5	Gunning Fog Score	16.7	Words	120
Comprehension Score Rater 2	5	SMOG Index	12.0	Complex words	26
Reading Age (years)	21.5	Coleman Liau Index	19.8	Percentage of complex words	21.67
Flesch-Kincaid Reading Ease	29.3	Automated Readability Index	17.1	Average words per sentence	20.0
Flesch-Kincaid Grade Level	14.1	Sentences	6	Average syllables per word	1.86

Lay Summary (Extended Prompt)

Prostate cancer can spread to nearby lymph nodes, which makes the disease more serious. Doctors usually predict this spread using clinical tools called nomograms, but these tools are not always accurate. In this study, we tested three advanced computer models, known as radiomics models, to see if they could do a better job. We analyzed medical images and clinical data from 95 prostate cancer patients who had surgery to remove their prostate and lymph nodes. Our results showed that one of the computer models, called the random forest model, predicted lymph node involvement more accurately than the traditional clinical tools. This means that combining medical imaging with advanced computer analysis could help doctors make better decisions about treatment. Using these models in the future may improve how we identify patients who need more aggressive care, leading to better outcomes.

**Assessment Category**	**Score**	**Assessment Category**	**Score**	**Assessment Category**	**Score**
Comprehension Score Rater 1	5	Gunning Fog Score	15.1	Words	139
Comprehension Score Rater 2	5	SMOG Index	11.0	Complex words	25
Reading Age (years)	18.5	Coleman Liau Index	15.4	Percentage of complex words	17.99
Flesch-Kincaid Reading Ease	46.1	Automated Readability Index	13.5	Average words per sentence	19.86
Flesch-Kincaid Grade Level	11.8	Sentences	7	Average syllables per word	1.66

Manuscript 81

Lee H, Thakker S, Pineault K, Wysock J, Tan WP. Salvage Cryoablation for Recurrent Prostate Cancer Following Radiation—A Comprehensive Review. Cancers [Internet]. 2024 Jul 31;16(15):2717. Available from: http://dx.doi.org/10.3390/cancers16152717

Abstract

The treatment options for prostate cancer typically entail active surveillance, surgery, radiation, or a combination of the above. Disease recurrence remains a concern, with a wide range of recurrence rates having been reported in the literature. In the setting of recurrence, the salvage treatment options include salvage prostatectomy, salvage high-intensity focused ultrasound (HIFU), stereotactic body radiotherapy (SBRT), salvage brachytherapy, and salvage cryoablation. In this review, we analyze the currently available data related to salvage cryoablation for recurrent prostate cancer following radiation.

Keywords:

cryoablation, cryotherapy, focal therapy, partial-gland ablation, prostate cancer, prostate cancer recurrence, radiation

Original Lay Summary

Apart from skin cancer, prostate cancer is the most prevalent cancer in men. The treatment options typically entail active surveillance, surgery, radiation, or a combination of the above. The treatment options for recurrent disease also include surgery, radiation, and focal therapy. In this review, we look at the use of cryoablation for recurrent prostate cancer following radiation treatment.

**Assessment Category**	**Score**	**Assessment Category**	**Score**	**Assessment Category**	**Score**
Comprehension Score Rater 1	4	Gunning Fog Score	15.5	Words	58
Comprehension Score Rater 2	3	SMOG Index	10.8	Complex words	14
Reading Age (years)	19.5	Coleman Liau Index	17.4	Percentage of complex words	24.14
Flesch-Kincaid Reading Ease	30.2	Automated Readability Index	12.4	Average words per sentence	14.5
Flesch-Kincaid Grade Level	12.6	Sentences	4	Average syllables per word	1.91

Lay Summary (Simple Prompt)

Prostate cancer can return even after treatments like surgery or radiation. When this happens, doctors need effective options to treat the cancer again, known as salvage treatments. One such option is salvage cryoablation, a procedure that uses extreme cold to destroy cancer cells. This review looks at the current research on salvage cryoablation for men whose prostate cancer has come back after radiation therapy. We aimed to understand how well this treatment works, its potential risks, and how it compares to other salvage treatments. By analyzing the available data, we hope to help doctors and patients make informed decisions about using salvage cryoablation as a treatment option. This information could improve care for men facing recurrent prostate cancer and guide future research in this field.

**Assessment Category**	**Score**	**Assessment Category**	**Score**	**Assessment Category**	**Score**
Comprehension Score Rater 1	4	Gunning Fog Score	12.3	Words	125
Comprehension Score Rater 2	3	SMOG Index	8.8	Complex words	16
Reading Age (years)	17.5	Coleman Liau Index	15.8	Percentage of complex words	12.8
Flesch-Kincaid Reading Ease	52.0	Automated Readability Index	12.8	Average words per sentence	17.86
Flesch-Kincaid Grade Level	10.4	Sentences	7	Average syllables per word	1.62

Lay Summary (Extended Prompt)

When prostate cancer comes back after radiation treatment, finding the right next step can be challenging. One option is salvage cryoablation, a procedure that freezes and destroys cancer cells in the prostate. This review looks at all the current research on how well salvage cryoablation works for men with recurrent prostate cancer after radiation. We explore how safe it is, how effective it can be in controlling the cancer, and what side effects patients might experience. By gathering and analyzing the available data, we aim to help doctors and patients make better decisions about treatment options when prostate cancer returns. Our findings may also guide future research to improve outcomes for men facing this difficult situation.

**Assessment Category**	**Score**	**Assessment Category**	**Score**	**Assessment Category**	**Score**
Comprehension Score Rater 1	4	Gunning Fog Score	13.3	Words	116
Comprehension Score Rater 2	3	SMOG Index	9.5	Complex words	16
Reading Age (years)	18.5	Coleman Liau Index	15.5	Percentage of complex words	13.79
Flesch-Kincaid Reading Ease	49.4	Automated Readability Index	13.2	Average words per sentence	19.33
Flesch-Kincaid Grade Level	11.2	Sentences	6	Average syllables per word	1.63

Manuscript 82

El Khoury CJ. Application of Geographic Information Systems (GIS) in the Study of Prostate Cancer Disparities: A Systematic Review. Cancers [Internet]. 2024 Jul 30;16(15):2715. Available from: http://dx.doi.org/10.3390/cancers16152715

Abstract

Introduction: PCa is one of the cancers that exhibits the widest disparity gaps. Geographical place of residence has been shown to be associated with healthcare access/utilization and PCa outcomes. Geographical Information Systems (GIS) are widely being utilized for PCa disparities research, however, inconsistencies in their application exist. This systematic review will summarize GIS application within PCa disparities research, highlight gaps in the literature, and propose alternative approaches. Methods: This paper followed the methods of the Cochrane Collaboration and the criteria set of the Preferred Reporting Items for Systematic Reviews and Meta-Analyses (PRISMA). Articles published in peer-reviewed journals were searched through the PubMed, Embase, and Web of Science databases until December 2022. The main inclusion criteria were employing a GIS approach and examining a relationship between geographical components and PCa disparities. The main exclusion criteria were studies conducted outside the US and those that were not published in English. Results: A total of 25 articles were included; 23 focused on PCa measures as outcomes: incidence, survival, and mortality, while only 2 examined PCa management. GIS application in PCa disparities research was grouped into three main categories: mapping, processing, and analysis. GIS mapping allowed for the visualization of quantitative, qualitative, and temporal trends of PCa factors. GIS processing was mainly used for geocoding and smoothing of PCa rates. GIS analysis mainly served to evaluate global spatial autocorrelation and distribution of PCa cases, while local cluster identification techniques were mainly employed to identify locations with poorer PCa outcomes, soliciting public health interventions. Discussion: Varied GIS applications and methodologies have been used in researching PCa disparities. Multiple geographical scales were adopted, leading to

variations in associations and outcomes. Geocoding quality varied considerably, leading to less robust findings. Limitations in cluster-detection approaches were identified, especially when variations were captured using the Spatial Scan Statistic. GIS approaches utilized in other diseases might be applied within PCa disparities research for more accurate inferences. A novel approach for GIS research in PCa disparities could be focusing more on geospatial disparities in procedure utilization especially when it comes to PCa screening techniques. Conclusions: This systematic review summarized and described the current state and trend of GIS application in PCa disparities research. Although GIS is of crucial importance when it comes to PCa disparities research, future studies should rely on more robust GIS techniques, carefully select the geographical scale studied, and partner with GIS scientists for more accurate inferences. Such interdisciplinary approaches have the potential to bridge the gaps between GIS and cancer prevention and control to further advance cancer equity.

Keywords:

GIS, disparities, geographic, prostate cancer, systematic review

<u>Original Lay Summary</u>

Prostate cancer (PCa) has significant disparities based on geography, affecting healthcare access and outcomes. This study reviews how Geographic Information Systems (GIS) are used to research these disparities. GIS helps visualize PCa incidence, survival, and mortality trends, but its application varies, leading to inconsistent results. The review followed Cochrane and PRISMA guidelines, analyzing 25 relevant studies. Most studies used GIS for mapping PCa data, geocoding, and spatial analysis to identify areas with poor PCa outcomes. However, inconsistencies in GIS methods and geographical scales used reduced the robustness of findings. The review suggests that better GIS techniques and interdisciplinary collaboration could improve the accuracy of PCa disparity research and support public health interventions.

**Assessment Category**	**Score**	**Assessment Category**	**Score**	**Assessment Category**	**Score**
Comprehension Score Rater 1	5	Gunning Fog Score	16.0	Words	112
Comprehension Score Rater 2	5	SMOG Index	11.8	Complex words	29
Reading Age (years)	21.5	Coleman Liau Index	10.4	Percentage of complex words	25.89
Flesch-Kincaid Reading Ease	20.6	Automated Readability Index	15.5	Average words per sentence	16.0
Flesch-Kincaid Grade Level	14.4	Sentences	7	Average syllables per word	2.01

Lay Summary (Simple Prompt)

Prostate cancer affects men differently depending on where they live, which can lead to unequal access to healthcare and differences in outcomes. Geographic Information Systems (GIS) are tools that help researchers study these patterns by mapping and analyzing data related to cancer rates, survival, and treatment. This systematic review examined how GIS has been used to study prostate cancer disparities in the United States. We reviewed 25 studies to understand how GIS helps identify areas with higher cancer risks and poorer outcomes, which can guide public health efforts. However, we found inconsistencies in how GIS is applied, with variations in data quality and analysis methods. To improve future research, we suggest using more advanced GIS techniques and collaborating with GIS experts. This can help create better strategies to address prostate cancer disparities and promote health equity for all men, regardless of where they live.

**Assessment Category**	**Score**	**Assessment Category**	**Score**	**Assessment Category**	**Score**
Comprehension Score Rater 1	5	Gunning Fog Score	15.2	Words	144
Comprehension Score Rater 2	5	SMOG Index	11.4	Complex words	27
Reading Age (years)	19.5	Coleman Liau Index	16.3	Percentage of complex words	18.75
Flesch-Kincaid Reading Ease	37.9	Automated Readability Index	14.6	Average words per sentence	20.57
Flesch-Kincaid Grade Level	13.1	Sentences	7	Average syllables per word	1.75

<u>Lay Summary (Extended Prompt)</u>

Prostate cancer affects men differently depending on where they live, which can impact their access to healthcare and their health outcomes. This study reviewed how Geographic Information Systems (GIS), a tool that helps analyze and map data based on location, have been used to study these differences. By looking at 25 studies, the review found that GIS helps show where prostate cancer rates are higher, where survival rates are lower, and where access to care is limited. However, the way GIS has been used varies a lot, which can lead to mixed results. The study suggests that future research should improve how GIS is applied, work closely with mapping experts, and focus on finding better ways to identify areas with the greatest need for cancer prevention and treatment. This can help reduce health gaps and improve outcomes for men with prostate cancer.

**Assessment Category**	**Score**	**Assessment Category**	**Score**	**Assessment Category**	**Score**
Comprehension Score Rater 1	5	Gunning Fog Score	12.3	Words	142
Comprehension Score Rater 2	5	SMOG Index	8.3	Complex words	12
Reading Age (years)	17.5	Coleman Liau Index	13.3	Percentage of complex words	8.45
Flesch-Kincaid Reading Ease	57.7	Automated Readability Index	13.7	Average words per sentence	23.67
Flesch-Kincaid Grade Level	11.1	Sentences	6	Average syllables per word	1.48

Manuscript 83

Gogineni E, Chen H, Cruickshank IK, Koempel A, Gogineni A, Li H, et al. In Silico Comparison of Three Different Beam Arrangements for Intensity-Modulated Proton Therapy for Postoperative Whole Pelvic Irradiation of Prostate Cancer. Cancers [Internet]. 2024 Jul 30;16(15):2702. Available from: http://dx.doi.org/10.3390/cancers16152702

Abstract

Background and purpose: Proton therapy has been shown to provide dosimetric benefits in comparison with IMRT when treating prostate cancer with whole pelvis radiation; however, the optimal proton beam arrangement has yet to be established. The aim of this study was to evaluate three different intensity-modulated proton therapy (IMPT) beam arrangements when treating the prostate bed and pelvis in the postoperative setting. Materials and Methods: Twenty-three post-prostatectomy patients were planned using three different beam arrangements: two-field (IMPT2B) (opposed laterals), three-field (IMPT3B) (opposed laterals inferiorly matched to a posterior-anterior beam superiorly), and four-field (IMPT4B) (opposed laterals inferiorly matched to two posterior oblique beams superiorly) arrangements. The prescription was 50 Gy radiobiological equivalent (GyE) to the pelvis and 70 GyE to the prostate bed. Comparisons were made using paired two-sided Wilcoxon signed-rank tests. Results: CTV coverages were met for all IMPT plans, with 99% of CTVs receiving ≥ 100% of prescription doses. All organ at risk (OAR) objectives were met with IMPT3B and IMPT4B plans, while several rectum objectives were exceeded by IMPT2B plans. IMPT4B provided the lowest doses to OARs for the majority of analyzed outcomes, with significantly lower doses than IMPT2B +/- IMPT3B for bladder V30-V50 and mean dose; bowel V15-V45 and mean dose; sigmoid maximum dose; rectum V40-V72.1, maximum dose, and mean dose; femoral head V37-40 and maximum dose; bone V40 and mean dose; penile bulb mean dose; and skin maximum dose. Conclusion: This study is the first to compare proton beam arrangements when treating the prostate bed and pelvis. four-field plans provided

better sparing of the bladder, bowel, and rectum than 2- and three-field plans. The data presented herein may help inform the future delivery of whole pelvis IMPT for prostate cancer.

Keywords:

IMPT, beam arrangement, dosimetric, dosimetry, intensity-modulated proton therapy, pelvic, pencil beam, prostate cancer, proton, radiation therapy, scanning beam, whole pelvis

Original Lay Summary

Proton therapy has been shown to provide dosimetric benefits in comparison with IMRT when treating prostate cancer with whole pelvis radiation; however, the optimal proton beam arrangement has yet to be established. Twenty-three post-prostatectomy patients were planned using three different beam arrangements: two-field (opposed laterals), three-field (opposed laterals inferiorly matched to a posterior–anterior beam superiorly), and four-field (opposed laterals inferiorly matched to two posterior oblique beams superiorly) arrangements. CTV coverages were similar for all plans, while the four-field plan provided the lowest doses to several metrics for bladder, bowel, sigmoid, rectum, femoral head, bone, penile bulb, and skin. The data presented herein may help inform the future delivery of whole pelvis IMPT for prostate cancer.

**Assessment Category**	**Score**	**Assessment Category**	**Score**	**Assessment Category**	**Score**
Comprehension Score Rater 1	5	Gunning Fog Score	21.7	Words	121
Comprehension Score Rater 2	5	SMOG Index	15.5	Complex words	29
Reading Age (years)	24.0	Coleman Liau Index	17.9	Percentage of complex words	23.97
Flesch-Kincaid Reading Ease	16.7	Automated Readability Index	20.7	Average words per sentence	30.25
Flesch-Kincaid Grade Level	18.4	Sentences	4	Average syllables per word	1.88

<u>Lay Summary (Simple Prompt)</u>

Prostate cancer patients who have undergone surgery often need radiation therapy to prevent the cancer from coming back. Proton therapy is a type of radiation that can target cancer more precisely, reducing damage to healthy organs. However, it's unclear which proton beam setup works best for treating the pelvis after prostate surgery. In this study, we compared three different proton beam arrangements to see which one offers the best balance between effectively treating the cancer area and protecting nearby healthy organs like the bladder, bowel, and rectum. We analyzed treatment plans from 23 patients and found that using four beams provided the best protection for healthy tissues without compromising cancer treatment. These findings could help improve future radiation therapy techniques, offering safer and more effective treatment options for prostate cancer patients.

**Assessment Category**	**Score**	**Assessment Category**	**Score**	**Assessment Category**	**Score**
Comprehension Score Rater 1	5	Gunning Fog Score	15.8	Words	131
Comprehension Score Rater 2	5	SMOG Index	11.3	Complex words	23
Reading Age (years)	20.5	Coleman Liau Index	17.4	Percentage of complex words	17.56
Flesch-Kincaid Reading Ease	33.6	Automated Readability Index	16.0	Average words per sentence	21.83
Flesch-Kincaid Grade Level	14.0	Sentences	6	Average syllables per word	1.79

<u>Lay Summary (Extended Prompt)</u>

Prostate cancer patients who have had surgery often need radiation therapy to reduce the risk of cancer returning. Proton therapy is a special type of radiation that can target cancer more precisely, helping to protect healthy tissues. However, the best way to arrange the proton beams for this treatment is still unclear. In this study, we compared three different ways of aiming proton beams to treat the pelvic area after prostate surgery. We looked at how well each method covered the target area and how much radiation reached healthy organs like the bladder, bowel, and rectum. Our results showed that using four proton beams reduced radiation to healthy organs better than using two or three beams. This means the four-beam method could lower the risk of side effects while still effectively treating the cancer. These findings may help doctors improve radiation treatment plans for prostate cancer patients in the future.

**Assessment Category**	**Score**	**Assessment Category**	**Score**	**Assessment Category**	**Score**
Comprehension Score Rater 1	5	Gunning Fog Score	11.3	Words	151
Comprehension Score Rater 2	5	SMOG Index	7.8	Complex words	14
Reading Age (years)	16.5	Coleman Liau Index	14.1	Percentage of complex words	9.27
Flesch-Kincaid Reading Ease	54.3	Automated Readability Index	11.9	Average words per sentence	18.88
Flesch-Kincaid Grade Level	10.4	Sentences	8	Average syllables per word	1.58

Manuscript 84

Papalia GF, Brigato P, Sisca L, Maltese G, Faiella E, Santucci D, et al. Artificial Intelligence in Detection, Management, and Prognosis of Bone Metastasis: A Systematic Review. Cancers [Internet]. 2024 Jul 29;16(15):2700. Available from: http://dx.doi.org/10.3390/cancers16152700

Abstract

Background: Metastasis commonly occur in the bone tissue. Artificial intelligence (AI) has become increasingly prevalent in the medical sector as support in decision-making, diagnosis, and treatment processes. The objective of this systematic review was to assess the reliability of AI systems in clinical, radiological, and pathological aspects of bone metastases.

Methods: We included studies that evaluated the use of AI applications in patients affected by bone metastases. Two reviewers performed a digital search on 31 December 2023 on PubMed, Scopus, and Cochrane library and extracted authors, AI method, interest area, main modalities used, and main objectives from the included studies.

Results: We included 59 studies that analyzed the contribution of computational intelligence in diagnosing or forecasting outcomes in patients with bone metastasis. Six studies were specific for spine metastasis. The study involved nuclear medicine (44.1%), clinical research (28.8%), radiology (20.4%), or molecular biology (6.8%). When a primary tumor was reported, prostate cancer was the most common, followed by lung, breast, and kidney.

Conclusions: Appropriately trained AI models may be very useful in merging information to achieve an overall improved diagnostic accuracy and treatment for metastasis in the bone. Nevertheless, there are still concerns with the use of AI systems in medical settings. Ethical considerations and legal issues must be

addressed to facilitate the safe and regulated adoption of AI technologies. The limitations of the study comprise a stronger emphasis on early detection rather than tumor management and prognosis as well as a high heterogeneity for type of tumor, AI technology and radiological techniques, pathology, or laboratory samples involved.

Keywords:

artificial intelligence, bone metastasis, deep learning, machine learning, orthopedic oncology

<u>Original Lay Summary</u>

Bone metastases represent a serious and common challenge in advanced cancer patients, making early detection crucial. The growing implementation of artificial intelligence (AI) in various medical fields is revolutionizing healthcare by enhancing diagnostic accuracy. This systematic review includes 59 articles and sought to assess the potential of machine learning models in the fields of nuclear medicine, clinical research, radiology, and molecular biology. The review provides a comprehensive analysis of AI's effectiveness in detecting bone metastases, which had not yet been thoroughly conducted. The findings highlight the benefits of integrating AI technologies in the inspected fields, encouraging further advancements and adoption of AI to ultimately improve patient care and treatment planning. This study provides invaluable perceptions into the promising application of computational intelligence in the detection of bone metastases, demonstrating its superiority over standard tests. However, further research is necessary to substantiate these findings with the aim of developing better-trained data-driven models soon.

**Assessment Category**	**Score**	**Assessment Category**	**Score**	**Assessment Category**	**Score**
Comprehension Score Rater 1	5	Gunning Fog Score	21.8	Words	154
Comprehension Score Rater 2	5	SMOG Index	15.4	Complex words	50
Reading Age (years)	24.0	Coleman Liau Index	20.9	Percentage of complex words	32.47
Flesch-Kincaid Reading Ease	4.9	Automated Readability Index	19.0	Average words per sentence	22.0

Flesch-Kincaid Grade Level	18.0	Sentences	7	Average syllables per word	2.12

Lay Summary (Simple Prompt)

Bone metastases, where cancer spreads to the bones, can cause serious health problems. Detecting and managing these conditions early is crucial for better outcomes. In recent years, artificial intelligence (AI) has shown great promise in helping doctors diagnose and treat various diseases. This systematic review looked at 59 studies to understand how reliable AI is in identifying, managing, and predicting the progression of bone metastases. The research covered different medical fields, including nuclear medicine, radiology, and clinical practice, with a focus on cancers like prostate, lung, breast, and kidney. The findings suggest that well-designed AI tools can improve the accuracy of diagnosing bone metastases and support better treatment decisions. However, challenges remain, such as ethical concerns and legal issues around using AI in healthcare. Addressing these challenges is important to ensure AI can be safely and effectively integrated into medical practice.

**Assessment Category**	**Score**	**Assessment Category**	**Score**	**Assessment Category**	**Score**
Comprehension Score Rater 1	5	Gunning Fog Score	16.7	Words	142
Comprehension Score Rater 2	5	SMOG Index	11.9	Complex words	34
Reading Age (years)	20.5	Coleman Liau Index	17.9	Percentage of complex words	23.94
Flesch-Kincaid Reading Ease	33.3	Automated Readability Index	14.4	Average words per sentence	17.75
Flesch-Kincaid Grade Level	13.0	Sentences	8	Average syllables per word	1.84

<u>Lay Summary (Extended Prompt)</u>

Bone metastasis happens when cancer spreads to the bones, which can cause serious health problems. This study looked at how artificial intelligence (AI)—a technology that helps computers learn and make decisions—can support doctors in detecting, managing, and predicting the outcomes of bone metastases. Researchers reviewed 59 studies to see how well AI works in different medical areas like imaging, lab tests, and patient care. They found that AI can improve how quickly and accurately bone metastases are diagnosed and may help guide better treatment decisions. However, using AI in healthcare also raises important questions about ethics and legal responsibilities. While AI shows promise, more work is needed to make sure it is safe, reliable, and used in the best way for patients. This research helps highlight both the benefits and challenges of using AI in managing bone metastasis, paving the way for future improvements in patient care.

**Assessment Category**	**Score**	**Assessment Category**	**Score**	**Assessment Category**	**Score**
Comprehension Score Rater 1	5	Gunning Fog Score	15.8	Words	148
Comprehension Score Rater 2	5	SMOG Index	11.4	Complex words	27
Reading Age (years)	19.5	Coleman Liau Index	15.2	Percentage of complex words	18.24
Flesch-Kincaid Reading Ease	44.8	Automated Readability Index	13.9	Average words per sentence	21.14
Flesch-Kincaid Grade Level	12.3	Sentences	7	Average syllables per word	1.66

Manuscript 85

Cruz-Montijano M, Amo-Salas M, Cassinello-Espinosa J, García-Carbonero I, Villa-Guzman JC, Garcia-Vicente AM. Predictive and Prognostic 18F-Fluorocholine PET/CT Radiomics Nomogram in Patients with Castration-Resistant Prostate Cancer with Bone Metastases Treated with 223Ra. Cancers [Internet]. 2024 Jul 29;16(15):2695. Available from: http://dx.doi.org/10.3390/cancers16152695

Abstract

Purpose: We aimed to develop a nomogram able to predict treatment failure, skeletal events, and overall survival (OS) in patients with castration-resistant prostate cancer with bone metastases (CRPC-BM) treated with Radium-223 dichloride (223Ra).

Patients and methods: Patients from the Castilla-La Mancha Spanish region were prospectively included in the ChoPET-Rad multicenter study from January 2015 to December 2022. Patients underwent baseline, interim, and end-of-treatment bone scintigraphy (BS) and 18F-Fluorocholine PET/CT (FCH PET/CT) scans, obtaining multiple imaging radiomics as well as clinical and biochemical variables during follow-up and studying their association with the previously defined end-points. Survival analysis was performed using the Kaplan-Meier method and Cox regression. Multivariate logistic and Cox regression models were calculated, and these models were depicted by means of nomograms.

Results: Median progression-free survival (PFS) and OS were 4 and 14 months (mo), respectively. The variables that showed independent and significant association with therapeutic failure were baseline alkaline phosphatase (AP) levels ($p = 0.022$) and the characteristics of BM on the CT portion of PET/CT ($p = 0.017$). In the case of OS, the significant variables were therapeutic failure ($p = 0.038$), the number of lines received after 223Ra ($p < 0.001$), average SUVmax ($p = 0.002$), bone marrow infiltration in FCH PET/CT ($p = 0.006$), and interim FCH PET/CT response ($p = 0.048$). Final nomograms included these variables, showing good discrimination among the 100 patients

included in our study. In the study of skeletal events, only OS showed a significant association in the multivariate analysis, resulting in an inconsistent nomogram design.

Conclusions: FCH PET/CT appears to be a good tool for evaluating patients eligible for treatment with 223Ra, as well as for their follow-up. Thus, findings derived from it, such as the morphological characteristics of BM in the CT, bone marrow infiltration, or the response to 223Ra in the interim study, have proven to be solid and useful variables in the creation of nomograms for predicting therapeutic failure and OS.

Keywords:

18F-fluorocholine PET/CT, bone scintigraphy, castration-resistant prostate cancer with bone metastases (CRPC-BM), nomogram, overall survival, radiomics, radium-223 dichloride, therapeutic failure

Original Lay Summary

Response to treatment and prognosis after 223Ra treatment varies among patients. Using a combination of clinical, biochemical, and radiomic data from bone scintigraphy and 18F-Fluorocholine PET/CT obtained from our prospective ChoPET-Rad study, we identified predictive and prognostic variables. The goal was to create a nomogram able to predict therapeutic failure and overall survival, aiding in the selection of more suitable candidates for this treatment.

**Assessment Category**	**Score**	**Assessment Category**	**Score**	**Assessment Category**	**Score**
Comprehension Score Rater 1	3	Gunning Fog Score	19.7	Words	66
Comprehension Score Rater 2	3	SMOG Index	14.9	Complex words	20
Reading Age (years)	22.5	Coleman Liau Index	18.2	Percentage of complex words	30.3
Flesch-Kincaid Reading Ease	16.6	Automated Readability Index	16.8	Average words per sentence	22.0
Flesch-Kincaid Grade Level	16.4	Sentences	3	Average syllables per word	1.98

Lay Summary (Simple Prompt)

Patients with advanced prostate cancer that has spread to the bones often face challenges in predicting how well treatments will work and how long they might live. This study aimed to develop a tool called a "nomogram" to help doctors make better predictions for these patients who are treated with a specific medication called Radium-223. By analyzing detailed medical images from PET/CT scans along with clinical information, researchers identified key factors—such as certain blood test results, the appearance of bone metastases on scans, and the patient's response to treatment—that can predict treatment success and survival. The results showed that these factors could be combined effectively to create prediction models, helping doctors personalize care and improve decision-making. This research highlights the value of advanced imaging techniques in guiding prostate cancer treatment and monitoring, potentially leading to better outcomes for patients.

**Assessment Category**	**Score**	**Assessment Category**	**Score**	**Assessment Category**	**Score**
Comprehension Score Rater 1	4	Gunning Fog Score	18.4	Words	141
Comprehension Score Rater 2	4	SMOG Index	13.4	Complex words	27
Reading Age (years)	22.5	Coleman Liau Index	17.7	Percentage of complex words	19.15
Flesch-Kincaid Reading Ease	33.6	Automated Readability Index	19.5	Average words per sentence	28.2
Flesch-Kincaid Grade Level	15.6	Sentences	5	Average syllables per word	1.71

Lay Summary (Extended Prompt)

Prostate cancer that spreads to the bones can be hard to treat, especially when it no longer responds to hormone therapy. This study aimed to find better ways to predict how these patients will respond to a treatment called Radium-223, which helps target bone tumors. We looked at detailed images from special scans (called 18F-Fluorocholine PET/CT) and combined this information with blood tests and other clinical data from 100 patients. Using this data, we created a tool called a "nomogram" to help doctors estimate the risk of treatment failure and predict how long a patient might live. We found that certain scan features, blood markers, and treatment responses were important in predicting outcomes. This tool can help doctors make more personalized treatment plans, improving care for patients with advanced prostate cancer.

**Assessment Category**	**Score**	**Assessment Category**	**Score**	**Assessment Category**	**Score**
Comprehension Score Rater 1	4	Gunning Fog Score	11.9	Words	133
Comprehension Score Rater 2	4	SMOG Index	8.6	Complex words	13
Reading Age (years)	17.5	Coleman Liau Index	14.1	Percentage of complex words	9.77
Flesch-Kincaid Reading Ease	57.8	Automated Readability Index	13.6	Average words per sentence	22.17
Flesch-Kincaid Grade Level	10.7	Sentences	6	Average syllables per word	1.5

Manuscript 86

Hein C, Burgard C, Blickle A, Bastian MB, Maus S, Schaefer-Schuler A, et al. Analysis of Molecular Imaging and Laboratory Baseline Biomarkers in PSMA-RLT: Whole-Body Total Lesion PSMA (TLP) Predicts Overall Survival. Cancers [Internet]. 2024 Jul 26;16(15):2670. Available from: http://dx.doi.org/10.3390/cancers16152670

Abstract

The aim of this retrospective study was to identify pre-therapeutic predictive laboratory and molecular imaging biomarkers for response and overall survival (OS) in patients with metastatic castration-resistant prostate cancer (mCRPC) treated with prostate-specific membrane antigen (PSMA)-targeted radioligand therapy (RLT). Pre-therapeutic laboratory and [68Ga]Ga-PSMA-11 PET/CT data of n = 102 mCRPC patients receiving [177Lu]Lu-PSMA-617 RLT within a prospective registry (REALITY Study, NCT04833517) were analyzed including laboratory parameters such as alkaline phosphatase (ALP), prostate-specific antigen (PSA), gamma glutamyl transferase (GGT), glutamate oxaloacetate transaminase (GOT), glutamate pyruvate transaminase (GPT), neuron specific enolase (NSE), hemoglobin (Hb), and imaging parameters such as maximum standardized uptake value of the tumor lesions (SUVmax), the mean standardized uptake value of all tumor lesions (SUVmean), the whole-body molecular tumor volume (MTV), and the whole-body total lesion PSMA (TLP). Mann-Whitney U test, univariate and multivariable Cox-regression were performed to test for association of the parameters with response and OS. The SUVmean of all lesions was significantly different between responders and non-responders (SUVmean responders 8.95 ± 2.83 vs. non-responders 7.88 ± 4.46, p = 0.003), whereas all other tested biochemical and imaging parameters did not reveal significant differences. Hb and the molecular imaging parameters MTV and TLP showed a significant association with OS (p = 0.013, p = 0.005; p = 0.009) in univariant Cox regression; however, only TLP remained significant in multivariable analysis (Hazard ratio 1.033, p = 0.009). This study demonstrates a statistically significant association between the quantitative PET/CT imaging parameter SUVmean and PSA

response, as well as between the baseline TLP and OS of mCRPC patients undergoing RLT.

Keywords:

PET/CT, biomarker, metastatic castration-resistant prostate cancer (mCRPC), prostate-specific membrane antigen (PSMA), radioligand therapy (RLT), total lesion PSMA (TLP)

<u>Original Lay Summary</u>

Prostate specific membrane antigen (PSMA)-targeted radioligand therapy (RLT) is a promising and recently approved treatment option for patients with metastatic castration-resistant prostate cancer (mCRPC). The aim of this study was to analyze which laboratory and PET imaging parameters are able to predict biochemical response and overall survival with this treatment. Two quantitative imaging biomarkers were identified that allow prediction of RLT outcome, further improving the pre-therapeutic characterization of mCRPC patients undergoing PSMA-RLT.

**Assessment Category**	**Score**	**Assessment Category**	**Score**	**Assessment Category**	**Score**
Comprehension Score Rater 1	4	Gunning Fog Score	24.3	Words	76
Comprehension Score Rater 2	5	SMOG Index	17.2	Complex words	27
Reading Age (years)	24.0	Coleman Liau Index	20.5	Percentage of complex words	25.53
Flesch-Kincaid Reading Ease	0.8	Automated Readability Index	20.2	Average words per sentence	25.33
Flesch-Kincaid Grade Level	19.4	Sentences	3	Average syllables per word	2.13

Lay Summary (Simple Prompt)

This study focused on finding reliable indicators that can help predict how well patients with advanced prostate cancer will respond to a specific treatment called PSMA-targeted radioligand therapy. Researchers analyzed blood tests and special imaging scans from 102 patients to identify factors linked to treatment success and overall survival. They discovered that a measurement from PET/CT scans, known as total lesion PSMA (TLP), was strongly associated with how long patients lived after treatment. Additionally, another imaging marker, SUVmean, helped predict whether the cancer responded to therapy. These findings are important because they may help doctors better identify which patients are most likely to benefit from this therapy, allowing for more personalized and effective treatment plans.

**Assessment Category**	**Score**	**Assessment Category**	**Score**	**Assessment Category**	**Score**
Comprehension Score Rater 1	5	Gunning Fog Score	18.2	Words	116
Comprehension Score Rater 2	5	SMOG Index	13.2	Complex words	26
Reading Age (years)	21.5	Coleman Liau Index	18.3	Percentage of complex words	22.41
Flesch-Kincaid Reading Ease	27.9	Automated Readability Index	17.5	Average words per sentence	23.2
Flesch-Kincaid Grade Level	15.1	Sentences	5	Average syllables per word	1.84

Lay Summary (Extended Prompt)

Prostate cancer that has spread and no longer responds to hormone therapy is hard to treat. This study looked at whether certain blood tests and imaging results could help predict how well patients respond to a special treatment called PSMA-targeted radioligand therapy. We studied 102 patients and analyzed their lab results and PET/CT scans taken before treatment. We found that a specific measurement from the scans, called total lesion PSMA (TLP), was linked to how long patients lived after treatment. Additionally, another scan measurement (SUVmean) was connected to how well patients responded to therapy. These findings suggest that TLP could help doctors better predict outcomes for prostate cancer patients, allowing for more personalized treatment plans and improved care.

**Assessment Category**	**Score**	**Assessment Category**	**Score**	**Assessment Category**	**Score**
Comprehension Score Rater 1	5	Gunning Fog Score	13.0	Words	119
Comprehension Score Rater 2	5	SMOG Index	9.2	Complex words	15
Reading Age (years)	18.5	Coleman Liau Index	16.2	Percentage of complex words	12.61
Flesch-Kincaid Reading Ease	48.1	Automated Readability Index	14.1	Average words per sentence	19.83
Flesch-Kincaid Grade Level	11.5	Sentences	6	Average syllables per word	1.64

Manuscript 87

van Oostwaard MM, van den Bergh JP, van de Wouw AJ, de Jong M, Janssen-Heijnen ML, Wyers CE. Development of a Multidisciplinary Care Pathway for Fracture Prevention in Men with Prostate Cancer at Initiation of Androgen Deprivation Therapy. Cancers [Internet]. 2024 Jul 26;16(15):2665. Available from: http://dx.doi.org/10.3390/cancers16152665

Abstract

Fracture risk is increased in men with prostate cancer (PCa) receiving Androgen Deprivation Therapy (ADT). However, routine assessment of fracture risk is often not systematically applied. We aimed to establish a comprehensive care pathway for fracture prevention in men with PCa starting ADT. Therefore, a multidisciplinary working group designed and implemented a care pathway using the 'Knowledge to Action' framework, based on current Dutch guidelines for PCa, osteoporosis and fracture prevention, and an extensive literature review of other guidelines. The pathway was developed according to a five-step clinical approach including case finding, fracture risk assessment based on risk factors, bone mineral density test, vertebral fracture assessment, differential diagnosis, treatment, and annual follow-up. Our fracture prevention care pathway for patients with PCa at the time of ADT initiation was designed to promote a patient-centered, multidisciplinary approach to facilitate the implementation of early fracture prevention measures.

Keywords:

androgen deprivation therapy, care pathway, fracture prevention, fracture risk, osteoporosis, prostate cancer

<u>Original Lay Summary</u>

Men with prostate cancer undergoing Androgen Deprivation Therapy (ADT) have an increased risk of fractures. Despite this risk, fracture risk assessment is often not systematically applied. To address this issue, a care pathway for preventing fractures in these patients was developed. A multidisciplinary working group designed this pathway based on Dutch guidelines for prostate cancer, osteoporosis and fracture prevention and a thorough literature review. The care pathway includes five steps: case finding, fracture risk assessment, bone mineral density testing, vertebral fracture assessment, differential diagnosis, treatment, and annual follow-up. This pathway focuses on a patient-centered approach and early preventive measures. The ongoing implementation and evaluation aim to enhance fracture preventive care for men with prostate cancer starting ADT.

**Assessment Category**	**Score**	**Assessment Category**	**Score**	**Assessment Category**	**Score**
Comprehension Score Rater 1	5	Gunning Fog Score	14.5	Words	119
Comprehension Score Rater 2	5	SMOG Index	11.2	Complex words	26
Reading Age (years)	20.5	Coleman Liau Index	20.1	Percentage of complex words	21.85
Flesch-Kincaid Reading Ease	26.1	Automated Readability Index	15.8	Average words per sentence	17.0
Flesch-Kincaid Grade Level	13.8	Sentences	7	Average syllables per word	1.93

Lay Summary (Simple Prompt)

Men with prostate cancer who start hormone therapy, known as androgen deprivation therapy (ADT), face a higher risk of bone fractures. Unfortunately, steps to assess and prevent these fractures are not always part of routine care. To address this, our study aimed to develop a clear, structured care plan to help prevent fractures in these patients. A team of healthcare experts from different specialties created a step-by-step approach, following the latest medical guidelines and research. This plan includes identifying patients at risk, assessing their bone health with specialized tests, diagnosing any existing bone issues, providing appropriate treatments, and ensuring regular follow-up care. By introducing this patient-centered, team-based pathway, we hope to improve early detection and prevention of bone fractures, ultimately enhancing the quality of life for men with prostate cancer undergoing ADT.

**Assessment Category**	**Score**	**Assessment Category**	**Score**	**Assessment Category**	**Score**
Comprehension Score Rater 1	5	Gunning Fog Score	17.0	Words	137
Comprehension Score Rater 2	5	SMOG Index	12.3	Complex words	27
Reading Age (years)	20.5	Coleman Liau Index	16.7	Percentage of complex words	19.71
Flesch-Kincaid Reading Ease	37.3	Automated Readability Index	16.0	Average words per sentence	22.83
Flesch-Kincaid Grade Level	13.7	Sentences	6	Average syllables per word	1.73

Lay Summary (Extended Prompt)

Men with prostate cancer who start hormone therapy, known as androgen deprivation therapy (ADT), have a higher risk of bone fractures. Despite this risk, doctors do not always check for bone problems early enough. Our study aimed to create a step-by-step care plan to help prevent fractures in these men. A team of experts from different medical fields worked together to design this plan, using the latest guidelines and research. The plan includes finding men at risk, checking their bone strength with tests, diagnosing bone issues, providing the right treatments, and scheduling regular check-ups. By following this care pathway, doctors can spot bone problems sooner and help prevent fractures. This approach puts the patient at the center of care and makes it easier for healthcare teams to work together to protect bone health from the very start of ADT.

**Assessment Category**	**Score**	**Assessment Category**	**Score**	**Assessment Category**	**Score**
Comprehension Score Rater 1	5	Gunning Fog Score	12.3	Words	142
Comprehension Score Rater 2	5	SMOG Index	8.6	Complex words	15
Reading Age (years)	16.5	Coleman Liau Index	13.3	Percentage of complex words	10.56
Flesch-Kincaid Reading Ease	61.1	Automated Readability Index	12.0	Average words per sentence	20.29
Flesch-Kincaid Grade Level	9.8	Sentences	7	Average syllables per word	1.48

Manuscript 88

Fujiwara R, Yamamoto S, Takemura K, Yuasa T, Numao N, Oguchi T, et al. Clinical Outcomes and Prognostic Factors in Nonmetastatic Castration-Resistant Prostate Cancer Treated with Androgen Receptor Signaling Inhibitors Therapy. Cancers [Internet]. 2024 Jul 26;16(15):2659. Available from: http://dx.doi.org/10.3390/cancers16152659

Abstract

We conducted a retrospective evaluation of the clinical outcomes and prognostic factors in patients with nonmetastatic castration-resistant prostate cancer (nmCRPC) treated with first-line androgen receptor signaling inhibitors (ARSI) in real-world clinical practice in Japan. Between 2012 and 2023, a total of 127 consecutive patients with nmCRPC received ARSI treatment. Overall survival (OS), metastatic-free survival (MFS), and prostate-specific antigen-progression-free survival (PSA-PFS) from ARSI initiation were assessed using the Kaplan-Meier methodology. Clinical factors associated with OS in nmCRPC were analyzed using the Cox proportional hazards model. Among the patients, 72, 26, 12, and 17 received enzalutamide (ENZ), abiraterone (ABI), apalutamide (APA), and darolutamide (DARO) as first-line therapy. The median OS and MFS for all patients were 79.0 and 42.0 months, respectively. Median PSA-PFS was 27.0, 20.0, 10.0, and 14.0 months for patients treated with ENZ, ABI, APA, and DARO, respectively ($p = 0.33$). Multivariate analysis revealed that a baseline PSA level ≥ 3.67 ng/mL at ARSI initiation was significantly associated with poorer OS ($p = 0.002$). ARSI demonstrated favorable efficacy in nmCRPC patients. There were no significant differences in clinical outcomes among different types of ARSI therapy for nmCRP. Elevated baseline PSA at ARSI initiation was significantly associated with poorer OS.

Keywords:

androgen receptor signaling inhibitors, nonmetastatic castration-resistant prostate cancer, overall survival, prostate-specific antigen, real-world clinical practice

<u>Original Lay Summary</u>

We retrospectively evaluated the clinical outcomes and prognostic factors of 127 patients with nonmetastatic castration-resistant prostate cancer (nmCRPC) treated with first-line androgen receptor signaling inhibitors (ARSI) in real-world clinical practice. Overall survival (OS), metastatic-free survival (MFS), and prostate-specific antigen–progression-free survival (PSA–PFS) from the initiation of ARSI were assessed. The median OS and MFS for all patients were 79.0 and 42.0 months, respectively. Median PSA–PFS varied among patients treated with enzalutamide, abiraterone acetate, apalutamide, and darolutamide (27.0, 20.0, 10.0, and 14.0 months, respectively; p = 0.33). Multivariate analysis indicated that a baseline PSA level of ≥3.67 ng/mL (p = 0.002) was significantly associated with poorer OS prognosis. ARSI demonstrated favorable efficacy in nmCRPC patients, with no significant differences in clinical outcomes among different types of ARSI. High baseline PSA of ARSI was found to have a significantly poor prognosis associated with OS.

**Assessment Category**	**Score**	**Assessment Category**	**Score**	**Assessment Category**	**Score**
Comprehension Score Rater 1	5	Gunning Fog Score	15.7	Words	156
Comprehension Score Rater 2	5	SMOG Index	10.0	Complex words	47
Reading Age (years)	17.5	Coleman Liau Index	15.4	Percentage of complex words	30.13
Flesch-Kincaid Reading Ease	28.3	Automated Readability Index	8.4	Average words per sentence	9.75
Flesch-Kincaid Grade Level	11.7	Sentences	16	Average syllables per word	1.99

<u>Lay Summary (Simple Prompt)</u>

This study explored how men with nonmetastatic castration-resistant prostate cancer, a type of prostate cancer that no longer responds to hormone therapy but has not yet spread, respond to treatments known as androgen receptor signaling inhibitors. Researchers reviewed the medical records of 127 patients in Japan treated between 2012 and 2023 with different drugs from this group. They looked at how long patients lived, how long they remained free from cancer spread, and how their prostate-specific antigen (PSA) levels changed, which helps track cancer activity. The study found that while these treatments were generally effective, men who started therapy with higher PSA levels tended to have shorter survival times. Interestingly, the specific drug used did not significantly affect outcomes. These findings can help doctors better understand which patients may need closer monitoring and guide treatment decisions to improve patient care.

**Assessment Category**	**Score**	**Assessment Category**	**Score**	**Assessment Category**	**Score**
Comprehension Score Rater 1	4	Gunning Fog Score	16.2	Words	142
Comprehension Score Rater 2	5	SMOG Index	11.6	Complex words	24
Reading Age (years)	20.5	Coleman Liau Index	16.5	Percentage of complex words	16.9
Flesch-Kincaid Reading Ease	40.4	Automated Readability Index	16.3	Average words per sentence	23.67
Flesch-Kincaid Grade Level	13.5	Sentences	6	Average syllables per word	1.68

<u>Lay Summary (Extended Prompt)</u>

Prostate cancer can sometimes become resistant to hormone therapy, even before it spreads to other parts of the body. This stage is called nonmetastatic castration-resistant prostate cancer. In this study, we looked at 127 men in Japan who were treated with different medicines that block the signals helping prostate cancer grow, known as androgen receptor signaling inhibitors. We wanted to see how well these treatments worked and what factors affected patient survival. We found that these medicines were generally effective, and most men lived several years after starting treatment. However, men with higher levels of prostate-specific antigen (PSA), a protein linked to prostate cancer, at the start of treatment had a shorter lifespan. Our findings suggest that PSA levels could help doctors predict patient outcomes and guide treatment decisions to improve care for men with this type of prostate cancer.

**Assessment Category**	**Score**	**Assessment Category**	**Score**	**Assessment Category**	**Score**
Comprehension Score Rater 1	4	Gunning Fog Score	14.6	Words	142
Comprehension Score Rater 2	5	SMOG Index	10.5	Complex words	23
Reading Age (years)	18.5	Coleman Liau Index	15.4	Percentage of complex words	16.2
Flesch-Kincaid Reading Ease	47.4	Automated Readability Index	13.7	Average words per sentence	20.29
Flesch-Kincaid Grade Level	11.7	Sentences	7	Average syllables per word	1.64

Manuscript 89

Liadi YM, Campbell T, Hwang BJ, Elliott B, Odero-Marah V. High Mobility Group AT-hook 2: A Biomarker Associated with Resistance to Enzalutamide in Prostate Cancer Cells. Cancers [Internet]. 2024 Jul 24;16(15):2631. Available from: http://dx.doi.org/10.3390/cancers16152631

Abstract

Metastatic prostate cancer (mPCa) is a leading cause of mortality, partly due to its resistance to anti-androgens like enzalutamide. Snail can promote this resistance by increasing full-length AR and AR-V7. High Mobility Group AT-hook 2 (HMGA2), a DNA-binding protein upstream of Snail, is crucial in proliferation and epithelial-mesenchymal transition (EMT). This study examines HMGA2's role in enzalutamide resistance. LNCaP and 22Rv1 cells overexpressing wild-type HMGA2, but not truncated HMGA2, showed EMT. Both variants led to a decreased sensitivity to enzalutamide but not alisertib compared to Neo control cells. The overexpression of HMGA2 did not alter AR expression. Enzalutamide-resistant C4-2B cells (C4-2B MDVR) had higher HMGA2 and AR/AR variant expression than enzalutamide-sensitive C4-2B cells but remained sensitive to alisertib. The HMGA2 knockdown in C4-2B MDVR cells increased sensitivity to both enzalutamide and alisertib without changing AR expression. A clinical analysis via cBioPortal revealed HMGA2 alterations in 3% and AR alterations in 59% of patients. The HMGA2 changes were linked to treatments like enzalutamide, abiraterone, or alisertib, with amplifications more prevalent in bone, lymph node, and liver metastases. Conclusively, HMGA2 is a potential biomarker for enzalutamide resistance in mPCa, independent of Snail and AR signaling, and alisertib may be an effective treatment for mPCa that expresses HMGA2.

Keywords:

Snail, alisertib, androgen receptor (AR), enzalutamide, enzalutamide-resistant, epithelial to mesenchymal transition (EMT), high mobility group AT-hook 2 (HMGA2), metastatic prostate cancer

Original Lay Summary

Metastatic prostate cancer (mPCa) is a leading cause of death primarily due to its resistance to treatments like enzalutamide. This study investigates how the protein HMGA2 contributes to this resistance. We found that cancer cells with high levels of HMGA2 became less sensitive to enzalutamide but not to another drug, alisertib. Interestingly, although these cells resisted enzalutamide, they did not show changes in a key prostate cancer protein called AR. Furthermore, a knockdown of HMGA2 sensitized the cells to both drugs. Clinically, 3% of patients showed changes in HMGA2, which were more common in cancers that had metastasized to bones, lymph nodes, and the liver. Our research suggests that HMGA2 could be used as a marker to predict enzalutamide resistance in mPCa cells. Additionally, alisertib may be an effective treatment for patients with high HMGA2 levels, offering a new potential therapeutic strategy for managing metastatic prostate cancer.

**Assessment Category**	**Score**	**Assessment Category**	**Score**	**Assessment Category**	**Score**
Comprehension Score Rater 1	5	Gunning Fog Score	15.0	Words	147
Comprehension Score Rater 2	5	SMOG Index	11.0	Complex words	29
Reading Age (years)	18.5	Coleman Liau Index	15.8	Percentage of complex words	19.73
Flesch-Kincaid Reading Ease	43.7	Automated Readability Index	13.0	Average words per sentence	18.38
Flesch-Kincaid Grade Level	11.7	Sentences	8	Average syllables per word	1.71

<u>Lay Summary (Simple Prompt)</u>

Prostate cancer that spreads to other parts of the body (metastatic prostate cancer) can become resistant to common treatments like enzalutamide, making it harder to control. This study explored the role of a protein called High Mobility Group AT-hook 2 (HMGA2) in this resistance. Researchers found that prostate cancer cells with high levels of HMGA2 were less responsive to enzalutamide but remained sensitive to another drug called alisertib. Importantly, reducing HMGA2 levels in resistant cells made them more responsive to both treatments. The study also discovered that changes in HMGA2 were more common in cancers that had spread to bones, lymph nodes, and the liver. These findings suggest that HMGA2 could be used as a biomarker to identify prostate cancers likely to resist enzalutamide and that alisertib may offer an effective treatment option for these cases. This research could help improve treatment strategies for advanced prostate cancer.

**Assessment Category**	**Score**	**Assessment Category**	**Score**	**Assessment Category**	**Score**
Comprehension Score Rater 1	5	Gunning Fog Score	14.1	Words	148
Comprehension Score Rater 2	5	SMOG Index	10.3	Complex words	22
Reading Age (years)	18.5	Coleman Liau Index	15.6	Percentage of complex words	14.86
Flesch-Kincaid Reading Ease	46.5	Automated Readability Index	14.3	Average words per sentence	21.14
Flesch-Kincaid Grade Level	12.0	Sentences	7	Average syllables per word	1.64

Lay Summary (Extended Prompt)

Prostate cancer can become harder to treat when it spreads to other parts of the body and stops responding to common medicines like enzalutamide. This study looked at a protein called High Mobility Group AT-hook 2 (HMGA2) to see if it plays a role in this drug resistance. Researchers found that when prostate cancer cells had more HMGA2, they were less sensitive to enzalutamide but still responded well to another drug called alisertib. Importantly, lowering HMGA2 levels made these resistant cancer cells respond better to both drugs. This means that HMGA2 could be a helpful marker to identify which prostate cancers might not respond to enzalutamide. It also suggests that alisertib could be an effective treatment option for these cases. Understanding HMGA2's role could help doctors choose better treatments for patients with advanced prostate cancer.

**Assessment Category**	**Score**	**Assessment Category**	**Score**	**Assessment Category**	**Score**
Comprehension Score Rater 1	5	Gunning Fog Score	12.5	Words	136
Comprehension Score Rater 2	5	SMOG Index	9.1	Complex words	17
Reading Age (years)	17.5	Coleman Liau Index	14.7	Percentage of complex words	12.5
Flesch-Kincaid Reading Ease	54.6	Automated Readability Index	12.7	Average words per sentence	19.43
Flesch-Kincaid Grade Level	10.5	Sentences	7	Average syllables per word	1.57

Manuscript 90

Iacovelli V, Carilli M, Bertolo R, Forte V, Vittori M, Filippi B, et al. Transperineal Laser Ablation for Focal Therapy of Localized Prostate Cancer: 12-Month Follow-up Outcomes from a Single Prospective Cohort Study. Cancers [Internet]. 2024 Jul 23;16(15):2620. Available from: http://dx.doi.org/10.3390/cancers16152620

Abstract

Introduction and objectives: To evaluate the oncological and functional outcomes of transperineal laser ablation (TPLA) as the focal therapy for localized prostate cancer (PCa) after a 12-month follow-up.

Materials and methods: Patients with low- and intermediate-risk localized PCa were prospectively treated with focal TPLA between July 2021 and December 2022. The inclusion criteria were the following: clinical stage < T2b; PSA < 20 ng/mL; International Society of Urological Pathology (ISUP) grade ≤ 2; MRI-fusion biopsy-confirmed lesion classified as PI-RADS v2.1 ≥ 3. Intra-, peri-, and post-operative data were collected. Variables including age, PSA, prostate volume (PVol), Charlson's Comorbidity Index (CCI), International Prostate Symptom Score (IPSS) with QoL score, International Index of Erectile Function (IIEF-5), International Consultation on Incontinence Questionnaire-Short Form (ICIQ-SF), and Male Sexual Health Questionnaire-Ejaculatory Dysfunction Short Form (MSHQ-EjD) were collected at baseline and at 3, 6 and 12 months after TPLA. Post-operative mpMRI was performed at 3 and 12 months. Finally, all patients underwent prostatic re-biopsy under fusion guidance at 12 months. The success of this technique was defined as no recurrence in the target treated lesion at the 12-month follow up.

Results: Twenty-four patients underwent focal TPLA. Baseline features were age [median 67 years (IQR 12)], PSA [5.7 ng/mL (3.9)], PVol [49 mL (27)], CCI [0 (0)], IPSS [11 (9)], IPSS-QoL [2 (2)], IIEF-5 [21 (6)], ICIQ-SF [0 (7)], MSHQ-EjD ejaculation domain [14 (4)] and bother score [0 (2)]. Median operative time was 34 min (IQR 12). Median

visual analogue scale (VAS) 6 h after TPLA was 0 (IQR 1). The post-operative course was regular for all patients, who were discharged on the second post-operative day and underwent catheter removal on the seventh post-operative day. No patient had incontinence at catheter removal. A significant reduction in PSA ($p = 0.01$) and an improvement in IPSS ($p = 0.009$), IPSS-QoL ($p = 0.02$) and ICIQ-SF scores ($p = 0.04$) compared to baseline were observed at the 3-month follow-up. Erectile and ejaculatory functions did not show any significant variation during the follow-up. No intra- and peri-operative complications were recorded. Three Clavien-Dindo post-operative complications were recorded (12%): grade 1 (two cases of urinary retention) and grade 2 (one case of urinary tract infection). At the 12-month follow-up, eight patients showed mpMRI images referable to suspicious recurrent disease (PIRADS v2.1 ≥ 3). After re-biopsy, 7/24 patients' (29%) results were histologically confirmed as PCa, 3 of which were recurrences in the treated lesion (12.5%). The success rate was 87.5%.

Conclusions: The focal TPLA oncological and functional results seemed to be encouraging. TPLA is a safe, painless, and effective technique with a good preservation of continence and sexual outcomes. Recurrence rate at 12 months was about 12.5%.

Keywords:

TPLA, focal therapy, prostate cancer

<u>Original Lay Summary</u>

Prostate cancer is the second most diagnosed cancer in men. Focal laser ablation has been proposed as an alternative to radical treatments in carefully selected patients in order to achieve long-term cancer control and reduce the morbidity associated with surgery and radiation therapy. This study aimed to evaluate the oncological and functional outcomes of transperineal laser ablation (TPLA) as the focal therapy for localized prostate cancer. We assessed that TPLA is a safe, painless, and effective technique with good oncological results and the preservation of continence and sexual outcomes.

**Assessment Category**	**Score**	**Assessment Category**	**Score**	**Assessment Category**	**Score**
Comprehension Score Rater 1	4	Gunning Fog Score	19.7	Words	90
Comprehension Score Rater 2	4	SMOG Index	14.1	Complex words	24
Reading Age (years)	22.5	Coleman Liau Index	17.0	Percentage of complex words	26.67
Flesch-Kincaid Reading Ease	22.3	Automated Readability Index	16.1	Average words per sentence	50.0
Flesch-Kincaid Grade Level	15.7	Sentences	4	Average syllables per word	1.91

Lay Summary (Simple Prompt)

Prostate cancer is one of the most common cancers in men, and new treatments aim to target the cancer while preserving quality of life. This study explored the use of a minimally invasive technique called transperineal laser ablation (TPLA) as a focal therapy for men with localized prostate cancer. The goal was to treat only the cancerous area, reducing the risk of side effects like incontinence and sexual dysfunction. Twenty-four men with low- to intermediate-risk prostate cancer underwent TPLA and were followed for 12 months. The procedure was safe, with no serious complications, and most patients maintained normal urinary and sexual functions. After a year, about 88% of the patients had no cancer recurrence in the treated area. These results suggest that TPLA could be an effective, safe, and less invasive option for men with localized prostate cancer, offering good cancer control while preserving quality of life.

**Assessment Category**	**Score**	**Assessment Category**	**Score**	**Assessment Category**	**Score**
Comprehension Score Rater 1	5	Gunning Fog Score	16.0	Words	149
Comprehension Score Rater 2	5	SMOG Index	11.6	Complex words	28
Reading Age (years)	18.5	Coleman Liau Index	14.1	Percentage of complex words	18.79
Flesch-Kincaid Reading Ease	44.4	Automated Readability Index	13.2	Average words per sentence	21.29
Flesch-Kincaid Grade Level	12.4	Sentences	7	Average syllables per word	1.66

<u>Lay Summary (Extended Prompt)</u>

This study looked at a treatment called transperineal laser ablation (TPLA) for men with early-stage prostate cancer. The goal was to see how well TPLA worked after 12 months, in terms of both cancer control and preserving quality of life. Twenty-four patients were treated, and their cancer was closely monitored using scans and biopsies. The results showed that TPLA was a safe and effective treatment, with most men (87.5%) not having cancer come back in the treated area. Men also experienced improvements in urinary symptoms, with no major side effects. While a small number of patients had cancer recurrence, TPLA showed promise as a way to treat prostate cancer without surgery, with minimal impact on sexual or urinary function. This research could offer a new, less invasive option for treating prostate cancer.

**Assessment Category**	**Score**	**Assessment Category**	**Score**	**Assessment Category**	**Score**
Comprehension Score Rater 1	4	Gunning Fog Score	11.2	Words	135
Comprehension Score Rater 2	4	SMOG Index	8.0	Complex words	15
Reading Age (years)	15.5	Coleman Liau Index	13.1	Percentage of complex words	11.11
Flesch-Kincaid Reading Ease	59.4	Automated Readability Index	10.1	Average words per sentence	16.88
Flesch-Kincaid Grade Level	9.2	Sentences	8	Average syllables per word	1.54

Manuscript 91

Sanders JL, Iczkowski KA, Shah GV. Predicting the Diagnosis of Prostate Cancer with a Novel Blood-Based Biomarker: Comparison of Its Performance with Prostate-Specific Antigen. Cancers [Internet]. 2024 Jul 23;16(15):2619. Available from: http://dx.doi.org/10.3390/cancers16152619

Abstract

The purpose of this study was to assess the efficacy, specificity, and predictive value of a newly discovered biomarker, Zinc finger-like1 protein (referred to as neuroendocrine marker, NEM) for the detection of prostate cancer (PCa). We retrospectively analyzed banked plasma samples from 508 men, with a median age of 67 years (range 48-97), to compare the performance of NEM and PSA in predicting subsequent histologic PCa. The cohort consisted of four groups of patients visiting a urology clinic: (1) patients not diagnosed with either benign prostatic disease or prostate cancer (PCa) were defined as normal; (2) patients diagnosed with benign hyperplasia (BPH) but not PCa; (3) patients with confirmed PCa; and (4) patients with cancer other than PCa. The normal men displayed a mean NEM plasma level of 0.948 ± 0.051 ng/mL, which increased to 1.813 ± 0.315 ng/mL in men with BPH, 86.49 ± 15.51 ng/mL in men with PCa, and 10.47 ± 1.029 ng/mL in men with other Ca. The corresponding concentrations of prostate-specific antigen (PSA) in these subjects were 1.787 ± 0.135, 5.405 ± 0.699, 35.77 ± 11.48 ng/mL, and 8.036 ± 0.518, respectively. Receiver operating characteristic (ROC) curve analysis was performed to compare NEM and PSA performance, and the Jouden Index for each biomarker was calculated to determine cut-off points for each biomarker. The area under the ROC curve to predict PCa was 0.99 for NEM and 0.81 for PSA ($p < 0.0001$). The cut-off for NEM was at 1.9 ng/mL, with sensitivity of 98% and specificity of 97%. The corresponding PSA values were 4.4 ng/mL, with sensitivity of 76% and specificity of 95%. The predictive value of each biomarker in a patient was matched with his pathologic data to determine the accuracy of each biomarker. NEM was more accurate than PSA in differentiating cancer from benign conditions, such as BPH or

prostatitis. In conclusion, NEM was a better predictor of PCa than PSA in patients visiting urology clinics. NEM tests, either alone or in conjunction with other biomarkers, provide a reliable, non-invasive, and inexpensive test to remarkably reduce false positives and thereby reduce the number of diagnostic biopsies and associated painful procedures and the loss of quality of life.

Keywords:

clinical studies, novel blood-based biomarker, prostate cancer, validation of a biomarker

Original Lay Summary

For decades, a blood test showing elevated prostate-specific antigen (PSA) has been the mainstay for detecting prostate cancer. However, the PSA is often elevated in the absence of cancer. Here, we show that the neuroendocrine marker (NEM) outperforms the PSA test, both for sensitivity and for the exclusion of false-positive results.

**Assessment Category**	**Score**	**Assessment Category**	**Score**	**Assessment Category**	**Score**
Comprehension Score Rater 1	3	Gunning Fog Score	15.4	Words	53
Comprehension Score Rater 2	2	SMOG Index	11.1	Complex words	11
Reading Age (years)	18.5	Coleman Liau Index	14.4	Percentage of complex words	20.75
Flesch-Kincaid Reading Ease	45.2	Automated Readability Index	11.6	Average words per sentence	17.67
Flesch-Kincaid Grade Level	11.3	Sentences	3	Average syllables per word	1.7

<u>Lay Summary (Simple Prompt)</u>

Prostate cancer is a common disease in men, and early detection is key to successful treatment. Currently, prostate-specific antigen (PSA) is the standard blood test used to detect prostate cancer, but it often leads to false positives, causing unnecessary biopsies and stress. This study evaluated a new blood-based biomarker called Zinc finger-like1 protein, or NEM, to see if it could more accurately identify prostate cancer. Researchers analyzed blood samples from over 500 men and compared the performance of NEM with PSA. The results showed that NEM was significantly more accurate than PSA in detecting prostate cancer and better at distinguishing cancer from non-cancerous conditions like benign prostate enlargement. NEM correctly identified cancer in 98% of cases and reduced false positives, which means fewer men would need unnecessary biopsies. This new test could improve prostate cancer screening, making it more reliable, less invasive, and reducing the risk of unnecessary procedures.

**Assessment Category**	**Score**	**Assessment Category**	**Score**	**Assessment Category**	**Score**
Comprehension Score Rater 1	5	Gunning Fog Score	17.1	Words	153
Comprehension Score Rater 2	5	SMOG Index	12.4	Complex words	32
Reading Age (years)	20.5	Coleman Liau Index	15.9	Percentage of complex words	20.92
Flesch-Kincaid Reading Ease	35.4	Automated Readability Index	14.9	Average words per sentence	21.86
Flesch-Kincaid Grade Level	13.8	Sentences	7	Average syllables per word	1.76

Lay Summary (Extended Prompt)

Prostate cancer is a common disease in men, often detected using a blood test called prostate-specific antigen (PSA). However, PSA tests can sometimes give false results, leading to unnecessary and painful procedures. In this study, we explored a new blood-based marker called Zinc finger-like1 protein (NEM) to see if it could detect prostate cancer more accurately than PSA. We tested blood samples from over 500 men, some with prostate cancer, some with other conditions, and some healthy. The results showed that NEM was much better at identifying prostate cancer correctly, with higher accuracy and fewer false alarms than PSA. This means that using NEM, either alone or with other tests, could help reduce the number of unnecessary biopsies, making prostate cancer detection safer, quicker, and less stressful for patients.

**Assessment Category**	**Score**	**Assessment Category**	**Score**	**Assessment Category**	**Score**
Comprehension Score Rater 1	5	Gunning Fog Score	13.3	Words	132
Comprehension Score Rater 2	5	SMOG Index	9.2	Complex words	15
Reading Age (years)	17.5	Coleman Liau Index	14.1	Percentage of complex words	11.36
Flesch-Kincaid Reading Ease	46.1	Automated Readability Index	13.5	Average words per sentence	22.0
Flesch-Kincaid Grade Level	12.3	Sentences	6	Average syllables per word	1.64

Manuscript 92

Pietrzak S, Marciniak W, Derkacz R, Matuszczak M, Kiljańczyk A, Baszuk P, et al. Cobalt Serum Level as a Biomarker of Cause-Specific Survival among Prostate Cancer Patients. Cancers [Internet]. 2024 Jul 23;16(15):2618. Available from: http://dx.doi.org/10.3390/cancers16152618

Abstract

Prostate cancer is the most common cancer diagnosed in men and the second leading cause of death in male cancer patients. The WHO suggests that cobalt is involved in the carcinogenesis of prostate cancer. There are, however, no studies associating cobalt levels and prostate cancer patient survival. In this study, 261 Polish prostate cancer (n = 261) patients were recruited into a prospective cohort between 2009 and 2015. Serum cobalt levels were measured using ICP-MS after prostate cancer diagnosis and before treatment. All study participants were assigned into quartiles (QI-QIV) based on the distribution of serum cobalt levels among censored patients. Univariable and multivariable COX regression models were used to calculate hazard ratios (HRs) for each serum cobalt level quartile. We found a significant relationship between high serum cobalt levels and poor prostate cancer patient total survival (HR = 2.60; 95% CI: 1.17-5.82; p = 0.02). In relation to prostate cancer patients who died as a result of other non-cancer causes, the association with high levels of cobalt was even stronger (HR = 3.67; 95% CI: 1.03-13.00; p = 0.04). The impact of high serum cobalt levels on overall survival of prostate cancer-specific-related deaths was not statistically significant.

Keywords:

biomarker, cobalt, prostate cancer, survival

Original Lay Summary

Prostate cancer is the most common cancer detected among men and it is the second leading cause of death. According to the WHO, cobalt is probably involved in carcinogenesis. However, there are no studies related to cobalt levels and survival in prostate cancer patients. The aim of this prospective study was to investigate the relationship between serum cobalt levels and survival among prostate cancer patients taking into consideration prostate cancer-specific deaths and non-cancer causes of death. Our findings, based on 261 Polish prostate cancer patients, show that individuals with high serum cobalt levels have a significantly worse survival compared to participants with low serum cobalt levels.

**Assessment Category**	**Score**	**Assessment Category**	**Score**	**Assessment Category**	**Score**
Comprehension Score Rater 1	3	Gunning Fog Score	14.9	Words	108
Comprehension Score Rater 2	3	SMOG Index	10.7	Complex words	17
Reading Age (years)	19.5	Coleman Liau Index	16.2	Percentage of complex words	15.74
Flesch-Kincaid Reading Ease	32.2	Automated Readability Index	15.0	Average words per sentence	21.6
Flesch-Kincaid Grade Level	14.1	Sentences	5	Average syllables per word	1.81

Lay Summary (Simple Prompt)

Prostate cancer is the most common cancer in men and a major cause of cancer-related deaths. Scientists are looking for new ways to predict how well patients might do after being diagnosed. This study explored whether the amount of cobalt, a naturally occurring metal, in the blood could help predict survival in men with prostate cancer. Researchers measured blood cobalt levels in 261 Polish men after their diagnosis but before starting treatment. They then tracked these patients over time to see how their cobalt levels related to their health outcomes. The results showed that men with higher cobalt levels in their blood had a greater risk of dying, especially from causes not related to cancer. However, cobalt levels did not significantly predict deaths specifically from prostate cancer. This research suggests that cobalt in the blood might serve as a useful marker to help doctors better understand a patient's overall health risks.

**Assessment Category**	**Score**	**Assessment Category**	**Score**	**Assessment Category**	**Score**
Comprehension Score Rater 1	4	Gunning Fog Score	11.5	Words	152
Comprehension Score Rater 2	4	SMOG Index	8.0	Complex words	15
Reading Age (years)	16.5	Coleman Liau Index	14.3	Percentage of complex words	9.87
Flesch-Kincaid Reading Ease	54.0	Automated Readability Index	12.1	Average words per sentence	19.0
Flesch-Kincaid Grade Level	10.5	Sentences	8	Average syllables per word	1.58

Lay Summary (Extended Prompt)

Prostate cancer is the most common cancer in men and can be life-threatening. Scientists are looking for ways to predict how prostate cancer might affect a person's health over time. In this study, we explored whether the amount of cobalt, a natural metal found in the body, could help predict survival in men with prostate cancer. We measured cobalt levels in the blood of 261 men in Poland after their diagnosis but before they started treatment. We then followed these men to see how their health changed over time. Our results showed that men with higher cobalt levels in their blood were more likely to die from causes other than prostate cancer compared to those with lower levels. This suggests that cobalt might be a useful marker to identify patients at higher risk, which could help doctors make better decisions about care and monitoring.

**Assessment Category**	**Score**	**Assessment Category**	**Score**	**Assessment Category**	**Score**
Comprehension Score Rater 1	4	Gunning Fog Score	10.2	Words	145
Comprehension Score Rater 2	4	SMOG Index	6.0	Complex words	7
Reading Age (years)	15.5	Coleman Liau Index	12.2	Percentage of complex words	4.83
Flesch-Kincaid Reading Ease	61.5	Automated Readability Index	11.3	Average words per sentence	20.71
Flesch-Kincaid Grade Level	9.8	Sentences	7	Average syllables per word	1.47

Manuscript 93

Lin SC, Yu G, Corn PG, Damasco J, Lee YC, Song JH, et al. Radium-223 Treatment Produces Prolonged Suppression of Resident Osteoblasts and Decreased Bone Mineral Density in Trabecular Bone in Osteoblast Reporter Mice. Cancers [Internet]. 2024 Jul 21;16(14):2603. Available from: http://dx.doi.org/10.3390/cancers16142603

Abstract

Radium 223 (Ra-223) is an α-emitting bone-homing radiopharmaceutical that targets tumor-induced osteoblasts and is used to reduce bone pain and prolong overall survival in men with bone-metastatic, castrate-resistant prostate cancer. However, increased fracture risk in skeletal sites with no bone metastasis has been observed in patients treated with Ra-223. Both luciferase- or green fluorescence protein (GFP)-labeled osteoblast reporter mice were used to monitor the effect of Ra-223 on resident osteoblasts and normal bone structure. Upon Ra-223 treatment, 70% of resident osteoblasts were reduced within 2 days, and the osteoblast reduction lasted for at least 18 weeks without detectable recovery, as measured by in vivo bioluminescent imaging. In GFP-labeled osteoblast reporter mice, Ra-223 mainly reduced osteoblasts localized in the trabecular bone areas; the osteoblasts in the growth plates were less affected. Micro-computed tomography analyses showed that Ra-223 significantly reduced bone mineral density and bone microstructure in the trabecular area of femurs but not in the cortical bone. Tumor-induced bone was generated by inoculating osteogenic TRAMP-BMP4 prostate cancer cells into the mouse femurs; Ra-223 treatment significantly reduced tumor-induced osteoblasts. Our study shows that Ra-223 affects bone structures that are not involved in bone metastasis. Strategies that improve bone health may reduce fracture risk in patients receiving Ra-223.

Keywords:

Radium-223, bone mass, osteoblast reporter mice, osteoblasts, prostate cancer

<u>Original Lay Summary</u>

Radium 223 (Ra-223) is a radiopharmaceutical that targets tumor-induced osteoblasts (bone-forming cells). Ra-223 reduces bone pain and prolongs overall survival in men with bone-metastatic, castrate-resistant prostate cancer. However, increased fracture risk in skeletal sites with no bone metastasis has been observed in patients treated with Ra-223. The aim of this study was to examine the effects of Ra-223 on resident osteoblasts and normal bone structure in mouse models. Upon Ra-223 treatment, 70% of resident osteoblasts were reduced within 2 days, and the reduction lasted for at least 18 weeks. Ra-223 reduced the osteoblasts mainly localized in trabecular bone areas. Ra-223 also reduced bone mineral density and altered bone microstructure in the trabecular area of femurs. Furthermore, Ra-223 treatment also significantly reduced tumor-induced osteoblasts. These studies show that Ra-223 affects the structure of bones that are not involved in bone metastasis. Strategies that improve bone health may reduce fracture risk in patients receiving Ra-223.

**Assessment Category**	**Score**	**Assessment Category**	**Score**	**Assessment Category**	**Score**
Comprehension Score Rater 1	5	Gunning Fog Score	13.4	Words	168
Comprehension Score Rater 2	5	SMOG Index	9.9	Complex words	29
Reading Age (years)	17.5	Coleman Liau Index	13.6	Percentage of complex words	17.26
Flesch-Kincaid Reading Ease	51.8	Automated Readability Index	10.5	Average words per sentence	16.8
Flesch-Kincaid Grade Level	10.2	Sentences	10	Average syllables per word	1.63

Lay Summary (Simple Prompt)

Radium-223 is a treatment used to help men with advanced prostate cancer that has spread to their bones, as it can ease bone pain and help them live longer. However, patients receiving this treatment sometimes experience bone fractures in areas without cancer. To understand why this happens, researchers studied mice specially designed to show how bone-forming cells, called osteoblasts, respond to Radium-223. The study found that Radium-223 quickly reduced the number of healthy osteoblasts in certain parts of the bones, and this reduction lasted for many weeks without signs of recovery. Additionally, the treated bones showed a decrease in bone strength and density, making them more fragile. These findings suggest that while Radium-223 is effective against cancer-related bone issues, it can also weaken healthy bones. This highlights the need for strategies to protect bone health in patients receiving this treatment, potentially reducing their risk of fractures.

**Assessment Category**	**Score**	**Assessment Category**	**Score**	**Assessment Category**	**Score**
Comprehension Score Rater 1	5	Gunning Fog Score	13.7	Words	152
Comprehension Score Rater 2	5	SMOG Index	10.5	Complex words	23
Reading Age (years)	18.5	Coleman Liau Index	14.9	Percentage of complex words	15.13
Flesch-Kincaid Reading Ease	51.2	Automated Readability Index	14.0	Average words per sentence	21.71
Flesch-Kincaid Grade Level	11.5	Sentences	7	Average syllables per word	1.58

<u>Lay Summary (Extended Prompt)</u>

Radium-223 is a medicine used to help men with prostate cancer that has spread to their bones. It helps reduce bone pain and helps patients live longer. However, some people treated with Radium-223 have a higher risk of bone fractures, even in bones without cancer. This study looked at how Radium-223 affects healthy bone cells called osteoblasts, which help keep bones strong. Using special mice that allow us to track these bone cells, we found that Radium-223 quickly reduces the number of healthy osteoblasts, and this effect can last for several months. The loss of these cells leads to weaker bones, especially in the inner, spongy parts. Interestingly, Radium-223 had less effect on the dense outer bone. These findings show that Radium-223 can weaken healthy bones, not just cancer-affected ones. This highlights the need for ways to protect bone health in patients receiving Radium-223 to lower their risk of fractures.

**Assessment Category**	**Score**	**Assessment Category**	**Score**	**Assessment Category**	**Score**
Comprehension Score Rater 1	5	Gunning Fog Score	9.3	Words	158
Comprehension Score Rater 2	5	SMOG Index	7.8	Complex words	16
Reading Age (years)	14.5	Coleman Liau Index	11.5	Percentage of complex words	10.13
Flesch-Kincaid Reading Ease	67.5	Automated Readability Index	9.2	Average words per sentence	17.56
Flesch-Kincaid Grade Level	8.2	Sentences	9	Average syllables per word	1.44

Manuscript 94

Zhou T, Nguyen S, Wu J, He B, Feng Q. LncRNA LOC730101 Promotes Darolutamide Resistance in Prostate Cancer by Suppressing miR-1-3p. Cancers [Internet]. 2024 Jul 20;16(14):2594. Available from: http://dx.doi.org/10.3390/cancers16142594

Abstract

Antiandrogen is part of the standard-of-care treatment option for metastatic prostate cancer. However, prostate cancers frequently relapse, and the underlying resistance mechanism remains incompletely understood. This study seeks to investigate whether long non-coding RNAs (lncRNAs) contribute to the resistance against the latest antiandrogen drug, darolutamide. Our RNA sequencing analysis revealed significant overexpression of LOC730101 in darolutamide-resistant cancer cells compared to the parental cells. Elevated LOC730101 levels were also observed in clinical samples of metastatic castration-resistant prostate cancer (CRPC) compared to primary prostate cancer samples. Silencing LOC730101 with siRNA significantly impaired the growth of darolutamide-resistant cells. Additional RNA sequencing analysis identified a set of genes regulated by LOC730101, including key players in the cell cycle regulatory pathway. We further demonstrated that LOC730101 promotes darolutamide resistance by competitively inhibiting microRNA miR-1-3p. Moreover, by Hi-C sequencing, we found that LOC730101 is located in a topologically associating domain (TAD) that undergoes specific gene induction in darolutamide-resistant cells. Collectively, our study demonstrates the crucial role of the lncRNA LOC730101 in darolutamide resistance and its potential as a target for overcoming antiandrogen resistance in CRPC.

Keywords:

antiandrogen, darolutamide, lncRNA, miRNA, prostate cancer

<u>Original Lay Summary</u>

This study investigates the role of non-coding RNAs in resistance to the antiandrogen drug darolutamide in prostate cancer. RNA sequencing identified LOC730101 as a significantly elevated lncRNA in darolutamide-resistant cancer cells. Silencing LOC730101 reduced cancer cell proliferation, in support of its role in drug-resistant cancer growth. Elevated LOC730101 levels were observed in metastatic castration-resistant prostate cancer (CRPC) tumors compared to primary prostate tumors. LOC730101 modulates the expression of cell cycle genes by suppressing microRNA miR-1-3p. Hi-C sequencing revealed that the LOC730101 gene is situated within a dynamic topologically associating domain (TAD) that is activated in resistant cells. This study indicates that LOC730101 is crucial for darolutamide resistance and a potential target to overcome antiandrogen resistance in CRPC.

**Assessment Category**	**Score**	**Assessment Category**	**Score**	**Assessment Category**	**Score**
Comprehension Score Rater 1	5	Gunning Fog Score	18.7	Words	124
Comprehension Score Rater 2	5	SMOG Index	13.1	Complex words	36
Reading Age (years)	21.5	Coleman Liau Index	17.5	Percentage of complex words	29.03
Flesch-Kincaid Reading Ease	24.4	Automated Readability Index	14.1	Average words per sentence	17.71
Flesch-Kincaid Grade Level	14.3	Sentences	7	Average syllables per word	1.94

Lay Summary (Simple Prompt)

Prostate cancer is often treated with antiandrogen drugs, like darolutamide, which block hormones that help cancer grow. Unfortunately, many patients eventually become resistant to these drugs, and the reasons for this are not fully understood. In this study, researchers explored whether a molecule called LOC730101, a type of long non-coding RNA (lncRNA), plays a role in this resistance. They found that LOC730101 levels were much higher in drug-resistant prostate cancer cells compared to non-resistant ones. When they reduced LOC730101 in these cells, the cancer's growth slowed down. The study also showed that LOC730101 helps cancer cells resist treatment by blocking another molecule, miR-1-3p, which normally helps control cell growth. These findings suggest that targeting LOC730101 could be a new way to overcome drug resistance in prostate cancer, potentially improving treatment outcomes for patients with advanced disease.

**Assessment Category**	**Score**	**Assessment Category**	**Score**	**Assessment Category**	**Score**
Comprehension Score Rater 1	5	Gunning Fog Score	13.4	Words	141
Comprehension Score Rater 2	5	SMOG Index	9.6	Complex words	19
Reading Age (years)	18.5	Coleman Liau Index	15.2	Percentage of complex words	13.48
Flesch-Kincaid Reading Ease	52.6	Automated Readability Index	13.5	Average words per sentence	20.14
Flesch-Kincaid Grade Level	10.9	Sentences	7	Average syllables per word	1.58

Lay Summary (Extended Prompt)

Prostate cancer is often treated with drugs called antiandrogens, like darolutamide, which help slow the cancer's growth. Unfortunately, some prostate cancers stop responding to these drugs over time, but the reasons for this are not fully understood. In this study, we looked at a molecule called LOC730101, which is a type of RNA that does not make proteins but can still affect how cells work. We found that LOC730101 is present at much higher levels in prostate cancer cells that are resistant to darolutamide. When we blocked LOC730101 in these resistant cells, the cancer cells grew much more slowly. We also discovered that LOC730101 interferes with another small molecule called miR-1-3p, which normally helps control cell growth. Our findings suggest that LOC730101 plays an important role in making prostate cancer resistant to treatment, and targeting this molecule might help develop new ways to treat advanced prostate cancer.

**Assessment Category**	**Score**	**Assessment Category**	**Score**	**Assessment Category**	**Score**
Comprehension Score Rater 1	5	Gunning Fog Score	13.6	Words	149
Comprehension Score Rater 2	5	SMOG Index	9.6	Complex words	19
Reading Age (years)	17.5	Coleman Liau Index	13.5	Percentage of complex words	12.75
Flesch-Kincaid Reading Ease	58.6	Automated Readability Index	12.7	Average words per sentence	21.29
Flesch-Kincaid Grade Level	10.4	Sentences	7	Average syllables per word	1.5

Manuscript 95

Perez-Navarro E, Conteduca V, Funes JM, Dominguez JI, Martin-Serrano M, Cremaschi P, et al. Prognostic Implications of Blood Immune-Cell Composition in Metastatic Castration-Resistant Prostate Cancer. Cancers [Internet]. 2024 Jul 14;16(14):2535. Available from: http://dx.doi.org/10.3390/cancers16142535

Abstract

The prognosis for patients with metastatic castration-resistant prostate cancer (mCRPC) varies, being influenced by blood-related factors such as transcriptional profiling and immune cell ratios. We aimed to address the contribution of distinct whole blood immune cell components to the prognosis of these patients. This study analyzed pre-treatment blood samples from 152 chemotherapy-naive mCRPC patients participating in a phase 2 clinical trial (NCT02288936) and a validation cohort. We used CIBERSORT-X to quantify 22 immune cell types and assessed their prognostic significance using Kaplan-Meier and Cox regression analyses. Reduced CD8 T-cell proportions and elevated monocyte levels were substantially connected with a worse survival. High monocyte counts correlated with a median survival of 32.2 months versus 40.3 months for lower counts (HR: 1.96, 95% CI 1.11-3.45). Low CD8 T-cell levels were associated with a median survival of 31.8 months compared to 40.3 months for higher levels (HR: 1.97, 95% CI 1.11-3.5). These findings were consistent in both the trial and validation cohorts. Multivariate analysis further confirmed the independent prognostic value of CD8 T-cell counts. This study highlights the prognostic implications of specific blood immune cells, suggesting they could serve as biomarkers in mCRPC patient management and should be further explored in clinical trials.

Keywords:

castration-resistant prostate cancer (CRPC), enzalutamide, prognostic factors, prostate cancer, whole blood

<u>Original Lay Summary</u>

Metastatic castration-resistant prostate cancer (mCRPC) represents a lethal stage of prostate cancer, characterized for its resistance to androgen deprivation therapy and variable survival outcomes. This study investigates how the composition of specific immune cells in the blood affects the prognosis of mCRPC patients who have not yet received chemotherapy. In looking at blood samples taken before treatment with the drug enzalutamide, we discovered significant correlations between lower levels of CD8 T cells and higher levels of monocytes, which were consistently linked to poorer survival rates. The prognostic value of blood CD8 T cells was independently validated in multivariate prognostic models and in an independent cohort of mCRPC patients. This study highlights the feasibility of blood immune-cell profiling in patients included in clinical trials and the association of blood CD8 T cells with the prognosis for mCRPC patients.

**Assessment Category**	**Score**	**Assessment Category**	**Score**	**Assessment Category**	**Score**
Comprehension Score Rater 1	5	Gunning Fog Score	20.9	Words	139
Comprehension Score Rater 2	5	SMOG Index	15.2	Complex words	35
Reading Age (years)	23.5	Coleman Liau Index	17.8	Percentage of complex words	25.18
Flesch-Kincaid Reading Ease	17.9	Automated Readability Index	19.3	Average words per sentence	27.8
Flesch-Kincaid Grade Level	17.7	Sentences	5	Average syllables per word	1.9

Lay Summary (Simple Prompt)

Prostate cancer that no longer responds to hormone therapy, known as castration-resistant prostate cancer, can behave very differently from patient to patient. This study aimed to find out if certain immune cells in the blood could help predict how long these patients might live. Researchers analyzed blood samples from 152 men with this type of prostate cancer before they started treatment. They discovered that patients with fewer CD8 T-cells, which help the body fight cancer, and higher levels of monocytes, a type of white blood cell, tended to have shorter survival times. These results were confirmed in an additional group of patients. Understanding how these immune cells are linked to survival could help doctors better predict outcomes and guide treatment decisions for men with advanced prostate cancer in the future.

**Assessment Category**	**Score**	**Assessment Category**	**Score**	**Assessment Category**	**Score**
Comprehension Score Rater 1	5	Gunning Fog Score	12.7	Words	132
Comprehension Score Rater 2	5	SMOG Index	8.6	Complex words	13
Reading Age (years)	17.5	Coleman Liau Index	14.7	Percentage of complex words	9.85
Flesch-Kincaid Reading Ease	55.0	Automated Readability Index	14.0	Average words per sentence	22.0
Flesch-Kincaid Grade Level	11.0	Sentences	6	Average syllables per word	1.53

Lay Summary (Extended Prompt)

Prostate cancer that no longer responds to hormone therapy, known as castration-resistant prostate cancer, can spread to other parts of the body and affect survival differently in each patient. This study looked at how certain immune cells in the blood might help predict how long these patients might live. Researchers analyzed blood samples from 152 patients before they started chemotherapy, focusing on specific types of immune cells. They found that patients with lower levels of CD8 T-cells (a type of immune cell that fights cancer) and higher levels of monocytes (another immune cell type) had shorter survival times compared to those with healthier levels. These results suggest that measuring certain immune cells in the blood could help doctors better understand a patient's outlook and potentially guide future treatment decisions. This information may improve how we manage prostate cancer in the future.

**Assessment Category**	**Score**	**Assessment Category**	**Score**	**Assessment Category**	**Score**
Comprehension Score Rater 1	5	Gunning Fog Score	14.8	Words	143
Comprehension Score Rater 2	5	SMOG Index	10.3	Complex words	19
Reading Age (years)	19.5	Coleman Liau Index	15.4	Percentage of complex words	13.29
Flesch-Kincaid Reading Ease	45.4	Automated Readability Index	15.4	Average words per sentence	23.83
Flesch-Kincaid Grade Level	12.8	Sentences	6	Average syllables per word	1.62

Manuscript 96

Cattabriga A, Renzetti B, Galuppi F, Bartalena L, Gaudiano C, Brocchi S, et al. Multiparametric Whole-Body MRI: A Game Changer in Metastatic Prostate Cancer. Cancers [Internet]. 2024 Jul 13;16(14):2531. Available from: http://dx.doi.org/10.3390/cancers16142531

Abstract

Prostate cancer ranks among the most prevalent tumours globally. While early detection reduces the likelihood of metastasis, managing advanced cases poses challenges in diagnosis and treatment. Current international guidelines support the concurrent use of 99Tc-Bone Scintigraphy and Contrast-Enhanced Chest and Abdomen CT for the staging of metastatic disease and response assessment. However, emerging evidence underscores the superiority of next-generation imaging techniques including PSMA-PET/CT and whole-body MRI (WB-MRI). This review explores the relevant scientific literature on the role of WB-MRI in metastatic prostate cancer. This multiparametric imaging technique, combining the high anatomical resolution of standard MRI sequences with functional sequences such as diffusion-weighted imaging (DWI) and bone marrow relative fat fraction (rFF%) has proved effective in comprehensive patient assessment, evaluating local disease, most of the nodal involvement, bone metastases and their complications, and detecting the increasing visceral metastases in prostate cancer. It does have the advantage of avoiding the injection of contrast medium/radionuclide administration, spares the patient the exposure to ionizing radiation, and lacks the confounder of FLARE described with nuclear medicine techniques. Up-to-date literature regarding the diagnostic capabilities of WB-MRI, though still limited compared to PSMA-PET/CT, strongly supports its widespread incorporation into standard clinical practice, alongside the latest nuclear medicine techniques.

Keywords:

MRI, metastatic prostate cancer, oncological radiology, prostate cancer, radiology, whole-body MRI

<u>Original Lay Summary</u>

In the realm of next-generation imaging, whole-body MRI (WB-MRI) is evolving as a key player in the modern management of patients with prostate cancer, showing great potential in the initial staging of high-risk disease, post-treatment evaluation, and recurrence assessment. This technique showcases both notable agreement and complementarity with PET/CT, particularly in identifying secondary bone, nodal, and visceral lesions. WB-MRI's strength lies in its "one size fits all" nature, avoiding contrast agents and radiotracers and adapting to diverse patient needs. Leveraging DWI and rFF%, it excels in bone metastasis detection, offering a comprehensive view. With promising potential for precise response assessment, WB-MRI emerges as a transformative tool in the management of metastatic prostate cancer.

**Assessment Category**	**Score**	**Assessment Category**	**Score**	**Assessment Category**	**Score**
Comprehension Score Rater 1	5	Gunning Fog Score	20.3	Words	120
Comprehension Score Rater 2	5	SMOG Index	14.6	Complex words	32
Reading Age (years)	22.5	Coleman Liau Index	17.0	Percentage of complex words	26.67
Flesch-Kincaid Reading Ease	23.1	Automated Readability Index	16.8	Average words per sentence	24.0
Flesch-Kincaid Grade Level	16.0	Sentences	5	Average syllables per word	1.88

<u>Lay Summary (Simple Prompt)</u>

Prostate cancer is one of the most common cancers worldwide, and when it spreads to other parts of the body (metastatic prostate cancer), it becomes more difficult to manage. Traditionally, doctors use a combination of bone scans and CT scans to detect and monitor this spread. However, new imaging methods are proving to be more effective. This study focuses on whole-body MRI, an advanced technique that provides detailed images of the entire body without exposing patients to radiation or requiring contrast injections. It can detect cancer in bones, lymph nodes, and even organs more accurately, helping doctors assess the disease more comprehensively. Whole-body MRI also avoids some of the limitations seen with other imaging methods. The findings suggest that this technique could play a key role in improving the diagnosis and monitoring of metastatic prostate cancer, potentially leading to better treatment decisions and outcomes for patients.

**Assessment Category**	**Score**	**Assessment Category**	**Score**	**Assessment Category**	**Score**
Comprehension Score Rater 1	5	Gunning Fog Score	14.9	Words	148
Comprehension Score Rater 2	5	SMOG Index	10.7	Complex words	24
Reading Age (years)	19.5	Coleman Liau Index	15.5	Percentage of complex words	16.22
Flesch-Kincaid Reading Ease	39.6	Automated Readability Index	14.2	Average words per sentence	21.14
Flesch-Kincaid Grade Level	13.0	Sentences	7	Average syllables per word	1.72

<u>Lay Summary (Extended Prompt)</u>

Prostate cancer is one of the most common cancers in men, and when it spreads to other parts of the body (metastatic prostate cancer), it becomes harder to diagnose and treat. Doctors usually rely on bone scans and CT scans to find and track these cancer spread areas, but newer imaging methods are showing better results. This study focuses on a powerful imaging technique called whole-body MRI, which provides detailed pictures of the body without using radiation or contrast dyes. It can spot cancer in bones, lymph nodes, and other organs more effectively and safely than some traditional methods. Whole-body MRI combines different scanning techniques to give a clearer view of how far the cancer has spread. The goal of this research is to show why whole-body MRI could become a key tool in managing metastatic prostate cancer, helping doctors make better treatment decisions while reducing risks for patients.

**Assessment Category**	**Score**	**Assessment Category**	**Score**	**Assessment Category**	**Score**
Comprehension Score Rater 1	5	Gunning Fog Score	14.3	Words	152
Comprehension Score Rater 2	5	SMOG Index	9.5	Complex words	16
Reading Age (years)	18.5	Coleman Liau Index	13.2	Percentage of complex words	10.53
Flesch-Kincaid Reading Ease	48.1	Automated Readability Index	14.4	Average words per sentence	25.33
Flesch-Kincaid Grade Level	12.8	Sentences	6	Average syllables per word	1.57

Manuscript 97

von Eyben FE, Virgolini I, Baum R. Review on the Increasing Role for PSMA-Based Radioligand Therapy in Prostate Cancer. Cancers [Internet]. 2024 Jul 12;16(14):2520. Available from: http://dx.doi.org/10.3390/cancers16142520

Abstract

In 2021, two randomized controlled trials (RCTs), TheraP and VISION, demonstrated that 177Lu-PSMA-617 as monotherapy was more effective for the decline of PSA than the comparator third-line treatments.

Methods: Our review summarizes new RCTs that add to the use of radioligand therapy (RLT) for patients with high-risk prostate cancer (PCa).

Results: Four past and present RCTs included 1081 patients. An RCT, ENZA-p, studied first-line treatment of patients with metastatic castration-resistant PCa (mCRPC). A combination of enzalutamide (ENZA) and 177Lu-PSMA-617 gave longer progression-free survival than ENZA as monotherapy. Other RCTs of patients with mCRPC, including the PSMAfore, and SPLASH trials, showed 177Lu-PSMA-617 as second-line treatment gave better progression-free survival than androgen receptor pathway inhibitors (combined p value < 6.9 × 10-6).

Conclusions: Patients with PCa gain if they are given PSMA-RLT early in the treatment of PCa and as part of combination therapies.

Keywords:

combination therapy, nuclear medicine, prostate neoplasm, prostate-specific membrane antigen, randomized controlled trials

<u>Original Lay Summary</u>

In 2021, the randomized controlled trial VISION showed that prostate-specific membrane antigen (PSMA)-based third-line radioligand therapy as monotherapy for patients with metastatic castration-resistant prostate cancer with positive lesions on PSMA PET/CT increases overall survival relative to the comparator treatment. The good results have inspired new trials. Our review provides a comprehensive update on post-VISION trials on RLT. The results are promising and may expand the role of PSMA-based RLT in patients with prostate cancer

**Assessment Category**	**Score**	**Assessment Category**	**Score**	**Assessment Category**	**Score**
Comprehension Score Rater 1	4	Gunning Fog Score	16.0	Words	80
Comprehension Score Rater 2	4	SMOG Index	11.6	Complex words	16
Reading Age (years)	20.5	Coleman Liau Index	17.2	Percentage of complex words	20.0
Flesch-Kincaid Reading Ease	36.4	Automated Readability Index	14.9	Average words per sentence	20.0
Flesch-Kincaid Grade Level	13.2	Sentences	4	Average syllables per word	1.78

Lay Summary (Simple Prompt)

Prostate cancer is one of the most common cancers in men, and finding effective treatments is crucial. This review looks at new studies on a promising therapy called PSMA-based radioligand therapy, which uses a radioactive substance to target and kill cancer cells more precisely. The review focuses on clinical trials involving over 1,000 patients with advanced prostate cancer. These studies show that using this therapy earlier in treatment and in combination with other drugs, like enzalutamide, helps slow down cancer progression more effectively than traditional treatments. The findings suggest that PSMA-based radioligand therapy can improve outcomes for men with prostate cancer, offering hope for longer survival and better quality of life.

**Assessment Category**	**Score**	**Assessment Category**	**Score**	**Assessment Category**	**Score**
Comprehension Score Rater 1	4	Gunning Fog Score	16.5	Words	114
Comprehension Score Rater 2	5	SMOG Index	11.9	Complex words	21
Reading Age (years)	20.5	Coleman Liau Index	16.4	Percentage of complex words	18.42
Flesch-Kincaid Reading Ease	36.0	Automated Readability Index	15.7	Average words per sentence	22.8
Flesch-Kincaid Grade Level	13.9	Sentences	5	Average syllables per word	1.75

<u>Lay Summary (Extended Prompt)</u>

Prostate cancer is a common type of cancer in men, and finding better treatments is very important. This review looks at new research on a treatment called PSMA-based radioligand therapy (PSMA-RLT), which uses a special radioactive drug to target prostate cancer cells. Studies have shown that this treatment helps slow down cancer growth more effectively than some older treatments, especially when given earlier in the disease or combined with other medicines. The review includes results from several large clinical trials with over 1,000 patients. These studies found that PSMA-RLT helped patients live longer without their cancer getting worse. This means that using PSMA-RLT sooner and in combination with other treatments could improve outcomes for many men with prostate cancer. Understanding these results can help doctors choose the best treatments to improve patients' quality of life and survival.

**Assessment Category**	**Score**	**Assessment Category**	**Score**	**Assessment Category**	**Score**
Comprehension Score Rater 1	4	Gunning Fog Score	11.1	Words	143
Comprehension Score Rater 2	5	SMOG Index	7.8	Complex words	14
Reading Age (years)	16.5	Coleman Liau Index	15.1	Percentage of complex words	9.79
Flesch-Kincaid Reading Ease	53.8	Automated Readability Index	12.2	Average words per sentence	17.88
Flesch-Kincaid Grade Level	10.2	Sentences	8	Average syllables per word	1.59

Manuscript 98

Zhou Y, Zeng F, Richards GO, Wang N. ENO2, a Glycolytic Enzyme, Contributes to Prostate Cancer Metastasis: A Systematic Review of Literature. Cancers [Internet]. 2024 Jul 10;16(14):2503. Available from: http://dx.doi.org/10.3390/cancers16142503

Abstract

Prostate cancer (PCa) is the second leading cause of male cancer deaths in the UK and the fifth worldwide. The presence of distant PCa metastasis can reduce the 5-year survival rate from 100% to approximately 30%. Enolase 2 (ENO2), a crucial glycolytic enzyme in cancer metabolism, is associated with the metastasis of multiple cancers and is also used as a marker for neuroendocrine tumours. However, its role in PCa metastasis remains unclear. In this study, we systematically reviewed the current literature to determine the association between ENO2 and metastatic PCa. Medline, Web of Science, and PubMed were searched for eligible studies. The search yielded five studies assessing ENO2 expression in PCa patients or cell lines. The three human studies suggested that ENO2 expression is correlated with late-stage, aggressive PCa, including castrate-resistant PCa (CRPC), metastatic CRPC, and neuroendocrine PCa (NEPC). This was further supported by two in vitro studies indicating that ENO2 expression can be regulated by the tumour microenvironment, such as androgen deprived conditions and the presence of bone-forming osteoblasts. Therefore, ENO2 may functionally contribute to PCa metastasis, possibly due to the unique metabolic features of PCa, which are glycolysis dependent only at the advanced metastatic stage.

Keywords:

ENO2, androgen, glycolysis, metastasis, neuroendocrine prostate cancer, prostate cancer

Original Lay Summary

This paper reviews the role of ENO2, a protein involved in sugar metabolism, in advanced prostate cancer. Analysing five studies, we found that ENO2 levels tend to be higher in aggressive forms of prostate cancer, particularly those that have spread or become resistant to hormone therapy. This increased presence might be linked to how prostate cancer cells change their energy production as the disease progresses, shifting to rely more on sugar breakdown in advanced stages. The study also suggests that ENO2 can be influenced by the tumour's environment, such as low hormone levels or the presence of bone cells, which is relevant, as prostate cancer often spreads to bones. While not proving a direct causal relationship, the research indicates that ENO2 could be an important marker for aggressive disease and potentially a target for future treatments, warranting further investigation into its role in prostate cancer progression, especially in bone metastasis.

**Assessment Category**	**Score**	**Assessment Category**	**Score**	**Assessment Category**	**Score**
Comprehension Score Rater 1	5	Gunning Fog Score	17.6	Words	150
Comprehension Score Rater 2	5	SMOG Index	11.9	Complex words	21
Reading Age (years)	21.5	Coleman Liau Index	15.6	Percentage of complex words	14.0
Flesch-Kincaid Reading Ease	33.7	Automated Readability Index	18.7	Average words per sentence	30.0
Flesch-Kincaid Grade Level	16.0	Sentences	5	Average syllables per word	1.69

Lay Summary (Simple Prompt)

Prostate cancer is one of the leading causes of cancer-related deaths in men, especially when it spreads to other parts of the body (metastasis), which greatly lowers survival rates. A protein called ENO2, involved in how cells produce energy, has been linked to the spread of several types of cancer, but its role in prostate cancer is not well understood. In this study, we reviewed existing research to explore the connection between ENO2 and advanced prostate cancer. We analyzed five studies, including research on patients and laboratory-grown cancer cells. The findings suggest that higher levels of ENO2 are associated with more aggressive forms of prostate cancer, particularly those that are resistant to hormone treatments and have spread to other organs. This suggests that ENO2 might play an important role in helping prostate cancer grow and spread, which could lead to new ways to diagnose or treat advanced prostate cancer in the future.

**Assessment Category**	**Score**	**Assessment Category**	**Score**	**Assessment Category**	**Score**
Comprehension Score Rater 1	5	Gunning Fog Score	14.9	Words	154
Comprehension Score Rater 2	5	SMOG Index	10.1	Complex words	18
Reading Age (years)	18.5	Coleman Liau Index	14.0	Percentage of complex words	11.69
Flesch-Kincaid Reading Ease	47.8	Automated Readability Index	15.2	Average words per sentence	25.67
Flesch-Kincaid Grade Level	13.0	Sentences	6	Average syllables per word	1.57

Lay Summary (Extended Prompt)

Prostate cancer is one of the leading causes of cancer-related deaths in men, especially when it spreads to other parts of the body, which greatly lowers survival rates. This study looks at a protein called ENO2, which helps cancer cells produce energy and has been linked to the spread of other types of cancer. However, its role in prostate cancer spread was not clear. To find out more, we reviewed five scientific studies that examined ENO2 in prostate cancer patients and lab-grown cancer cells. The results showed that ENO2 is more active in aggressive, advanced forms of prostate cancer, especially in cases that no longer respond to treatment. Changes in the tumor environment, like low hormone levels or bone involvement, seem to affect ENO2 levels. Understanding how ENO2 works in prostate cancer could help researchers develop new ways to detect or treat cancer that has spread.

**Assessment Category**	**Score**	**Assessment Category**	**Score**	**Assessment Category**	**Score**
Comprehension Score Rater 1	5	Gunning Fog Score	12.2	Words	148
Comprehension Score Rater 2	5	SMOG Index	8.3	Complex words	14
Reading Age (years)	16.5	Coleman Liau Index	12.9	Percentage of complex words	9.46
Flesch-Kincaid Reading Ease	58.5	Automated Readability Index	12.1	Average words per sentence	21.14
Flesch-Kincaid Grade Level	10.4	Sentences	7	Average syllables per word	1.5

Manuscript 99

Foley GR, Marthick JR, Lucas SE, Raspin K, Banks A, Stanford JL, et al. Germline Sequencing of DNA Damage Repair Genes in Two Hereditary Prostate Cancer Cohorts Reveals New Disease Risk-Associated Gene Variants. Cancers [Internet]. 2024 Jul 7;16(13):2482. Available from: http://dx.doi.org/10.3390/cancers16132482

Abstract

Rare, inherited variants in DNA damage repair (DDR) genes have a recognised role in prostate cancer (PrCa) susceptibility. In addition, these genes are therapeutically targetable. While rare variants are informing clinical management in other common cancers, defining the rare disease-associated variants in PrCa has been challenging. Here, whole-genome and -exome sequencing data from two independent, high-risk Australian and North American familial PrCa datasets were interrogated for novel DDR risk variants. Rare DDR gene variants (predicted to be damaging and present in two or more family members) were identified and subsequently genotyped in 1963 individuals (700 familial and 459 sporadic PrCa cases, 482 unaffected relatives, and 322 screened controls), and association analyses accounting for relatedness (MQLS) undertaken. In the combined datasets, rare ERCC3 (rs145201970, p = 2.57 × 10-4) and BRIP1 (rs4988345, p = 0.025) variants were significantly associated with PrCa risk. A PARP2 (rs200603922, p = 0.028) variant in the Australian dataset and a MUTYH (rs36053993, p = 0.031) variant in the North American dataset were also associated with risk. Evaluation of clinicopathological characteristics provided no evidence for a younger age or higher-grade disease at diagnosis in variant carriers, which should be taken into consideration when determining genetic screening eligibility criteria for targeted, gene-based treatments in the future. This study adds valuable knowledge to our understanding of PrCa-associated DDR genes, which will underpin effective clinical screening and treatment strategies.

Keywords:

DNA repair, genetic susceptibility, germline variants, prostate cancer, rare high-risk genes

<u>Original Lay Summary</u>

An urgent demand exists to identify inherited genetic risk variants for prostate cancer (PrCa), particularly in DNA damage repair genes targetable with precision medicine-based strategies. Though the most heritable common cancer, discovery of rare germline PrCa risk variants is hampered by their low frequency, even in sizeable population datasets. Through utilising two large, independent, familial PrCa resources and their likely enrichment of rare causative variants, we provide robust evidence for several novel risk variants in DNA damage repair genes.

**Assessment Category**	**Score**	**Assessment Category**	**Score**	**Assessment Category**	**Score**
Comprehension Score Rater 1	3	Gunning Fog Score	23.2	Words	80
Comprehension Score Rater 2	3	SMOG Index	16.6	Complex words	25
Reading Age (years)	24.0	Coleman Liau Index	18.6	Percentage of complex words	31.25
Flesch-Kincaid Reading Ease	12.7	Automated Readability Index	10.5	Average words per sentence	26.67
Flesch-Kincaid Grade Level	18.1	Sentences	3	Average syllables per word	1.98

Lay Summary (Simple Prompt)

Prostate cancer can run in families, partly due to inherited changes in certain genes that help repair damaged DNA. These genes are important because they not only influence cancer risk but can also guide treatment options. In this study, researchers analyzed genetic data from two groups of men with a family history of prostate cancer in Australia and North America. They searched for rare genetic changes that might increase the risk of developing the disease. By comparing the genes of men with and without prostate cancer, they identified new variations in four specific genes—ERCC3, BRIP1, PARP2, and MUTYH—that are linked to a higher risk of prostate cancer. Interestingly, men with these gene changes did not develop cancer at a younger age or have more aggressive disease. These findings could help improve genetic screening and support the development of personalized treatments for prostate cancer in the future.

**Assessment Category**	**Score**	**Assessment Category**	**Score**	**Assessment Category**	**Score**
Comprehension Score Rater 1	5	Gunning Fog Score	14.1	Words	146
Comprehension Score Rater 2	5	SMOG Index	10.7	Complex words	24
Reading Age (years)	18.5	Coleman Liau Index	14.8	Percentage of complex words	16.44
Flesch-Kincaid Reading Ease	47.8	Automated Readability Index	13.5	Average words per sentence	20.86
Flesch-Kincaid Grade Level	11.8	Sentences	7	Average syllables per word	1.63

Lay Summary (Extended Prompt)

Prostate cancer can run in families, partly because of changes in certain genes that help repair damaged DNA. These gene changes, called variants, can increase the risk of developing cancer. In this study, researchers looked for new gene variants linked to prostate cancer risk by studying the DNA of families from Australia and North America with a history of the disease. They used advanced genetic tests to find rare gene changes that might cause prostate cancer. The study found several new gene variants that are linked to a higher risk of prostate cancer. Interestingly, having these variants did not mean the cancer would be more aggressive or appear at a younger age. These findings can help doctors better understand who is at risk and may lead to improved genetic screening and personalized treatments for prostate cancer in the future.

**Assessment Category**	**Score**	**Assessment Category**	**Score**	**Assessment Category**	**Score**
Comprehension Score Rater 1	4	Gunning Fog Score	12.8	Words	139
Comprehension Score Rater 2	5	SMOG Index	9.6	Complex words	19
Reading Age (years)	17.5	Coleman Liau Index	13.7	Percentage of complex words	13.67
Flesch-Kincaid Reading Ease	58.3	Automated Readability Index	12.1	Average words per sentence	19.86
Flesch-Kincaid Grade Level	10.1	Sentences	7	Average syllables per word	1.52

Manuscript 100

Reitano G, Ceccato T, Botti S, Bruniera M, Carrozza S, Bovolenta E, et al. Treatment and Staging Intensification Strategies Associated with Radical Prostatectomy for High-Risk Prostate Cancer: Efficacy Evaluation and Exploration of Novel Approaches. Cancers [Internet]. 2024 Jul 5;16(13):2465. Available from: http://dx.doi.org/10.3390/cancers16132465

Abstract

The management of high-risk prostate cancer (PCa) presents a significant clinical challenge, often necessitating treatment intensification due to the potential presence of micrometastases. While radical prostatectomy (RP) constitutes one of the primary treatment modalities, the integration of neoadjuvant and adjuvant therapies suggests a paradigm shift towards more aggressive treatment strategies, also guided by new imaging modalities like positron emission tomography using prostate-specific membrane antigen (PSMA-PET). Despite the benefits, treatment intensification raises concerns regarding increased side effects. This review synthesizes the latest evidence on perioperative treatment intensification and de-escalation for high-risk localized and locally advanced PCa patients eligible for surgery. Through a non-systematic literature review conducted via PubMed, Scopus, Web of Science, and ClinicalTrials.gov, we explored various dimensions of perioperative treatments, including neoadjuvant systemic therapies, adjuvant therapies, and the role of novel diagnostic technologies. Emerging evidence provides more support for neoadjuvant systemic therapies. Preliminary results from studies suggest the potential for treatments traditionally reserved for metastatic PCa to show apparent benefit in a non-metastatic setting. The role of adjuvant treatments remains debated, particularly the use of androgen deprivation therapy (ADT) and adjuvant radiotherapy in patients at higher risk of biochemical recurrence. The potential role of radio-guided PSMA lymph node dissection emerges as a cutting-edge approach, offering a targeted method for eradicating disease with greater

precision. Innovations such as artificial intelligence and machine learning are potential game-changers, offering new avenues for personal-
ized treatment and improved prognostication. The intensification of surgical treatment in high-risk PCa patients is a dynamic and evolving field, underscored by the integration of traditional and novel therapeutic approaches. As evidence continues to emerge, these strategies will refine patient selection, enhance treatment efficacy, and mitigate the risk of progression, although with an attentive consideration of the associated side effects.

Keywords:

PSMA-PET, adjuvant therapy, high-risk prostate cancer, lymphadenectomy, neoadjuvant therapy, prostatectomy

Original Lay Summary

High-risk prostate cancer is an aggressive disease, and its treatment can be complex and require the involvement of several specialists. Advances in imaging and therapies in this field can improve survival and help physicians choose the best personalized approach that maintains quality of life. This article summarizes the most recent publications on this condition and its treatments, aiming to provide an updated guide for managing patients with prostate cancer who experience a higher risk of progression and death.

**Assessment Category**	**Score**	**Assessment Category**	**Score**	**Assessment Category**	**Score**
Comprehension Score Rater 1	4	Gunning Fog Score	19.6	Words	79
Comprehension Score Rater 2	4	SMOG Index	14.1	Complex words	18
Reading Age (years)	22.5	Coleman Liau Index	16.7	Percentage of complex words	22.78
Flesch-Kincaid Reading Ease	33.4	Automated Readability Index	17.7	Average words per sentence	26.33
Flesch-Kincaid Grade Level	15.1	Sentences	3	Average syllables per word	1.73

Lay Summary (Simple Prompt)

This study reviews ways to improve treatment for men with aggressive prostate cancer. The research examines whether adding treatments before and after prostate removal surgery, along with using advanced imaging tests to better detect cancer spread, can lead to better outcomes. By reviewing various studies, the authors found that giving treatment before surgery may help reduce the chance of the cancer returning, although it might also increase side effects. Traditional follow-up treatments still have some uncertainty about their benefits, while new methods—such as targeted removal of affected lymph nodes and computer-based analysis—offer promising steps toward more personalized care. Overall, these insights could help doctors choose the best treatment plans and ultimately improve both the health and quality of life for patients with high-risk prostate cancer.

**Assessment Category**	**Score**	**Assessment Category**	**Score**	**Assessment Category**	**Score**
Comprehension Score Rater 1	5	Gunning Fog Score	17.4	Words	128
Comprehension Score Rater 2	5	SMOG Index	12.4	Complex words	23
Reading Age (years)	21.5	Coleman Liau Index	17.8	Percentage of complex words	17.97
Flesch-Kincaid Reading Ease	35.4	Automated Readability Index	18.2	Average words per sentence	25.6
Flesch-Kincaid Grade Level	14.7	Sentences	5	Average syllables per word	1.72

Lay Summary (Extended Prompt)

This study looks at new ways to treat men with high-risk prostate cancer. The authors reviewed many studies to see if giving treatment before or after surgery can help control the cancer. They also looked at new scanning tests that can find hidden signs of the disease. Early findings show that treatment before surgery might lower the chance of the cancer coming back. Some treatments given after surgery could also help, but more research is needed to be sure. New tools, like advanced scans and smart computer programs, may help doctors choose the best treatment for each patient. Overall, this work may lead to better care and improved health for people with high-risk prostate cancer.

**Assessment Category**	**Score**	**Assessment Category**	**Score**	**Assessment Category**	**Score**
Comprehension Score Rater 1	5	Gunning Fog Score	8.4	Words	117
Comprehension Score Rater 2	5	SMOG Index	5.2	Complex words	5
Reading Age (years)	14.5	Coleman Liau Index	12.5	Percentage of complex words	4.27
Flesch-Kincaid Reading Ease	72.0	Automated Readability Index	9.6	Average words per sentence	16.71
Flesch-Kincaid Grade Level	7.4	Sentences	7	Average syllables per word	1.39

Manuscript 101

Erdmann K, Distler F, Gräfe S, Kwe J, Erb HHH, Fuessel S, et al. Transcript Markers from Urinary Extracellular Vesicles for Predicting Risk Reclassification of Prostate Cancer Patients on Active Surveillance. Cancers [Internet]. 2024 Jul 4;16(13):2453. Available from: http://dx.doi.org/10.3390/cancers16132453

Abstract

Serum prostate-specific antigen (PSA), its derivatives, and magnetic resonance tomography (MRI) lack sufficient specificity and sensitivity for the prediction of risk reclassification of prostate cancer (PCa) patients on active surveillance (AS). We investigated selected transcripts in urinary extracellular vesicles (uEV) from PCa patients on AS to predict PCa risk reclassification (defined by ISUP 1 with PSA > 10 ng/mL or ISUP 2-5 with any PSA level) in control biopsy. Before the control biopsy, urine samples were prospectively collected from 72 patients, of whom 43% were reclassified during AS. Following RNA isolation from uEV, multiplexed reverse transcription, and pre-amplification, 29 PCa-associated transcripts were quantified by quantitative PCR. The predictive ability of the transcripts to indicate PCa risk reclassification was assessed by receiver operating characteristic (ROC) curve analyses via calculation of the area under the curve (AUC) and was then compared to clinical parameters followed by multivariate regression analysis. ROC curve analyses revealed a predictive potential for AMACR, HPN, MALAT1, PCA3, and PCAT29 (AUC = 0.614-0.655, p p p < 0.001) with a sensitivity, specificity, and accuracy of 87%, 83%, and 85%, respectively, thus surpassing the predictive power of the individual markers. These findings highlight the potential of uEV transcripts in combination with clinical parameters as monitoring markers during the AS of PCa.

Keywords:

active surveillance, biomarker, liquid biopsy, monitoring, prediction, prostate cancer, quantitative PCR, risk reclassification, transcripts, urinary extracellular vesicles

<u>Original Lay Summary</u>

Active surveillance is the preferred treatment strategy for low-risk prostate cancer and includes regular monitoring by control biopsies, which bear the risk of various side effects. In case of risk reclassification, therapy is switched to radical treatment. Currently used clinical parameters only possess a limited capability to indicate risk reclassification. Molecular markers identified via liquid biopsies, such as urine, could facilitate the detection of aggressive disease since they provide a more global assessment of prostate cancer than tissue biopsies. Moreover, an improved predictability could reduce the number of control biopsies needed during active surveillance. In this study, we identified a set of molecular markers from the urine of men on active surveillance that could predict the outcome of control biopsies. The combination of these molecular markers with clinical parameters resulted in further improved predictability of risk reclassification and, thus, has the potential to refine the monitoring strategies in active surveillance.

**Assessment Category**	**Score**	**Assessment Category**	**Score**	**Assessment Category**	**Score**
Comprehension Score Rater 1	5	Gunning Fog Score	20.0	Words	151
Comprehension Score Rater 2	5	SMOG Index	14.3	Complex words	43
Reading Age (years)	22.5	Coleman Liau Index	19.1	Percentage of complex words	28.48
Flesch-Kincaid Reading Ease	14.6	Automated Readability Index	17.3	Average words per sentence	21.57
Flesch-Kincaid Grade Level	16.6	Sentences	7	Average syllables per word	2.01

<u>Lay Summary (Simple Prompt)</u>

This research addresses the need for better monitoring of prostate cancer patients who are under observation rather than immediate treatment. Instead of relying solely on blood tests and imaging scans, which sometimes give unclear results, the study investigates genetic signals found in tiny particles present in urine. By analyzing these signals, the researchers aimed to predict more accurately when a patient's cancer might become more serious. Their findings suggest that using these urine markers alongside other clinical information could offer a more reliable method for tracking changes in the disease. This approach may not only improve patient care by helping doctors decide when to adjust treatment plans but also guide future research on developing more effective monitoring techniques.

**Assessment Category**	**Score**	**Assessment Category**	**Score**	**Assessment Category**	**Score**
Comprehension Score Rater 1	5	Gunning Fog Score	16.6	Words	118
Comprehension Score Rater 2	5	SMOG Index	11.9	Complex words	21
Reading Age (years)	21.5	Coleman Liau Index	17.8	Percentage of complex words	17.8
Flesch-Kincaid Reading Ease	28.7	Automated Readability Index	17.2	Average words per sentence	23.6
Flesch-Kincaid Grade Level	15.1	Sentences	5	Average syllables per word	1.82

<u>Lay Summary (Extended Prompt)</u>

This study looks at a new way to help doctors watch over men with prostate cancer who are not getting immediate treatment. Current tests sometimes miss when the cancer is changing. The researchers collected urine samples from patients and used a simple lab test to measure tiny pieces of genetic material. They found that a group of these genetic markers could better predict if a patient's cancer might become more serious. This approach could help doctors decide more quickly when to change a patient's treatment, making care safer and more effective.

**Assessment Category**	**Score**	**Assessment Category**	**Score**	**Assessment Category**	**Score**
Comprehension Score Rater 1	4	Gunning Fog Score	11.2	Words	91
Comprehension Score Rater 2	5	SMOG Index	7.9	Complex words	9
Reading Age (years)	16.5	Coleman Liau Index	13.6	Percentage of complex words	9.89
Flesch-Kincaid Reading Ease	57.3	Automated Readability Index	11.2	Average words per sentence	18.2
Flesch-Kincaid Grade Level	9.8	Sentences	5	Average syllables per word	1.55

Manuscript 102

Lishman-Walker E, Coffey K. Casein Kinase 1α—A Target for Prostate Cancer Therapy? Cancers [Internet]. 2024 Jul 2;16(13):2436. Available from: http://dx.doi.org/
10.3390/cancers16132436

Abstract

The androgen receptor (AR) is a key driver of prostate cancer (PCa) and, as such, current mainstay treatments target this molecule. However, resistance commonly arises to these therapies and, therefore, additional targets must be evaluated to improve patient outcomes. Consequently, alternative approaches for indirectly targeting the AR are sought. AR crosstalk with other signalling pathways, including several protein kinase signalling cascades, has been identified as a potential route to combat therapy resistance. The casein kinase 1 (CK1) family of protein kinases phosphorylate a multitude of substrates, allowing them to regulate a diverse range of pathways from the cell cycle to DNA damage repair. As well as its role in several signalling pathways that are de-regulated in PCa, mutational data suggest its potential to promote prostate carcinogenesis. CK1α is one isoform predicted to regulate AR activity via phosphorylation and has been implicated in the progression of several other cancer types. In this review, we explore how the normal biological function of CK1 is de-regulated in cancer, the impact on signalling pathways and how this contributes towards prostate tumourigenesis, with a particular focus on the CK1α isoform as a novel therapeutic target for PCa.

Keywords:

androgen receptor, casein kinase, cell signalling, prostate cancer

<u>Original Lay Summary</u>

Prostate cancer (PCa) is the most common cancer in males in the UK, resulting in more than 12,000 deaths per year in the UK. A large proportion of patients stop responding to currently available treatments; consequently, identifying alternative treatment options is key to prolonging the life of these men and improving survival rates. Protein kinases regulate signalling pathways, which are often dysregulated in cancer, leading to tumour growth. Additionally, kinase driven phosphorylation regulates androgen receptor activity, which is a key driver of PCa. Understanding how these signalling pathways contribute towards PCa is critical in the development of novel treatment options. The kinase, casein kinase 1 alpha (CK1α), has potential as a PCa therapeutic. Here we describe the current literature supporting this hypothesis and identify where further research is needed to fully understand the functional role and therapeutic potential of CK1α in PCa.

**Assessment Category**	**Score**	**Assessment Category**	**Score**	**Assessment Category**	**Score**
Comprehension Score Rater 1	5	Gunning Fog Score	18.0	Words	143
Comprehension Score Rater 2	5	SMOG Index	12.9	Complex words	35
Reading Age (years)	20.5	Coleman Liau Index	16.6	Percentage of complex words	24.48
Flesch-Kincaid Reading Ease	29.3	Automated Readability Index	14.7	Average words per sentence	20.43
Flesch-Kincaid Grade Level	14.2	Sentences	7	Average syllables per word	1.85

<u>Lay Summary (Simple Prompt)</u>

Prostate cancer often grows because of a protein called the androgen receptor, which current treatments target. However, many patients eventually stop responding to these therapies. This study looks at another protein, casein kinase 1 alpha, which might also control the growth of prostate cancer cells by affecting important cell processes like growth and DNA repair. By reviewing how this protein normally works and what goes wrong in cancer, the researchers hope to find new ways to fight the disease. Their work may lead to alternative treatments that can overcome resistance to current drugs, potentially offering better outcomes for patients and new insights for scientists studying prostate cancer.

**Assessment Category**	**Score**	**Assessment Category**	**Score**	**Assessment Category**	**Score**
Comprehension Score Rater 1	4	Gunning Fog Score	15.7	Words	107
Comprehension Score Rater 2	5	SMOG Index	11.3	Complex words	19
Reading Age (years)	19.5	Coleman Liau Index	16.5	Percentage of complex words	17.76
Flesch-Kincaid Reading Ease	42.8	Automated Readability Index	15.1	Average words per sentence	21.4
Flesch-Kincaid Grade Level	12.6	Sentences	5	Average syllables per word	1.68

Lay Summary (Extended Prompt)

Prostate cancer is often driven by a key molecule that helps the cancer grow. Many current treatments work by stopping this molecule, but over time, the cancer can learn to resist them. In this study, we review how a protein called casein kinase one alpha works in normal cells and what happens when it does not work properly. By looking at how this protein interacts with the key molecule, we hope to find new ways to fight prostate cancer when existing treatments fail. Our review shows that understanding this process could lead to more effective therapies and provide scientists with new ideas to tackle prostate cancer better.

**Assessment Category**	**Score**	**Assessment Category**	**Score**	**Assessment Category**	**Score**
Comprehension Score Rater 1	4	Gunning Fog Score	11.9	Words	107
Comprehension Score Rater 2	5	SMOG Index	7.9	Complex words	9
Reading Age (years)	16.5	Coleman Liau Index	12.6	Percentage of complex words	8.41
Flesch-Kincaid Reading Ease	58.6	Automated Readability Index	12.0	Average words per sentence	21.4
Flesch-Kincaid Grade Level	10.4	Sentences	5	Average syllables per word	1.5

Manuscript 103

Książek I, Ligęza A, Drzymała F, Borek A, Miszczyk M, Francuz MR, et al. Role of Lutetium Radioligand Therapy in Prostate Cancer. Cancers [Internet]. 2024 Jul 1;16(13):2433. Available from: http://dx.doi.org/10.3390/cancers16132433

Abstract

Theranostics utilize ligands that chelate radionuclides and selectively bind with cancer-specific membrane antigens. In the case of prostate cancer (PCa), the state-of-the-art lutetium-177-PSMA combines the radioactive β-emitter 177Lu with Vipivotide Tetraxetan, a prostate-specific membrane antigen (PSMA)-binding ligand. Several studies have been conducted, and the therapy is not without adverse effects (e.g., xerostomia, nausea, and fatigue); however, few events are reported as severe. The available evidence supports the use of 177Lu-PSMA in selected metastatic castration-resistant prostate cancer patients, and the treatment is considered a standard of care in several clinical scenarios. Emerging research shows promising results in the setting of hormone-sensitive prostate cancer; however, evidence from high-quality controlled trials is still missing. In this review, we discuss the available evidence for the application of 177Lu-PSMA in the management of PCa patients.

Keywords:

Lutetium-177-PSMA, prostate cancer, radiation therapy, radioactive isotopes

<u>Original Lay Summary</u>

Lutetium-177-PSMA—a radiopharmaceutical composed of a radionuclide lutetium-177 and a PSMA-binding ligand—is a theranostic medicine for prostate cancer. In this review, a summary of the impact of this drug on castration-resistant prostate cancer, hormone-sensitive prostate cancer, and localized prostate cancer is provided. Available data demonstrate that 177Lu-PSMA could be a successful therapy in those types of prostate cancers, but it is not without adverse effects. Conclusions revealed the need for further well-designed high-quality controlled trials to evaluate long-term outcomes and effectiveness.

**Assessment Category**	**Score**	**Assessment Category**	**Score**	**Assessment Category**	**Score**
Comprehension Score Rater 1	5	Gunning Fog Score	17.9	Words	90
Comprehension Score Rater 2	5	SMOG Index	13.2	Complex words	21
Reading Age (years)	21.5	Coleman Liau Index	17.0	Percentage of complex words	23.33
Flesch-Kincaid Reading Ease	25.1	Automated Readability Index	16.0	Average words per sentence	22.5
Flesch-Kincaid Grade Level	15.3	Sentences	4	Average syllables per word	1.88

Lay Summary (Simple Prompt)

This study explores a new treatment for prostate cancer that uses a special radioactive drug to target cancer cells. The drug attaches to a substance that naturally seeks out these cells and delivers a small, focused dose of radiation. The research finds that while some patients might experience mild side effects such as dry mouth, nausea, or fatigue, these are generally not serious. The treatment has shown promise for patients with advanced cancer and could potentially help those with less advanced forms as well. By improving our understanding of this approach, the study aims to guide doctors toward better care and may lead to more effective and safer therapies for prostate cancer in the future.

**Assessment Category**	**Score**	**Assessment Category**	**Score**	**Assessment Category**	**Score**
Comprehension Score Rater 1	5	Gunning Fog Score	13.7	Words	115
Comprehension Score Rater 2	5	SMOG Index	9.4	Complex words	13
Reading Age (years)	17.5	Coleman Liau Index	13.9	Percentage of complex words	11.3
Flesch-Kincaid Reading Ease	54.0	Automated Readability Index	13.9	Average words per sentence	23.0
Flesch-Kincaid Grade Level	11.4	Sentences	5	Average syllables per word	1.53

Lay Summary (Extended Prompt)

This study explores a new way to treat prostate cancer by using a tiny amount of radioactive medicine that seeks out and attacks cancer cells. The treatment works by attaching the radioactive substance to a molecule that finds the cancer, helping to deliver the radiation directly where it is needed. It has already shown promise in men with advanced prostate cancer and may help even more people in the future. While some patients may experience mild side effects such as dry mouth, nausea, or tiredness, these are generally not serious. The authors believe that with further research, this method could become an important tool for doctors and improve care for many patients fighting prostate cancer.

**Assessment Category**	**Score**	**Assessment Category**	**Score**	**Assessment Category**	**Score**
Comprehension Score Rater 1	5	Gunning Fog Score	14.1	Words	115
Comprehension Score Rater 2	5	SMOG Index	9.7	Complex words	14
Reading Age (years)	18.5	Coleman Liau Index	13.8	Percentage of complex words	12.17
Flesch-Kincaid Reading Ease	48.1	Automated Readability Index	13.7	Average words per sentence	23.0
Flesch-Kincaid Grade Level	12.3	Sentences	5	Average syllables per word	1.6

Manuscript 104

Jager A, Oddens JR, Postema AW, Miclea RL, Schoots IG, Nooijen PGTA, et al. Is There an Added Value of Quantitative DCE-MRI by Magnetic Resonance Dispersion Imaging for Prostate Cancer Diagnosis? Cancers [Internet]. 2024 Jul 1;16(13):2431. Available from: http://dx.doi.org/10.3390/cancers16132431

Abstract

In this multicenter, retrospective study, we evaluated the added value of magnetic resonance dispersion imaging (MRDI) to standard multiparametric MRI (mpMRI) for PCa detection. The study included 76 patients, including 51 with clinically significant prostate cancer (csPCa), who underwent radical prostatectomy and had an mpMRI including dynamic contrast-enhanced MRI. Two radiologists performed three separate randomized scorings based on mpMRI, MRDI and mpMRI+MRDI. Radical prostatectomy histopathology was used as the reference standard. Imaging and histopathology were both scored according to the Prostate Imaging-Reporting and Data System V2.0 sector map. Sensitivity and specificity for PCa detection were evaluated for mpMRI, MRDI and mpMRI+MRDI. Inter- and intra-observer variability for both radiologists was evaluated using Cohen's Kappa. On a per-patient level, sensitivity for csPCa for radiologist 1 (R1) for mpMRI, MRDI and mpMRI+MRDI was 0.94, 0.82 and 0.94, respectively. For the second radiologist (R2), these were 0.78, 0.94 and 0.96. R1 detected 4% additional csPCa cases using MRDI compared to mpMRI, and R2 detected 20% extra csPCa cases using MRDI. Inter-observer agreement was significant only for MRDI (Cohen's Kappa = 0.4250, p = 0.004). The results of this study show the potential of MRDI to improve inter-observer variability and the detection of csPCa.

Keywords:

dynamic constrast-enhanced MRI, multiparametric MRI, pharmacokinetic analysis, prostate cancer

<u>Original Lay Summary</u>

This multicenter, retrospective study assessed the added value of magnetic resonance dispersion imaging (MRDI), a quantitative analysis of dynamic contrast-enhanced MRI (DCE-MRI), alongside standard multiparametric MRI (mpMRI) for detecting clinically significant prostate cancer (csPCa). Seventy-six patients, including fifty-one with csPCa, who underwent mpMRI and radical prostatectomy, were included. Two radiologists evaluated mpMRI, MRDI and a combination of both, with histopathology serving as the reference standard. The study found that MRDI improved inter-observer agreement and enhanced csPCa detection when combined with mpMRI. MRDI enabled the detection of up to 20% more cases compared to mpMRI alone. With the role of DCE-MRI in the context of mpMRI being debated, this study suggests that quantitative analysis of DCE-MRI by MRDI could enhance csPCa detection and reduce variability between observers.

**Assessment Category**	**Score**	**Assessment Category**	**Score**	**Assessment Category**	**Score**
Comprehension Score Rater 1	5	Gunning Fog Score	19.4	Words	133
Comprehension Score Rater 2	5	SMOG Index	13.9	Complex words	35
Reading Age (years)	22.5	Coleman Liau Index	17.8	Percentage of complex words	26.32
Flesch-Kincaid Reading Ease	24.7	Automated Readability Index	16.6	Average words per sentence	22.17
Flesch-Kincaid Grade Level	15.3	Sentences	6	Average syllables per word	1.89

Lay Summary (Simple Prompt)

This study explored a new imaging approach to improve the detection of significant prostate cancer. Researchers compared the regular magnetic resonance imaging method with an additional technique that examines how contrast material moves through prostate tissue. They reviewed scans from 76 patients who later had surgery, with two doctors assessing the images using three different methods. The extra imaging technique helped one doctor identify 4% more cases of important cancer, while the other detected 20% more compared to the standard method alone. These results suggest that incorporating this extra step into regular scans could lead to more consistent and accurate diagnoses. Better detection can guide doctors in choosing the most effective treatments, ultimately benefiting patient care and advancing research in prostate cancer diagnosis.

**Assessment Category**	**Score**	**Assessment Category**	**Score**	**Assessment Category**	**Score**
Comprehension Score Rater 1	5	Gunning Fog Score	18.3	Words	123
Comprehension Score Rater 2	5	SMOG Index	13.1	Complex words	31
Reading Age (years)	21.5	Coleman Liau Index	18.3	Percentage of complex words	25.2
Flesch-Kincaid Reading Ease	27.8	Automated Readability Index	16.1	Average words per sentence	20.5
Flesch-Kincaid Grade Level	14.5	Sentences	6	Average syllables per word	1.87

Lay Summary (Extended Prompt)

Prostate cancer is a common disease that can be hard to diagnose correctly. In this study, researchers tested a new way of taking MRI pictures that shows how a special dye moves through the prostate. They compared this new method with the usual MRI scans by having two doctors review the images. The new technique helped the doctors find more important cases of prostate cancer and made it easier for them to agree on their findings. This suggests that the new imaging method could lead to quicker and more accurate diagnoses, which may improve treatment decisions and outcomes for patients.

**Assessment Category**	**Score**	**Assessment Category**	**Score**	**Assessment Category**	**Score**
Comprehension Score Rater 1	4	Gunning Fog Score	12.4	Words	100
Comprehension Score Rater 2	4	SMOG Index	8.7	Complex words	11
Reading Age (years)	16.5	Coleman Liau Index	12.8	Percentage of complex words	11.0
Flesch-Kincaid Reading Ease	60.5	Automated Readability Index	11.4	Average words per sentence	20.0
Flesch-Kincaid Grade Level	9.8	Sentences	5	Average syllables per word	1.49

Manuscript 105

MacDonald C, Ilie G, Kephart G, Rendon R, Mason R, Bailly G, et al. Mediating Effects of Self-Efficacy and Illness Perceptions on Mental Health in Men with Localized Prostate Cancer: A Secondary Analysis of the Prostate Cancer Patient Empowerment Program (PC-PEP) Randomized Controlled Trial. Cancers [Internet]. 2024 Jun 27;16(13):2352. Available from: http://dx.doi.org/10.3390/cancers16132352

Abstract

Understanding how interventions reduce psychological distress in patients with prostate cancer is crucial for improving patient care. This study examined the roles of self-efficacy, illness perceptions, and heart rhythm coherence in mediating the effects of the Prostate Cancer Patient Empowerment Program (PC-PEP) on psychological distress compared to standard care. In a randomized controlled trial, 128 patients were assigned to either the PC-PEP intervention or standard care. The PC-PEP, a six-month program emphasizing daily healthy living habits, included relaxation and stress management, diet, exercise, pelvic floor muscle exercises, and strategies to improve relationships and intimacy, with daily activities supported by online resources and live sessions. Participants in the intervention group showed significant improvements in self-efficacy and specific illness perceptions, such as personal control and emotional response, compared to the control group. These factors mediated the relationship between the intervention and its psychological benefits, with self-efficacy accounting for 52% of the reduction in psychological distress. No significant differences in heart rhythm coherence were observed. This study highlights the critical role of self-efficacy and illness perceptions in enhancing psychological health in prostate cancer patients through the PC-PEP. The results underscore this program's effectiveness and the key mechanisms through which it operates. Given the high rates of distress among men undergoing prostate cancer treatments, these findings emphasize the importance of integrating the PC-PEP into clinical practice. The implementation of the PC-PEP in clinical settings can provide a

structured approach to reducing psychological distress and improving overall patient well-being.

Keywords:

behavioural intervention, empowerment, illness perception, mental health, patient activation, patient education, prostate cancer, quality of life, self-efficacy, survivorship

<u>Original Lay Summary</u>

This study investigates how the Prostate Cancer Patient Empowerment Program (PC-PEP) helps men with prostate cancer by promoting patient empowerment and activation. This program encourages patients to engage actively in their care, fostering healthy living habits daily, over six months. Involving 128 patients, this research found that those participating in the PC-PEP experienced significant improvements in self-efficacy and emotional responses, leading to reduced psychological distress compared to those receiving standard care. These findings underscore the importance of integrating the PC-PEP into clinical practice to enhance mental health and patient care strategies.

**Assessment Category**	**Score**	**Assessment Category**	**Score**	**Assessment Category**	**Score**
Comprehension Score Rater 1	5	Gunning Fog Score	17.9	Words	95
Comprehension Score Rater 2	5	SMOG Index	13.2	Complex words	21
Reading Age (years)	22.5	Coleman Liau Index	19.6	Percentage of complex words	22.11
Flesch-Kincaid Reading Ease	24.2	Automated Readability Index	18.8	Average words per sentence	23.75
Flesch-Kincaid Grade Level	15.8	Sentences	4	Average syllables per word	1.87

Lay Summary (Simple Prompt)

Many men with prostate cancer often experience high stress, which can affect their overall health. This study looked at whether a six-month program designed to support healthy living—through activities like relaxation, exercise, healthy eating, and relationship building—can reduce this stress. The program aimed to boost patients' confidence in managing their illness and help them see their condition in a more positive light. The findings showed that men who participated in the program felt more capable and had a healthier view of their illness, which was linked to lower levels of stress. These results suggest that incorporating such supportive programs into routine care could improve mental well-being and quality of life for prostate cancer patients.

**Assessment Category**	**Score**	**Assessment Category**	**Score**	**Assessment Category**	**Score**
Comprehension Score Rater 1	5	Gunning Fog Score	15.5	Words	115
Comprehension Score Rater 2	5	SMOG Index	11.0	Complex words	18
Reading Age (years)	19.5	Coleman Liau Index	16.6	Percentage of complex words	15.65
Flesch-Kincaid Reading Ease	43.0	Automated Readability Index	16.0	Average words per sentence	23.0
Flesch-Kincaid Grade Level	13.0	Sentences	5	Average syllables per word	1.66

Lay Summary (Extended Prompt)

This study explored a special program designed to help men with prostate cancer feel less stressed and more in control of their lives. The program taught simple healthy habits, such as relaxation techniques, regular exercise, a balanced diet, and ways to improve personal relationships. Researchers compared men who took part in the program to those who received regular care. They found that the program boosted the men's confidence and changed how they viewed their illness, which led to lower stress levels. These results show that small changes in daily habits can make a big difference in mental health, suggesting that similar programs could become an important part of care for men with prostate cancer.

**Assessment Category**	**Score**	**Assessment Category**	**Score**	**Assessment Category**	**Score**
Comprehension Score Rater 1	5	Gunning Fog Score	13.7	Words	114
Comprehension Score Rater 2	5	SMOG Index	9.4	Complex words	13
Reading Age (years)	18.5	Coleman Liau Index	14.4	Percentage of complex words	11.4
Flesch-Kincaid Reading Ease	57.5	Automated Readability Index	14.1	Average words per sentence	22.8
Flesch-Kincaid Grade Level	10.9	Sentences	5	Average syllables per word	1.49

Manuscript 106

Gómez-Aparicio M, López-Campos F, Buchser D, Lazo A, Willisch P, Ocanto A, et al. Is There an Opportunity to De-Escalate Treatments in Selected Patients with Metastatic Hormone-Sensitive Prostate Cancer? Cancers [Internet]. 2024 Jun 26;16(13):2331. Available from: http://dx.doi.org/10.3390/cancers16132331

Abstract

The treatment landscape for metastatic hormone-sensitive prostate cancer continues to evolve, with systemic treatment being the mainstay of current treatment. Prognostic and predictive factors such as tumour volume and disease presentation have been studied to assess responses to different treatments. Intensification and de-escalation strategies arouse great interest, so several trials are being developed to further personalize the therapy in these populations. Is there an optimal sequence and a possible option to de-intensify treatment in selected patients with a favourable profile? This and other goals will be the subject of this review.

Keywords:

androgen receptor signalling inhibitors, de-intensification, metastatic hormone-sensitive prostate cancer, treatment intensification

<u>Original Lay Summary</u>

First-line treatment options for patients with hormone-sensitive metastatic prostate cancer (mHSPC) have evolved in recent years with treatment intensification strategies used to improve survival and delay disease progression. This study reviews the evolution of treatment intensification in these patients, as well as ongoing trials that will provide us with answers to different questions that we ask in routine clinical practice.

**Assessment Category**	**Score**	**Assessment Category**	**Score**	**Assessment Category**	**Score**
Comprehension Score Rater 1	4	Gunning Fog Score	19.5	Words	62
Comprehension Score Rater 2	4	SMOG Index	13.5	Complex words	11
Reading Age (years)	23.5	Coleman Liau Index	19.0	Percentage of complex words	17.74
Flesch-Kincaid Reading Ease	21.2	Automated Readability Index	21.9	Average words per sentence	31.0
Flesch-Kincaid Grade Level	18.0	Sentences	2	Average syllables per word	1.82

Lay Summary (Simple Prompt)

This study looks at whether some men with advanced prostate cancer might safely receive less intense treatment. Doctors usually use strong, broad treatments, but the research explores if patients with certain favorable health indicators could do just as well with a gentler approach. By reviewing current studies and trials, the authors aim to pinpoint factors that might predict a good response to lower treatment doses. If successful, this strategy could reduce side effects and improve patients' quality of life without sacrificing the effectiveness of the therapy. The findings could help guide doctors in choosing the best treatment options and encourage further research into personalized care for prostate cancer.

**Assessment Category**	**Score**	**Assessment Category**	**Score**	**Assessment Category**	**Score**
Comprehension Score Rater 1	5	Gunning Fog Score	13.5	Words	108
Comprehension Score Rater 2	5	SMOG Index	9.4	Complex words	13
Reading Age (years)	19.5	Coleman Liau Index	17.1	Percentage of complex words	12.04
Flesch-Kincaid Reading Ease	44.7	Automated Readability Index	15.7	Average words per sentence	21.6
Flesch-Kincaid Grade Level	12.4	Sentences	5	Average syllables per word	1.66

<u>Lay Summary (Extended Prompt)</u>

This study asks if some patients with advanced prostate cancer could be treated with gentler therapies. Doctors usually use strong treatments that can cause many side effects. The researchers are looking at simple factors like tumor size and how the disease starts to see if they can tell which patients might do well with less intense care. They hope to find a better way to match treatment strength with a patient's needs. This work could lead to more personalized care that is easier on patients while still fighting the cancer. It is important because it might help reduce harmful side effects and improve the quality of life for people with this disease.

**Assessment Category**	**Score**	**Assessment Category**	**Score**	**Assessment Category**	**Score**
Comprehension Score Rater 1	5	Gunning Fog Score	10.3	Words	112
Comprehension Score Rater 2	5	SMOG Index	6.8	Complex words	8
Reading Age (years)	15.5	Coleman Liau Index	12.6	Percentage of complex words	7.14
Flesch-Kincaid Reading Ease	69.3	Automated Readability Index	10.7	Average words per sentence	18.67
Flesch-Kincaid Grade Level	8.2	Sentences	6	Average syllables per word	1.4

Manuscript 107

Morote J, Paesano N, Picola N, Miró B, Abascal JM, Servian P, et al. Comparing Two Targeted Biopsy Schemes for Detecting Clinically Significant Prostate Cancer in Magnetic Resonance Index Lesions: Two- to Four-Core versus Saturated Transperineal Targeted Biopsy. Cancers [Internet]. 2024 Jun 23;16(13):2306. Available from: http://dx.doi.org/10.3390/cancers16132306

Abstract

Since the optimal scheme for targeted biopsies of magnetic resonance imaging (MRI) suspicious lesions remains unclear, we compare the efficacy of two schemes for these index lesions. A prospective trial was conducted in 1161 men with Prostate Imaging Reporting and Data System v 2.1 3-5 undergoing targeted and 12-core systematic biopsy in four centers between 2021 and 2023. Two- to four-core MRI-transrectal ultrasound fusion-targeted biopsies via the transperineal route were conducted in 900 men in three centers, while a mapping per 0.5 mm core method (saturated scheme) was employed in 261 men biopsied in another center. A propensity-matched 261 paired cases were selected for avoiding confounders other than the targeted biopsy scheme. CsPCa (grade group ≥ 2) was identified in 125 index lesions (41.1%) when the two- to four-core scheme was employed, while in 187 (71.9%) when the saturated biopsy (p p = 0.019). Rates of csPCa and iPCa remained similar in systematic biopsies. CsPCa detected only in systematic biopsies were 5 and 1.5%, respectively (p = 0.035) in each group. The saturated scheme for targeted biopsies detected more csPCa and less iPCa than did the two- to four-core scheme in the index lesions. The rate of csPCa detected only in the systematic biopsies decreased when the saturated scheme was employed.

Keywords:

index lesion, number of cores, prostate cancer, targeted biopsy

<u>Original Lay Summary</u>

Targeted biopsies of suspicious lesions detected in magnetic resonance imaging are crucial to discover clinically significant prostate cancer, especially those corresponding to index lesions. However, the optimal scheme for targeted biopsies remains unclear, despite the fact that a two- to four-core scheme is usually recommended. Saturated biopsies of the prostate gland have shown high efficiency in detecting significant PCa. In this article, we report a better efficacy for mapping using a 0.5 mm core biopsy scheme than that using the two- to four-core scheme for detecting clinically significant prostate cancer in magnetic resonance index lesions.

**Assessment Category**	**Score**	**Assessment Category**	**Score**	**Assessment Category**	**Score**
Comprehension Score Rater 1	5	Gunning Fog Score	20.1	Words	98
Comprehension Score Rater 2	5	SMOG Index	14.3	Complex words	31
Reading Age (years)	21.5	Coleman Liau Index	17.0	Percentage of complex words	31.63
Flesch-Kincaid Reading Ease	22.9	Automated Readability Index	14.6	Average words per sentence	19.6
Flesch-Kincaid Grade Level	14.9	Sentences	5	Average syllables per word	1.94

<u>Lay Summary (Simple Prompt)</u>

This study compared two different biopsy methods for finding prostate cancer in men with suspicious areas seen on MRI scans. One method used a few tissue samples from the area of concern, while the other collected many more samples along the same region. The findings showed that the method using more samples was better at detecting serious forms of prostate cancer and reduced the chance of finding less dangerous cases. This suggests that using a more thorough sampling approach could lead to more accurate diagnoses, helping doctors make better treatment decisions. In turn, these improvements in detection may lead to more effective care for patients and provide valuable insights for further research in prostate cancer diagnosis.

**Assessment Category**	**Score**	**Assessment Category**	**Score**	**Assessment Category**	**Score**
Comprehension Score Rater 1	5	Gunning Fog Score	14.5	Words	116
Comprehension Score Rater 2	5	SMOG Index	10.1	Complex words	15
Reading Age (years)	19.5	Coleman Liau Index	15.3	Percentage of complex words	12.93
Flesch-Kincaid Reading Ease	44.7	Automated Readability Index	15.1	Average words per sentence	23.2
Flesch-Kincaid Grade Level	12.8	Sentences	5	Average syllables per word	1.64

<u>Lay Summary (Extended Prompt)</u>

This study compared two simple methods for taking tissue samples from areas that look suspicious on MRI scans of the prostate. One method took a few samples from the area, while the other method took many small samples to cover the whole area more thoroughly. The goal was to see which method found more cases of serious prostate cancer and fewer cases of less harmful changes. The research involved over a thousand men and found that taking more small samples detected more serious cancer and reduced the chance of finding cancers that might not need treatment. These findings may help doctors choose the best method to find and treat important prostate cancers, leading to better care for patients.

**Assessment Category**	**Score**	**Assessment Category**	**Score**	**Assessment Category**	**Score**
Comprehension Score Rater 1	5	Gunning Fog Score	11.5	Words	118
Comprehension Score Rater 2	5	SMOG Index	6.5	Complex words	6
Reading Age (years)	16.5	Coleman Liau Index	12.9	Percentage of complex words	5.08
Flesch-Kincaid Reading Ease	60.3	Automated Readability Index	13.4	Average words per sentence	23.6
Flesch-Kincaid Grade Level	10.7	Sentences	5	Average syllables per word	1.45

Manuscript 108

Gerke MB, Jansen CS, Bilen MA. Circulating Tumor DNA in Genitourinary Cancers: Detection, Prognostics, and Therapeutic Implications. Cancers [Internet]. 2024 Jun 20;16(12):2280. Available from: http://dx.doi.org/10.3390/cancers16122280

Abstract

CtDNA is emerging as a non-invasive clinical detection method for several cancers, including genitourinary (GU) cancers such as prostate cancer, bladder cancer, and renal cell carcinoma (RCC). CtDNA assays have shown promise in early detection of GU cancers, providing prognostic information, assessing real-time treatment response, and detecting residual disease and relapse. The ease of obtaining a "liquid biopsy" from blood or urine in GU cancers enhances its potential to be used as a biomarker. Interrogating these "liquid biopsies" for ctDNA can then be used to detect common cancer mutations, novel genomic alterations, or epigenetic modifications. CtDNA has undergone investigation in numerous clinical trials, which could address clinical needs in GU cancers, for instance, earlier detection in RCC, therapeutic response prediction in castration-resistant prostate cancer, and monitoring for recurrence in bladder cancers. The utilization of liquid biopsy for ctDNA analysis provides a promising method of advancing precision medicine within the field of GU cancers.

Keywords:

bladder cancer, circulating tumor DNA, genitourinary cancers, liquid biopsy, prostate cancer, renal cell carcinoma, tumor biomarkers

<u>Original Lay Summary</u>

Circulating tumor DNA (ctDNA) is a non-invasive method of identifying and monitoring genitourinary cancers, including prostate, bladder, and renal cell carcinoma, via blood or urine samples. CtDNA introduces a potential method for cancer screening. If detected, ctDNA may reveal genetic alterations that have prognostic value. As treatment options for genitourinary cancers progress towards accounting for tumor genetic profiles, ctDNA may predict which patients have improved responses to therapeutic targets. CtDNA may play an important role in surveillance after tumor resection and may be used to reveal mechanisms of treatment resistance. The clinical utility of ctDNA has yet to be established. Significantly more research is needed to understand the utility ctDNA has in clinical practice.

**Assessment Category**	**Score**	**Assessment Category**	**Score**	**Assessment Category**	**Score**
Comprehension Score Rater 1	5	Gunning Fog Score	16.3	Words	115
Comprehension Score Rater 2	5	SMOG Index	11.8	Complex words	29
Reading Age (years)	20.5	Coleman Liau Index	18.3	Percentage of complex words	25.22
Flesch-Kincaid Reading Ease	26.8	Automated Readability Index	14.1	Average words per sentence	16.43
Flesch-Kincaid Grade Level	13.6	Sentences	7	Average syllables per word	1.93

<u>Lay Summary (Simple Prompt)</u>

This study explores a new way to detect and monitor cancers affecting the urinary and reproductive systems by examining tiny fragments of DNA shed by tumors. Researchers are using simple blood or urine tests to find these fragments, which can help identify cancers such as those of the prostate, bladder, and kidney at an early stage. The goal is to use this approach not only to spot cancer sooner but also to monitor how well treatments are working and to catch any return of the disease quickly. This method could lead to more personalized care, reduce the need for invasive procedures, and ultimately improve patient outcomes by making cancer management simpler and more effective.

**Assessment Category**	**Score**	**Assessment Category**	**Score**	**Assessment Category**	**Score**
Comprehension Score Rater 1	5	Gunning Fog Score	16.3	Words	114
Comprehension Score Rater 2	5	SMOG Index	10.8	Complex words	14
Reading Age (years)	19.5	Coleman Liau Index	13.3	Percentage of complex words	12.28
Flesch-Kincaid Reading Ease	41.4	Automated Readability Index	16.1	Average words per sentence	28.5
Flesch-Kincaid Grade Level	14.6	Sentences	4	Average syllables per word	1.61

Lay Summary (Extended Prompt)

This study looks at a new, simple test that finds small pieces of cancer DNA in blood and urine. The test may help doctors spot cancers in the bladder, kidney, and prostate much earlier than before. It works like a tiny search tool that finds clues of cancer, shows how well a treatment is working, and even warns if the cancer returns. The goal is to see if this method can be used safely in everyday care. If it works well, this test could change how we detect and treat these cancers, making it easier for patients to get the right care quickly.

**Assessment Category**	**Score**	**Assessment Category**	**Score**	**Assessment Category**	**Score**
Comprehension Score Rater 1	5	Gunning Fog Score	9.4	Words	103
Comprehension Score Rater 2	5	SMOG Index	4.8	Complex words	3
Reading Age (years)	13.5	Coleman Liau Index	9.0	Percentage of complex words	2.91
Flesch-Kincaid Reading Ease	75.9	Automated Readability Index	8.7	Average words per sentence	20.6
Flesch-Kincaid Grade Level	7.8	Sentences	5	Average syllables per word	1.3

Manuscript 109

George AK, Miocinovic R, Patel AR, Lomas DJ, Correa AF, Chen DYT, et al. A Description and Safety Overview of Irreversible Electroporation for Prostate Tissue Ablation in Intermediate-Risk Prostate Cancer Patients: Preliminary Results from the PRESERVE Trial. Cancers [Internet]. 2024 Jun 8;16(12):2178. Available from: http://dx.doi.org/10.3390/cancers16122178

Abstract

The PRESERVE study (NCT04972097) aims to evaluate the safety and effectiveness of the NanoKnife System to ablate prostate tissue in patients with intermediate-risk prostate cancer (PCa). The NanoKnife uses irreversible electroporation (IRE) to deliver high-voltage electrical pulses to change the permeability of cell membranes, leading to cell death. A total of 121 subjects with organ-confined PCa ≤ T2c, prostate-specific antigens (PSAs) ≤ 15 ng/mL, and a Gleason score of 3 + 4 or 4 + 3 underwent focal ablation of the index lesion. The primary endpoints included negative in-field biopsy and adverse event incidence, type, and severity through 12 months. At the time of analysis, the trial had completed accrual with preliminary follow-up available. Demographics, disease characteristics, procedural details, PSA responses, and adverse events (AEs) are presented. The median (IQR) age at screening was 67.0 (61.0-72.0) years and Gleason distribution 3 + 4 (80.2%) and 4 + 3 (19.8%). At 6 months, all patients with available data (n = 74) experienced a median (IQR) percent reduction in PSA of 67.6% (52.3-82.2%). Only ten subjects (8.3%) experienced a Grade 3 adverse event; five were procedure-related. No Grade ≥ 4 AEs were reported. This study supports prior findings that IRE prostate ablation with the NanoKnife System can be performed safely. Final results are required to fully assess oncological, functional, and safety outcomes.

Keywords:

ablation, clinical trial, focal therapy, irreversible electroporation, prostate cancer

<u>Original Lay Summary</u>

The PRESERVE study is the first, large, prospective, pivotal trial of irreversible electroporation using the NanoKnife System for the prostate in the United States. The data from this United States Food and Drug Administration Investigational Device Exemption study aim to evaluate the safety and effectiveness of irreversible electroporation with the NanoKnife System to ablate prostate tissue in patients with intermediate-risk prostate cancer.

**Assessment Category**	**Score**	**Assessment Category**	**Score**	**Assessment Category**	**Score**
Comprehension Score Rater 1	3	Gunning Fog Score	18.9	Words	63
Comprehension Score Rater 2	3	SMOG Index	16.8	Complex words	17
Reading Age (years)	24.0	Coleman Liau Index	19.6	Percentage of complex words	26.98
Flesch-Kincaid Reading Ease	-1.1	Automated Readability Index	22.7	Average words per sentence	31.5
Flesch-Kincaid Grade Level	21.2	Sentences	2	Average syllables per word	2.08

Lay Summary (Simple Prompt)

This study explores a new treatment option for men with prostate cancer that is confined to the prostate gland. Researchers are testing a device that uses short electrical pulses to destroy small areas of cancerous tissue, aiming to offer a treatment that is both effective and safe. In an early trial involving 121 patients, the treatment led to a significant drop in a blood marker used to track prostate cancer, and only a few patients experienced moderate side effects. These promising early results suggest that this focused approach may reduce complications compared to more traditional treatments. If further research confirms these findings, this method could offer a safer, targeted treatment option and provide valuable insights to guide future studies in the field.

**Assessment Category**	**Score**	**Assessment Category**	**Score**	**Assessment Category**	**Score**
Comprehension Score Rater 1	5	Gunning Fog Score	13.7	Words	122
Comprehension Score Rater 2	5	SMOG Index	9.0	Complex words	12
Reading Age (years)	18.5	Coleman Liau Index	15.2	Percentage of complex words	9.84
Flesch-Kincaid Reading Ease	44.8	Automated Readability Index	15.6	Average words per sentence	24.4
Flesch-Kincaid Grade Level	13.1	Sentences	5	Average syllables per word	1.62

<u>Lay Summary (Extended Prompt)</u>

This study looked at a new treatment for prostate cancer that uses a special device to send small electric pulses into the tumor. The goal is to kill cancer cells while keeping the healthy parts of the prostate safe. Over 100 men with early-stage prostate cancer were treated and then followed for a year to see how well the treatment worked and how safe it was. Early results show that many patients had a big drop in a key blood test level that helps track cancer, and only a few experienced moderate side effects. These findings may lead to a safer and less invasive treatment option for those fighting prostate cancer.

**Assessment Category**	**Score**	**Assessment Category**	**Score**	**Assessment Category**	**Score**
Comprehension Score Rater 1	5	Gunning Fog Score	10.0	Words	112
Comprehension Score Rater 2	5	SMOG Index	4.8	Complex words	3
Reading Age (years)	14.5	Coleman Liau Index	10.6	Percentage of complex words	2.68
Flesch-Kincaid Reading Ease	69.3	Automated Readability Index	10.9	Average words per sentence	22.4
Flesch-Kincaid Grade Level	9.2	Sentences	5	Average syllables per word	1.36

Manuscript 110

Ni Y, Shi M, Liu L, Lin D, Zeng H, Ong C, et al. G9a in Cancer: Mechanisms, Therapeutic Advancements, and Clinical Implications. Cancers [Internet]. 2024 Jun 8;16(12):2175. Available from: http://dx.doi.org/10.3390/cancers16122175

Abstract

G9a, also named EHMT2, is a histone 3 lysine 9 (H3K9) methyltransferase responsible for catalyzing H3K9 mono- and dimethylation (H3K9me1 and H3K9me2). G9a contributes to various aspects of embryonic development and tissue differentiation through epigenetic regulation. Furthermore, the aberrant expression of G9a is frequently observed in various tumors, particularly in prostate cancer, where it contributes to cancer pathogenesis and progression. This review highlights the critical role of G9a in multiple cancer-related processes, such as epigenetic dysregulation, tumor suppressor gene silencing, cancer lineage plasticity, hypoxia adaption, and cancer progression. Despite the increased research on G9a in prostate cancer, there are still significant gaps, particularly in understanding its interactions within the tumor microenvironment and its broader epigenetic effects. Furthermore, this review discusses the recent advancements in G9a inhibitors, including the development of dual-target inhibitors that target G9a along with other epigenetic factors such as EZH2 and HDAC. It aims to bring together the existing knowledge, identify gaps in the current research, and suggest future directions for research and treatment strategies.

Keywords:

EHMT2, G9a, epigenetic inhibitor, histone methyltransferase, prostate cancer

<u>Original Lay Summary</u>

In this review, we have summarized the crucial role of G9a in cellular biology and disease. By synthesizing recent research discoveries, we delve into the functions of G9a in gene expression regulation, cell proliferation, and biological development. Additionally, we explore its potential involvement in cancer and discuss its prospects as a therapeutic target specifically in prostate cancer. In summary, this review provides a comprehensive perspective on G9a, enhances understanding of the significance of G9a in biology and cancer, and offers insights into future research and drug development.

**Assessment Category**	**Score**	**Assessment Category**	**Score**	**Assessment Category**	**Score**
Comprehension Score Rater 1	5	Gunning Fog Score	18.8	Words	87
Comprehension Score Rater 2	5	SMOG Index	13.5	Complex words	22
Reading Age (years)	22.5	Coleman Liau Index	17.8	Percentage of complex words	25.29
Flesch-Kincaid Reading Ease	15.6	Automated Readability Index	16.4	Average words per sentence	21.75
Flesch-Kincaid Grade Level	16.5	Sentences	4	Average syllables per word	2.0

Lay Summary (Simple Prompt)

Cancer often develops when the normal control of gene activity is disrupted. In this study, researchers focus on a protein called G9a, which plays a crucial role in turning genes on and off during cell growth and development. They have found that abnormal levels of this protein can silence important genes that protect against tumors, especially in prostate cancer, and help cancer cells adapt to harsh conditions. By reviewing existing research and identifying what is still not understood about G9a's behavior within tumors, the study aims to pave the way for new research directions. It also highlights emerging treatments that target G9a and related pathways, which may lead to more effective cancer therapies and improved care for patients.

**Assessment Category**	**Score**	**Assessment Category**	**Score**	**Assessment Category**	**Score**
Comprehension Score Rater 1	5	Gunning Fog Score	15.9	Words	118
Comprehension Score Rater 2	5	SMOG Index	11.3	Complex words	19
Reading Age (years)	19.5	Coleman Liau Index	14.8	Percentage of complex words	16.1
Flesch-Kincaid Reading Ease	42.4	Automated Readability Index	14.9	Average words per sentence	23.6
Flesch-Kincaid Grade Level	13.2	Sentences	5	Average syllables per word	1.66

Lay Summary (Extended Prompt)

This study looks at a protein called G9a that helps control how our cells work. When G9a does not work properly, it can lead to cancer, especially in the prostate. The authors reviewed many research studies to understand how this protein changes cell behavior and helps cancer grow. They also explored new medicines that might stop G9a from causing harm. The goal is to fill gaps in our current knowledge and to find better ways to treat cancer. By learning more about G9a, scientists and doctors may develop new treatments that improve the care and lives of people affected by cancer.

**Assessment Category**	**Score**	**Assessment Category**	**Score**	**Assessment Category**	**Score**
Comprehension Score Rater 1	5	Gunning Fog Score	9.5	Words	101
Comprehension Score Rater 2	5	SMOG Index	6.4	Complex words	7
Reading Age (years)	14.5	Coleman Liau Index	11.7	Percentage of complex words	6.93
Flesch-Kincaid Reading Ease	70.8	Automated Readability Index	9.0	Average words per sentence	16.83
Flesch-Kincaid Grade Level	7.6	Sentences	6	Average syllables per word	1.41

Manuscript 111

Alanezi ST, Kraśny MJ, Kleefeld C, Colgan N. Differential Diagnosis of Prostate Cancer Grade to Augment Clinical Diagnosis Based on Classifier Models with Tuned Hyperparameters. Cancers [Internet]. 2024 Jun 6;16(11):2163. Available from: http://dx.doi.org/10.3390/cancers16112163

Abstract

We developed a novel machine-learning algorithm to augment the clinical diagnosis of prostate cancer utilizing first and second-order texture analysis metrics in a novel application of machine-learning radiomics analysis. We successfully discriminated between significant prostate cancers versus non-tumor regions and provided accurate prediction between Gleason score cohorts with statistical sensitivity of 0.82, 0.81 and 0.91 in three separate pathology classifications. Tumor heterogeneity and prediction of the Gleason score were quantified using two feature selection approaches and two separate classifiers with tuned hyperparameters. There was a total of 71 patients analyzed in this study. Multiparametric MRI, incorporating T2WI and ADC maps, were used to derive radiomics features. Recursive feature elimination (RFE), the least absolute shrinkage and selection operator (LASSO), and two classification approaches, incorporating a support vector machine (SVM) (with randomized search) and random forest (RF) (with grid search), were utilized to differentiate between non-tumor regions and significant cancer while also predicting the Gleason score. In T2WI images, the RFE feature selection approach combined with RF and SVM classifiers outperformed LASSO with SVM and RF classifiers. The best performance was achieved by combining LASSO and SVM into a model that used both T2WI and ADC images. This model had an area under the curve (AUC) of 0.91. Radiomic features computed from ADC and T2WI images were used to predict three groups of Gleason score using two kinds of feature selection methods (RFE and LASSO), RF and SVM classifier models with tuned hyperparameters. Using combined sequences (T2WI and ADC map images) and combined radiomics (1st and GLCM features), LASSO, with a feature selection method with RF, was able to predict G3

with the highest sensitivity at a level AUC of 0.92. To predict G3 for single sequence (T2WI images) using GLCM features, LASSO with SVM achieved the highest sensitivity with an AUC of 0.92.

Keywords:

machine learning, multiparametric (mp-MRI), prostate cancer

Original Lay Summary

Multiparametric MRI with radiomics features derived from T2WI and ADC maps distinguished non-tumor regions from significant cancer and predicted the Gleason score using support vector machine (SVM) and random forest (RF) classification methods with tuned hyperparameters, as well as recursive feature elimination (RFE) and the least absolute shrinkage and selection operator (LASSO) feature selection methods. Successful application of a novel approach to machine learning incorporating recursive feature elimination combined with random forest and support vector classifiers allowed stratification of Gleeson scores in clinical cohorts at a sensitivity greater than 0.91.

**Assessment Category**	**Score**	**Assessment Category**	**Score**	**Assessment Category**	**Score**
Comprehension Score Rater 1	5	Gunning Fog Score	21.4	Words	92
Comprehension Score Rater 2	5	SMOG Index	15.6	Complex words	22
Reading Age (years)	24.0	Coleman Liau Index	20.4	Percentage of complex words	23.91
Flesch-Kincaid Reading Ease	2.8	Automated Readability Index	22.9	Average words per sentence	30.67
Flesch-Kincaid Grade Level	20.5	Sentences	3	Average syllables per word	2.04

Lay Summary (Simple Prompt)

This study aims to improve prostate cancer diagnosis using advanced computer techniques that analyze detailed MRI scans. The researchers created a new computer model that looks for subtle patterns in the images to distinguish between cancerous and non-cancerous tissue and to predict the aggressiveness of the cancer. Tested on patient data, the method showed strong potential in accurately identifying significant cancer areas. This improved accuracy can help doctors make better treatment decisions more quickly. Ultimately, the findings offer a promising step toward more reliable, computer-assisted diagnoses, which could enhance patient care and support further research in medical imaging and cancer detection.

**Assessment Category**	**Score**	**Assessment Category**	**Score**	**Assessment Category**	**Score**
Comprehension Score Rater 1	5	Gunning Fog Score	17.9	Words	103
Comprehension Score Rater 2	5	SMOG Index	12.9	Complex words	25
Reading Age (years)	21.5	Coleman Liau Index	18.8	Percentage of complex words	24.27
Flesch-Kincaid Reading Ease	24.9	Automated Readability Index	16.5	Average words per sentence	20.6
Flesch-Kincaid Grade Level	14.9	Sentences	5	Average syllables per word	1.9

Lay Summary (Extended Prompt)

This study was done to help doctors find and treat prostate cancer more accurately. Researchers made a computer program that learns from MRI pictures of the prostate. The program looks for small changes in the tissue to tell the difference between healthy areas and areas with cancer. It also helps predict how serious the cancer is. The best results came from using two kinds of MRI images together. This new method could lead to quicker and more precise diagnoses, which may help doctors decide on the best treatment faster and improve care for patients.

**Assessment Category**	**Score**	**Assessment Category**	**Score**	**Assessment Category**	**Score**
Comprehension Score Rater 1	5	Gunning Fog Score	9.2	Words	94
Comprehension Score Rater 2	5	SMOG Index	6.4	Complex words	7
Reading Age (years)	14.5	Coleman Liau Index	12.8	Percentage of complex words	7.45
Flesch-Kincaid Reading Ease	68.5	Automated Readability Index	9.3	Average words per sentence	15.67
Flesch-Kincaid Grade Level	7.6	Sentences	6	Average syllables per word	1.45

Manuscript 112

Vialat M, Baabdaty E, Trousson A, Kocer A, Lobaccaro JMA, Baron S, et al. Cholesterol Dietary Intake and Tumor Cell Homeostasis Drive Early Epithelial Tumorigenesis: A Potential Modelization of Early Prostate Tumorigenesis. Cancers [Internet]. 2024 Jun 6;16(11):2153. Available from: http://dx.doi.org/10.3390/cancers16112153

Abstract

Epidemiological studies point to cholesterol as a possible key factor for both prostate cancer incidence and progression. It could represent a targetable metabolite as the most aggressive tumors also appear to be sensitive to therapies designed to decrease hypercholesterolemia, such as statins. However, it remains unknown whether and how cholesterol, through its dietary uptake and its metabolism, could be important for early tumorigenesis. Oncogene clonal induction in the Drosophila melanogaster accessory gland allows us to reproduce tumorigenesis from initiation to early progression, where tumor cells undergo basal extrusion to form extra-epithelial tumors. Here we show that these tumors accumulate lipids, and especially esterified cholesterol, as in human late carcinogenesis. Interestingly, a high-cholesterol diet has a limited effect on accessory gland tumorigenesis. On the contrary, cell-specific downregulation of cholesterol uptake, intracellular transport, or metabolic response impairs the formation of such tumors. Furthermore, in this context, a high-cholesterol diet suppresses this impairment. Interestingly, expression data from primary prostate cancer tissues indicate an early signature of redirection from cholesterol de novo synthesis to uptake. Taken together, these results reveal that during early tumorigenesis, tumor cells strongly increase their uptake and use of dietary cholesterol to specifically promote the step of basal extrusion. Hence, these results suggest the mechanism by which a reduction in dietary cholesterol could lower the risk and slow down the progression of prostate cancer.

Keywords:

basal extrusion, cholesterol, early tumorigenesis, prostate cancer

Original Lay Summary

Cholesterol and cholesterol derivatives accumulate in prostate cancer cells. Epidemiologic studies about diet and/or treatment with cholesterol-lowering drugs indicate that this metabolite plays a role in cancer progression, but they do not discriminate its possible role in cancer incidence. The goal of this study is so to determine whether cholesterol availability impacts tumor formation itself. Using a drosophila model specifically dedicated to the study of early epithelial tumorigenesis, we find that basal extrusion, a critical step of tumor formation, directly depends on cholesterol availability through dietary intake, and on cholesterol metabolization by the tumor cells. As we find that many genes related to cholesterol homeostasis and metabolism are ill-expressed in primary prostate cancer samples, this work indicates that cholesterol levels and metabolism could play a crucial role in the early phases of prostate cancer as well.

**Assessment Category**	**Score**	**Assessment Category**	**Score**	**Assessment Category**	**Score**
Comprehension Score Rater 1	5	Gunning Fog Score	21.8	Words	138
Comprehension Score Rater 2	5	SMOG Index	15.9	Complex words	38
Reading Age (years)	24.0	Coleman Liau Index	18.3	Percentage of complex words	27.54
Flesch-Kincaid Reading Ease	3.5	Automated Readability Index	19.6	Average words per sentence	27.6
Flesch-Kincaid Grade Level	19.6	Sentences	5	Average syllables per word	2.07

Lay Summary (Simple Prompt)

This study looks at how cholesterol in our diet might influence the early steps of prostate cancer. Using a simple fruit fly model to mimic early tumor development, the researchers found that cancer cells gather cholesterol from food, which helps them break away from their original location and start growing in new areas. When the cells' ability to take in cholesterol was reduced, tumor growth slowed down, but a diet high in cholesterol could reverse this effect. These results suggest that the cholesterol we eat may play an important role in the onset and spread of prostate cancer. By understanding this link, scientists hope to develop new strategies that could lower the risk or slow down the progression of the disease, offering potential benefits for both patients and the broader research community.

**Assessment Category**	**Score**	**Assessment Category**	**Score**	**Assessment Category**	**Score**
Comprehension Score Rater 1	5	Gunning Fog Score	16.3	Words	132
Comprehension Score Rater 2	5	SMOG Index	11.3	Complex words	19
Reading Age (years)	19.5	Coleman Liau Index	14.0	Percentage of complex words	14.39
Flesch-Kincaid Reading Ease	46.7	Automated Readability Index	15.6	Average words per sentence	26.4
Flesch-Kincaid Grade Level	13.3	Sentences	5	Average syllables per word	1.58

<u>Lay Summary (Extended Prompt)</u>

This study explores how the fat in our diet, especially cholesterol, may help start prostate cancer. Researchers used fruit flies to mimic the early stages of cancer development. They found that cells beginning to form tumors take in extra cholesterol from food. When the ability of these cells to take in cholesterol was reduced, fewer tumors formed. However, a diet high in cholesterol helped the cells grow into tumors even when their cholesterol uptake was lowered. These results suggest that eating less cholesterol could lower the risk or slow the start of prostate cancer. The findings are important because they offer a simple way to think about preventing or managing this common cancer.

**Assessment Category**	**Score**	**Assessment Category**	**Score**	**Assessment Category**	**Score**
Comprehension Score Rater 1	5	Gunning Fog Score	11.8	Words	113
Comprehension Score Rater 2	5	SMOG Index	8.6	Complex words	15
Reading Age (years)	16.5	Coleman Liau Index	14.0	Percentage of complex words	13.27
Flesch-Kincaid Reading Ease	57.2	Automated Readability Index	10.5	Average words per sentence	16.14
Flesch-Kincaid Grade Level	9.3	Sentences	7	Average syllables per word	1.58

Manuscript 113

Porcaro AB, Bianchi A, Gallina S, Panunzio A, Tafuri A, Serafin E, et al. Prognostic Impact and Clinical Implications of Adverse Tumor Grade in Very Favorable Low- and Intermediate-Risk Prostate Cancer Patients Treated with Robot-Assisted Radical Prostatectomy: Experience of a Single Tertiary Referral Center. Cancers [Internet]. 2024 Jun 4;16(11):2137. Available from: http://dx.doi.org/10.3390/cancers16112137

Abstract

Objectives: To assess the prognostic impact and predictors of adverse tumor grade in very favorable low- and intermediate-risk prostate cancer (PCa) patients treated with robot-assisted radical prostatectomy (RARP).

Methods: Data of low- and intermediate PCa risk-class patients were retrieved from a prospectively maintained institutional database. Adverse tumor grade was defined as pathology ISUP grade group > 2. Disease progression was defined as a biochemical recurrence event and/or local recurrence and/or distant metastases. Associations were assessed by Cox's proportional hazards and logistic regression model.

Results: Between January 2013 and October 2020, the study evaluated a population of 289 patients, including 178 low-risk cases (61.1%) and 111 intermediate-risk subjects (38.4%); unfavorable tumor grade was detected in 82 cases (28.4%). PCa progression, which occurred in 29 patients (10%), was independently predicted by adverse tumor grade and biopsy ISUP grade group 2, with the former showing stronger associations (hazard ratio, HR = 4.478; 95% CI: 1.840-10.895; p = 0.001) than the latter (HR = 2.336; 95% CI: 1.057-5.164; p = 0.036). Older age and biopsy ISUP grade group 2 were independent clinical predictors of adverse tumor grade, associated with larger tumors that eventually presented non-organ-confined disease.

Conclusions: In a very favorable PCa patient population, adverse tumor grade was an unfavorable prognostic factor for disease progression. Active surveillance in very favorable intermediate-risk patients is still a hazard, so molecular and genetic testing of biopsy specimens is needed.

Keywords:

adverse pathology outcomes, intermediate-risk prostate cancer, low-risk prostate cancer, progression of prostate cancer, prostate cancer, robot-assisted radical prostatectomy (RARP), tumor upgrading, tumor upstaging

Original Lay Summary

The prognosis of PCa at diagnosis is assessed by classification systems that aim to group homogenous sets of patients. However, great heterogeneity exists within these risk groups, in terms of prognostic factors and treatment options. The aim of this study was to assess the prognostic impact and predictors of adverse tumor grade in very favorable low- and intermediate-risk prostate cancer patients treated with robot-assisted radical prostatectomy at a high-volume tertiary referral center.

**Assessment Category**	**Score**	**Assessment Category**	**Score**	**Assessment Category**	**Score**
Comprehension Score Rater 1	3	Gunning Fog Score	18.5	Words	75
Comprehension Score Rater 2	4	SMOG Index	13.3	Complex words	16
Reading Age (years)	21.5	Coleman Liau Index	16.6	Percentage of complex words	21.33
Flesch-Kincaid Reading Ease	23.5	Automated Readability Index	17.0	Average words per sentence	25.0
Flesch-Kincaid Grade Level	16.2	Sentences	3	Average syllables per word	1.87

<u>Lay Summary (Simple Prompt)</u>

This study explored whether a higher tumor grade in men with seemingly low-risk prostate cancer could indicate a greater chance of the disease returning after surgery. The researchers analyzed patients who underwent robot-assisted prostate surgery and discovered that those with more aggressive tumor characteristics were more likely to experience cancer progression. They also found that older age and certain biopsy results could predict these higher tumor grades. The findings suggest that even patients initially considered to have favorable prostate cancer might benefit from additional testing and closer monitoring. This research can help doctors better tailor treatment plans and follow-up care, ultimately aiming to improve patient outcomes in the future.

**Assessment Category**	**Score**	**Assessment Category**	**Score**	**Assessment Category**	**Score**
Comprehension Score Rater 1	5	Gunning Fog Score	16.8	Words	112
Comprehension Score Rater 2	5	SMOG Index	12.1	Complex words	22
Reading Age (years)	21.5	Coleman Liau Index	18.2	Percentage of complex words	19.64
Flesch-Kincaid Reading Ease	29.3	Automated Readability Index	17.0	Average words per sentence	22.4
Flesch-Kincaid Grade Level	14.7	Sentences	5	Average syllables per word	1.83

Lay Summary (Extended Prompt)

This study looked at men with early prostate cancer who were expected to do well. The researchers wanted to see if a higher grade of cancer cells could mean a greater chance of the cancer returning. They studied almost 300 patients who had surgery using special robots to remove the prostate. They found that some patients had a higher grade of cancer, which made it more likely for the cancer to come back. Older age and certain test results before surgery were linked to these higher cancer grades. These findings suggest that even when prostate cancer appears low risk, more detailed testing might be needed. This could help doctors decide who needs closer care or more treatment to prevent the cancer from returning.

**Assessment Category**	**Score**	**Assessment Category**	**Score**	**Assessment Category**	**Score**
Comprehension Score Rater 1	5	Gunning Fog Score	9.0	Words	123
Comprehension Score Rater 2	5	SMOG Index	5.6	Complex words	6
Reading Age (years)	14.5	Coleman Liau Index	12.2	Percentage of complex words	4.88
Flesch-Kincaid Reading Ease	66.6	Automated Readability Index	9.8	Average words per sentence	17.57
Flesch-Kincaid Grade Level	8.3	Sentences	7	Average syllables per word	1.45

Manuscript 114

Cloître M, Benkhaled S, Boughdad S, Schaefer N, Prior JO, Zeverino M, et al. Spatial Distribution of Recurrence and Long-Term Toxicity Following Dose Escalation to the Dominant Intra-Prostatic Nodule for Intermediate–High-Risk Prostate Cancer: Insights from a Phase I/II Study. Cancers [Internet]. 2024 May 31;16(11):2097. Available from: http://dx.doi.org/10.3390/cancers16112097

Abstract

Objectives: We investigated spatial patterns between primary and recurrent tumor sites and assessed long-term toxicity after dose escalation stereotactic body radiation therapy (SBRT) to the dominant intra-prostatic nodule (DIN). Materials and methods: In 33 patients with intermediate-high-risk prostate cancer (PCa), doses up to 50 Gy were administered to the DIN. Recurrence sites were determined and compared to the original tumor development sites through multiparametric MRI and 68Ga-labeled prostate-specific membrane antigen (PSMA) positron emission tomography/computed tomography (68Ga-PSMA-PET/CT) images. Overlap rates, categorized as 75% or higher for full overlap, and 25-74% for partial overlap, were assessed. Long-term toxicity is reported. Results: All patients completed treatment, with only one receiving concomitant androgen deprivation therapy (ADT). Recurrences were diagnosed after a median of 33 months (range: 17-76 months), affecting 13 out of 33 patients (39.4%). Intra-prostatic recurrences occurred in 7 patients (21%), with ≥75% overlap in two, a partial overlap in another two, and no overlap in the remaining three patients. Notably, five patients with intra-prostatic recurrences had synchronous bone and/or lymph node metastases, while six patients had isolated bone or lymph node metastasis without intra-prostatic recurrences. Extended follow-up revealed late grade ≥ 2 GU and GI toxicity in 18% (n = 6) and 6% (n = 2) of the patients. Conclusions: Among patients with intermediate-high-risk PCa undergoing focal dose-escalated SBRT without ADT, DIN recurrences were infrequent. When present, these recurrences were typically located at the original site or adjacent to the initial tumor. Conversely, relapses beyond the DIN and

in extra-prostatic (metastatic) sites were prevalent, underscoring the significance of systemic ADT in managing this patient population. Advances in knowledge: Focal dose-escalated prostate SBRT prevented recurrences in the dominant nodule; however, extra-prostatic recurrence sites were frequent.

Keywords:

SBRT, dosimetry analysis, intermediate–high-risk, pattern of recurrence, prostate cancer, stereotactic treatment

<u>Original Lay Summary</u>

This reassessment of the findings from our phase I/II study seeks to evaluate the effectiveness of SBRT for treating local intermediate–high-risk prostate cancer in the absence of androgen deprivation therapy due to patient refusal, with a median follow-up of 6.5 years. Our primary focus is on analyzing the treated areas compared to recurrence patterns in the seven patients who experienced local relapse. Additionally, we provide updated data on late toxicities of grade ≥ 2, both genitourinary and gastrointestinal.

**Assessment Category**	**Score**	**Assessment Category**	**Score**	**Assessment Category**	**Score**
Comprehension Score Rater 1	4	Gunning Fog Score	17.0	Words	81
Comprehension Score Rater 2	4	SMOG Index	12.6	Complex words	19
Reading Age (years)	20.5	Coleman Liau Index	15.2	Percentage of complex words	23.46
Flesch-Kincaid Reading Ease	23.3	Automated Readability Index	13.5	Average words per sentence	20.25
Flesch-Kincaid Grade Level	15.0	Sentences	4	Average syllables per word	1.93

<u>Lay Summary (Simple Prompt)</u>

This study explored a new treatment method for prostate cancer by delivering extra radiation specifically to the main tumor within the prostate. Researchers treated 33 patients with higher risk cancer to understand where the disease might return and to monitor long-term side effects. Using advanced imaging techniques, they discovered that the extra dose rarely led to a return of cancer at the treated spot. When cancer did come back, it usually appeared close to the original tumor, although many cases showed spread beyond the prostate. Only a small number of patients experienced significant lasting side effects that affected their urinary or digestive systems. Overall, these findings suggest that while targeting the main tumor is effective in controlling local cancer, additional treatments may be necessary to manage cancer that spreads outside the prostate.

**Assessment Category**	**Score**	**Assessment Category**	**Score**	**Assessment Category**	**Score**
Comprehension Score Rater 1	5	Gunning Fog Score	15.2	Words	133
Comprehension Score Rater 2	5	SMOG Index	10.8	Complex words	21
Reading Age (years)	19.5	Coleman Liau Index	16.2	Percentage of complex words	15.79
Flesch-Kincaid Reading Ease	38.7	Automated Readability Index	15.3	Average words per sentence	22.17
Flesch-Kincaid Grade Level	13.4	Sentences	6	Average syllables per word	1.72

<u>Lay Summary (Extended Prompt)</u>

This study explored a new way of giving radiation to treat prostate cancer by focusing a higher dose on the main tumor in the prostate. The researchers treated patients with a higher risk of cancer and then used special scans to see where the cancer returned. They found that most patients did not have the cancer come back in the main treated area. When the cancer did return, it was usually found outside of that area. Some patients experienced side effects, but these were not common or very severe. The findings suggest that this focused radiation may help control the main tumor, but doctors may still need to use other treatments to stop the cancer from spreading elsewhere.

**Assessment Category**	**Score**	**Assessment Category**	**Score**	**Assessment Category**	**Score**
Comprehension Score Rater 1	4	Gunning Fog Score	9.9	Words	118
Comprehension Score Rater 2	4	SMOG Index	6.0	Complex words	6
Reading Age (years)	15.5	Coleman Liau Index	11.8	Percentage of complex words	5.08
Flesch-Kincaid Reading Ease	63.6	Automated Readability Index	10.5	Average words per sentence	19.67
Flesch-Kincaid Grade Level	9.3	Sentences	6	Average syllables per word	1.46

Manuscript 115

Mahmoud AM, Moustafa A, Day C, Ahmed ME, Zeina W, Marzouk UM, et al. Prostate Cancer Lung Metastasis: Clinical Insights and Therapeutic Strategies. Cancers [Internet]. 2024 May 30;16(11):2080. Available from: http://dx.doi.org/10.3390/cancers16112080

Abstract

Prostate cancer lung metastasis represents a clinical conundrum due to its implications for advanced disease progression and the complexities it introduces in treatment planning. As the disease progresses to distant sites such as the lung, the clinical management becomes increasingly intricate, requiring tailored therapeutic strategies to address the unique characteristics of metastatic lesions. This review seeks to synthesize the current state of knowledge surrounding prostate cancer metastasis to the lung, shedding light on the diverse array of clinical presentations encountered, ranging from subtle radiological findings to overt symptomatic manifestations. By examining the diagnostic modalities utilized in identifying this metastasis, including advanced imaging techniques and histopathological analyses, this review aims to provide insights into the diagnostic landscape and the challenges associated with accurately characterizing lung metastatic lesions in prostate cancer patients. Moreover, this review delves into the nuances of therapeutic interventions employed in managing prostate cancer lung metastasis, encompassing systemic treatments such as hormonal therapies and chemotherapy, as well as metastasis-directed therapies including surgery and radiotherapy.

Keywords:

lung metastasis, metastasis-directed therapy, prostate cancer, systemic therapy

Original Lay Summary

In our paper, we examine various facets of prostate cancer lung metastasis. We explore their clinical manifestations, such as respiratory symptoms and potential complications. Diagnostic modalities, including imaging techniques and biomarker analysis, are scrutinized for accurate identification. Treatment strategies, encompassing surgery, radiation therapy, chemotherapy, and targeted therapies, are discussed for their efficacy. Survival outcomes are assessed to gauge the effectiveness of different interventions. Through comprehensive analysis, we aim to provide insights into managing prostate cancer lung metastasis and enhancing patient care and outcomes.

**Assessment Category**	**Score**	**Assessment Category**	**Score**	**Assessment Category**	**Score**
Comprehension Score Rater 1	5	Gunning Fog Score	22.9	Words	83
Comprehension Score Rater 2	5	SMOG Index	14.1	Complex words	36
Reading Age (years)	24.0	Coleman Liau Index	24.3	Percentage of complex words	43.37
Flesch-Kincaid Reading Ease	-7.0	Automated Readability Index	17.5	Average words per sentence	13.83
Flesch-Kincaid Grade Level	17.7	Sentences	6	Average syllables per word	2.36

Lay Summary (Simple Prompt)

This study looks into how prostate cancer can spread to the lungs and the problems this causes for treatment. It reviews current knowledge about the different ways the cancer appears when it reaches the lungs, from very subtle signs on scans to clear symptoms. The research explains how doctors use various tests, such as imaging and tissue analysis, to detect these changes. It also discusses different treatment options, including medications, surgery, and radiation. By gathering and explaining this information, the study aims to help doctors better understand and manage this type of cancer spread, which could lead to improved treatment plans and better care for patients with advanced prostate cancer.

**Assessment Category**	**Score**	**Assessment Category**	**Score**	**Assessment Category**	**Score**
Comprehension Score Rater 1	5	Gunning Fog Score	13.9	Words	110
Comprehension Score Rater 2	5	SMOG Index	9.7	Complex words	14
Reading Age (years)	18.5	Coleman Liau Index	15.6	Percentage of complex words	12.73
Flesch-Kincaid Reading Ease	50.7	Automated Readability Index	14.7	Average words per sentence	22.0
Flesch-Kincaid Grade Level	11.7	Sentences	5	Average syllables per word	1.58

Lay Summary (Extended Prompt)

This study looks at how prostate cancer can spread to the lungs and why this makes treatment harder. The researchers review many cases to learn how this spread shows up in tests like scans and lab reports. They also examine different treatments such as medicines, surgery, and radiation to find out which work best. The goal is to help doctors find this type of cancer sooner and choose the best care for their patients. By understanding these challenges better, the study may lead to improved treatment plans and spark new ideas for research in this difficult area.

**Assessment Category**	**Score**	**Assessment Category**	**Score**	**Assessment Category**	**Score**
Comprehension Score Rater 1	5	Gunning Fog Score	11.1	Words	97
Comprehension Score Rater 2	5	SMOG Index	7.5	Complex words	8
Reading Age (years)	15.5	Coleman Liau Index	12.2	Percentage of complex words	8.25
Flesch-Kincaid Reading Ease	67.7	Automated Readability Index	10.7	Average words per sentence	19.4
Flesch-Kincaid Grade Level	8.6	Sentences	5	Average syllables per word	1.41

Manuscript 116

Westley R, Casey F, Mitchell A, Alexander S, Nill S, Murray J, et al. Stereotactic Body Radiotherapy (SBRT) to Localised Prostate Cancer in the Era of MRI-Guided Adaptive Radiotherapy: Doses Delivered in the HERMES Trial Comparing Two- and Five-Fraction Treatments. Cancers [Internet]. 2024 May 30;16(11):2073. Available from: http://dx.doi.org/10.3390/cancers16112073

Abstract

HERMES is a phase II trial of MRI-guided daily-adaptive radiotherapy (MRIgART) randomising men with localised prostate cancer to either 2-fractions of SBRT with a boost to the tumour or 5-fraction SBRT. In the context of this highly innovative regime the dose delivered must be carefully considered. The first ten patients recruited to HERMES were analysed in order to establish the dose received by the targets and organs at risk (OARS) in the context of intrafraction motion. A regression analysis was performed to measure how the volume of air within the rectum might further impact rectal dose secondary to the electron return effect (ERE). One hundred percent of CTV target objectives were achieved on the MRI taken prior to beam-on-time. The post-delivery MRI showed that high-dose CTV coverage was achieved in 90% of sub-fractions (each fraction is delivered in two sub-fractions) in the 2-fraction cohort and in 88% of fractions the 5-fraction cohort. Rectal D1 cm3 was the most exceeded constraint; three patients exceeded the D1 cm3 3 3, with a strong (R = 0.69) and very strong (R = 0.90) correlation in the 2-fraction and 5-fraction cohort respectively. Dose delivery specified in HERMES is feasible, although for some patients delivered doses to both target and OARs may vary from those planned.

Keywords:

MRI-guided adaptive radiotherapy, SBRT, intrafraction motion, prostate radiotherapy

Original Lay Summary

Radiotherapy to the prostate with curative intent was previously a procedure that was delivered over seven and a half weeks. The standard of care is now 20 treatments over 4 weeks; however, 5 treatments have recently been shown to be as good as 20 treatments in patients with intermediate risk disease. Fewer treatments mean that patients spend less time in the hospital and travelling to appointments and helps to reduce the burden on radiotherapy departments. When reducing the number of treatments, the dose per treatment is increased. This requires greater precision and certainty of dose delivery. The magnetic resonance linear accelerator (MRL) delivers radiotherapy under MRI guidance with the ability to modify the plan to adapt to any changes in the patient's anatomy, thus potentially enabling the safe delivery of higher doses per treatment. This study addresses the true dose delivered in two and five treatments of the MRL and whether target objects are being met.

**Assessment Category**	**Score**	**Assessment Category**	**Score**	**Assessment Category**	**Score**
Comprehension Score Rater 1	4	Gunning Fog Score	16.4	Words	156
Comprehension Score Rater 2	4	SMOG Index	12.0	Complex words	30
Reading Age (years)	19.5	Coleman Liau Index	14.5	Percentage of complex words	19.23
Flesch-Kincaid Reading Ease	37.2	Automated Readability Index	13.9	Average words per sentence	22.29
Flesch-Kincaid Grade Level	13.6	Sentences	7	Average syllables per word	1.74

<u>Lay Summary (Simple Prompt)</u>

This study explores a new way to treat localized prostate cancer using radiation that is precisely adjusted each day with magnetic resonance imaging. The researchers compared two treatment schedules: one with two larger sessions and another with five smaller sessions. They aimed to deliver the exact amount of radiation needed to target the tumor while minimizing exposure to nearby organs such as the rectum. By carefully measuring the doses received during treatment, the study found that the treatment plan was generally successful in covering the intended target. However, in some cases, patients received slightly higher doses to the rectum when more air was present. These results show that this innovative approach is feasible and may help improve the safety and effectiveness of prostate cancer treatment for future patients.

**Assessment Category**	**Score**	**Assessment Category**	**Score**	**Assessment Category**	**Score**
Comprehension Score Rater 1	5	Gunning Fog Score	15.4	Words	128
Comprehension Score Rater 2	5	SMOG Index	11.1	Complex words	22
Reading Age (years)	19.5	Coleman Liau Index	16.3	Percentage of complex words	17.19
Flesch-Kincaid Reading Ease	39.1	Automated Readability Index	14.9	Average words per sentence	21.33
Flesch-Kincaid Grade Level	13.1	Sentences	6	Average syllables per word	1.73

Lay Summary (Extended Prompt)

This study looks at a new way to treat prostate cancer using advanced imaging to guide radiation therapy. The researchers compared two treatment plans: one given in two sessions and one given in five sessions. They wanted to see if the planned dose of radiation was accurately delivered to the cancer while protecting nearby healthy organs. The study also explored how small movements or extra air in the body might change the dose. The results showed that most patients received the proper amount of radiation, although some small differences were found. These findings may help doctors plan safer and more effective treatments, leading to better outcomes for men with prostate cancer.

**Assessment Category**	**Score**	**Assessment Category**	**Score**	**Assessment Category**	**Score**
Comprehension Score Rater 1	4	Gunning Fog Score	11.7	Words	111
Comprehension Score Rater 2	4	SMOG Index	8.3	Complex words	12
Reading Age (years)	16.5	Coleman Liau Index	14.3	Percentage of complex words	10.81
Flesch-Kincaid Reading Ease	56.2	Automated Readability Index	11.9	Average words per sentence	18.5
Flesch-Kincaid Grade Level	10.0	Sentences	6	Average syllables per word	1.56

Manuscript 117

Sibert NT, Soff J, La Ferla S, Quaranta M, Kremer A, Kowalski C. Transforming a Large-Scale Prostate Cancer Outcomes Dataset to the OMOP Common Data Model—Experiences from a Scientific Data Holder's Perspective. Cancers [Internet]. 2024 May 30;16(11):2069. Available from: http://dx.doi.org/10.3390/cancers16112069

Abstract

To enhance international and joint research collaborations in prostate cancer research, data from different sources should use a common data model (CDM) that enables researchers to share their analysis scripts and merge results. The OMOP CDM maintained by OHDSI is such a data model developed for a federated data analysis with partners from different institutions that want to jointly investigate research questions using clinical care data. The German Cancer Society as the scientific lead of the Prostate Cancer Outcomes (PCO) study gathers data from prostate cancer care including routine oncological care data and survey data (incl. patient-reported outcomes) and uses a common data specification (called OncoBox Research Prostate) for this purpose. To further enhance research collaborations outside the PCO study, the purpose of this article is to describe the process of transferring the PCO study data to the internationally well-established OMOP CDM. This process was carried out together with an IT company that specialised in supporting research institutions to transfer their data to OMOP CDM. Of n = 49,692 prostate cancer cases with 318 data fields each, n = 392 had to be excluded during the OMOPing process, and n = 247 of the data fields could be mapped to OMOP CDM. The resulting PostgreSQL database with OMOPed PCO study data is now ready to use within larger research collaborations such as the EU-funded EHDEN and OPTIMA consortium.

Keywords:

OMOP CDM, data harmonisation, observational dataset, prostate cancer

Original Lay Summary

To obtain new insights in prostate cancer care, multiple clinics and research institutions must jointly analyse their data (by some, referred to as “big data analysis”). For this purpose, a common “data language” should be used. The OMOP CDM (common data model) is such a data language that can be used by different data holders. The aim of this article is to describe how a large dataset from a prostate cancer study with almost 50,000 prostate cancer cases was successfully transferred to the OMOP CDM standard. Challenges and their solutions during this process are discussed, and the importance of using data standards within prostate cancer research are reported from a scientific data holder’s perspective.

**Assessment Category**	**Score**	**Assessment Category**	**Score**	**Assessment Category**	**Score**
Comprehension Score Rater 1	5	Gunning Fog Score	13.7	Words	115
Comprehension Score Rater 2	5	SMOG Index	9.4	Complex words	13
Reading Age (years)	18.5	Coleman Liau Index	13.6	Percentage of complex words	11.3
Flesch-Kincaid Reading Ease	45.9	Automated Readability Index	13.6	Average words per sentence	23.0
Flesch-Kincaid Grade Level	12.6	Sentences	5	Average syllables per word	1.63

Lay Summary (Simple Prompt)

This study seeks to improve how information about prostate cancer is shared among scientists around the world. The researchers took a large collection of patient data and carefully converted it into a common format, making it easier for different research groups to work together. They gathered details from everyday cancer care and patient surveys and then worked to match most of the data to a widely accepted structure. Although a small portion of the data did not fit perfectly, the majority was successfully transformed. This work lays the foundation for better collaboration, which can speed up discoveries and lead to improved treatment options for prostate cancer in the future.

**Assessment Category**	**Score**	**Assessment Category**	**Score**	**Assessment Category**	**Score**
Comprehension Score Rater 1	5	Gunning Fog Score	14.6	Words	109
Comprehension Score Rater 2	5	SMOG Index	10.4	Complex words	16
Reading Age (years)	18.5	Coleman Liau Index	15.0	Percentage of complex words	14.68
Flesch-Kincaid Reading Ease	43.4	Automated Readability Index	14.1	Average words per sentence	21.8
Flesch-Kincaid Grade Level	12.6	Sentences	5	Average syllables per word	1.67

<u>Lay Summary (Extended Prompt)</u>

This study shows how researchers turned a large collection of prostate cancer information into a single, easy-to-use format. They did this so that scientists from different places can share their work and learn from each other. The team carefully moved data from nearly 50,000 cases into a new system and made adjustments to remove or fix any issues. The new database is now ready to support further research and help experts compare and combine results from many studies. This work makes it easier to find patterns in prostate cancer care and could lead to better treatments in the future.

**Assessment Category**	**Score**	**Assessment Category**	**Score**	**Assessment Category**	**Score**
Comprehension Score Rater 1	5	Gunning Fog Score	11.3	Words	102
Comprehension Score Rater 2	5	SMOG Index	7.5	Complex words	8
Reading Age (years)	15.5	Coleman Liau Index	11.8	Percentage of complex words	7.84
Flesch-Kincaid Reading Ease	61.7	Automated Readability Index	10.8	Average words per sentence	20.4
Flesch-Kincaid Grade Level	9.7	Sentences	5	Average syllables per word	1.47

Manuscript 118

Wang J, O'Dwyer E, Martinez Zuloaga J, Subramanian K, Hu JC, Jhanwar YS, et al. Reasons for Discordance between 68Ga-PSMA-PET and Magnetic Resonance Imaging in Men with Metastatic Prostate Cancer. Cancers [Internet]. 2024 May 29;16(11):2056. Available from: http://dx.doi.org/10.3390/cancers16112056

<u>Abstract</u>

Background: PSMA PET has emerged as a "gold standard" imaging modality for assessing prostate cancer metastases. However, it is not universally available, and this limits its impact. In contrast, whole-body MRI is much more widely available but misses more lesions. This study aims to improve the interpretation of whole-body MRI by comparing false negative scans retrospectively to PSMA PET.

Methods: This study was a retrospective sub-analysis of a prospectively collected database of patients who participated in a clinical trial of PSMA PET/MRI comparing PSMA PET and whole-body MRI from 2018-2021. Subjects whose separately read PSMA PET and MRI diagnostic reports showed discrepancies ("false negative" MRI cases) were selected for sub-analysis. The cases were reviewed by the same attending radiologist who originally read the scans. The radiologist noted specific features on MRI indicating metastatic disease that were initially missed.

Results: Of 263 cases, 38 (14%) met the inclusion criteria and were reviewed. Six classes of mpMRI false negatives were identified: anatomically normal (18, 47%), atypical MRI appearance (6, 16%), mischaracterization (1, 3%), undercall (6, 16%), obscured (4, 11%), and no abnormality on MRI (3, 8%). Considering that the atypical and undercalled cases could have been adjusted in retrospect, and that 4 additional cases had positive lesions to the same extent and 11 further cases had disease confined to the pelvis, only 11 (4%) of the original 263 would have had disease outside of a conventional radiation treatment plan.

Conclusion: Notably, almost 50% of the cases, including most lymph node metastases, were anatomically normal using standard criteria. This suggests that current anatomic criteria for evaluating prostate cancer lymph node metastases are not ideal, and there is a need for improved criteria. In addition, 32% of cases involved some element of human interpretive error, and, therefore, improving reader training may lead to more accurate results.

Keywords:

MRI, PET, PSMA, cancer, prostate, whole-body

<u>Original Lay Summary</u>

MRI uses magnetic pulses to create images of the body. PET scans use radioactive chemicals to determine what kinds of processes are going on in the body. PSMA is a chemical that is abundant on prostate cancer cells, so a PET scan using a radioactive chemical that attaches to PSMA shows where prostate cancer is, but this test has drawbacks: it is not widely available, it is expensive, and it exposes patients to radiation. However, a recent study found that PSMA PET was more accurate than MRI for finding areas where prostate cancer spreads. This study looks at the “false negative” cases—the specific cases where the MRI did not find prostate cancer when PSMA PET did—and how reading MRI can be improved.

**Assessment Category**	**Score**	**Assessment Category**	**Score**	**Assessment Category**	**Score**
Comprehension Score Rater 1	5	Gunning Fog Score	15.3	Words	122
Comprehension Score Rater 2	5	SMOG Index	10.7	Complex words	17
Reading Age (years)	18.5	Coleman Liau Index	11.8	Percentage of complex words	13.93
Flesch-Kincaid Reading Ease	51.7	Automated Readability Index	12.9	Average words per sentence	24.4
Flesch-Kincaid Grade Level	12.1	Sentences	5	Average syllables per word	1.54

<u>Lay Summary (Simple Prompt)</u>

This study looks at two common scans used to detect prostate cancer that has spread. One scan uses a special radioactive marker and is very accurate but is not available everywhere. The other, magnetic resonance imaging, is widely available but sometimes misses signs of cancer. Researchers reviewed cases where the common scan did not show cancer even though the more accurate scan did. They found that many missed areas appeared normal or were not recognized due to reading errors. The study suggests that improving how these scans are interpreted and enhancing training for those who read them could help doctors find cancer more reliably, which may lead to better treatment plans for patients.

**Assessment Category**	**Score**	**Assessment Category**	**Score**	**Assessment Category**	**Score**
Comprehension Score Rater 1	5	Gunning Fog Score	13.2	Words	113
Comprehension Score Rater 2	5	SMOG Index	9.5	Complex words	16
Reading Age (years)	17.5	Coleman Liau Index	14.2	Percentage of complex words	14.16
Flesch-Kincaid Reading Ease	53.0	Automated Readability Index	12.0	Average words per sentence	18.83
Flesch-Kincaid Grade Level	10.6	Sentences	6	Average syllables per word	1.59

Lay Summary (Extended Prompt)

This study looks at two ways to find cancer that has spread in men with prostate cancer. One scan, called a PSMA PET scan, is very good at showing cancer but is not available everywhere. The other scan, magnetic resonance imaging, is easier to get but sometimes misses cancer. The researchers checked past scans where the magnetic resonance imaging did not show cancer that the PSMA PET scan did. They found that in nearly half of these cases, the magnetic resonance imaging appeared normal even when cancer was present. They also discovered that mistakes in reading the scans played a role. These results suggest that current methods for checking these scans need to be improved. Better training for doctors and new guidelines could help in finding cancer more accurately, which may lead to improved care and research outcomes.

**Assessment Category**	**Score**	**Assessment Category**	**Score**	**Assessment Category**	**Score**
Comprehension Score Rater 1	5	Gunning Fog Score	11.8	Words	138
Comprehension Score Rater 2	5	SMOG Index	8.5	Complex words	17
Reading Age (years)	16.5	Coleman Liau Index	13.1	Percentage of complex words	12.32
Flesch-Kincaid Reading Ease	63.0	Automated Readability Index	10.3	Average words per sentence	17.25
Flesch-Kincaid Grade Level	8.8	Sentences	8	Average syllables per word	1.49

Manuscript 119

Hoffmann M, Ermler TF, Hoffmann F, Alexa R, Kranz J, Steinke N, et al. Therapeutic and Diagnostic Potential of Folic Acid Receptors and Glycosylphosphatidylinositol (GPI) Transamidase in Prostate Cancer. Cancers [Internet]. 2024 May 25;16(11):2008. Available from: http://dx.doi.org/10.3390/cancers16112008

Abstract

Due to the proliferation-induced high demand of cancer cells for folic acid (FA), significant overexpression of folate receptors 1 (FR1) is detected in most cancers. To our knowledge, a detailed characterization of FR1 expression and regulation regarding therapeutic and diagnostic feasibilities in prostate cancer (PCa) has not been described. In the present study, cell cultures, as well as tissue sections, were analyzed using Western blot, qRT-PCR and immunofluorescence. In addition, we utilized FA-functionalized lipoplexes to characterize the potential of FR1-targeted delivery into PCa cells. Interestingly, we detected a high level of FR1-mRNA in healthy prostate epithelial cells and healthy prostate tissue. However, we were able to show that PCa cells in vitro and PCa tissue showed a massively enhanced FR1 membrane localization where the receptor can finally gain its function. We were able to link these changes to the overexpression of GPI-transamidase (GPI-T) by image analysis. PCa cells in vitro and PCa tissue show the strongest overexpression of GPI-T and thereby induce FR1 membrane localization. Finally, we utilized FA-functionalized lipoplexes to selectively transfer pDNA into PCa cells and demonstrate the therapeutic potential of FR1. Thus, FR1 represents a very promising candidate for targeted therapeutic transfer pathways in PCa and in combination with GPI-T, may provide predictive imaging in addition to established diagnostics.

Keywords:

diagnostics, folic acid receptor, glycosylphosphatidylinositol transamidase, prostate cancer, targeted drug delivery

<u>Original Lay Summary</u>

Folate receptors can serve to increase folate uptake in cancer cells through significant overexpression, which is ultimately critical for cell proliferation and tumor growth. Therefore, the characterization of folate receptor expression in cancer presents exciting opportunities in multiple dimensions. On the one hand, the folate receptor can be used as a target for selective transfer of therapeutic agents. On the other hand, it can be used for risk stratification. We found that in prostate cancer (PCa), activation and membrane integration by GPI–transamidase exhibit significant changes. By image analysis, we could demonstrate these differences to healthy control cells and tissues, and we expect high potential in this approach to identify biological subtypes and allow risk stratification based on these alterations in PCa.

**Assessment Category**	**Score**	**Assessment Category**	**Score**	**Assessment Category**	**Score**
Comprehension Score Rater 1	5	Gunning Fog Score	18.0	Words	121
Comprehension Score Rater 2	5	SMOG Index	13.1	Complex words	31
Reading Age (years)	21.5	Coleman Liau Index	18.1	Percentage of complex words	25.62
Flesch-Kincaid Reading Ease	23.5	Automated Readability Index	15.7	Average words per sentence	20.17
Flesch-Kincaid Grade Level	15.0	Sentences	6	Average syllables per word	1.93

Lay Summary (Simple Prompt)

This study explores how a protein that binds folic acid, which is crucial for cell growth, behaves differently in healthy versus cancerous prostate cells. The researchers discovered that in prostate cancer, this protein is much more active on the cell surface, a change helped by another enzyme. By using specially designed particles that target this protein, they successfully delivered genetic material into the cancer cells. These findings suggest that both the folic acid binding protein and the supporting enzyme could be key in developing new, more precise methods for diagnosing and treating prostate cancer, potentially leading to therapies that target cancer cells more effectively while sparing healthy tissue.

**Assessment Category**	**Score**	**Assessment Category**	**Score**	**Assessment Category**	**Score**
Comprehension Score Rater 1	5	Gunning Fog Score	17.1	Words	108
Comprehension Score Rater 2	5	SMOG Index	11.9	Complex words	17
Reading Age (years)	21.5	Coleman Liau Index	17.0	Percentage of complex words	15.74
Flesch-Kincaid Reading Ease	32.2	Automated Readability Index	18.3	Average words per sentence	27.0
Flesch-Kincaid Grade Level	15.5	Sentences	4	Average syllables per word	1.74

Lay Summary (Extended Prompt)

This study looks for new ways to find and treat prostate cancer. Cancer cells need a lot of folic acid, and they show extra markers on their surface to grab it. The researchers studied both cells grown in the lab and real tissue samples to see where these markers appear. They also examined an enzyme that seems to help move these markers to the cell surface. By using special particles with folic acid, they were able to send medicine directly into the cancer cells. Their work shows that these markers and the enzyme are much more common in cancer cells than in healthy ones. This discovery could lead to better tests and treatments for prostate cancer in the future.

**Assessment Category**	**Score**	**Assessment Category**	**Score**	**Assessment Category**	**Score**
Comprehension Score Rater 1	5	Gunning Fog Score	8.8	Words	119
Comprehension Score Rater 2	5	SMOG Index	5.6	Complex words	6
Reading Age (years)	13.5	Coleman Liau Index	11.0	Percentage of complex words	5.04
Flesch-Kincaid Reading Ease	73.0	Automated Readability Index	8.5	Average words per sentence	17.0
Flesch-Kincaid Grade Level	7.3	Sentences	7	Average syllables per word	1.38

Manuscript 120

Compérat E, Kläger J, Rioux-Leclercq N, Oszwald A, Wasinger G. Cribriform versus Intraductal: How to Determine the Difference. Cancers [Internet]. 2024 May 24;16(11):2002. Available from: http://dx.doi.org/10.3390/cancers16112002

Abstract

Over the years, our understanding of cribriform and intraductal prostate cancer (PCa) has evolved significantly, leading to substantial changes in their classification and clinical management. This review discusses the histopathological disparities between intraductal and cribriform PCa from a diagnostic perspective, aiming to aid pathologists in achieving accurate diagnoses. Furthermore, it discusses the ongoing debate surrounding the different recommendations between ISUP and GUPS, which pose challenges for practicing pathologists and complicates consensus among them. Recent studies have shown promising results in integrating these pathological features into clinical decision-making tools, improving predictions of PCa recurrence, cancer spread, and mortality. Future research efforts should focus on further unraveling the biological backgrounds of these entities and their implications for clinical management to ultimately improve PCa patient outcomes.

Keywords:

cribriform, intraductal carcinoma, pathological risk factors, prostate cancer

<u>Original Lay Summary</u>

Prostate cancer is a common and challenging disease among men, driving researchers to find better ways to understand and manage it. The understanding of two specific types, cribriform and intraductal prostate cancer (IDC-P), has evolved significantly over the years. This review aims to help pathologists differentiate between cribriform prostate cancer and IDC-P, and addresses current recommendations. Recent studies show that including these features in decision-making tools enhances predictions of cancer recurrence, spread, and patient outcomes. Future research should further focus on their pathological and molecular aspects to improve risk stratification, treatment approaches, and patient care.

**Assessment Category**	**Score**	**Assessment Category**	**Score**	**Assessment Category**	**Score**
Comprehension Score Rater 1	5	Gunning Fog Score	16.0	Words	98
Comprehension Score Rater 2	5	SMOG Index	11.6	Complex words	20
Reading Age (years)	21.5	Coleman Liau Index	19.8	Percentage of complex words	20.41
Flesch-Kincaid Reading Ease	28.1	Automated Readability Index	16.8	Average words per sentence	19.6
Flesch-Kincaid Grade Level	14.2	Sentences	5	Average syllables per word	1.88

<u>Lay Summary (Simple Prompt)</u>

This study examines two types of prostate cancer tissue patterns that can be confused with one another. The researchers aim to clarify the differences between these patterns so that doctors can diagnose and treat prostate cancer more accurately. By looking closely at tissue structures and current expert debates, the study shows that understanding these distinctions can lead to better tools for predicting if the cancer will return or spread. In the long run, this work may guide future research and help improve patient care by making treatment decisions more precise.

**Assessment Category**	**Score**	**Assessment Category**	**Score**	**Assessment Category**	**Score**
Comprehension Score Rater 1	5	Gunning Fog Score	13.9	Words	90
Comprehension Score Rater 2	5	SMOG Index	9.7	Complex words	11
Reading Age (years)	18.5	Coleman Liau Index	15.3	Percentage of complex words	12.22
Flesch-Kincaid Reading Ease	49.6	Automated Readability Index	14.7	Average words per sentence	22.5
Flesch-Kincaid Grade Level	11.9	Sentences	4	Average syllables per word	1.59

<u>Lay Summary (Extended Prompt)</u>

This study looks at two kinds of prostate cancer. One kind makes small holes in the tissue, while the other grows inside tube-like spaces. The authors want to help doctors tell these two types apart so they can choose the best care for patients. They discuss the challenges and debates among experts about how to test for and treat these cancers. By studying these differences, the research shows that doctors may better predict if the cancer will come back or spread. In short, this work aims to improve how prostate cancer is diagnosed and managed, which could lead to clearer tests, better treatments, and healthier lives for patients.

**Assessment Category**	**Score**	**Assessment Category**	**Score**	**Assessment Category**	**Score**
Comprehension Score Rater 1	4	Gunning Fog Score	8.7	Words	109
Comprehension Score Rater 2	4	SMOG Index	5.0	Complex words	4
Reading Age (years)	14.5	Coleman Liau Index	12.2	Percentage of complex words	3.67
Flesch-Kincaid Reading Ease	75.9	Automated Readability Index	10.0	Average words per sentence	18.17
Flesch-Kincaid Grade Level	7.2	Sentences	6	Average syllables per word	1.33

Manuscript 121

Chen Y, Lin PH, Freedland SJ, Chi JT. Metabolic Response to Androgen Deprivation Therapy of Prostate Cancer. Cancers [Internet]. 2024 May 24;16(11):1991. Available from: http://dx.doi.org/10.3390/cancers16111991

Abstract

Prostate cancer (PC) stands as the most frequently diagnosed non-skin cancer and ranks as the second highest cause of cancer-related deaths among men in the United States. For those facing non-metastatic PC necessitating intervention, solely local treatments may not suffice, leading to a possible transition toward systemic therapies, including androgen deprivation therapy (ADT), chemotherapy, and therapies targeting androgen. Yet, these systemic treatments often bring about considerable adverse effects. Additionally, it is observed that overweight men are at a higher risk of developing aggressive forms of PC, advancing to metastatic stages, and succumbing to the disease. Consequently, there is a pressing demand for new treatment options that carry fewer side effects and enhance the current standard treatments, particularly for the majority of American men who are overweight or obese. In this article, we will review the metabolic response to ADT and how lifestyle modulation can mitigate these ADT-associated metabolic responses with a particular focus on the two clinical trials, Carbohydrate and Prostate Study 1 (CAPS1) and Carbohydrate and Prostate Study 2 (CAPS2), which tested the effects of low-carbohydrate diets on the metabolic side effects of ADT and PC progression, respectively. Furthermore, we will summarize the findings of serum metabolomic studies to elucidate the potential mechanisms by which ADT and low-carbohydrate diets can affect the metabolic response to mitigate the metabolic side effects while maximizing therapeutic efficacy.

Keywords:

ADT, diet, metabolic response, prostate cancer

<u>Original Lay Summary</u>

Prostate cancer is the most common cancer in American men, aside from skin cancer, and it is the second deadliest. For patients with advanced or metastatic disease, systemic therapies are warranted. The backbone of these systemic therapies is androgen deprivation therapy (ADT). While providing effective tumor control, ADT can have significant side effects, especially problematic for the many overweight or obese men who are at higher risk for aggressive cancer. Our review investigates how lifestyle changes, particularly a low- carbohydrate diet, might reduce the unwanted metabolic effects of ADT. We specifically discuss two clinical trials, Carbohydrate and Prostate Study 1 (CAPS1) and Carbohydrate and Prostate Study 2 (CAPS2), examining this diet's impact on metabolic issues and cancer progression. Additionally, we explore how ADT might influence metabolism, aiming to improve patient outcomes by balancing treatment efficacy with quality of life.

**Assessment Category**	**Score**	**Assessment Category**	**Score**	**Assessment Category**	**Score**
Comprehension Score Rater 1	5	Gunning Fog Score	17.4	Words	139
Comprehension Score Rater 2	5	SMOG Index	13.1	Complex words	36
Reading Age (years)	21.5	Coleman Liau Index	17.7	Percentage of complex words	25.9
Flesch-Kincaid Reading Ease	24.2	Automated Readability Index	15.3	Average words per sentence	19.86
Flesch-Kincaid Grade Level	14.8	Sentences	7	Average syllables per word	1.92

Lay Summary (Simple Prompt)

Prostate cancer is one of the most common cancers among men and can be particularly dangerous for those who are overweight. Treatments often include hormone therapy that lowers male hormone levels, but these treatments can cause unwanted changes in the body's metabolism. This study examines how the body responds to hormone therapy and explores whether a low-carbohydrate diet can help reduce these side effects. By looking at dietary changes during treatment in two clinical trials, the research aims to find better ways to lessen the harmful effects of therapy and improve patient well-being. The findings could lead to treatment plans that combine lifestyle changes with standard care, offering safer and more effective options for patients and providing valuable insights for future research in this field.

**Assessment Category**	**Score**	**Assessment Category**	**Score**	**Assessment Category**	**Score**
Comprehension Score Rater 1	5	Gunning Fog Score	15.5	Words	127
Comprehension Score Rater 2	5	SMOG Index	10.7	Complex words	17
Reading Age (years)	19.5	Coleman Liau Index	15.5	Percentage of complex words	13.39
Flesch-Kincaid Reading Ease	41.8	Automated Readability Index	16.3	Average words per sentence	25.4
Flesch-Kincaid Grade Level	13.7	Sentences	5	Average syllables per word	1.65

<u>Lay Summary (Extended Prompt)</u>

This study explores how a common treatment for prostate cancer, which reduces male hormones, can affect the body's metabolism. Many men with this cancer are overweight, which may make treatment side effects worse. The research examines whether a low-carbohydrate diet can lessen these unwanted effects. The authors studied two groups of patients to see how eating fewer carbohydrates might change the body's reaction to treatment. They also looked at blood tests to understand the changes better. The goal is to find a safer way to treat prostate cancer that makes patients feel better and improves treatment outcomes. This work may help doctors choose treatments that are easier on the body and support healthier lifestyles for those fighting prostate cancer.

**Assessment Category**	**Score**	**Assessment Category**	**Score**	**Assessment Category**	**Score**
Comprehension Score Rater 1	5	Gunning Fog Score	10.2	Words	120
Comprehension Score Rater 2	5	SMOG Index	7.1	Complex words	10
Reading Age (years)	16.5	Coleman Liau Index	15.1	Percentage of complex words	8.33
Flesch-Kincaid Reading Ease	59.0	Automated Readability Index	11.8	Average words per sentence	17.14
Flesch-Kincaid Grade Level	9.3	Sentences	7	Average syllables per word	1.54

Manuscript 122

Al-Ibraheem A, Abdlkadir AS, Sweedat DA, Maus S, Al-Rasheed U, Salah S, et al. From Despair to Hope: First Arabic Experience of 177Lu-PSMA and 161Tb-PSMA Therapy for Metastatic Castration-Resistant Prostate Cancer. Cancers [Internet]. 2024 May 23;16(11):1974. Available from: http://dx.doi.org/10.3390/cancers16111974

Abstract

The objective of this retrospective study is to assess the effectiveness and safety of two beta-emitting prostate-specific membrane antigen (PSMA) radioligands, [177Lu]Lu and [161Tb]Tb, in heavily treated patients with metastatic castration-resistant prostate cancer (mCRPC). A total of 148 cycles of beta-emitting PSMA radioligand therapy were given to 53 patients at a specialized cancer care center in Amman, Jordan. This treatment was offered following the exhaustion of all prior treatment modalities. Approximately half of the cases (n = 26) demonstrated an initial partial response to PSMA radioligand therapy. Moreover, roughly one-fourth of the patients (n = 13) exhibited a sustained satisfactory biochemical response, which qualified them to receive a total of six PSMA radioligand therapy cycles and maintain continued follow-up for additional treatment cycles. This was reflected by an adequate prostate-specific antigen (PSA) decline and a concomitant partial response evident on [68Ga]Ga-PSMA positron emission tomography/computed tomography imaging. A minority of patients (n= 18; 34%) experienced side effects. Generally, these were low-grade and self-limiting toxicities. This study endorses previous research evidence about PSMA radioligand therapy's safety and efficacy. It also provides the first clinical insight from patients of Arab ethnicity. This should facilitate and promote further evidence, both regionally and internationally.

Keywords:

161Tb-PSMA, 177Lu-PSMA, PRLT, PSA, PSMA therapy, PSMA-RLT, mCRPC, prostate cancer, radioligand therapy, specific membrane antigen

Original Lay Summary

The recent approval of [177Lu]Lu-prostate-specific membrane antigen (PSMA) for managing metastatic castration-resistant prostate cancer (mCRPC) has catalyzed the innovation of various PSMA-targeted radiopharmaceuticals. In our retrospective study, we explored both safety and efficacy of two beta-emitting PSMA radioligands, [177Lu]Lu and [161Tb]Tb, for mCRPC therapy. Our study included 53 patients and reinforced prior evidence validating the clinical safety and efficacy of these radioligands. Our research suggested that these treatments are characterized by a favorable safety profile with negligible toxicity. Moreover, [161Tb]Tb-PSMA recipients, though trialed in a smaller patient sample, yielded concordant outcomes on par with those receiving [177Lu]Lu-PSMA, highlighting its promise as an alternative therapy and warrants additional investigation.

**Assessment Category**	**Score**	**Assessment Category**	**Score**	**Assessment Category**	**Score**
Comprehension Score Rater 1	5	Gunning Fog Score	23.1	Words	115
Comprehension Score Rater 2	5	SMOG Index	16.3	Complex words	40
Reading Age (years)	24.0	Coleman Liau Index	20.1	Percentage of complex words	34.78
Flesch-Kincaid Reading Ease	5.5	Automated Readability Index	18.8	Average words per sentence	23.0
Flesch-Kincaid Grade Level	18.2	Sentences	5	Average syllables per word	2.1

<u>Lay Summary (Simple Prompt)</u>

This study looked at a new treatment option for advanced prostate cancer when other therapies no longer work. Researchers used two types of radioactive drugs designed to target cancer cells directly. Many patients experienced early signs of improvement, and some showed lasting benefits that allowed them to continue with further treatment cycles. The side effects were usually mild and went away on their own. Notably, this is the first report of these treatments being used in Arab patients, adding a fresh perspective to earlier findings. The results offer hope for better management of advanced prostate cancer and support more research into these promising treatment methods.

**Assessment Category**	**Score**	**Assessment Category**	**Score**	**Assessment Category**	**Score**
Comprehension Score Rater 1	4	Gunning Fog Score	12.0	Words	105
Comprehension Score Rater 2	3	SMOG Index	8.6	Complex words	13
Reading Age (years)	17.5	Coleman Liau Index	16.0	Percentage of complex words	12.38
Flesch-Kincaid Reading Ease	48.9	Automated Readability Index	12.8	Average words per sentence	17.5
Flesch-Kincaid Grade Level	10.8	Sentences	6	Average syllables per word	1.66

<u>Lay Summary (Extended Prompt)</u>

This study explored a new treatment for advanced prostate cancer when other treatments have not worked. The researchers tested two drugs on patients who had already undergone many treatments. They found that many patients showed signs of improvement, and some experienced lasting benefits. Most of the side effects were mild and went away on their own. This work is important because it offers hope for patients who have few options left and provides new information that could help doctors improve future care.

**Assessment Category**	**Score**	**Assessment Category**	**Score**	**Assessment Category**	**Score**
Comprehension Score Rater 1	3	Gunning Fog Score	10.0	Words	82
Comprehension Score Rater 2	3	SMOG Index	7.0	Complex words	7
Reading Age (years)	15.5	Coleman Liau Index	14.6	Percentage of complex words	8.54
Flesch-Kincaid Reading Ease	63.3	Automated Readability Index	11.1	Average words per sentence	16.4
Flesch-Kincaid Grade Level	8.5	Sentences	5	Average syllables per word	1.5

Manuscript 123

Santourlidis S, Araúzo-Bravo MJ, Erichsen L, Bendhack ML. Epigenetics Meets CAR-T-Cell Therapy to Fight Cancer. Cancers [Internet]. 2024 May 20;16(10):1941. Available from: http://dx.doi.org/10.3390/cancers16101941

Abstract

Based on the impressive success of Car-T-cell therapy in the treatment of hematological malignancies, a broad application for solid tumors also appears promising. However, some important hurdles need to be overcome. One of these is certainly the identification of specific target antigens on cancer cells. Hypomethylation is a characteristic epigenetic aberration in many tumor entities. Genome-wide screenings for consistent DNA hypomethylations in tumors enable the identification of aberrantly upregulated transcripts, which might result in cell surface proteins. Thus, this approach provides a new perspective for the discovery of potential new Car-T-cell target antigens for almost every tumor entity. First, we focus on this approach as a possible treatment for prostate cancer.

Keywords:

Car-T-cell targets, Car-T-cell therapy, epigenetics, hypomethylation

<u>Original Lay Summary</u>

Cancer treatment could be revolutionized by using particular CAR-T-cell therapies for all solid tumors. Finding appropriate CAR-T-cell antigens for every tumor entity would be necessary for this though. Our findings provide new insight into the possibility of employing CAR-T-cell therapy to treat nearly all cancers, as genome-wide screening following consistent occurring DNA hypomethylations may uncover novel antigens for every cancer entity.

**Assessment Category**	**Score**	**Assessment Category**	**Score**	**Assessment Category**	**Score**
Comprehension Score Rater 1	3	Gunning Fog Score	20.2	Words	68
Comprehension Score Rater 2	3	SMOG Index	14.5	Complex words	19
Reading Age (years)	22.5	Coleman Liau Index	16.7	Percentage of complex words	27.94
Flesch-Kincaid Reading Ease	18.4	Automated Readability Index	15.9	Average words per sentence	22.67
Flesch-Kincaid Grade Level	16.3	Sentences	3	Average syllables per word	1.96

Lay Summary (Simple Prompt)

This study explores a new approach to treat cancer by combining a specialized cell therapy with the study of DNA changes in tumor cells. The research focuses on finding unique markers on cancer cells that arise from abnormal DNA patterns. These markers, which may appear on the cell surface due to reduced chemical modifications, could serve as clear targets for the cell therapy. By identifying these targets, the study aims to extend a treatment that has worked well for blood cancers to solid tumors like prostate cancer. This work not only addresses a major hurdle in current treatments but also has the potential to improve how precisely cancer can be attacked, ultimately offering new hope and valuable insights for future cancer therapies.

**Assessment Category**	**Score**	**Assessment Category**	**Score**	**Assessment Category**	**Score**
Comprehension Score Rater 1	4	Gunning Fog Score	20.2	Words	68
Comprehension Score Rater 2	4	SMOG Index	14.5	Complex words	19
Reading Age (years)	22.5	Coleman Liau Index	16.7	Percentage of complex words	27.94
Flesch-Kincaid Reading Ease	18.4	Automated Readability Index	15.9	Average words per sentence	22.67
Flesch-Kincaid Grade Level	16.3	Sentences	3	Average syllables per word	1.96

Lay Summary (Extended Prompt)

Cancer is a complex disease, and finding better ways to treat it is an ongoing challenge. CAR-T-cell therapy, which uses specially modified immune cells to fight cancer, has been very successful in treating blood cancers. However, using this approach for solid tumors, like prostate cancer, is more difficult because it is hard to find unique markers on tumor cells that CAR-T cells can target. This study explores a new way to identify these markers by looking at changes in the tumor's DNA. Specifically, we focus on "hypomethylation," a common change in cancer cells that can lead to the production of new surface proteins. By identifying these proteins, we hope to discover better targets for CAR-T-cell therapy, making it more effective for solid tumors. Our findings could help researchers develop new treatments that improve survival and quality of life for cancer patients.

**Assessment Category**	**Score**	**Assessment Category**	**Score**	**Assessment Category**	**Score**
Comprehension Score Rater 1	4	Gunning Fog Score	13.6	Words	147
Comprehension Score Rater 2	4	SMOG Index	9.8	Complex words	20
Reading Age (years)	17.5	Coleman Liau Index	12.9	Percentage of complex words	13.61
Flesch-Kincaid Reading Ease	49.7	Automated Readability Index	12.0	Average words per sentence	21.0
Flesch-Kincaid Grade Level	11.5	Sentences	7	Average syllables per word	1.61

Manuscript 124

Waseem M, Wang BD. Combination of miR-99b-5p and Enzalutamide or Abiraterone Synergizes the Suppression of EMT-Mediated Metastasis in Prostate Cancer. Cancers [Internet]. 2024 May 19;16(10):1933. Available from: http://dx.doi.org/10.3390/cancers16101933

Abstract

Prostate cancer (PCa) is the most frequently diagnosed cancer and second leading cause of cancer deaths among American men. Androgen deprivation therapy (ADT) has been systemically applied as a first-line therapy for PCa patients. Despite the initial responses, the majority of patients under ADT eventually experienced tumor progression to castration-resistant prostate cancer (CRPC), further leading to tumor metastasis to distant organs. Therefore, identifying the key molecular mechanisms underlying PCa progression remains crucial for the development of novel therapies for metastatic PCa. Previously, we identified that tumor-suppressive miR-99b-5p is frequently downregulated in aggressive African American (AA) PCa and European American (EA) CRPC, leading to upregulation of mTOR, androgen receptor (AR), and HIF-1α signaling. Given the fact that mTOR and HIF-1α signaling are critical upstream pathways that trigger the activation of epithelial-mesenchymal transition (EMT), we hypothesized that miR-99b-5p may play a critical functional role in regulating EMT-mediated PCa metastasis. To test this hypothesis, a series of cell biology, biochemical, and in vitro functional assays (wound healing, transwell migration, cell/ECM adhesion, and capillary-like tube formation assays) were performed to examine the effects of miR-99b-5p mimic on regulating EMT-mediated PCa metastasis processes. Our results have demonstrated that miR-99b-5p simultaneously targets MTOR and AR signaling, leading to upregulation of E-cadherin, downregulation of Snail/N-cadherin/Vimentin, and suppression of EMT-mediated PCa metastasis. MiR-99b-5p alone and in combination with enzalutamide or abiraterone significantly inhibits the EMT-mediated metastasis of AA PCa and EA CRPC.

Keywords:

AA PCa, AR signaling, CRPC, EMT, PI3K/AKT/mTOR signaling, abiraterone, enzalutamide, metastasis, miR-99b-5p, prostate cancer

<u>Original Lay Summary</u>

To date, effective treatment for metastatic prostate cancer (PCa) remains a clinical challenge. Our previous study demonstrated that tumor suppressive miR-99b-5p negatively regulates the expression of androgen receptor (AR) and mTOR, potentially serving as a therapeutic agent for aggressive PCa. In this study, we show for the first time that combination of miR-99b-5p mimic and second-generation AR antagonist (such as enzalutamide or abiraterone) simultaneously targets AR and mTOR signaling, consequently inhibiting epithelial-mesenchymal transition (EMT) in PCa. This novel combination therapy may provide a unique opportunity for effectively suppressing EMT-mediated metastasis in aggressive PC, especially African American PCa and castration-resistant prostate cancer (CRPC).

**Assessment Category**	**Score**	**Assessment Category**	**Score**	**Assessment Category**	**Score**
Comprehension Score Rater 1	5	Gunning Fog Score	24.8	Words	110
Comprehension Score Rater 2	5	SMOG Index	18.2	Complex words	40
Reading Age (years)	24.0	Coleman Liau Index	18.9	Percentage of complex words	36.36
Flesch-Kincaid Reading Ease	-1.8	Automated Readability Index	20.1	Average words per sentence	27.5
Flesch-Kincaid Grade Level	20.3	Sentences	4	Average syllables per word	2.14

Lay Summary (Simple Prompt)

Prostate cancer is a common and life-threatening disease in men. While standard treatments that reduce male hormones (androgen deprivation therapy) can slow cancer growth, many patients eventually develop a more aggressive form called castration-resistant prostate cancer (CRPC), which spreads to other parts of the body. This study explores a potential new approach to slow the spread of prostate cancer by using a small molecule called miR-99b-5p. Researchers found that miR-99b-5p can block key signals that drive cancer cell movement, helping to prevent metastasis. When combined with existing treatments like enzalutamide or abiraterone, miR-99b-5p further enhances the suppression of cancer spread. These findings suggest that miR-99b-5p could be a promising addition to current therapies, offering new hope for patients with advanced prostate cancer. Further research is needed to explore its full potential in clinical settings.

**Assessment Category**	**Score**	**Assessment Category**	**Score**	**Assessment Category**	**Score**
Comprehension Score Rater 1	4	Gunning Fog Score	14.9	Words	144
Comprehension Score Rater 2	4	SMOG Index	10.7	Complex words	24
Reading Age (years)	18.5	Coleman Liau Index	15.5	Percentage of complex words	16.67
Flesch-Kincaid Reading Ease	45.5	Automated Readability Index	13.9	Average words per sentence	20.57
Flesch-Kincaid Grade Level	12.0	Sentences	7	Average syllables per word	1.66

<u>Lay Summary (Extended Prompt)</u>

Prostate cancer is a common and serious disease that can spread to other parts of the body, making treatment difficult. Many patients receive hormone-based therapies like enzalutamide or abiraterone to slow cancer growth, but over time, the cancer often becomes resistant and continues to spread. This study explores the role of a small molecule called miR-99b-5p, which is naturally found in the body but is often missing in aggressive prostate cancer. Researchers found that restoring miR-99b-5p helps block key pathways that drive cancer spread. When combined with existing treatments, miR-99b-5p further reduces cancer cells' ability to move and invade new areas. These findings suggest that adding miR-99b-5p to current therapies could improve treatment outcomes for patients with advanced prostate cancer and potentially slow down or prevent its spread. This discovery may lead to better treatment options in the future.

**Assessment Category**	**Score**	**Assessment Category**	**Score**	**Assessment Category**	**Score**
Comprehension Score Rater 1	4	Gunning Fog Score	13.1	Words	148
Comprehension Score Rater 2	4	SMOG Index	9.1	Complex words	17
Reading Age (years)	17.5	Coleman Liau Index	14.2	Percentage of complex words	11.49
Flesch-Kincaid Reading Ease	50.5	Automated Readability Index	13.1	Average words per sentence	21.14
Flesch-Kincaid Grade Level	11.5	Sentences	7	Average syllables per word	1.59

Manuscript 125

Goßler C, May M, Weikert S, Lenart S, Ponholzer A, Dreissig C, et al. Long-Term Follow-Up of Peritoneal Interposition Flap in Symptomatic Lymphocele Reduction following Robot-Assisted Radical Prostatectomy: Insights from the PIANOFORTE Trial. Cancers [Internet]. 2024 May 19;16(10):1932. Available from: http://dx.doi.org/10.3390/cancers16101932

Abstract

The available randomised controlled trials (RCTs) assessing the influence of peritoneal interposition flaps (PIF) on the reduction of symptomatic lymphoceles (sLCs) post robot-assisted radical prostatectomy (RARP) do not constitute a sufficient follow-up (FU) to assess the long-term effects. The PIANOFORTE trial was the first of these RCTs, showing no sLC reduction at the 3-month FU. Therefore, all 232 patients from the PIANOFORTE trial were invited for long-term FU. One hundred seventy-six patients (76%) presented themselves for FU and constituted the study group (SG). The median FU duration was 43 months. No significant differences in group allocation or LC endpoints at 90 days were observed between SG patients and patients not presenting themselves for the FU. During the FU period, four patients (2.3%) in the SG developed sLCs, and six patients (3.4%) developed asymptomatic lymphoceles (aLCs), which persisted in five patients (2.9%). There were no significant differences between PIF and non-PIF regarding sLC/aLC formation or persistence, newly developed complications, stress urinary incontinence or biochemical/clinical tumour recurrence. Therefore, this long-term FU confirms the primary outcomes of the PIANOFORTE trial that, while PIF does not impact complications or functionality, it does not reduce sLC/aLC rates. Furthermore, it shows the potential occurrence of LC after the third postoperative month.

Keywords:

RARP, lymphocele, prostate cancer, prostatic neoplasia, robotics

<u>Original Lay Summary</u>

Lymphoceles (lymphatic fluid collections in the pelvis) are a common complication of robot-assisted radical prostatectomy. Peritoneal interposition flaps have been proposed as an intraoperative modification to reduce lymphocele formation. However, data from randomised controlled trials on this subject are not conclusive. In particular, data on long-term efficacy and complications are lacking. The PIANOFORTE trial was the first randomised controlled trial exploring this subject and showed a negative outcome regarding lymphocele reduction by performing a peritoneal interposition flap. In this long-term follow-up of the trial (median 43 months postoperatively), we could confirm the initial result that, while the interposition flap does not have a negative impact on complications or functionality, there is also no effect on lymphocele reduction in the long term. Additionally, these results confirm the possibility of lymphocele formation beyond the third postoperative month, which has to be borne in mind in the follow-up of these patients.

**Assessment Category**	**Score**	**Assessment Category**	**Score**	**Assessment Category**	**Score**
Comprehension Score Rater 1	5	Gunning Fog Score	19.5	Words	153
Comprehension Score Rater 2	5	SMOG Index	14.3	Complex words	43
Reading Age (years)	22.5	Coleman Liau Index	18.0	Percentage of complex words	28.1
Flesch-Kincaid Reading Ease	19.9	Automated Readability Index	16.5	Average words per sentence	21.86
Flesch-Kincaid Grade Level	15.9	Sentences	7	Average syllables per word	1.95

Lay Summary (Simple Prompt)

After prostate cancer surgery, some patients develop fluid-filled sacs called lymphoceles, which can cause discomfort or complications. Previous studies suggested that placing a peritoneal interposition flap (PIF) during surgery might help reduce these issues, but short-term results showed no clear benefit. This study followed patients from the PIANOFORTE trial for more than three years to assess whether PIF had any long-term advantages. Among 176 patients who participated in this follow-up, the overall rate of symptomatic lymphoceles remained low, and there was no significant difference between those who had a PIF and those who did not. The study confirms that PIF does not reduce lymphocele formation or improve surgical outcomes. These findings provide important insights for surgeons and patients, helping guide treatment decisions and improving the understanding of long-term risks after prostate cancer surgery.

**Assessment Category**	**Score**	**Assessment Category**	**Score**	**Assessment Category**	**Score**
Comprehension Score Rater 1	5	Gunning Fog Score	16.4	Words	138
Comprehension Score Rater 2	5	SMOG Index	12.0	Complex words	26
Reading Age (years)	20.5	Coleman Liau Index	17.1	Percentage of complex words	18.84
Flesch-Kincaid Reading Ease	38.2	Automated Readability Index	16.4	Average words per sentence	23.0
Flesch-Kincaid Grade Level	13.6	Sentences	6	Average syllables per word	1.72

Lay Summary (Extended Prompt)

After prostate cancer surgery, some patients develop fluid-filled pockets called lymphoceles, which can cause discomfort or complications. A surgical technique called a peritoneal interposition flap (PIF) has been tested to see if it helps prevent these lymphoceles, but previous studies did not follow patients long enough to understand its long-term effects. In this study, researchers followed 176 patients for more than three years after their prostate surgery to see if PIF made a difference. They found that PIF did not reduce the risk of developing lymphoceles, nor did it affect complications, urinary control, or cancer recurrence. A small number of patients developed lymphoceles even after the first few months. These findings confirm that PIF does not provide long-term benefits in preventing this issue. This study helps doctors make better decisions about surgical techniques to improve patient recovery after prostate cancer treatment.

**Assessment Category**	**Score**	**Assessment Category**	**Score**	**Assessment Category**	**Score**
Comprehension Score Rater 1	4	Gunning Fog Score	16.0	Words	144
Comprehension Score Rater 2	4	SMOG Index	11.6	Complex words	28
Reading Age (years)	19.5	Coleman Liau Index	16.4	Percentage of complex words	19.44
Flesch-Kincaid Reading Ease	41.4	Automated Readability Index	14.6	Average words per sentence	20.57
Flesch-Kincaid Grade Level	12.6	Sentences	7	Average syllables per word	1.71

Manuscript 126

Huynh LM, Swanson S, Cima S, Haddadin E, Baine M. Prostate-Specific Membrane Antigen Positron Emission Tomography/Computed Tomography-Derived Radiomic Models in Prostate Cancer Prognostication. Cancers [Internet]. 2024 May 16;16(10):1897. Available from: http://dx.doi.org/10.3390/cancers16101897

Abstract

The clinical integration of prostate membrane specific antigen (PSMA) positron emission tomography and computed tomography (PET/CT) scans represents potential for advanced data analysis techniques in prostate cancer (PC) prognostication. Among these tools is the use of radiomics, a computer-based method of extracting and quantitatively analyzing subvisual features in medical imaging. Within this context, the present review seeks to summarize the current literature on the use of PSMA PET/CT-derived radiomics in PC risk stratification. A stepwise literature search of publications from 2017 to 2023 was performed. Of 23 articles on PSMA PET/CT-derived prostate radiomics, PC diagnosis, prediction of biopsy Gleason score (GS), prediction of adverse pathology, and treatment outcomes were the primary endpoints of 4 (17.4%), 5 (21.7%), 7 (30.4%), and 7 (30.4%) studies, respectively. In predicting PC diagnosis, PSMA PET/CT-derived models performed well, with receiver operator characteristic curve area under the curve (ROC-AUC) values of 0.85-0.925. Similarly, in the prediction of biopsy and surgical pathology results, ROC-AUC values had ranges of 0.719-0.84 and 0.84-0.95, respectively. Finally, prediction of recurrence, progression, or survival following treatment was explored in nine studies, with ROC-AUC ranging 0.698-0.90. Of the 23 studies included in this review, 2 (8.7%) included external validation. While explorations of PSMA PET/CT-derived radiomic models are immature in follow-up and experience, these results represent great potential for future investigation and exploration. Prior to consideration for clinical use, however, rigorous validation in feature reproducibility and biologic validation of radiomic signatures must be prioritized.

Keywords:

artificial intelligence, personalized medicine, prostate cancer, radiomics

Original Lay Summary

The contemporary development of radiomics offers an opportune methodology for the interpretation of prostate-specific membrane antigen (PSMA) positron emission tomography/computed tomography (PET/CT). While both technologies are relatively new for consideration of clinical integration, the present exploration seeks to review current literature on their intersection. Review of twenty-three peer-reviewed articles revealed promising results for the use of PSMA PET/CT-derived radiomics in the prediction of biopsy Gleason score, adverse pathology, and treatment outcomes for prostate cancer (PC). Clinical integration of these findings, however, are limited by lack of biologic validation and reproducible methodology.

**Assessment Category**	**Score**	**Assessment Category**	**Score**	**Assessment Category**	**Score**
Comprehension Score Rater 1	5	Gunning Fog Score	23.8	Words	95
Comprehension Score Rater 2	5	SMOG Index	16.8	Complex words	34
Reading Age (years)	24.0	Coleman Liau Index	21.8	Percentage of complex words	35.79
Flesch-Kincaid Reading Ease	-7.0	Automated Readability Index	20.5	Average words per sentence	23.75
Flesch-Kincaid Grade Level	20.1	Sentences	4	Average syllables per word	2.24

<u>Lay Summary (Simple Prompt)</u>

Prostate cancer is one of the most common cancers in men, and accurate diagnosis and risk assessment are crucial for choosing the best treatment. A modern imaging technique called PSMA PET/CT helps detect prostate cancer, but advanced computer-based methods, such as radiomics, can further improve its ability to predict cancer severity and treatment outcomes. Radiomics analyzes hidden details in medical images that the human eye cannot see, potentially leading to more precise and personalized care. This study reviewed 23 research articles to evaluate how well radiomics-based models using PSMA PET/CT scans predict prostate cancer diagnosis, aggressiveness, and treatment success. The results suggest that these models perform well, but more research is needed to confirm their reliability before they can be used in routine clinical practice. If validated, this approach could significantly improve prostate cancer management by providing more tailored treatment decisions for patients.

**Assessment Category**	**Score**	**Assessment Category**	**Score**	**Assessment Category**	**Score**
Comprehension Score Rater 1	5	Gunning Fog Score	16.8	Words	145
Comprehension Score Rater 2	5	SMOG Index	12.0	Complex words	26
Reading Age (years)	21.5	Coleman Liau Index	17.9	Percentage of complex words	17.93
Flesch-Kincaid Reading Ease	27.1	Automated Readability Index	17.6	Average words per sentence	24.17
Flesch-Kincaid Grade Level	15.5	Sentences	6	Average syllables per word	1.83

Lay Summary (Extended Prompt)

Prostate cancer is a serious disease, and doctors use advanced imaging techniques to detect and predict its behavior. One promising tool is a special type of scan called PSMA PET/CT, which can provide detailed images of the prostate. Researchers are now exploring a technique called radiomics, which uses computers to analyze tiny details in these images that may not be visible to the human eye. This study reviewed research on how radiomics can help doctors better understand prostate cancer, predict its severity, and guide treatment decisions. The findings show that radiomics-based models from PSMA PET/CT scans can accurately predict important factors like cancer diagnosis, severity, and treatment outcomes. However, before these tools can be used in everyday medical practice, more research is needed to confirm their reliability. If successful, these methods could lead to more personalized and effective treatment plans for prostate cancer patients.

**Assessment Category**	**Score**	**Assessment Category**	**Score**	**Assessment Category**	**Score**
Comprehension Score Rater 1	5	Gunning Fog Score	16.0	Words	145
Comprehension Score Rater 2	5	SMOG Index	11.6	Complex words	28
Reading Age (years)	19.5	Coleman Liau Index	16.5	Percentage of complex words	19.31
Flesch-Kincaid Reading Ease	38.8	Automated Readability Index	14.8	Average words per sentence	20.71
Flesch-Kincaid Grade Level	13.0	Sentences	7	Average syllables per word	1.74

Manuscript 127

Benzaquen D, Lawrence YR, Taussky D, Zwahlen D, Oehler C, Champion A. The Crosstalk between Nerves and Cancer—A Poorly Understood Phenomenon and New Possibilities. Cancers [Internet]. 2024 May 15;16(10):1875. Available from: http://dx.doi.org/10.3390/cancers16101875

Abstract

Introduction: Crosstalk occurs between nerve and cancer cells. These interactions are important for cancer homeostasis and metabolism. Nerve cells influence the tumor microenvironment (TME) and participate in metastasis through neurogenesis, neural extension, and axonogenesis. We summarized the past and current literature on the interaction between nerves and cancer, with a special focus on pancreatic ductal adenocarcinoma (PDAC), prostate cancer (PCa), and the role of the nerve growth factor (NGF) in cancer.

Materials/methods: We reviewed PubMed and Google Scholar for the relevant literature on the relationship between nerves, neurotrophins, and cancer in general and specifically for both PCa and PDAC.

Results: The NGF helped sustain cancer cell proliferation and evade immune defense. It is a neuropeptide involved in neurogenic inflammation through the activation of several cells of the immune system by several proinflammatory cytokines. Both PCa and PDAC employ different strategies to evade immune defense. The prostate is richly innervated by both the sympathetic and parasympathetic nerves, which helps in both growth control and homeostasis. Newly formed autonomic nerve fibers grow into cancer cells and contribute to cancer initiation and progression through the activation of β-adrenergic and muscarinic cholinergic signaling. Surgical or chemical sympathectomy prevents the development of prostate cancer. Beta-blockers have a high therapeutic potential for cancer, although current clinical data have been contradictory. With a better

understanding of the beta-receptors, one could identify specific receptors that could have an effect on prostate cancer development or act as therapeutic agents.

Conclusion: The bidirectional crosstalk between the nervous system and cancer cells has emerged as a crucial regulator of cancer and its microenvironment. Denervation has been shown to be promising in vitro and in animal models. Additionally, there is a potential relationship between cancer and psychosocial biology through neurotransmitters and neurotrophins.

Keywords:
axonogenesis, cancer, nerves, tumor microenvironment

<u>Original Lay Summary</u>

There is an interaction between cancer and neural cells. This interaction was discovered as early as 1948. The first messenger of this interaction, called crosstalk, was discovered shortly after and was named the nerve growth factor (NGF). This factor influences many different organs, among them, the immune system and the nervous system. Its influence on cancer is little-known. We summarized the present knowledge on the interaction between cancer and nerves with a special focus on prostate and pancreas cancer, cancers that are richly innervated and therefore called neurotrophic. We found that this interaction plays a crucial role as a regulator of the cancer microenvironment. The surgical, chemical, and radiotherapeutical ablation of nerves has been shown to have a therapeutic effect on both cancers. There seems to be a potential relationship between cancer and psychosocial biology via neurotransmitters and neurotrophins such as the NGF. The effect of beta-blockers on cancer seems limited so far.

**Assessment Category**	**Score**	**Assessment Category**	**Score**	**Assessment Category**	**Score**
Comprehension Score Rater 1	5	Gunning Fog Score	13.2	Words	155
Comprehension Score Rater 2	5	SMOG Index	9.6	Complex words	27
Reading Age (years)	17.5	Coleman Liau Index	15.9	Percentage of complex words	17.42
Flesch-Kincaid Reading Ease	41.0	Automated Readability Index	11.7	Average words per sentence	15.5
Flesch-Kincaid Grade Level	11.4	Sentences	10	Average syllables per word	1.77

Lay Summary (Simple Prompt)

Cancer and the nervous system are more connected than previously thought. Recent research shows that nerve cells can influence how tumors grow and spread, particularly in prostate and pancreatic cancers. Nerve signals help cancer cells survive, escape the immune system, and even create new nerve pathways to support tumor growth. Our study reviews past and current research on this interaction, exploring the role of nerve-related molecules like the nerve growth factor (NGF) in cancer progression. We also examine whether blocking nerve signals, through surgery or medication like beta-blockers, could slow down tumor growth. Although some treatments show promise in lab studies and animal models, more research is needed to determine their effectiveness in humans. Understanding how nerves and cancer communicate could lead to new treatment strategies and improve cancer therapies in the future.

**Assessment Category**	**Score**	**Assessment Category**	**Score**	**Assessment Category**	**Score**
Comprehension Score Rater 1	5	Gunning Fog Score	13.3	Words	135
Comprehension Score Rater 2	5	SMOG Index	9.6	Complex words	19
Reading Age (years)	18.5	Coleman Liau Index	16.8	Percentage of complex words	14.07
Flesch-Kincaid Reading Ease	44.4	Automated Readability Index	14.3	Average words per sentence	19.29
Flesch-Kincaid Grade Level	11.9	Sentences	7	Average syllables per word	1.69

<u>Lay Summary (Extended Prompt)</u>

Cancer and the nervous system are more connected than we once thought. Nerve cells can help cancer grow and spread by changing the tumor's surroundings. This study explores how nerves and cancer interact, especially in prostate and pancreatic cancer. Scientists reviewed past research to understand how nerve signals and growth factors influence cancer. They found that nerve-related molecules help cancer cells multiply and avoid the body's immune defenses. Some studies suggest that blocking certain nerve signals could slow cancer growth, and drugs like beta-blockers might be useful, though more research is needed. Understanding this nerve-cancer connection could lead to new treatments that target how nerves help cancer develop. This research highlights the importance of looking beyond cancer cells alone and considering the nervous system as part of the disease. Future studies may bring new ways to treat cancer by stopping harmful nerve activity.

**Assessment Category**	**Score**	**Assessment Category**	**Score**	**Assessment Category**	**Score**
Comprehension Score Rater 1	5	Gunning Fog Score	10.9	Words	146
Comprehension Score Rater 2	5	SMOG Index	7.8	Complex words	16
Reading Age (years)	16.5	Coleman Liau Index	16.4	Percentage of complex words	10.96
Flesch-Kincaid Reading Ease	55.9	Automated Readability Index	12.4	Average words per sentence	16.22
Flesch-Kincaid Grade Level	9.5	Sentences	9	Average syllables per word	1.59

Manuscript 128

Sayan M, Tuac Y, Kucukcolak S, Rowan MD, Pratt GK, Aktan C, et al. Histologically Overt Stromal Response and the Risk of Progression after Radical Prostatectomy for Prostate Cancer. Cancers [Internet]. 2024 May 14;16(10):1871. Available from: http://dx.doi.org/10.3390/cancers16101871

Abstract

Purpose: Given the variable clinical course of prostate cancer and the limitations of current prognostic factors, this study was conducted to investigate the impact of a histologically overt stromal response (HOST-response) to prostate cancer on clinical outcomes after radical prostatectomy.

Methods: This retrospective analysis utilized The Cancer Genome Atlas (TCGA) to evaluate data from individuals with a confirmed diagnosis of prostate cancer who underwent radical prostatectomy and had available pathology slides. These slides were assessed for the presence of a HOST-response, similar to desmoplasia. The primary endpoint was progression-free survival (PFS). A multivariable competing risk regression analysis was used to assess whether a significant association existed between HOST-response and PFS, adjusting for known prostate cancer prognostic factors.

Results: Among the 348 patients analyzed, 166 (47.70%) demonstrated a HOST-response. After a median follow-up of 37.87 months (IQR: 21.20, 65.50), the presence of a HOST-response was significantly associated with a shorter PFS (SDHR, 2.10; 95% CI, 1.26 to 3.50; p = 0.004), after adjusting for covariates.

Conclusions: HOST-response in prostate cancer patients treated with radical prostatectomy is significantly associated with reduced PFS, suggesting a potential benefit from adjuvant therapy and highlighting the need for further investigation in a prospective randomized clinical trial.

Keywords:

HOST-response, adjuvant therapy, progression-free survival, prostate cancer, radical prostatectomy

<u>Original Lay Summary</u>

Prostate cancer is a commonly diagnosed cancer in men with varying outcomes following treatment. This study focuses on a specific feature observed in some prostate cancers called a histologically overt stromal response (HOST-response), which involves changes in the tissue surrounding the cancer cells. Our research used data from patients who had undergone surgery for prostate cancer to explore how this stromal response impacted their recovery without the cancer returning. We found that the presence of a HOST-response is linked to a higher chance of the cancer coming back sooner. These findings suggest that monitoring for this feature could help identify patients who might benefit from additional treatments after surgery, which could potentially improve their outcomes. The results of this study could guide future clinical trials aiming to refine treatment strategies for prostate cancer patients exhibiting a HOST-response.

**Assessment Category**	**Score**	**Assessment Category**	**Score**	**Assessment Category**	**Score**
Comprehension Score Rater 1	5	Gunning Fog Score	17.0	Words	140
Comprehension Score Rater 2	5	SMOG Index	12.3	Complex words	27
Reading Age (years)	21.5	Coleman Liau Index	17.2	Percentage of complex words	19.29
Flesch-Kincaid Reading Ease	33.3	Automated Readability Index	16.6	Average words per sentence	23.33
Flesch-Kincaid Grade Level	14.4	Sentences	6	Average syllables per word	1.77

Lay Summary (Simple Prompt)

Prostate cancer can follow different clinical courses, and predicting its progression remains challenging. This study explored whether a specific tissue reaction, called a histologically overt stromal response (HOST-response), can help predict outcomes after prostate removal surgery. Researchers analyzed patient data from a large cancer database, examining tissue samples for signs of this response. Among 348 patients, nearly half showed a HOST-response, and these patients had a significantly higher risk of cancer progression after surgery. The findings suggest that detecting this tissue reaction may help identify patients who could benefit from additional treatments to reduce their risk of recurrence. Understanding the role of the HOST-response could improve prostate cancer management and guide future research into personalized treatment strategies. Further clinical studies are needed to confirm these results and determine how best to integrate this information into patient care.

**Assessment Category**	**Score**	**Assessment Category**	**Score**	**Assessment Category**	**Score**
Comprehension Score Rater 1	5	Gunning Fog Score	17.7	Words	140
Comprehension Score Rater 2	5	SMOG Index	12.7	Complex words	34
Reading Age (years)	21.5	Coleman Liau Index	19.4	Percentage of complex words	24.29
Flesch-Kincaid Reading Ease	34.6	Automated Readability Index	16.7	Average words per sentence	20.0
Flesch-Kincaid Grade Level	14.8	Sentences	7	Average syllables per word	1.91

<u>Lay Summary (Extended Prompt)</u>

Prostate cancer can behave very differently from person to person, making it difficult to predict how it will progress after surgery. In this study, researchers examined whether a specific tissue reaction, called a histologically overt stromal response (HOST-response), could help doctors better understand a patient's risk of cancer coming back after prostate removal. Using data from cancer patients who had undergone surgery, they analyzed tissue samples and tracked patient outcomes. They found that nearly half of the patients showed this response, and those who did were more likely to experience cancer progression sooner. These findings suggest that detecting a HOST-response could help doctors identify patients who might need additional treatment after surgery. If confirmed in future studies, this could improve treatment decisions and help more patients receive the right care at the right time.

**Assessment Category**	**Score**	**Assessment Category**	**Score**	**Assessment Category**	**Score**
Comprehension Score Rater 1	5	Gunning Fog Score	15.8	Words	136
Comprehension Score Rater 2	5	SMOG Index	11.3	Complex words	23
Reading Age (years)	20.5	Coleman Liau Index	16.9	Percentage of complex words	16.91
Flesch-Kincaid Reading Ease	37.0	Automated Readability Index	16.1	Average words per sentence	22.67
Flesch-Kincaid Grade Level	13.7	Sentences	6	Average syllables per word	1.74

Manuscript 129

Mayer R, Turkbey B, Simone CB. Autonomous Tumor Signature Extraction Applied to Spatially Registered Bi-Parametric MRI to Predict Prostate Tumor Aggressiveness: A Pilot Study. Cancers [Internet]. 2024 May 10;16(10):1822. Available from: http://dx.doi.org/10.3390/cancers16101822

Abstract

Background: Accurate, reliable, non-invasive assessment of patients diagnosed with prostate cancer is essential for proper disease management. Quantitative assessment of multi-parametric MRI, such as through artificial intelligence or spectral/statistical approaches, can provide a non-invasive objective determination of the prostate tumor aggressiveness without side effects or potential poor sampling from needle biopsy or overdiagnosis from prostate serum antigen measurements. To simplify and expedite prostate tumor evaluation, this study examined the efficacy of autonomously extracting tumor spectral signatures for spectral/statistical algorithms for spatially registered bi-parametric MRI.

Methods: Spatially registered hypercubes were digitally constructed by resizing, translating, and cropping from the image sequences (Apparent Diffusion Coefficient (ADC), High B-value, T2) from 42 consecutive patients in the bi-parametric MRI PI-CAI dataset. Prostate cancer blobs exceeded a threshold applied to the registered set from normalizing the registered set into an image that maximizes High B-value, but minimizes the ADC and T2 images, appearing "green" in the color composite. Clinically significant blobs were selected based on size, average normalized green value, sliding window statistics within a blob, and position within the hypercube. The center of mass and maximized sliding window statistics within the blobs identified voxels associated with tumor signatures. We used correlation coefficients (R) and p-values, to evaluate the linear regression fits of the z-score and SCR (with processed covariance matrix) to tumor aggressiveness, as well as Area Under the Curves (AUC) for Receiver Operator Curves (ROC) from logistic probability fits to clinically

significant prostate cancer.

Results: The highest R (R > 0.45), AUC (>0.90), and lowest p-values (<0.01) were achieved using z-score and modified registration applied to the covariance matrix and tumor signatures selected from the "greenest" parts from the selected blob.

Conclusions: The first autonomous tumor signature applied to spatially registered bi-parametric MRI shows promise for determining prostate tumor aggressiveness.

Keywords:

Gleason score (GS), bi-parametric magnetic resonance imaging (BP-MRI), clinically significant prostate cancer, correlation coefficient, logistic probability, principal components filtering, prostate cancer, regularization, signal to clutter ratio (SCR)

<u>Original Lay Summary</u>

The proper management of prostate cancer requires accurate assessment of patients diagnosed with prostate cancer, a common and often lethal cancer. Current conventional prostate cancer diagnosis relies on: (1) needle biopsy, which can result in side effects such as hemorrhage or infection, as well as misplacement of needles resulting in evaluation inaccuracy, (2) prostate serum antigen detection that can lead to overdiagnosis, or (3) MRI interpretations using the Prostate Imaging-Reporting and Data System (PI-RADS), which is a subjective method that depends on the experience of the radiologist's interpretation of the images. Quantitative analysis of MRI can alleviate the limitations of conventional approaches. Artificial intelligence applied to MRI requires big training datasets, and its application is restricted to patients scanned under certain restricted conditions. More recently, spectral/statistical techniques, adapted from remote sensing, have been applied and tested in spatially registered multi-parametric MRI. The new spectral/statistical techniques require limited training, are flexible regarding patient scanning conditions, and perform well relative to other techniques. This study extends spectral/statistical techniques to make them more independent of the individual radiologist by autonomously finding the appropriate tumor signature within a prostate and, therefore, providing an objective, non-invasive, accurate means for determining prostate tumor aggressiveness.

**Assessment Category**	**Score**	**Assessment Category**	**Score**	**Assessment Category**	**Score**
Comprehension Score Rater 1	5	Gunning Fog Score	23.8	Words	202
Comprehension Score Rater 2	5	SMOG Index	17.4	Complex words	64
Reading Age (years)	24.0	Coleman Liau Index	20.9	Percentage of complex words	31.68

Flesch-Kincaid Reading Ease	-2.1	Automated Readability Index	22.4	Average words per sentence	28.86
Flesch-Kincaid Grade Level	20.7	Sentences	7	Average syllables per word	2.12

Lay Summary (Simple Prompt)

Prostate cancer is one of the most common cancers in men, and accurately assessing how aggressive a tumor is can help doctors make better treatment decisions. Traditional methods, like biopsies, can be invasive and sometimes miss key tumor areas. This study explores a new, non-invasive approach using MRI scans combined with an artificial intelligence-based method to identify unique tumor patterns, or "signatures," that predict how aggressive the cancer is. By analyzing MRI images from 42 patients, researchers developed a method that automatically detects and highlights suspicious areas in the prostate. The findings showed that this approach could reliably identify aggressive tumors with high accuracy. If further validated, this technique could improve prostate cancer diagnosis, reduce unnecessary biopsies, and provide doctors with a faster, more precise tool for patient care.

**Assessment Category**	**Score**	**Assessment Category**	**Score**	**Assessment Category**	**Score**
Comprehension Score Rater 1	5	Gunning Fog Score	17.3	Words	132
Comprehension Score Rater 2	5	SMOG Index	12.7	Complex words	29
Reading Age (years)	21.5	Coleman Liau Index	17.1	Percentage of complex words	21.97
Flesch-Kincaid Reading Ease	27.5	Automated Readability Index	15.9	Average words per sentence	22.0
Flesch-Kincaid Grade Level	14.9	Sentences	6	Average syllables per word	1.86

<u>Lay Summary (Extended Prompt)</u>

Prostate cancer is one of the most common cancers in men, and doctors need reliable ways to assess how aggressive a tumor is to choose the best treatment. Traditional methods, such as biopsies, can be invasive and may not always give the full picture. This study tested a new way to analyze MRI scans using advanced computer techniques to identify tumor characteristics automatically. By applying a special method to MRI images from 42 patients, researchers could detect key tumor features and predict how aggressive the cancer was. The results showed that this approach was highly accurate, offering a non-invasive way to assess prostate cancer. If further validated, this technology could help doctors diagnose and manage prostate cancer more precisely, reducing unnecessary procedures and improving patient care.

**Assessment Category**	**Score**	**Assessment Category**	**Score**	**Assessment Category**	**Score**
Comprehension Score Rater 1	5	Gunning Fog Score	16.3	Words	127
Comprehension Score Rater 2	5	SMOG Index	11.8	Complex words	25
Reading Age (years)	19.5	Coleman Liau Index	15.4	Percentage of complex words	19.69
Flesch-Kincaid Reading Ease	37.5	Automated Readability Index	14.1	Average words per sentence	21.17
Flesch-Kincaid Grade Level	13.3	Sentences	6	Average syllables per word	1.75

Manuscript 130

Talyshinskii A, Hameed BMZ, Ravinder PP, Naik N, Randhawa P, Shah M, et al. Catalyzing Precision Medicine: Artificial Intelligence Advancements in Prostate Cancer Diagnosis and Management. Cancers [Internet]. 2024 May 9;16(10):1809. Available from: http://dx.doi.org/10.3390/cancers16101809

Abstract

Background: The aim was to analyze the current state of deep learning (DL)-based prostate cancer (PCa) diagnosis with a focus on magnetic resonance (MR) prostate reconstruction; PCa detection/stratification/reconstruction; positron emission tomography/computed tomography (PET/CT); androgen deprivation therapy (ADT); prostate biopsy; associated challenges and their clinical implications.

Methods: A search of the PubMed database was conducted based on the inclusion and exclusion criteria for the use of DL methods within the abovementioned areas.

Results: A total of 784 articles were found, of which, 64 were included. Reconstruction of the prostate, the detection and stratification of prostate cancer, the reconstruction of prostate cancer, and diagnosis on PET/CT, ADT, and biopsy were analyzed in 21, 22, 6, 7, 2, and 6 studies, respectively. Among studies describing DL use for MR-based purposes, datasets with magnetic field power of 3 T, 1.5 T, and 3/1.5 T were used in 18/19/5, 0/1/0, and 3/2/1 studies, respectively, of 6/7 studies analyzing DL for PET/CT diagnosis which used data from a single institution. Among the radiotracers, [68Ga]Ga-PSMA-11, [18F]DCFPyl, and [18F]PSMA-1007 were used in 5, 1, and 1 study, respectively. Only two studies that analyzed DL in the context of DT met the inclusion criteria. Both were performed with a single-institution dataset with only manual labeling of training data. Three studies, each analyzing DL for prostate biopsy, were performed with single- and multi-institutional datasets. TeUS, TRUS, and MRI were used as input modalities in two, three, and one study, respectively.

Conclusion: DL models in prostate cancer diagnosis show promise but are not yet ready for clinical use due to variability in methods, labels, and evaluation criteria. Conducting additional research while acknowledging all the limitations outlined is crucial for reinforcing the utility and effectiveness of DL-based models in clinical settings.

Keywords:

ADT, MRI, PCa detection, PCa reconstruction, PET/CT, artificial intelligence, biopsy, deep learning, prostate cancer, prostate reconstruction

Original Lay Summary

In this paper, we look at the role of artificial intelligence (AI) advancements in prostate cancer diagnosis and management. Specifically, we focus on magnetic resonance prostate reconstruction, prostate cancer detection/stratification/reconstruction, positron emission tomography/computed tomography, androgen deprivation therapy, and prostate biopsy. A total of 64 studies were included. Our results showed that deep learning AI models in prostate cancer diagnosis show promise but are not yet ready for clinical use due to variability in methods, labels, and evaluation criteria. Conducting additional research while acknowledging the limitations is crucial for reinforcing the utility and effectiveness of AI-based models in clinical settings.

**Assessment Category**	**Score**	**Assessment Category**	**Score**	**Assessment Category**	**Score**
Comprehension Score Rater 1	4	Gunning Fog Score	20.8	Words	100
Comprehension Score Rater 2	5	SMOG Index	14.6	Complex words	32
Reading Age (years)	24.0	Coleman Liau Index	21.2	Percentage of complex words	32.0
Flesch-Kincaid Reading Ease	-1.3	Automated Readability Index	18.1	Average words per sentence	20.0
Flesch-Kincaid Grade Level	18.4	Sentences	5	Average syllables per word	2.22

Lay Summary (Simple Prompt)

Prostate cancer is a major health concern, and early and accurate diagnosis is crucial for effective treatment. Artificial intelligence, particularly deep learning, has shown promise in improving prostate cancer detection and management. This study analyzed the latest research on deep learning applications in prostate cancer, focusing on MRI-based imaging, PET/CT scans, hormone therapy, and prostate biopsies. Researchers reviewed 64 high-quality studies and found that deep learning could help reconstruct prostate images, detect and classify tumors, and assist in diagnosis. However, the methods used in these studies varied widely, making it difficult to apply them consistently in real-world clinical settings. More research is needed to standardize these technologies before they can be safely integrated into medical practice. This study highlights both the potential and the current challenges of artificial intelligence in prostate cancer care, paving the way for future advancements in precision medicine.

**Assessment Category**	**Score**	**Assessment Category**	**Score**	**Assessment Category**	**Score**
Comprehension Score Rater 1	5	Gunning Fog Score	17.7	Words	145
Comprehension Score Rater 2	5	SMOG Index	12.7	Complex words	34
Reading Age (years)	21.5	Coleman Liau Index	18.8	Percentage of complex words	23.45
Flesch-Kincaid Reading Ease	26.5	Automated Readability Index	16.6	Average words per sentence	20.71
Flesch-Kincaid Grade Level	14.7	Sentences	7	Average syllables per word	1.88

Lay Summary (Extended Prompt)

Prostate cancer is one of the most common cancers in men, and early and accurate diagnosis is crucial for effective treatment. Artificial intelligence (AI), especially deep learning (DL), has shown great promise in improving the detection and evaluation of prostate cancer using medical imaging techniques like MRI, PET/CT, and ultrasound. In this study, we reviewed recent research on how DL is being used to analyze prostate cancer, including tumor detection, grading, and guiding biopsies. We found that while DL models can assist doctors in diagnosing and assessing prostate cancer, their accuracy and reliability vary due to differences in study methods and data quality. More research is needed to refine these AI tools so they can be safely used in real-world medical settings. If improved, DL could help doctors diagnose prostate cancer more accurately and efficiently, leading to better treatment decisions and outcomes for patients.

**Assessment Category**	**Score**	**Assessment Category**	**Score**	**Assessment Category**	**Score**
Comprehension Score Rater 1	5	Gunning Fog Score	17.4	Words	145
Comprehension Score Rater 2	5	SMOG Index	12.5	Complex words	28
Reading Age (years)	20.5	Coleman Liau Index	15.7	Percentage of complex words	19.31
Flesch-Kincaid Reading Ease	31.2	Automated Readability Index	15.8	Average words per sentence	23.17
Flesch-Kincaid Grade Level	14.9	Sentences	6	Average syllables per word	1.79

Manuscript 131

Chuang HD, Lin YH, Lin CH, Lai YC, Wu CH, Hsu SM. Radiation Safety Assessment in Prostate Cancer Treatment: A Predictive Approach for I-125 Brachytherapy. Cancers [Internet]. 2024 May 7;16(10):1790. Available from: http://dx.doi.org/10.3390/cancers16101790

Abstract

This study uses Monte Carlo simulation and experimental measurements to develop a predictive model for estimating the external dose rate associated with permanent radioactive source implantation in prostate cancer patients. The objective is to estimate the accuracy of the patient's external dose rate measurement. First, I-125 radioactive sources were implanted into Mylar window water phantoms to simulate the permanent implantation of these sources in patients. Water phantom experimental measurement was combined with Monte Carlo simulation to develop predictive equations, whose performance was verified against external clinical data. The model's accuracy in predicting the external dose rate in patients with permanently implanted I-125 radioactive sources was high (R2 = 0.999). A comparative analysis of the experimental measurements and the Monte Carlo simulations revealed that the maximum discrepancy between the measured and calculated values for the water phantom was less than 5.00%. The model is practical for radiation safety assessments, enabling the evaluation of radiation exposure risks to individuals around patients with permanently implanted I-125 radioactive sources.

Keywords:

Monte Carlo, permanent implantation brachytherapy, predictive model, radiation safety assessments

<u>Original Lay Summary</u>

External dose rate measurements for I-125 permanent radioactive source implantation brachytherapy for prostate cancer requires standardized external dose rate measurements and radiation safety procedures. We developed predictive models for external dose rates to ensure compliance with radiation safety guidelines. We used Monte Carlo simulation and experimental measurements to construct an external dose rate model. The model's accuracy was validated against external dose rate measurements from clinical patients. This model facilitates the calculation and minimization of family members' radiation dose, aiding in the development of personalized radiation protection strategies.

**Assessment Category**	**Score**	**Assessment Category**	**Score**	**Assessment Category**	**Score**
Comprehension Score Rater 1	5	Gunning Fog Score	23.7	Words	89
Comprehension Score Rater 2	5	SMOG Index	15.9	Complex words	38
Reading Age (years)	24.0	Coleman Liau Index	22.8	Percentage of complex words	42.7
Flesch-Kincaid Reading Ease	-5.1	Automated Readability Index	18.3	Average words per sentence	17.8
Flesch-Kincaid Grade Level	18.4	Sentences	5	Average syllables per word	2.29

<u>Lay Summary (Simple Prompt)</u>

This study focuses on improving radiation safety for patients with prostate cancer who receive a specialized form of internal radiation therapy called I-125 brachytherapy. When tiny radioactive sources are permanently implanted in the prostate, small amounts of radiation can be emitted externally. To better predict and assess this radiation exposure, researchers developed a model using advanced computer simulations and laboratory experiments. By testing their approach with water-based models and real patient data, they found that their model was highly accurate in estimating radiation levels. The findings suggest that this method can help ensure better safety guidelines for patients and those around them, such as family members and healthcare workers. By providing a reliable way to measure radiation exposure, this study contributes to safer prostate cancer treatments and improved monitoring of radiation levels in clinical settings.

**Assessment Category**	**Score**	**Assessment Category**	**Score**	**Assessment Category**	**Score**
Comprehension Score Rater 1	4	Gunning Fog Score	19.6	Words	137
Comprehension Score Rater 2	4	SMOG Index	14.1	Complex words	36
Reading Age (years)	22.5	Coleman Liau Index	18.4	Percentage of complex words	26.28
Flesch-Kincaid Reading Ease	16.3	Automated Readability Index	17.3	Average words per sentence	22.83
Flesch-Kincaid Grade Level	16.7	Sentences	6	Average syllables per word	1.98

Lay Summary (Extended Prompt)

Radiation therapy is a common treatment for prostate cancer, but it involves implanting small radioactive seeds into the prostate, which can expose people nearby to low levels of radiation. To improve safety, this study developed a model to predict how much radiation is released outside the body. Researchers used a special type of computer simulation, called Monte Carlo simulation, along with lab experiments to measure radiation levels from these implants. They tested their model using real patient data and found it was highly accurate. The model helps doctors estimate radiation exposure with confidence, ensuring that patients and those around them remain safe. This research provides a valuable tool for improving radiation safety guidelines and protecting caregivers, family members, and healthcare workers from unnecessary radiation exposure while allowing patients to receive effective treatment.

**Assessment Category**	**Score**	**Assessment Category**	**Score**	**Assessment Category**	**Score**
Comprehension Score Rater 1	5	Gunning Fog Score	18.8	Words	132
Comprehension Score Rater 2	5	SMOG Index	13.7	Complex words	34
Reading Age (years)	22.5	Coleman Liau Index	18.2	Percentage of complex words	25.76
Flesch-Kincaid Reading Ease	18.5	Automated Readability Index	16.8	Average words per sentence	22.0
Flesch-Kincaid Grade Level	16.1	Sentences	6	Average syllables per word	1.96

Manuscript 132

Chin J, Tan YG, Lee A, Ng TK, Shi R, Tang CYL, et al. "Seeing Is Believing": Additive Utility of 68Ga-PSMA-11 PET/CT in Prostate Cancer Diagnosis. Cancers [Internet]. 2024 May 5;16(9):1777. Available from: http://dx.doi.org/10.3390/cancers16091777

Abstract

Widespread adoption of mpMRI has led to a decrease in the number of patients requiring prostate biopsies. 68Ga-PSMA-11 PET/CT has demonstrated added benefits in identifying csPCa. Integrating the use of these imaging techniques may hold promise for predicting the presence of csPCa without invasive biopsy. A retrospective analysis of 42 consecutive patients who underwent mpMRI, 68Ga-PSMA-11 PET/CT, prostatic biopsy, and radical prostatectomy (RP) was carried out. A lesion-based model (n = 122) using prostatectomy histopathology as reference standard was used to analyze the accuracy of 68Ga-PSMA-11 PET/CT, mpMRI alone, and both in combination to identify ISUP-grade group ≥ 2 lesions. 68Ga-PSMA-11 PET/CT demonstrated greater specificity and positive predictive value (PPV), with values of 73.3% (vs. 40.0%) and 90.1% (vs. 82.2%), while the mpMRI Prostate Imaging Reporting and Data System (PI-RADS) 4-5 had better sensitivity and negative predictive value (NPV): 90.2% (vs. 78.5%) and 57.1% (vs. 52.4%), respectively. When used in combination, the sensitivity, specificity, PPV, and NPV were 74.2%, 83.3%, 93.2%, and 51.0%, respectively. Subgroup analysis of PI-RADS 3, 4, and 5 lesions was carried out. For PI-RADS 3 lesions, 68Ga-PSMA-11 PET/CT demonstrated a NPV of 77.8%. For PI-RADS 4-5 lesions, 68Ga-PSMA-11 PET/CT achieved PPV values of 82.1% and 100%, respectively, with an NPV of 100% in PI-RADS 5 lesions. A combination of 68Ga-PSMA-11 PET/CT and mpMRI improved the radiological diagnosis of csPCa. This suggests that avoidance of prostate biopsy prior to RP may represent a valid option in a selected subgroup of high-risk patients with a high suspicion of csPCa on mpMRI and 68Ga-PSMA-11 PET/CT.

Keywords:

PSMA PET, biopsy, mpMRI, prostate cancer diagnosis

<u>Original Lay Summary</u>

This study investigates the effectiveness of combining two imaging techniques, multiparametric magnetic resonance imaging (mpMRI) and 68Ga-Prostate-specific membrane antigen (PSMA-11) positron emission tomography/computed tomography (PET/CT), to diagnose clinically significant prostate cancer (csPCa). While mpMRI is commonly used, it has limitations in its accuracy, requires further confirmation with prostatic biopsy. This study explores whether adding 68Ga-PSMA-11 PET/CT enhances diagnostic accuracy. The results show that the combined approach significantly improves the detection of csPCa compared to using either modality alone. Specifically, when both imaging methods are able to detect suspicious lesions, the likelihood of csPCa is high. This study suggests that, in select cases with convincing imaging results, it may be possible to forgo biopsy before surgical treatment. However, further research is needed to validate these findings and develop predictive models for accurate diagnosis without biopsy.

**Assessment Category**	**Score**	**Assessment Category**	**Score**	**Assessment Category**	**Score**
Comprehension Score Rater 1	5	Gunning Fog Score	20.9	Words	139
Comprehension Score Rater 2	5	SMOG Index	14.6	Complex words	45
Reading Age (years)	23.5	Coleman Liau Index	20.0	Percentage of complex words	32.37
Flesch-Kincaid Reading Ease	9.6	Automated Readability Index	17.1	Average words per sentence	19.86
Flesch-Kincaid Grade Level	16.9	Sentences	7	Average syllables per word	2.09

Lay Summary (Simple Prompt)

Prostate cancer diagnosis is evolving with advanced imaging techniques that may reduce the need for invasive biopsies. This study investigated whether combining two imaging methods—multiparametric MRI (mpMRI) and PSMA PET/CT—could improve the accuracy of detecting significant prostate cancer. Researchers analyzed 42 patients who underwent both scans, a biopsy, and prostate surgery. The findings showed that while mpMRI was better at identifying potential cancerous areas, PSMA PET/CT had greater accuracy in confirming cancer presence. When used together, the two methods provided a more precise diagnosis. In some high-risk cases, this approach may help avoid unnecessary biopsies, reducing patient discomfort and complications. These results suggest that integrating mpMRI and PSMA PET/CT could enhance prostate cancer detection, leading to more informed treatment decisions. Further research is needed to confirm whether biopsies can be safely omitted in carefully selected patients.

**Assessment Category**	**Score**	**Assessment Category**	**Score**	**Assessment Category**	**Score**
Comprehension Score Rater 1	5	Gunning Fog Score	17.9	Words	137
Comprehension Score Rater 2	5	SMOG Index	12.6	Complex words	38
Reading Age (years)	21.5	Coleman Liau Index	19.8	Percentage of complex words	27.74
Flesch-Kincaid Reading Ease	25.2	Automated Readability Index	15.6	Average words per sentence	17.13
Flesch-Kincaid Grade Level	14.0	Sentences	8	Average syllables per word	1.94

Lay Summary (Extended Prompt)

Prostate cancer is one of the most common cancers in men, and early detection is crucial for effective treatment. Doctors often use MRI scans to detect suspicious areas in the prostate, but this method is not always accurate, leading to unnecessary biopsies. A new type of imaging called PSMA PET/CT can help doctors identify aggressive prostate cancer more precisely. In this study, researchers analyzed 42 patients who underwent both MRI and PSMA PET/CT scans, followed by a biopsy and prostate surgery. They found that PSMA PET/CT was better at confirming cancer, while MRI was better at ruling it out. When combined, these two imaging techniques improved the accuracy of prostate cancer diagnosis. This could help reduce the need for unnecessary biopsies in men at high risk of aggressive prostate cancer, making diagnosis safer and more reliable.

**Assessment Category**	**Score**	**Assessment Category**	**Score**	**Assessment Category**	**Score**
Comprehension Score Rater 1	5	Gunning Fog Score	15.1	Words	136
Comprehension Score Rater 2	5	SMOG Index	11.0	Complex words	25
Reading Age (years)	18.5	Coleman Liau Index	14.3	Percentage of complex words	18.38
Flesch-Kincaid Reading Ease	44.7	Automated Readability Index	12.4	Average words per sentence	19.43
Flesch-Kincaid Grade Level	11.9	Sentences	7	Average syllables per word	1.68

Manuscript 133

Mikami H, Noguchi S, Akatsuka J, Hasegawa H, Obayashi K, Takeda H, et al. snRNAs from Radical Prostatectomy Specimens Have the Potential to Serve as Prognostic Factors for Clinical Recurrence after Biochemical Recurrence in Patients with High-Risk Prostate Cancer. Cancers [Internet]. 2024 May 1;16(9):1757. Available from: http://dx.doi.org/10.3390/cancers16091757

Abstract

In patients with high-risk prostate cancer (HRPC) after radical prostatectomy (RP), biochemical recurrence (BCR) increases the risk of distant metastasis. Accordingly, additional prognostic biomarkers are required to identify the subpopulation of patients with HRPC who develop clinical recurrence (CR) after BCR. The objective of this study was to identify biomarkers in formalin-fixed paraffin-embedded (FFPE) RP samples that are prognostic for CR in patients with HRPC who experience BCR after RP (post-RP BCR). First, we performed a preliminary RNA sequencing analysis to comprehensively profile RNA expression in FFPE RP samples obtained from patients with HRPC who developed CR after post-RP BCR and found that many snRNAs were very abundant in preserved FFPE samples. Subsequently, we used quantitative polymerase chain reaction (qPCR) to compare the expression levels of highly abundant snRNAs in FFPE RP samples from patients with HRPC with and without CR after post-RP BCR (21 CR patients and 46 non-CR patients who had more than 5 years of follow-up after BCR). The qPCR analysis revealed that the expression levels of snRNA RNU1-1/1-2 and RNU4-1 were significantly higher in patients with CR than in patients without CR. These snRNAs were significantly correlated with clinical recurrence-free survival (RFS) in patients with HRPC who experienced post-RP BCR. Furthermore, snRNA RNU1-1/1-2 could serve as an independent prognostic factor for clinical RFS in post-RP BCR of HRPC cases where known prognostic factors (e.g., Gleason score) cannot distinguish between CR and non-CR patients. Our findings provide new insights into the involvement of snRNAs in prostate cancer progression.

Keywords:

RNA sequencing, biochemical recurrence, clinical recurrence, formalin-fixed paraffin-embedded sample, prognostic factor, prostate cancer, radical prostatectomy, snRNA

Original Lay Summary

In patients with high-risk prostate cancer (HRPC) after radical prostatectomy, biochemical recurrence increases the risk of distant metastasis. Therefore, complementary prognostic biomarkers are required to identify the subpopulation of patients with HRPC who develop clinical recurrence after biochemical recurrence. This study was performed to identify prognostic factors for clinical recurrence in patients with HRPC who experience biochemical recurrence by conducting an analysis of the expression levels of snRNAs in formalin-fixed paraffin-embedded (FFPE) radical prostatectomy samples. The FFPE sample-derived snRNA RNU1-1/RNU1-2 could serve as an independent prognostic factor of clinical recurrence-free survival after biochemical recurrence of HRPC cases where known prognostic factors (e.g., Gleason score) cannot distinguish between patients with clinical and non-clinical recurrence. Thus, snRNAs associated with prostate cancer may assist the early detection of clinical recurrence in patients with HRPC, allowing for more tailored and restorative treatments.

**Assessment Category**	**Score**	**Assessment Category**	**Score**	**Assessment Category**	**Score**
Comprehension Score Rater 1	5	Gunning Fog Score	21.5	Words	147
Comprehension Score Rater 2	5	SMOG Index	15.1	Complex words	48
Reading Age (years)	23.5	Coleman Liau Index	20.4	Percentage of complex words	32.65
Flesch-Kincaid Reading Ease	12.9	Automated Readability Index	18.1	Average words per sentence	21.0
Flesch-Kincaid Grade Level	16.7	Sentences	7	Average syllables per word	2.04

<u>Lay Summary (Simple Prompt)</u>

Prostate cancer can return even after surgery, making it important to find better ways to predict which patients are at higher risk of disease progression. In this study, we analyzed tissue samples from patients with high-risk prostate cancer who had undergone surgery but later experienced a rise in their PSA levels, indicating a possible recurrence. Using advanced genetic techniques, we discovered that specific small RNA molecules, called snRNAs, were found at higher levels in patients whose cancer later spread. One of these snRNAs, RNU1-1/1-2, was particularly useful in predicting clinical progression, even when standard risk factors could not. Our findings suggest that measuring these snRNAs in surgical tissue may help doctors identify patients who need closer monitoring or additional treatment. This could improve prostate cancer care by allowing for more personalized and timely interventions.

**Assessment Category**	**Score**	**Assessment Category**	**Score**	**Assessment Category**	**Score**
Comprehension Score Rater 1	5	Gunning Fog Score	16.7	Words	137
Comprehension Score Rater 2	5	SMOG Index	12.0	Complex words	26
Reading Age (years)	20.5	Coleman Liau Index	16.6	Percentage of complex words	18.98
Flesch-Kincaid Reading Ease	36.7	Automated Readability Index	15.9	Average words per sentence	22.83
Flesch-Kincaid Grade Level	13.8	Sentences	6	Average syllables per word	1.74

Lay Summary (Extended Prompt)

Prostate cancer can return after surgery, increasing the risk of it spreading. Doctors need better ways to predict which patients are at higher risk of this happening. In this study, researchers analyzed small RNA molecules (snRNAs) from preserved tissue samples of patients who had surgery for high-risk prostate cancer. They compared RNA levels between patients whose cancer returned and those whose cancer did not. They found that two specific snRNAs (RNU1-1/1-2 and RNU4-1) were present at higher levels in patients whose cancer returned. One of these snRNAs (RNU1-1/1-2) was a strong predictor of cancer returning, even when other risk factors could not make this distinction. These findings suggest that snRNAs could help doctors identify high-risk patients earlier, allowing for better monitoring and treatment decisions. This research provides new insights into how prostate cancer progresses and may lead to improved patient care.

**Assessment Category**	**Score**	**Assessment Category**	**Score**	**Assessment Category**	**Score**
Comprehension Score Rater 1	5	Gunning Fog Score	12.0	Words	148
Comprehension Score Rater 2	5	SMOG Index	8.5	Complex words	17
Reading Age (years)	16.5	Coleman Liau Index	14.5	Percentage of complex words	11.49
Flesch-Kincaid Reading Ease	56.6	Automated Readability Index	12.1	Average words per sentence	18.5
Flesch-Kincaid Grade Level	10.0	Sentences	8	Average syllables per word	1.55

Manuscript 134

Smith SF, Brewer DS, Hurst R, Cooper CS. Applications of Urinary Extracellular Vesicles in the Diagnosis and Active Surveillance of Prostate Cancer. Cancers [Internet]. 2024 Apr 28;16(9):1717. Available from: http://dx.doi.org/10.3390/cancers16091717

Abstract

Prostate cancer is the most common non-cutaneous cancer among men in the UK, causing significant health and economic burdens. Diagnosis and risk prognostication can be challenging due to the genetic and clinical heterogeneity of prostate cancer as well as uncertainties in our knowledge of the underlying biology and natural history of disease development. Urinary extracellular vesicles (EVs) are microscopic, lipid bilayer defined particles released by cells that carry a variety of molecular cargoes including nucleic acids, proteins and other molecules. Urine is a plentiful source of prostate-derived EVs. In this narrative review, we summarise the evidence on the function of urinary EVs and their applications in the evolving field of prostate cancer diagnostics and active surveillance. EVs are implicated in the development of all hallmarks of prostate cancer, and this knowledge has been applied to the development of multiple diagnostic tests, which are largely based on RNA and miRNA. Common gene probes included in multi-probe tests include PCA3 and ERG, and the miRNAs miR-21 and miR-141. The next decade will likely bring further improvements in the diagnostic accuracy of biomarkers as well as insights into molecular biological mechanisms of action that can be translated into opportunities in precision uro-oncology.

Keywords:

RNA, active surveillance, diagnostics, extracellular vesicles, prostate cancer, urinary biomarkers

<u>Original Lay Summary</u>

Prostate cancer is the most common cancer among men. Diagnosis and risk stratification of prostate cancer can be challenging due to gaps in our understanding of the disease as well as the limitations of tests used in clinical practice. Extracellular vesicles are microscopic particles containing genetic material, proteins and other molecules that are released by cells. Urine contains extracellular vesicles which can originate from the prostate gland. This review addresses how extracellular vesicles are involved in the development of prostate cancer, as well as how urinary extracellular vesicles can be analysed to diagnose and monitor prostate cancer.

**Assessment Category**	**Score**	**Assessment Category**	**Score**	**Assessment Category**	**Score**
Comprehension Score Rater 1	5	Gunning Fog Score	18.9	Words	97
Comprehension Score Rater 2	5	SMOG Index	13.4	Complex words	27
Reading Age (years)	21.5	Coleman Liau Index	17.8	Percentage of complex words	27.84
Flesch-Kincaid Reading Ease	23.2	Automated Readability Index	15.2	Average words per sentence	19.4
Flesch-Kincaid Grade Level	14.8	Sentences	5	Average syllables per word	1.94

Lay Summary (Simple Prompt)

Prostate cancer is a common and serious disease affecting many men, and diagnosing it accurately can be challenging. Current tests sometimes struggle to distinguish between aggressive and slow-growing cancers, making it difficult to decide on the best treatment. This study explores the potential of tiny particles in urine, called extracellular vesicles (EVs), which are naturally released by prostate cells and contain important genetic and protein information. Scientists have discovered that these EVs could help detect prostate cancer more precisely and may also assist in monitoring men who choose active surveillance instead of immediate treatment. By analyzing EVs, researchers aim to improve diagnostic accuracy and personalize care, reducing unnecessary biopsies and treatments. In the future, EV-based tests could become an essential tool in prostate cancer management, making diagnosis and monitoring easier, less invasive, and more reliable for patients and doctors.

**Assessment Category**	**Score**	**Assessment Category**	**Score**	**Assessment Category**	**Score**
Comprehension Score Rater 1	5	Gunning Fog Score	19.9	Words	141
Comprehension Score Rater 2	5	SMOG Index	14.3	Complex words	37
Reading Age (years)	22.5	Coleman Liau Index	18.5	Percentage of complex words	26.24
Flesch-Kincaid Reading Ease	19.8	Automated Readability Index	17.7	Average words per sentence	23.5
Flesch-Kincaid Grade Level	16.3	Sentences	6	Average syllables per word	1.93

Lay Summary (Extended Prompt)

Prostate cancer is one of the most common cancers in men and can be difficult to diagnose and monitor over time. Current tests do not always provide clear answers, making it hard for doctors to decide on the best treatment. Scientists are studying tiny particles in urine, called extracellular vesicles, which come from prostate cells and carry important biological signals. These particles may help detect prostate cancer earlier and more accurately by analyzing their genetic material. In this review, we explore how urine-based tests using these vesicles could improve prostate cancer diagnosis and help doctors monitor patients without the need for repeated biopsies. As research continues, these findings may lead to better, more precise ways to detect and track prostate cancer, reducing unnecessary treatments and improving patient care. This approach could shape the future of prostate cancer management and make testing easier and more reliable.

**Assessment Category**	**Score**	**Assessment Category**	**Score**	**Assessment Category**	**Score**
Comprehension Score Rater 1	5	Gunning Fog Score	15.7	Words	146
Comprehension Score Rater 2	5	SMOG Index	11.4	Complex words	27
Reading Age (years)	19.5	Coleman Liau Index	16.3	Percentage of complex words	18.49
Flesch-Kincaid Reading Ease	36.2	Automated Readability Index	14.6	Average words per sentence	20.86
Flesch-Kincaid Grade Level	13.4	Sentences	7	Average syllables per word	1.77

Manuscript 135

Liu Z, Chiu YC, Chen Y, Huang Y. A Metastatic Cancer Expression Generator (MetGen): A Generative Contrastive Learning Framework for Metastatic Cancer Generation. Cancers [Internet]. 2024 Apr 25;16(9):1653. Available from: http://dx.doi.org/10.3390/cancers16091653

Abstract

Despite significant advances in tumor biology and clinical therapeutics, metastasis remains the primary cause of cancer-related deaths. While RNA-seq technology has been used extensively to study metastatic cancer characteristics, challenges persist in acquiring adequate transcriptomic data. To overcome this challenge, we propose MetGen, a generative contrastive learning tool based on a deep learning model. MetGen generates synthetic metastatic cancer expression profiles using primary cancer and normal tissue expression data. Our results demonstrate that MetGen generates comparable samples to actual metastatic cancer samples, and the cancer and tissue classification yields performance rates of 99.8 ± 0.2% and 95.0 ± 2.3%, respectively. A benchmark analysis suggests that the proposed model outperforms traditional generative models such as the variational autoencoder. In metastatic subtype classification, our generated samples show 97.6% predicting power compared to true metastatic samples. Additionally, we demonstrate MetGen's interpretability using metastatic prostate cancer and metastatic breast cancer. MetGen has learned highly relevant signatures in cancer, tissue, and tumor microenvironments, such as immune responses and the metastasis process, which can potentially foster a more comprehensive understanding of metastatic cancer biology. The development of MetGen represents a significant step toward the study of metastatic cancer biology by providing a generative model that identifies candidate therapeutic targets for the treatment of metastatic cancer.

Keywords:

contrastive learning, deep learning, metastatic cancer, tumor microenvironment

Original Lay Summary

Metastasis, the spread of cancer cells to other parts of the body, is the leading cause of cancer-related deaths despite medical advances. The current methods used to study metastatic cancer face challenges in gathering enough data. To tackle this issue, we developed MetGen, a deep learning model that generates metastatic gene expression files using cancer and tissue samples. Our results show that the proposed model generated samples comparable to real data. The interpretability of the model could help researchers better understand cancer metastasis and lead to the discovery of new treatments to combat metastatic cancer.

**Assessment Category**	**Score**	**Assessment Category**	**Score**	**Assessment Category**	**Score**
Comprehension Score Rater 1	4	Gunning Fog Score	14.4	Words	96
Comprehension Score Rater 2	4	SMOG Index	10.7	Complex words	17
Reading Age (years)	19.5	Coleman Liau Index	16.3	Percentage of complex words	17.7
Flesch-Kincaid Reading Ease	33.1	Automated Readability Index	13.9	Average words per sentence	19.2
Flesch-Kincaid Grade Level	13.4	Sentences	5	Average syllables per word	1.8

Lay Summary (Simple Prompt)

Cancer that spreads to other parts of the body (metastatic cancer) is the leading cause of cancer-related deaths. Scientists need detailed genetic information to study this process, but gathering such data is difficult. To help overcome this challenge, researchers developed MetGen, a smart computer program that uses artificial intelligence to generate realistic genetic profiles of metastatic cancer. By analyzing patterns in existing cancer data, MetGen can predict and simulate cancer progression with high accuracy. Tests show that its results closely match real cancer samples, outperforming older methods. This tool can help scientists better understand how cancer spreads and identify possible treatment targets. MetGen could play a key role in improving cancer research, leading to new therapies and better outcomes for patients.

**Assessment Category**	**Score**	**Assessment Category**	**Score**	**Assessment Category**	**Score**
Comprehension Score Rater 1	5	Gunning Fog Score	15.8	Words	122
Comprehension Score Rater 2	5	SMOG Index	11.4	Complex words	27
Reading Age (years)	20.5	Coleman Liau Index	18.1	Percentage of complex words	22.1
Flesch-Kincaid Reading Ease	33.8	Automated Readability Index	14.4	Average words per sentence	17.4
Flesch-Kincaid Grade Level	12.9	Sentences	7	Average syllables per word	1.8

Lay Summary (Extended Prompt)

Cancer can spread from one part of the body to another, a process called metastasis, which is the leading cause of cancer-related deaths. Scientists need large amounts of genetic data to study metastatic cancer, but collecting enough real patient samples is difficult. To help with this, we created MetGen, an artificial intelligence tool that can generate realistic genetic profiles of metastatic cancer using data from normal and primary cancer tissues. Our results show that MetGen can accurately predict cancer types and tissue origins, performing better than traditional models. It also helps researchers understand how metastatic cancer behaves, which may lead to better treatments in the future. By generating high-quality cancer data, MetGen offers a new way to study metastatic cancer biology and identify potential treatment targets, making cancer research more efficient and effective.

**Assessment Category**	**Score**	**Assessment Category**	**Score**	**Assessment Category**	**Score**
Comprehension Score Rater 1	5	Gunning Fog Score	17.6	Words	135
Comprehension Score Rater 2	5	SMOG Index	12.7	Complex words	29
Reading Age (years)	21.5	Coleman Liau Index	16.8	Percentage of complex words	21.5
Flesch-Kincaid Reading Ease	26.7	Automated Readability Index	15.9	Average words per sentence	22.5
Flesch-Kincaid Grade Level	15.1	Sentences	6	Average syllables per word	1.9

Manuscript 136

Wasinger G, Cussenot O, Compérat E. Clinical Management of Intraductal Carcinoma of the Prostate. Cancers [Internet]. 2024 Apr 25;16(9):1650. Available from: http://dx.doi.org/10.3390/cancers16091650

Abstract

Intraductal carcinoma of the prostate (IDC-P) has emerged as a distinct entity with significant clinical implications in prostate cancer (PCa) management. Despite historically being considered an extension of invasive PCa, IDC-P shows unique biological characteristics that challenge traditional diagnostic and therapeutic settings. This review explores the clinical management of IDC-P. While the diagnosis of IDC-P relies on specific morphological criteria, its detection remains challenging due to inter-observer variability. Emerging evidence underscores the association of IDC-P with aggressive disease and poor clinical outcomes across various PCa stages. However, standardized management guidelines for IDC-P are lacking. Recent studies suggest considering adjuvant and neoadjuvant therapies in specific patient cohorts to improve outcomes and tailor treatment strategies based on the IDC-P status. However, the current level of evidence regarding this is low. Moving forward, a deeper understanding of the pathogenesis of IDC-P and its interaction with conventional PCa subtypes is crucial for refining risk stratification and therapeutic interventions.

Keywords:

intraductal carcinoma, pathological risk factors, prostate cancer, treatment implication

<u>Original Lay Summary</u>

Prostate cancer is a common and challenging disease among men, prompting researchers to explore new ways to understand and manage it effectively. In this review, we focus on a specific type of prostate cancer called intraductal carcinoma (IDC-P), which has unique characteristics and implications for patient care. We aim to clarify the clinical significance of IDC-P and its impact on treatment decisions. Pathological features and molecular aspects offer insights into better risk stratification and treatment approaches for patients with IDC-P.

**Assessment Category**	**Score**	**Assessment Category**	**Score**	**Assessment Category**	**Score**
Comprehension Score Rater 1	5	Gunning Fog Score	16.5	Words	83
Comprehension Score Rater 2	5	SMOG Index	11.9	Complex words	17
Reading Age (years)	20.5	Coleman Liau Index	16.5	Percentage of complex words	20.5
Flesch-Kincaid Reading Ease	30.8	Automated Readability Index	14.8	Average words per sentence	120.8
Flesch-Kincaid Grade Level	14.1	Sentences	4	Average syllables per word	1.8

Lay Summary (Simple Prompt)

Intraductal carcinoma of the prostate (IDC-P) is a specific type of prostate cancer that behaves more aggressively than other forms of the disease. It is difficult to diagnose because it shares similarities with common prostate cancer, and doctors often disagree on how to identify it. Research has shown that IDC-P is linked to worse patient outcomes, but there are no clear guidelines on how to treat it. Some studies suggest that adding extra treatments before or after surgery might help certain patients, but more research is needed to confirm this. This review aims to summarize what is currently known about IDC-P and how it may affect treatment decisions. By improving our understanding of IDC-P, we can develop better ways to diagnose and manage it, helping doctors provide the most effective care for patients with prostate cancer.

**Assessment Category**	**Score**	**Assessment Category**	**Score**	**Assessment Category**	**Score**
Comprehension Score Rater 1	5	Gunning Fog Score	14.5	Words	140
Comprehension Score Rater 2	5	SMOG Index	10.3	Complex words	19
Reading Age (years)	18.5	Coleman Liau Index	13.2	Percentage of complex words	13.6
Flesch-Kincaid Reading Ease	49.0	Automated Readability Index	13.2	Average words per sentence	23.3
Flesch-Kincaid Grade Level	12.2	Sentences	6	Average syllables per word	1.6

Lay Summary (Extended Prompt)

Intraductal carcinoma of the prostate (IDC-P) is a rare but aggressive form of prostate cancer. Doctors often struggle to diagnose it because it looks similar to other types of prostate cancer under a microscope. Research shows that patients with IDC-P tend to have more severe disease and worse outcomes, but there are no clear treatment guidelines yet. Some studies suggest that adding extra treatments, like chemotherapy or hormone therapy, before or after surgery might help certain patients. However, more research is needed to confirm the best approach. Understanding how IDC-P develops and interacts with other prostate cancer types is important for improving diagnosis and treatment. This study reviews current knowledge about IDC-P and highlights the urgent need for better ways to detect and treat this disease. By advancing our understanding, doctors can make better treatment decisions and improve patient outcomes.

**Assessment Category**	**Score**	**Assessment Category**	**Score**	**Assessment Category**	**Score**
Comprehension Score Rater 1	5	Gunning Fog Score	12.5	Words	144
Comprehension Score Rater 2	5	SMOG Index	9.2	Complex words	20
Reading Age (years)	17.5	Coleman Liau Index	15.6	Percentage of complex words	13.9
Flesch-Kincaid Reading Ease	47.0	Automated Readability Index	12.7	Average words per sentence	18.0
Flesch-Kincaid Grade Level	11.2	Sentences	8	Average syllables per word	1.7

Manuscript 137

Donners R, Candito A, Rata M, Sharp A, Messiou C, Koh DM, et al. Inter- and Intra-Patient Repeatability of Radiomic Features from Multiparametric Whole-Body MRI in Patients with Metastatic Prostate Cancer. Cancers [Internet]. 2024 Apr 25;16(9):1647. Available from: http://dx.doi.org/10.3390/cancers16091647

Abstract

(1) Background: We assessed the test-re-test repeatability of radiomics in metastatic castration-resistant prostate cancer (mCPRC) bone disease on whole-body diffusion-weighted (DWI) and T1-weighted Dixon MRI. (2) Methods: In 10 mCRPC patients, 1.5 T MRI, including DWI and T1-weighted gradient-echo Dixon sequences, was performed twice on the same day. Apparent diffusion coefficient (ADC) and relative fat-fraction-percentage (rFF%) maps were calculated. Per study, up to 10 target bone metastases were manually delineated on DWI and Dixon images. All 106 radiomic features included in the Pyradiomics toolbox were derived for each target volume from the ADC and rFF% maps. To account for inter- and intra-patient measurement repeatability, the log-transformed individual target measurements were fitted to a hierarchical model, represented as a Bayesian network. Repeatability measurements, including the intraclass correlation coefficient (ICC), were derived. Feature ICCs were compared with mean ADC and rFF ICCs. (3) Results: A total of 65 DWI and 47 rFF% targets were analysed. There was no significant bias for any features. Pairwise correlation revealed fifteen ADC and fourteen rFF% feature sub-groups, without specific patterns between feature classes. The median intra-patient ICC was generally higher than the inter-patient ICC. Features that describe extremes in voxel values (minimum, maximum, range, skewness, and kurtosis) showed generally lower ICCs. Several mostly shape-based texture features were identified, which showed high inter- and intra-patient ICCs when compared with the mean ADC or mean rFF%, respectively. (4) Conclusions: Pyradiomics texture features of mCRPC bone metastases varied greatly in inter- and intra-patient repeatability. Several

features demonstrated good repeatability, allowing for further exploration as diagnostic parameters in mCRPC bone disease.

Keywords:

diffusion magnetic resonance imaging, neoplasm metastases, radiomics

Original Lay Summary

Prostate cancer bone metastases are a heterogonous disease with heterogeneous therapy-response, not adequately captured by one-dimensional imaging biomarker measurements. DWI and Dixon MRI radiomics analysis may tackle this shortcoming, but technical assessment of repeatability is an essential prerequisite before implementation. In this manuscript we identified whole-body MRI radiomics features in prostate cancer bone disease with good inter- and intra-patient repeatability. These features may be further explored to improve outcome predictions and therapy response assessment in prostate cancer patients.

**Assessment Category**	**Score**	**Assessment Category**	**Score**	**Assessment Category**	**Score**
Comprehension Score Rater 1	4	Gunning Fog Score	21.4	Words	82
Comprehension Score Rater 2	4	SMOG Index	15.0	Complex words	27
Reading Age (years)	24.0	Coleman Liau Index	21.7	Percentage of complex words	33.0
Flesch-Kincaid Reading Ease	-0.7	Automated Readability Index	18.8	Average words per sentence	20.5
Flesch-Kincaid Grade Level	18.5	Sentences	4	Average syllables per word	2.2

Lay Summary (Simple Prompt)

Doctors use whole-body MRI scans to examine prostate cancer that has spread to the bones. However, advanced image analysis techniques, known as radiomics, must be reliable to be useful in diagnosis and treatment planning. This study tested whether radiomic features in MRI scans are consistent when repeated in the same patient and across different patients. Researchers analyzed MRI images from ten patients by scanning them twice on the same day and measuring specific features of bone metastases. The results showed that some features remained stable, while others varied significantly. Features that describe shape were more reliable than those capturing extreme values in the images. These findings suggest that certain radiomic features could be used as dependable biomarkers to help doctors assess prostate cancer in the bones more accurately, potentially improving diagnosis and treatment decisions in the future.

**Assessment Category**	**Score**	**Assessment Category**	**Score**	**Assessment Category**	**Score**
Comprehension Score Rater 1	5	Gunning Fog Score	15.7	Words	138
Comprehension Score Rater 2	5	SMOG Index	11.4	Complex words	27
Reading Age (years)	19.5	Coleman Liau Index	17.1	Percentage of complex words	19.6
Flesch-Kincaid Reading Ease	37.9	Automated Readability Index	14.8	Average words per sentence	19.7
Flesch-Kincaid Grade Level	12.9	Sentences	7	Average syllables per word	1.8

Lay Summary (Extended Prompt)

Doctors use special MRI scans to study bone tumors in patients with advanced prostate cancer. These scans provide detailed images, but it is important to know if the measurements from these images are consistent when taken multiple times. In this study, researchers examined whether specific image-based features, called radiomic features, remain stable when scans are repeated on the same day. They analyzed MRI scans from ten patients, measuring different features of bone tumors. Some features showed strong consistency, while others varied more between and within patients. The findings suggest that certain image-based features may be useful for tracking disease progression and helping doctors make better treatment decisions. By identifying the most reliable features, this study may improve how MRI is used in prostate cancer care, making diagnoses more precise and helping to personalize treatment plans for patients with metastatic prostate cancer.

**Assessment Category**	**Score**	**Assessment Category**	**Score**	**Assessment Category**	**Score**
Comprehension Score Rater 1	5	Gunning Fog Score	13.8	Words	143
Comprehension Score Rater 2	5	SMOG Index	9.8	Complex words	20
Reading Age (years)	19.5	Coleman Liau Index	17.2	Percentage of complex words	14.0
Flesch-Kincaid Reading Ease	45.9	Automated Readability Index	15.2	Average words per sentence	20.4
Flesch-Kincaid Grade Level	11.9	Sentences	7	Average syllables per word	1.7

Manuscript 138

Mattana F, Muraglia L, Barone A, Colandrea M, Saker Diffalah Y, Provera S, et al. Prostate-Specific Membrane Antigen-Targeted Therapy in Prostate Cancer: History, Combination Therapies, Trials, and Future Perspective. Cancers [Internet]. 2024 Apr 25;16(9):1643. Available from: http://dx.doi.org/10.3390/cancers16091643

Abstract

In the last decades, the development of PET/CT radiopharmaceuticals, targeting the Prostate-Specific Membrane Antigen (PSMA), changed the management of prostate cancer (PCa) patients thanks to its higher diagnostic accuracy in comparison with conventional imaging both in staging and in recurrence. Alongside molecular imaging, PSMA was studied as a therapeutic agent targeted with various isotopes. In 2021, results from the VISION trial led to the Food and Drug Administration (FDA) approval of [177Lu]Lu-PSMA-617 as a novel therapy for metastatic castration-resistant prostate cancer (mCRPC) and set the basis for a radical change in the future perspectives of PCa treatment and the history of Nuclear Medicine. Despite these promising results, primary resistance in patients treated with single-agent [177Lu]Lu-PSMA-617 remains a real issue. Emerging trials are investigating the use of [177Lu]Lu-PSMA-617 in combination with other PCa therapies in order to cover the multiple oncologic resistance pathways and to overcome tumor heterogeneity. In this review, our aim is to retrace the history of PSMA-targeted therapy from the first preclinical studies to its future applications in PCa.

Keywords:

PSMA, lutetium, oncology, prostate cancer, radioligand therapy, targeted therapy

<u>Original Lay Summary</u>

Radioligand therapy plays a crucial role in the management of prostate cancer patients, for whom despite all available treatments, natural progression is almost inevitable. The failure of therapeutic options is likely to refer to the intrinsic tumor heterogeneity and the development of oncologic resistance pathways. To address this resistance, different trials are attempting to study the effectiveness and safety of combined therapies. This review provides a comprehensive overview of the current and future applications of radioligand therapy in prostate cancer from its initial application, moving towards future perspectives, and encompassing the main characteristics of ongoing trials related to this topic.

**Assessment Category**	**Score**	**Assessment Category**	**Score**	**Assessment Category**	**Score**
Comprehension Score Rater 1	5	Gunning Fog Score	22.0	Words	100
Comprehension Score Rater 2	5	SMOG Index	16.0	Complex words	31
Reading Age (years)	24.0	Coleman Liau Index	19.9	Percentage of complex words	31.0
Flesch-Kincaid Reading Ease	2.1	Automated Readability Index	19.6	Average words per sentence	25.0
Flesch-Kincaid Grade Level	19.2	Sentences	4	Average syllables per word	2.1

Lay Summary (Simple Prompt)

Prostate cancer is one of the most common cancers in men, and finding better ways to detect and treat it is crucial. A protein called Prostate-Specific Membrane Antigen (PSMA) is found in high amounts on prostate cancer cells, making it a useful target for both imaging and treatment. A new therapy, [177Lu]Lu-PSMA-617, was recently approved to treat advanced prostate cancer, offering new hope for patients. However, some tumors do not respond well to this treatment alone. Researchers are now exploring ways to improve its effectiveness by combining it with other therapies. This review traces the history of PSMA-targeted treatments, from early research to future possibilities. By understanding how these therapies work and how they can be improved, this study aims to help develop more effective treatments, ultimately improving outcomes for prostate cancer patients.

**Assessment Category**	**Score**	**Assessment Category**	**Score**	**Assessment Category**	**Score**
Comprehension Score Rater 1	5	Gunning Fog Score	13.4	Words	137
Comprehension Score Rater 2	5	SMOG Index	10.1	Complex words	21
Reading Age (years)	18.5	Coleman Liau Index	14.7	Percentage of complex words	15.3
Flesch-Kincaid Reading Ease	47.4	Automated Readability Index	12.8	Average words per sentence	19.6
Flesch-Kincaid Grade Level	11.5	Sentences	7	Average syllables per word	1.7

<u>Lay Summary (Extended Prompt)</u>

Prostate cancer is one of the most common cancers in men, and scientists are always looking for better ways to detect and treat it. A special protein, called Prostate-Specific Membrane Antigen (PSMA), is found in high amounts on prostate cancer cells. Doctors now use PSMA to help find cancer more accurately with advanced imaging scans. Recently, a new treatment called [177Lu]Lu-PSMA-617 was approved to target and destroy cancer cells in men with advanced prostate cancer. While this treatment has shown great promise, some cancers still resist it. Scientists are now testing whether combining this therapy with other cancer treatments could make it even more effective. This article explores the history of PSMA-targeted therapy, recent advancements, and what the future may hold for prostate cancer treatment. These findings could help doctors develop better treatments, giving hope to patients facing this disease.

**Assessment Category**	**Score**	**Assessment Category**	**Score**	**Assessment Category**	**Score**
Comprehension Score Rater 1	5	Gunning Fog Score	10.5	Words	144
Comprehension Score Rater 2	5	SMOG Index	7.8	Complex words	14
Reading Age (years)	16.5	Coleman Liau Index	14.9	Percentage of complex words	9.7
Flesch-Kincaid Reading Ease	57.0	Automated Readability Index	12.1	Average words per sentence	18.0
Flesch-Kincaid Grade Level	9.8	Sentences	8	Average syllables per word	1.6

Manuscript 139

Nguyen DT, Mahajan U, Angappulige DH, Doshi A, Mahajan NP, Mahajan K. Amino Terminal Acetylation of HOXB13 Regulates the DNA Damage Response in Prostate Cancer. Cancers [Internet]. 2024 Apr 23;16(9):1622. Available from: http://dx.doi.org/10.3390/cancers16091622

Abstract

Advanced localized prostate cancers (PC) recur despite chemotherapy, radiotherapy and/or androgen deprivation therapy. We recently reported HOXB13 lysine (K)13 acetylation as a gain-of-function modification that regulates interaction with the SWI/SNF chromatin remodeling complex and is critical for anti-androgen resistance. However, whether acetylated HOXB13 promotes PC cell survival following treatment with genotoxic agents is not known. Herein, we show that K13-acetylated HOXB13 is induced rapidly in PC cells in response to DNA damage induced by irradiation (IR). It colocalizes with the histone variant γH2AX at sites of double strand breaks (DSBs). Treatment of PCs with the Androgen Receptor (AR) antagonist Enzalutamide (ENZ) did not suppress DNA-damage-induced HOXB13 acetylation. In contrast, HOXB13 depletion or loss of acetylation overcame resistance of PC cells to ENZ and synergized with IR. HOXB13K13A mutants show diminished replication fork progression, impaired G2/M arrest with significant cell death following DNA damage. Mechanistically, we found that amino terminus regulates HOXB13 nuclear puncta formation that is essential for proper DNA damage response. Therefore, targeting HOXB13 acetylation with CBP/p300 inhibitors in combination with DNA damaging therapy may be an effective strategy to overcome anti-androgen resistance of PCs.

Keywords:

CBP/p300, DNA damage, HOXB13, acetylation, condensate, prostate cancer, radio resistance

Original Lay Summary

A significant number of prostate cancers (PC) recur in patients despite treatment with chemotherapy or radiotherapy. While HOXB13 contributes to resistance to anti-androgen treatment, its role in radio resistance is unknown. Herein, we show that HOXB13 assembles at DNA damage sites and colocalizes with γH2AX at double strand breaks despite Androgen Receptor antagonism. Functionally, ablation of HOXB13 sensitizes PC cells to either radiotherapy or anti-androgen Enzalutamide as well as combination therapies. Resistance to these agents is mediated by acetylation of HOXB13 at lysine 13, which acts as an interaction module for the Switch deficient Sucrose Nonfermenting (SWI/SNF) chromatin remodeling complex. K13-acetylated HOXB13 is required for effective DNA replication following DNA damage and for the formation of nuclear puncta. Our results reveal a hitherto unknown but critical role for HOXB13 in ensuring genomic integrity in PC.

**Assessment Category**	**Score**	**Assessment Category**	**Score**	**Assessment Category**	**Score**
Comprehension Score Rater 1	5	Gunning Fog Score	18.1	Words	136
Comprehension Score Rater 2	5	SMOG Index	13.6	Complex words	39
Reading Age (years)	21.5	Coleman Liau Index	17.6	Percentage of complex words	28.7
Flesch-Kincaid Reading Ease	20.4	Automated Readability Index	15.0	Average words per sentence	19.4
Flesch-Kincaid Grade Level	15.2	Sentences	7	Average syllables per word	2.0

Lay Summary (Simple Prompt)

Prostate cancer often returns despite treatment with chemotherapy, radiation, or hormone therapy. This study investigates how a specific protein, HOXB13, helps cancer cells survive after DNA damage. The researchers found that when prostate cancer cells are exposed to radiation, HOXB13 undergoes a chemical modification called acetylation, which allows it to interact with damaged DNA and protect the cancer cells. Even when treated with hormone-blocking drugs, this modification continues to support cancer cell survival. However, when HOXB13 is removed or its acetylation is blocked, cancer cells become more sensitive to treatment and are less able to repair DNA damage. These findings suggest that targeting HOXB13 acetylation with specific inhibitors could make prostate cancer cells more vulnerable to existing therapies, potentially improving treatment outcomes for patients with resistant disease. This research highlights a new approach to overcoming treatment resistance in advanced prostate cancer.

**Assessment Category**	**Score**	**Assessment Category**	**Score**	**Assessment Category**	**Score**
Comprehension Score Rater 1	5	Gunning Fog Score	16.6	Words	142
Comprehension Score Rater 2	5	SMOG Index	12.0	Complex words	30
Reading Age (years)	21.5	Coleman Liau Index	19.2	Percentage of complex words	21.1
Flesch-Kincaid Reading Ease	29.0	Automated Readability Index	16.7	Average words per sentence	20.3
Flesch-Kincaid Grade Level	14.3	Sentences	7	Average syllables per word	1.9

<u>Lay Summary (Extended Prompt)</u>

Prostate cancer can be hard to treat, especially when it becomes resistant to common therapies like radiation, chemotherapy, and hormone-blocking drugs. Scientists are studying a protein called HOXB13, which plays a role in helping cancer cells survive treatment. This study found that when HOXB13 undergoes a specific chemical change, called acetylation, it helps cancer cells repair DNA damage, making them more resistant to therapy. The researchers discovered that blocking this modification can weaken cancer cells, making treatments like radiation and hormone therapy work better. They also identified a potential way to stop this process using drugs that target enzymes called CBP/p300. These findings could lead to better treatments for prostate cancer, helping to overcome resistance and improve survival rates. By understanding how HOXB13 affects cancer cells, scientists may develop new strategies to make existing therapies more effective.

**Assessment Category**	**Score**	**Assessment Category**	**Score**	**Assessment Category**	**Score**
Comprehension Score Rater 1	5	Gunning Fog Score	16.0	Words	138
Comprehension Score Rater 2	5	SMOG Index	11.6	Complex words	28
Reading Age (years)	20.5	Coleman Liau Index	17.7	Percentage of complex words	20.3
Flesch-Kincaid Reading Ease	37.9	Automated Readability Index	15.3	Average words per sentence	19.7
Flesch-Kincaid Grade Level	12.9	Sentences	7	Average syllables per word	1.7

Manuscript 140

Totaro A, Scarciglia E, Marino F, Campetella M, Gandi C, Ragonese M, et al. Robot-Assisted Radical Prostatectomy Performed with the Novel Surgical Robotic Platform Hugo™ RAS: Monocentric First Series of 132 Cases Reporting Surgical, and Early Functional and Oncological Outcomes at a Tertiary Referral Robotic Center. Cancers [Internet]. 2024 Apr 22;16(8):1602. Available from: http://dx.doi.org/10.3390/cancers16081602

Abstract

Background: Robotic-assisted surgery is the gold standard for performing radical prostatectomy (RARP), with new robotic devices such as HugoTM RAS gaining prominence worldwide.

Objective: We report the surgical, perioperative, and early postoperative outcomes of RARP using HugoTM RAS.

Design, setting, and participants: Between April 2022 and October 2023, we performed 132 procedures using the Montsouris technique with a four-robotic-arm configuration in patients with biopsy-proven prostate cancer (PCa).

Outcome measures: We collected intraoperative and perioperative data during hospitalization, along with follow-up data at predefined postoperative intervals of 3 and 6 months.

Results and limitations: Lymphadenectomy was performed in 25 procedures, with a bilateral nerve-sparing technique in 33 and a monolateral nerve-sparing technique in 33 cases. The mean total surgery time was 242 (±57) min, the mean console time was 124 (±48) min, and the mean docking time was 10 (±2) min. We identified 17 system errors related to robotic arm failures, 9 robotic instrument breakdowns, and 8 significant conflicts between robotic arms. One post-operative complication was

classified as Clavien-Dindo 3b. None of the adverse events, whether singular or combined, increased the operative time. Positive margins (pR1) were found in 54 (40.9%) histological specimens, 37 (28.0%) of which were clinically significant. At 3 and 6 months post-surgery, the PSA levels were undetectable in 94.6% and 92.1% of patients, respectively. Social urinary continence was regained in 86% after 6 months. Limitations of our study include its observational monocentric case-series design and the short follow-up data for functional and oncological outcomes.

Conclusions: Our initial experience highlights the reliability of the HugoTM RAS system in performing RARP. Additionally, we also list problems and solutions found in our daily work.

Keywords:

Medtronic HugoTM RAS system, intraoperative technical data, prostate cancer, robot-assisted radical prostatectomy, robotic surgery, surgical outcomes

<u>Original Lay Summary</u>

New devices are being developed and proliferated worldwide to perform robotic surgery. The sharing of data resulting from performing a large number of procedures with the HugoTM RAS system aims to demonstrate its reliability and potential use in different scenarios. In our case series, this new device has proven to be reliable in performing robot-assisted radical prostatectomy. We list the solutions we applied to address the technical issues of the system and show that these did not have a significant impact on patients and procedures.

**Assessment Category**	**Score**	**Assessment Category**	**Score**	**Assessment Category**	**Score**
Comprehension Score Rater 1	4	Gunning Fog Score	18.4	Words	85
Comprehension Score Rater 2	4	SMOG Index	13.5	Complex words	22
Reading Age (years)	20.5	Coleman Liau Index	15.4	Percentage of complex words	25.9
Flesch-Kincaid Reading Ease	31.0	Automated Readability Index	14.2	Average words per sentence	21.3
Flesch-Kincaid Grade Level	14.2	Sentences	4	Average syllables per word	1.8

<u>Lay Summary (Simple Prompt)</u>

Prostate cancer is one of the most common cancers in men, and robotic-assisted surgery has become the preferred method for removing the prostate when treatment is necessary. Our study evaluates a new surgical robotic system, the Hugo™ RAS, to determine its effectiveness and safety in performing this procedure. We analyzed 132 surgeries at a specialized medical center, focusing on surgical success, complications, and early recovery outcomes. The results showed that the system was reliable, with good cancer control and a high rate of urinary continence recovery after six months. However, we also encountered technical challenges, such as robotic arm malfunctions, which did not significantly affect surgery times. Our findings provide valuable insights into the performance of this new robotic system and can help improve future prostate cancer surgeries using advanced robotic technology.

**Assessment Category**	**Score**	**Assessment Category**	**Score**	**Assessment Category**	**Score**
Comprehension Score Rater 1	4	Gunning Fog Score	20.3	Words	133
Comprehension Score Rater 2	5	SMOG Index	14.5	Complex words	38
Reading Age (years)	22.5	Coleman Liau Index	17.0	Percentage of complex words	28.6
Flesch-Kincaid Reading Ease	21.5	Automated Readability Index	15.9	Average words per sentence	22.2
Flesch-Kincaid Grade Level	15.8	Sentences	6	Average syllables per word	1.9

Lay Summary (Extended Prompt)

Prostate cancer is one of the most common cancers in men, and many patients need surgery to remove the prostate. Robotic-assisted surgery has become the preferred method for this procedure because it allows for high precision and smaller incisions. A new robotic system called Hugo™ RAS has been introduced, but real-world data on its use is still limited. In this study, doctors performed 132 surgeries using this system and carefully tracked surgical details, recovery times, and early results. The findings show that the system is effective and safe, with most patients recovering well and regaining bladder control within six months. However, there were some technical challenges with the robotic arms that needed adjustments. These results help doctors understand how the Hugo™ RAS system works in real surgical settings and how to improve its use in the future, ultimately benefiting prostate cancer patients worldwide.

**Assessment Category**	**Score**	**Assessment Category**	**Score**	**Assessment Category**	**Score**
Comprehension Score Rater 1	4	Gunning Fog Score	14.9	Words	145
Comprehension Score Rater 2	5	SMOG Index	10.7	Complex words	24
Reading Age (years)	18.5	Coleman Liau Index	15.1	Percentage of complex words	16.6
Flesch-Kincaid Reading Ease	45.2	Automated Readability Index	13.6	Average words per sentence	20.7
Flesch-Kincaid Grade Level	12.1	Sentences	7	Average syllables per word	1.7

Manuscript 141

Ward Grados DF, Ergun O, Miller CD, Gaburak P, Frimpong NA, Shittu O, et al. Prostate Tissue Microbiome in Patients with Prostate Cancer: A Systematic Review. Cancers [Internet]. 2024 Apr 18;16(8):1549. Available from: http://dx.doi.org/10.3390/cancers16081549

Abstract

Some researchers have speculated that the prostatic microbiome is involved in the development of prostate cancer (PCa) but there is no consensus on certain microbiota in the prostatic tissue of PCa vs. healthy controls. This systematic review aims to investigate and compare the microbiome of PCa and healthy tissue to determine the microbial association with the pathogenesis of PCa. We searched MEDLINE, Embase, and Scopus databases. Articles were screened by two independent and blinded reviewers. Literature that compared the prostatic tissue microbiome of patients with PCa with benign controls was included. We found that PCa may be associated with increased Propionibacterium acnes, the herpesviridae and papillomaviridae families, and Mycoplasma genitalium, but definitive conclusions cannot be drawn from the existing data. Challenges include the difficulty of obtaining uncontaminated tissue samples and securing tissue from healthy controls. As a result, methods are varied with many studies using cancerous and "healthy" tissue from the same prostate. The organisms chosen for each study were also highly variable, making it difficult to compare studies. These issues have led to lower confidence in our results. Overall, further work is warranted to better understand the implications of the prostatic microbiome in the pathogenesis of PCa.

Keywords:

Cutibacterium, microbiota, prostate cancer, prostate microbiome

<u>Original Lay Summary</u>

Prostate cancer is a leading cause of death among men worldwide. Some researchers have speculated that the prostatic microbiome is involved in prostatic inflammation and the pathogenesis of prostate cancer; however, there has not been consensus regarding specific organisms or their overall impact on this process. In order to synthesize the data that exists on the prostate microbiome, we performed a systematic review of the literature. In this review, we concluded that the methods that have been used to identify microbes within the prostate are highly variable and therefore do not constitute robust evidence. Further research is necessary to refine the methods used and to better understand which organisms may play a role in the development of prostate cancer.

**Assessment Category**	**Score**	**Assessment Category**	**Score**	**Assessment Category**	**Score**
Comprehension Score Rater 1	5	Gunning Fog Score	18.6	Words	119
Comprehension Score Rater 2	5	SMOG Index	13.4	Complex words	27
Reading Age (years)	21.5	Coleman Liau Index	15.9	Percentage of complex words	22.7
Flesch-Kincaid Reading Ease	30.5	Automated Readability Index	15.8	Average words per sentence	23.8
Flesch-Kincaid Grade Level	14.9	Sentences	5	Average syllables per word	1.8

Lay Summary (Simple Prompt)

Scientists are exploring whether bacteria and viruses in the prostate might play a role in the development of prostate cancer. However, there is no clear agreement on which microorganisms are specifically linked to the disease. This study systematically reviewed existing research comparing the microbiome of prostate cancer tissue with that of non-cancerous prostate tissue. While some studies suggest that certain bacteria and viruses, such as Propionibacterium acnes and human papillomavirus, may be more common in cancerous prostates, the evidence remains inconclusive. A major challenge is obtaining uncontaminated tissue samples and finding truly healthy prostate tissue for comparison. Differences in research methods and the microorganisms studied make it difficult to draw firm conclusions. More research is needed to understand whether microbes contribute to prostate cancer and whether this knowledge could lead to new ways to prevent or treat the disease.

**Assessment Category**	**Score**	**Assessment Category**	**Score**	**Assessment Category**	**Score**
Comprehension Score Rater 1	5	Gunning Fog Score	15.4	Words	140
Comprehension Score Rater 2	5	SMOG Index	11.4	Complex words	27
Reading Age (years)	20.5	Coleman Liau Index	18.6	Percentage of complex words	19.3
Flesch-Kincaid Reading Ease	28.2	Automated Readability Index	16.1	Average words per sentence	20.0
Flesch-Kincaid Grade Level	14.3	Sentences	7	Average syllables per word	1.9

Lay Summary (Extended Prompt)

Scientists are exploring whether tiny organisms, called microbes, living in the prostate may play a role in the development of prostate cancer. However, there is no clear agreement on which microbes might be linked to the disease. In this study, researchers reviewed existing scientific papers to compare the microbes found in prostate cancer tissue and healthy prostate tissue. Some studies suggest that certain bacteria and viruses, such as Propionibacterium acnes and papillomaviruses, may be more common in prostate cancer. However, differences in research methods and challenges in collecting clean tissue samples make it hard to reach firm conclusions. Many studies use tissue from the same prostate to compare "healthy" and cancerous areas, which may not provide a complete picture. More research is needed to understand if and how microbes contribute to prostate cancer, which could lead to new ways to prevent or treat the disease.

**Assessment Category**	**Score**	**Assessment Category**	**Score**	**Assessment Category**	**Score**
Comprehension Score Rater 1	5	Gunning Fog Score	13.0	Words	145
Comprehension Score Rater 2	5	SMOG Index	9.3	Complex words	18
Reading Age (years)	18.5	Coleman Liau Index	15.8	Percentage of complex words	12.4
Flesch-Kincaid Reading Ease	46.4	Automated Readability Index	14.2	Average words per sentence	20.7
Flesch-Kincaid Grade Level	11.9	Sentences	7	Average syllables per word	1.7

Manuscript 142

Gamal A, Moschovas MC, Jaber AR, Saikali S, Sandri M, Patel E, et al. Peritoneal Flap Following Lymph Node Dissection in Robotic Radical Prostatectomy: A Novel "Bunching" Technique. Cancers [Internet]. 2024 Apr 18;16(8):1547. Available from: http://dx.doi.org/10.3390/cancers16081547

Abstract

Background: Pelvic lymph node dissection (PLND) is recommended while performing robot-assisted radical prostatectomy (RARP) for patients with localized intermediate or high-risk prostate cancer. However, symptomatic lymphoceles can occur after surgery, adding significant morbidity to patients. Our objective is to describe a novel Peritoneal Bladder Flap Bunching technique (PBFB) to reduce the risk of clinically significant lymphoceles in patients undergoing RARP and PLND.

Methods: We evaluated 2267 patients who underwent RARP with PLND, dividing them into two groups: Group 1, comprising 567 patients who had the peritoneal flap (PBFB), and Group 2, comprising 1700 patients without the flap; propensity score matching carried out at a 1:3 ratio. Variables analyzed included estimated blood loss (EBL), operative time, postoperative complications, lymphocele formation, and the development of symptomatic lymphocele.

Results: The two groups exhibited similar preoperative characteristics after matching. There was no statistically significant difference in the occurrence of lymphoceles between the flap group and the non-flap group, with rates of 24% and 20.9%, respectively ($p = 0.14$). However, none of the patients in the flap group (0%) developed symptomatic lymphoceles, whereas 2.2% of patients in the non-flap group experienced symptomatic lymphoceles ($p = 0.01$).

Conclusion: We have demonstrated a modified technique for a peritoneal flap (PBFB) with the initial elimination of postoperative symptomatic lymphoceles and promising short-term outcomes.

Keywords:

lymphocele, pelvic lymph node dissection, peritoneal flap, radical prostatectomy, robot-assisted, symptomatic lymphoceles

<u>Original Lay Summary</u>

Symptomatic lymphocele can occur in 2–8% of patients following PLND in RARP, and the condition can add significant morbidities, such as fever, lower abdominal pain, deep venous thrombosis (DVT), and lower urinary tract symptoms. The literature has outlined diverse techniques for preventing the formation of lymphoceles, including ligature, clipping, or the mono/bipolar sealing of lymphatic vessels. Nevertheless, there exists ongoing controversy and debate regarding the efficacy of these varied approaches. Recent studies suggest that incorporating a peritoneal flap (PF) may reduce the risk of lymphoceles by enhancing the absorption of lymph fluid through the peritoneum. We described a novel technique for peritoneal flap creation that involves bunching the bladder peritoneum and suturing it to the periosteum of the pubic bone to secure it in place. Our technique has been shown to be effective in elimination of symptomatic lymphocele formation. The bunching technique is safe, feasible, does not add significant morbidities, and does not require a learning curve.

**Assessment Category**	**Score**	**Assessment Category**	**Score**	**Assessment Category**	**Score**
Comprehension Score Rater 1	4	Gunning Fog Score	20.2	Words	157
Comprehension Score Rater 2	4	SMOG Index	14.6	Complex words	45
Reading Age (years)	22.5	Coleman Liau Index	17.5	Percentage of complex words	28.7
Flesch-Kincaid Reading Ease	23.0	Automated Readability Index	16.5	Average words per sentence	22.4
Flesch-Kincaid Grade Level	15.6	Sentences	7	Average syllables per word	1.9

Lay Summary (Simple Prompt)

When treating prostate cancer, doctors often remove nearby lymph nodes to check for cancer spread. However, this procedure can sometimes lead to fluid buildup (lymphoceles), which may cause discomfort and require additional treatment. This study introduces a new surgical technique called the Peritoneal Bladder Flap Bunching (PBFB) method, designed to reduce the risk of problematic lymphoceles after surgery. Researchers analyzed data from over 2,200 patients who underwent robotic prostate surgery with lymph node removal, comparing those who received the PBFB technique to those who did not. While the overall rate of fluid buildup was similar in both groups, none of the patients who received the new technique developed severe symptoms, whereas 2.2% of those without the flap did. These findings suggest that this innovative approach could improve recovery and reduce complications, making prostate cancer surgery safer for patients.

**Assessment Category**	**Score**	**Assessment Category**	**Score**	**Assessment Category**	**Score**
Comprehension Score Rater 1	5	Gunning Fog Score	15.7	Words	140
Comprehension Score Rater 2	5	SMOG Index	11.6	Complex words	28
Reading Age (years)	19.5	Coleman Liau Index	16.8	Percentage of complex words	20.0
Flesch-Kincaid Reading Ease	40.9	Automated Readability Index	14.6	Average words per sentence	20.0
Flesch-Kincaid Grade Level	12.5	Sentences	7	Average syllables per word	1.7

Lay Summary (Extended Prompt)

Prostate cancer surgery often involves removing nearby lymph nodes to check for cancer spread. However, this procedure can lead to fluid buildup called lymphoceles, which may cause discomfort or require medical treatment. In this study, researchers tested a new surgical technique called the Peritoneal Bladder Flap Bunching (PBFB) method to see if it helps reduce these complications. They compared two groups of patients—one that received the new technique and one that did not. While both groups had similar overall rates of lymphoceles, none of the patients who received the PBFB technique developed serious symptoms, whereas some patients in the other group did. This suggests that the PBFB technique may help prevent painful or problematic fluid buildup after surgery. If confirmed in larger studies, this method could improve recovery and reduce complications for patients undergoing robotic prostate cancer surgery.

**Assessment Category**	**Score**	**Assessment Category**	**Score**	**Assessment Category**	**Score**
Comprehension Score Rater 1	5	Gunning Fog Score	16.0	Words	138
Comprehension Score Rater 2	5	SMOG Index	11.8	Complex words	29
Reading Age (years)	19.5	Coleman Liau Index	17.0	Percentage of complex words	21.0
Flesch-Kincaid Reading Ease	42.1	Automated Readability Index	14.7	Average words per sentence	19.7
Flesch-Kincaid Grade Level	12.3	Sentences	7	Average syllables per word	1.7

Manuscript 143

Alhajahjeh A, Al-Faouri R, Bahmad HF, Bader T, Dobbs RW, Abdulelah AA, et al. From Diabetes to Oncology: Glucagon-like Peptide-1 (GLP-1) Receptor Agonist's Dual Role in Prostate Cancer. Cancers [Internet]. 2024 Apr 18;16(8):1538. Available from: http://dx.doi.org/10.3390/cancers16081538

Abstract

Glucagon-like peptide-1 (GLP-1), an incretin hormone renowned for its role in post-meal blood sugar regulation and glucose-dependent insulin secretion, has gained attention as a novel treatment for diabetes through GLP-1 receptor agonists (GLP-1-RA). Despite their efficacy, concerns have been raised regarding the potential associations between GLP-1-RA and certain malignancies, including medullary thyroid cancer. However, evidence of its association with prostate cancer (PCa) remains inconclusive. This review delves into the intricate relationship between GLP-1-RA and PCa, exploring the mechanisms through which GLP-1-Rs may impact PCa cells. We discuss the potential pathways involving cAMP, ERK, AMPK, mTOR, and P27. Furthermore, we underscore the imperative for additional research to elucidate the impact of GLP-1-RA treatment on PCa progression, patient outcomes, and potential interactions with existing therapies. Translational studies and clinical trials are crucial for a comprehensive understanding of the role of GLP-1-RA in PCa management.

Keywords:

GLP-1 receptor, GLP-1-RA, antidiabetic, prostate cancer, signaling pathway

<u>Original Lay Summary</u>

In this review paper, we outline the role of GLP-1-RAs in potentially influencing PCa development and progression. While GLP-1-RAs show promise in inhibiting cancer cell proliferation, particularly when combined with metformin or radiotherapy, clinical data on their impact on PCa outcomes are limited. Future research should focus on dedicated trials to evaluate GLP-1-RA's role in PCa management, including its potential as a prophylactic treatment and adjunct therapy in various PCa stages.

**Assessment Category**	**Score**	**Assessment Category**	**Score**	**Assessment Category**	**Score**
Comprehension Score Rater 1	5	Gunning Fog Score	20.1	Words	77
Comprehension Score Rater 2	5	SMOG Index	14.5	Complex words	19
Reading Age (years)	22.5	Coleman Liau Index	15.2	Percentage of complex words	24.7
Flesch-Kincaid Reading Ease	22.6	Automated Readability Index	16.2	Average words per sentence	25.7
Flesch-Kincaid Grade Level	16.5	Sentences	3	Average syllables per word	1.9

<u>Lay Summary (Simple Prompt)</u>

Glucagon-like peptide-1 (GLP-1) is a natural hormone that helps control blood sugar levels, and medications that mimic its effects (called GLP-1 receptor agonists) are widely used to treat diabetes. While these drugs have shown great benefits for blood sugar control and weight management, scientists are still investigating whether they might influence cancer risk, particularly in prostate cancer. This review explores how GLP-1 receptor agonists may interact with prostate cancer cells and affect cancer growth. By examining biological pathways involved in cell growth and metabolism, we aim to clarify whether these medications have a protective or harmful effect on prostate cancer. Understanding this relationship is crucial, as many patients with diabetes or obesity may also be at risk for prostate cancer. Further research, including laboratory studies and clinical trials, is essential to determine how these treatments impact prostate cancer progression and whether they could be safely used in affected patients.

**Assessment Category**	**Score**	**Assessment Category**	**Score**	**Assessment Category**	**Score**
Comprehension Score Rater 1	5	Gunning Fog Score	17.8	Words	154
Comprehension Score Rater 2	5	SMOG Index	12.9	Complex words	30
Reading Age (years)	21.5	Coleman Liau Index	16.7	Percentage of complex words	19.5
Flesch-Kincaid Reading Ease	30.3	Automated Readability Index	17.4	Average words per sentence	25.7
Flesch-Kincaid Grade Level	15.4	Sentences	6	Average syllables per word	1.8

Lay Summary (Extended Prompt)

Scientists have discovered that a hormone called glucagon-like peptide-1 (GLP-1), which helps regulate blood sugar, may also play a role in cancer. Medications that mimic GLP-1 (GLP-1 receptor agonists) are commonly used to treat diabetes, but researchers are still investigating whether these drugs could affect prostate cancer. Some studies suggest they might impact cancer cell growth, but the evidence is unclear. This review explores how GLP-1 medications interact with prostate cancer cells and what biological pathways may be involved. Understanding these effects is important because many people with diabetes also have prostate cancer. More studies and clinical trials are needed to determine if GLP-1 drugs influence cancer progression or patient outcomes. This research could help doctors make better treatment decisions for patients with both conditions and improve future cancer therapies.

**Assessment Category**	**Score**	**Assessment Category**	**Score**	**Assessment Category**	**Score**
Comprehension Score Rater 1	5	Gunning Fog Score	14.5	Words	137
Comprehension Score Rater 2	5	SMOG Index	10.5	Complex words	23
Reading Age (years)	18.5	Coleman Liau Index	16.2	Percentage of complex words	16.8
Flesch-Kincaid Reading Ease	44.3	Automated Readability Index	14.0	Average words per sentence	19.6
Flesch-Kincaid Grade Level	11.9	Sentences	7	Average syllables per word	1.7

Manuscript 144

Einspieler H, Kluge K, Haberl D, Schatz K, Nics L, Schmitl S, et al. Assessment of PSMA Expression of Healthy Organs in Different Stages of Prostate Cancer Using [68Ga]Ga-PSMA-11-PET Examinations. Cancers [Internet]. 2024 Apr 16;16(8):1514. Available from: http://dx.doi.org/10.3390/cancers16081514

Abstract

The efficacy of radioligand therapy (RLT) targeting prostate-specific membrane antigen (PSMA) is currently being investigated for its application in patients with early-stage prostate cancer (PCa). However, little is known about PSMA expression in healthy organs in this cohort. Collectively, 202 [68Ga]Ga-PSMA-11 positron emission tomography (PET) scans from 152 patients were studied. Of these, 102 PET scans were from patients with primary PCa and hormone-sensitive biochemically recurrent PCa and 50 PET scans were from patients with metastatic castration-resistant PCa (mCRPC) before and after three cycles of [177Lu]Lu-PSMA-RLT. PSMA-standardized uptake values (SUV) were measured in multiple organs and PSMA-total tumor volume (PSMA-TTV) was determined in all cohorts. The measured PET parameters of the different cohorts were normalized to the bloodpool and compared using t- or Mann-Whitney U tests. Patients with early-stage PCa had lower PSMA-TTVs (10.39 mL vs. 462.42 mL, p p 177Lu]Lu-PSMA-RLT (462.42 mL vs. 276.29 mL, p = 0.023), no significant organ differences in PET parameters were detected. These suggest different degrees of PSMA-ligand binding among patients with different stages of PCa that could influence radiotoxicity during earlier stages of disease in different organs when PSMA-RLT is administered.

Keywords:

PSMA expression, PSMA-PET scans, mCRPC, prostate cancer, radioligand therapy

Original Lay Summary

Radioligand therapies targeting prostate-specific membrane antigen (PSMA) receptors are currently being investigated in several ongoing trials for their application in early stages of prostate cancer (PCa). The aim of this study is to identify the PSMA-ligand binding in patients with early and advanced stages of PCa by measuring changes in the PSMA uptake in various organs, including non-physiologically PSMA-expressing organs, as differences in PSMA expression could lead to unpredictable radiotoxicity in early-stage PCa patients. In addition, total tumor volume (TTV) was determined to evaluate differences in PSMA-ligand uptake related to TTV in the investigated cohorts.

**Assessment Category**	**Score**	**Assessment Category**	**Score**	**Assessment Category**	**Score**
Comprehension Score Rater 1	4	Gunning Fog Score	23.8	Words	101
Comprehension Score Rater 2	5	SMOG Index	17.2	Complex words	27
Reading Age (years)	24.0	Coleman Liau Index	17.2	Percentage of complex words	26.7
Flesch-Kincaid Reading Ease	7.7	Automated Readability Index	21.8	Average words per sentence	33.7
Flesch-Kincaid Grade Level	20.6	Sentences	3	Average syllables per word	2.0

Lay Summary (Simple Prompt)

Prostate cancer can be treated with a special type of targeted radiation therapy called PSMA radioligand therapy (PSMA-RLT), which attaches to a specific protein (PSMA) found in cancer cells. However, this protein is also present in healthy organs, which may lead to side effects. To better understand this, we analyzed PSMA levels in different organs using PET scans from 152 patients at various stages of prostate cancer. Our findings showed that patients with early-stage prostate cancer had much lower PSMA tumor levels than those with advanced cancer. Although the total PSMA tumor volume decreased after treatment, we did not find major differences in PSMA levels in healthy organs between different patient groups. These insights could help doctors optimize PSMA-RLT for patients in earlier stages of prostate cancer, potentially improving safety and reducing side effects in healthy tissues.

**Assessment Category**	**Score**	**Assessment Category**	**Score**	**Assessment Category**	**Score**
Comprehension Score Rater 1	5	Gunning Fog Score	14.8	Words	140
Comprehension Score Rater 2	5	SMOG Index	10.3	Complex words	19
Reading Age (years)	19.5	Coleman Liau Index	15.4	Percentage of complex words	13.6
Flesch-Kincaid Reading Ease	44.2	Automated Readability Index	15.2	Average words per sentence	23.3
Flesch-Kincaid Grade Level	12.9	Sentences	6	Average syllables per word	1.6

Lay Summary (Extended Prompt)

Prostate cancer is often treated with a special type of targeted radiation therapy that attaches to a protein called PSMA, which is found on cancer cells. However, PSMA is also present in healthy organs, and we do not fully understand how this might affect treatment, especially in early-stage patients. In this study, we analyzed PET scans from 152 prostate cancer patients at different stages of the disease to see how much PSMA was present in both cancerous and healthy tissues. We found that patients with early-stage prostate cancer had lower overall PSMA levels than those with advanced cancer, but the amount of PSMA in healthy organs remained similar across all stages. These findings suggest that early treatment with PSMA-targeting radiation might affect healthy organs differently than in later stages. Understanding this could help doctors improve treatment safety and effectiveness for prostate cancer patients in the future.

**Assessment Category**	**Score**	**Assessment Category**	**Score**	**Assessment Category**	**Score**
Comprehension Score Rater 1	5	Gunning Fog Score	14.5	Words	149
Comprehension Score Rater 2	5	SMOG Index	9.8	Complex words	17
Reading Age (years)	19.5	Coleman Liau Index	14.8	Percentage of complex words	11.4
Flesch-Kincaid Reading Ease	44.2	Automated Readability Index	15.5	Average words per sentence	24.8
Flesch-Kincaid Grade Level	13.3	Sentences	6	Average syllables per word	1.6

Manuscript 145

Dunne VL, Wright TC, Liberal FDCG, O'Sullivan JM, Prise KM. Synergistic Activity of DNA Damage Response Inhibitors in Combination with Radium-223 in Prostate Cancer. Cancers [Internet]. 2024 Apr 15;16(8):1510. Available from: http://dx.doi.org/10.3390/cancers16081510

Abstract

Radium-223 (223Ra) and Lutetium-177-labelled-PSMA-617 (177Lu-PSMA) are currently the only radiopharmaceutical treatments to prolong survival for patients with metastatic-castration-resistant prostate cancer (mCRPC); however, mCRPC remains an aggressive disease. Recent clinical evidence suggests patients with mutations in DNA repair genes associated with homologous recombination have a greater clinical benefit from 223Ra. In this study, we aimed to determine the utility of combining DNA damage response (DDR) inhibitors to increase the therapeutic efficacy of X-rays, or 223Ra. Radiobiological responses were characterised by in vitro assessment of clonogenic survival, repair of double strand breaks, cell cycle distribution, and apoptosis via PARP-1 cleavage. Here, we show that DDR inhibitors increase the therapeutic efficacy of both radiation qualities examined, which is associated with greater levels of residual DNA damage. Co-treatment of ATM or PARP inhibition with 223Ra increased cell cycle arrest in the G2/M phase. In comparison, combined ATR inhibition and radiation qualities caused G2/M checkpoint abrogation. Additionally, greater levels of apoptosis were observed after the combination of DDR inhibitors with 223Ra. This study identified the ATR inhibitor as the most synergistic inhibitor for both radiation qualities, supporting further pre-clinical evaluation of DDR inhibitors in combination with 223Ra for the treatment of prostate cancer.

Keywords:

ATM inhibition, ATR inhibition, DNA damage, PARP inhibition, X-rays, cell cycle checkpoints, prostate cancer, radium-223

<u>Original Lay Summary</u>

Radium-223 is used for the management of metastatic castration-resistant prostate cancer. However, the response to this treatment is poor. Targeting the DNA damage response pathway through pharmacological inhibition has the ability to enhance the radiation response. In this study, we investigated the radiosensitising effects of three different DNA damage response inhibitors targeting ATM, ATR, and PARP in combination with X-rays and radium-223 on human prostate cancer cell lines. Their effects on cell survival, DNA double-strand break repair, cell cycle distribution, and apoptosis were evaluated. We determined that these inhibitors increase the ability of X-rays and radium-223 to kill prostate cells to varying degrees. Moreover, inhibition of the DNA damage response pathways impeded the repair of radiation-induced DNA double-strand breaks and induced changes in cell cycle distribution. Altogether, our study determined DDR inhibition as a promising strategy to increase the effectiveness of treatments utilising X-rays and/or 223Ra for localised and metastatic castration-resistant prostate cancer.

**Assessment Category**	**Score**	**Assessment Category**	**Score**	**Assessment Category**	**Score**
Comprehension Score Rater 1	5	Gunning Fog Score	19.9	Words	163
Comprehension Score Rater 2	5	SMOG Index	14.3	Complex words	49
Reading Age (years)	21.5	Coleman Liau Index	17.4	Percentage of complex words	30.1
Flesch-Kincaid Reading Ease	22.1	Automated Readability Index	15.3	Average words per sentence	20.4
Flesch-Kincaid Grade Level	15.2	Sentences	8	Average syllables per word	1.9

<u>Lay Summary (Simple Prompt)</u>

Prostate cancer that has spread to other parts of the body can be difficult to treat, and current therapies often have limited effectiveness. This study explores whether combining specific drugs, called DNA damage response (DDR) inhibitors, with a radiation treatment known as Radium-223 (Ra-223) can improve outcomes. Researchers tested how these drug combinations affected prostate cancer cells in the lab, focusing on their ability to stop cancer cells from repairing DNA damage, causing them to die. The results showed that DDR inhibitors made radiation therapy more effective by increasing DNA damage and preventing cancer cells from repairing themselves. One drug, an ATR inhibitor, was the most effective at enhancing the impact of radiation. These findings suggest that combining DDR inhibitors with Ra-223 could be a promising new strategy for treating advanced prostate cancer and should be further studied in clinical trials.

**Assessment Category**	**Score**	**Assessment Category**	**Score**	**Assessment Category**	**Score**
Comprehension Score Rater 1	5	Gunning Fog Score	18.5	Words	144
Comprehension Score Rater 2	5	SMOG Index	13.5	Complex words	33
Reading Age (years)	21.5	Coleman Liau Index	15.6	Percentage of complex words	22.9
Flesch-Kincaid Reading Ease	36.2	Automated Readability Index	15.7	Average words per sentence	24.0
Flesch-Kincaid Grade Level	14.2	Sentences	6	Average syllables per word	1.7

<u>Lay Summary (Extended Prompt)</u>

Prostate cancer that has spread and no longer responds to hormone therapy is very difficult to treat. Radium-223 is a special type of radiation treatment that helps patients live longer, but it does not work for everyone. Scientists have found that patients with certain genetic changes in their cancer cells may benefit more from this treatment. In this study, researchers tested whether combining Radium-223 with drugs that block DNA repair in cancer cells could make the treatment work better. They found that these drugs made the cancer cells more sensitive to radiation, stopping them from repairing damage and making them more likely to die. One drug, called an ATR inhibitor, worked especially well. These results suggest that combining DNA repair inhibitors with Radium-223 may improve treatment for advanced prostate cancer, and further studies could help bring this approach to patients in the future.

**Assessment Category**	**Score**	**Assessment Category**	**Score**	**Assessment Category**	**Score**
Comprehension Score Rater 1	5	Gunning Fog Score	12.7	Words	146
Comprehension Score Rater 2	5	SMOG Index	9.3	Complex words	18
Reading Age (years)	17.5	Coleman Liau Index	14.2	Percentage of complex words	12.3
Flesch-Kincaid Reading Ease	55.3	Automated Readability Index	13.0	Average words per sentence	20.9
Flesch-Kincaid Grade Level	10.7	Sentences	7	Average syllables per word	1.5

Manuscript 146

Jawli A, Nabi G, Huang Z. The Performance of Different Parametric Ultrasounds in Prostate Cancer Diagnosis: Correlation with Radical Prostatectomy Specimens. Cancers [Internet]. 2024 Apr 15;16(8):1502. Available from: http://dx.doi.org/10.3390/cancers16081502

Abstract

Background: Prostate cancer is a prevalent cancer among men. Multiparametric ultrasound [mpUS] is a diagnostic instrument that uses various types of ultrasounds to diagnose it. This systematic review aims to evaluate the performance of different parametric ultrasounds in diagnosing prostate cancer by associating with radical prostatectomy specimens.

Methodology: A review was performed on various ultrasound parameters using five databases. Systematic review tools were utilized to eliminate duplicates and identify relevant results. Reviewers used the Quality Assessment of Diagnostic Accuracy Results [QUADAS-2] to evaluate the bias and applicability of the study outcomes.

Result: Between 2012 and 2023, eleven studies were conducted to evaluate the performance of the different ultrasound parametric procedures in detecting prostate cancer using grayscale TRUS, SWE, CEUS, and mpUS. The high sensitivity of these procedures was found at 55%, 88.6%, 81%, and 74%, respectively. The specificity of these procedures was found to be 93.4%, 97%, 88%, and 59%, respectively. This high sensitivity and specificity may be associated with the large lesion size. The studies revealed that the sensitivity of these procedures in diagnosing clinically significant prostate cancer was 55%, 73%, 70%, and 74%, respectively, while the specificity was 61%, 78.2%, 62%, and 59%, respectively.

Conclusions: The mpUS procedure provides high sensitivity and specificity in PCa detection, especially for clinically significant prostate cancer.

Keywords:

contrast-enhanced ultrasound, multiparametric ultrasound, prostate cancer, shear-wave elastography

<u>Original Lay Summary</u>

A systematic review assessed multiparametric ultrasound [mpUS] modalities in diagnosing prostate cancer via radical prostatectomy specimens. Between 2012 and 2023, eleven studies evaluated grayscale TRUS, SWE, CEUS, and mpUS. Sensitivity ranged from 37.7% to 55% for grayscale TRUS, 55% to 88.1% for SWE, 59% to 81% for CEUS, and 74% for mpUS, with specificities ranging accordingly. Notably, sensitivity for clinically significant prostate cancer was 55%, 73%, 70%, and 74%, respectively, with varying specificities. Quality Assessment of Diagnostic Accuracy Studies-2 [QUADAS-2] was used to evaluate bias and applicability. The review underscores the significance of mpUS modalities in prostate cancer diagnosis, highlighting their varying sensitivity and specificity in detecting both overall and clinically significant prostate cancer lesions.

**Assessment Category**	**Score**	**Assessment Category**	**Score**	**Assessment Category**	**Score**
Comprehension Score Rater 1	5	Gunning Fog Score	17.7	Words	119
Comprehension Score Rater 2	5	SMOG Index	12.6	Complex words	38
Reading Age (years)	20.5	Coleman Liau Index	16.8	Percentage of complex words	31.9
Flesch-Kincaid Reading Ease	11.9	Automated Readability Index	12.1	Average words per sentence	14.9
Flesch-Kincaid Grade Level	15.3	Sentences	8	Average syllables per word	2.1

<u>Lay Summary (Simple Prompt)</u>

Prostate cancer is one of the most common cancers in men, and early detection is essential for effective treatment. Ultrasound technology is often used to diagnose prostate cancer, but different types of ultrasound may vary in accuracy. This study reviewed multiple research articles to compare the effectiveness of different ultrasound techniques, including grayscale ultrasound, contrast-enhanced ultrasound, shear-wave elastography, and multiparametric ultrasound. By analyzing data from 11 studies, the researchers found that these techniques had varying levels of sensitivity and specificity, which measure how well they detect prostate cancer. Multiparametric ultrasound showed promising results in identifying clinically significant prostate cancer. These findings can help doctors choose the most reliable ultrasound methods for detecting prostate cancer at an early stage, leading to better treatment decisions and improved patient outcomes.

**Assessment Category**	**Score**	**Assessment Category**	**Score**	**Assessment Category**	**Score**
Comprehension Score Rater 1	4	Gunning Fog Score	20.1	Words	129
Comprehension Score Rater 2	5	SMOG Index	14.3	Complex words	37
Reading Age (years)	23.5	Coleman Liau Index	20.9	Percentage of complex words	28.7
Flesch-Kincaid Reading Ease	13.8	Automated Readability Index	18.7	Average words per sentence	21.5
Flesch-Kincaid Grade Level	16.7	Sentences	6	Average syllables per word	2.0

Lay Summary (Extended Prompt)

Prostate cancer is one of the most common cancers in men, and early detection is crucial for effective treatment. Ultrasound is a widely used imaging method to help diagnose this disease. However, different types of ultrasound may perform differently in identifying prostate cancer. This study reviewed existing research to compare how well various ultrasound techniques detect prostate cancer and whether they provide accurate results when compared to actual prostate tissue samples from surgery. The analysis included data from eleven studies conducted between 2012 and 2023. The findings show that some ultrasound methods are more sensitive in detecting cancer, while others are better at ruling it out. One approach, called multiparametric ultrasound, showed promising results in identifying clinically significant prostate cancer. These insights may help doctors choose the best imaging method for prostate cancer diagnosis, improving early detection and treatment planning for patients.

**Assessment Category**	**Score**	**Assessment Category**	**Score**	**Assessment Category**	**Score**
Comprehension Score Rater 1	5	Gunning Fog Score	16.7	Words	142
Comprehension Score Rater 2	5	SMOG Index	11.9	Complex words	34
Reading Age (years)	20.5	Coleman Liau Index	18.6	Percentage of complex words	23.9
Flesch-Kincaid Reading Ease	28.6	Automated Readability Index	14.9	Average words per sentence	17.8
Flesch-Kincaid Grade Level	13.7	Sentences	8	Average syllables per word	1.9

Manuscript 147

Lautert-Dutra W, Melo CM, Chaves LP, Sousa FC, Crozier C, Dion D, et al. Investigating the Role of SNAI1 and ZEB1 Expression in Prostate Cancer Progression and Immune Modulation of the Tumor Microenvironment. Cancers [Internet]. 2024 Apr 12;16(8):1480. Available from: http://dx.doi.org/10.3390/cancers16081480

Abstract

Prostate cancer (PCa) is an immunologically cold tumor and the molecular processes that underlie this behavior are poorly understood. In this study, we investigated a primary cohort of intermediate-risk PCa (n = 51) using two NanoString profiling panels designed to study cancer progression and immune response. We identified differentially expressed genes (DEGs) and pathways associated with biochemical recurrence (BCR) and clinical risk. Confirmatory analysis was performed using the TCGA-PRAD cohort. Noteworthy DEGs included collagens such as COL1A1, COL1A2, and COL3A1. Changes in the distribution of collagens may influence the immune activity in the tumor microenvironment (TME). In addition, immune-related DEGs such as THY1, IRF5, and HLA-DRA were also identified. Enrichment analysis highlighted pathways such as those associated with angiogenesis, TGF-beta, UV response, and EMT. Among the 39 significant DEGs, 11 (28%) were identified as EMT target genes for ZEB1 using the Harmonizome database. Elevated ZEB1 expression correlated with reduced BCR risk. Immune landscape analysis revealed that ZEB1 was associated with increased immunosuppressive cell types in the TME, such as naïve B cells and M2 macrophages. Increased expression of both ZEB1 and SNAI1 was associated with elevated immune checkpoint expression. In the future, modulation of EMT could be beneficial for overcoming immunotherapy resistance in a cold tumor, such as PCa.

Keywords:

bioinformatics, biomarkers, collagens, digital flow cytometry, extracellular matrix, gene signatures, immune checkpoint proteins, immune evasion, immunotherapy, transcriptomics

Original Lay Summary

We evaluate the downstream effects of the Epithelial-to-Mesenchymal Transition (EMT) transcription factors, ZEB1 and SNAI1, and analyze their potential significance as biomarkers for increased aggressiveness and immune response in prostate cancer (PCa). We used two commercial expression profiling panels to examine a primary PCa cohort (n = 51) and identified changes in gene expression linked to downstream pathways associated with biochemical recurrence and increased clinical risk. Genes such as COL1A1, COL1A2, and COL3A1, which are implicated in the tumor microenvironment, and immune-related genes, such as THY1, IRF5, and HLA-DRA, exhibited significant expression level changes. Enrichment analysis identified pathways associated with angiogenesis, TGF-beta, EMT, and UV response in PCa progression. Confirmatory analyses conducted using public domain data demonstrated the downstream impacts of ZEB1 and SNAI1 on pathways and immune responses, highlighting their potential influence on immune modulation in PCa. Future treatment strategies aimed at modulating EMT may enhance immune cell infiltration toward an anti-tumorigenic phenotype.

**Assessment Category**	**Score**	**Assessment Category**	**Score**	**Assessment Category**	**Score**
Comprehension Score Rater 1	5	Gunning Fog Score	22.8	Words	149
Comprehension Score Rater 2	5	SMOG Index	16.9	Complex words	52
Reading Age (years)	24.0	Coleman Liau Index	21.6	Percentage of complex words	34.9
Flesch-Kincaid Reading Ease	-2.3	Automated Readability Index	20.9	Average words per sentence	24.8

Flesch-Kincaid Grade Level	19.8	Sentences	6	Average syllables per word	2.2

Lay Summary (Simple Prompt)

Prostate cancer is often difficult to treat with immunotherapy because it does not trigger a strong immune response. In this study, we explored how certain genes, particularly SNAI1 and ZEB1, influence prostate cancer progression and affect the tumor's immune environment. By analyzing tumor samples from patients and using advanced genetic profiling techniques, we identified key genes linked to cancer recurrence and immune system activity. We found that changes in certain proteins, like collagens, may influence how immune cells behave inside the tumor. Additionally, high levels of ZEB1 and SNAI1 were associated with increased immune suppression, potentially making immunotherapy less effective. These findings suggest that targeting these genes could help make prostate cancer more responsive to immune-based treatments. Understanding how the tumor environment evolves may lead to new strategies for overcoming treatment resistance and improving patient outcomes.

**Assessment Category**	**Score**	**Assessment Category**	**Score**	**Assessment Category**	**Score**
Comprehension Score Rater 1	5	Gunning Fog Score	16.3	Words	137
Comprehension Score Rater 2	5	SMOG Index	11.8	Complex words	29
Reading Age (years)	21.5	Coleman Liau Index	19.2	Percentage of complex words	21.2
Flesch-Kincaid Reading Ease	27.7	Automated Readability Index	16.3	Average words per sentence	19.6
Flesch-Kincaid Grade Level	14.3	Sentences	7	Average syllables per word	1.9

Lay Summary (Extended Prompt)

Prostate cancer is often difficult to treat with immunotherapy because it does not trigger a strong immune response. This study explored how specific genes influence cancer growth and the immune system in prostate tumors. Researchers analyzed 51 tumor samples and confirmed their findings using a large public database. They found that certain genes related to tissue structure and immune activity may play a role in cancer progression. In particular, two genes, ZEB1 and SNAI1, were linked to changes in immune responses, including increased activity of immune-suppressing cells. Higher levels of ZEB1 were associated with a lower risk of cancer returning. These findings suggest that targeting these genes could help make prostate cancer more responsive to immunotherapy. Future treatments could focus on modifying these genetic pathways to improve patient outcomes. This research may pave the way for better therapies in prostate cancer.

**Assessment Category**	**Score**	**Assessment Category**	**Score**	**Assessment Category**	**Score**
Comprehension Score Rater 1	5	Gunning Fog Score	12.8	Words	142
Comprehension Score Rater 2	5	SMOG Index	9.3	Complex words	23
Reading Age (years)	17.5	Coleman Liau Index	16.6	Percentage of complex words	16.2
Flesch-Kincaid Reading Ease	44.3	Automated Readability Index	12.4	Average words per sentence	15.8
Flesch-Kincaid Grade Level	11.0	Sentences	9	Average syllables per word	1.7

Manuscript 148

Sawayanagi S, Yamashita H, Ogita M, Kawai T, Sato Y, Kume H. In Curative Stereotactic Body Radiation Therapy for Prostate Cancer, There Is a High Possibility That 45 Gy in Five Fractions Will Not Be Tolerated without a Hydrogel Spacer. Cancers [Internet]. 2024 Apr 11;16(8):1472. Available from: http://dx.doi.org/10.3390/cancers16081472

Abstract

The purpose of this study was to determine the maximum tolerated dose (MTD) for stereotactic body radiation therapy (SBRT) in the treatment of non-metastatic prostate cancer. This study was a phase 1 dose escalation trial conducted in Japan. Patients with histologically proven prostate cancer without lymph nodes or distant metastases were enrolled. The prescribed doses were 42.5, 45, or 47.5 Gy in five fractions. Dose-limiting toxicity (DLT) was defined as grade (G) 3+ gastrointestinal or genitourinary toxicity within 180 days after SBRT completion, and a 6 plus 6 design was used as the method of dose escalation. A total of 16 patients were enrolled, with 6 in the 42.5 Gy group and 10 in the 45 Gy group. No DLT was observed in the 42.5 Gy group. In the 45 Gy group, one patient experienced G3 rectal hemorrhage, and another had G4 rectal perforation, leading to the determination of 42.5 Gy as the MTD. None of the patients experienced biochemical recurrence or death during the follow-up period. We concluded that SBRT for non-metastatic prostate cancer at 42.5 Gy in five fractions could be safely performed, but a total dose of 45 Gy increased severe toxicity.

Keywords:

dose escalation, phase 1 study, prostate cancer, stereotactic body radiation therapy

Original Lay Summary

While stereotactic body radiation therapy (SBRT) has become increasingly used for the treatment of non-metastatic prostate cancer since its insurance coverage in Japan in 2016, optimal dose fractionation remains undetermined. This study was a phase 1 dose escalation trial of SBRT, aiming to assess the maximum tolerated dose (MTD) of SBRT using five fractions. Patients were planned to receive SBRT at doses of 42.5, 45, or 47.5 Gy, with toxicity as the primary endpoint. No dose-limiting toxicities were observed at 42.5 Gy, and one patient experienced a grade 4 rectal perforation at 45 Gy, leading to the determination of 42.5 Gy as the MTD. There were no deaths or biochemical recurrences during the follow-up period. This study underscores the need for further trials to ascertain the optimal SBRT dose fractionation, balancing efficacy and safety for non-metastatic prostate cancer treatment.

**Assessment Category**	**Score**	**Assessment Category**	**Score**	**Assessment Category**	**Score**
Comprehension Score Rater 1	3	Gunning Fog Score	13.8	Words	147
Comprehension Score Rater 2	4	SMOG Index	9.9	Complex words	29
Reading Age (years)	16.5	Coleman Liau Index	12.7	Percentage of complex words	19.7
Flesch-Kincaid Reading Ease	46.9	Automated Readability Index	8.8	Average words per sentence	14.7
Flesch-Kincaid Grade Level	10.4	Sentences	10	Average syllables per word	1.7

<u>Lay Summary (Simple Prompt)</u>

Prostate cancer is often treated with a precise type of radiation therapy called stereotactic body radiation therapy (SBRT). However, finding the safest and most effective radiation dose is crucial to avoid severe side effects. This study tested different radiation doses to determine the maximum amount that patients can tolerate without serious complications. Researchers treated 16 men with prostate cancer using SBRT, delivering either 42.5 or 45 Gy in five sessions. While the lower dose was well tolerated, the higher dose caused severe rectal side effects in two patients, including one who required surgery. As a result, the study concluded that 42.5 Gy is the safest option when no protective spacer is used between the prostate and the rectum. These findings are important for improving prostate cancer treatment, ensuring effective radiation therapy while minimizing risks for patients.

**Assessment Category**	**Score**	**Assessment Category**	**Score**	**Assessment Category**	**Score**
Comprehension Score Rater 1	4	Gunning Fog Score	14.5	Words	138
Comprehension Score Rater 2	4	SMOG Index	10.4	Complex words	29
Reading Age (years)	18.5	Coleman Liau Index	15.6	Percentage of complex words	21.0
Flesch-Kincaid Reading Ease	39.2	Automated Readability Index	11.3	Average words per sentence	15.3
Flesch-Kincaid Grade Level	11.6	Sentences	9	Average syllables per word	1.8

Lay Summary (Extended Prompt)

Prostate cancer is often treated with a special type of radiation therapy called stereotactic body radiation therapy (SBRT), which delivers high doses of radiation in a few sessions. This study aimed to find the highest safe radiation dose that does not cause serious side effects. Researchers tested different dose levels on men with prostate cancer that had not spread. They found that while 42.5 Gy (a unit of radiation) was well tolerated, 45 Gy led to severe complications, including serious rectal bleeding and damage. Based on these results, 42.5 Gy in five treatments was identified as the safest option. This finding is important because it helps doctors choose the right radiation dose to effectively treat prostate cancer while reducing the risk of harmful side effects. The study also suggests that using a protective gel (hydrogel spacer) may be needed for higher doses.

**Assessment Category**	**Score**	**Assessment Category**	**Score**	**Assessment Category**	**Score**
Comprehension Score Rater 1	4	Gunning Fog Score	12.8	Words	144
Comprehension Score Rater 2	4	SMOG Index	9.3	Complex words	23
Reading Age (years)	16.5	Coleman Liau Index	13.4	Percentage of complex words	16.0
Flesch-Kincaid Reading Ease	51.4	Automated Readability Index	10.0	Average words per sentence	16.0
Flesch-Kincaid Grade Level	10.1	Sentences	9	Average syllables per word	1.7

Manuscript 149

Gamallat Y, Felipe Lima J, Seyedi S, Li Q, Rokne JG, Alhajj R, et al. Exploring The Prognostic Significance of SET-Domain Containing 2 (SETD2) Expression in Advanced and Castrate-Resistant Prostate Cancer. Cancers [Internet]. 2024 Apr 8;16(7):1436. Available from: http://dx.doi.org/10.3390/cancers16071436

Abstract

SET-domain containing 2 (SETD2) is a histone methyltransferase and an epigenetic modifier with oncogenic functionality. In the current study, we investigated the potential prognostic role of SETD2 in prostate cancer. A cohort of 202 patients' samples was assembled on tissue microarrays (TMAs) containing incidental, advanced, and castrate-resistant CRPCa cases. Our data showed significant elevated SETD2 expression in advanced and castrate-resistant disease (CRPCa) compared to incidental cases (2.53 ± 0.58 and 2.21 ± 0.63 vs. 1.9 ± 0.68; p p = 0.003, respectively). Overall, high SETD2 expression was found to be considered high risk and was significantly associated with poor prognosis and worse overall survival (OS) (HR 1.80; 95% CI: 1.28-2.53, p = 0.001) and lower cause specific survival (CSS) (HR 3.14; 95% CI: 1.94-5.08, p p = 0.008) and unfavorable CSS (HR 3.74; 95% CI: 1.67-8.34, p = 0.001). Additionally, high SETD2 intensity with ERG positive expression showed worse prognosis for both OS (HR 1.99, 95% CI 0.87-4.59; p = 0.015) and CSS (HR 2.14, 95% CI 0.98-4.68, p = 0.058). We also investigated the protein expression database TCPA, and our results showed that high SETD2 expression is associated with a poor prognosis. Finally, we performed TCGA PRAD gene set enrichment analysis (GSEA) data for SETD2 overexpression, and our data revealed a potential association with pathways involved in tumor progression such as the AMPK signaling pathway, the cAMP signaling pathway, and the PI3K-Akt signaling pathway, which are potentially associated with tumor progression, chemoresistance, and a poor prognosis.

Keywords:

ERG, PTEN, SETD2, cause specific survival, overall survival, prostate cancer

<u>Original Lay Summary</u>

SETD2, a histone methyltransferase and epigenetic modifier, and SETD2 protein expression were explored in a prostate cancer non-surgical cohort of 202 cases. Notably, SETD2 showed higher intensity in advanced and castrate-resistant disease compared to incidental cases. Moreover, elevated SETD2 expression is significantly associated with poorer prognosis, lower overall survival (OS), and decreased cancer-specific survival (CSS). High-risk SETD2 combined with PTEN loss or ERG positivity improved the prognostication for these outcomes. Additionally, the TCPA protein database and TCGA PRAD GSEA implicated SETD2 in pathways linked to tumor progression, chemoresistance, and adverse prognosis, including the AMPK, cAMP, and PI3K-Akt signaling pathways.

**Assessment Category**	**Score**	**Assessment Category**	**Score**	**Assessment Category**	**Score**
Comprehension Score Rater 1	5	Gunning Fog Score	19.5	Words	104
Comprehension Score Rater 2	5	SMOG Index	13.9	Complex words	29
Reading Age (years)	22.5	Coleman Liau Index	19.3	Percentage of complex words	27.9
Flesch-Kincaid Reading Ease	19.0	Automated Readability Index	17.0	Average words per sentence	20.8
Flesch-Kincaid Grade Level	15.8	Sentences	5	Average syllables per word	2.0

Lay Summary (Simple Prompt)

Prostate cancer can become more aggressive over time, especially in advanced stages or when it stops responding to standard treatments. In this study, we explored whether a protein called SETD2 plays a role in predicting disease severity and patient outcomes. We analyzed prostate cancer samples from 202 patients and found that higher levels of SETD2 were linked to more aggressive disease and poorer survival rates. Additionally, patients with both high SETD2 levels and another protein marker, ERG, had an even worse prognosis. To further support our findings, we examined large-scale genetic databases, which confirmed that SETD2 overexpression is associated with pathways that promote cancer growth and resistance to therapy. These findings suggest that SETD2 could serve as a potential marker to identify high-risk prostate cancer patients early, helping doctors refine treatment strategies and improve patient care.

**Assessment Category**	**Score**	**Assessment Category**	**Score**	**Assessment Category**	**Score**
Comprehension Score Rater 1	5	Gunning Fog Score	15.3	Words	138
Comprehension Score Rater 2	5	SMOG Index	10.8	Complex words	21
Reading Age (years)	20.5	Coleman Liau Index	16.8	Percentage of complex words	15.2
Flesch-Kincaid Reading Ease	39.4	Automated Readability Index	16.1	Average words per sentence	23.0
Flesch-Kincaid Grade Level	13.5	Sentences	6	Average syllables per word	1.7

Lay Summary (Extended Prompt)

Prostate cancer is one of the most common cancers in men, but not all cases behave the same way. Some become aggressive and resist treatment, making them harder to manage. In this study, we looked at a protein called SETD2, which plays a role in how cancer cells grow and survive. By analyzing tissue samples from 202 prostate cancer patients, we found that higher levels of SETD2 were linked to more advanced disease and a worse prognosis. Patients with high SETD2 levels had a shorter survival time compared to those with lower levels. Our findings suggest that SETD2 could be used as a marker to help doctors predict which patients may develop more aggressive prostate cancer. This research may lead to better ways to identify high-risk patients and develop more targeted treatments in the future.

**Assessment Category**	**Score**	**Assessment Category**	**Score**	**Assessment Category**	**Score**
Comprehension Score Rater 1	5	Gunning Fog Score	10.4	Words	136
Comprehension Score Rater 2	5	SMOG Index	6.7	Complex words	9
Reading Age (years)	15.5	Coleman Liau Index	12.2	Percentage of complex words	6.6
Flesch-Kincaid Reading Ease	65.2	Automated Readability Index	10.7	Average words per sentence	19.4
Flesch-Kincaid Grade Level	9.0	Sentences	7	Average syllables per word	1.4

Manuscript 150

Lang J, McClure TD, Margolis DJA. MRI–Ultrasound Fused Approach for Prostate Biopsy—How It Is Performed. Cancers [Internet]. 2024 Apr 7;16(7):1424. Available from: http://dx.doi.org/10.3390/cancers16071424

Abstract

The use of MRI-ultrasound image fusion targeted biopsy of the prostate in the face of an elevated serum PSA is now recommended by multiple societies, and results in improved detection of clinically significant cancer and, potentially, decreased detection of indolent disease. This combines the excellent sensitivity of MRI for clinically significant prostate cancer and the real-time biopsy guidance and confirmation of ultrasound. Both transperineal and transrectal approaches can be implemented using cognitive fusion, mechanical fusion with an articulated arm and electromagnetic registration, or pure software registration. The performance has been shown comparable to in-bore MRI biopsy performance. However, a number of factors influence the performance of this technique, including the quality and interpretation of the MRI, the approach used for biopsy, and experience of the practitioner, with most studies showing comparable performance of MRI-ultrasound fusion to in-bore targeted biopsy. Future improvements including artificial intelligence promise to refine the performance of all approaches.

Keywords:

MRI, biopsy, cancer, prostate

<u>Original Lay Summary</u>

Prostate cancer screening has traditionally been accomplished by a blood test for prostate serum antigen (PSA) followed by biopsy. MRI is very accurate at finding cancer, but ultrasound provides real-time guidance for biopsy. Using magnetic resonance imaging (MRI) to guide the biopsy under ultrasound improves detection and, in the case that only non-aggressive cancer is found, confidence in avoiding treating cancers that are unlikely to be deadly. Different approaches can be used with many different MRI–ultrasound fusion techniques, including "cognitive" fusion using only the practitioner's reading of the scans, but also software fusion using mechanical or even pure software-aided matching of the information from MRI and ultrasound.

**Assessment Category**	**Score**	**Assessment Category**	**Score**	**Assessment Category**	**Score**
Comprehension Score Rater 1	3	Gunning Fog Score	20.1	Words	110
Comprehension Score Rater 2	3	SMOG Index	15.0	Complex words	27
Reading Age (years)	23.5	Coleman Liau Index	17.2	Percentage of complex words	24.6
Flesch-Kincaid Reading Ease	23.6	Automated Readability Index	18.7	Average words per sentence	27.5
Flesch-Kincaid Grade Level	16.8	Sentences	4	Average syllables per word	1.8

Lay Summary (Simple Prompt)

Prostate cancer is one of the most common cancers in men, and early detection is essential for effective treatment. Doctors often check for prostate cancer using a blood test called PSA, but this test alone is not always reliable. This study focuses on a more advanced method that combines MRI and ultrasound to guide prostate biopsies more accurately. By using MRI to detect suspicious areas and ultrasound to help doctors take precise tissue samples, this approach improves the chances of finding aggressive cancer while reducing unnecessary biopsies. There are different ways to perform this procedure, and factors such as MRI quality and doctor experience can affect the results. Research shows that this technique works as well as more invasive MRI-only biopsies. Future advances, including artificial intelligence, may further improve accuracy and efficiency. This method could lead to better diagnosis and treatment decisions for patients with suspected prostate cancer.

**Assessment Category**	**Score**	**Assessment Category**	**Score**	**Assessment Category**	**Score**
Comprehension Score Rater 1	4	Gunning Fog Score	15.0	Words	149
Comprehension Score Rater 2	4	SMOG Index	10.8	Complex words	28
Reading Age (years)	19.5	Coleman Liau Index	16.4	Percentage of complex words	18.8
Flesch-Kincaid Reading Ease	40.9	Automated Readability Index	13.6	Average words per sentence	18.6
Flesch-Kincaid Grade Level	12.2	Sentences	8	Average syllables per word	1.7

Lay Summary (Extended Prompt)

Detecting prostate cancer early is important, and doctors now use a combination of MRI and ultrasound to improve the accuracy of prostate biopsies. This method helps find aggressive cancers while reducing unnecessary detection of slow-growing ones. MRI provides a clear image of the prostate, while ultrasound allows doctors to guide the biopsy in real time. There are different ways to combine these technologies, such as using a robotic arm or computer software. Studies show that this approach works as well as biopsies performed inside an MRI machine. However, its success depends on the quality of the MRI, the biopsy method used, and the doctor's experience. In the future, artificial intelligence may make this technique even better. This research helps improve how prostate cancer is diagnosed, leading to better treatment decisions and outcomes for patients.

**Assessment Category**	**Score**	**Assessment Category**	**Score**	**Assessment Category**	**Score**
Comprehension Score Rater 1	4	Gunning Fog Score	14.2	Words	135
Comprehension Score Rater 2	4	SMOG Index	10.5	Complex words	26
Reading Age (years)	18.5	Coleman Liau Index	15.4	Percentage of complex words	19.3
Flesch-Kincaid Reading Ease	44.9	Automated Readability Index	12.0	Average words per sentence	16.9
Flesch-Kincaid Grade Level	11.2	Sentences	8	Average syllables per word	1.7

Manuscript 151

Manenti G, Perretta T, Nezzo M, Fraioli FR, Carreri B, Gigliotti PE, et al. Transperineal Laser Ablation (TPLA) Treatment of Focal Low–Intermediate Risk Prostate Cancer. Cancers [Internet]. 2024 Apr 3;16(7):1404. Available from: http://dx.doi.org/10.3390/cancers16071404

Abstract

Background: This interventional pilot study aimed to evaluate the short-term (3 years) efficacy of focal laser ablation (FLA) in treating the index lesion of low-intermediate-risk prostate cancer, along with assessing the safety of the procedure (ClinicalTrials.gov ID NCT04045756). Methods: Forty patients aged between 46 and 86 with histologically proven organ-confined prostate cancer and low-to-intermediate progression risk were included. FLA was performed under percutaneous fusion magnetic resonance/ultrasound guidance in a Day Hospital setting under local anesthesia. Patients underwent regular clinical and functional assessments through the international index of erectile function (IIEF-5) and the International Prostatism Symptom Score (IPSS), PSA measurements, post-procedure MRI scans, and biopsies at 36 months or if positive findings were detected earlier. Statistical analyses were conducted to assess trends in PSA levels and cavity dimensions over time. Results: Forty patients were initially included, with fifteen lost to follow-up. At 36 months, a mean PSA reduction of 60% was observed, and 80% of MRI scans showed no signs of in-field clinically significant residual/recurrent cancer. Biopsies at 36 months revealed no malignant findings in 20 patients. No deterioration in sexual function or urinary symptoms was recorded. Conclusions: FLA appears to be safe, feasible, and effective in the index lesion treatment of low-intermediate-risk prostate cancer, with a high rate of tumor eradication and preservation of quality of life.

Keywords:

FLA, TPLA, focal laser ablation, mpMRI, prostate cancer, transperineal laser ablation

<u>Original Lay Summary</u>

Over the past few years, there has been a notable escalation in scholarly attention towards the implementation of focal therapeutic interventions for patients diagnosed with low- and intermediate-risk prostate cancer in order to achieve long-term cancer control and reduce morbidity associated with surgery and radiation therapy. This interventional pilot study (ClinicalTrials.gov ID NCT04045756) aimed to evaluate the short/medium-term (3 years) efficacy of trans perineal laser ablation (TPLA) in treating the index lesion of low–intermediate-risk prostate cancer, along with assessing the safety of the procedure and Quality of Life (QoL) of patients. Our results suggested that TPLA is a safe, feasible and effective treatment of low–intermediate-risk prostate cancer, with a high rate of tumor eradication and preservation of Quality of Life.

**Assessment Category**	**Score**	**Assessment Category**	**Score**	**Assessment Category**	**Score**
Comprehension Score Rater 1	5	Gunning Fog Score	22.1	Words	125
Comprehension Score Rater 2	5	SMOG Index	16.5	Complex words	33
Reading Age (years)	24.0	Coleman Liau Index	16.9	Percentage of complex words	26.4
Flesch-Kincaid Reading Ease	10.7	Automated Readability Index	20.4	Average words per sentence	31.3
Flesch-Kincaid Grade Level	19.5	Sentences	4	Average syllables per word	1.9

Lay Summary (Simple Prompt)

Prostate cancer is a common disease among men, and treating it effectively while preserving quality of life remains a challenge. This study explored a minimally invasive treatment called transperineal laser ablation (TPLA) for men with low-to-intermediate-risk prostate cancer. The procedure, performed under local anesthesia, uses targeted laser energy to destroy cancerous tissue while avoiding major surgery. Forty patients participated, and follow-ups over three years showed promising results. PSA levels, an indicator of cancer activity, dropped significantly, and imaging scans confirmed that most treated areas remained cancer-free. Follow-up biopsies revealed no cancer in many patients, and importantly, there were no negative effects on urinary or sexual function. These findings suggest that TPLA is a safe and effective treatment option that may offer an alternative to more aggressive therapies, potentially improving outcomes and quality of life for men with prostate cancer.

**Assessment Category**	**Score**	**Assessment Category**	**Score**	**Assessment Category**	**Score**
Comprehension Score Rater 1	5	Gunning Fog Score	17.4	Words	145
Comprehension Score Rater 2	5	SMOG Index	12.5	Complex words	33
Reading Age (years)	21.5	Coleman Liau Index	17.8	Percentage of complex words	22.8
Flesch-Kincaid Reading Ease	26.5	Automated Readability Index	15.8	Average words per sentence	20.7
Flesch-Kincaid Grade Level	14.7	Sentences	7	Average syllables per word	1.9

Lay Summary (Extended Prompt)

Prostate cancer is one of the most common cancers in men, and new treatment options are being explored to improve outcomes while preserving quality of life. This study tested a minimally invasive treatment called transperineal laser ablation (TPLA) for men with early-stage prostate cancer. The procedure was performed under local anesthesia using advanced imaging to target and destroy the cancer while avoiding damage to healthy tissue. Over three years, most patients showed a significant reduction in PSA levels, and MRI scans confirmed that the treatment successfully removed the cancer in 80% of cases. Follow-up biopsies found no remaining cancer in many patients, and no negative effects on urinary or sexual function were reported. These results suggest that TPLA is a safe and effective treatment for some men with prostate cancer, offering a less invasive alternative to surgery or radiation while maintaining quality of life.

**Assessment Category**	**Score**	**Assessment Category**	**Score**	**Assessment Category**	**Score**
Comprehension Score Rater 1	5	Gunning Fog Score	17.1	Words	146
Comprehension Score Rater 2	5	SMOG Index	12.3	Complex words	27
Reading Age (years)	20.5	Coleman Liau Index	15.5	Percentage of complex words	18.5
Flesch-Kincaid Reading Ease	36.7	Automated Readability Index	15.8	Average words per sentence	24.3
Flesch-Kincaid Grade Level	14.2	Sentences	6	Average syllables per word	1.7

Manuscript 152

Wakita H, Lu Y, Li X, Kobayashi T, Hachiya T, Ide H, et al. Evaluating Leukocyte Telomere Length and Myeloid-Derived Suppressor Cells as Biomarkers for Prostate Cancer. Cancers [Internet]. 2024 Mar 31;16(7):1386. Available from: http://dx.doi.org/10.3390/cancers16071386

Abstract

Background: Leukocyte telomere length (LTL) and myeloid-derived suppressor cells (MDSC) are associated with aging and the development and progression of cancer. However, the exact nature of this relationship remains unclear. Our study aimed to investigate the potential of LTL and MDSC as diagnostic biomarkers for prostate cancer while also seeking to deepen our understanding of the relationship of these potential biomarkers to each other.

Methods: Our study involved patients undergoing a prostate biopsy. We analyzed the relative LTL in genomic DNA obtained from peripheral blood leukocytes as well as the percentage of MDSC and their subtypes in peripheral blood mononuclear cells (PBMC). Our evaluation focused on examining the relationship between LTL and MDSC and pathological diagnoses as well as investigating the correlation between LTL and MDSC levels.

Results: In our study of 102 participants, 56 were pathologically diagnosed with localized prostate cancer (cancer group), while 46 tested negative (control group). The cancer group exhibited significantly shorter LTL in comparison to the control group ($p = 0.024$). Additionally, the cancer group showed a tendency towards a higher percentage of monocytic MDSC (M-MDSC), although this difference did not reach statistical significance ($p = 0.056$). Our multivariate logistic regression analysis revealed that patients with shorter LTL and higher percentages of M-MDSC had a 2.98-fold (95% CI = 1.001-8.869, $p = 0.049$) and 3.03-fold (95% CI = 1.152-7.977, $p = 0.025$) increased risk of prostate cancer diagnosis, respectively. There was also a

significant negative correlation between LTL and M-MDSC. ($r = -0.347$, $p < 0.001$).

Conclusions: Our research has established a correlation between LTL and MDSC in patients undergoing biopsy for prostate cancer. Notably, we observed that individuals with localized prostate cancer tend to have shorter LTL and a higher percentage of M-MDSC prior to their diagnosis. These findings suggest that LTL and M-MDSC could potentially serve as adjunctive biomarkers for the early diagnosis of prostate cancer.

Keywords:

aging, leukocyte telomere length (LTL), myeloid-derived suppressor cell (MDSC), prostate cancer

<u>Original Lay Summary</u>

Prostate cancer is the most common cancer in men. The pursuit of novel biomarkers for early detection of prostate cancer poses a contemporary challenge given the age-associated escalation in prostate cancer risk and severity. Our focus was directed towards evaluating leukocyte telomere length (LTL) and myeloid-derived suppressor cells (MDSC) in prostate cancer patients, considering their potential as adjunctive diagnostic markers. In a study of 102 patients who underwent prostate biopsy, those diagnosed with prostate cancer demonstrated significantly shorter LTL and an increased proportion of M-MDSC prior to diagnosis, along with elevated PSA levels and age, in comparison to controls. Furthermore, a significant negative correlation was observed between LTL and MDSC levels. This initial report of those findings could potentially contribute to a deeper understanding of the molecular, biological, and immunological factors involved in cancer development.

**Assessment Category**	**Score**	**Assessment Category**	**Score**	**Assessment Category**	**Score**
Comprehension Score Rater 1	5	Gunning Fog Score	19.6	Words	138
Comprehension Score Rater 2	5	SMOG Index	14.1	Complex words	36
Reading Age (years)	22.5	Coleman Liau Index	18.6	Percentage of complex words	26.1
Flesch-Kincaid Reading Ease	13.7	Automated Readability Index	17.6	Average words per sentence	23.0
Flesch-Kincaid Grade Level	17.1	Sentences	6	Average syllables per word	2.0

<u>Lay Summary (Simple Prompt)</u>

Prostate cancer is one of the most common cancers in men, and early detection is crucial for better treatment outcomes. Scientists are looking for new ways to identify prostate cancer before it becomes advanced. This study investigated whether two biological markers—leukocyte telomere length (LTL) and myeloid-derived suppressor cells (MDSC)—can help detect prostate cancer. By analyzing blood samples from men undergoing a prostate biopsy, researchers found that patients with prostate cancer had shorter LTL and a higher percentage of certain MDSC types. These findings suggest that these markers may help doctors assess cancer risk and improve early detection methods. While further research is needed, this study provides important insights into how biological changes in the immune system may be linked to prostate cancer and could lead to better diagnostic tools in the future.

**Assessment Category**	**Score**	**Assessment Category**	**Score**	**Assessment Category**	**Score**
Comprehension Score Rater 1	5	Gunning Fog Score	13.4	Words	134
Comprehension Score Rater 2	5	SMOG Index	9.2	Complex words	15
Reading Age (years)	19.5	Coleman Liau Index	16.6	Percentage of complex words	11.2
Flesch-Kincaid Reading Ease	40.2	Automated Readability Index	15.6	Average words per sentence	22.3
Flesch-Kincaid Grade Level	13.2	Sentences	6	Average syllables per word	1.7

Lay Summary (Extended Prompt)

Prostate cancer is one of the most common cancers in men, but finding better ways to detect it early is still a challenge. This study looked at two possible biological markers in the blood—leukocyte telomere length (LTL) and myeloid-derived suppressor cells (MDSC)—to see if they could help identify prostate cancer. Researchers analyzed blood samples from 102 men who underwent a prostate biopsy, comparing those with and without prostate cancer. They found that men with cancer had shorter LTL and a higher percentage of a specific type of MDSC. These factors were linked to an increased risk of prostate cancer. The results suggest that measuring LTL and MDSC levels could support early detection efforts, potentially leading to improved diagnosis and better outcomes for patients. More research is needed, but these findings may help doctors develop new tools for identifying prostate cancer sooner.

**Assessment Category**	**Score**	**Assessment Category**	**Score**	**Assessment Category**	**Score**
Comprehension Score Rater 1	5	Gunning Fog Score	13.2	Words	143
Comprehension Score Rater 2	5	SMOG Index	9.3	Complex words	18
Reading Age (years)	17.5	Coleman Liau Index	14.7	Percentage of complex words	12.6
Flesch-Kincaid Reading Ease	50.6	Automated Readability Index	13.2	Average words per sentence	20.4
Flesch-Kincaid Grade Level	11.3	Sentences	7	Average syllables per word	1.6

Manuscript 153

Olivero A, Tappero S, Chierigo F, Maltzman O, Secco S, Palagonia E, et al. A Comprehensive Overview of Intraoperative Complications during Retzius-Sparing Robot-Assisted Radical Prostatectomy: Single Series from High-Volume Center. Cancers [Internet]. 2024 Mar 31;16(7):1385. Available from: http://dx.doi.org/10.3390/cancers16071385

Abstract

Background: Intraoperative complications (ICs) are invariably underreported in urological surgery despite the recent endorsement of new classification systems. We aimed to provide a detailed overview of ICs during Retzius-sparing robot-assisted radical prostatectomy (RS-RARP).

Methods: We prospectively collected data from 1891 patients who underwent RS-RARP at a single high-volume European center from January 2010 to December 2022. ICs were collected based on surgery reports and categorized according to the Intraoperative Adverse Incident Classification (EAUiaiC). The quality criteria for accurate and comprehensive reporting of intraoperative adverse events proposed by the Intraoperative Complications Assessment and Reporting with Universal Standards (ICARUS) Global Surgical Collaboration Project were fulfilled. To better classify the role of the RS-RARP approach, ICs were classified into anesthesiologic and surgical ICs. Surgical ICs were further divided according to the timing of the complication in RARP-related ICs and ePNLD-related ICs.

Results: Overall, 40 ICs were reported in 40 patients (2.1%). Ten out of thirteen ICARUS criteria were satisfied. According to EAUiaiC grading of ICs, 27 (67.5%), 7 (17.5%), 2 (5%), 2 (5%), and 2 (5%) patients experienced Grade 1, 2, 3, 4A, and 4B, respectively. When we classified the ICs, two cases (5%) were classified as anesthesiologic ICs. Among the 38 surgical ICs, 16 (42%) were ePNLD-related, and 22 (58%) were RARP-related. ICs led to seven (0.37%) post-operative sequelae (four

non-permanent and three permanent). Patients who suffered ICs were significantly older (67 years vs. 65 years, p = 0.02) and had a higher median BMI (27.0 vs. 26.1, p = 0.01), but did not differ in terms of comorbidities or tumor characteristics (all p values ≥ 0.05).

Conclusions: Intraoperative complications during RS-RARP are relatively infrequent, but should not be underestimated. Patients suffering from ICs are older, have a higher body mass index, a higher rate of intraoperative blood transfusion, and a longer length of stay.
Keywords:
adverse events, intraoperative complications, prostate cancer, robot-assisted radical prostatectomy, robotic surgery

Original Lay Summary

Intraoperative complications during Retzius-sparing robot-assisted radical prostatectomy are uncommon, affecting 2.1% of patients in our series of almost two thousand cases. Only 0.37% of the patients treated with Retzius-sparing robot-assisted radical prostatectomy developed post-operative sequelae due to Intraoperative complications. When divided according to the time of the procedure 42% of the events were ePNLD-related, and the 58% were RARP-related Intraoperative complications are more likely to occur in older individuals with a higher BMI. Patient suffering intraoperative complications demonstrated a higher rate of intraoperative blood transfusion, and a longer length of stay. These complications are mostly of low severity but underline the need for careful monitoring and reporting.

**Assessment Category**	**Score**	**Assessment Category**	**Score**	**Assessment Category**	**Score**
Comprehension Score Rater 1	5	Gunning Fog Score	17.3	Words	116
Comprehension Score Rater 2	5	SMOG Index	13.1	Complex words	36
Reading Age (years)	20.5	Coleman Liau Index	17.8	Percentage of complex words	31.0
Flesch-Kincaid Reading Ease	18.6	Automated Readability Index	13.8	Average words per sentence	16.6
Flesch-Kincaid Grade Level	14.8	Sentences	7	Average syllables per word	2.0

<u>Lay Summary (Simple Prompt)</u>

Prostate cancer is often treated with a surgical procedure called Retzius-sparing robot-assisted radical prostatectomy (RS-RARP), which preserves important structures to improve recovery. However, complications that occur during surgery are not always well-documented. This study analyzed data from 1,891 patients who underwent RS-RARP at a high-volume surgical center to provide a clearer picture of the risks involved. Researchers classified complications based on international surgical reporting standards and found that intraoperative complications were relatively rare, occurring in only 2.1% of patients. Most complications were minor, but a small percentage led to lasting effects. Patients who experienced complications were generally older and had a higher body mass index. These findings highlight the importance of accurate complication reporting to improve patient safety, refine surgical techniques, and help doctors better inform patients about potential risks before surgery.

**Assessment Category**	**Score**	**Assessment Category**	**Score**	**Assessment Category**	**Score**
Comprehension Score Rater 1	5	Gunning Fog Score	17.6	Words	140
Comprehension Score Rater 2	5	SMOG Index	12.6	Complex words	38
Reading Age (years)	21.5	Coleman Liau Index	19.1	Percentage of complex words	27.1
Flesch-Kincaid Reading Ease	22.3	Automated Readability Index	15.2	Average words per sentence	17.5
Flesch-Kincaid Grade Level	14.5	Sentences	8	Average syllables per word	2.0

Lay Summary (Extended Prompt)

When undergoing surgery to remove the prostate due to cancer, some patients experience complications during the procedure. However, these issues are not always fully reported or categorized in detail. This study examined complications that occurred during a specific type of robotic prostate surgery, called Retzius-sparing robot-assisted radical prostatectomy (RS-RARP), in nearly 1,900 patients at a high-volume medical center. The researchers carefully recorded and classified complications to better understand their causes and effects. They found that complications were rare, occurring in just over 2% of cases, and were more common in older patients and those with a higher body mass index. Some complications led to longer hospital stays or the need for a blood transfusion. These findings help improve surgical safety by identifying risk factors and providing surgeons with better ways to prevent and manage complications in future patients.

**Assessment Category**	**Score**	**Assessment Category**	**Score**	**Assessment Category**	**Score**
Comprehension Score Rater 1	5	Gunning Fog Score	17.1	Words	143
Comprehension Score Rater 2	5	SMOG Index	12.5	Complex words	33
Reading Age (years)	20.5	Coleman Liau Index	16.6	Percentage of complex words	23.1
Flesch-Kincaid Reading Ease	32.9	Automated Readability Index	14.7	Average words per sentence	20.4
Flesch-Kincaid Grade Level	13.7	Sentences	7	Average syllables per word	1.8

Manuscript 154

Orlovskiy S, Gupta PK, Roman J, Arias-Mendoza F, Nelson DS, Koch CJ, et al. Lonidamine Induced Selective Acidification and De-Energization of Prostate Cancer Xenografts: Enhanced Tumor Response to Radiation Therapy. Cancers [Internet]. 2024 Mar 31;16(7):1384. Available from: http://dx.doi.org/10.3390/cancers16071384

Abstract

Prostate cancer is a multi-focal disease that can be treated using surgery, radiation, androgen deprivation, and chemotherapy, depending on its presentation. Standard dose-escalated radiation therapy (RT) in the range of 70-80 Gray (GY) is a standard treatment option for prostate cancer. It could be used at different phases of the disease (e.g., as the only primary treatment when the cancer is confined to the prostate gland, combined with other therapies, or as an adjuvant treatment after surgery). Unfortunately, RT for prostate cancer is associated with gastro-intestinal and genitourinary toxicity. We have previously reported that the metabolic modulator lonidamine (LND) produces cancer sensitization through tumor acidification and de-energization in diverse neoplasms. We hypothesized that LND could allow lower RT doses by producing the same effect in prostate cancer, thus reducing the detrimental side effects associated with RT. Using the Seahorse XFe96 and YSI 2300 Stat Plus analyzers, we corroborated the expected LND-induced intracellular acidification and de-energization of isolated human prostate cancer cells using the PC3 cell line. These results were substantiated by non-invasive 31P magnetic resonance spectroscopy (MRS), studying PC3 prostate cancer xenografts treated with LND (100 mg/kg, i.p.). In addition, we found that LND significantly increased tumor lactate levels in the xenografts using 1H MRS non-invasively. Subsequently, LND was combined with radiation therapy in a growth delay experiment, where we found that 150 μM LND followed by 4 GY RT produced a significant growth delay in PC3 prostate cancer xenografts, compared to either control, LND, or RT alone. We conclude that the metabolic modulator LND radio-sensitizes experimental prostate cancer

models, allowing the use of lower radiation doses and diminishing the potential side effects of RT. These results suggest the possible clinical translation of LND as a radio-sensitizer in patients with prostate cancer.

Keywords:

1H and 31P magnetic resonance spectroscopy, lonidamine, prostate cancer, radiation growth delay, radiation therapy, tumor acidification, tumor de-energization

Original Lay Summary

Early prostate cancer treatment with radiation therapy is effective but involves substantial harmful side effects throughout therapy. This study explores the use of the metabolic modulator lonidamine (LND) to sensitize prostate cancer cells to radiation therapy. 1H and 31P magnetic resonance spectroscopy (MRS) were used to non-invasively monitor metabolic changes associated with LND treatment in human prostate cancer tumors implanted in athymic nude mice. The in vivo MRS results were substantiated using classic biochemistry methods by gathering in vitro data from isolated human prostate cancer cell lines. A radiation growth delay experiment performed in xenografted mice combining LND and radiation therapy showed that LND-modified prostate cancer metabolism and significantly increased the efficacy of radiation therapy. These findings indicate that less radiation may be required with LND, reducing side effects, and potentially improving patient management in the clinic.

**Assessment Category**	**Score**	**Assessment Category**	**Score**	**Assessment Category**	**Score**
Comprehension Score Rater 1	5	Gunning Fog Score	21.7	Words	138
Comprehension Score Rater 2	5	SMOG Index	15.4	Complex words	43
Reading Age (years)	24.0	Coleman Liau Index	19.6	Percentage of complex words	31.2
Flesch-Kincaid Reading Ease	7.5	Automated Readability Index	18.4	Average words per sentence	23.0
Flesch-Kincaid Grade Level	17.9	Sentences	6	Average syllables per word	2.1

Lay Summary (Simple Prompt)

Prostate cancer is often treated with radiation therapy, but high doses can cause serious side effects. This study explores a way to make radiation therapy more effective while reducing its harmful impact. Researchers tested a drug called lonidamine (LND), which weakens cancer cells by lowering their energy and increasing their acidity. Using laboratory techniques and imaging, they confirmed that LND affects prostate cancer cells as expected. When combined with radiation therapy, LND significantly slowed tumor growth in experimental models. These findings suggest that using LND with lower radiation doses could still effectively treat prostate cancer while reducing side effects. If further studies confirm these results, this approach could improve treatment options for patients, making radiation therapy safer and more tolerable.

**Assessment Category**	**Score**	**Assessment Category**	**Score**	**Assessment Category**	**Score**
Comprehension Score Rater 1	5	Gunning Fog Score	15.5	Words	120
Comprehension Score Rater 2	5	SMOG Index	11.2	Complex words	26
Reading Age (years)	20.5	Coleman Liau Index	18.8	Percentage of complex words	21.7
Flesch-Kincaid Reading Ease	30.1	Automated Readability Index	14.8	Average words per sentence	17.1
Flesch-Kincaid Grade Level	13.3	Sentences	7	Average syllables per word	1.9

Lay Summary (Extended Prompt)

Prostate cancer is often treated with radiation therapy, but high doses can cause serious side effects. Scientists are exploring ways to make tumors more sensitive to radiation so that lower doses can be used safely. This study tested a drug called lonidamine (LND), which changes the way cancer cells produce energy, making them more vulnerable to treatment. Researchers found that LND made prostate cancer cells more acidic and less energized, weakening them. When LND was combined with a lower dose of radiation, tumors in experimental models grew more slowly than with radiation alone. This suggests that LND could help doctors reduce radiation doses while still effectively treating prostate cancer. If confirmed in clinical trials, this approach could lower treatment side effects and improve the quality of life for patients.

**Assessment Category**	**Score**	**Assessment Category**	**Score**	**Assessment Category**	**Score**
Comprehension Score Rater 1	5	Gunning Fog Score	13.6	Words	129
Comprehension Score Rater 2	5	SMOG Index	9.8	Complex words	20
Reading Age (years)	18.5	Coleman Liau Index	15.6	Percentage of complex words	15.5
Flesch-Kincaid Reading Ease	48.4	Automated Readability Index	12.9	Average words per sentence	18.4
Flesch-Kincaid Grade Level	11.1	Sentences	7	Average syllables per word	1.7

Manuscript 155

Sequeira JP, Salta S, Freitas R, López-López R, Díaz-Lagares Á, Henrique R, et al. Biomarkers for Pre-Treatment Risk Stratification of Prostate Cancer Patients: A Systematic Review. Cancers [Internet]. 2024 Mar 30;16(7):1363. Available from: http://dx.doi.org/10.3390/cancers16071363

Abstract

Background: Prostate cancer (PCa) is one of the most frequently occurring malignancies. Although most cases are not life-threatening, approximately 20% endure an unfavorable outcome. PSA-based screening reduced mortality but at the cost of an increased overdiagnosis/overtreatment of low-risk (lrPCa) and favorable intermediate-risk (firPCa) PCa. PCa risk-groups are usually identified based on serum Prostate-Specific Antigen (PSA), the Gleason score, and clinical T stage, which have consistent although variable specificity or subjectivity. Thus, more effective and specific tools for risk assessment are needed, ideally making use of minimally invasive methods such as liquid biopsies. In this systematic review we assessed the clinical potential and analytical performance of liquid biopsy-based biomarkers for pre-treatment risk stratification of PCa patients.

Methods: Studies that assessed PCa pre-treatment risk were retrieved from PubMed, Scopus, and MedLine. PCa risk biomarkers were analyzed, and the studies' quality was assessed using the QUADAS-2 tool.

Results: The final analysis comprised 24 full-text articles, in which case-control studies predominated, mostly reporting urine-based biomarkers (54.2%) and biomarker quantification by qPCR (41.7%). Categorization into risk groups was heterogeneous, predominantly making use of the Gleason score.

Conclusion: This systematic review unveils the substantial clinical promise of using circulating biomarkers in assessing the risk for prostate cancer patients. However,

the standardization of groups, categories, and biomarker validation are mandatory before this technique can be implemented. Circulating biomarkers might represent a viable alternative to currently available tools, obviating the need for tissue biopsies, and allowing for faster and more cost-effective testing, with superior analytical performance, specificity, and reproducibility.

Keywords:

biomarkers, liquid biopsy, prostatic neoplasms, risk assessment

<u>Original Lay Summary</u>

PCa remains a leading health concern worldwide. Serum PSA-based PCa screening led to a well-documented decreased mortality but at the cost of the increased overdiagnosis/overtreatment of indolent disease. Although various tools have been developed to predict PCa patient outcome prior to treatment, mostly based on serum PSA, the Gleason score, and clinical T stage, all have a suboptimal performance and require tissue biopsies from the prostate. To obviate that need, overcome Gleason score subjectivity and the limited specificity of serum PSA, devising more effective tools is mandatory, while also taking the opportunity to adopt minimally invasive strategies based on liquid biopsies.

**Assessment Category**	**Score**	**Assessment Category**	**Score**	**Assessment Category**	**Score**
Comprehension Score Rater 1	3	Gunning Fog Score	19.2	Words	103
Comprehension Score Rater 2	4	SMOG Index	13.8	Complex words	23
Reading Age (years)	22.5	Coleman Liau Index	17.1	Percentage of complex words	22.3
Flesch-Kincaid Reading Ease	21.4	Automated Readability Index	17.8	Average words per sentence	25.8
Flesch-Kincaid Grade Level	16.7	Sentences	4	Average syllables per word	1.9

<u>Lay Summary (Simple Prompt)</u>

Prostate cancer is a common disease, but not all cases require aggressive treatment. Doctors currently use blood tests, biopsy results, and scans to assess the risk of a patient's cancer progressing. However, these methods have limitations and may lead to unnecessary treatments. In this study, we reviewed research on a promising alternative—biomarkers found in blood and urine, known as liquid biopsies. These biomarkers could help doctors more accurately determine a patient's risk level before treatment, potentially reducing the need for invasive biopsies. Our findings show that liquid biopsies have strong potential for improving prostate cancer risk assessment, but further studies are needed to standardize the testing methods. If properly validated, these biomarkers could lead to quicker, more precise, and less invasive risk assessment, helping doctors make better treatment decisions while reducing unnecessary procedures for patients.

**Assessment Category**	**Score**	**Assessment Category**	**Score**	**Assessment Category**	**Score**
Comprehension Score Rater 1	5	Gunning Fog Score	16.9	Words	135
Comprehension Score Rater 2	5	SMOG Index	12.2	Complex words	31
Reading Age (years)	21.5	Coleman Liau Index	18.7	Percentage of complex words	23.0
Flesch-Kincaid Reading Ease	29.3	Automated Readability Index	15.8	Average words per sentence	19.3
Flesch-Kincaid Grade Level	14.0	Sentences	7	Average syllables per word	1.9

<u>Lay Summary (Extended Prompt)</u>

Prostate cancer is a common disease, but not all cases are life-threatening. Doctors use tests like PSA levels and tissue samples to decide how risky a patient's cancer might be. However, these methods are not always precise and can lead to unnecessary treatments. This study reviewed research on a new type of test that looks for cancer-related substances in blood and urine, known as liquid biopsy biomarkers. These tests could help doctors more accurately predict how aggressive a patient's cancer is before treatment, without needing an invasive biopsy. The researchers found promising results, but these tests need further validation and standardization before they can be widely used. If proven reliable, liquid biopsies could make diagnosing prostate cancer easier, faster, and more accurate, reducing unnecessary treatments while ensuring high-risk patients receive the care they need.

**Assessment Category**	**Score**	**Assessment Category**	**Score**	**Assessment Category**	**Score**
Comprehension Score Rater 1	5	Gunning Fog Score	14.3	Words	137
Comprehension Score Rater 2	5	SMOG Index	10.3	Complex words	22
Reading Age (years)	18.5	Coleman Liau Index	16.0	Percentage of complex words	16.1
Flesch-Kincaid Reading Ease	41.9	Automated Readability Index	13.8	Average words per sentence	19.6
Flesch-Kincaid Grade Level	12.3	Sentences	7	Average syllables per word	1.7

Manuscript 156

Campanelli G, Francois E, Parupathi P, Devarakonda LS, Yang C, Kumar A, et al. The Therapeutic Efficacy and Mechanism of Action of Gnetin C, a Natural Compound from the Melinjo Plant, in a Preclinical Mouse Model of Advanced Prostate Cancer. Cancers [Internet]. 2024 Mar 29;16(7):1344. Available from: http://dx.doi.org/10.3390/cancers16071344

Abstract

The metastasis-associated protein 1/protein kinase B (MTA1/AKT) signaling pathway has been shown to cooperate in promoting prostate tumor growth. Targeted interception strategies by plant-based polyphenols, specifically stilbenes, have shown great promise against MTA1-mediated prostate cancer progression. In this study, we employed a prostate-specific transgenic mouse model with MTA1 overexpression on the background of phosphatase and tensin homolog (Pten) null (R26MTA1; Ptenf/f) and PC3M prostate cancer cells which recapitulate altered molecular pathways in advanced prostate cancer. Mechanistically, the MTA1 knockdown or pharmacological inhibition of MTA1 by gnetin C (dimer resveratrol) in cultured PC3M cells resulted in the marked inactivation of mammalian target of rapamycin (mTOR) signaling. In vivo, mice tolerated a daily intraperitoneal treatment of gnetin C (7 mg/kg bw) for 12 weeks without any sign of toxicity. Treatment with gnetin C markedly reduced cell proliferation and angiogenesis and promoted apoptosis in mice with advanced prostate cancer. Further, in addition to decreasing MTA1 levels in prostate epithelial cells, gnetin C significantly reduced mTOR signaling activity in prostate tissues, including the activity of mTOR-target proteins: p70 ribosomal protein S6 kinase (S6K) and eukaryotic translational initiation factor 4E (elF4E)-binding protein 1 (4EBP1). Collectively, these findings established gnetin C as a new natural compound with anticancer properties against MTA1/AKT/mTOR-activated prostate cancer, with potential as monotherapy and as a possible adjunct to clinically approved mTOR pathway inhibitors in the future.

Keywords:

MTA1/mTOR, advanced prostate cancer, anticancer effects, gnetin C, natural products, plant-derived polyphenols, targeted therapeutics

<u>Original Lay Summary</u>

Incidence and mortality rates for prostate cancer remain high due to advanced disease characterized by the heterogeneous activation of numerous molecular pathways. In the current study, utilizing a genetic mouse model of advanced prostate cancer with hyperactivated metastasis-associated protein 1/mammalian target of rapamycin (MTA1/mTOR) tumor-promoting pathway, we show for the first time that gnetin C, a natural compound from the melinjo plant, blocks the progression of prostate cancer by reducing cell proliferation and angiogenesis and promoting cell death through the efficient targeting of the MTA1/mTOR pathway. These data may provide a foundation from which to explore gnetin C as a monotherapy and/or combination therapy with approved drugs against advanced prostate cancer in patients with a loss of phosphatase and tensin homolog (PTEN) expression and activated MTA1/mTOR signaling.

**Assessment Category**	**Score**	**Assessment Category**	**Score**	**Assessment Category**	**Score**
Comprehension Score Rater 1	5	Gunning Fog Score	26.8	Words	129
Comprehension Score Rater 2	5	SMOG Index	19.0	Complex words	33
Reading Age (years)	24.0	Coleman Liau Index	18.4	Percentage of complex words	25.6
Flesch-Kincaid Reading Ease	-2.7	Automated Readability Index	27.4	Average words per sentence	43.0
Flesch-Kincaid Grade Level	24.3	Sentences	3	Average syllables per word	2.0

<u>Lay Summary (Simple Prompt)</u>

Prostate cancer remains a significant health challenge, especially in advanced stages where effective treatments are limited. This study explores the potential of gnetin C, a natural compound found in the melinjo plant, as a new treatment option. Researchers tested gnetin C in laboratory-grown prostate cancer cells and in mice with aggressive prostate tumors. The results showed that gnetin C effectively slowed tumor growth by blocking key cancer-promoting signals while being well tolerated without toxicity. Specifically, it reduced cancer cell proliferation, prevented the formation of new blood vessels that feed tumors, and promoted cancer cell death. These findings suggest that gnetin C could be a promising natural therapy for advanced prostate cancer. In the future, it may be used alone or alongside existing treatments that target the same cancer-related pathways, offering hope for improved therapeutic options.

**Assessment Category**	**Score**	**Assessment Category**	**Score**	**Assessment Category**	**Score**
Comprehension Score Rater 1	5	Gunning Fog Score	15.7	Words	138
Comprehension Score Rater 2	5	SMOG Index	11.4	Complex words	27
Reading Age (years)	19.5	Coleman Liau Index	17.2	Percentage of complex words	19.6
Flesch-Kincaid Reading Ease	36.6	Automated Readability Index	14.8	Average words per sentence	19.7
Flesch-Kincaid Grade Level	13.0	Sentences	7	Average syllables per word	1.8

Lay Summary (Extended Prompt)

Prostate cancer is a serious disease that can be hard to treat, especially in its advanced stages. Scientists are always looking for new ways to slow down cancer growth and improve treatment options. In this study, researchers tested a natural compound called gnetin C, which comes from the Melinjo plant, to see if it could help fight prostate cancer. They used a special type of lab-grown cancer cells and a mouse model that mimics advanced prostate cancer. The results showed that gnetin C blocked a key cancer growth pathway, reduced tumor size, and slowed cancer cell growth—without causing harmful side effects. These findings suggest that gnetin C could be a promising new treatment option, either on its own or combined with existing therapies. This research provides hope for developing safer, plant-based cancer treatments in the future.

**Assessment Category**	**Score**	**Assessment Category**	**Score**	**Assessment Category**	**Score**
Comprehension Score Rater 1	5	Gunning Fog Score	10.5	Words	138
Comprehension Score Rater 2	5	SMOG Index	7.1	Complex words	10
Reading Age (years)	16.5	Coleman Liau Index	13.8	Percentage of complex words	7.3
Flesch-Kincaid Reading Ease	63.0	Automated Readability Index	12.1	Average words per sentence	19.7
Flesch-Kincaid Grade Level	9.4	Sentences	7	Average syllables per word	1.5

Manuscript 157

Friedemann M, Jandeck C, Tautz L, Gutewort K, von Rein L, Sukocheva O, et al. Blood-Based DNA Methylation Analysis by Multiplexed OBBPA-ddPCR to Verify Indications for Prostate Biopsies in Suspected Prostate Cancer Patients. Cancers [Internet]. 2024 Mar 28;16(7):1324. Available from: http://dx.doi.org/10.3390/cancers16071324

Abstract

Current prostate carcinoma (PCa) biomarkers, including total prostate-specific antigen (tPSA), have unsatisfactory diagnostic sensitivity and specificity resulting in overdiagnosis and overtreatment. Previously, we described an optimised bias-based preamplification-digital droplet PCR (OBBPA-ddPCR) technique, which detects tumour DNA in blood-derived cell-free DNA (cfDNA) of cancer patients. The current study investigated the performance of newly developed OBBPA-ddPCR-based biomarkers. Blood plasma samples from healthy individuals (n = 90, controls) and PCa (n = 39) and benign prostatic hyperplasia patients (BPH, n = 40) were analysed. PCa and BPH patients had tPSA values within a diagnostic grey area of 2-15 ng/mL, for whom further diagnostic validation is most crucial. Methylation levels of biomarkers RASSF1A, MIR129-2, NRIP3, and SOX8 were found significantly increased in PCa patients compared to controls. By combining classical PCa risk factors (percentage of free PSA compared to tPSA (QfPSA) and patient's age) with cfDNA-based biomarkers, we developed PCa risk scores with improved sensitivity and specificity compared to established tPSA and QfPSA single-marker analyses. The diagnostic specificity was increased to 70% with 100% sensitivity for clinically significant PCa patients. Thus, prostate biopsies could be avoided for 28 out of 40 BPH patients. In conclusion, the newly developed risk scores may help to confirm the clinical decision and prevent unnecessary prostate biopsy.

Keywords:

DNA methylation, cell-free tumour DNA, circulating cell-free DNA (cfDNA), digital PCR, liquid biopsy, prostate cancer

<u>Original Lay Summary</u>

A multiplexed optimised bias-based preamplification–digital droplet PCR (OBBPA-ddPCR) diagnostic technique was developed and tested in this study. The method estimates blood-derived cell-free DNA (cfDNA) methylation levels of RASSF1A, MIR129-2, NRIP3, and SOX8 target sequences in blood samples. The study included 90 healthy individuals, 40 benign prostatic hyperplasia patients, and 39 prostate cancer (PCa) patients. The risk scores were developed to further reduce unnecessary prostate biopsies and PCa overdiagnosis. The scores included classical risk factors and methylation data, delivering an improved 70% specificity for BPH patients and 100% sensitivity for clinically significant PCa patients.

**Assessment Category**	**Score**	**Assessment Category**	**Score**	**Assessment Category**	**Score**
Comprehension Score Rater 1	5	Gunning Fog Score	18.5	Words	95
Comprehension Score Rater 2	5	SMOG Index	13.2	Complex words	26
Reading Age (years)	22.5	Coleman Liau Index	19.5	Percentage of complex words	27.4
Flesch-Kincaid Reading Ease	14.8	Automated Readability Index	16.3	Average words per sentence	19.0
Flesch-Kincaid Grade Level	15.9	Sentences	5	Average syllables per word	2.0

<u>Lay Summary (Simple Prompt)</u>

Prostate cancer diagnosis often relies on a blood test called PSA, which can be unreliable, leading to unnecessary biopsies. This study explores a new blood-based test that detects cancer-related DNA changes to improve prostate cancer detection. Researchers analyzed blood samples from healthy individuals, prostate cancer patients, and those with a non-cancerous prostate condition (benign prostatic hyperplasia, BPH). By combining cancer-related DNA markers with existing risk factors, they developed a more accurate risk score. This approach correctly identified all clinically significant prostate cancers while reducing unnecessary biopsies in patients with non-cancerous conditions. The findings suggest that this new test could help doctors make better decisions about who truly needs a biopsy, reducing unnecessary procedures and improving prostate cancer diagnosis.

**Assessment Category**	**Score**	**Assessment Category**	**Score**	**Assessment Category**	**Score**
Comprehension Score Rater 1	5	Gunning Fog Score	19.3	Words	123
Comprehension Score Rater 2	5	SMOG Index	13.7	Complex words	34
Reading Age (years)	22.5	Coleman Liau Index	19.5	Percentage of complex words	27.6
Flesch-Kincaid Reading Ease	20.3	Automated Readability Index	17.0	Average words per sentence	20.5
Flesch-Kincaid Grade Level	15.5	Sentences	6	Average syllables per word	2.0

<u>Lay Summary (Extended Prompt)</u>

Prostate cancer is often difficult to diagnose accurately, leading to unnecessary biopsies that can be uncomfortable and risky for patients. Currently, doctors use a blood test called PSA to check for prostate cancer, but it is not always reliable. In this study, researchers tested a new method that looks for cancer-related changes in DNA found in the blood. By analyzing specific DNA markers and combining them with traditional risk factors, they developed a more precise test to identify men who truly need a biopsy. This new approach correctly identified all cases of significant prostate cancer while avoiding unnecessary biopsies for many men with non-cancerous conditions. These findings suggest that this blood test could improve prostate cancer diagnosis, helping doctors make better decisions and reducing unnecessary medical procedures for patients.

**Assessment Category**	**Score**	**Assessment Category**	**Score**	**Assessment Category**	**Score**
Comprehension Score Rater 1	5	Gunning Fog Score	18.2	Words	131
Comprehension Score Rater 2	5	SMOG Index	13.1	Complex words	31
Reading Age (years)	21.5	Coleman Liau Index	16.5	Percentage of complex words	23.7
Flesch-Kincaid Reading Ease	29.7	Automated Readability Index	15.3	Average words per sentence	21.8
Flesch-Kincaid Grade Level	14.5	Sentences	6	Average syllables per word	1.8

Manuscript 158

Pinheiro PS, Koru-Sengul T, Zhao W, Hernandez DR, Hernandez MN, Kobetz EN, et al. Distinct Prostate Cancer Survival Outcomes in Firefighters: A Population-Based Study. Cancers [Internet]. 2024 Mar 27;16(7):1305. Available from: http://dx.doi.org/10.3390/cancers16071305

Abstract

Introduction: Survival outcomes for prostate cancer among specific occupational groups prone to regular medical check-ups vis-à-vis the general population have been understudied. For firefighters, a demographic subject to rigorous medical evaluations, possessing above-average medical expertise, and exposed to specific carcinogens of interest, prostate cancer survival in the US has never been studied. Methods: We conducted a retrospective study, utilizing data from the Florida Cancer Data System spanning 2004 to 2014, coupled with firefighter certification records from the Florida State Fire Marshal's Office. Our study cohort consisted of 1058 prostate cancer cases among firefighters as well as prostate cases for the Florida general population (n = 150,623). We compared cause-specific survival between the two using Cox regression models adjusted for demographics and clinical characteristics, including PSA levels, Gleason scores, and treatment modalities. Results: Firefighters demonstrated a higher five-year cause-specific survival rate (96.1%, 95% CI: 94.7-97.1%) than the general population (94.2%, 95%CI: 94.1-94.3%). Overall, firefighters' diagnoses were established at younger ages (median age 63 vs. 67 in the general population), exhibited a higher proportion of localized stage cancers (84.7% vs. 81.1%), and had a greater utilization of surgery (46.4% vs. 37.6%), a treatment modality with a high success rate but potential side effects. In multivariable analysis, firefighters displayed a survival advantage for localized stage (adjusted hazard ratio [aHR] = 0.53; 95%CI: 0.34-0.82). However, for regional or distant stages, firefighters aged 65 and above exhibited a higher risk of death (aHR = 1.84; 95% CI: 1.18-2.86) than the general population. Conclusion: Firefighters experience enhanced prostate cancer survival, primarily in cases diagnosed at

localized stages, likely due to increased PSA testing. Nonetheless, for regional or distant stage, survival among older firefighters' lags behind that of the general population. Further investigations are warranted to unravel factors influencing the development of aggressive disease beyond PSA and Gleason scores in this population, as well as to assess the impact of a higher rate of surgical treatment on firefighters' quality of life.

Keywords:

Florida, firefighters, occupational exposure, prostate cancer, survival

<u>Original Lay Summary</u>

Prostate cancer survival among US firefighters, who undergo regular medical check-ups and have unique exposures, has not been well-studied. Using population-based Florida cancer registry data, this retrospective study compared 1058 firefighter prostate cancer cases to patients with prostate cancer in the general population, finding significantly better five-year survival (96.1% vs. 94.2%) in firefighters. Firefighters were diagnosed earlier, had more localized cancers, and underwent surgery more often. However, older firefighters with regional or distant-stage cancer faced a higher risk of death. Enhanced survival in firefighters may be due to increased PSA testing, but further research is needed to understand factors influencing aggressive disease development and the impact of surgical treatments on their quality of life.

**Assessment Category**	**Score**	**Assessment Category**	**Score**	**Assessment Category**	**Score**
Comprehension Score Rater 1	5	Gunning Fog Score	17.0	Words	121
Comprehension Score Rater 2	5	SMOG Index	11.9	Complex words	34
Reading Age (years)	19.5	Coleman Liau Index	17.6	Percentage of complex words	28.1
Flesch-Kincaid Reading Ease	29.3	Automated Readability Index	12.8	Average words per sentence	15.1
Flesch-Kincaid Grade Level	12.9	Sentences	8	Average syllables per word	1.9

Lay Summary (Simple Prompt)

Firefighters are exposed to hazardous substances and undergo frequent medical check-ups, which may influence their health outcomes, including cancer survival. This study examined prostate cancer survival among Florida firefighters compared to the general population. Using data from 2004 to 2014, researchers analyzed over 1,000 firefighter cases and 150,000 cases from the general public. The results showed that firefighters were diagnosed at younger ages, had more early-stage prostate cancers, and received surgery more often. Their survival rates for localized prostate cancer were better, likely due to frequent medical screenings. However, older firefighters with advanced disease had worse survival outcomes than the general population. These findings highlight the benefits of early detection but also raise concerns about the treatment and survival of firefighters with aggressive prostate cancer. Further research is needed to understand the long-term effects of firefighting on cancer progression and quality of life.

**Assessment Category**	**Score**	**Assessment Category**	**Score**	**Assessment Category**	**Score**
Comprehension Score Rater 1	5	Gunning Fog Score	16.6	Words	148
Comprehension Score Rater 2	5	SMOG Index	12.3	Complex words	36
Reading Age (years)	20.5	Coleman Liau Index	18.0	Percentage of complex words	24.3
Flesch-Kincaid Reading Ease	32.0	Automated Readability Index	14.8	Average words per sentence	18.5
Flesch-Kincaid Grade Level	13.4	Sentences	8	Average syllables per word	1.8

Lay Summary (Extended Prompt)

Prostate cancer is one of the most common cancers in men, but survival rates can vary based on job-related factors. Firefighters face unique risks due to exposure to harmful chemicals and frequent medical check-ups. This study compared prostate cancer survival in Florida firefighters with the general population. Using cancer registry data from 2004 to 2014, researchers found that firefighters were diagnosed at younger ages and had more cases detected at early, treatable stages. They also had better overall survival rates for localized cancer, likely due to regular medical screenings. However, older firefighters with advanced cancer had a higher risk of death compared to others. These findings suggest that early detection helps firefighters survive prostate cancer, but more research is needed to understand why some develop aggressive disease and how treatment choices impact their long-term well-being. This could improve cancer care for firefighters and similar high-risk professions.

**Assessment Category**	**Score**	**Assessment Category**	**Score**	**Assessment Category**	**Score**
Comprehension Score Rater 1	5	Gunning Fog Score	16.0	Words	151
Comprehension Score Rater 2	5	SMOG Index	11.7	Complex words	33
Reading Age (years)	19.5	Coleman Liau Index	16.3	Percentage of complex words	21.9
Flesch-Kincaid Reading Ease	39.8	Automated Readability Index	13.7	Average words per sentence	18.9
Flesch-Kincaid Grade Level	12.4	Sentences	8	Average syllables per word	1.8

Manuscript 159

Kano H, Kadono Y, Naito R, Makino T, Iwamoto H, Yaegashi H, et al. Salvage Androgen Deprivation Therapy as Potential Treatment for Recurrence after Robot-Assisted Radical Prostatectomy. Cancers [Internet]. 2024 Mar 27;16(7):1304. Available from: http://dx.doi.org/10.3390/cancers16071304

Abstract

Background: The efficacy of intermittent androgen deprivation therapy (ADT) for biochemical recurrence (BCR) after robot-assisted radical prostatectomy (RARP) is unknown, and its usefulness in Japanese practice needs to be investigated.

Methods: We conducted a retrospective analysis of 85 patients who underwent RARP and were selected for intermittent ADT for postoperative recurrence at Kanazawa University Hospital between 2009 and 2019. Intermittent ADT was administered for 2 years. If prostate-specific antigen levels increased post-treatment, intermittent ADT was reinitiated. The median follow-up period was 47 months.

Results: The 73 patients had completed the initial course of ADT, and 12 were under initial ADT. The 5-year castration-resistant prostate-cancer-free survival rates, cancer-specific survival, and overall survival were 92.7%, 98.3%, and 94.7%, respectively. A subgroup analysis of 69 patients who completed intermittent ADT was conducted to evaluate the BCR rate following initial ADT. The 5-year BCR-free survival rate was 53.2%. Multivariate analysis identified testosterone ≤ 0.03 ng/mL during ADT as the sole predictor of BCR after ADT.

Conclusions: Salvage intermittent ADT may be an effective treatment option for BCR after RARP. In addition, it would be useful to confirm strong testosterone suppression as a criterion for transition to intermittent therapy.

Keywords:

intermittent androgen deprivation therapy, recurrence, robot-assisted radical prostatectomy, salvage treatment, testosterone

<u>Original Lay Summary</u>

The efficacy of intermittent androgen deprivation therapy (ADT) for biochemical recurrence (BCR) after robot-assisted radical prostatectomy (RARP) is unknown, and its usefulness in Japanese practice needs to be investigated. A retrospective analysis of 85 patients who underwent RARP at Kanazawa University Hospital between 2009 and 2019 and were selected for intermittent ADT for postoperative recurrence was analyzed. The 5-year castration-resistant prostate-cancer-free survival rates, cancer-specific survival, and overall survival were 92.7%, 98.3%, and 94.7%, respectively. A subgroup analysis of 69 patients who completed initial ADT was conducted to evaluate the rate of BCR following initial ADT. The 5-year BCR-free survival rate was 53.2%. Multivariate analysis identified testosterone ≤ 0.03 ng/mL during ADT as the sole predictor of BCR after ADT. ADT may be applicable for BCR after RARP, and strong testosterone suppression could lead to better outcomes.

**Assessment Category**	**Score**	**Assessment Category**	**Score**	**Assessment Category**	**Score**
Comprehension Score Rater 1	5	Gunning Fog Score	16.5	Words	149
Comprehension Score Rater 2	5	SMOG Index	11.6	Complex words	48
Reading Age (years)	18.5	Coleman Liau Index	14.5	Percentage of complex words	32.2
Flesch-Kincaid Reading Ease	30.7	Automated Readability Index	9.0	Average words per sentence	12.4
Flesch-Kincaid Grade Level	12.1	Sentences	12	Average syllables per word	1.9

Lay Summary (Simple Prompt)

Prostate cancer can return even after surgery, requiring further treatment. This study examines whether a type of hormone therapy called intermittent androgen deprivation therapy (ADT) can help manage cancer recurrence after robot-assisted prostate removal. Researchers analyzed 85 patients who received this treatment, where hormones that fuel cancer growth were temporarily suppressed. The results showed that most patients remained free from aggressive cancer progression for five years, and those whose testosterone levels were extremely low during treatment had a lower risk of further recurrence. These findings suggest that intermittent ADT could be a useful treatment option for men facing cancer recurrence after surgery. Additionally, measuring testosterone levels during treatment may help doctors decide when to pause or restart therapy. This research could improve post-surgery treatment strategies, potentially leading to better outcomes for patients with recurrent prostate cancer.

**Assessment Category**	**Score**	**Assessment Category**	**Score**	**Assessment Category**	**Score**
Comprehension Score Rater 1	5	Gunning Fog Score	16.6	Words	138
Comprehension Score Rater 2	5	SMOG Index	12.0	Complex words	30
Reading Age (years)	21.5	Coleman Liau Index	20.2	Percentage of complex words	21.7
Flesch-Kincaid Reading Ease	28.0	Automated Readability Index	17.2	Average words per sentence	19.7
Flesch-Kincaid Grade Level	14.2	Sentences	7	Average syllables per word	1.9

Lay Summary (Extended Prompt)

Prostate cancer can return after surgery, even if the prostate has been removed. Doctors often use hormone therapy, called androgen deprivation therapy (ADT), to lower testosterone levels and slow cancer growth. This study looked at 85 patients who had surgery but later needed ADT to control their cancer. Researchers at Kanazawa University Hospital tested a method called intermittent ADT, where treatment is given for two years, paused, and restarted if needed. After five years, most patients remained cancer-free, and survival rates were high. The study found that patients with very low testosterone during treatment had better outcomes. These findings suggest that intermittent ADT could be an effective option for managing cancer recurrence after surgery. Doctors may also use testosterone levels to decide when to start and stop treatment, helping patients avoid unnecessary side effects while keeping cancer under control.

**Assessment Category**	**Score**	**Assessment Category**	**Score**	**Assessment Category**	**Score**
Comprehension Score Rater 1	5	Gunning Fog Score	12.4	Words	140
Comprehension Score Rater 2	5	SMOG Index	9.7	Complex words	22
Reading Age (years)	18.5	Coleman Liau Index	16.7	Percentage of complex words	15.7
Flesch-Kincaid Reading Ease	39.8	Automated Readability Index	13.3	Average words per sentence	17.5
Flesch-Kincaid Grade Level	12.1	Sentences	8	Average syllables per word	1.8

Manuscript 160

Liyanage C, Fernando A, Chamberlain A, Moradi A, Batra J. RNA m6a Methylation Regulator Expression in Castration-Resistant Prostate Cancer Progression and Its Genetic Associations. Cancers [Internet]. 2024 Mar 27;16(7):1303. Available from: http://dx.doi.org/10.3390/cancers16071303

Abstract

N6-methyladenosine (m6A) methylation, a prevalent epitranscriptomic modification, plays a crucial role in regulating mRNA expression, stability, and translation in mammals. M6A regulators have gained attention for their potential implications in tumorigenesis and clinical applications, such as cancer diagnosis and therapeutics. The existing literature predominantly addresses m6A regulators in the context of primary prostate cancer (PCa). However, a notable gap in the knowledge emerges regarding the dynamic expression patterns of these regulators as PCa progresses towards the castration-resistant stage (CRPC). Employing sequential window acquisition of all theoretical mass spectra (SWATH-MS) and RNAseq analysis, we comprehensively profiled the expression of 27 m6A regulators in hormone/androgen-dependent and -independent PCa cell lines, revealing distinct clustering between tumor and adjacent normal prostate tissues. High-grade PCa tumors demonstrated the upregulation of METTL3, RBM15B, and HNRNAPA2B1 and the downregulation of ZC3H13, NUDT21, and FTO. Notably, we identified six m6A regulators associated with PCa survival. Additionally, association analysis of the PCa-associated risk loci in the cancer genome atlas program (TCGA) data unveiled genetic variations near the WTAP, HNRNPA2B1, and FTO genes as significant expression quantitative trait loci. In summary, our study unraveled abnormalities in m6A regulator expression in PCa progression, elucidating their association with PCa risk loci. Considering the heterogeneity within the PCa phenotypes and treatment responses, our findings suggest that prognostic stratification based on m6A regulator expression could enhance PCa diagnosis and prognosis.

Keywords:

androgen-targeted therapy, castration-resistant prostate cancer, m6A methylation, prostate cancer, single-nucleotide polymorphisms

Original Lay Summary

The study investigates N6-methyladenosine (m6A) methylation, the most common RNA modification that influences cell function by regulating RNA's function and metabolism. The reversible control of m6A modification involves methyltransferases ("writers") and demethylases ("erasers"), with the interactions facilitated by RNA-binding proteins ("readers"). The dysregulation of these processes is implicated in various diseases, including cancers. Using mass spectrometry and RNA sequencing, this study profiles the dynamic m6A regulator expression patterns in prostate cancer. It identifies specific regulators whose expression significantly changes during cancer development, therapy resistance, and progression to advanced stages like castration-resistant prostate cancer. The research also uncovers significant correlations between dysregulated m6A regulators and genetic polymorphisms linked to prostate cancer risk. In conclusion, this study underscores the clinical importance of m6A-regulators, offering potential molecular targets for diagnostic and prognostic assessments in prostate cancer.

**Assessment Category**	**Score**	**Assessment Category**	**Score**	**Assessment Category**	**Score**
Comprehension Score Rater 1	5	Gunning Fog Score	23.0	Words	137
Comprehension Score Rater 2	5	SMOG Index	15.8	Complex words	53
Reading Age (years)	24.0	Coleman Liau Index	24.0	Percentage of complex words	38.7
Flesch-Kincaid Reading Ease	-3.8	Automated Readability Index	20.2	Average words per sentence	19.6
Flesch-Kincaid Grade Level	18.7	Sentences	7	Average syllables per word	2.3

Lay Summary (Simple Prompt)

Prostate cancer can become resistant to standard hormone-based treatments, making it more difficult to manage. Scientists are exploring how small chemical changes in RNA, known as m6A methylation, may influence this resistance. In this study, researchers examined the activity of 27 key m6A-related genes in prostate cancer cells, comparing those that still respond to hormone therapy with those that do not. They found significant differences in gene expression between normal and cancerous prostate tissues, identifying specific genes that may play a role in disease progression. Some of these genes were also linked to inherited genetic variations that might increase prostate cancer risk. These findings could help improve how prostate cancer is diagnosed and treated, potentially leading to more personalized treatment strategies based on a patient's unique genetic profile. Understanding these molecular changes may also offer new targets for future therapies.

**Assessment Category**	**Score**	**Assessment Category**	**Score**	**Assessment Category**	**Score**
Comprehension Score Rater 1	5	Gunning Fog Score	16.8	Words	142
Comprehension Score Rater 2	5	SMOG Index	12.2	Complex words	31
Reading Age (years)	20.5	Coleman Liau Index	17.6	Percentage of complex words	21.8
Flesch-Kincaid Reading Ease	36.7	Automated Readability Index	15.4	Average words per sentence	20.3
Flesch-Kincaid Grade Level	13.2	Sentences	7	Average syllables per word	1.8

<u>Lay Summary (Extended Prompt)</u>

Prostate cancer is one of the most common cancers in men, and in advanced stages, it can become resistant to hormone therapy, making it harder to treat. Scientists are studying a type of chemical modification called m6A methylation, which affects how genes are turned on or off in cancer cells. In this study, researchers analyzed the activity of 27 m6A regulators in different prostate cancer types. They discovered key differences between early and advanced prostate cancer, identifying specific genes that may influence cancer growth and patient survival. They also found genetic variations linked to these regulators, which could help explain why some patients respond differently to treatments. Understanding these changes may lead to better ways to diagnose and predict prostate cancer progression, helping doctors personalize treatments for patients in the future.

**Assessment Category**	**Score**	**Assessment Category**	**Score**	**Assessment Category**	**Score**
Comprehension Score Rater 1	5	Gunning Fog Score	16.1	Words	131
Comprehension Score Rater 2	5	SMOG Index	11.6	Complex words	24
Reading Age (years)	20.5	Coleman Liau Index	16.7	Percentage of complex words	18.32
Flesch-Kincaid Reading Ease	38.7	Automated Readability Index	15.5	Average words per sentence	21.8
Flesch-Kincaid Grade Level	13.3	Sentences	6	Average syllables per word	1.7

Manuscript 161

Vaccarin C, Mapanao AK, Deberle LM, Becker AE, Borgna F, Marzaro G, et al. Design and Preclinical Evaluation of a Novel Prostate-Specific Membrane Antigen Radioligand Modified with a Transthyretin Binder. Cancers [Internet]. 2024 Mar 23;16(7):1262. Available from: http://dx.doi.org/10.3390/cancers16071262

Abstract

Transthyretin binders have previously been used to improve the pharmacokinetic properties of small-molecule drug conjugates and could, thus, be utilized for radiopharmaceuticals as an alternative to the widely explored "albumin binder concept". In this study, a novel PSMA ligand modified with a transthyretin-binding entity (TB-01) was synthesized and labeled with lutetium-177 to obtain [177Lu]Lu-PSMA-TB-01. A high and specific uptake of [177Lu]Lu-PSMA-TB-01 was found in PSMA-positive PC-3 PIP cells (69 ± 3% after 4 h incubation), while uptake in PSMA-negative PC-3 flu cells was negligible (177Lu]Lu-PSMA-TB-01 to transthyretin than to human serum albumin. Biodistribution studies in PC-3 PIP/flu tumor-bearing mice confirmed the enhanced blood retention of [177Lu]Lu-PSMA-TB-01 (16 ± 1% IA/g at 1 h p.i.), which translated to a high tumor uptake (69 ± 13% IA/g at 4 h p.i.) with only slow wash-out over time (31 ± 8% IA/g at 96 h p.i.), while accumulation in the PC-3 flu tumor and non-targeted normal tissue was reasonably low. Further optimization of the radioligand design would be necessary to fine-tune the biodistribution and enable its use for therapeutic purposes. This study was the first of this kind and could motivate the use of the "transthyretin binder concept" for the development of future radiopharmaceuticals.

Keywords:

PC-3 PIP tumor cells, PSMA radioligand, lutetium-177, plasma protein binding, prostate cancer, transthyretin binder

<u>Original Lay Summary</u>

Prostate-specific membrane antigen (PSMA)-targeting radioligands have been used clinically to treat metastatic prostate cancer. To deliver a sufficient radiation dose to eliminate cancer cells, the accumulation of the radioligand in the tumor should be high. Therefore, small-molecular-weight molecules have been modified with albumin binders to enhance their blood circulation time and, hence, increase the tumor uptake. The goal of this study was to investigate the utility of a transthyretin binder (TB-01) to achieve high tumor uptake of the resultant PSMA radioligand. Indeed, the newly developed [177Lu]Lu-PSMA-TB-01 demonstrated enhanced blood circulation as compared to the clinically employed [177Lu]Lu-PSMA-617. Consequently, a significantly higher accumulation of activity was observed in the tumor tissue, while activity retention in off-target tissue was only higher in the kidneys, but otherwise in the same range as for [177Lu]Lu-PSMA-617.

**Assessment Category**	**Score**	**Assessment Category**	**Score**	**Assessment Category**	**Score**
Comprehension Score Rater 1	4	Gunning Fog Score	19.0	Words	144
Comprehension Score Rater 2	4	SMOG Index	13.7	Complex words	34
Reading Age (years)	21.5	Coleman Liau Index	15.6	Percentage of complex words	23.6
Flesch-Kincaid Reading Ease	24.4	Automated Readability Index	15.7	Average words per sentence	24.0
Flesch-Kincaid Grade Level	15.8	Sentences	6	Average syllables per word	1.9

Lay Summary (Simple Prompt)

Prostate cancer is one of the most common cancers in men, and researchers are continuously looking for better ways to detect and treat it. In this study, scientists developed a new type of radioactive compound designed to target prostate cancer cells more effectively. This compound attaches to a protein called PSMA, which is found in high amounts on prostate cancer cells. By adding a special component that binds to a blood protein called transthyretin, the researchers aimed to improve how long the compound stays in the bloodstream, which could help deliver radiation more precisely to cancer cells. Laboratory tests and experiments in mice showed that the compound successfully attached to prostate cancer cells while avoiding healthy tissue. These promising results suggest that further improvements could lead to a more effective treatment for prostate cancer. This study introduces a new approach that could inspire future developments in cancer-targeting radiopharmaceuticals.

**Assessment Category**	**Score**	**Assessment Category**	**Score**	**Assessment Category**	**Score**
Comprehension Score Rater 1	5	Gunning Fog Score	14.2	Words	149
Comprehension Score Rater 2	5	SMOG Index	10.1	Complex words	21
Reading Age (years)	19.5	Coleman Liau Index	16.8	Percentage of complex words	14.1
Flesch-Kincaid Reading Ease	42.1	Automated Readability Index	15.3	Average words per sentence	21.3
Flesch-Kincaid Grade Level	12.7	Sentences	7	Average syllables per word	1.7

Lay Summary (Extended Prompt)

Prostate cancer is one of the most common cancers in men, and doctors use special imaging and treatment methods to detect and fight it. This study explores a new way to improve a type of cancer-targeting drug called a radioligand, which delivers radiation directly to cancer cells. Researchers modified a prostate-specific radioligand by adding a special protein binder called a transthyretin binder, which helps the drug stay in the blood longer. Tests in cancer cells and mice showed that the new drug design resulted in high and long-lasting accumulation in prostate cancer tumors while minimizing unwanted buildup in healthy tissues. These findings suggest that transthyretin binders could be a promising tool for making radioligand therapies more effective. With further improvements, this approach could help develop better treatments for prostate cancer in the future.

**Assessment Category**	**Score**	**Assessment Category**	**Score**	**Assessment Category**	**Score**
Comprehension Score Rater 1	5	Gunning Fog Score	15.5	Words	136
Comprehension Score Rater 2	5	SMOG Index	11.1	Complex words	22
Reading Age (years)	19.5	Coleman Liau Index	15.8	Percentage of complex words	16.2
Flesch-Kincaid Reading Ease	38.9	Automated Readability Index	15.2	Average words per sentence	22.7
Flesch-Kincaid Grade Level	13.5	Sentences	6	Average syllables per word	1.7

Manuscript 162

Sayan M, Tuac Y, Akgul M, Pratt GK, Rowan MD, Akbulut D, et al. Prognostic Significance of the Cribriform Pattern in Prostate Cancer: Clinical Outcomes and Genomic Alterations. Cancers [Internet]. 2024 Mar 22;16(7):1248. Available from: http://
dx.doi.org/10.3390/cancers16071248

Abstract

Purpose: Given the diverse clinical progression of prostate cancer (PC) and the evolving significance of histopathological factors in its management, this study aimed to explore the impact of cribriform pattern 4 (CP4) on clinical outcomes in PC patients and examine its molecular characteristics.

Methods: This retrospective study analyzed data from The Cancer Genome Atlas (TCGA) database and included PC patients who underwent radical prostatectomy (RP) and had pathology slides available for the assessment of CP4. A multivariable competing risk regression analysis was used to assess the association between CP4 and progression-free survival (PFS) while adjusting for established PC prognostic factors. The frequency of genomic alterations was compared between patients with and without CP4 using the Fisher's exact test.

Results: Among the 394 patients analyzed, 129 (32.74%) had CP4. After a median follow-up of 40.50 months (IQR: 23.90, 65.60), the presence of CP4 was significantly associated with lower PFS (AHR, 1.84; 95% CI, 1.08 to 3.114; p = 0.023) after adjusting for covariates. Seven hub genes-KRT13, KRT5, KRT15, COL17A1, KRT14, KRT16, and TP63-had significantly lower mRNA expression levels in patients with CP4 compared to those without.

Conclusions: PC patients with CP4 have distinct genomic alterations and are at a high risk of disease progression following RP. Therefore, these patients may benefit

from additional post-RP treatments and should be the subject of a prospective randomized clinical trial.

Keywords:

cribriform pattern, genomic alterations, progression-free survival, prostate cancer, radical prostatectomy

<u>Original Lay Summary</u>

Prostate cancer is a condition with varying outcomes, and understanding its progression is vital for treatment. Our research focused on a specific pattern observed in tumor cells, known as cribriform pattern 4 (CP4), which can indicate how aggressive a cancer might be. By studying patients who had undergone surgery to remove the prostate gland, we found that those with CP4 tended to have a higher risk of cancer recurrence. Additionally, we discovered certain genes that behaved differently in patients with CP4, suggesting unique genetic changes associated with this pattern. Our findings suggest that patients with CP4 might need more intense treatment after surgery and could help doctors decide on the best course of action. Importantly, our research provides new insights that could lead to better management of prostate cancer, aiming to improve patient outcomes.

**Assessment Category**	**Score**	**Assessment Category**	**Score**	**Assessment Category**	**Score**
Comprehension Score Rater 1	5	Gunning Fog Score	15.8	Words	134
Comprehension Score Rater 2	5	SMOG Index	11.3	Complex words	23
Reading Age (years)	19.5	Coleman Liau Index	15.7	Percentage of complex words	17.2
Flesch-Kincaid Reading Ease	41.5	Automated Readability Index	15.0	Average words per sentence	22.3
Flesch-Kincaid Grade Level	13.0	Sentences	6	Average syllables per word	1.7

Lay Summary (Simple Prompt)

Prostate cancer is a complex disease, and doctors use different features of cancer cells to predict how aggressive it may be. One such feature is the cribriform pattern, a specific arrangement of cancer cells that may indicate a higher risk of the disease worsening. In this study, we analyzed medical data from prostate cancer patients who had undergone surgery to see whether this pattern was linked to poorer outcomes. We found that patients with the cribriform pattern were more likely to experience cancer progression after surgery. Additionally, we identified specific genetic changes in these patients, which could help explain why their cancer behaves more aggressively. These findings suggest that patients with this pattern may need additional treatments after surgery. Future research should focus on testing new therapies for these high-risk patients to improve their chances of long-term recovery.

**Assessment Category**	**Score**	**Assessment Category**	**Score**	**Assessment Category**	**Score**
Comprehension Score Rater 1	4	Gunning Fog Score	14.9	Words	140
Comprehension Score Rater 2	5	SMOG Index	10.7	Complex words	24
Reading Age (years)	19.5	Coleman Liau Index	16.1	Percentage of complex words	17.1
Flesch-Kincaid Reading Ease	42.7	Automated Readability Index	14.1	Average words per sentence	20.0
Flesch-Kincaid Grade Level	12.3	Sentences	7	Average syllables per word	1.7

Lay Summary (Extended Prompt)

Prostate cancer can behave very differently from one patient to another, making it important to find features that help predict how the disease will progress. This study focused on a specific growth pattern in prostate cancer called the cribriform pattern, which may indicate a higher risk of cancer returning after surgery. Researchers analyzed data from 394 patients who had their prostate removed and compared those with and without this pattern. They found that patients with the cribriform pattern had a greater chance of cancer coming back. The study also identified genetic differences in these patients, suggesting that their cancer may be more aggressive at a molecular level. These findings highlight the need for closer monitoring and possibly additional treatments for affected patients. Understanding these risks could help doctors make better treatment decisions and improve outcomes for men with prostate cancer.

**Assessment Category**	**Score**	**Assessment Category**	**Score**	**Assessment Category**	**Score**
Comprehension Score Rater 1	4	Gunning Fog Score	14.6	Words	140
Comprehension Score Rater 2	4	SMOG Index	10.5	Complex words	23
Reading Age (years)	19.5	Coleman Liau Index	16.8	Percentage of complex words	16.4
Flesch-Kincaid Reading Ease	42.1	Automated Readability Index	14.6	Average words per sentence	20.0
Flesch-Kincaid Grade Level	12.4	Sentences	7	Average syllables per word	1.7

Manuscript 163

Kougioumtzopoulou A, Syrigos N, Zygogianni A, Georgakopoulos I, Platoni K, Patatoukas G, et al. Comprehensive 3DCRT Hypofractionated Radiotherapy Schedule for Localized Prostate Adenocarcinoma in the Era of IMRT: Dosimetric and Endoscopic Analysis. Cancers [Internet]. 2024 Mar 18;16(6):1192. Available from: http://dx.doi.org/10.3390/cancers16061192

Abstract

Background: Moderate hypofractionated radiotherapy (MHRT) has emerged as the preferred treatment modality for localized prostate cancer based on randomized controlled studies regarding efficacy and toxicity using contemporary radiotherapy techniques. In the setting of MHRT, available data on dosimetric parameters and late rectal toxicity are limited. Aim: To present the effects of MHRT on late rectal toxicity while conducting an extensive dosimetric analysis in conjunction with rectoscopy results. Methods: This is a prospective study including patients with intermediate-risk prostate adenocarcinoma. All patients were treated with MHRT 44 Gy in 16 fractions to the seminal vesicles and to the prostate, followed by a sequential boost to the prostate alone of 16.5 Gy in 6 fractions delivered with three-dimensional conformal radiation therapy (3DCRT). Acute and late toxicity were assessed. Endoscopy was performed at baseline, every 3 months post-therapy for the first year, and every 6 months for the year after. The Vienna Rectoscopy Score (VRS) was used to assess rectal mucosal injury related to radiotherapy. Dosimetric analysis for the rectum, rectal wall, and its subsegments (upper, mid, and low 1/3) was performed. Results: Between September 2015 and December 2019, 20 patients enrolled. Grade 1 late gastrointestinal toxicity occurred in 10% of the patients, whereas 5% had a grade ≥2. Twelve months post radiotherapy: 4 (20%) patients had VRS 1; 2 (10%) patients had VRS 2; 1(5%) patient had VRS 3. 24 months post radiotherapy, VRS 1 was observed in 4 patients (20%) and VRS 2 in 3 (15%) patients. The dosimetric analysis demonstrated noticeable variations between the rectum, rectal wall, and rectal wall subsegments. The dosimetric analysis of the rectum, rectal wall, and its

mid and low segments with respect to rectoscopy findings showed that the higher dose endpoints V52.17Gy and V56.52Gy are associated with rectal mucosal injury. Conclusions: A thorough delineation of the rectal wall and its subsegments, together with the dosimetric analysis of these structures, may reduce late rectal toxicity. Dosimetric parameters such as V52.17Gy and V56.52Gy were identified to have a significant impact on rectal mucosal injury; additional dose endpoint validation and its relation to late GI toxicity is needed.

Keywords:

Vienna Rectoscopy Score (VRS), dose volume histogram (DVH), dosimetry, intensity-modulated radiation therapy (IMRT), moderate hypofractionated radiotherapy, prostate cancer, rectal wall, rectoscopy, three-dimensional conformal radiation therapy (3DCRT)

<u>Original Lay Summary</u>

Moderate hypofractionated radiotherapy (MHRT) has emerged as the preferred treatment modality for localized prostate cancer. The aim of this prospective study was to evaluate late rectal toxicity while performing a comprehensive dosimetric analysis in conjunction with rectoscopy results in the setting of MHRT. We confirmed in an homogeneous population (20 patients) that when 3DCRT is employed, delineation of rectal wall and its subsegments can provide a more in depth accurate dosimetric analysis. Furthermore, we identified that dose endpoints V52.17Gy and V56.52Gy, when hypofractionated schedule (2.75 Gy per fraction) is used, may have a significant impact on rectal mucosal injury. Implementing these variables in clinical practice may result in a decrease rate of late rectal toxicity; more data is needed to assist in further validation of this conclusion.

**Assessment Category**	**Score**	**Assessment Category**	**Score**	**Assessment Category**	**Score**
Comprehension Score Rater 1	5	Gunning Fog Score	16.3	Words	130
Comprehension Score Rater 2	5	SMOG Index	11.7	Complex words	33
Reading Age (years)	19.5	Coleman Liau Index	16.3	Percentage of complex words	25.4
Flesch-Kincaid Reading Ease	29.0	Automated Readability Index	12.3	Average words per sentence	16.3
Flesch-Kincaid Grade Level	13.3	Sentences	8	Average syllables per word	1.9

Lay Summary (Simple Prompt)

Prostate cancer is often treated with radiotherapy, but it can sometimes cause side effects in the rectum, leading to discomfort or complications. This study explored a specific type of radiation treatment, called moderate hypofractionated radiotherapy, which delivers radiation in fewer sessions while maintaining effectiveness. Researchers closely monitored patients using endoscopy to check for any rectal damage and analyzed radiation doses in different rectal areas. The results showed that while most patients had mild or no long-term rectal side effects, higher radiation doses in certain areas were linked to increased tissue damage. These findings suggest that carefully adjusting radiation delivery, particularly to specific rectal regions, may help minimize side effects. This research provides valuable insights for improving radiation treatment plans, ultimately aiming to enhance the safety and comfort of prostate cancer patients undergoing radiotherapy.

**Assessment Category**	**Score**	**Assessment Category**	**Score**	**Assessment Category**	**Score**
Comprehension Score Rater 1	5	Gunning Fog Score	19.1	Words	134
Comprehension Score Rater 2	5	SMOG Index	13.7	Complex words	34
Reading Age (years)	23.5	Coleman Liau Index	20.3	Percentage of complex words	25.4
Flesch-Kincaid Reading Ease	11.8	Automated Readability Index	18.6	Average words per sentence	22.3
Flesch-Kincaid Grade Level	17.2	Sentences	6	Average syllables per word	2.0

Lay Summary (Extended Prompt)

Prostate cancer is often treated with radiation therapy, but high doses can sometimes cause long-term side effects in the rectum. This study looked at a specific type of radiation treatment called moderate hypofractionated radiotherapy (MHRT) to see how it affects the rectum over time. Researchers treated 20 patients with localized prostate cancer using a precise radiation technique and then monitored their rectal health with regular endoscopic exams. They found that while most patients had minimal side effects, higher doses of radiation in certain areas of the rectum were linked to tissue damage. These findings suggest that carefully planning radiation doses and targeting specific parts of the rectum more precisely may help reduce side effects. This research provides valuable insights for doctors who aim to make radiation therapy for prostate cancer safer and more effective while minimizing long-term complications for patients.

**Assessment Category**	**Score**	**Assessment Category**	**Score**	**Assessment Category**	**Score**
Comprehension Score Rater 1	5	Gunning Fog Score	17.4	Words	142
Comprehension Score Rater 2	5	SMOG Index	12.5	Complex words	28
Reading Age (years)	21.5	Coleman Liau Index	16.9	Percentage of complex words	19.7
Flesch-Kincaid Reading Ease	32.1	Automated Readability Index	16.6	Average words per sentence	23.7
Flesch-Kincaid Grade Level	14.7	Sentences	6	Average syllables per word	1.8

Manuscript 164

Calik J, Calik K, Sauer N, Zdzisław B, Giedziun P, Mackiewicz J, et al. Impact of Bisphosphonate Therapy on Oral Health in Patients with Breast and Prostate Cancer and Bone Metastases: A Comprehensive Study. Cancers [Internet]. 2024 Mar 11;16(6):1124. Available from: http://dx.doi.org/10.3390/cancers16061124

Abstract

This study investigates the impact of bisphosphonate therapy on the stomatognathic system in 80 patients with cancer of the breast and prostate with bone metastases. Bisphosphonates are integral for managing skeletal complications in these malignancies but are associated with bisphosphonate-related osteonecrosis of the jaw (BRONJ), affecting 0.8-18.5% of patients. BRONJ manifests with pain, neuropathy, tissue swelling, mucosal ulceration, tooth mobility, and abscesses, yet its pathogenesis remains elusive, complicating risk prediction. The research employed comprehensive dental and radiological evaluations. Dental status was assessed using DMFT and OHI-S indices, Eichner's classification, and clinical periodontal measurements like the pocket depth (PD), clinical attachment loss (CAL), and modified Sulcus Bleeding Index (mSBI). A radiological analysis included panoramic X-rays for radiomorphometric measurements and TMJ lateral radiographs. Results indicated a significant decline in oral hygiene in patients with cancer after bisphosphonate therapy, marked by increased DMFT and OHI-S scores. Periodontal health also showed deterioration, with increased PD and CAL readings. The incidence of BRONJ symptoms was noted, although exact figures are not quantified in this abstract. The study also revealed changes in radiomorphometric parameters, suggesting bisphosphonates' impact on bone density and structure. No substantial alterations were observed in TMJ function, indicating a need for extended observation to understand bisphosphonates' long-term effects on the stomatognathic system. These findings highlight the importance of continuous dental monitoring and prophylaxis in patients undergoing bisphosphonate therapy.

Implementing meticulous oral care protocols is essential for mitigating BRONJ risk and managing the complex oral health challenges in patients with cancer.

Keywords:

BRONJ (bisphosphonate-related osteonecrosis of the jaw), bisphosphonates, bone metastases, oral hygiene, stomatognathic system

Original Lay Summary

This study delves into bisphosphonate therapy's effects on oral health in 80 breast and prostate cancer patients with bone metastases. Bisphosphonates, crucial for managing skeletal complications, are linked to bisphosphonate-related osteonecrosis of the jaw (BRONJ), presenting in 0.8–18.5% of patients with symptoms like pain, tissue swelling, and tooth mobility. Through dental and radiological assessments—including DMFT and OHI-S indices, periodontal measurements, and panoramic X-rays—the research uncovers a notable decline in oral hygiene post-therapy, evidenced by worsened DMFT, OHI-S, PD, and CAL scores. Additionally, radiological changes hint at bisphosphonates' impact on bone density and structure. Despite no significant changes in TMJ function, the findings underscore the need for ongoing dental care and risk mitigation strategies for BRONJ, emphasizing the vital role of regular dental monitoring and proactive oral hygiene practices in the comprehensive care of cancer patients undergoing bisphosphonate therapy.

Assessment Category	Score	Assessment Category	Score	Assessment Category	Score
Comprehension Score Rater 1	5	Gunning Fog Score	18.2	Words	145
Comprehension Score Rater 2	5	SMOG Index	13.1	Complex words	36
Reading Age (years)	21.5	Coleman Liau Index	18.9	Percentage of complex words	24.8
Flesch-Kincaid Reading Ease	20.7	Automated Readability Index	16.7	Average words per sentence	20.7
Flesch-Kincaid Grade Level	15.5	Sentences	7	Average syllables per word	2.0

Lay Summary (Simple Prompt)

Many patients with breast or prostate cancer receive bisphosphonate therapy to prevent bone complications caused by cancer spreading to the bones. However, this treatment can lead to serious dental problems, including a painful condition called bisphosphonate-related osteonecrosis of the jaw (BRONJ). In this study, we examined 80 patients to assess how bisphosphonates affect oral health. We conducted thorough dental check-ups and X-rays to evaluate gum disease, tooth decay, and jawbone health. Our findings revealed that bisphosphonate therapy is linked to a decline in oral hygiene, increased gum disease, and changes in jawbone structure. While jaw joint function remained largely unaffected, long-term effects remain uncertain. These results highlight the need for regular dental care and early preventive measures in cancer patients receiving bisphosphonates. By improving oral health monitoring, we may reduce complications and enhance patients' quality of life.

**Assessment Category**	**Score**	**Assessment Category**	**Score**	**Assessment Category**	**Score**
Comprehension Score Rater 1	5	Gunning Fog Score	14.4	Words	141
Comprehension Score Rater 2	5	SMOG Index	10.5	Complex words	26
Reading Age (years)	19.5	Coleman Liau Index	18.0	Percentage of complex words	18.4
Flesch-Kincaid Reading Ease	38.3	Automated Readability Index	14.4	Average words per sentence	17.6
Flesch-Kincaid Grade Level	12.3	Sentences	8	Average syllables per word	1.8

Lay Summary (Extended Prompt)

People with breast and prostate cancer that has spread to their bones often receive bisphosphonate therapy to protect their bones. However, this treatment can sometimes lead to serious jaw problems, such as pain, infections, and tooth loss. In this study, researchers examined 80 patients to understand how bisphosphonates affect oral health. They looked at dental hygiene, gum health, and jawbone structure using dental exams and X-rays. The findings showed that patients on bisphosphonates had worse oral hygiene, more gum disease, and changes in bone structure, though their jaw joint function remained stable. These results highlight the need for regular dental care and early prevention strategies for patients receiving this therapy. By improving oral hygiene, the risk of jaw complications could be reduced, leading to better overall health and quality of life for cancer patients undergoing bisphosphonate treatment.

**Assessment Category**	**Score**	**Assessment Category**	**Score**	**Assessment Category**	**Score**
Comprehension Score Rater 1	5	Gunning Fog Score	14.3	Words	138
Comprehension Score Rater 2	5	SMOG Index	10.3	Complex words	22
Reading Age (years)	19.5	Coleman Liau Index	16.9	Percentage of complex words	15.9
Flesch-Kincaid Reading Ease	47.1	Automated Readability Index	14.6	Average words per sentence	19.7
Flesch-Kincaid Grade Level	11.6	Sentences	7	Average syllables per word	1.7

Manuscript 165

Dondi F, Antonelli A, Suardi N, Treglia G, Bertagna F. The Role of PSMA PET Imaging in the Classification of the Risk of Prostate Cancer Patients: A Systematic Review on the Insights to Guide an Active Surveillance Approach. Cancers [Internet]. 2024 Mar 11;16(6):1122. Available from: http://dx.doi.org/10.3390/cancers16061122

Abstract

Background: active surveillance (AS) is a suitable strategy for patients with prostate cancer (PCa). Prostate-specific membrane antigen (PSMA) positron emission tomography (PET) imaging is an established tool used to assess PCa. The aim of this review was to evaluate the role of PSMA imaging to guide correct risk-based classification and the AS approach in PCa patients.

Methods: The Scopus, Embase, Web of Science, Cochrane Library, and PubMed/MEDLINE databases were screened to find relevant published articles.

Results: 1774 articles were revealed with the literature search. A total of 1764 articles were excluded after applying exclusion criteria (data not within the field of interest, preclinical papers, conference proceedings, reviews, or editorials). Ten studies were finally included in the review, revealing that PSMA PET could have the ability to guide risk-based classification of PCa and the choice of AS, and to guide the execution of biopsies for the research of high-grade PCa, therefore precluding AS.

Conclusion: this systematic review underlined a possible role of PSMA PET imaging in patients with PCa by correctly re-classifying them on the basis of their risk and guiding AS.

Keywords:

PET, PSMA, active surveillance, nuclear medicine, positron emission tomography, prostate cancer, prostate-specific membrane antigen

<u>Original Lay Summary</u>

The prognosis of prostate cancer (PCa) patients and their best therapeutic approach are related to the risk-based classification of this neoplasm since subjects with higher risk could have a higher incidence of recurrence. In addition, patients with low- and intermediate-risk PCa could benefit from active surveillance (AS). Prostate-specific membrane antigen (PSMA) positron emission tomography (PET) imaging has demonstrated its value for the assessment, prognostic role, and ability to guide the therapy of PCa. The aim of this systematic review was to assess the role of PSMA PET in guiding the correct classification of low-to-intermediate risk PCa subjects and the AS approach. Insights on the value of this imaging modality in these settings have emerged; however, further research in this field is necessary to clearly define the role of PSMA PET.

**Assessment Category**	**Score**	**Assessment Category**	**Score**	**Assessment Category**	**Score**
Comprehension Score Rater 1	5	Gunning Fog Score	19.1	Words	135
Comprehension Score Rater 2	5	SMOG Index	13.6	Complex words	28
Reading Age (years)	21.5	Coleman Liau Index	14.9	Percentage of complex words	20.7
Flesch-Kincaid Reading Ease	34.0	Automated Readability Index	16.6	Average words per sentence	27.0
Flesch-Kincaid Grade Level	15.2	Sentences	5	Average syllables per word	1.7

Lay Summary (Simple Prompt)

Prostate cancer is a common condition, and not all cases require immediate treatment. Some patients can safely follow an approach called active surveillance, which involves careful monitoring instead of surgery or radiation. However, accurately identifying which patients can benefit from this approach remains a challenge. This study reviewed existing research on a special imaging technique called PSMA PET, which helps doctors see prostate cancer more clearly. After analyzing many studies, the researchers found that PSMA PET could improve how patients are classified based on their risk level. This could help doctors make better decisions about whether active surveillance is a safe option or if a biopsy is needed to check for more aggressive cancer. These findings suggest that PSMA PET imaging may play an important role in guiding personalized care for prostate cancer patients, potentially reducing unnecessary treatments while ensuring high-risk cases receive proper attention.

**Assessment Category**	**Score**	**Assessment Category**	**Score**	**Assessment Category**	**Score**
Comprehension Score Rater 1	5	Gunning Fog Score	16.3	Words	146
Comprehension Score Rater 2	5	SMOG Index	11.8	Complex words	29
Reading Age (years)	20.5	Coleman Liau Index	17.7	Percentage of complex words	19.9
Flesch-Kincaid Reading Ease	34.4	Automated Readability Index	15.8	Average words per sentence	20.9
Flesch-Kincaid Grade Level	13.6	Sentences	7	Average syllables per word	1.8

Lay Summary (Extended Prompt)

Prostate cancer is one of the most common cancers in men, and not all cases require immediate treatment. Some patients can safely choose active surveillance, which means closely monitoring the cancer instead of undergoing surgery or radiation. However, deciding who should follow this approach can be challenging. This study reviewed existing research on a special type of imaging called PSMA PET scans, which help doctors see prostate cancer more clearly. By analyzing 10 high-quality studies, the researchers found that PSMA PET imaging may improve how doctors assess prostate cancer risk. This means it could help identify which patients are good candidates for active surveillance and which may need treatment sooner. More precise risk assessment could prevent unnecessary treatments while ensuring that aggressive cancers are treated early. These findings suggest that PSMA PET imaging could play a key role in improving prostate cancer care in the future.

**Assessment Category**	**Score**	**Assessment Category**	**Score**	**Assessment Category**	**Score**
Comprehension Score Rater 1	5	Gunning Fog Score	13.9	Words	147
Comprehension Score Rater 2	5	SMOG Index	10.1	Complex words	24
Reading Age (years)	18.5	Coleman Liau Index	16.2	Percentage of complex words	16.3
Flesch-Kincaid Reading Ease	47.2	Automated Readability Index	13.4	Average words per sentence	18.4
Flesch-Kincaid Grade Level	11.2	Sentences	8	Average syllables per word	1.7

Manuscript 166

Smith SC, Wobker SE. Intraductal Carcinoma of the Prostate versus Simulants: A Differential Diagnosis Growing in Clinical Impact. Cancers [Internet]. 2024 Mar 8;16(6):1097. Available from: http://dx.doi.org/10.3390/cancers16061097

Abstract

Despite its first recognition even longer ago, in the past nearly 20 years, intraductal carcinoma of the prostate has become a standard histopathologic reporting parameter conveying a strong negative prognostic factor for prostatic adenocarcinoma. When seen at biopsy, intraductal carcinoma of the prostate is associated with risk for aggressive prostatectomy outcomes, including frequently high-grade, high-stage, high-volume disease, with increased risk for recurrence and progression. Multiple organizations, including the uropathology subspecialty societies to the World Health Organization, recognize and recommend reporting the presence of intraductal carcinoma, whether sampled in "pure" form or present with concomitant invasive adenocarcinoma. Moreover, emerging scholarship relates intraductal carcinoma to higher prevalence of homologous recombination repair deficiency mutations in prostatic adenocarcinoma, whether somatic or germline, which serve as indications for approved targeted therapies. Taken together, this is a diagnosis for the histopathologist not to miss. In view of these elevated stakes and the opportunity to further precision medicine, this review details neoplastic and non-neoplastic simulants in the differential diagnosis of intraductal carcinoma of the prostate.

Keywords:

cribriform pattern, gleason grading, grade groups, intraductal carcinoma of the prostate, prostate cancer

<u>Original Lay Summary</u>

Though intraductal carcinoma of the prostate has been observed and recognized for years, more recently this process has emerged, to become simultaneously both a consensus, major prognostic factor and controversial parameter as relates to potential impact on the histologic grading in prostate cancer. Due to the increasing incorporation of intraductal carcinoma into risk stratification approaches and management considerations in this disease, the differential diagnoses of this lesion have become of increasing importance. Herein, we review the background of recognition of and evolution of the criteria for intraductal carcinoma, emphasizing its differential diagnoses, both neoplastic and non-neoplastic. We furthermore recommend useful immunohistochemical markers to help correctly recognize and diagnose this key process.

**Assessment Category**	**Score**	**Assessment Category**	**Score**	**Assessment Category**	**Score**
Comprehension Score Rater 1	4	Gunning Fog Score	24.8	Words	112
Comprehension Score Rater 2	4	SMOG Index	17.7	Complex words	38
Reading Age (years)	24.0	Coleman Liau Index	21.3	Percentage of complex words	33.9
Flesch-Kincaid Reading Ease	-2.1	Automated Readability Index	22.2	Average words per sentence	28.0
Flesch-Kincaid Grade Level	20.5	Sentences	4	Average syllables per word	2.1

Lay Summary (Simple Prompt)

Prostate cancer is one of the most common cancers in men, and correctly identifying aggressive forms is crucial for choosing the best treatment. One important but often overlooked type is intraductal carcinoma of the prostate, which is strongly linked to aggressive disease and a higher risk of recurrence. This study highlights the importance of distinguishing this cancer type from other look-alike conditions under the microscope. Recognizing intraductal carcinoma can help doctors predict how aggressive a patient's cancer may be and determine if genetic testing or targeted treatments are needed. Since leading health organizations now recommend reporting this condition, improving diagnostic accuracy can enhance patient care. This review explains the key features that help pathologists tell intraductal carcinoma apart from non-cancerous or less aggressive conditions, ensuring that patients receive the most precise diagnosis and best possible treatment options.

**Assessment Category**	**Score**	**Assessment Category**	**Score**	**Assessment Category**	**Score**
Comprehension Score Rater 1	5	Gunning Fog Score	19.6	Words	139
Comprehension Score Rater 2	5	SMOG Index	14.1	Complex words	36
Reading Age (years)	22.5	Coleman Liau Index	19.0	Percentage of complex words	25.9
Flesch-Kincaid Reading Ease	22.6	Automated Readability Index	18.0	Average words per sentence	23.2
Flesch-Kincaid Grade Level	15.9	Sentences	6	Average syllables per word	1.9

Lay Summary (Extended Prompt)

Prostate cancer is one of the most common cancers in men, and diagnosing it correctly is crucial for effective treatment. A specific type of prostate cancer, called intraductal carcinoma of the prostate (IDC-P), is linked to more aggressive disease and a higher risk of cancer returning. Doctors and scientists now recognize IDC-P as an important warning sign, and its presence can influence treatment decisions. Some cases of IDC-P may also indicate genetic changes that make patients eligible for advanced targeted therapies. However, diagnosing IDC-P can be challenging because it looks similar to other non-cancerous and cancerous conditions under the microscope. This study explores how pathologists can accurately distinguish IDC-P from these look-alike conditions. By improving diagnostic accuracy, this research helps ensure that patients receive the right treatment at the right time, leading to better outcomes and advancing precision medicine for prostate cancer care.

**Assessment Category**	**Score**	**Assessment Category**	**Score**	**Assessment Category**	**Score**
Comprehension Score Rater 1	5	Gunning Fog Score	17.6	Words	150
Comprehension Score Rater 2	5	SMOG Index	12.7	Complex words	34
Reading Age (years)	20.5	Coleman Liau Index	16.1	Percentage of complex words	22.7
Flesch-Kincaid Reading Ease	36.8	Automated Readability Index	14.8	Average words per sentence	21.4
Flesch-Kincaid Grade Level	13.5	Sentences	7	Average syllables per word	1.8

Manuscript 167

Lawen T, Ilie G, Mason R, Rendon R, Spooner J, Champion E, et al. Six-Month Prostate Cancer Empowerment Program (PC-PEP) Improves Urinary Function: A Randomized Trial. Cancers [Internet]. 2024 Feb 27;16(5):958. Available from: http://dx.doi.org/10.3390/cancers16050958

<u>Abstract</u>

Purpose: This is a secondary analysis examining a six-month home-based Prostate Cancer-Patient Empowerment Program (PC-PEP) on patient-reported urinary, bowel, sexual, and hormonal function in men with curative prostate cancer (PC) against standard of care. Methods: In a crossover clinical trial, 128 men scheduled for PC surgery (n = 62) or radiotherapy with/without hormones (n = 66) were randomized to PC-PEP (n = 66) or waitlist-control and received the standard of care for 6 months, and then PC-PEP to the end of the year. PC-PEP included daily emails with video instructions, aerobic and strength training, dietary guidance, stress management, and social support, with an initial PFMT nurse consultation. Over 6 months, participants in the PC-PEP received optional text alerts (up to three times daily) reminding them to follow the PFMT video program, encompassing relaxation, quick-twitch, and endurance exercises; compliance was assessed weekly. Participants completed baseline, 6, and 12-month International Prostate Symptom Score (IPSS) and Expanded Prostate Cancer Index Composite (EPIC) questionnaires. Results: At 6 months, men in the PC-PEP reported improved urinary bother (IPSS, p = 0.004), continence (EPIC, p p = 0.008) compared to controls, with sustained urinary continence benefits at 12 months (p = 0.002). Surgery patients in the waitlist-control group had 3.5 (95% CI: 1.2, 10, p = 0.024) times and 2.3 (95% CI: 0.82, 6.7, p = 0.11) times higher odds of moderate to severe urinary problems compared to PC-PEP at 6 and 12 months, respectively. Conclusions: PC-PEP significantly improves lower urinary tract symptoms, affirming its suitability for clinical integration alongside established mental health benefits in men with curative prostate cancer.

Keywords:

behavioural intervention, cancer survivorship, eHealth, erectile dysfunction, incontinence, patient education and activation, pelvic floor muscle training (PFMT), prostate cancer, sexual function, urinary function

<u>Original Lay Summary</u>

In this research, we explore the effectiveness of a unique home-based 6-month comprehensive program designed to empower prostate cancer patients during their treatment. The Prostate Cancer-Patient Empowerment Program (PC-PEP) aims to enhance the quality of life for men undergoing curative treatment for prostate cancer, focusing on improving urologic function. Through a blend of physical activities, dietary education and recommendations, stress management, and social support, PC-PEP offers an innovative approach to patient care. This study rigorously assesses the impact of PC-PEP through a detailed 6-month comparison with standard care, evaluating its potential to significantly improve patient-reported outcomes. Our findings hold the promise of reshaping patient care strategies, presenting a potentially valuable addition to clinical practices for men battling prostate cancer, with the hope of improving not just their physical well-being but also their overall quality of life.

**Assessment Category**	**Score**	**Assessment Category**	**Score**	**Assessment Category**	**Score**
Comprehension Score Rater 1	5	Gunning Fog Score	21.5	Words	145
Comprehension Score Rater 2	5	SMOG Index	15.6	Complex words	37
Reading Age (years)	24.0	Coleman Liau Index	17.8	Percentage of complex words	25.5
Flesch-Kincaid Reading Ease	16.4	Automated Readability Index	19.9	Average words per sentence	29.0
Flesch-Kincaid Grade Level	18.2	Sentences	5	Average syllables per word	1.9

Lay Summary (Simple Prompt)

Men treated for prostate cancer often experience urinary problems, which can affect their quality of life. This study tested whether a six-month home-based program called the Prostate Cancer-Patient Empowerment Program (PC-PEP) could help improve urinary function compared to standard care. The program included daily guidance on physical activity, diet, stress management, and pelvic floor muscle exercises, with regular reminders to encourage participation. The results showed that men who followed the program had significantly better urinary control and fewer bothersome symptoms after six months compared to those who did not. These benefits were maintained for up to a year. Men who did not participate in the program were more likely to have moderate to severe urinary issues after surgery. These findings suggest that PC-PEP could be a valuable addition to standard prostate cancer care, helping men regain bladder control and improve their well-being after treatment.

**Assessment Category**	**Score**	**Assessment Category**	**Score**	**Assessment Category**	**Score**
Comprehension Score Rater 1	5	Gunning Fog Score	14.4	Words	150
Comprehension Score Rater 2	5	SMOG Index	10.5	Complex words	23
Reading Age (years)	19.5	Coleman Liau Index	16.1	Percentage of complex words	15.3
Flesch-Kincaid Reading Ease	37.9	Automated Readability Index	14.8	Average words per sentence	21.4
Flesch-Kincaid Grade Level	13.3	Sentences	7	Average syllables per word	1.7

Lay Summary (Extended Prompt)

Many men experience bladder control problems after prostate cancer treatment, which can affect their daily lives. This study tested a six-month home program called the Prostate Cancer-Patient Empowerment Program (PC-PEP) to see if it could help improve urinary function. The program included daily video instructions for pelvic floor exercises, fitness routines, stress management, and dietary guidance, along with social support. Men in the program had better bladder control after six months compared to those who received standard care. These benefits lasted for at least a year. Those who did not participate were more likely to have moderate to severe urinary problems. The findings suggest that PC-PEP is an effective way to support men recovering from prostate cancer treatment, helping them regain bladder control and improve their quality of life. This program could be a valuable addition to routine care for prostate cancer patients.

**Assessment Category**	**Score**	**Assessment Category**	**Score**	**Assessment Category**	**Score**
Comprehension Score Rater 1	5	Gunning Fog Score	11.7	Words	147
Comprehension Score Rater 2	5	SMOG Index	8.5	Complex words	17
Reading Age (years)	17.5	Coleman Liau Index	15.5	Percentage of complex words	11.6
Flesch-Kincaid Reading Ease	47.8	Automated Readability Index	12.8	Average words per sentence	18.4
Flesch-Kincaid Grade Level	11.2	Sentences	8	Average syllables per word	1.7

Manuscript 168

Wang R, Hu P, Wang F, Lyu J, Ou Y, Edderkaoui M, et al. Spontaneous Fusion with Transformed Mesenchymal Stromal Cells Results in Complete Heterogeneity in Prostate Cancer Cells. Cancers [Internet]. 2024 Feb 27;16(5):951. Available from: http://dx.doi.org/10.3390/cancers16050951

Abstract

Tumor cells gain advantages in growth and survival by acquiring genotypic and phenotypic heterogeneity. Interactions with bystander cells in the tumor microenvironment contribute to the progression of heterogeneity. We have shown that fusion between tumor and bystander cells is one form of interaction, and that tumor-bystander cell fusion has contrasting effects. By trapping fusion hybrids in the heterokaryon or synkaryon state, tumor-bystander cell fusion prevents the progression of heterogeneity. However, if trapping fails, fusion hybrids will resume replication to form derivative clones with diverse genomic makeups and behavioral phenotypes. To determine the characteristics of bystander cells that influence the fate of fusion hybrids, we co-cultured prostate mesenchymal stromal cell lines and their spontaneously transformed sublines with LNCaP as well as HPE-15 prostate cancer cells. Subclones derived from cancer-stromal fusion hybrids were examined for genotypic and phenotypic diversifications. Both stromal cell lines were capable of fusing with cancer cells, but only fusion hybrids with the transformed stromal subline generated large numbers of derivative subclones. Each subclone had distinct cell morphologies and growth behaviors and was detected with complete genomic hybridization. The health conditions of the bystander cell compartment play a crucial role in the progression of tumor cell heterogeneity.

Keywords:

bystander cells, cancer–stromal hybridization, cell fusion, heterogeneity progression, stromal cell transformation, tumor cells

<u>Original Lay Summary</u>

We have previously demonstrated that the interaction between cancer cells and bystander cells in the tumor microenvironment can promote the development of tumor cell heterogeneity. This heterogeneity is the basis for cancer progression, therapeutic resistance, and distant metastasis. In this study, we co-cultured prostate cancer cells with stromal cells to study the process of cancer–bystander cell interaction. Compared to primary stromal cells, spontaneously transformed stromal cells had significantly enhanced activity in inducing heterogeneity in cancer cells. By tracking the individual cancer–stromal interactions, we found that the transformed stromal sublines had a high tendency to fuse with cancer cells, forming cancer–stromal fusion hybrids that had the ability to proliferate. Hybridization between individual cancer cells and stromal cells resulted in genotypic and phenotypic diversification. This study indicates that the pathophysiologic status of the bystander cell compartment is crucial to the progression of tumor cell heterogeneity.

**Assessment Category**	**Score**	**Assessment Category**	**Score**	**Assessment Category**	**Score**
Comprehension Score Rater 1	4	Gunning Fog Score	19.1	Words	144
Comprehension Score Rater 2	4	SMOG Index	13.6	Complex words	39
Reading Age (years)	23.5	Coleman Liau Index	21.0	Percentage of complex words	27.08
Flesch-Kincaid Reading Ease	6.8	Automated Readability Index	18.3	Average words per sentence	20.57
Flesch-Kincaid Grade Level	17.4	Sentences	7	Average syllables per word	2.12

Lay Summary (Simple Prompt)

This study explores how prostate cancer cells become more diverse, which helps them grow and survive. The researchers focused on the role of nearby supportive cells, known as mesenchymal stromal cells, in this process. They discovered that cancer cells can fuse with these stromal cells, and this fusion can lead to different outcomes. When fusion hybrids are "trapped," they stop changing, which prevents further diversity. However, if this trapping fails, the fused cells create new subclones with different shapes, behaviors, and genetic features. Interestingly, fusion with healthy stromal cells did not produce many new subclones, but fusion with altered, transformed stromal cells led to a wide variety of cancer cell types. This suggests that the condition of the surrounding stromal cells plays a key role in how prostate cancer cells evolve. Understanding this process could help develop new strategies to control cancer progression.

**Assessment Category**	**Score**	**Assessment Category**	**Score**	**Assessment Category**	**Score**
Comprehension Score Rater 1	5	Gunning Fog Score	12.5	Words	144
Comprehension Score Rater 2	5	SMOG Index	9.0	Complex words	19
Reading Age (years)	17.5	Coleman Liau Index	16.0	Percentage of complex words	13.19
Flesch-Kincaid Reading Ease	54.0	Automated Readability Index	13.0	Average words per sentence	18.0
Flesch-Kincaid Grade Level	10.2	Sentences	8	Average syllables per word	1.59

Lay Summary (Extended Prompt)

Cancer cells can change and become more diverse, which helps them grow and survive. This study looked at how prostate cancer cells interact with nearby support cells, called stromal cells. Sometimes, these cells merge together, creating hybrid cells. We found that when cancer cells fused with normal stromal cells, the hybrids didn't grow much. But when they fused with stromal cells that had already changed (transformed), the hybrids grew rapidly and formed many new types of cancer cells, each with different shapes and behaviors. This shows that the health of nearby support cells can influence how cancer changes and spreads. Understanding this process may help find new ways to control cancer growth in the future.

**Assessment Category**	**Score**	**Assessment Category**	**Score**	**Assessment Category**	**Score**
Comprehension Score Rater 1	5	Gunning Fog Score	9.4	Words	115
Comprehension Score Rater 2	5	SMOG Index	6.4	Complex words	8
Reading Age (years)	15.5	Coleman Liau Index	14.4	Percentage of complex words	6.96
Flesch-Kincaid Reading Ease	70.2	Automated Readability Index	10.9	Average words per sentence	16.43
Flesch-Kincaid Grade Level	7.5	Sentences	7	Average syllables per word	1.42

Manuscript 169

Al Saffar H, Chen DC, Delgado C, Ingvar J, Hofman MS, Lawrentschuk N, et al. The Current Landscape of Prostate-Specific Membrane Antigen (PSMA) Imaging Biomarkers for Aggressive Prostate Cancer. Cancers [Internet]. 2024 Feb 26;16(5):939. Available from: http://dx.doi.org/10.3390/cancers16050939

Abstract

The review examines the vital role of prostate-specific membrane antigen (PSMA) positron emission tomography/computed tomography (PET/CT) in the diagnosis, staging, and treatment of prostate cancer (PCa). It focuses on the superior diagnostic abilities of PSMA PET/CT for identifying both nodal and distant PCa, and its potential as a prognostic indicator for biochemical recurrence and overall survival. Additionally, we focused on the variability of PSMA's expression and its impact on personalised treatment, particularly the use of [177Lu] Lu-PSMA-617 radioligand therapy. This review emphasises the essential role of PSMA PET/CT in enhancing treatment approaches, improving patient outcomes, and reducing unnecessary interventions, positioning it as a key element in personalised PCa management.

Keywords:

PSMA PET/CT, aggressive prostate cancer, biochemical recurrence, prostate-specific membrane antigen, uro-oncology

<u>Original Lay Summary</u>

The review explores the critical role of prostate-specific membrane antigen (PSMA) PET/CT imaging in diagnosing, staging, and treating prostate cancer. PSMA PET/CT offers superior diagnostic capabilities for identifying prostate cancer's spread, with potential as a prognostic indicator for the disease's recurrence and survival. It highlights PSMA's variability in expression, impacting personalised treatment plans, notably in radioligand therapy with [177Lu] Lu-PSMA-617. This technology enhances treatment strategies, improves outcomes, and reduces unnecessary interventions, marking a significant advancement in personalised prostate cancer management.

**Assessment Category**	**Score**	**Assessment Category**	**Score**	**Assessment Category**	**Score**
Comprehension Score Rater 1	5	Gunning Fog Score	22.3	Words	83
Comprehension Score Rater 2	5	SMOG Index	15.5	Complex words	29
Reading Age (years)	24.0	Coleman Liau Index	23.1	Percentage of complex words	34.94
Flesch-Kincaid Reading Ease	-0.8	Automated Readability Index	20.0	Average words per sentence	20.75
Flesch-Kincaid Grade Level	18.5	Sentences	4	Average syllables per word	2.2

Lay Summary (Simple Prompt)

This review explores how a special imaging technique called PSMA PET/CT helps in diagnosing and managing aggressive prostate cancer. PSMA PET/CT is highly effective at detecting cancer both in nearby lymph nodes and in distant parts of the body. It also helps predict the chances of cancer returning and the patient's overall survival. The review highlights how the levels of PSMA in cancer cells can vary, which is important when deciding on personalized treatments, such as targeted radioligand therapy with [177Lu] Lu-PSMA-617. By using PSMA PET/CT, doctors can improve treatment decisions, avoid unnecessary procedures, and achieve better outcomes for patients. This imaging technique is becoming a crucial tool in the personalized care of men with prostate cancer.

**Assessment Category**	**Score**	**Assessment Category**	**Score**	**Assessment Category**	**Score**
Comprehension Score Rater 1	5	Gunning Fog Score	15.7	Words	119
Comprehension Score Rater 2	5	SMOG Index	11.3	Complex words	23
Reading Age (years)	19.5	Coleman Liau Index	15.2	Percentage of complex words	19.33
Flesch-Kincaid Reading Ease	43.1	Automated Readability Index	13.3	Average words per sentence	19.83
Flesch-Kincaid Grade Level	12.2	Sentences	6	Average syllables per word	1.7

Lay Summary (Extended Prompt)

Prostate cancer is a common disease in men, and finding it early can help save lives. This study looks at a special imaging test called PSMA PET/CT, which helps doctors see prostate cancer more clearly than traditional methods. It can detect cancer that has spread to other parts of the body, even when it's hard to find. This test also helps predict if the cancer might come back after treatment. We explored how differences in PSMA levels affect treatment choices, especially with a targeted therapy called radioligand therapy. By using PSMA PET/CT, doctors can create more personalized treatment plans, avoid unnecessary procedures, and improve patient outcomes. This technology is becoming an important tool in the fight against aggressive prostate cancer.

**Assessment Category**	**Score**	**Assessment Category**	**Score**	**Assessment Category**	**Score**
Comprehension Score Rater 1	5	Gunning Fog Score	11.9	Words	120
Comprehension Score Rater 2	5	SMOG Index	8.6	Complex words	15
Reading Age (years)	16.5	Coleman Liau Index	14.8	Percentage of complex words	12.5
Flesch-Kincaid Reading Ease	52.7	Automated Readability Index	11.6	Average words per sentence	17.14
Flesch-Kincaid Grade Level	10.2	Sentences	7	Average syllables per word	1.62

Manuscript 170

Karwacki J, Gurwin A, Jaworski A, Jarocki M, Stodolak M, Dłubak A, et al. Association of Lymphovascular Invasion with Lymph Node Metastases in Prostate Cancer—Lateralization Concept. Cancers [Internet]. 2024 Feb 25;16(5):925. Available from: http://dx.doi.org/10.3390/cancers16050925

Abstract

Background: Lymphovascular invasion (LVI) is a vital but often overlooked prognostic factor in prostate cancer. As debates on lymphadenectomy's overtreatment emerge, understanding LVI laterality gains importance. This study pioneers the investigation into PCa, aiming to uncover patterns that could influence tailored surgical strategies in the future.

Methods: Data from 96 patients with both LVI and lymph node invasion (LNI) were retrospectively analyzed. All participants underwent radical prostatectomy (RP) with modified-extended pelvic lymph node dissection (mePLND). All specimens underwent histopathological examination. The assessment of LVI was conducted separately for the right and left lobes of the prostate. Associations within subgroups were assessed using U-Mann-Whitney and Kruskal-Wallis tests, as well as Kendall's tau-b coefficient, yielding p-values and odds ratios (ORs).

Results: Out of the 96 patients, 61 (63.5%) exhibited exclusive left-sided lymphovascular invasion (LVI), 24 (25.0%) had exclusive right-sided LVI, and 11 (11.5%) showed bilateral LVI. Regarding nodal involvement, 23 patients (24.0%) had LNI solely on the left, 25 (26.0%) exclusively on the right, and 48 (50.0%) on both sides. A significant correlation was observed between lateralized LVI and lateralized LNI ($p < 0.001$), particularly in patients with right-sided LVI only. LN-positive patients with left-sided LVI tended to have higher pT stages ($p = 0.047$) and increased odds ratios (OR) of bilateral LNI (OR = 2.795; 95% confidence interval [CI]: 1.231-6.348) compared to those with exclusive right-sided LVI (OR = 0.692; 95% CI: 0.525-0.913).

Conclusions: Unilateral LVI correlates with ipsilateral LNI in PCa patients with positive LNs, notably in cases of exclusively right-sided LVI. Left-sided LVI associates with higher pT stages and a higher percentage of bilateral LNI cases.

Keywords:

histopathological examination, lymph node invasion, lymphovascular invasion, nodal involvement lateralization, pelvic lymph node dissection, prostate cancer, radical prostatectomy

Original Lay Summary

Prostate cancer (PCa) patients often face uncertainties in treatment decisions, particularly regarding lymphadenectomy. This study, involving 96 PCa patients, explores the significance of lymphovascular invasion (LVI) laterality in influencing lymph node invasion (LNI) patterns. Out of these patients, 63.5% exhibited LVI exclusively on the left, 25.0% on the right, and 11.5% on both sides. Significant correlations were observed between LVI laterality and lymph node involvement ($p < 0.001$), especially on the right side. Left-sided LVI correlated with higher cancer stage ($p = 0.047$) and greater odds of bilateral lymph node involvement. This pioneering study emphasizes the need for future prospective, multi-center investigations, ideally incorporating preoperative LVI assessment, to refine PCa treatment decisions.

**Assessment Category**	**Score**	**Assessment Category**	**Score**	**Assessment Category**	**Score**
Comprehension Score Rater 1	5	Gunning Fog Score	14.9	Words	117
Comprehension Score Rater 2	5	SMOG Index	9.8	Complex words	31
Reading Age (years)	18.5	Coleman Liau Index	17.1	Percentage of complex words	26.5
Flesch-Kincaid Reading Ease	31.9	Automated Readability Index	10.2	Average words per sentence	10.64
Flesch-Kincaid Grade Level	11.5	Sentences	11	Average syllables per word	1.94

Lay Summary (Simple Prompt)

This study explored how the spread of prostate cancer through blood and lymph vessels (called lymphovascular invasion, or LVI) is linked to cancer spreading to nearby lymph nodes. Researchers examined tissue samples from 96 prostate cancer patients who had surgery to remove their prostate and nearby lymph nodes. They discovered that when LVI was found on one side of the prostate, the cancer was more likely to spread to lymph nodes on the same side. Interestingly, patients with LVI on the left side had more advanced tumors and a higher chance of cancer spreading to lymph nodes on both sides compared to those with LVI on the right. These findings could help doctors predict how prostate cancer spreads and guide more personalized surgical decisions in the future.

**Assessment Category**	**Score**	**Assessment Category**	**Score**	**Assessment Category**	**Score**
Comprehension Score Rater 1	5	Gunning Fog Score	13.3	Words	127
Comprehension Score Rater 2	5	SMOG Index	8.3	Complex words	10
Reading Age (years)	17.5	Coleman Liau Index	13.5	Percentage of complex words	7.87
Flesch-Kincaid Reading Ease	59.2	Automated Readability Index	14.7	Average words per sentence	25.4
Flesch-Kincaid Grade Level	11.3	Sentences	5	Average syllables per word	1.44

<u>Lay Summary (Extended Prompt)</u>

This study looks at how prostate cancer spreads, focusing on a factor called lymphovascular invasion (LVI), where cancer cells are found in the body's small blood or lymph vessels. We wanted to see if LVI on one side of the prostate is linked to cancer spreading to lymph nodes on the same side. We examined tissue from 96 prostate cancer patients who had surgery to remove the prostate and nearby lymph nodes. We found that when LVI was present on just one side of the prostate, cancer often spread to lymph nodes on that same side. Interestingly, cancer on the left side was more likely to be advanced and to spread to both sides of the lymph nodes compared to cancer on the right. These findings may help doctors better predict how prostate cancer spreads and make more personalized decisions about surgery and treatment.

**Assessment Category**	**Score**	**Assessment Category**	**Score**	**Assessment Category**	**Score**
Comprehension Score Rater 1	5	Gunning Fog Score	12.1	Words	144
Comprehension Score Rater 2	5	SMOG Index	7.2	Complex words	9
Reading Age (years)	16.5	Coleman Liau Index	11.4	Percentage of complex words	6.25
Flesch-Kincaid Reading Ease	64.4	Automated Readability Index	12.4	Average words per sentence	24.0
Flesch-Kincaid Grade Level	10.2	Sentences	6	Average syllables per word	1.4

Manuscript 171

Piątkowska D, Klimaszewska-Wiśniewska A, Kosińska A, Wujec R, Grzanka D, Durślewicz J. Ubiquitin B, Ubiquitin C, and β-Catenin as Promising Diagnostic and Prognostic Tools in Prostate Cancer. Cancers [Internet]. 2024 Feb 23;16(5):902. Available from: http://dx.doi.org/10.3390/cancers16050902

Abstract

Prostate cancer (PC) is a major global public health concern, imposing a significant burden on men and ranking as the second most prevalent malignancy. This study delves into the intricate world of ubiquitination processes and expression regulation, with a specific focus on understanding the roles of ubiquitin B (UBB), ubiquitin C (UBC), and β-Catenin in PC development. We thoroughly analyze the expression profiles of UBB, UBC, and β-Catenin, investigating their interactions and associations with clinical and histopathological data. These findings offer valuable insights into their potential as robust prognostic markers and their significance for patient survival. Our research uncovers the upregulation of UBB and UBC expression in PC tissues, and an even more pronounced expression in lymph node metastases, highlighting their pivotal roles in PC progression. Moreover, we identify a compelling correlation between high UBB and UBC levels and diminished overall survival in PC patients, emphasizing their clinical relevance. Additionally, we observe a significant reduction in membranous β-Catenin expression in PC tissues. Importantly, abnormal β-Catenin expression is strongly associated with shorter survival in PC patients and serves as a significant, independent prognostic factor for patient outcomes. Kaplan-Meier survival analysis indicates that patients with tumors characterized by simultaneous UBB and aberrant β-Catenin expression exhibit the poorest overall survival. These collective insights underline the clinical importance of evaluating UBB, UBC, and β-Catenin as combined prognostic markers in PC.

Keywords:

UBB, UBC, prognostic markers, prostate cancer, ubiquitination, β-Catenin

<u>Original Lay Summary</u>

Prostate cancer (PC) is a major global health concern, ranking as the second most common malignancy in men. This study investigates the role of ubiquitin B (UBB), ubiquitin C (UBC), and β-Catenin in PC development, focusing on their expression and interactions. By analyzing their profiles in PC tissues and lymph node metastases, the research identifies elevated UBB and UBC levels, particularly in metastatic cases, emphasizing their crucial roles in disease progression. Furthermore, the study reveals a correlation between high UBB and UBC expression and reduced overall survival in PC patients. Additionally, abnormal β-Catenin expression is associated with shorter survival and serves as a significant independent prognostic factor. The combined assessment of UBB, UBC, and β-Catenin emerges as a valuable approach for predicting patient outcomes in PC.

**Assessment Category**	**Score**	**Assessment Category**	**Score**	**Assessment Category**	**Score**
Comprehension Score Rater 1	5	Gunning Fog Score	18.5	Words	129
Comprehension Score Rater 2	5	SMOG Index	13.9	Complex words	35
Reading Age (years)	21.5	Coleman Liau Index	16.4	Percentage of complex words	27.13
Flesch-Kincaid Reading Ease	23.0	Automated Readability Index	15.1	Average words per sentence	21.5
Flesch-Kincaid Grade Level	15.4	Sentences	6	Average syllables per word	1.91

<u>Lay Summary (Simple Prompt)</u>

Prostate cancer is one of the most common cancers in men worldwide. This study explores the roles of three specific proteins—ubiquitin B (UBB), ubiquitin C (UBC), and β-Catenin—to understand how they influence the development and progression of prostate cancer. Researchers examined prostate cancer tissue samples to measure the levels of these proteins and see how they relate to patient outcomes. They found that UBB and UBC levels were higher in cancerous tissues, especially in cases where the cancer had spread to lymph nodes. High levels of these proteins were linked to shorter survival times. Additionally, changes in β-Catenin levels were associated with worse outcomes. The combination of high UBB and abnormal β-Catenin expression predicted the poorest survival rates. These findings suggest that testing for UBB, UBC, and β-Catenin in prostate cancer patients could help doctors better predict disease progression and guide treatment decisions.

**Assessment Category**	**Score**	**Assessment Category**	**Score**	**Assessment Category**	**Score**
Comprehension Score Rater 1	5	Gunning Fog Score	12.8	Words	147
Comprehension Score Rater 2	5	SMOG Index	10.1	Complex words	24
Reading Age (years)	18.5	Coleman Liau Index	15.7	Percentage of complex words	16.33
Flesch-Kincaid Reading Ease	46.0	Automated Readability Index	12.9	Average words per sentence	18.38
Flesch-Kincaid Grade Level	11.4	Sentences	8	Average syllables per word	1.68

<u>Lay Summary (Extended Prompt)</u>

Prostate cancer is one of the most common cancers in men worldwide. This study looks at three specific proteins—ubiquitin B (UBB), ubiquitin C (UBC), and β-Catenin—to see how they might help doctors diagnose and predict the course of prostate cancer. We found that UBB and UBC levels were higher in prostate cancer tissues, especially in cancers that had spread to the lymph nodes. Patients with high levels of these proteins tended to have shorter survival times. Additionally, changes in β-Catenin were linked to worse outcomes. When both UBB and β-Catenin were abnormal, survival rates were the lowest. These results suggest that testing for these proteins could help identify aggressive forms of prostate cancer early, allowing for better treatment planning and improved patient care.

**Assessment Category**	**Score**	**Assessment Category**	**Score**	**Assessment Category**	**Score**
Comprehension Score Rater 1	5	Gunning Fog Score	11.0	Words	126
Comprehension Score Rater 2	5	SMOG Index	8.6	Complex words	15
Reading Age (years)	16.5	Coleman Liau Index	14.1	Percentage of complex words	11.9
Flesch-Kincaid Reading Ease	55.6	Automated Readability Index	11.5	Average words per sentence	18.0
Flesch-Kincaid Grade Level	10.0	Sentences	7	Average syllables per word	1.57

Manuscript 172

Barsegian V, Möckel D, Buehler S, Müller SP, Kreissl MC, Ostheim P, et al. Lymphocyte Function at Baseline Could Be a New Predictor of Tumor Burden following Six Cycles of Radium-223 Therapy in Patients with Metastasized, Castration-Resistant Prostate Cancer. Cancers [Internet]. 2024 Feb 22;16(5):886. Available from: http://dx.doi.org/10.3390/cancers16050886

Abstract

Previous data indicate that one cycle of treatment with radium-223 (223Ra) did not significantly impair lymphocyte function in patients with metastasized, castration-resistant prostate cancer. The aim of the current study was to assess in 21 patients whether six cycles of this therapy had an effect on lymphocyte proliferation and interferon-γ and interleukin (IL)-10 ELISpot results. Lymphocyte proliferation after stimulation with microbial antigens and the production of interferon-γ continuously decreased after six cycles of radionuclide therapy, reaching statistical significance (p p r = -0.82). As determined by receiver operating characteristic (ROC) curve analysis, tetanus-specific IL-10 spots at baseline had the highest predictive value (p = 0.005) for tumor burden at month 6, with an area under the curve (AUC) of 0.90 (sensitivity 100%, specificity 78%). In conclusion, we observed an additive effect of treatment with 223Ra on immune function and found that IL-10 secretion at baseline predicted tumor burden at month 6 after treatment.

Keywords:

ELISpot, antimicrobial immune response, interferon-γ, interleukin-10, lymphocyte proliferation, prediction of tumor burden, radium-223 therapy

Original Lay Summary

Patients with metastasized, castration-resistant prostate cancer can be treated locally with radioactive radium-223, which usually comprises six cycles. We wanted to know whether this treatment affects immune function. We performed cell culture experiments with white blood cells from the patients and added components of microorganisms. We tested the ability of the cells to proliferate and produce two cytokines (interferon-gamma and interleukin-10) that are important for the balance of the immune system. Our data in 21 patients indicate that the immune cells show impairment in their defense against microorganisms after treatment. As determined by bone scintigraphy, a reduction in tumor burden was observed in 67% of patients. Interestingly, even before treatment, the number of cells producing interleukin-10, an anti-inflammatory cytokine, was an indicator of tumor burden at the end of treatment. Thus, a blood test can help assess whether the treatment is likely to be successful.

**Assessment Category**	**Score**	**Assessment Category**	**Score**	**Assessment Category**	**Score**
Comprehension Score Rater 1	4	Gunning Fog Score	15.5	Words	151
Comprehension Score Rater 2	4	SMOG Index	11.2	Complex words	30
Reading Age (years)	19.5	Coleman Liau Index	15.8	Percentage of complex words	19.87
Flesch-Kincaid Reading Ease	36.4	Automated Readability Index	13.3	Average words per sentence	18.88
Flesch-Kincaid Grade Level	12.9	Sentences	8	Average syllables per word	1.79

<u>Lay Summary (Simple Prompt)</u>

This study explored how a type of cancer treatment called radium-223 affects the immune system in men with advanced prostate cancer that no longer responds to hormone therapy. The researchers wanted to see if the activity of certain immune cells, called lymphocytes, could predict how much cancer would remain after six treatment cycles. They found that the ability of lymphocytes to respond to infections weakened during treatment. Interestingly, they discovered that measuring the immune response to a common vaccine (tetanus) before starting treatment could help predict the amount of cancer left after therapy. This finding is important because it suggests that simple immune tests done before treatment may help doctors estimate how well radium-223 therapy will work, potentially leading to more personalized cancer care.

**Assessment Category**	**Score**	**Assessment Category**	**Score**	**Assessment Category**	**Score**
Comprehension Score Rater 1	4	Gunning Fog Score	16.4	Words	126
Comprehension Score Rater 2	4	SMOG Index	11.6	Complex words	20
Reading Age (years)	20.5	Coleman Liau Index	16.2	Percentage of complex words	15.87
Flesch-Kincaid Reading Ease	35.6	Automated Readability Index	16.7	Average words per sentence	25.2
Flesch-Kincaid Grade Level	14.6	Sentences	5	Average syllables per word	1.72

<u>Lay Summary (Extended Prompt)</u>

In this study, we explored how a type of cancer treatment called radium-223 affects the immune system in patients with advanced prostate cancer that has spread and no longer responds to hormone therapy. We focused on white blood cells, known as lymphocytes, to see if their activity could predict the size of the tumor after six rounds of treatment. We found that the ability of these cells to grow and produce important immune signals decreased over time. Interestingly, the level of a specific immune marker, interleukin-10, measured before starting treatment, helped predict how much cancer would remain after six months. This finding is important because it may help doctors identify which patients will benefit most from radium-223 therapy, leading to more personalized and effective cancer care.

**Assessment Category**	**Score**	**Assessment Category**	**Score**	**Assessment Category**	**Score**
Comprehension Score Rater 1	4	Gunning Fog Score	15.3	Words	129
Comprehension Score Rater 2	5	SMOG Index	10.4	Complex words	16
Reading Age (years)	19.5	Coleman Liau Index	14.0	Percentage of complex words	12.4
Flesch-Kincaid Reading Ease	44.9	Automated Readability Index	15.3	Average words per sentence	25.8
Flesch-Kincaid Grade Level	13.4	Sentences	5	Average syllables per word	1.6

Manuscript 173

Jabbari P, Yazdanpanah O, Benjamin DJ, Rezazadeh Kalebasty A. Supplement Use and Increased Risks of Cancer: Unveiling the Other Side of the Coin. Cancers [Internet]. 2024 Feb 22;16(5):880. Available from: http://dx.doi.org/10.3390/cancers16050880

Abstract

There is a rising trend in the consumption of dietary supplements, especially among adults, with the purpose of improving health. While marketing campaigns tout the potential health benefits of using dietary supplements, it is critical to evaluate the potential harmful effects associated with these supplements as well. The majority of the scarce research on the potential harmful effects of vitamins focuses on the acute or chronic toxicities associated with the use of dietary supplements. Quality research is still required to further investigate the risks of long-term use of dietary supplements, especially the risk of developing cancers. The present review concentrates on studies that have investigated the association between the risk of developing cancers and associated mortality with the risk of dietary supplements. Such an association has been reported for several vitamins, minerals, and other dietary supplements. Even though several of these studies come with their own shortcomings and critics, they must draw attention to further investigate long-term adverse effects of dietary supplements and advise consumers and healthcare providers to ponder the extensive use of dietary supplements.

Keywords:

cancer risk, prostate cancer, supplement, vitamin

<u>Original Lay Summary</u>

This study investigates the overlooked harmful effects of dietary supplements, particularly their potential association with increased cancer risk. In response to a rising trend in supplement consumption, we address the “more is better” mentality prevalent in marketing campaigns by focusing on the long-term and regular use of these supplements. Our findings reveal that beyond known toxicities at high levels, dietary supplements may contribute to increased risk of cancer. We consolidate evidence from studies highlighting the correlation between substantial micronutrient intake, supplement use, and the potential implications for cancer development and mortality. Recognizing the link between supplement consumption and increased cancer risk is crucial for informing consumers and empowering healthcare professionals in how to guide patients more effectively.

**Assessment Category**	**Score**	**Assessment Category**	**Score**	**Assessment Category**	**Score**
Comprehension Score Rater 1	5	Gunning Fog Score	22.7	Words	118
Comprehension Score Rater 2	5	SMOG Index	16.1	Complex words	39
Reading Age (years)	24.0	Coleman Liau Index	20.9	Percentage of complex words	33.05
Flesch-Kincaid Reading Ease	14.4	Automated Readability Index	19.7	Average words per sentence	23.6
Flesch-Kincaid Grade Level	17.1	Sentences	5	Average syllables per word	1.99

Lay Summary (Simple Prompt)

Many people take dietary supplements, such as vitamins and minerals, believing they improve health. While these products are often marketed for their benefits, it is important to understand that they may also carry risks. This review explores studies that have looked at the possible link between supplement use and an increased risk of developing cancer. Some research suggests that taking certain supplements over a long period might raise the chances of cancer or even affect survival rates. Although these studies have limitations, they highlight the need for more research on the long-term safety of supplements. The findings aim to raise awareness among consumers and healthcare providers, encouraging them to carefully consider the risks before using or recommending dietary supplements regularly.

**Assessment Category**	**Score**	**Assessment Category**	**Score**	**Assessment Category**	**Score**
Comprehension Score Rater 1	5	Gunning Fog Score	17.3	Words	121
Comprehension Score Rater 2	5	SMOG Index	12.5	Complex words	28
Reading Age (years)	20.5	Coleman Liau Index	16.9	Percentage of complex words	23.14
Flesch-Kincaid Reading Ease	38.1	Automated Readability Index	14.9	Average words per sentence	20.17
Flesch-Kincaid Grade Level	12.9	Sentences	6	Average syllables per word	1.75

Lay Summary (Extended Prompt)

Many people take dietary supplements like vitamins and minerals to improve their health. While these supplements are often advertised as beneficial, it's important to understand that they might also have risks. This study reviewed research on how using dietary supplements could be linked to a higher chance of developing cancer. Some studies have found connections between certain supplements and an increased risk of cancer, but more research is needed to fully understand these risks. The goal of this study is to raise awareness about the possible long-term harmful effects of supplements and to encourage both consumers and healthcare providers to carefully consider the need for these products.

**Assessment Category**	**Score**	**Assessment Category**	**Score**	**Assessment Category**	**Score**
Comprehension Score Rater 1	5	Gunning Fog Score	16.8	Words	108
Comprehension Score Rater 2	5	SMOG Index	12.1	Complex words	22
Reading Age (years)	19.5	Coleman Liau Index	16.1	Percentage of complex words	20.37
Flesch-Kincaid Reading Ease	43.9	Automated Readability Index	14.9	Average words per sentence	21.6
Flesch-Kincaid Grade Level	12.5	Sentences	5	Average syllables per word	1.67

Manuscript 174

Titze U, Titze B, Hansen T, Barth PJ, Ali FA, Schneider F, et al. Ex Vivo Fluorescence Confocal Microscopy of MRI-Guided Targeted Prostate Biopsies for Rapid Detection of Clinically Significant Carcinomas—A Feasibility Study. Cancers [Internet]. 2024 Feb 22;16(5):873. Available from: http://dx.doi.org/10.3390/cancers16050873

Abstract

Background: MRI-guided prostate biopsies from visible tumor-specific lesions (TBx) can be used to diagnose clinically significant carcinomas (csPCa) requiring treatment more selectively than conventional systematic biopsies (SBx). Ex vivo fluorescence confocal microscopy (FCM) is a novel technique that can be used to examine TBx prior to conventional histologic workup.

Methods: TBx from 150 patients were examined with FCM on the day of collection. Preliminary findings were reported within 2 h of collection. The results were statistically compared with the final histology.

Results: 27/40 (68%) of the csPCa were already recognized in the intraday FCM in accordance with the results of conventional histology. Even non-significant carcinomas (cisPCa) of the intermediate and high-risk groups (serum prostate-specific antigen (PSA) > 10 or 20 ng/mL) according to conventional risk stratifications were reliably detectable. In contrast, small foci of cisPCa were often not detected or were difficult to distinguish from reactive changes.

Conclusion: The rapid reporting of preliminary FCM findings helps to reduce the psychological stress on patients, and can improve the clinical management of csPCa. Additional SBx can be avoided in individual cases, leading to lower rates of complications and scarring in the future surgical area. Additional staging examinations can be arranged without losing time. FCM represents a promising basis for future AI-based diagnostic algorithms.

Keywords:

confocal microscopy, prostate MRI, prostate cancer, targeted prostate biopsy

<u>Original Lay Summary</u>

Ex vivo fluorescence confocal microscopy is a novel microscopic technique that allows lossless analysis of unfixed tissue. By examining MRI-guided biopsies from suspicious lesions of the prostate, the majority of prostate carcinomas requiring treatment can be diagnosed. The recorded digital images are a promising basis for future AI-supported diagnostic algorithms. The rapid feedback within a few hours of the biopsy collection can reduce the stress burden on patients and contribute to better patient care, as the necessary steps for the therapies can be organized promptly. It is also a promising diagnostic approach for future local therapies in which effective substances can be applied specifically to tumor foci.

**Assessment Category**	**Score**	**Assessment Category**	**Score**	**Assessment Category**	**Score**
Comprehension Score Rater 1	5	Gunning Fog Score	19.4	Words	109
Comprehension Score Rater 2	5	SMOG Index	13.9	Complex words	29
Reading Age (years)	21.5	Coleman Liau Index	16.9	Percentage of complex words	26.61
Flesch-Kincaid Reading Ease	23.3	Automated Readability Index	15.7	Average words per sentence	21.8
Flesch-Kincaid Grade Level	15.4	Sentences	5	Average syllables per word	1.91

Lay Summary (Simple Prompt)

Prostate cancer is a common disease, and accurate diagnosis is crucial for effective treatment. This study explored a new method called fluorescence confocal microscopy (FCM) to quickly examine prostate tissue samples taken through MRI-guided targeted biopsies. Researchers analyzed samples from 150 patients and compared the fast, same-day FCM results with traditional laboratory tests. They found that FCM could reliably detect most significant prostate cancers within just two hours, reducing the need for additional biopsies and unnecessary procedures. This faster diagnosis helps lower patient stress, reduces potential complications, and allows doctors to plan treatments more efficiently. FCM also holds promise for future improvements using artificial intelligence to support cancer diagnosis.

**Assessment Category**	**Score**	**Assessment Category**	**Score**	**Assessment Category**	**Score**
Comprehension Score Rater 1	5	Gunning Fog Score	17.5	Words	111
Comprehension Score Rater 2	5	SMOG Index	12.5	Complex words	28
Reading Age (years)	21.5	Coleman Liau Index	20.0	Percentage of complex words	25.23
Flesch-Kincaid Reading Ease	21.1	Automated Readability Index	16.5	Average words per sentence	18.5
Flesch-Kincaid Grade Level	14.9	Sentences	6	Average syllables per word	1.97

Lay Summary (Extended Prompt)

This study looked at a new way to detect serious prostate cancers quickly after a biopsy. Usually, doctors take tissue samples from the prostate using MRI to find suspicious areas, but it can take days to get the final results. In this study, researchers tested a new imaging technique called fluorescence confocal microscopy (FCM) to examine biopsy samples right after collection. They tested this method on 150 patients and found that FCM could identify most significant prostate cancers within just two hours. This faster diagnosis can help reduce stress for patients, allow doctors to make quicker treatment decisions, and even avoid unnecessary additional biopsies, which can cause complications. In the future, this method may also help improve computer-based diagnostic tools, making cancer detection even more accurate.

**Assessment Category**	**Score**	**Assessment Category**	**Score**	**Assessment Category**	**Score**
Comprehension Score Rater 1	5	Gunning Fog Score	16.3	Words	127
Comprehension Score Rater 2	5	SMOG Index	11.8	Complex words	25
Reading Age (years)	19.5	Coleman Liau Index	15.8	Percentage of complex words	19.69
Flesch-Kincaid Reading Ease	34.8	Automated Readability Index	14.4	Average words per sentence	21.17
Flesch-Kincaid Grade Level	13.7	Sentences	6	Average syllables per word	1.78

Manuscript 175

Feretzakis G, Juliebø-Jones P, Tsaturyan A, Sener TE, Verykios VS, Karapiperis D, et al. Emerging Trends in AI and Radiomics for Bladder, Kidney, and Prostate Cancer: A Critical Review. Cancers [Internet]. 2024 Feb 16;16(4):810. Available from: http://dx.doi.org/10.3390/cancers16040810

Abstract

This comprehensive review critically examines the transformative impact of artificial intelligence (AI) and radiomics in the diagnosis, prognosis, and management of bladder, kidney, and prostate cancers. These cutting-edge technologies are revolutionizing the landscape of cancer care, enhancing both precision and personalization in medical treatments. Our review provides an in-depth analysis of the latest advancements in AI and radiomics, with a specific focus on their roles in urological oncology. We discuss how AI and radiomics have notably improved the accuracy of diagnosis and staging in bladder cancer, especially through advanced imaging techniques like multiparametric MRI (mpMRI) and CT scans. These tools are pivotal in assessing muscle invasiveness and pathological grades, critical elements in formulating treatment plans. In the realm of kidney cancer, AI and radiomics aid in distinguishing between renal cell carcinoma (RCC) subtypes and grades. The integration of radiogenomics offers a comprehensive view of disease biology, leading to tailored therapeutic approaches. Prostate cancer diagnosis and management have also seen substantial benefits from these technologies. AI-enhanced MRI has significantly improved tumor detection and localization, thereby aiding in more effective treatment planning. The review also addresses the challenges in integrating AI and radiomics into clinical practice, such as the need for standardization, ensuring data quality, and overcoming the "black box" nature of AI. We emphasize the importance of multicentric collaborations and extensive studies to enhance the applicability and generalizability of these technologies in diverse clinical settings. In conclusion, AI and radiomics represent a major paradigm shift in oncology, offering more precise, personalized, and patient-centric approaches to cancer care. While

their potential to improve diagnostic accuracy, patient outcomes, and our understanding of cancer biology is profound, challenges in clinical integration and application persist. We advocate for continued research and development in AI and radiomics, underscoring the need to address existing limitations to fully leverage their capabilities in the field of oncology.

Keywords:

artificial intelligence, bladder cancer, diagnostic imaging, kidney cancer, oncology, personalized medicine, prostate cancer, radiomics, urological cancers

<u>Original Lay Summary</u>

In an age where technology is deeply intertwined with healthcare, this review focuses on the synergistic role of artificial intelligence (AI) and radiomics in the management of urological cancers, particularly bladder, kidney, and prostate cancers. Our comprehensive review explores how AI's rapid data-processing capabilities, combined with the intricate image analysis offered by radiomics, are reshaping cancer diagnosis and treatment. We delve into current research findings to illustrate how these innovative technologies are steering oncology toward more accurate, personalized care. This summary is crafted to be accessible, avoiding complex medical jargon and extensive academic references, aiming to highlight the essence and potential impact of these advancements. Our objective is to showcase how AI and radiomics are instrumental in early cancer detection, informed therapeutic decisions, and potentially improved patient outcomes. The research compiled in this paper not only charts a course for the future integration of these technologies in cancer care but also underscores the emerging trend towards patient-centric strategies in the medical community, offering renewed hope and direction in the fight against these cancers.

**Assessment Category**	**Score**	**Assessment Category**	**Score**	**Assessment Category**	**Score**
Comprehension Score Rater 1	5	Gunning Fog Score	22.6	Words	175
Comprehension Score Rater 2	5	SMOG Index	16.3	Complex words	48
Reading Age (years)	24.0	Coleman Liau Index	19.4	Percentage of complex words	27.43
Flesch-Kincaid Reading Ease	7.5	Automated Readability Index	21.3	Average words per sentence	29.17

Flesch-Kincaid Grade Level	19.5	Sentences	6	Average syllables per word	2.01

Lay Summary (Simple Prompt)

Artificial intelligence (AI) and radiomics are transforming how we diagnose and treat bladder, kidney, and prostate cancers. This review explores how these advanced technologies improve the accuracy of cancer detection and help doctors plan more personalized treatments. AI can analyze complex medical images, like MRI and CT scans, to identify tumors more precisely, predict how aggressive the cancer might be, and suggest the best treatment options. In kidney cancer, AI helps distinguish between different types of tumors, while in prostate cancer, it improves the detection and targeting of cancer cells. Despite their promising potential, challenges remain, such as ensuring the quality of data, standardizing methods, and understanding how AI makes its decisions. This review highlights the importance of ongoing research and collaboration to overcome these hurdles, with the goal of making cancer care more precise, effective, and tailored to each patient's needs.

**Assessment Category**	**Score**	**Assessment Category**	**Score**	**Assessment Category**	**Score**
Comprehension Score Rater 1	5	Gunning Fog Score	17.6	Words	142
Comprehension Score Rater 2	5	SMOG Index	12.9	Complex words	30
Reading Age (years)	21.5	Coleman Liau Index	17.6	Percentage of complex words	21.13
Flesch-Kincaid Reading Ease	32.7	Automated Readability Index	17.1	Average words per sentence	23.67

Flesch-Kincaid Grade Level	14.6	Sentences	6	Average syllables per word	1.77

Lay Summary (Extended Prompt)

This review explores how new technologies like artificial intelligence (AI) and radiomics are changing the way doctors detect and treat bladder, kidney, and prostate cancers. AI helps computers analyze medical images, making it easier to find tumors and understand how aggressive they are. Radiomics looks at details in these images that the human eye might miss, providing important clues about the cancer. Together, these tools help doctors make more accurate diagnoses and create personalized treatment plans tailored to each patient's needs. In bladder cancer, they improve the ability to see if cancer has spread. For kidney cancer, they help identify different cancer types. In prostate cancer, they make it easier to spot tumors early. While these technologies offer exciting benefits, there are still challenges in using them widely in hospitals. Continued research is needed to make sure they work well for all patients, leading to better care and outcomes in the future.

**Assessment Category**	**Score**	**Assessment Category**	**Score**	**Assessment Category**	**Score**
Comprehension Score Rater 1	5	Gunning Fog Score	13.6	Words	152
Comprehension Score Rater 2	5	SMOG Index	9.9	Complex words	26
Reading Age (years)	17.5	Coleman Liau Index	15.5	Percentage of complex words	17.11
Flesch-Kincaid Reading Ease	48.9	Automated Readability Index	12.0	Average words per sentence	16.89
Flesch-Kincaid Grade Level	10.6	Sentences	9	Average syllables per word	1.66

Manuscript 176

Kouroukli O, Bravou V, Giannitsas K, Tzelepi V. Tissue-Based Diagnostic Biomarkers of Aggressive Variant Prostate Cancer: A Narrative Review. Cancers [Internet]. 2024 Feb 16;16(4):805. Available from: http://dx.doi.org/10.3390/cancers16040805

Abstract

Prostate cancer (PC) is a common malignancy among elderly men, characterized by great heterogeneity in its clinical course, ranging from an indolent to a highly aggressive disease. The aggressive variant of prostate cancer (AVPC) clinically shows an atypical pattern of disease progression, similar to that of small cell PC (SCPC), and also shares the chemo-responsiveness of SCPC. The term AVPC does not describe a specific histologic subtype of PC but rather the group of tumors that, irrespective of morphology, show an aggressive clinical course, dictated by androgen receptor (AR) indifference. AR indifference represents an adaptive response to androgen deprivation therapy (ADT), driven by epithelial plasticity, an inherent ability of tumor cells to adapt to their environment by changing their phenotypic characteristics in a bi-directional way. The molecular profile of AVPC entails combined alterations in the tumor suppressor genes retinoblastoma protein 1 (RB1), tumor protein 53 (TP53), and phosphatase and tensin homolog (PTEN). The understanding of the biologic heterogeneity of castration-resistant PC (CRPC) and the need to identify the subset of patients that would potentially benefit from specific therapies necessitate the development of prognostic and predictive biomarkers. This review aims to discuss the possible pathophysiologic mechanisms of AVPC development and the potential use of emerging tissue-based biomarkers in clinical practice.

Keywords:

ADT, AVPC, DDR, NEPC, biomarkers, epigenetic regulation, transdifferentiation, tumor suppressors

<u>Original Lay Summary</u>

Metastatic prostate cancer is traditionally treated with androgen deprivation therapy. The introduction of second-generation antiandrogens into clinical practice elicits prolonged responses but also gives rise to mechanisms of resistance that do not rely on androgens. The emergent phenotype of androgen-indifferent prostate cancer is associated with an aggressive, atypical clinical course. The term “aggressive variant prostate cancer” (AVPC) has been coined in order to separate this phenotype from hormone-responsive tumors. Unfortunately, morphology alone cannot reliably predict virulent behavior. The development of prognostic and predictive biomarkers is therefore crucial. In line with this, research has been focusing on unraveling the biological identity of AVPC. Drawing from the current knowledge about AVPC molecular pathogenesis and evolution, we attempt to identify candidate tissue-based AVPC biomarkers.

**Assessment Category**	**Score**	**Assessment Category**	**Score**	**Assessment Category**	**Score**
Comprehension Score Rater 1	5	Gunning Fog Score	20.7	Words	125
Comprehension Score Rater 2	5	SMOG Index	14.0	Complex words	47
Reading Age (years)	23.5	Coleman Liau Index	21.0	Percentage of complex words	37.6
Flesch-Kincaid Reading Ease	7.6	Automated Readability Index	15.8	Average words per sentence	15.63
Flesch-Kincaid Grade Level	16.1	Sentences	8	Average syllables per word	2.17

Lay Summary (Simple Prompt)

Prostate cancer is a common disease in older men and can behave very differently from one person to another—some cases grow slowly, while others are much more aggressive. This review focuses on a particularly aggressive type of prostate cancer that grows quickly and does not respond well to standard hormone treatments. Unlike typical forms, this aggressive variant does not look the same under the microscope but behaves similarly because it has adapted to resist therapy. Scientists believe this behavior is linked to specific changes in certain genes that usually help control cell growth. Understanding these changes can help doctors predict how the cancer will behave and choose the best treatment. This review explores new tissue-based tests, called biomarkers, that might help identify aggressive prostate cancers earlier and guide more effective treatment strategies in the future.

**Assessment Category**	**Score**	**Assessment Category**	**Score**	**Assessment Category**	**Score**
Comprehension Score Rater 1	4	Gunning Fog Score	15.5	Words	136
Comprehension Score Rater 2	4	SMOG Index	11.1	Complex words	22
Reading Age (years)	19.5	Coleman Liau Index	16.4	Percentage of complex words	16.18
Flesch-Kincaid Reading Ease	40.8	Automated Readability Index	15.7	Average words per sentence	22.67
Flesch-Kincaid Grade Level	13.2	Sentences	6	Average syllables per word	1.69

<u>Lay Summary (Extended Prompt)</u>

Prostate cancer is a common disease in older men, and while some cases grow slowly, others are very aggressive and hard to treat. This review focuses on aggressive prostate cancer, which grows and spreads quickly, often not responding to standard hormone therapy. These aggressive cancers change how they behave to survive, making treatment more difficult. Scientists are studying specific changes in the cancer's genes to understand why this happens. By identifying new tissue-based markers—special signs in the cancer cells—doctors may be able to detect aggressive cancer earlier and choose better treatments for each patient. This research aims to help improve the diagnosis and care of men with aggressive prostate cancer, leading to more personalized and effective treatments.

**Assessment Category**	**Score**	**Assessment Category**	**Score**	**Assessment Category**	**Score**
Comprehension Score Rater 1	4	Gunning Fog Score	14.0	Words	118
Comprehension Score Rater 2	4	SMOG Index	10.1	Complex words	18
Reading Age (years)	18.5	Coleman Liau Index	16.6	Percentage of complex words	15.25
Flesch-Kincaid Reading Ease	45.6	Automated Readability Index	14.3	Average words per sentence	19.67
Flesch-Kincaid Grade Level	11.8	Sentences	6	Average syllables per word	1.67

Manuscript 177

Kaulfers T, Lattery G, Cheng C, Zhao X, Selvaraj B, Wu H, et al. Pencil Beam Scanning Proton Bragg Peak Conformal FLASH in Prostate Cancer Stereotactic Body Radiotherapy. Cancers [Internet]. 2024 Feb 15;16(4):798. Available from: http://dx.doi.org/10.3390/cancers16040798

Abstract

Bragg peak FLASH radiotherapy (RT) uses a distal tracking method to eliminate exit doses and can achieve superior OAR sparing. This study explores the application of this novel method in stereotactic body radiotherapy prostate FLASH-RT. An in-house platform was developed to enable intensity-modulated proton therapy (IMPT) planning using a single-energy Bragg peak distal tracking method. The patients involved in the study were previously treated with proton stereotactic body radiotherapy (SBRT) using the pencil beam scanning (PBS) technique to 40 Gy in five fractions. FLASH plans were optimized using a four-beam arrangement to generate a dose distribution similar to the conventional opposing beams. All of the beams had a small angle of two degrees from the lateral direction to increase the dosimetry quality. Dose metrics were compared between the conventional PBS and the Bragg peak FLASH plans. The dose rate histogram (DRVH) and FLASH metrics of 40 Gy/s coverage (V40Gy/s) were investigated for the Bragg peak plans. There was no significant difference between the clinical and Bragg peak plans in rectum, bladder, femur heads, large bowel, and penile bulb dose metrics, except for Dmax. For the CTV, the FLASH plans resulted in a higher Dmax than the clinical plans (116.9% vs. 103.3%). For the rectum, the V40Gy/s reached 94% and 93% for 1 Gy dose thresholds in composite and single-field evaluations, respectively. Additionally, the FLASH ratio reached close to 100% after the application of the 5 Gy threshold in composite dose rate assessment. In conclusion, the Bragg peak distal tracking method can yield comparable plan quality in most OARs while preserving sufficient FLASH dose rate coverage, demonstrating that the ultra-high dose technique can be applied in prostate FLASH SBRT.

Keywords:

FLASH radiotherapy, intensity-modulated proton therapy, prostate cancer, proton pencil beam scanning, single-energy Bragg peak, stereotactic body radiotherapy, ultra-high dose rate

<u>Original Lay Summary</u>

Prostate cancer is one of the most diagnosed cancers in men. While it can be successfully treated with radiotherapy (RT), patients often leave treatment with previously healthy organs affected by radiation. This work assessed a novel ultra-high dose rate FLASH proton therapy technique for the treatment of prostate cancers. We were able to generate clinically viable Bragg peak FLASH treatment plans for prostate cancer patients previously treated with conventional proton RT. Feasible prostate treatment quality was observed with all constraints for organs at risk (OARs) being met while maintaining an ultra-high dose rate.

**Assessment Category**	**Score**	**Assessment Category**	**Score**	**Assessment Category**	**Score**
Comprehension Score Rater 1	5	Gunning Fog Score	14.3	Words	95
Comprehension Score Rater 2	5	SMOG Index	10.4	Complex words	16
Reading Age (years)	19.5	Coleman Liau Index	16.5	Percentage of complex words	16.84
Flesch-Kincaid Reading Ease	40.6	Automated Readability Index	14.0	Average words per sentence	19.0
Flesch-Kincaid Grade Level	12.3	Sentences	5	Average syllables per word	1.74

Lay Summary (Simple Prompt)

This study investigates a new way to deliver radiation therapy to patients with prostate cancer using a technique called Bragg peak FLASH radiotherapy. This method aims to reduce damage to healthy tissues while still effectively treating the cancer. Researchers developed a special planning system to compare this new method with the conventional radiation approach. They tested it on patients who had already received prostate cancer treatment, focusing on how well the radiation targeted the tumor while sparing nearby organs like the rectum and bladder. The results showed that the new method provides similar protection to healthy organs as the traditional approach, with some differences in the maximum dose delivered to certain areas. Importantly, this technique allows for ultra-high radiation doses to be delivered very quickly, which could lead to fewer side effects and shorter treatment times. This promising approach may improve future prostate cancer treatments.

**Assessment Category**	**Score**	**Assessment Category**	**Score**	**Assessment Category**	**Score**
Comprehension Score Rater 1	5	Gunning Fog Score	15.5	Words	146
Comprehension Score Rater 2	5	SMOG Index	11.2	Complex words	26
Reading Age (years)	20.5	Coleman Liau Index	17.2	Percentage of complex words	17.81
Flesch-Kincaid Reading Ease	35.6	Automated Readability Index	15.4	Average words per sentence	20.86
Flesch-Kincaid Grade Level	13.5	Sentences	7	Average syllables per word	1.77

Lay Summary (Extended Prompt)

This study looks at a new way to treat prostate cancer using a special type of radiation therapy called FLASH radiotherapy. Unlike traditional methods, FLASH delivers radiation very quickly, which may help protect healthy organs while still effectively treating cancer. The researchers tested a new planning method that focuses the radiation more precisely using a technique called the Bragg peak. They compared this new method with the standard approach to see how well it worked and if it was safe. The results showed that the new method provided similar protection for healthy tissues and delivered the radiation at ultra-high speeds, which might reduce side effects. This means that prostate cancer patients could receive faster, more targeted treatments in the future with fewer risks to surrounding organs.

**Assessment Category**	**Score**	**Assessment Category**	**Score**	**Assessment Category**	**Score**
Comprehension Score Rater 1	5	Gunning Fog Score	14.1	Words	127
Comprehension Score Rater 2	5	SMOG Index	10.1	Complex words	18
Reading Age (years)	18.5	Coleman Liau Index	15.5	Percentage of complex words	14.17
Flesch-Kincaid Reading Ease	46.1	Automated Readability Index	14.2	Average words per sentence	21.17
Flesch-Kincaid Grade Level	12.1	Sentences	6	Average syllables per word	1.65

Manuscript 178

Vieceli M, Park J, Hsi WC, Saki M, Mendenhall NP, Johnson P, et al. Potential Therapeutic Improvements in Prostate Cancer Treatment Using Pencil Beam Scanning Proton Therapy with LETd Optimization and Disease-Specific RBE Models. Cancers [Internet]. 2024 Feb 14;16(4):780. Available from: http://dx.doi.org/10.3390/cancers16040780

Abstract

Purpose: To demonstrate the feasibility of improving prostate cancer patient outcomes with PBS proton LETd optimization.

Methods: SFO, IPT-SIB, and LET-optimized plans were created for 12 patients, and generalized-tissue and disease-specific LET-dependent RBE models were applied. The mean LETd in several structures was determined and used to calculate mean RBEs. LETd- and dose-volume histograms (LVHs/DVHs) are shown. TODRs were defined based on clinical dose goals and compared between plans. The impact of robust perturbations on LETd, TODRs, and DVH spread was evaluated.

Results: LETd optimization achieved statistically significant increased target volume LETd of ~4 keV/μm compared to SFO and IPT-SIB LETd of ~2 keV/μm while mitigating OAR LETd increases. A disease-specific RBE model predicted target volume RBEs > 1.5 for LET-optimized plans, up to 18% higher than for SFO plans. LET-optimized target LVHs/DVHs showed a large increase not present in OARs. All RBE models showed a statistically significant increase in TODRs from SFO to IPT-SIB to LET-optimized plans. RBE = 1.1 does not accurately represent TODRs when using LETd optimization. Robust evaluations demonstrated a trade-off between increased mean target LETd and decreased DVH spread.

Conclusion: The demonstration of improved TODRs provided via LETd optimization shows potential for improved patient outcomes.

Keywords:

LET-optimized pencil beam scanning proton therapy, disease-specific/prostate-cancer-specific LET-dependent RBE modeling, prostate cancer treatment

Original Lay Summary

LET-optimized pencil beam scanning (PBS) proton treatment plans have the potential to improve patient outcomes. Dose-escalated x-ray conformal therapy has improved the prostate cancer 5-year freedom from biochemical progression (FFBP) but has also increased gastrointestinal and genitourinary toxicities; toxicities can be reduced by escalating dose via a simultaneously-integrated boost (SIB) to the intraprostatic tumor (IPT). These techniques can be applied with greater precision using protons, which have shown high overall 5-year FFBP and low toxicities, although improvement in FFBP is needed for high-risk patients. Relative biological effectiveness (RBE) of 1.1 is uniformly assumed for proton treatment planning, although an increasing number of studies show that RBE increases with linear energy transfer (LETd). We show improvement with PBS LET-optimized plans over single-field optimized (SFO), or IPT-SIB plans through increased target volume LETd, RBE, and target-to-organs-at-risk (OAR) dose ratios (TODRs). This study also highlights the necessity of developing disease-specific LET-dependent RBE models.

**Assessment Category**	**Score**	**Assessment Category**	**Score**	**Assessment Category**	**Score**
Comprehension Score Rater 1	5	Gunning Fog Score	17.7	Words	167
Comprehension Score Rater 2	5	SMOG Index	12.7	Complex words	34
Reading Age (years)	21.5	Coleman Liau Index	16.6	Percentage of complex words	20.36
Flesch-Kincaid Reading Ease	27.6	Automated Readability Index	16.4	Average words per sentence	23.86
Flesch-Kincaid Grade Level	15.3	Sentences	7	Average syllables per word	1.83

Lay Summary (Simple Prompt)

Prostate cancer treatment can be improved using a special type of radiation therapy called pencil beam scanning proton therapy. This study explored how adjusting a specific factor in the therapy, known as LETd, could make the treatment more effective while reducing harm to healthy tissues. Researchers tested different treatment plans on 12 patients, comparing traditional methods with a new LETd-optimized approach. They found that the new method significantly increased the effectiveness of radiation in targeting cancer cells without causing additional damage to surrounding healthy organs. The results suggest that this optimized therapy could lead to better outcomes for prostate cancer patients, offering more precise treatment with potentially fewer side effects. This research highlights a promising step forward in making prostate cancer therapy more effective and safer for patients.

**Assessment Category**	**Score**	**Assessment Category**	**Score**	**Assessment Category**	**Score**
Comprehension Score Rater 1	5	Gunning Fog Score	16.7	Words	129
Comprehension Score Rater 2	5	SMOG Index	12.0	Complex words	26
Reading Age (years)	21.5	Coleman Liau Index	18.6	Percentage of complex words	20.16
Flesch-Kincaid Reading Ease	30.2	Automated Readability Index	16.8	Average words per sentence	21.5
Flesch-Kincaid Grade Level	14.4	Sentences	6	Average syllables per word	1.83

<u>Lay Summary (Extended Prompt)</u>

This study explored a new way to improve prostate cancer treatment using a special type of proton therapy called pencil beam scanning. The researchers focused on a method known as LETd optimization, which helps direct radiation more effectively to cancer cells while reducing harm to healthy tissue. They tested this approach on treatment plans for 12 patients and found that it increased the strength of radiation in the tumor, making the treatment more powerful without increasing risks to nearby organs. The new method showed better results compared to standard techniques, suggesting it could help improve patient outcomes. This means prostate cancer treatments could become more effective and safer in the future, offering hope for better recovery and quality of life for patients.

**Assessment Category**	**Score**	**Assessment Category**	**Score**	**Assessment Category**	**Score**
Comprehension Score Rater 1	5	Gunning Fog Score	14.7	Words	122
Comprehension Score Rater 2	5	SMOG Index	10.1	Complex words	15
Reading Age (years)	19.5	Coleman Liau Index	15.8	Percentage of complex words	12.3
Flesch-Kincaid Reading Ease	42.0	Automated Readability Index	16.0	Average words per sentence	24.4
Flesch-Kincaid Grade Level	13.5	Sentences	5	Average syllables per word	1.66

Manuscript 179

Nakamura S, Nagata M, Nagaya N, Ashizawa T, Hirano H, Lu Y, et al. The Detection and Negative Reversion of Circulating Tumor Cells as Prognostic Biomarkers for Metastatic Castration-Resistant Prostate Cancer with Bone Metastases Treated by Enzalutamide. Cancers [Internet]. 2024 Feb 13;16(4):772. Available from: http://dx.doi.org/10.3390/cancers16040772

Abstract

Enzalutamide is a second-generation androgen receptor inhibitor that increases overall survival (OS) rates in patients with metastatic castration-resistant prostate cancer (mCRPC). This study evaluates the efficacy of circulating tumor cell (CTC) status as a prognostic biomarker following enzalutamide administration. A retrospective subgroup analysis and prognostic survey were conducted on 43 patients with mCRPC and bone metastases treated in Juntendo University-affiliated hospitals from 2015 to 2022. Patients were treated with 160 mg enzalutamide daily. CTC analyses on blood samples were performed regularly before and every three months after treatment. The relationship between the patients' clinical factors and the OS rate was analyzed using the log-rank test; the median OS was 37 months. Patients with no detected CTCs at baseline showed significantly longer OS than those with detectable CTCs at baseline. Furthermore, patients demonstrating negative reversion of CTCs during enzalutamide treatment had significantly longer OS than patients with CTC-positivity. Two biomarkers-higher hemoglobin at baseline and achieving negative reversion of CTCs-were significantly associated with prolonged OS. This study suggests that patients achieving CTC-negative reversion during treatment for mCRPC with bone metastases exhibit improved long-term OS. Chronological measurement of CTC status might be clinically useful in the treatment of mCRPC.

Keywords:

androgen receptor signaling inhibitor, bone metastases, castration-resistant prostate cancer, circulating tumor cells (CTCs), enzalutamide

<u>Original Lay Summary</u>

The development of biomarkers that can predict the effectiveness of treatments for metastatic castration-resistant prostate cancer (mCRPC) has been a recent challenge. We focused on the efficacy of circulating tumor cell (CTC) status as a prognostic biomarker after enzalutamide administration. A retrospective subgroup analysis and prognostic survey were conducted on 43 patients with mCRPC and bone metastases. The results showed that patients with no detected CTCs at baseline showed significantly longer overall survival (OS) than those with CTCs at baseline. Furthermore, patients who exhibited a negative reversion of CTC status during enzalutamide treatment had significantly longer OS compared to patients with persistent CTC positivity. Achieving CTC-negative reversion during treatment for mCRPC with bone metastases was associated with improved long-term OS. Chronological measurement of CTC status might be clinically useful in the treatment of mCRPC.

**Assessment Category**	**Score**	**Assessment Category**	**Score**	**Assessment Category**	**Score**
Comprehension Score Rater 1	5	Gunning Fog Score	19.2	Words	137
Comprehension Score Rater 2	5	SMOG Index	13.6	Complex words	39
Reading Age (years)	22.5	Coleman Liau Index	19.1	Percentage of complex words	28.47
Flesch-Kincaid Reading Ease	18.4	Automated Readability Index	16.2	Average words per sentence	19.57
Flesch-Kincaid Grade Level	15.6	Sentences	7	Average syllables per word	1.99

<u>Lay Summary (Simple Prompt)</u>

This study explored how specific cancer cells found in the blood, called circulating tumor cells (CTCs), can help predict the survival of patients with advanced prostate cancer that has spread to the bones. The patients were treated with enzalutamide, a medication that blocks cancer growth signals. Researchers regularly tested blood samples from 43 patients to see if CTCs were present before and during treatment. They found that patients who either had no CTCs at the start or whose CTCs disappeared during treatment lived significantly longer than those with persistent CTCs. Additionally, having higher hemoglobin levels at the beginning of treatment was linked to better outcomes. These results suggest that monitoring CTCs over time can provide valuable information about how well a patient is responding to treatment, helping doctors make more informed decisions to improve patient care.

**Assessment Category**	**Score**	**Assessment Category**	**Score**	**Assessment Category**	**Score**
Comprehension Score Rater 1	5	Gunning Fog Score	14.4	Words	136
Comprehension Score Rater 2	5	SMOG Index	10.1	Complex words	18
Reading Age (years)	19.5	Coleman Liau Index	16.5	Percentage of complex words	13.24
Flesch-Kincaid Reading Ease	42.6	Automated Readability Index	15.7	Average words per sentence	22.67
Flesch-Kincaid Grade Level	12.9	Sentences	6	Average syllables per word	1.67

<u>Lay Summary (Extended Prompt)</u>

This study looked at men with advanced prostate cancer that had spread to their bones and was no longer responding to hormone therapy. The researchers wanted to see if tiny cancer cells found in the blood, called circulating tumor cells (CTCs), could help predict how well patients would respond to a drug called enzalutamide. They followed 43 patients, checking their blood before and during treatment. They found that patients who had no CTCs in their blood at the start of treatment lived longer than those who did. Even more importantly, patients whose CTCs disappeared during treatment also lived longer. This suggests that regularly checking for CTCs in the blood could help doctors understand how well the treatment is working and predict patient outcomes. This simple blood test might help improve care for men with advanced prostate cancer in the future.

**Assessment Category**	**Score**	**Assessment Category**	**Score**	**Assessment Category**	**Score**
Comprehension Score Rater 1	5	Gunning Fog Score	10.6	Words	140
Comprehension Score Rater 2	5	SMOG Index	6.7	Complex words	9
Reading Age (years)	16.5	Coleman Liau Index	14.0	Percentage of complex words	6.43
Flesch-Kincaid Reading Ease	64.5	Automated Readability Index	12.4	Average words per sentence	20.0
Flesch-Kincaid Grade Level	9.2	Sentences	7	Average syllables per word	1.44

Manuscript 180

Faiella E, Santucci D, D'Amone G, Cirimele V, Vertulli D, Bruno A, et al. Focal Minimally Invasive Treatment in Localized Prostate Cancer: Comprehensive Review of Different Possible Strategies. Cancers [Internet]. 2024 Feb 13;16(4):765. Available from: http://dx.doi.org/10.3390/cancers16040765

Abstract

Background: Focal therapy is a promising, minimally invasive method for the treatment of patients with localized prostate cancer. According to the existing literature, there is growing evidence for positive functional outcomes and oncological effectiveness. The aim of this review is to evaluate the technical efficacy of three minimally invasive techniques (cryoablation, electroporation, and microwave ablation) and their impact on quality of life in patients with prostate cancer.

Methods: Studies between January 2020 and July 2023 were selected using PubMed, Embase, and The Cochrane Library and analyzed following PRISMA guidelines; they have not been registered.

Results: Twenty-three studies investigating three different sources of energy to deliver focal therapy were found. Thirteen studies evaluated the performance of the cryoablation therapy, seven studies of the irreversible electroporation, and three studies of microwave ablation option. The majority of studies were retrospective cohort studies. Cryoablation showed excellent oncological outcomes for low-grade prostate cancer, whether performed on the lesion, on the hemigland, or on the entire gland, with the best results obtained for patients with intermediate risk. Irreversible electroporation showed promising oncological outcomes with no significant changes in functional outcomes. Microwave ablation showed great early functional outcomes.

Conclusions: The oncological effectiveness of minimally invasive treatment in comparison to standard of care is still under investigation, despite encouraging results in terms of functional outcomes improvement and adverse events reduction. More comprehensive research is needed to fully understand the function of minimally invasive treatment in patients with localized PCa.

Keywords:

ablation techniques, cryotherapy, focal therapy, irreversible electroporation, microwave ablation, prostate cancer

<u>Original Lay Summary</u>

This systematic review includes 23 studies about minimally invasive methods for selective treatment of localized prostate cancer. At present, standard treatment options for localized prostate cancer are active surveillance and whole-gland treatment (radical prostatectomy and radiotherapy). Regrettably, morbidities are represented by urinary incontinence, erectile dysfunction, and bowel dysfunction, which can worsen the quality of life without necessarily improving the patient's oncological outcome. Minimally invasive methods have demonstrated encouraging results in terms of functional outcomes and low adverse events. The focus of this review is to update evidence about the oncological effectiveness of three minimally invasive techniques (cryoablation, irreversible electroporation, and microwave ablation).

**Assessment Category**	**Score**	**Assessment Category**	**Score**	**Assessment Category**	**Score**
Comprehension Score Rater 1	5	Gunning Fog Score	23.0	Words	103
Comprehension Score Rater 2	5	SMOG Index	15.9	Complex words	38
Reading Age (years)	24.0	Coleman Liau Index	23.9	Percentage of complex words	36.89
Flesch-Kincaid Reading Ease	-8.7	Automated Readability Index	20.7	Average words per sentence	20.6
Flesch-Kincaid Grade Level	19.6	Sentences	5	Average syllables per word	2.3

Lay Summary (Simple Prompt)

Prostate cancer is one of the most common cancers in men, and new ways to treat it with less harm to healthy tissue are being explored. This study reviews three promising, minimally invasive treatments—cryoablation, irreversible electroporation, and microwave ablation—that target only the cancer while leaving the rest of the prostate intact. Researchers analyzed 23 recent studies to understand how effective these treatments are and how they affect patients' quality of life. The results showed that cryoablation works well for patients with low- to intermediate-risk prostate cancer, irreversible electroporation has encouraging cancer control with minimal impact on normal functions, and microwave ablation offers good early results. While these methods show potential to improve outcomes and reduce side effects compared to traditional treatments, more research is needed to confirm their long-term benefits. This review helps doctors and patients better understand new options for treating localized prostate cancer.

**Assessment Category**	**Score**	**Assessment Category**	**Score**	**Assessment Category**	**Score**
Comprehension Score Rater 1	5	Gunning Fog Score	16.9	Words	147
Comprehension Score Rater 2	5	SMOG Index	12.0	Complex words	26
Reading Age (years)	21.5	Coleman Liau Index	18.6	Percentage of complex words	17.69
Flesch-Kincaid Reading Ease	28.9	Automated Readability Index	18.3	Average words per sentence	24.5
Flesch-Kincaid Grade Level	15.3	Sentences	6	Average syllables per word	1.81

<u>Lay Summary (Extended Prompt)</u>

Prostate cancer is a common disease, and finding ways to treat it effectively with fewer side effects is important. This study looks at three minimally invasive treatments—cryoablation (freezing cancer cells), irreversible electroporation (using electrical pulses), and microwave ablation (using heat)—to see how well they work for men with localized prostate cancer. Researchers reviewed 23 studies published between 2020 and 2023 to compare these methods. Cryoablation showed the best cancer control, especially for men with intermediate-risk cancer. Irreversible electroporation had promising results without major changes in quality of life, and microwave ablation showed good early outcomes. While these treatments look promising, more research is needed to understand how they compare to standard treatments. The goal is to improve cancer outcomes while reducing side effects, helping men live better, healthier lives after treatment.

**Assessment Category**	**Score**	**Assessment Category**	**Score**	**Assessment Category**	**Score**
Comprehension Score Rater 1	5	Gunning Fog Score	15.1	Words	33
Comprehension Score Rater 2	5	SMOG Index	11.0	Complex words	25
Reading Age (years)	20.5	Coleman Liau Index	18.5	Percentage of complex words	18.8
Flesch-Kincaid Reading Ease	33.6	Automated Readability Index	15.6	Average words per sentence	19.0
Flesch-Kincaid Grade Level	13.3	Sentences	7	Average syllables per word	1.82

Manuscript 181

Sciarra A, Santarelli V, Salciccia S, Moriconi M, Basile G, Santodirocco L, et al. How the Management of Biochemical Recurrence in Prostate Cancer Will Be Modified by the Concept of Anticipation and Incrementation of Therapy. Cancers [Internet]. 2024 Feb 13;16(4):764. Available from: http://dx.doi.org/10.3390/cancers16040764

Abstract

Biochemical recurrence (BCR) after primary treatments for prostate cancer (PC) is an extremely heterogeneous phase and at least a stratification into low- and high-risk cases for early progression in metastatic disease is necessary. At present, PSA-DT represents the best parameter to define low- and high-risk BCR PC, but real precision medicine is strongly suggested to define tailored management for patients with BCR. Before defining management, it is necessary to exclude the presence of low-volume metastasis associated with PSA progression using new-generation imaging, preferably with PSMA PET/CT. Low-risk BCR cases should be actively observed without early systemic therapies. Early treatment of low-risk BCR with continuous androgen deprivation therapy (ADT) can produce disadvantages such as the development of castration resistance before the appearance of metastases (non-metastatic castration-resistant PC). Patients with high-risk BCR benefit from early systemic therapy. Even with overall survival (OS) as the primary treatment endpoint, metastasis-free survival (MFS) should be used as a surrogate endpoint in clinical trials, especially in long survival stages of the disease. The EMBARK study has greatly influenced the management of high-risk BCR, by introducing the concept of anticipation and intensification through the use of androgen receptor signaling inhibitors (ARSIs) and ADT combination therapy. In high-risk (PSA-DT ≤ 9 months) BCR cases, the combination of enzalutamide with leuprolide significantly improves MFS when compared to leuprolide alone, maintaining an unchanged quality of life in the asymptomatic phase of the disease. The possibility of using ARSIs alone in this early disease setting is suggested by the EMBARK study (arm with enzalutamide alone) with less evidence than with the intensification of the combination therapy.

Continued use versus discontinuation of enzalutamide plus leuprolide intensified therapy upon reaching undetectable PSA levels needs to be better defined with further analysis. Real-world analysis must verify the significant results obtained in the context of a phase 3 study.

Keywords:

androgen deprivation therapy, androgen receptor signaling inhibitors, biochemical recurrence, prostatic neoplasm

<u>Original Lay Summary</u>

The risk of early progression to metastatic disease in the event of biochemical recurrence (BCR) after primary treatment for prostate cancer (PC) is extremely variable. PSA-doubling time (DT) is the best parameter currently available to define patients who are at a higher risk of progression, but a single temporal parameter is not sufficient to describe the heterogeneity of this condition. A real precision medicine approach is strongly suggested to offer tailored management to patients with biochemical relapse. At present, patients with low-risk BCR should be offered active observation, while patients with high-risk BCR are likely to benefit from early systemic therapy. Results from the EMBARK study are likely to impact how physicians choose to manage high-risk BCR. The introduction of the concept of anticipation and intensification of treatment through the use of androgen receptor signaling inhibitors (ARSIs) and ADT combination therapy in this indolent but crucial phase is the main innovation of the study. The aim of this review is to provide a comprehensive understanding of the definition, classification, diagnosis, and treatment modalities of BCR, with a focus on the latest innovations and ongoing trials in the management of high-risk BCR.

**Assessment Category**	**Score**	**Assessment Category**	**Score**	**Assessment Category**	**Score**
Comprehension Score Rater 1	4	Gunning Fog Score	21.0	Words	196
Comprehension Score Rater 2	4	SMOG Index	15.1	Complex words	48
Reading Age (years)	22.5	Coleman Liau Index	15.6	Percentage of complex words	24.49
Flesch-Kincaid Reading Ease	20.9	Automated Readability Index	17.7	Average words per sentence	28.0

Flesch-Kincaid Grade Level	17.3	Sentences	7	Average syllables per word	1.86

Lay Summary (Simple Prompt)

After prostate cancer treatment, some men experience a rise in their PSA levels, known as biochemical recurrence (BCR). Managing BCR can be challenging because the risk of cancer spreading varies from person to person. This study explores how new strategies can improve treatment by focusing on two key ideas: starting therapy earlier (anticipation) and using stronger treatments (intensification) when needed. For men at low risk of cancer spread, careful monitoring without immediate treatment is often the best approach to avoid unnecessary side effects. However, men at higher risk benefit from early treatment with a combination of hormone therapies, which can delay cancer progression while maintaining a good quality of life. New imaging techniques help detect hidden cancer spread early, guiding more precise treatment decisions. These findings aim to improve patient outcomes by tailoring therapies based on individual risk and disease progression.

**Assessment Category**	**Score**	**Assessment Category**	**Score**	**Assessment Category**	**Score**
Comprehension Score Rater 1	4	Gunning Fog Score	16.0	Words	141
Comprehension Score Rater 2	4	SMOG Index	11.6	Complex words	28
Reading Age (years)	20.5	Coleman Liau Index	17.5	Percentage of complex words	19.86
Flesch-Kincaid Reading Ease	31.6	Automated Readability Index	15.3	Average words per sentence	20.14

Flesch-Kincaid Grade Level	13.9	Sentences	7	Average syllables per word	1.83

<u>Lay Summary (Extended Prompt)</u>

After prostate cancer treatment, some men experience a rise in PSA levels, called biochemical recurrence (BCR), which may signal the cancer's return. This study explores how new strategies can help manage BCR more effectively. By using advanced imaging (like PSMA PET/CT), doctors can detect hidden cancer spread early. For men at low risk of cancer progression, close monitoring without immediate treatment is often best to avoid unnecessary side effects. However, men at high risk benefit from starting treatment early with hormone therapies like androgen deprivation therapy (ADT) combined with drugs that block cancer growth signals, such as enzalutamide. The EMBARK study shows that this combination improves outcomes without reducing quality of life. Ongoing research is needed to determine how long treatment should continue once PSA levels drop. These findings aim to help doctors personalize care, ensuring patients receive the right treatment at the right time.

**Assessment Category**	**Score**	**Assessment Category**	**Score**	**Assessment Category**	**Score**
Comprehension Score Rater 1	4	Gunning Fog Score	14.1	Words	145
Comprehension Score Rater 2	4	SMOG Index	10.3	Complex words	25
Reading Age (years)	18.5	Coleman Liau Index	16.8	Percentage of complex words	17.24
Flesch-Kincaid Reading Ease	42.6	Automated Readability Index	13.7	Average words per sentence	18.13
Flesch-Kincaid Grade Level	11.8	Sentences	8	Average syllables per word	1.72

Manuscript 182

Mahaveer Chand N, Tekumalla PK, Rosenberg MT, Dobi A, Ali A, Miller GM, et al. Filamin A Is a Prognostic Serum Biomarker for Differentiating Benign Prostatic Hyperplasia from Prostate Cancer in Caucasian and African American Men. Cancers [Internet]. 2024 Feb 8;16(4):712. Available from: http://dx.doi.org/10.3390/cancers16040712

Abstract

Prostate cancer represents a significant health risk to aging men, in which diagnostic challenges to the identification of aggressive cancers remain unmet. Prostate cancer screening is driven by the prostate-specific antigen (PSA); however, in men with benign prostatic hyperplasia (BPH) due to an enlarged prostate and elevated PSA, PSA's screening utility is diminished, resulting in many unnecessary biopsies. To address this issue, we previously identified a cleaved fragment of Filamin A (FLNA) protein (as measured with IP-MRM mass spectrometry assessment as a prognostic biomarker for stratifying BPH from prostate cancer and subsequently evaluated its expanded utility in Caucasian (CA) and African American (AA) men. All men had a negative digital rectal examination (DRE) and PSA between 4 and 10 ng/mL and underwent prostate biopsy. In AA men, FLNA serum levels exhibited diagnostic utility for stratifying BPH from patients with aggressive prostate cancer (0.71 AUC and 12.2 OR in 48 men with BPH and 60 men with PCa) and outperformed PSA (0.50 AUC, 2.2 OR). In CA men, FLNA serum levels also exhibited diagnostic utility for stratifying BPH from patients with aggressive prostate cancer (0.74 AUC and 19.4 OR in 191 men with BPH and 109 men with PCa) and outperformed PSA (0.46 AUC, 0.32 OR). Herein, we established FLNA alone as a serum biomarker for stratifying men with BPH vs. those with high Gleason (7-10) prostate cancers compared to the current diagnostic paradigm of using PSA. This approach demonstrates clinical actionability of FLNA alone without the requirement of prostate volume measurement as a test with utility in AA and CA men and represents a significant opportunity to decrease the number of unnecessary biopsies in aggressive prostate cancer diagnoses.

Keywords:

FLNA, benign prostatic hyperplasia, biomarker, prostate cancer, prostate specific antigen

<u>Original Lay Summary</u>

Prostate Cancer represents a significant health risk for men, especially African American men, despite the availability of PSA testing. Although PSA testing is the current gold-standard test for identifying at-risk men, an increased PSA level may arise from Benign Prostatic Hyperplasia instead of Prostate Cancer. For men with BPH, PSA testing may lead them to undergo unnecessary biopsies. As an alternative to PSA, we have previously described a Filamin-A and prostate volume based biomarker test with superior performance. To simplify this test, we removed the requirement of prostate volume measurement. Herein, we present results of this updated test utilizing Filamin-A alone in Caucasian and African American men. Filamin-A demonstrates superior predictive power compared to PSA in both patient populations. By reliably separating benign conditions from aggressive prostate cancer, this test would reduce the health care burden resulting from unnecessary prostate biopsies.

**Assessment Category**	**Score**	**Assessment Category**	**Score**	**Assessment Category**	**Score**
Comprehension Score Rater 1	5	Gunning Fog Score	15.5	Words	146
Comprehension Score Rater 2	5	SMOG Index	12.7	Complex words	39
Reading Age (years)	20.5	Coleman Liau Index	17.3	Percentage of complex words	26.71
Flesch-Kincaid Reading Ease	24.3	Automated Readability Index	14.1	Average words per sentence	18.25
Flesch-Kincaid Grade Level	14.4	Sentences	8	Average syllables per word	1.94

Lay Summary (Simple Prompt)

Prostate cancer can be difficult to detect, especially in men with benign prostatic hyperplasia (BPH), a common condition that enlarges the prostate and affects the accuracy of the usual screening test, the prostate-specific antigen (PSA) test. This study explored a new blood marker called Filamin A (FLNA) to see if it could better distinguish between BPH and aggressive prostate cancer in both Caucasian and African American men. Researchers measured FLNA levels in men who had elevated PSA levels and underwent prostate biopsies. The results showed that FLNA was more reliable than PSA in identifying aggressive prostate cancer, helping to reduce unnecessary biopsies. Importantly, FLNA worked well in both racial groups, making it a promising tool for improving prostate cancer diagnosis and reducing the risks and stress associated with unnecessary procedures.

**Assessment Category**	**Score**	**Assessment Category**	**Score**	**Assessment Category**	**Score**
Comprehension Score Rater 1	4	Gunning Fog Score	19.0	Words	131
Comprehension Score Rater 2	4	SMOG Index	14.6	Complex words	32
Reading Age (years)	22.5	Coleman Liau Index	16.5	Percentage of complex words	24.43
Flesch-Kincaid Reading Ease	24.6	Automated Readability Index	17.5	Average words per sentence	26.2
Flesch-Kincaid Grade Level	16.3	Sentences	5	Average syllables per word	1.84

Lay Summary (Extended Prompt)

Prostate cancer is a serious health concern for men as they age, and current screening methods like the prostate-specific antigen (PSA) test can lead to unnecessary biopsies, especially in men with benign prostate enlargement (BPH). This study looked at a protein called Filamin A (FLNA) to see if it could better distinguish between BPH and aggressive prostate cancer in Caucasian and African American men. Researchers tested blood samples from men with similar PSA levels and found that FLNA was more accurate than PSA in identifying aggressive prostate cancer in both groups. Using FLNA as a simple blood test could help doctors reduce unnecessary prostate biopsies while still catching cancers that need treatment. This finding is important because it offers a new way to improve prostate cancer diagnosis without additional invasive procedures.

**Assessment Category**	**Score**	**Assessment Category**	**Score**	**Assessment Category**	**Score**
Comprehension Score Rater 1	4	Gunning Fog Score	16.9	Words	132
Comprehension Score Rater 2	4	SMOG Index	12.9	Complex words	25
Reading Age (years)	20.5	Coleman Liau Index	15.6	Percentage of complex words	18.94
Flesch-Kincaid Reading Ease	33.9	Automated Readability Index	16.9	Average words per sentence	26.4
Flesch-Kincaid Grade Level	15.1	Sentences	5	Average syllables per word	1.73

Manuscript 183

Chen QH, Munoz E, Ashong D. Insight into Recent Advances in Degrading Androgen Receptor for Castration-Resistant Prostate Cancer. Cancers [Internet]. 2024 Feb 4;16(3):663. Available from: http://dx.doi.org/10.3390/cancers16030663

Abstract

Induced protein degradation has emerged as an innovative drug discovery approach, complementary to the classical method of suppressing protein function. The androgen receptor signaling pathway has been identified as the primary driving force in the development and progression of lethal castration-resistant prostate cancer. Since androgen receptor degraders function differently from androgen receptor antagonists, they hold the promise to overcome the drug resistance challenges faced by current therapeutics. Proteolysis-targeting chimeras (PROTACs), monomeric degraders, hydrophobic tagging, molecular glues, and autophagic degradation have demonstrated their capability in downregulating intracellular androgen receptor concentrations. The potential of these androgen receptor degraders to treat castration-resistant prostate cancer is substantiated by the advancement of six PROTACs and two monomeric androgen receptor degraders into phase I or II clinical trials. Although the chemical structures, in vitro and in vivo data, and degradation mechanisms of androgen receptor degraders have been reviewed, it is crucial to stay updated on recent advances in this field as novel androgen receptor degraders and new strategies continue to emerge. This review thus provides insight into recent advancements in this paradigm, offering an overview of the progress made since 2020.

Keywords:

PROTACs, androgen receptor, autophagy, castration-resistant prostate cancer, hydrophobic tag, molecular glue, protein degradation, ubiquitin proteosome system

Original Lay Summary

A promising approach to combat aggressive prostate cancer involves androgen receptor degraders, which break down the crucial protein driving cancer progression. In contrast to traditional drugs, these degraders offer a potential solution to drug resistance. Methods like PROTACs have shown promise in reducing androgen receptor levels in cells and clinical benefits in patients. Six PROTACs and two other degraders have entered early clinical trials for potential treatment. While progress has been made in understanding these degraders, it is essential to stay updated on emerging developments. This article provides an overview of advancements in this field since 2020.

**Assessment Category**	**Score**	**Assessment Category**	**Score**	**Assessment Category**	**Score**
Comprehension Score Rater 1	5	Gunning Fog Score	18.4	Words	97
Comprehension Score Rater 2	5	SMOG Index	12.7	Complex words	29
Reading Age (years)	20.5	Coleman Liau Index	18.3	Percentage of complex words	29.9
Flesch-Kincaid Reading Ease	31.7	Automated Readability Index	13.9	Average words per sentence	16.17
Flesch-Kincaid Grade Level	12.9	Sentences	6	Average syllables per word	1.88

<u>Lay Summary (Simple Prompt)</u>

Prostate cancer can become resistant to treatments that lower testosterone levels, making it harder to control. This type of cancer is known as castration-resistant prostate cancer. Scientists are exploring new ways to treat it by targeting the androgen receptor, a key protein that helps prostate cancer grow. Instead of just blocking this protein, researchers have found methods to break it down completely. These methods include innovative strategies like PROTACs, molecular glues, and autophagy, which help remove the androgen receptor from cancer cells. Several of these new treatments are already being tested in clinical trials. This review highlights the latest progress in these promising therapies, showing how they could offer new hope for patients with advanced prostate cancer.

**Assessment Category**	**Score**	**Assessment Category**	**Score**	**Assessment Category**	**Score**
Comprehension Score Rater 1	5	Gunning Fog Score	13.9	Words	118
Comprehension Score Rater 2	5	SMOG Index	10.1	Complex words	21
Reading Age (years)	18.5	Coleman Liau Index	17.1	Percentage of complex words	17.8
Flesch-Kincaid Reading Ease	46.3	Automated Readability Index	13.3	Average words per sentence	16.86
Flesch-Kincaid Grade Level	11.0	Sentences	7	Average syllables per word	1.69

Lay Summary (Extended Prompt)

Castration-resistant prostate cancer is a tough form of cancer that keeps growing even when treatments lower male hormone levels. A key player in this cancer is the androgen receptor, which helps cancer cells grow. Current treatments aim to block this receptor, but over time, cancer can become resistant to these drugs. Scientists are now exploring new ways to tackle this problem by not just blocking the receptor, but actually breaking it down inside the cancer cells. This review looks at the latest progress in these new treatments, including special molecules called PROTACs, monomeric degraders, and other innovative methods. Some of these new drugs are already being tested in early clinical trials. Understanding these advances could lead to better treatments for patients, offering hope for more effective ways to control this aggressive cancer.

**Assessment Category**	**Score**	**Assessment Category**	**Score**	**Assessment Category**	**Score**
Comprehension Score Rater 1	5	Gunning Fog Score	13.3	Words	133
Comprehension Score Rater 2	5	SMOG Index	9.8	Complex words	20
Reading Age (years)	18.5	Coleman Liau Index	15.5	Percentage of complex words	15.04
Flesch-Kincaid Reading Ease	48.9	Automated Readability Index	13.1	Average words per sentence	19.0
Flesch-Kincaid Grade Level	11.2	Sentences	7	Average syllables per word	1.64

Manuscript 184

Brassetti A, Cacciatore L, Bove AM, Anceschi U, Proietti F, Misuraca L, et al. The Impact of Physical Activity on the Outcomes of Active Surveillance in Prostate Cancer Patients: A Scoping Review. Cancers [Internet]. 2024 Feb 1;16(3):630. Available from: http://dx.doi.org/10.3390/cancers16030630

Abstract

Introduction: Active surveillance has emerged as a valid therapeutic option in patients with low-risk prostate cancer, allowing for the deferral of definitive treatment until the time of possible disease progression. Although it is known that physical activity plays a protective role in the onset and progression of this tumor, its impact on patients with low-risk disease who are managed with active surveillance remains unclear. Our scoping review aims to summarize the existing evidence on this subject.

Evidence acquisition: On 9 April 2023, a systematic search was conducted using the PubMed and Scopus databases. The search employed the combination of the following terms: ("prostate cancer" OR "prostate tumor") AND ("active surveillance") AND ("physical activity" OR "physical exercise" OR "physical intensive activity" OR "intensive exercise") AND ("lifestyle"). Out of the 506 identified articles, 9 were used for the present scoping review, and their results were reported according to the PRISMA-ScR statement.

Evidence synthesis: We discovered a lack of uniformity in the assessment of PA and its stratification by intensity. There was no consensus regarding what constitutes cancer progression in patients choosing expectant management. In terms of the impact of PA on AS outcomes, conflicting results were reported: some authors found no correlation, while others (six of total studies included) revealed that active men experience smaller increases in PSA levels compared to their sedentary

counterparts. Additionally, higher levels of exercise were associated with a significantly reduced risk of PCa reclassification.

Conclusion: Due to the heterogeneity of the methodologies used in the available studies and the conflicting results reported, it is not possible to draw definitive conclusions concerning the role physical activity may play in the risk of prostate cancer progression in men managed with active surveillance.

Keywords:

active surveillance, disease progression, exercise, lifestyle, physical activity, prostate cancer

<u>Original Lay Summary</u>

The present review explores the impact of physical activity (PA) on patients with prostate cancer (PCa) managed through active surveillance (AS). Specifically, the correlation between the duration, frequency, and intensity of physical exercise and the risk of tumor reclassification has been investigated. We pointed out inconsistencies in PA assessment and conflicting findings regarding its association with AS outcomes. Nevertheless, several studies suggest that active individuals may experience reduced risk of PCa progression. This renders PA a cost-effective approach for minimizing the need for definitive treatment, thereby classifying it within tertiary prevention strategies. Given the increasing number of patients diagnosed with PCa, this strategy may assume significant relevance for the public health in the coming years.

**Assessment Category**	**Score**	**Assessment Category**	**Score**	**Assessment Category**	**Score**
Comprehension Score Rater 1	5	Gunning Fog Score	19.8	Words	116
Comprehension Score Rater 2	5	SMOG Index	13.9	Complex words	35
Reading Age (years)	21.5	Coleman Liau Index	19.9	Percentage of complex words	30.17
Flesch-Kincaid Reading Ease	17.3	Automated Readability Index	16.8	Average words per sentence	19.33
Flesch-Kincaid Grade Level	15.7	Sentences	6	Average syllables per word	2.01

Lay Summary (Simple Prompt)

Prostate cancer is common among men, and for those with low-risk disease, active surveillance allows doctors to monitor the cancer closely without immediate treatment. Physical activity is known to help prevent and slow down many diseases, but its effect on prostate cancer under active surveillance is less clear. This review looked at nine studies to see if exercise impacts prostate cancer progression in these patients. The results were mixed: some studies found no link, while others showed that men who are more physically active tend to have slower increases in PSA levels, a marker of cancer activity, and a lower chance of their cancer becoming more aggressive. However, because the studies used different methods and had varying results, we cannot say for sure how much physical activity helps. Still, staying active is generally good for overall health and may offer benefits for men with prostate cancer on active surveillance.

**Assessment Category**	**Score**	**Assessment Category**	**Score**	**Assessment Category**	**Score**
Comprehension Score Rater 1	5	Gunning Fog Score	15.6	Words	150
Comprehension Score Rater 2	5	SMOG Index	10.8	Complex words	21
Reading Age (years)	19.5	Coleman Liau Index	14.6	Percentage of complex words	14.0
Flesch-Kincaid Reading Ease	42.7	Automated Readability Index	15.4	Average words per sentence	25.0
Flesch-Kincaid Grade Level	13.5	Sentences	6	Average syllables per word	1.64

Lay Summary (Extended Prompt)

This study explores how physical activity might affect prostate cancer patients who are monitored closely without immediate treatment, a strategy known as active surveillance. While exercise is known to help prevent cancer from getting worse, it's unclear how it impacts patients with low-risk prostate cancer. The researchers reviewed studies that looked at the relationship between physical activity and cancer progression. They found mixed results: some studies showed no link, while others suggested that more exercise may help slow cancer progression by keeping certain markers, like PSA levels, lower. However, the studies used different methods, making it hard to draw firm conclusions. This research helps highlight the need for more consistent studies to better understand how exercise might help men with prostate cancer avoid treatment or slow the disease.

**Assessment Category**	**Score**	**Assessment Category**	**Score**	**Assessment Category**	**Score**
Comprehension Score Rater 1	5	Gunning Fog Score	15.1	Words	129
Comprehension Score Rater 2	5	SMOG Index	10.8	Complex words	21
Reading Age (years)	19.5	Coleman Liau Index	17.2	Percentage of complex words	16.28
Flesch-Kincaid Reading Ease	43.4	Automated Readability Index	15.8	Average words per sentence	21.5
Flesch-Kincaid Grade Level	12.6	Sentences	6	Average syllables per word	1.67

Manuscript 185

Zhou W, Zhang W, Yan S, Zhang K, Wu H, Chen H, et al. Novel Therapeutic Targets on the Horizon: An Analysis of Clinical Trials on Therapies for Bone Metastasis in Prostate Cancer. Cancers [Internet]. 2024 Jan 31;16(3):627. Available from: http://dx.doi.org/10.3390/cancers16030627

Abstract

In the absence of early detection and initial treatment, prostate cancer often progresses to an advanced stage, frequently spreading to the bones and significantly impacting patients' well-being and healthcare resources. Therefore, managing patients with prostate cancer that has spread to the bones often involves using bone-targeted medications like bisphosphonates and denosumab to enhance bone structure and minimize skeletal complications. Additionally, researchers are studying the tumor microenvironment and biomarkers to understand the mechanisms and potential treatment targets for bone metastases in prostate cancer. A literature search was conducted to identify clinical studies from 2013 to 2023 that focused on pain, performance status, or quality of life as primary outcomes. The analysis included details such as patient recruitment, prior palliative therapies, baseline characteristics, follow-up, and outcome reporting. The goal was to highlight the advancements and trends in bone metastasis research in prostate cancer over the past decade, with the aim of developing strategies to prevent and treat bone metastases and improve the quality of life and survival rates for prostate cancer patients.

Keywords:

bone metastasis, bone-targeted therapies, prostate cancer, skeletal-related event

Original Lay Summary

Prostate cancer is the most common non-cutaneous malignancy among men in the United States. Bone metastases and health are crucial issues in prostate cancer patient management. The aim of our review was to summarize the novel therapeutic targets of prostate cancer with bone metastasis, including treatments to palliate pain and therapies to prevent complications of osseous metastasis. Additionally, our study offers a comprehensive overview of the research on bone metastases in prostate cancer, which can be a valuable resource for researchers in the field.

**Assessment Category**	**Score**	**Assessment Category**	**Score**	**Assessment Category**	**Score**
Comprehension Score Rater 1	5	Gunning Fog Score	17.4	Words	85
Comprehension Score Rater 2	5	SMOG Index	12.9	Complex words	20
Reading Age (years)	20.5	Coleman Liau Index	16.7	Percentage of complex words	23.53
Flesch-Kincaid Reading Ease	28.0	Automated Readability Index	15.2	Average words per sentence	21.25
Flesch-Kincaid Grade Level	14.6	Sentences	4	Average syllables per word	1.86

Lay Summary (Simple Prompt)

Prostate cancer can spread to the bones, causing serious health problems and reducing patients' quality of life. This study looked at clinical trials from the past decade to find better ways to manage bone complications in men with prostate cancer. Researchers focused on treatments that aim to strengthen bones and reduce pain, such as bone-targeted medications like bisphosphonates and denosumab. They also explored new areas, including how the cancer interacts with its surrounding environment and the role of biological markers in bone spread. By analyzing data from studies conducted between 2013 and 2023, the research highlights important trends and advancements in treating bone metastases. The goal is to improve strategies for preventing and managing bone-related issues, ultimately enhancing the quality of life and survival rates for men with prostate cancer.

**Assessment Category**	**Score**	**Assessment Category**	**Score**	**Assessment Category**	**Score**
Comprehension Score Rater 1	5	Gunning Fog Score	17.3	Words	132
Comprehension Score Rater 2	5	SMOG Index	12.5	Complex words	28
Reading Age (years)	20.5	Coleman Liau Index	16.3	Percentage of complex words	21.21
Flesch-Kincaid Reading Ease	40.3	Automated Readability Index	15.3	Average words per sentence	22.0
Flesch-Kincaid Grade Level	13.1	Sentences	6	Average syllables per word	1.7

Lay Summary (Extended Prompt)

Bone metastasis, or the spread of prostate cancer to the bones, is a serious health issue that affects many patients. This study looks at treatments for bone metastasis in prostate cancer, including drugs that help protect the bones and reduce pain. Researchers analyzed clinical trials from the last decade to learn more about these treatments. The aim was to identify new ways to manage bone metastasis and improve patients' lives by reducing pain and helping them stay active. The findings from this research could help doctors better treat prostate cancer patients whose disease has spread to the bones, improving their overall health, quality of life, and survival. The study also highlights the importance of ongoing research to find better treatments for this common and challenging cancer complication.

**Assessment Category**	**Score**	**Assessment Category**	**Score**	**Assessment Category**	**Score**
Comprehension Score Rater 1	5	Gunning Fog Score	14.1	Words	127
Comprehension Score Rater 2	5	SMOG Index	10.1	Complex words	18
Reading Age (years)	18.5	Coleman Liau Index	15.3	Percentage of complex words	14.17
Flesch-Kincaid Reading Ease	50.1	Automated Readability Index	14.0	Average words per sentence	21.17
Flesch-Kincaid Grade Level	11.5	Sentences	6	Average syllables per word	1.6

Manuscript 186

Wang L, Sanders J, Ward JF, Lee SR, Poenisch F, Swanson DM, et al. A Novel Polymer-Encapsulated Multi-Imaging Modality Fiducial Marker with Positive Signal Contrast for Image-Guided Radiation Therapy. Cancers [Internet]. 2024 Jan 31;16(3):625. Available from: http://dx.doi.org/10.3390/cancers16030625

Abstract

Background: Current fiducial markers (FMs) in external-beam radiotherapy (EBRT) for prostate cancer (PCa) cannot be positively visualized on magnetic resonance imaging (MRI) and create dose perturbation and significant imaging artifacts on computed tomography (CT) and MRI. We report our initial experience with clinical imaging of a novel multimodality FM, NOVA.

Methods: We tested Gold Anchor [G-FM], BiomarC [carbon, C-FM], and NOVA FMs in phantoms imaged with kilovoltage (kV) X-rays, transrectal ultrasound (TRUS), CT, and MRI. Artifacts of the FMs on CT were quantified by the relative streak artifacts level (rSAL) metric. Proton dose perturbations (PDPs) were measured with Gafchromic EBT3 film, with FMs oriented either perpendicular to or parallel with the beam axis. We also tested the performance of NOVA-FMs in a patient.

Results: NOVA-FMs were positively visualized on all 4 imaging modalities tested. The rSAL on CT was 0.750 ± 0.335 for 2-mm reconstructed slices. In F-tests, PDP was associated with marker type and depth of measurement (p < 10-6); at 5-mm depth, PDP was significantly greater for the G-FM (12.9%, p = 10-6) and C-FM (6.0%, p = 0.011) than NOVA (4.5%). EBRT planning with MRI/CT image co-registration and daily alignments using NOVA-FMs in a patient was feasible and reproducible.

Conclusions: NOVA-FMs were positively visible and produced less PDP than G-FMs or C-FMs. NOVA-FMs facilitated MRI/CT fusion and identification of regions of interest.

Keywords:

NOVA, fiducial marker, magnetic resonance imaging, prostate cancer, radiation therapy

<u>Original Lay Summary</u>

External-beam radiotherapy (EBRT) is the standard of care for prostate cancer (PCa). Precision is critical to target the intraprostatic dominant lesion(s) and limit treatment-related toxicity from unnecessary irradiation of surrounding normal tissues. Currently, to achieve this goal, fiducial markers (FMs) are used in EBRT planning and treatment delivery. However, some of the features of current FMs (e.g., poor visibility on MRI, substantial CT and MR artifacts, radiation dose perturbations) affect treatment accuracy. The aim of our study was to evaluate the performance of a novel FM (NOVA) in phantoms and in a patient. We found that NOVA FMs were positively visualized on ultrasound (US), kV X-ray, CT, and MR images, had reduced CT and MR artifacts, and caused less proton dose perturbations than Gold Anchor and BiomarC (carbon) FMs. Our findings indicate the value of NOVA FMs in daily treatment positioning, MRI/CT co-image registration, and MRI-based treatment-plan generation for patients with PCa undergoing EBRT.

**Assessment Category**	**Score**	**Assessment Category**	**Score**	**Assessment Category**	**Score**
Comprehension Score Rater 1	5	Gunning Fog Score	15.1	Words	162
Comprehension Score Rater 2	5	SMOG Index	11.3	Complex words	34
Reading Age (years)	18.5	Coleman Liau Index	14.4	Percentage of complex words	20.99
Flesch-Kincaid Reading Ease	38.7	Automated Readability Index	11.7	Average words per sentence	18.0
Flesch-Kincaid Grade Level	12.3	Sentences	9	Average syllables per word	1.77

<u>Lay Summary (Simple Prompt)</u>

In radiation therapy for prostate cancer, small markers called fiducial markers help doctors precisely target the tumor. However, current markers can cause blurry images on scans like MRI and CT, making it harder to plan treatment accurately. This study tested a new marker, called NOVA, designed to show up clearly on different types of medical imaging without causing image problems. Researchers compared NOVA with two commonly used markers and found that NOVA provided clearer images across all imaging methods and caused fewer issues with radiation dose delivery. In a real patient, the NOVA marker made it easier to align images from MRI and CT scans, improving the accuracy of treatment. This new marker could help doctors deliver radiation therapy more precisely, potentially leading to better outcomes for patients.

**Assessment Category**	**Score**	**Assessment Category**	**Score**	**Assessment Category**	**Score**
Comprehension Score Rater 1	5	Gunning Fog Score	15.7	Words	128
Comprehension Score Rater 2	5	SMOG Index	11.3	Complex words	23
Reading Age (years)	19.5	Coleman Liau Index	15.6	Percentage of complex words	17.97
Flesch-Kincaid Reading Ease	37.8	Automated Readability Index	14.3	Average words per sentence	21.33
Flesch-Kincaid Grade Level	13.3	Sentences	6	Average syllables per word	1.74

Lay Summary (Extended Prompt)

This study introduces a new type of marker, called NOVA, designed to improve the accuracy of radiation therapy for prostate cancer. Current markers used for treatment are difficult to see in important imaging tests like MRI and CT scans, and they can cause errors in treatment. The NOVA marker was tested using various imaging methods, including CT, MRI, and ultrasound, and was found to be clearly visible without causing significant problems. The researchers also found that NOVA caused less interference in treatment planning compared to older markers. This means that using NOVA could help doctors plan and guide radiation therapy more accurately, leading to better treatment results for prostate cancer patients. These findings may help improve the precision and effectiveness of cancer treatment in the future.

**Assessment Category**	**Score**	**Assessment Category**	**Score**	**Assessment Category**	**Score**
Comprehension Score Rater 1	5	Gunning Fog Score	14.4	Words	126
Comprehension Score Rater 2	5	SMOG Index	10.3	Complex words	19
Reading Age (years)	19.5	Coleman Liau Index	15.8	Percentage of complex words	15.08
Flesch-Kincaid Reading Ease	41.2	Automated Readability Index	14.4	Average words per sentence	21.0
Flesch-Kincaid Grade Level	12.7	Sentences	6	Average syllables per word	1.71

Manuscript 187

Lohm G, Knörnschild F, Neumann K, Budach V, Schwartz S, Burock S, et al. Salvage Radiotherapy for Relapsed Prostate Cancer after Radical Prostatectomy Is Associated with Normal Life Expectancy. Cancers [Internet]. 2024 Jan 26;16(3):534. Available from: http://dx.doi.org/10.3390/cancers16030534

Abstract

In patients with prostate cancer (PCa), salvage radiotherapy (SRT) for biochemical progression (BP) after radical prostatectomy (RP) improves PCa-specific survival. However, no prospective randomized trials have compared the effect of SRT with untreated patients. In this analysis of 151 patients who received SRT for post-RP BP, we compared their overall survival (OS) with virtual, age-matched controls (n = 151,000) retrieved from government life tables. We also investigated the risk factors associated with BP and OS and compared the prostate-specific antigen (PSA) doubling times (DTs) before and after SRT for patients with BP. The median follow-up was 9.3 years for BP and 17.4 years for OS. The risk factors significantly affecting BP were Gleason score (p p = 0.003), and negative surgical margins (p = 0.003). None of these risk factors were associated with OS. In 93 patients with BP after SRT, the median PSADT was significantly prolonged compared with pre-SRT values (3.7 vs. 8.3 months, p p = 0.112), and life expectancy was similar, likely due to the survival benefit of SRT. The prolonged PSADT after SRT further supports the beneficial role of SRT in this patient population. However, subsequent treatments were not systematically recorded, which may have affected the results.

Keywords:

PSA doubling time, biochemical relapse, overall survival, prostate cancer, salvage radiotherapy

Original Lay Summary

Radiotherapy is a treatment option for prostate cancer patients who have increasing prostate-specific antigen values after prostatectomy. However, this treatment has never been compared directly in a single study with the course of untreated relapsed prostate cancer. We analyzed the course of prostate-specific antigen values before and after radiotherapy by calculating prostate-specific antigen doubling times. Prolongation of these values after radiotherapy indicated slower progression with this treatment. The survival analysis also suggests the advantages of salvage radiotherapy. In our analysis, when comparing our cohort with an age-matched cohort from life tables, it was observed that patients had a normal life expectancy after radiotherapy for recurrent prostate cancer following prostatectomy.

**Assessment Category**	**Score**	**Assessment Category**	**Score**	**Assessment Category**	**Score**
Comprehension Score Rater 1	4	Gunning Fog Score	17.8	Words	113
Comprehension Score Rater 2	4	SMOG Index	12.9	Complex words	30
Reading Age (years)	22.5	Coleman Liau Index	20.0	Percentage of complex words	26.55
Flesch-Kincaid Reading Ease	13.3	Automated Readability Index	16.6	Average words per sentence	18.83
Flesch-Kincaid Grade Level	16.1	Sentences	6	Average syllables per word	2.06

Lay Summary (Simple Prompt)

This study explored how effective salvage radiotherapy (SRT) is for men with prostate cancer who experienced a return of the disease after having their prostate surgically removed. The researchers compared the life expectancy of 151 men who received SRT with that of 151,000 healthy individuals of the same age. They found that men who underwent SRT lived just as long as those without prostate cancer, suggesting that SRT may help maintain a normal life expectancy. Additionally, SRT slowed the growth rate of prostate-specific antigen (PSA), a marker used to monitor prostate cancer progression. The results highlight the important role of SRT in improving survival and controlling cancer progression in men with relapsed prostate cancer after surgery.

**Assessment Category**	**Score**	**Assessment Category**	**Score**	**Assessment Category**	**Score**
Comprehension Score Rater 1	5	Gunning Fog Score	16.2	Words	118
Comprehension Score Rater 2	5	SMOG Index	11.6	Complex words	20
Reading Age (years)	19.5	Coleman Liau Index	14.9	Percentage of complex words	16.95
Flesch-Kincaid Reading Ease	40.9	Automated Readability Index	15.0	Average words per sentence	23.6
Flesch-Kincaid Grade Level	13.4	Sentences	5	Average syllables per word	1.68

<u>Lay Summary (Extended Prompt)</u>

This study looks at how salvage radiotherapy (SRT) can help men with prostate cancer whose cancer comes back after surgery. The researchers wanted to see if SRT improves life expectancy for these men. They compared the survival rates of 151 men who had SRT with a much larger group of healthy, age-matched men. They found that men who received SRT lived just as long as the general population. The study also found that certain factors, like cancer severity and surgery results, influenced the chance of cancer returning, but they didn't affect overall survival. The findings suggest that SRT can be an effective treatment, extending life expectancy and helping slow down cancer growth, giving hope to patients with relapsed prostate cancer after surgery.

**Assessment Category**	**Score**	**Assessment Category**	**Score**	**Assessment Category**	**Score**
Comprehension Score Rater 1	5	Gunning Fog Score	13.4	Words	123
Comprehension Score Rater 2	5	SMOG Index	9.5	Complex words	16
Reading Age (years)	17.5	Coleman Liau Index	13.6	Percentage of complex words	13.01
Flesch-Kincaid Reading Ease	53.3	Automated Readability Index	12.4	Average words per sentence	20.5
Flesch-Kincaid Grade Level	10.9	Sentences	6	Average syllables per word	1.57

Manuscript 188

Maekawa S, Takata R, Obara W. Molecular Mechanisms of Prostate Cancer Development in the Precision Medicine Era: A Comprehensive Review. Cancers [Internet]. 2024 Jan 25;16(3):523. Available from: http://dx.doi.org/10.3390/cancers16030523

Abstract

The progression of prostate cancer (PCa) relies on the activation of the androgen receptor (AR) by androgens. Despite efforts to block this pathway through androgen deprivation therapy, resistance can occur through several mechanisms, including the abnormal activation of AR, resulting in castration-resistant PCa following the introduction of treatment. Mutations, amplifications, and splicing variants in AR-related genes have garnered attention in this regard. Furthermore, recent large-scale next-generation sequencing analysis has revealed the critical roles of AR and AR-related genes, as well as the DNA repair, PI3K, and cell cycle pathways, in the onset and progression of PCa. Moreover, research on epigenomics and microRNA has increasingly become popular; however, it has not translated into the development of effective therapeutic strategies. Additionally, treatments targeting homologous recombination repair mutations and the PI3K/Akt pathway have been developed and are increasingly accessible, and multiple clinical trials have investigated the efficacy of immune checkpoint inhibitors. In this comprehensive review, we outline the status of PCa research in genomics and briefly explore potential future developments in the field of epigenetic modifications and microRNAs.

Keywords:

cancer immunology, drug resistance, epigenomics, immune checkpoint inhibitor, mutagenesis, next-generation sequencing, non-coding RNAs, poly (ADP-ribose) polymerase inhibitors, prostate cancer, signal pathways

Original Lay Summary

Prostate cancer (PCa) is characterized by various genomic alterations that play a pivotal role in carcinogenesis. Efforts in precision medicine aimed at improving diagnosis, prevention, and surveillance based on genetic alterations are advancing. Notably, no tumor markers surpass prostate-specific antigen in specificity, and existing treatments primarily target the androgen receptor axis, with exceptions for patients with alterations in homologous recombination repair-related genes, such as BRCA1/2 and ATM, who may benefit from poly (ADP-ribose) polymerase inhibitors. In order to delineate the current state of research on PCa, we provide an overview of cutting-edge genomic research, including genome alterations, cancer immunology, and non-coding RNAs, in PCa. These aspects are relevant to comprehending the molecular mechanisms underlying health disparities, PCa initiation and progression, and drug resistance in the precision medicine era.

**Assessment Category**	**Score**	**Assessment Category**	**Score**	**Assessment Category**	**Score**
Comprehension Score Rater 1	5	Gunning Fog Score	25.5	Words	130
Comprehension Score Rater 2	5	SMOG Index	18.0	Complex words	49
Reading Age (years)	24.0	Coleman Liau Index	20.2	Percentage of complex words	37.69
Flesch-Kincaid Reading Ease	0.2	Automated Readability Index	20.3	Average words per sentence	26.0
Flesch-Kincaid Grade Level	19.7	Sentences	5	Average syllables per word	2.13

Lay Summary (Simple Prompt)

Prostate cancer is a common disease in men, and understanding how it develops is key to finding better treatments. This review looks at how certain genes and biological pathways, like those controlling hormones, DNA repair, and cell growth, contribute to prostate cancer. While therapies that block male hormones can slow the disease, some cancers become resistant. New research using advanced genetic techniques has found specific changes in genes and other factors, such as microRNAs and epigenetic modifications, that may help explain this resistance. Although these discoveries are promising, many have not yet led to new treatments. However, drugs targeting DNA repair issues and immune-based therapies are showing potential. This review provides an overview of what scientists have learned about prostate cancer at the molecular level and discusses future directions for improving diagnosis and treatment, offering hope for more effective therapies.

**Assessment Category**	**Score**	**Assessment Category**	**Score**	**Assessment Category**	**Score**
Comprehension Score Rater 1	5	Gunning Fog Score	16.3	Words	141
Comprehension Score Rater 2	5	SMOG Index	11.8	Complex words	29
Reading Age (years)	20.5	Coleman Liau Index	17.5	Percentage of complex words	20.57
Flesch-Kincaid Reading Ease	38.2	Automated Readability Index	15.3	Average words per sentence	20.14
Flesch-Kincaid Grade Level	12.9	Sentences	7	Average syllables per word	1.75

Lay Summary (Extended Prompt)

This study explores how prostate cancer develops and why treatments often stop working over time. The researchers focus on how certain genes and pathways in the body, like those controlled by male hormones, play a big role in the disease. Even though treatments that lower hormone levels can help, many prostate cancers become resistant, and the disease continues to grow. The study looks at new technology that helps us understand these changes in greater detail, including how genes and tiny molecules inside cells might affect the cancer. The findings are important because they could lead to better treatments, such as those targeting specific genes or using new immune-based therapies. This research helps scientists understand more about prostate cancer and could eventually lead to more effective therapies for patients.

**Assessment Category**	**Score**	**Assessment Category**	**Score**	**Assessment Category**	**Score**
Comprehension Score Rater 1	4	Gunning Fog Score	13.6	Words	129
Comprehension Score Rater 2	4	SMOG Index	9.5	Complex words	16
Reading Age (years)	18.5	Coleman Liau Index	15.6	Percentage of complex words	12.4
Flesch-Kincaid Reading Ease	49.3	Automated Readability Index	14.4	Average words per sentence	21.5
Flesch-Kincaid Grade Level	11.7	Sentences	6	Average syllables per word	1.6

Manuscript 189

Mita K, Izumi K, Goriki A, Tasaka R, Hatayama T, Shima T, et al. Enzalutamide versus Abiraterone Plus Prednisolone for Nonmetastatic Castration-Resistant Prostate Cancer: A Sub-Analysis from the ENABLE Study for PCa. Cancers [Internet]. 2024 Jan 24;16(3):508. Available from: http://dx.doi.org/10.3390/cancers16030508

Abstract

Enzalutamide (ENZ) and abiraterone plus prednisolone (ABI) can improve the survival of patients with castration-resistant prostate cancer (CRPC). However, the agent that is more effective against nonmetastatic CRPC remains unclear. To evaluate the agent that can be used as the first-line treatment for CRPC, an investigator-initiated, multicenter, randomized controlled trial (ENABLE Study for PCa) including both metastatic and nonmetastatic CRPC was conducted in Japan. The prostate-specific antigen (PSA) response rate, overall survival, some essential survival endpoints, and safety of patients with nonmetastatic CRPC were also analyzed. In this subanalysis, 15 and 26 patients in the ENZ and ABI arms, respectively, presented with nonmetastatic CRPC. There was no significant difference in terms of the PSA response rate between the ENZ and ABI arms (80% and 64%, respectively; $p = 0.3048$). The overall survival did not significantly differ between the two arms (HR: 0.68; 95% CI: 0.22-2.14, $p = 0.5260$). No significant differences were observed in terms of radiographic progression-free survival and cancer-specific survival between the ENZ and ABI arms (HR: 0.81; 95% CI: 0.35-1.84; $p = 0.6056$ and HR: 0.72; 95% CI: 0.19-2.73; $p = 0.6443$, respectively). Only four and six patients in the ENZ and ABI arms, respectively, had ≥grade 3 adverse events. ABI and ENZ had similar efficacy and safety profiles in patients with nonmetastatic CRPC.

Keywords:

abiraterone, castration-resistant, enzalutamide, nonmetastatic

Original Lay Summary

The efficacy of abiraterone plus prednisolone (ABI) against nonmetastatic castration-resistant prostate cancer (CRPC) remains unclear. To evaluate enzalutamide and ABI as the first-line treatment for CRPC, we conducted the randomized controlled trial including both metastatic and nonmetastatic CRPC. As a sub-analysis, we focused on nonmetastatic CRPC in this study. ABI and enzalutamide had similar efficacy and safety profiles in patients with nonmetastatic CRPC.

**Assessment Category**	**Score**	**Assessment Category**	**Score**	**Assessment Category**	**Score**
Comprehension Score Rater 1	5	Gunning Fog Score	18.7	Words	66
Comprehension Score Rater 2	5	SMOG Index	12.9	Complex words	20
Reading Age (years)	21.5	Coleman Liau Index	19.1	Percentage of complex words	30.3
Flesch-Kincaid Reading Ease	9.4	Automated Readability Index	14.7	Average words per sentence	16.5
Flesch-Kincaid Grade Level	16.1	Sentences	4	Average syllables per word	2.14

<u>Lay Summary (Simple Prompt)</u>

This study compared two treatments, enzalutamide and abiraterone plus prednisolone, for men with nonmetastatic castration-resistant prostate cancer, a type of prostate cancer that no longer responds to hormone therapy but has not spread to other parts of the body. Researchers analyzed data from a larger clinical trial in Japan to see which treatment worked better in slowing the cancer's progression and improving survival. The results showed no significant difference between the two treatments in terms of reducing prostate-specific antigen (PSA) levels, overall survival, or preventing the cancer from worsening. Both treatments also had similar safety profiles, with only a few patients experiencing severe side effects. These findings suggest that either treatment option could be considered effective and safe for men with this type of prostate cancer.

**Assessment Category**	**Score**	**Assessment Category**	**Score**	**Assessment Category**	**Score**
Comprehension Score Rater 1	5	Gunning Fog Score	18.7	Words	128
Comprehension Score Rater 2	5	SMOG Index	13.4	Complex words	27
Reading Age (years)	22.5	Coleman Liau Index	17.3	Percentage of complex words	21.09
Flesch-Kincaid Reading Ease	28.8	Automated Readability Index	17.8	Average words per sentence	25.6
Flesch-Kincaid Grade Level	15.6	Sentences	5	Average syllables per word	1.8

<u>Lay Summary (Extended Prompt)</u>

This study compared two treatments, enzalutamide and abiraterone plus prednisolone, for patients with prostate cancer that has stopped responding to standard hormone therapy but has not yet spread to other parts of the body. The research aimed to determine which treatment works better for these patients. The study included 15 patients treated with enzalutamide and 26 patients treated with abiraterone plus prednisolone. The results showed that both treatments had similar effects on cancer control, with no significant differences in survival rates or the progression of cancer. Both treatments were also found to be similarly safe, with a few patients experiencing serious side effects. This research helps doctors understand that both treatments could be used for these patients, offering more options for managing this difficult condition.

**Assessment Category**	**Score**	**Assessment Category**	**Score**	**Assessment Category**	**Score**
Comprehension Score Rater 1	5	Gunning Fog Score	15.7	Words	125
Comprehension Score Rater 2	5	SMOG Index	11.3	Complex words	23
Reading Age (years)	20.5	Coleman Liau Index	17.5	Percentage of complex words	18.4
Flesch-Kincaid Reading Ease	38.8	Automated Readability Index	15.6	Average words per sentence	20.83
Flesch-Kincaid Grade Level	13.0	Sentences	6	Average syllables per word	1.74

Manuscript 190

Oka R, Utsumi T, Noro T, Suzuki Y, Iijima S, Sugizaki Y, et al. Progress in Oligometastatic Prostate Cancer: Emerging Imaging Innovations and Therapeutic Approaches. Cancers [Internet]. 2024 Jan 24;16(3):507. Available from: http://dx.doi.org/
10.3390/cancers16030507

Abstract

Prostate cancer (PCa) exhibits a spectrum of heterogeneity, from indolent to highly aggressive forms, with approximately 10-20% of patients experiencing metastatic PCa. Oligometastatic PCa, characterized by a limited number of metastatic lesions in specific anatomical locations, has gained attention due to advanced imaging modalities. Although patients with metastatic PCa typically receive systemic therapy, personalized treatment approaches for oligometastatic PCa are emerging, including surgical and radiotherapeutic interventions. This comprehensive review explores the latest developments in the field of oligometastatic PCa, including its biological mechanisms, advanced imaging techniques, and relevant clinical studies. Oligometastatic PCa is distinct from widespread metastases and presents challenges in patient classification. Imaging plays a crucial role in identifying and characterizing oligometastatic lesions, with new techniques such as prostate-specific membrane antigen positron emission tomography demonstrating a remarkable efficacy. The management strategies encompass cytoreductive surgery, radiotherapy targeting the primary tumor, and metastasis-directed therapy for recurrent lesions. Ongoing clinical trials are evaluating the effectiveness of these approaches. Oligometastatic PCa occupies a unique position between locally advanced and high-volume metastatic diseases. While a universally accepted definition and standardized diagnostic criteria are still evolving, emerging imaging technologies and therapeutic strategies hold promise for improving the patient outcomes in this intermediate stage of PCa.

Keywords:

PSMA-PET, cytoreductive surgery, metastasis-directed therapy, oligometastatic prostate cancer, stereotactic body radiation therapy

Original Lay Summary

In recent years, there has been growing interest in oligometastatic prostate cancer (PCa). Unlike more widespread forms of the disease, oligometastatic PCa involves only a limited number of cancerous lesions in specific areas of the body. This review delves into the latest advancements in our understanding of oligometastatic PCa, including how it works on a biological level, the use of advanced imaging techniques to spot it, and the various treatment approaches being explored. For patients with this intermediate-stage cancer, there is hope on the horizon, as personalized treatments such as surgery and targeted radiation therapy are showing promise. The findings from ongoing research may ultimately lead to better outcomes for individuals facing this unique challenge, bridging the gap between localized and widespread PCa.

**Assessment Category**	**Score**	**Assessment Category**	**Score**	**Assessment Category**	**Score**
Comprehension Score Rater 1	5	Gunning Fog Score	19.3	Words	124
Comprehension Score Rater 2	5	SMOG Index	13.9	Complex words	29
Reading Age (years)	22.5	Coleman Liau Index	17.1	Percentage of complex words	23.39
Flesch-Kincaid Reading Ease	19.3	Automated Readability Index	17.3	Average words per sentence	24.8
Flesch-Kincaid Grade Level	16.7	Sentences	5	Average syllables per word	1.92

<u>Lay Summary (Simple Prompt)</u>

Prostate cancer can behave very differently from person to person, ranging from slow-growing to aggressive forms. Some men develop a specific type called oligometastatic prostate cancer, where the cancer has spread but only to a few areas. This stage is important because new imaging technologies, like PSMA-PET scans, help doctors find these small cancer spots more accurately. With better imaging, treatments can be more personalized, combining surgery or targeted radiation with standard therapies to control the disease more effectively. This review looks at the latest advances in how we detect and treat oligometastatic prostate cancer.

**Assessment Category**	**Score**	**Assessment Category**	**Score**	**Assessment Category**	**Score**
Comprehension Score Rater 1	5	Gunning Fog Score	15.2	Words	97
Comprehension Score Rater 2	5	SMOG Index	11.0	Complex words	18
Reading Age (years)	19.5	Coleman Liau Index	16.7	Percentage of complex words	18.56
Flesch-Kincaid Reading Ease	36.3	Automated Readability Index	14.2	Average words per sentence	19.4
Flesch-Kincaid Grade Level	13.0	Sentences	5	Average syllables per word	1.78

Lay Summary (Extended Prompt)

Oligometastatic prostate cancer is a type of cancer that has spread to only a few specific areas in the body, rather than widely. This research explores how new imaging techniques can help doctors find these small cancer spots and how treatments, like surgery and targeted radiation, might help control the disease. The study highlights the potential of advanced imaging methods, such as a special scan called PSMA-PET, to detect these limited cancer spread areas more accurately. The research also looks at new ways to treat patients, focusing on personalized approaches that could lead to better outcomes for those with this intermediate stage of cancer. By improving how doctors detect and treat oligometastatic prostate cancer, this research could offer hope for better survival and quality of life for patients.

**Assessment Category**	**Score**	**Assessment Category**	**Score**	**Assessment Category**	**Score**
Comprehension Score Rater 1	5	Gunning Fog Score	15.6	Words	129
Comprehension Score Rater 2	5	SMOG Index	11.0	Complex words	18
Reading Age (years)	19.5	Coleman Liau Index	15.1	Percentage of complex words	13.95
Flesch-Kincaid Reading Ease	40.3	Automated Readability Index	16.2	Average words per sentence	25.8
Flesch-Kincaid Grade Level	14.0	Sentences	5	Average syllables per word	1.66

Manuscript 191

Pyun JH, Ko YH, Kim SW, Son NH. The Short- and Long-Term Anticipation of Prostate Cancer Incidence in Korea: Based on Social Aging Trends and Prostate-Specific Antigen Testing Rate during the Last Decade. Cancers [Internet]. 2024 Jan 24;16(3):503. Available from: http://dx.doi.org/10.3390/cancers16030503

Abstract

The current incidence of prostate-specific antigen (PSA) testing, which plays a crucial role in detecting prostate cancer (PCa) in an aged population, is low in Korea. Reflecting these epidemiologic characteristics, we estimated the short- and long-term incidences of PCa. A regression equation model was extracted based on two critical pieces of information: (1) the distribution of newly detected PCa cases in each age group of the 50s, 60s, 70s, and over 80s from a recent period (2006-2020), and (2) the PSA testing rate (PSAr) from the previous decade (2006-2016) for each age subgroup. The incidence increased fourfold (4533 in 2006 to 16,815 in 2020), with each age subgroup accounting for 7.9% (50s), 31.4% (60s), 43.0% (70s), and 17.1% (over 80s) of cases in 2020. PSAr increased by an average of 1.08% annually. If these trends are maintained, 28,822 new cases will be diagnosed in 2030 (expected PSAr: 14.4%) and 40,478 cases in 2040 (expected PSAr: 26.4%). If a public PSA screening were implemented for men only in their 60s (assuming a PSAr of 60% in the 60s) and 70s (assuming a PSAr of 80% in the 70s) in 2030, 37,503 cases in 2030 (expected PSAr: 23.1%) and 43,719 cases in 2040 (expected PSAr: 29.9%) would be estimated. According to the projection, the incidence of PCa will increase twofold by 2034 compared to 2020. If national screening were only conducted in the 60s and 70s, a higher detection of almost threefold would be expected by 2040.

Keywords:

incidence, prostate cancer, screening

<u>Original Lay Summary</u>

This study aimed to anticipate the short- and long-term PCa incidence projected from the PSA testing rate (PSAr) trend and social aging during the last decade. We found that the incidence of PCa increased fourfold (4533 in 2006 to 16,815 in 2020), with each age subgroup accounting for 7.9% (50s), 31.4% (60s), 43.0% (70s), and 17.1% (over 80s) of cases in 2020. According to the projection of this study, the incidence of PCa will increase twofold by 2034 compared to 2020. If national screening were only carried out in the 60s and 70s age groups, a higher detection of almost threefold would be expected by 2040.

**Assessment Category**	**Score**	**Assessment Category**	**Score**	**Assessment Category**	**Score**
Comprehension Score Rater 1	5	Gunning Fog Score	9.5	Words	112
Comprehension Score Rater 2	5	SMOG Index	6.9	Complex words	11
Reading Age (years)	11.5	Coleman Liau Index	6.3	Percentage of complex words	9.82
Flesch-Kincaid Reading Ease	75.5	Automated Readability Index	3.3	Average words per sentence	14.0
Flesch-Kincaid Grade Level	6.2	Sentences	8	Average syllables per word	1.38

Lay Summary (Simple Prompt)

Prostate cancer is becoming more common in Korea, partly because the population is aging and more men are getting tested with a blood test called the prostate-specific antigen (PSA) test, which helps detect this cancer early. However, PSA testing rates are still relatively low. This study estimated how many new prostate cancer cases might occur in the future based on current testing trends and aging patterns. The results suggest that if current trends continue, the number of new cases could double by 2034 compared to 2020. The study also predicts that if Korea introduced national PSA screening for men in their 60s and 70s, prostate cancer detection rates could nearly triple by 2040. These findings highlight the potential impact of wider PSA testing on early cancer detection, which could help improve outcomes for many men in the future.

**Assessment Category**	**Score**	**Assessment Category**	**Score**	**Assessment Category**	**Score**
Comprehension Score Rater 1	5	Gunning Fog Score	12.7	Words	139
Comprehension Score Rater 2	5	SMOG Index	8.3	Complex words	12
Reading Age (years)	17.5	Coleman Liau Index	13.0	Percentage of complex words	8.63
Flesch-Kincaid Reading Ease	51.2	Automated Readability Index	13.2	Average words per sentence	23.17
Flesch-Kincaid Grade Level	11.9	Sentences	6	Average syllables per word	1.56

Lay Summary (Extended Prompt)

This study looks at how common prostate cancer is in Korea and how the rate of testing for prostate cancer might affect future cases. Researchers analyzed data from 2006 to 2020 to estimate how many new cases will occur in the future, based on trends in testing and age. They found that prostate cancer cases have increased significantly, especially among older adults, and expect even more cases in the coming years if current trends continue. The study also suggests that if more men in their 60s and 70s get tested, many more cases of prostate cancer could be detected, helping people get treatment earlier. This information is important for understanding how prostate cancer might change over time and how testing could help reduce its impact.

**Assessment Category**	**Score**	**Assessment Category**	**Score**	**Assessment Category**	**Score**
Comprehension Score Rater 1	5	Gunning Fog Score	13.5	Words	125
Comprehension Score Rater 2	5	SMOG Index	8.7	Complex words	11
Reading Age (years)	17.5	Coleman Liau Index	12.5	Percentage of complex words	8.8
Flesch-Kincaid Reading Ease	51.5	Automated Readability Index	13.7	Average words per sentence	25.0
Flesch-Kincaid Grade Level	12.3	Sentences	5	Average syllables per word	1.54

Manuscript 192

Liu J, Cundy TP, Woon DTS, Lawrentschuk N. A Systematic Review on Artificial Intelligence Evaluating Metastatic Prostatic Cancer and Lymph Nodes on PSMA PET Scans. Cancers [Internet]. 2024 Jan 23;16(3):486. Available from: http://dx.doi.org/10.3390/cancers16030486

Abstract

Early detection of metastatic prostate cancer (mPCa) is crucial. Whilst the prostate-specific membrane antigen (PSMA) PET scan has high diagnostic accuracy, it suffers from inter-reader variability, and the time-consuming reporting process. This systematic review was registered on PROSPERO (ID CRD42023456044) and aims to evaluate AI's ability to enhance reporting, diagnostics, and predictive capabilities for mPCa on PSMA PET scans. Inclusion criteria covered studies using AI to evaluate mPCa on PSMA PET, excluding non-PSMA tracers. A search was conducted on Medline, Embase, and Scopus from inception to July 2023. After screening 249 studies, 11 remained eligible for inclusion. Due to the heterogeneity of studies, meta-analysis was precluded. The prediction model risk of bias assessment tool (PROBAST) indicated a low overall risk of bias in ten studies, though only one incorporated clinical parameters (such as age, and Gleason score). AI demonstrated a high accuracy (98%) in identifying lymph node involvement and metastatic disease, albeit with sensitivity variation (62-97%). Advantages included distinguishing bone lesions, estimating tumour burden, predicting treatment response, and automating tasks accurately. In conclusion, AI showcases promising capabilities in enhancing the diagnostic potential of PSMA PET scans for mPCa, addressing current limitations in efficiency and variability.

Keywords:

PSMA PET, artificial intelligence, convolutional neural network, deep learning, machine learning, prostate cancer

<u>Original Lay Summary</u>

This systematic review demonstrated that artificial intelligence (AI) can help detect metastatic prostate cancer with or without lymph node involvement on prostate-specific membrane antigen (PSMA) PET scans with high accuracy. Additional benefits of AI include the ability to estimate the volume of metastatic cancer, prognosticate, and differentiate bony metastasis from post-radiotherapy bone changes. AI can also improve workflow by helping to standardize reporting and automate time-consuming tasks. However, given the variable sensitivity and positive predictive value of AI, it is recommended that an experienced nuclear medicine physician proofread the final report. Larger studies producing more consistent results are needed before AI can be fully integrated into PSMA reporting.

**Assessment Category**	**Score**	**Assessment Category**	**Score**	**Assessment Category**	**Score**
Comprehension Score Rater 1	5	Gunning Fog Score	21.1	Words	111
Comprehension Score Rater 2	5	SMOG Index	15.0	Complex words	34
Reading Age (years)	23.5	Coleman Liau Index	19.5	Percentage of complex words	30.63
Flesch-Kincaid Reading Ease	9.8	Automated Readability Index	17.9	Average words per sentence	22.2
Flesch-Kincaid Grade Level	17.4	Sentences	5	Average syllables per word	2.06

<u>Lay Summary (Simple Prompt)</u>

Detecting the spread of prostate cancer early is vital for effective treatment. This study reviewed how artificial intelligence (AI) can help improve the accuracy and speed of identifying prostate cancer that has spread to other parts of the body using PSMA PET scans, a common imaging technique. Researchers analyzed 11 studies that used AI to detect cancer in lymph nodes and bones. The findings showed that AI can identify cancer with high accuracy, especially in lymph nodes, although its sensitivity varied. AI also helped estimate the size of tumors, predict how well treatments might work, and reduce the time doctors need to analyze scans. Overall, AI shows great potential in making prostate cancer diagnoses faster and more reliable, which could lead to better outcomes for patients.

**Assessment Category**	**Score**	**Assessment Category**	**Score**	**Assessment Category**	**Score**
Comprehension Score Rater 1	4	Gunning Fog Score	14.4	Words	126
Comprehension Score Rater 2	4	SMOG Index	10.6	Complex words	20
Reading Age (years)	18.5	Coleman Liau Index	14.6	Percentage of complex words	15.87
Flesch-Kincaid Reading Ease	44.5	Automated Readability Index	13.4	Average words per sentence	21.0
Flesch-Kincaid Grade Level	12.3	Sentences	6	Average syllables per word	1.67

Lay Summary (Extended Prompt)

This study looks at how artificial intelligence (AI) can help improve the accuracy and speed of diagnosing metastatic prostate cancer using a special scan called PSMA PET. The researchers reviewed several studies to see how well AI performs in identifying cancer spread to lymph nodes and other parts of the body. The results show that AI can be very accurate, with 98% accuracy in detecting cancer in lymph nodes, but there were some differences in how well it worked in different studies. AI can also help by finding bone lesions, predicting how the cancer might respond to treatment, and speeding up the process of reading the scans. This research suggests that AI could improve the way doctors use PSMA PET scans to detect prostate cancer and help make the process faster and more reliable.

**Assessment Category**	**Score**	**Assessment Category**	**Score**	**Assessment Category**	**Score**
Comprehension Score Rater 1	4	Gunning Fog Score	15.2	Words	134
Comprehension Score Rater 2	5	SMOG Index	10.1	Complex words	15
Reading Age (years)	18.5	Coleman Liau Index	12.6	Percentage of complex words	11.19
Flesch-Kincaid Reading Ease	49.6	Automated Readability Index	14.7	Average words per sentence	26.8
Flesch-Kincaid Grade Level	13.0	Sentences	5	Average syllables per word	1.54

Manuscript 193

Büttner T, Dietrich D, Zarbl R, Klümper N, Ellinger J, Krausewitz P, et al. Feasibility of Monitoring Response to Metastatic Prostate Cancer Treatment with a Methylation-Based Circulating Tumor DNA Approach. Cancers [Internet]. 2024 Jan 23;16(3):482. Available from: http://dx.doi.org/10.3390/cancers16030482

Abstract

Background: Metastatic prostate cancer (mPCA) poses challenges in treatment response assessment, particularly in cases where prostate-specific antigen (PSA) levels do not reliably indicate a response. Liquid biopsy, focusing on circulating cell-free DNA (ccfDNA) methylation analysis as a proxy for circulating tumor DNA, offers a non-invasive and cost-effective approach. This study explores the potential of two methylation markers, short stature homeobox 2 (SHOX2) and Septin 9 (SEPT9), as on-mPCA-treatment biomarkers.

Methods: Plasma samples were collected from 11 mPCA patients undergoing various treatments. Quantitative assessment of hypermethylated SHOX2 (mSHOX2) and SEPT9 (mSEPT9) levels in ccfDNA was conducted through methylation-specific real-time PCR. Early and overall dynamics of PSA, mSHOX2, and mSEPT9 were analyzed. Statistical evaluation employed Wilcoxon tests.

Results: mSHOX2 demonstrated a significant decline post-treatment in patients with a radiographic treatment response as well as in an early treatment setting. mSEPT9 and PSA exhibited non-significant declines. In individual cases, biomarker dynamics revealed unique patterns compared to PSA.

Discussion: mSHOX2 and mSEPT9 exhibit dynamics on mPCA treatment. This proof-of-concept study lays the groundwork for further investigation into these markers as valuable additions to treatment response monitoring in mPCA. Further validation in larger cohorts is essential for establishing clinical utility.

Keywords:

SEPT9, SHOX2, biomarkers, liquid biopsy, metastatic prostate cancer

<u>Original Lay Summary</u>

This study tackles the challenges of assessing treatment response in advanced prostate cancer. The standard test, measuring the PSA protein in blood, lacks reliability in some patients. We explored a method examining genetic material in blood samples. We focused on two methylation markers, SHOX2 and SEPT9, as proxies of circulating tumor DNA. We collected blood samples from 11 patients with advanced prostate cancer undergoing different treatments. The results showed that all markers showed a response to treatment, especially SHOX2. This suggests that tracking advanced prostate cancer through liquid biopsy might harbor potential to monitor treatment effectiveness. This study is designed as a feasibility assessment and starting point, and more research with a larger group of patients is needed for confirmation.

**Assessment Category**	**Score**	**Assessment Category**	**Score**	**Assessment Category**	**Score**
Comprehension Score Rater 1	5	Gunning Fog Score	13.0	Words	120
Comprehension Score Rater 2	5	SMOG Index	9.4	Complex words	21
Reading Age (years)	18.5	Coleman Liau Index	17.7	Percentage of complex words	17.5
Flesch-Kincaid Reading Ease	41.4	Automated Readability Index	12.8	Average words per sentence	15.0
Flesch-Kincaid Grade Level	11.2	Sentences	8	Average syllables per word	1.78

Lay Summary (Simple Prompt)

Monitoring how well treatments work in patients with metastatic prostate cancer can be challenging, especially when common blood tests like PSA don't give clear answers. This study explored a new, simple blood test called a liquid biopsy that looks for specific changes in tumor DNA circulating in the blood. Researchers focused on two markers, SHOX2 and SEPT9, to see if they could track how the cancer responds to treatment. Blood samples from 11 patients showed that levels of SHOX2 dropped significantly in those who responded well to therapy, even at an early stage of treatment, while SEPT9 and PSA levels were less consistent. These findings suggest that SHOX2 could become a useful tool for doctors to monitor treatment success more accurately in the future. However, more research with larger groups of patients is needed to confirm these results and help improve cancer care.

**Assessment Category**	**Score**	**Assessment Category**	**Score**	**Assessment Category**	**Score**
Comprehension Score Rater 1	5	Gunning Fog Score	13.2	Words	143
Comprehension Score Rater 2	5	SMOG Index	8.9	Complex words	14
Reading Age (years)	17.5	Coleman Liau Index	13.9	Percentage of complex words	9.79
Flesch-Kincaid Reading Ease	53.7	Automated Readability Index	14.3	Average words per sentence	23.83
Flesch-Kincaid Grade Level	11.7	Sentences	6	Average syllables per word	1.52

Lay Summary (Extended Prompt)

This study looks at a new method for monitoring how well treatments work for patients with metastatic prostate cancer, a type of cancer that has spread beyond the prostate. The researchers are testing whether they can use a blood test to measure certain changes in DNA, which could show how the cancer is responding to treatment. They focused on two specific markers, called SHOX2 and SEPT9, and found that SHOX2 levels decreased in patients whose treatment was working. This is important because current tests, like PSA levels, don't always show the true effects of treatment. The results of this study suggest that blood tests measuring these markers could become a helpful tool for doctors to track treatment progress, potentially leading to better care for patients in the future.

**Assessment Category**	**Score**	**Assessment Category**	**Score**	**Assessment Category**	**Score**
Comprehension Score Rater 1	5	Gunning Fog Score	12.7	Words	128
Comprehension Score Rater 2	5	SMOG Index	7.5	Complex words	8
Reading Age (years)	17.5	Coleman Liau Index	13.7	Percentage of complex words	6.25
Flesch-Kincaid Reading Ease	57.3	Automated Readability Index	15.0	Average words per sentence	25.6
Flesch-Kincaid Grade Level	11.6	Sentences	5	Average syllables per word	1.46

Manuscript 194

Kim M, Kim SH, Hong S, Kim YJ, Kim HR, Kim JY. Evaluation of Extra-Prostatic Extension on Deep Learning-Reconstructed High-Resolution Thin-Slice T2-Weighted Images in Patients with Prostate Cancer. Cancers [Internet]. 2024 Jan 18;16(2):413. Available from: http://dx.doi.org/10.3390/cancers16020413

Abstract

The aim of this study was to compare diagnostic performance for extra-prostatic extension (EPE) and image quality among three image datasets: conventional T2-weighted images (T2WIconv, slice thickness, 3 mm) and high-resolution thin-slice T2WI (T2WIHR, 2 mm), with and without deep learning reconstruction (DLR) in patients with prostatic cancer (PCa). A total of 88 consecutive patients (28 EPE-positive and 60 negative) diagnosed with PCa via radical prostatectomy who had undergone 3T-MRI were included. Two independent reviewers performed a crossover review in three sessions, in which each reviewer recorded five-point confidence scores for the presence of EPE and image quality using a five-point Likert scale. Pathologic topographic maps served as the reference standard. For both reviewers, T2WIconv showed better diagnostic performance than T2WIHR with and without DLR (AUCs, in order, for reviewer 1, 0.883, 0.806, and 0.772, p = 0.0006; for reviewer 2, 0.803, 0.762, and 0.745, p = 0.022). The image quality was also the best in T2WIconv, followed by T2WIHR with DLR and T2WIHR without DLR for both reviewers (median, in order, 3, 4, and 5, p conv was optimal in regard to image quality and diagnostic performance for the evaluation of EPE in patients with PCa.

Keywords:

deep learning, image reconstruction, magnetic resonance imaging (MRI), prostate cancer

<u>Original Lay Summary</u>

Extra-prostatic extension (EPE) is a well-known poor prognostic factor of prostate cancer. As such, accurate radiological diagnosis of EPE is important for urology surgeons due to its surgical implications prior to radical prostatectomy. T2-weighted imaging (T2WI) is a key sequence in prostate MRI for evaluating EPE. Recent technical advancements have allowed for the acquisition of thin-slice T2WI with resultant increased image noise. Deep learning reconstruction (DLR) techniques have been used to decrease the image noise in MRI; however, previous studies have focused mainly on image quality or acquisition times, and the efficacy of DLR regarding the diagnostic performance for EPE has not yet been reported. Our observations reveal that conventional 3 mm T2WI was better than 2 mm thin-slice T2WI with DLR with respect to diagnostic performance for EPE and image quality, thus supporting the minimal technical requirements described in the prostate imaging quality guidelines.

**Assessment Category**	**Score**	**Assessment Category**	**Score**	**Assessment Category**	**Score**
Comprehension Score Rater 1	5	Gunning Fog Score	20.9	Words	150
Comprehension Score Rater 2	5	SMOG Index	15.0	Complex words	41
Reading Age (years)	22.5	Coleman Liau Index	16.1	Percentage of complex words	27.33
Flesch-Kincaid Reading Ease	27.5	Automated Readability Index	16.6	Average words per sentence	25.0
Flesch-Kincaid Grade Level	15.6	Sentences	6	Average syllables per word	1.82

<u>Lay Summary (Simple Prompt)</u>

This study explored how well different MRI imaging techniques can detect the spread of prostate cancer beyond the prostate, known as extra-prostatic extension (EPE). Researchers compared traditional MRI images with newer high-resolution images, both with and without advanced deep learning technology to enhance image quality. They analyzed scans from 88 prostate cancer patients, comparing the accuracy and clarity of each imaging method. The results showed that conventional MRI images provided the best combination of image quality and diagnostic accuracy for identifying EPE, outperforming the newer high-resolution images, even when enhanced with deep learning. These findings suggest that, despite technological advancements, traditional MRI remains the most reliable tool for evaluating the spread of prostate cancer, which is crucial for making informed treatment decisions.

**Assessment Category**	**Score**	**Assessment Category**	**Score**	**Assessment Category**	**Score**
Comprehension Score Rater 1	5	Gunning Fog Score	19.6	Words	125
Comprehension Score Rater 2	5	SMOG Index	14.1	Complex words	30
Reading Age (years)	23.5	Coleman Liau Index	19.1	Percentage of complex words	24.0
Flesch-Kincaid Reading Ease	23.1	Automated Readability Index	19.0	Average words per sentence	25.0
Flesch-Kincaid Grade Level	16.2	Sentences	5	Average syllables per word	1.87

<u>Lay Summary (Extended Prompt)</u>

This study looks at a new way to take clearer images of the prostate to help doctors better detect cancer spread. The researchers compared different types of magnetic resonance imaging (MRI) scans in 88 patients with prostate cancer. They used two types of MRI scans: a regular scan and a new, higher-quality scan with deep learning technology to improve the images. The results showed that the regular MRI scan gave the clearest images and worked best at detecting cancer spread outside the prostate. The study's findings suggest that using deep learning to improve MRI scans could be helpful, but it may not be better than the regular method for detecting cancer spread in patients.

**Assessment Category**	**Score**	**Assessment Category**	**Score**	**Assessment Category**	**Score**
Comprehension Score Rater 1	5	Gunning Fog Score	13.4	Words	115
Comprehension Score Rater 2	5	SMOG Index	9.0	Complex words	12
Reading Age (years)	17.5	Coleman Liau Index	12.7	Percentage of complex words	10.43
Flesch-Kincaid Reading Ease	59.2	Automated Readability Index	12.9	Average words per sentence	23.0
Flesch-Kincaid Grade Level	10.7	Sentences	5	Average syllables per word	1.47

Manuscript 195

Takagi M, Hasegawa Y, Tateoka K, Takada Y, Hareyama M. Dosimetric Comparison Study of Proton Therapy Using Line Scanning versus Passive Scattering and Volumetric Modulated Arc Therapy for Localized Prostate Cancer. Cancers [Internet]. 2024 Jan 17;16(2):403. Available from: http://dx.doi.org/10.3390/cancers16020403

Abstract

Background: The proton irradiation modality has transitioned from passive scattering (PS) to pencil beam scanning. Nevertheless, the documented outcomes predominantly rely on PS.

Methods: Thirty patients diagnosed with prostate cancer were selected to assess treatment planning across line scanning (LS), PS, and volumetric modulated arc therapy (VMAT). Dose constraints encompassed clinical target volume (CTV) D98 ≥ 73.0 Gy (RBE), rectal wall V65 < 17% and V40 < 35%, and bladder wall V65 < 25% and V40 < 50%. The CTV, rectal wall, and bladder wall dose volumes were calculated and evaluated using the Freidman test.

Results: The LS technique adhered to all dose limitations. For the rectal and bladder walls, 10 (33.3%) and 21 (70.0%) patients in the PS method and 5 (16.7%) and 1 (3.3%) patients in VMAT, respectively, failed to meet the stipulated requirements. The wide ranges of the rectal and bladder wall volumes (V10-70) were lower with LS than with PS and VMAT. LS outperformed VMAT across all dose-volume rectal and bladder wall indices.

Conclusion: The LS method demonstrated a reduction in rectal and bladder doses relative to PS and VMAT, thereby suggesting the potential for mitigating toxicities.

Keywords:

line scanning, passive scattering, pencil beam scanning, prostate cancer, side effect, treatment planning

Original Lay Summary

The currently documented outcomes of proton therapy for patients with localized prostate cancer are based on passive scattering (PS). In this study, we compared the dose distribution of the line scanning (LS) method, one of the pencil beam scanning (PBS) techniques, with that of PS and volumetric modulated arc therapy (VMAT) in 30 patients. The results showed that LS could significantly reduce the wide range of rectal and bladder doses compared to the PS and VMAT while upholding the requisite target dose. The results suggest that proton therapy for prostate cancer with the PBS method may have the potential to reduce toxicities further while preserving therapeutic efficacies.

**Assessment Category**	**Score**	**Assessment Category**	**Score**	**Assessment Category**	**Score**
Comprehension Score Rater 1	5	Gunning Fog Score	17.8	Words	107
Comprehension Score Rater 2	5	SMOG Index	12.6	Complex words	19
Reading Age (years)	20.5	Coleman Liau Index	15.0	Percentage of complex words	17.76
Flesch-Kincaid Reading Ease	38.2	Automated Readability Index	16.6	Average words per sentence	26.75
Flesch-Kincaid Grade Level	14.6	Sentences	4	Average syllables per word	1.67

Lay Summary (Simple Prompt)

Prostate cancer treatment often involves radiation therapy, which can affect nearby healthy organs like the rectum and bladder, causing side effects. This study compared three different radiation techniques—line scanning (LS), passive scattering (PS), and volumetric modulated arc therapy (VMAT)—to see which method minimizes harm to these organs while effectively targeting the cancer. Researchers analyzed treatment plans from 30 prostate cancer patients, focusing on how much radiation reached the rectum and bladder. The results showed that LS was the most precise method, delivering lower radiation doses to these healthy tissues compared to PS and VMAT. This suggests that LS could reduce the risk of side effects, potentially improving the quality of life for prostate cancer patients undergoing radiation therapy.

**Assessment Category**	**Score**	**Assessment Category**	**Score**	**Assessment Category**	**Score**
Comprehension Score Rater 1	5	Gunning Fog Score	17.9	Words	119
Comprehension Score Rater 2	5	SMOG Index	12.9	Complex words	25
Reading Age (years)	21.5	Coleman Liau Index	17.9	Percentage of complex words	21.01
Flesch-Kincaid Reading Ease	29.1	Automated Readability Index	17.5	Average words per sentence	23.8
Flesch-Kincaid Grade Level	15.1	Sentences	5	Average syllables per word	1.82

Lay Summary (Extended Prompt)

This study compares different types of proton therapy used to treat prostate cancer, focusing on how well each method minimizes side effects to nearby organs like the bladder and rectum. The researchers looked at three treatment approaches: line scanning, passive scattering, and volumetric modulated arc therapy. They found that line scanning, a newer method, was the best at keeping doses to the bladder and rectum low, compared to the other two methods. In fact, fewer patients who received line scanning therapy experienced harmful side effects, such as high radiation exposure to these organs. These results suggest that line scanning might be a safer option for patients, potentially reducing the risk of side effects and improving the overall treatment experience. This research is important because it helps guide doctors toward the best treatment options for prostate cancer, improving patient care and outcomes.

**Assessment Category**	**Score**	**Assessment Category**	**Score**	**Assessment Category**	**Score**
Comprehension Score Rater 1	5	Gunning Fog Score	14.8	Words	141
Comprehension Score Rater 2	5	SMOG Index	10.3	Complex words	19
Reading Age (years)	19.5	Coleman Liau Index	16.1	Percentage of complex words	13.48
Flesch-Kincaid Reading Ease	43.2	Automated Readability Index	15.8	Average words per sentence	23.5
Flesch-Kincaid Grade Level	13.1	Sentences	6	Average syllables per word	1.65

Manuscript 196

Zhang J, Chen Z, Mao Y, He Y, Wu X, Wu J, et al. ID2 Promotes Lineage Transition of Prostate Cancer through FGFR and JAK-STAT Signaling. Cancers [Internet]. 2024 Jan 17;16(2):392. Available from: http://dx.doi.org/10.3390/cancers16020392

Abstract

The use of androgen receptor pathway inhibitors (ARPIs) has led to an increase in the proportion of AR-null prostate cancer, including neuroendocrine prostate cancer (NEPC) and double-negative prostate cancer (DNPC), but the mechanism underlying this lineage transition has not been elucidated. We found that ID2 expression was increased in AR-null prostate cancer. In vitro and in vivo studies confirmed that ID2 promotes PCa malignancy and can confer resistance to enzalutamide in PCa cells. We generated an ID2 UP50 signature, which is capable of determining resistance to enzalutamide and is valuable for predicting patient prognosis. Functional experiments showed that ID2 could activate stemness-associated JAK/STAT and FGFR signaling while inhibiting the AR signaling pathway. Our study indicates a potentially strong association between ID2 and the acquisition of a stem-like phenotype in adenocarcinoma cells, leading to resistance to androgen deprivation therapy (ADT) and next-generation ARPIs in prostate cancer.

Keywords:

FGFR signaling, ID2, ID2 UP50 signature, lineage transition, prostate cancer

<u>Original Lay Summary</u>

Androgen receptor-negative prostate cancer is one of the important mechanisms of castration resistant prostate cancer. The application of next generation androgen receptor signaling pathway inhibitors (ARPIs) has led to a gradual increase of AR-negative prostate cancer in the clinic. In this paper, we demonstrated the potential promotional role of ID2 in androgen receptor-negative prostate cancer through extensive bioinformatics analysis and experimental studies. Through transcriptome sequencing and downstream functional enrichment analysis, we found that ID2 can activate neuroendocrine or stemness-related pathways and inhibit androgen receptor signaling pathways. ID2 can activate JAK/STAT signaling pathway as well as FGFR signaling pathway to promote the acquisition of prostate cancer lineage plasticity, which in turn leads to androgen receptor-negative prostate cancer. Through cell function experiments and mouse experiments, we reveal that ID2 can promote prostate cancer evolution. Using transcriptome sequencing and publicly available clinical data, we generated ID2-related gene signatures to help determine clinical prognosis.

**Assessment Category**	**Score**	**Assessment Category**	**Score**	**Assessment Category**	**Score**
Comprehension Score Rater 1	5	Gunning Fog Score	23.8	Words	156
Comprehension Score Rater 2	5	SMOG Index	16.7	Complex words	59
Reading Age (years)	24.0	Coleman Liau Index	20.8	Percentage of complex words	37.82
Flesch-Kincaid Reading Ease	8.0	Automated Readability Index	19.0	Average words per sentence	22.29

Flesch-Kincaid Grade Level	17.7	Sentences	7	Average syllables per word	2.08

Lay Summary (Simple Prompt)

Prostate cancer can become more aggressive and resistant to treatment over time, especially when therapies block male hormones that fuel cancer growth. This study focused on a protein called ID2, which was found at higher levels in aggressive forms of prostate cancer that no longer respond to these hormone-blocking treatments. Researchers discovered that ID2 helps cancer cells change their behavior, making them more resistant to common drugs like enzalutamide. By studying cancer cells in the lab and in animal models, the team showed that ID2 activates specific biological pathways (JAK/STAT and FGFR signaling) that promote cancer growth while suppressing others that usually keep the disease in check. They also created a tool, the ID2 UP50 signature, to help predict which cancers might resist treatment. Understanding the role of ID2 could lead to better ways to identify and treat aggressive prostate cancers, improving outcomes for patients.

**Assessment Category**	**Score**	**Assessment Category**	**Score**	**Assessment Category**	**Score**
Comprehension Score Rater 1	5	Gunning Fog Score	16.0	Words	146
Comprehension Score Rater 2	5	SMOG Index	11.3	Complex words	23
Reading Age (years)	20.5	Coleman Liau Index	15.8	Percentage of complex words	15.75
Flesch-Kincaid Reading Ease	41.9	Automated Readability Index	16.0	Average words per sentence	24.33
Flesch-Kincaid Grade Level	13.5	Sentences	6	Average syllables per word	1.66

Lay Summary (Extended Prompt)

This study explores how certain changes in prostate cancer cells might make them harder to treat. Researchers focused on a protein called ID2, which was found to be more active in a type of prostate cancer that doesn't respond well to traditional treatments. By studying these changes in the lab and in animals, they discovered that ID2 helps prostate cancer cells become more aggressive and resistant to medications. The team also created a special test, called the ID2 UP50 signature, to help predict which patients might not respond to treatment. This research shows that ID2 could play a key role in making prostate cancer harder to treat, and understanding it better may help doctors develop more effective treatments in the future.

**Assessment Category**	**Score**	**Assessment Category**	**Score**	**Assessment Category**	**Score**
Comprehension Score Rater 1	4	Gunning Fog Score	13.6	Words	121
Comprehension Score Rater 2	4	SMOG Index	9.0	Complex words	12
Reading Age (years)	17.5	Coleman Liau Index	13.4	Percentage of complex words	9.92
Flesch-Kincaid Reading Ease	56.4	Automated Readability Index	14.0	Average words per sentence	24.2
Flesch-Kincaid Grade Level	11.4	Sentences	5	Average syllables per word	1.49

Manuscript 197

Moteabbed M, Bobić M, Paganetti H, Efstathiou JA. The Role of Proton Therapy for Prostate Cancer in the Setting of Hip Prosthesis. Cancers [Internet]. 2024 Jan 11;16(2):330. Available from: http://dx.doi.org/10.3390/cancers16020330

Abstract

Purpose: Given that the current standard of proton therapy (PT) for prostate cancer is through bilateral beams, this modality is typically avoided when it comes to treatment of patients with hip prosthesis. The purpose of this study was to evaluate whether novel PT methods, i.e., anterior proton beams and proton arc therapy (PArc), could be feasible options to treat this patient subpopulation. We evaluate PT methods in the context of dosimetry and robustness and compare with standard of practice volumetric modulated arc therapy (VMAT) to explore any potential benefits.

Methods: Two PT and one VMAT treatment plans were retrospectively created for 10 patients who participated in a clinical trial with a weekly repeat CT (rCT) imaging component. All plans were robustly optimized and featured: (1) combination anterior oblique and lateral proton beams (AoL), (2) PArc, and (3) VMAT. All patients had hydrogel spacers in place, which enabled safe application of anterior proton beams. The planned dose was 70 Gy (RBE) to the entire prostate gland and 50 Gy (RBE) to the proximal seminal vesicles in 28 fractions. Along with plan dose-volume metrics, robustness to setup and interfractional variations were evaluated using the weekly rCT images. The linear energy transfer (LET)-weighted dose was evaluated for PArc plans to ensure urethra sparing given the typical high-LET region at the end of range.

Results: Both PT methods were dosimetrically feasible and provided reduction of some key OAR metrics compared to VMAT except for penile bulb, while providing equally good target coverage. Significant differences in median rectum V35 (22-25%), penile bulb Dmean (5 Gy), rectum V61 (2%), right femoral head Dmean (5 Gy), and bladder V39 (4%) were found between PT and VMAT. All plans were equally robust to

variations. LET-weighted dose in urethra was equivalent to the physical dose for PArc plans and hence no added urethral toxicity was expected.

Conclusions: PT for treatment of prostate cancer patients with hip prosthesis is feasible and equivalent or potentially superior to VMAT in quality in some cases. The choice of radiotherapy regimen can be personalized based on patient characteristics to achieve the best treatment outcome.

Keywords:
hip prosthesis, prostate cancer, proton arc therapy

<u>Original Lay Summary</u>

As proton therapy evolves and becomes more accessible, it is important to explore its potential value for all patient cases to enable more informed treatment individualization. Currently, external beam radiotherapy for prostate cancer patients with hip prosthesis is limited to photons because traditional proton therapy beams interfere with the implanted hardware. The goal of this study was to investigate the dosimetric feasibility and robustness of novel proton therapy techniques, i.e., proton arc therapy and anterior beam directions for prostate cancer patients with unilateral hip prosthesis. It was demonstrated that proton therapy for prostate cancer in the setting of hip prosthesis is feasible and could potentially reduce the dose to some critical organs and hence reduce toxicities.

**Assessment Category**	**Score**	**Assessment Category**	**Score**	**Assessment Category**	**Score**
Comprehension Score Rater 1	5	Gunning Fog Score	19.4	Words	117
Comprehension Score Rater 2	5	SMOG Index	13.7	Complex words	34
Reading Age (years)	21.5	Coleman Liau Index	18.0	Percentage of complex words	29.06
Flesch-Kincaid Reading Ease	20.0	Automated Readability Index	15.3	Average words per sentence	19.5
Flesch-Kincaid Grade Level	15.3	Sentences	6	Average syllables per word	1.97

Lay Summary (Simple Prompt)

Prostate cancer patients with hip prostheses often face challenges when receiving proton therapy, a precise form of radiation treatment, because metal implants can interfere with the treatment's accuracy. This study explored two advanced proton therapy methods—anterior proton beams and proton arc therapy—to determine if they could safely and effectively treat these patients. By comparing these approaches to the standard radiation technique (VMAT), researchers found that both proton methods were feasible, provided excellent cancer coverage, and even reduced radiation exposure to some healthy tissues like the rectum and bladder. Importantly, these new techniques did not increase the risk of side effects to sensitive areas like the urethra. The results suggest that proton therapy can be a personalized, effective, and safe option for prostate cancer patients with hip prostheses, potentially improving treatment outcomes.

**Assessment Category**	**Score**	**Assessment Category**	**Score**	**Assessment Category**	**Score**
Comprehension Score Rater 1	5	Gunning Fog Score	19.0	Words	131
Comprehension Score Rater 2	5	SMOG Index	13.6	Complex words	28
Reading Age (years)	23.5	Coleman Liau Index	19.3	Percentage of complex words	21.37
Flesch-Kincaid Reading Ease	23.3	Automated Readability Index	19.7	Average words per sentence	26.2
Flesch-Kincaid Grade Level	16.5	Sentences	5	Average syllables per word	1.85

<u>Lay Summary (Extended Prompt)</u>

This study explores whether proton therapy (PT) can be used safely and effectively for prostate cancer patients who have a hip prosthesis. Typically, standard PT is avoided for these patients because the prosthesis can interfere with the treatment. The researchers tested new methods of PT, including proton arc therapy and using proton beams from the front of the body. They compared these new approaches to the standard treatment method, volumetric modulated arc therapy (VMAT), in terms of how well they target the cancer and spare healthy tissue. The results showed that the proton therapy methods were safe and provided similar or even better outcomes than the standard method in some cases, with less damage to nearby organs. This study suggests that proton therapy may be a good option for patients with both prostate cancer and hip prostheses, offering personalized treatment choices for better results.

**Assessment Category**	**Score**	**Assessment Category**	**Score**	**Assessment Category**	**Score**
Comprehension Score Rater 1	5	Gunning Fog Score	14.9	Words	144
Comprehension Score Rater 2	5	SMOG Index	10.3	Complex words	19
Reading Age (years)	19.5	Coleman Liau Index	14.9	Percentage of complex words	13.19
Flesch-Kincaid Reading Ease	44.4	Automated Readability Index	15.2	Average words per sentence	24.0
Flesch-Kincaid Grade Level	13.0	Sentences	6	Average syllables per word	1.63

Manuscript 198

Kiś J, Góralczyk M, Sikora D, Stępień E, Drop B, Polz-Dacewicz M. Can the Epstein–Barr Virus Play a Role in the Development of Prostate Cancer? Cancers [Internet]. 2024 Jan 11;16(2):328. Available from: http://dx.doi.org/10.3390/cancers16020328

Abstract

Prostate cancer (PCa) is the fourth most frequently diagnosed cancer worldwide, accounting for 7.3% of all cancers. PCa mortality is the fifth most common cause of cancer death. Despite well-known factors influencing the development of PCa, such as age, race/ethnicity and family history, many researchers have raised the possibility of persistent infections with oncogenic viruses. Therefore, we aimed to assess the frequency of Epstein-Barr virus (EBV) DNA in tissue collected from PCa patients. Next, the frequency and the level of Epstein-Barr virus capsid antigen (EBVCA) and Epstein-Barr nuclear antigen 1 (EBNA1) antibodies in both IgA and IgG classes were measured. The antibody titer was also analyzed depending on the risk group, Gleason score (GS) and tumor, node, metastasis (TNM) classification. Serum samples were analyzed using the Microblot-Array EBV IgM, IgA and IgG test kits. The study group consisted of 115 patients diagnosed and histopathologically confirmed with PCa. In 49% of patients included in the study, EBV DNA was detected in the tumor tissue. The studies showed both higher seroprevalence and higher antibody titers in patients with EBV-positive PCa compared to patients with EBV-negative PCa. We also observed a dependence of antibody titer on pathological features, such as GS, risk group and T stage.

Keywords:

EBNA1, EBV, EBVCA, prostate cancer

Original Lay Summary

Prostate cancer (PCa) is a serious men's health problem worldwide. Some authors report the occurrence of the Epstein–Barr virus (EBV), a well-known oncovirus, in PCa. Therefore, in this study, PCa tissues were screened for the presence of EBV. Then, the frequency and titer of antibodies against EBV in the serum of EBV-positive and EBV-negative patients were compared. The results obtained showed a higher frequency and level of Epstein–Barr virus capsid antigen (EBVCA) and Epstein–Barr nuclear antigen 1 (EBNA1) antibodies in patients with EBV-positive PCa. Higher levels of tested antibodies were observed in more advanced stages of the disease (high-risk group, high Gleason score (GS) and stage T2). These observations may suggest a role for EBV in the development and/or progression of PCa.

**Assessment Category**	**Score**	**Assessment Category**	**Score**	**Assessment Category**	**Score**
Comprehension Score Rater 1	5	Gunning Fog Score	12.6	Words	127
Comprehension Score Rater 2	5	SMOG Index	9.8	Complex words	20
Reading Age (years)	17.5	Coleman Liau Index	13.8	Percentage of complex words	15.75
Flesch-Kincaid Reading Ease	51.9	Automated Readability Index	11.3	Average words per sentence	18.14
Flesch-Kincaid Grade Level	10.5	Sentences	7	Average syllables per word	1.61

Lay Summary (Simple Prompt)

Prostate cancer is one of the most common cancers worldwide, but its exact causes are not fully understood. While factors like age and family history are known risks, some researchers believe certain infections might also play a role. This study focused on the Epstein-Barr virus (EBV), a virus linked to other types of cancer, to see if it could be involved in prostate cancer development. We analyzed tissue samples and blood from 115 prostate cancer patients to detect EBV DNA and measure specific antibodies that show the body's response to the virus. EBV was found in nearly half of the tumor samples, and patients with EBV in their cancer tissue had higher levels of antibodies compared to those without the virus. The antibody levels also varied depending on how advanced the cancer was. These findings suggest EBV might contribute to prostate cancer development, which could help improve future prevention or treatment strategies.

**Assessment Category**	**Score**	**Assessment Category**	**Score**	**Assessment Category**	**Score**
Comprehension Score Rater 1	5	Gunning Fog Score	13.2	Words	153
Comprehension Score Rater 2	5	SMOG Index	9.1	Complex words	17
Reading Age (years)	17.5	Coleman Liau Index	13.7	Percentage of complex words	11.11
Flesch-Kincaid Reading Ease	49.7	Automated Readability Index	13.1	Average words per sentence	21.86
Flesch-Kincaid Grade Level	11.8	Sentences	7	Average syllables per word	1.59

Lay Summary (Extended Prompt)

This study investigates whether a virus called Epstein-Barr virus (EBV) could be linked to the development of prostate cancer, a common cancer in men. Researchers tested tissue samples from 115 prostate cancer patients to see if EBV DNA was present in the tumors. They also measured the levels of certain antibodies related to EBV in the patients' blood. The study found that nearly half of the patients had EBV DNA in their tumor tissue, and those with EBV-positive prostate cancer had higher levels of EBV-related antibodies. The results suggest that EBV might play a role in prostate cancer, especially in how aggressive the cancer might be. These findings could lead to better understanding of prostate cancer and potentially new ways to treat or prevent it.

**Assessment Category**	**Score**	**Assessment Category**	**Score**	**Assessment Category**	**Score**
Comprehension Score Rater 1	5	Gunning Fog Score	12.3	Words	128
Comprehension Score Rater 2	5	SMOG Index	8.3	Complex words	12
Reading Age (years)	16.5	Coleman Liau Index	12.7	Percentage of complex words	9.38
Flesch-Kincaid Reading Ease	53.0	Automated Readability Index	12.1	Average words per sentence	21.33
Flesch-Kincaid Grade Level	11.2	Sentences	6	Average syllables per word	1.56

Manuscript 199

Baston C, Preda A, Iordache A, Olaru V, Surcel C, Sinescu I, et al. How to Integrate Prostate Cancer Biomarkers in Urology Clinical Practice: An Update. Cancers [Internet]. 2024 Jan 11;16(2):316. Available from: http://dx.doi.org/10.3390/cancers16020316

Abstract

Nowadays, the management of prostate cancer has become more and more challenging due to the increasing number of available treatment options, therapeutic agents, and our understanding of its carcinogenesis and disease progression. Moreover, currently available risk stratification systems used to facilitate clinical decision-making have limitations, particularly in providing a personalized and patient-centered management strategy. Although prognosis and prostate cancer-specific survival have improved in recent years, the heterogenous behavior of the disease among patients included in the same risk prognostic group negatively impacts not only our clinical decision-making but also oncological outcomes, irrespective of the treatment strategy. Several biomarkers, along with available tests, have been developed to help clinicians in difficult decision-making scenarios and guide management strategies. In this review article, we focus on the scientific evidence that supports the clinical use of several biomarkers considered by professional urological societies (and included in uro-oncological guidelines) in the diagnosis process and specific difficult management strategies for clinically localized or advanced prostate cancer.

Keywords:

biomarkers, clinical practice, individualized medicine, personalized medicine, prostate cancer genetics

<u>Original Lay Summary</u>

The management of prostate cancer becomes more and more challenging mainly due to limitations of risk stratifications systems and its heterogenous clinical and biological behavior. Therefore, to increase detection rate of clinically significant prostate cancer and to assess the aggressiveness of the disease is a clinical priority in daily urology practice. Several biomarkers have been developed over the last decade to improve the accuracy of serum PSA and to help clinicians in difficult management scenarios. In this review article, we focus on the scientific evidence that supports the clinical use of the most robust and reliable biomarkers considered by professional urological societies (and included in uro-oncological guidelines) in the early diagnosis process and treatment decisions for clinically localized or advanced prostate cancer.

**Assessment Category**	**Score**	**Assessment Category**	**Score**	**Assessment Category**	**Score**
Comprehension Score Rater 1	5	Gunning Fog Score	25.6	Words	123
Comprehension Score Rater 2	5	SMOG Index	18.4	Complex words	41
Reading Age (years)	24.0	Coleman Liau Index	18.6	Percentage of complex words	33.33
Flesch-Kincaid Reading Ease	-1.8	Automated Readability Index	21.5	Average words per sentence	30.75
Flesch-Kincaid Grade Level	21.2	Sentences	4	Average syllables per word	2.1

<u>Lay Summary (Simple Prompt)</u>

Managing prostate cancer has become more complex due to the growing number of treatment options and the varying ways the disease progresses in different individuals. Current tools that help doctors make treatment decisions don't always account for these differences, which can affect patient outcomes. This review highlights the role of specific biological markers, known as biomarkers, that can provide valuable information about a patient's cancer. By using these biomarkers, doctors can make more personalized decisions, choosing treatments that are better suited to each patient's unique situation. We discuss the scientific evidence supporting the use of these biomarkers in clinical practice, as recommended by professional urology organizations. This approach aims to improve both the accuracy of prostate cancer diagnosis and the effectiveness of treatment plans, ultimately leading to better care for patients.

**Assessment Category**	**Score**	**Assessment Category**	**Score**	**Assessment Category**	**Score**
Comprehension Score Rater 1	5	Gunning Fog Score	17.6	Words	131
Comprehension Score Rater 2	5	SMOG Index	12.9	Complex words	30
Reading Age (years)	21.5	Coleman Liau Index	18.9	Percentage of complex words	22.9
Flesch-Kincaid Reading Ease	23.2	Automated Readability Index	17.3	Average words per sentence	21.83
Flesch-Kincaid Grade Level	15.4	Sentences	6	Average syllables per word	1.91

Lay Summary (Extended Prompt)

This study looks at how certain biomarkers (biological markers) can help doctors better understand and treat prostate cancer. Prostate cancer can behave very differently from one patient to another, which makes it hard to predict how well a treatment will work for each person. Although doctors have several ways to measure the risk of prostate cancer, these methods don't always give personalized answers for each patient. The study reviews the latest evidence on which biomarkers can be used to help doctors make better decisions about the best treatment for each patient, whether they have early-stage or advanced prostate cancer. By improving how doctors use these biomarkers, the hope is to provide more personalized care, ultimately improving survival rates and quality of life for patients.

**Assessment Category**	**Score**	**Assessment Category**	**Score**	**Assessment Category**	**Score**
Comprehension Score Rater 1	5	Gunning Fog Score	15.4	Words	125
Comprehension Score Rater 2	5	SMOG Index	10.7	Complex words	17
Reading Age (years)	19.5	Coleman Liau Index	15.3	Percentage of complex words	13.6
Flesch-Kincaid Reading Ease	42.7	Automated Readability Index	15.9	Average words per sentence	25.0
Flesch-Kincaid Grade Level	13.5	Sentences	5	Average syllables per word	1.64

Manuscript 200

Caggia S, Johnston A, Walunj DT, Moore AR, Peer BH, Everett RW, et al. Gαi2 Protein Inhibition Blocks Chemotherapy- and Anti-Androgen-Induced Prostate Cancer Cell Migration. Cancers [Internet]. 2024 Jan 10;16(2):296. Available from: http://dx.doi.org/10.3390/cancers16020296

Abstract

We have previously shown that heterotrimeric G-protein subunit alphai2 (Gαi2) is essential for cell migration and invasion in prostate, ovarian and breast cancer cells, and novel small molecule inhibitors targeting Gαi2 block its effects on migratory and invasive behavior. In this study, we have identified potent, metabolically stable, second generation Gαi2 inhibitors which inhibit cell migration in prostate cancer cells. Recent studies have shown that chemotherapy can induce the cancer cells to migrate to distant sites to form metastases. In the present study, we determined the effects of taxanes (docetaxel), anti-androgens (enzalutamide and bicalutamide) and histone deacetylase (HDAC) inhibitors (SAHA and SBI-I-19) on cell migration in prostate cancer cells. All treatments induced cell migration, and simultaneous treatments with new Gαi2 inhibitors blocked their effects on cell migration. We concluded that a combination treatment of Gαi2 inhibitors and chemotherapy could blunt the capability of cancer cells to migrate and form metastases.

Keywords:

Gαi2, HDACi, cancer, cell migration, chemotherapy, metastases

<u>Original Lay Summary</u>

Chemotherapy is the first line of treatment for malignant tumors. However, recent discoveries have shown that a relapse and the formation of metastatic disease could be facilitated by these treatments. In this study, we show how several chemotherapeutic drugs induced prostate cancer cells migration. Additionally we propose that combination therapy with newly synthesized inhibitors could block the chemotherapy-driven metastatic behavior of prostate cancer cells.

**Assessment Category**	**Score**	**Assessment Category**	**Score**	**Assessment Category**	**Score**
Comprehension Score Rater 1	4	Gunning Fog Score	17.6	Words	65
Comprehension Score Rater 2	4	SMOG Index	12.6	Complex words	19
Reading Age (years)	21.5	Coleman Liau Index	20.0	Percentage of complex words	29.23
Flesch-Kincaid Reading Ease	17.2	Automated Readability Index	15.3	Average words per sentence	16.25
Flesch-Kincaid Grade Level	14.9	Sentences	4	Average syllables per word	2.05

Lay Summary (Simple Prompt)

Prostate cancer treatments like chemotherapy and anti-androgens can unintentionally cause cancer cells to move to other parts of the body, leading to the spread of the disease (metastasis). This study focused on a specific protein, Gαi2, which plays a key role in helping cancer cells migrate. Researchers tested new drugs designed to block Gαi2 and found that these drugs successfully stopped cancer cells from moving, even when combined with treatments known to promote cell migration. The results suggest that adding Gαi2 inhibitors to prostate cancer therapy could help prevent the spread of cancer, potentially improving treatment outcomes and patient survival.

**Assessment Category**	**Score**	**Assessment Category**	**Score**	**Assessment Category**	**Score**
Comprehension Score Rater 1	5	Gunning Fog Score	15.2	Words	101
Comprehension Score Rater 2	5	SMOG Index	10.5	Complex words	13
Reading Age (years)	20.5	Coleman Liau Index	16.2	Percentage of complex words	12.87
Flesch-Kincaid Reading Ease	39.6	Automated Readability Index	16.8	Average words per sentence	25.25
Flesch-Kincaid Grade Level	14.0	Sentences	4	Average syllables per word	1.67

<u>Lay Summary (Extended Prompt)</u>

This study investigates how blocking a specific protein called Gαi2 can stop prostate cancer cells from spreading. Researchers have found that when prostate cancer cells are treated with chemotherapy or anti-hormone drugs, they can move to other parts of the body and form new tumors. The team tested new drugs that block Gαi2 and discovered they can prevent this movement. By using these drugs along with chemotherapy, they found that cancer cells were less likely to spread. This research is important because it shows a potential way to make cancer treatments more effective by stopping cancer cells from moving and forming new tumors, which could improve the chances of treating prostate cancer and other cancers in the future.

**Assessment Category**	**Score**	**Assessment Category**	**Score**	**Assessment Category**	**Score**
Comprehension Score Rater 1	5	Gunning Fog Score	12.9	Words	119
Comprehension Score Rater 2	5	SMOG Index	8.3	Complex words	10
Reading Age (years)	17.5	Coleman Liau Index	13.9	Percentage of complex words	8.4
Flesch-Kincaid Reading Ease	54.7	Automated Readability Index	14.3	Average words per sentence	23.8
Flesch-Kincaid Grade Level	11.5	Sentences	5	Average syllables per word	1.51

Manuscript 201

Zaalberg A, Pottendorfer E, Zwart W, Bergman AM. It Takes Two to Tango: The Interplay between Prostate Cancer and Its Microenvironment from an Epigenetic Perspective. Cancers [Internet]. 2024 Jan 10;16(2):294. Available from: http://dx.doi.org/
10.3390/cancers16020294

Abstract

Prostate cancer is the second most common cancer in men worldwide and is associated with high morbidity and mortality. Consequently, there is an urgent unmet need for novel treatment avenues. In addition to somatic genetic alterations, deviations in the epigenetic landscape of cancer cells and their tumor microenvironment (TME) are critical drivers of prostate cancer initiation and progression. Unlike genomic mutations, epigenetic modifications are potentially reversible. Therefore, the inhibition of aberrant epigenetic modifications represents an attractive and exciting novel treatment strategy for castration-resistant prostate cancer patients. Moreover, drugs targeting the epigenome also exhibit synergistic interactions with conventional therapeutics by directly enhancing their anti-tumorigenic properties by "priming" the tumor and tumor microenvironment to increase drug sensitivity. This review summarizes the major epigenetic alterations in prostate cancer and its TME, and their involvement in prostate tumorigenesis, and discusses the impact of epigenome-targeted therapies.

Keywords:

androgen receptor targeted therapy, epigenetics, immunotherapy, prostate cancer, tumor microenvironment

Original Lay Summary

Prostate cancer is a prevalent cancer in men. Metastatic disease is initially responsive to androgen receptor signaling inhibition, but eventually resistance develops despite continuation of therapy. This resistance involves multiple adaptations in the cell but also to the tumor microenvironment, including epigenetic alterations. Epigenetic alterations change gene expression without DNA sequence modifications and play a key role as regulators of cell functions both in the tumor and the tumor microenvironment. Moreover, these epigenetic alterations highlight potential therapeutic targets. Targeting these epigenetic modifications could improve androgen receptor-targeted therapy and enhance anti-tumor immunity. In this review we discuss the role of epigenetics in prostate cancer, strategies to target them, and their impact on the tumor microenvironment, with the goal of identifying novel therapeutic avenues for advanced prostate cancer.

**Assessment Category**	**Score**	**Assessment Category**	**Score**	**Assessment Category**	**Score**
Comprehension Score Rater 1	5	Gunning Fog Score	21.7	Words	128
Comprehension Score Rater 2	5	SMOG Index	14.8	Complex words	46
Reading Age (years)	24.0	Coleman Liau Index	21.3	Percentage of complex words	35.94
Flesch-Kincaid Reading Ease	0.6	Automated Readability Index	17.4	Average words per sentence	18.29
Flesch-Kincaid Grade Level	17.7	Sentences	7	Average syllables per word	2.22

<u>Lay Summary (Simple Prompt)</u>

Prostate cancer is one of the most common cancers in men and remains difficult to treat, especially in advanced stages. While genetic changes contribute to its growth, scientists have also discovered that changes in how genes are regulated—called "epigenetic modifications"—play a crucial role. Unlike genetic mutations, these modifications can potentially be reversed, making them an exciting target for new treatments. This study explores how prostate cancer interacts with its surrounding environment at an epigenetic level and highlights promising therapies that aim to correct these changes. Some of these treatments may also work together with existing cancer drugs to make them more effective. By understanding these mechanisms, researchers hope to develop better treatment options for patients with aggressive prostate cancer.

**Assessment Category**	**Score**	**Assessment Category**	**Score**	**Assessment Category**	**Score**
Comprehension Score Rater 1	5	Gunning Fog Score	16.7	Words	119
Comprehension Score Rater 2	5	SMOG Index	12.3	Complex words	27
Reading Age (years)	21.5	Coleman Liau Index	18.9	Percentage of complex words	22.69
Flesch-Kincaid Reading Ease	33.1	Automated Readability Index	16.2	Average words per sentence	19.83
Flesch-Kincaid Grade Level	13.6	Sentences	6	Average syllables per word	1.82

Lay Summary (Extended Prompt)

Prostate cancer is a common and serious illness in men, causing many health problems and deaths. This study explores how changes in the way genes are controlled, called epigenetics, contribute to the development and spread of prostate cancer. Unlike genetic mutations, which are often permanent, epigenetic changes can be reversed, offering new hope for treatment. The researchers focus on the cancer's environment, which includes surrounding cells and tissues, and how it can influence the cancer's behavior. They suggest that targeting epigenetic changes could improve treatments for patients whose cancer no longer responds to traditional therapies. This research could lead to better, more effective treatments, giving hope to those suffering from this difficult disease.

**Assessment Category**	**Score**	**Assessment Category**	**Score**	**Assessment Category**	**Score**
Comprehension Score Rater 1	5	Gunning Fog Score	14.6	Words	113
Comprehension Score Rater 2	5	SMOG Index	10.6	Complex words	20
Reading Age (years)	19.5	Coleman Liau Index	17.8	Percentage of complex words	17.7
Flesch-Kincaid Reading Ease	41.0	Automated Readability Index	14.9	Average words per sentence	18.83
Flesch-Kincaid Grade Level	12.2	Sentences	6	Average syllables per word	1.73

Manuscript 202

Baude J, Niogret J, Jacob P, Bardet F, Desmoulins I, Zanetta S, et al. Carboplatin and Etoposide for the Treatment of Metastatic Prostate Cancer with or without Neuroendocrine Features: A French Single-Center Experience. Cancers [Internet]. 2024 Jan 9;16(2):280. Available from: http://dx.doi.org/10.3390/cancers16020280

Abstract

Background: Chemotherapy using carboplatin and etoposide (CE) is frequently pragmatically proposed to treat metastatic prostate cancer (mPC), both primary small-cell neuroendocrine (PSC-NE) carcinoma and adenocarcinoma with or without neuroendocrine (NE) marker elevation. However, the real benefit of CE is poorly reported in the recent therapeutic context.

Methods: We retrospectively analyzed the efficacy and tolerance of CE chemotherapy in these three different groups of mPC patients. Efficacy endpoints included radiological response, progression-free survival (PFS), and overall survival (OS), as well as PSA response and PFS2/PFS1 ratio in patients with adenocarcinoma.

Results: Sixty-nine patients were included in this single-center study (N = 18 with PSC-NE carcinoma and 51 with adenocarcinoma with (N = 18) or without (N = 33) NE marker elevation). Patients with adenocarcinoma were highly pretreated with next-generation hormonal agents (NHAs) and taxanes. In patients with adenocarcinoma, a PSA response ≥50% was observed in six patients (15.8%), four of whom had NE marker elevation. The radiological response was higher in PSC-NE and tended to be higher in adenocarcinoma when NE marker elevation was present. Comparing patients with adenocarcinoma with vs. without NE marker elevation, the median PFS was 3.7 and 2.1 months and the median OS was 7.7 and 4.7 months, respectively. Overall, 62.3% of patients experienced grade 3-4 adverse events (mainly hematological), and three treatment-related deaths were recorded.

Conclusion: Reports of the clinical results of CE suggest that we should not mix PSC-NE and castration-resistant adenocarcinoma of the prostate. In patients with heavily pretreated adenocarcinoma, the benefit/risk ratio of CE chemotherapy seems unfavorable due to poor response and high toxicity.

Keywords:

NSE, PSA, anaplastic, carboplatin, chromogranin, etoposide, neuroendocrine, prostate cancer, survival

<u>Original Lay Summary</u>

Chemotherapy using carboplatin and etoposide (CE) is often proposed to treat metastatic prostate cancer (mPC), both primary small-cell neuroendocrine (PSC-NE) and adenocarcinoma. We aimed to investigate the benefit of CE, especially in patients with heavily pretreated adenocarcinoma. In this retrospective series, we showed that the reports of clinical results of CE indicate that we should not mix PSC-NE and adenocarcinoma. In patients with PSC-NE, 58.8% had a radiological response to CE and median progression-free survival was 7.9 months. In pretreated patients with adenocarcinoma, the benefit/risk ratio of CE seems unfavorable with poor response and high toxicity irrespective of the elevation of neuroendocrine markers.

**Assessment Category**	**Score**	**Assessment Category**	**Score**	**Assessment Category**	**Score**
Comprehension Score Rater 1	5	Gunning Fog Score	17.6	Words	110
Comprehension Score Rater 2	5	SMOG Index	12.4	Complex words	32
Reading Age (years)	19.5	Coleman Liau Index	16.1	Percentage of complex words	29.09
Flesch-Kincaid Reading Ease	20.9	Automated Readability Index	12.0	Average words per sentence	15.71
Flesch-Kincaid Grade Level	14.2	Sentences	7	Average syllables per word	2.01

Lay Summary (Simple Prompt)

This study looked at how effective and safe a combination of two chemotherapy drugs, carboplatin and etoposide, is for treating men with advanced prostate cancer. The research included 69 patients from a single center in France, some with a rare type of prostate cancer called small-cell neuroendocrine carcinoma and others with typical prostate cancer, with or without signs of neuroendocrine activity. The results showed that patients with neuroendocrine features responded slightly better to the treatment, but overall, the benefits were limited. Many patients experienced serious side effects, and three died due to treatment complications. The study suggests that this chemotherapy may not be a good option for patients with heavily treated prostate cancer because the risks often outweigh the benefits. This highlights the need for more effective and safer treatment options for these patients.

**Assessment Category**	**Score**	**Assessment Category**	**Score**	**Assessment Category**	**Score**
Comprehension Score Rater 1	3	Gunning Fog Score	15.2	Words	135
Comprehension Score Rater 2	3	SMOG Index	10.8	Complex words	21
Reading Age (years)	19.5	Coleman Liau Index	17.0	Percentage of complex words	15.56
Flesch-Kincaid Reading Ease	39.9	Automated Readability Index	16.0	Average words per sentence	22.5
Flesch-Kincaid Grade Level	13.3	Sentences	6	Average syllables per word	1.7

Lay Summary (Extended Prompt)

This study looks at the use of two chemotherapy drugs, carboplatin and etoposide, to treat metastatic prostate cancer. Prostate cancer can sometimes spread to other parts of the body, and in some cases, it may have special features that affect treatment. The researchers studied the treatment's effectiveness in three groups of patients: those with a rare form of prostate cancer called small-cell neuroendocrine carcinoma, and those with more common types of prostate cancer, some of which showed markers suggesting neuroendocrine features. The results showed that chemotherapy worked better for the rare form of cancer, and that patients with certain markers had a better response. However, the treatment caused serious side effects in many patients, making it less suitable for those who had already been treated with other therapies. These findings help guide future treatment choices for prostate cancer patients.

**Assessment Category**	**Score**	**Assessment Category**	**Score**	**Assessment Category**	**Score**
Comprehension Score Rater 1	4	Gunning Fog Score	14.5	Words	140
Comprehension Score Rater 2	4	SMOG Index	10.1	Complex words	18
Reading Age (years)	19.5	Coleman Liau Index	16.2	Percentage of complex words	12.86
Flesch-Kincaid Reading Ease	45.4	Automated Readability Index	15.9	Average words per sentence	23.33
Flesch-Kincaid Grade Level	12.7	Sentences	6	Average syllables per word	1.63

Manuscript 203

Barashi NS, Li T, Angappulige DH, Zhang B, O'Gorman H, Nottingham CU, et al. Symptomatic Benign Prostatic Hyperplasia with Suppressed Epigenetic Regulator HOXB13 Shows a Lower Incidence of Prostate Cancer Development. Cancers [Internet]. 2024 Jan 2;16(1):213. Available from: http://dx.doi.org/10.3390/cancers16010213

Abstract

Our objective was to identify variations in gene expression that could help elucidate the pathways for the development of prostate cancer (PCa) in men with Benign Prostatic Hyperplasia (BPH). We included 98 men with BPH, a positive prostate MRI (Prostate Imaging Reporting and Data System; PIRADS ≥ 4), and a negative biopsy from November 2014 to January 2018. RNA sequencing (RNA-Seq) was performed on tissue cores from the MRI lesion and a geographically distant region (two regions per patient). All patients were followed for at least three years to identify who went on to develop PCa. We compared the gene expressions of those who did not develop PCa ("BPH-only") vs. those who did ("BPH/PCa"). Then, we identified the subset of men with BPH who had the highest American Urological Association (AUA) symptom scores ("symptomatic BPH") and compared their gene expression to the BPH/PCa group. At a median follow-up of 47.5 months, 15 men had developed PCa while 83 did not. We compared gene expressions of 14 men with symptomatic BPH (AUAss ≥ 18) vs. 15 with BPH/PCa. We found two clusters of genes, suggesting the two groups had distinctive molecular features. Differential analysis revealed genes that were upregulated in BPH-only and downregulated in BPH/PCa, and vice versa. Symptomatic BPH men had upregulation of T-cell activation markers (TCR, CD3, ZAP70, IL-2 and IFN-γ and chemokine receptors, CXCL9/10) expression. In contrast, men with BPH/PCa had upregulation of NKX3-1 and HOXB13 transcription factors associated with luminal epithelial progenitors but depleted of immune cells, suggesting a cell-autonomous role in immune evasion. Symptomatic BPH with immune-enriched landscapes may support anti-tumor immunity. RNA sequencing of

benign prostate biopsy tissue showing upregulation of NKX3-1 and HOXB13 with the absence of T-cells might help in identifying men at higher risk of future PCa development, which may be useful in determining ongoing PCa screening.

Keywords:

HOXB13, RNA, epigenetics, prostate cancer, prostatic hyperplasia

<u>Original Lay Summary</u>

Patients with symptomatic Benign Prostatic Hyperplasia (BPH) showed upregulated gene expression of biological pathways associated with T cell activation and suppression of a key transcription factor HOXB13, which is associated with transcription and epigenetic regulation of prostate cancer (PCa). In contrast, patients with BPH who later developed PCa showed significantly reduced inflammation and revealed activation of several transcription factors related to PCa, including HOXB13, AR, FOXA1 and SIM2. It may be clinically beneficial for urologists to be able to distinguish between men with BPH who are at a higher risk of developing PCa in the future based on their molecular subtype.

**Assessment Category**	**Score**	**Assessment Category**	**Score**	**Assessment Category**	**Score**
Comprehension Score Rater 1	4	Gunning Fog Score	23.8	Words	101
Comprehension Score Rater 2	4	SMOG Index	17.5	Complex words	28
Reading Age (years)	24.0	Coleman Liau Index	17.5	Percentage of complex words	27.72
Flesch-Kincaid Reading Ease	7.7	Automated Readability Index	22.1	Average words per sentence	33.67
Flesch-Kincaid Grade Level	20.6	Sentences	3	Average syllables per word	1.95

<u>Lay Summary (Simple Prompt)</u>

This study explored why some men with benign prostatic hyperplasia (BPH)—a common condition causing an enlarged prostate—develop prostate cancer while others do not. Researchers analyzed prostate tissue samples from 98 men with BPH who had suspicious MRI results but no cancer detected on biopsy. They tracked these men for over three years to see who later developed prostate cancer. By comparing gene activity in men who remained cancer-free to those who developed cancer, they found key differences. Men with severe BPH symptoms showed signs of an active immune response in their prostate tissue, which may help protect against cancer. In contrast, those who developed prostate cancer had genes linked to immune system evasion. These findings suggest that analyzing gene patterns in prostate tissue could help predict future cancer risk, potentially guiding more personalized screening and prevention strategies.

**Assessment Category**	**Score**	**Assessment Category**	**Score**	**Assessment Category**	**Score**
Comprehension Score Rater 1	5	Gunning Fog Score	14.3	Words	139
Comprehension Score Rater 2	5	SMOG Index	10.3	Complex words	22
Reading Age (years)	18.5	Coleman Liau Index	16.4	Percentage of complex words	15.83
Flesch-Kincaid Reading Ease	44.9	Automated Readability Index	14.3	Average words per sentence	19.86
Flesch-Kincaid Grade Level	11.9	Sentences	7	Average syllables per word	1.68

Lay Summary (Extended Prompt)

This study looked at how certain genes might affect whether men with an enlarged prostate (a condition known as Benign Prostatic Hyperplasia, or BPH) will develop prostate cancer. The researchers studied 98 men with BPH who had a positive prostate scan but a negative biopsy. Over about four years, they tracked who developed prostate cancer. They compared the gene activity in men who developed cancer to those who did not. The study found that men with more severe BPH symptoms had different gene activity compared to those who developed prostate cancer. The researchers discovered that certain genes related to immune system activity were higher in men with severe BPH, which might help protect against cancer. These findings could help doctors predict which men with BPH might need more attention for prostate cancer screening in the future.

**Assessment Category**	**Score**	**Assessment Category**	**Score**	**Assessment Category**	**Score**
Comprehension Score Rater 1	5	Gunning Fog Score	12.8	Words	136
Comprehension Score Rater 2	5	SMOG Index	9.6	Complex words	19
Reading Age (years)	17.5	Coleman Liau Index	14.3	Percentage of complex words	13.97
Flesch-Kincaid Reading Ease	55.9	Automated Readability Index	12.4	Average words per sentence	19.43
Flesch-Kincaid Grade Level	10.3	Sentences	7	Average syllables per word	1.55

Manuscript 204

Nicoletti G, Mazzetti S, Maimone G, Cignini V, Cuocolo R, Faletti R, et al. Development and Validation of an Explainable Radiomics Model to Predict High-Aggressive Prostate Cancer: A Multicenter Radiomics Study Based on Biparametric MRI. Cancers [Internet]. 2024 Jan 1;16(1):203. Available from: http://dx.doi.org/10.3390/cancers16010203

Abstract

In the last years, several studies demonstrated that low-aggressive (Grade Group (GG) ≤ 2) and high-aggressive (GG ≥ 3) prostate cancers (PCas) have different prognoses and mortality. Therefore, the aim of this study was to develop and externally validate a radiomic model to noninvasively classify low-aggressive and high-aggressive PCas based on biparametric magnetic resonance imaging (bpMRI). To this end, 283 patients were retrospectively enrolled from four centers. Features were extracted from apparent diffusion coefficient (ADC) maps and T2-weighted (T2w) sequences. A cross-validation (CV) strategy was adopted to assess the robustness of several classifiers using two out of the four centers. Then, the best classifier was externally validated using the other two centers. An explanation for the final radiomics signature was provided through Shapley additive explanation (SHAP) values and partial dependence plots (PDP). The best combination was a naïve Bayes classifier trained with ten features that reached promising results, i.e., an area under the receiver operating characteristic (ROC) curve (AUC) of 0.75 and 0.73 in the construction and external validation set, respectively. The findings of our work suggest that our radiomics model could help distinguish between low- and high-aggressive PCa. This noninvasive approach, if further validated and integrated into a clinical decision support system able to automatically detect PCa, could help clinicians managing men with suspicion of PCa.

Keywords:

explainable artificial intelligence, feature extraction, magnetic resonance imaging, prostate cancer, radiomics, tumor aggressiveness

<u>Original Lay Summary</u>

Prostate cancer (PCa) is one of the leading causes of mortality for men worldwide. PCa aggressiveness affects the patient's prognosis, with less aggressive tumors, i.e., Grade Group (GG) 1 and 2, having lower mortality and better outcomes. For this reason, the aim of this study is to distinguish between GG ≤ 2 and ≥3 PCa using an automatic and noninvasive approach based on artificial intelligence methods. The results obtained are promising, as the system achieved robust results on a multicenter external dataset. If further validated, this approach, combined with the expert knowledge of urologists, could help identify PCa patients who have a better prognosis and may benefit from less invasive treatments.

**Assessment Category**	**Score**	**Assessment Category**	**Score**	**Assessment Category**	**Score**
Comprehension Score Rater 1	3	Gunning Fog Score	13.5	Words	112
Comprehension Score Rater 2	3	SMOG Index	9.8	Complex words	20
Reading Age (years)	17.5	Coleman Liau Index	14.4	Percentage of complex words	17.86
Flesch-Kincaid Reading Ease	43.3	Automated Readability Index	10.8	Average words per sentence	16.0
Flesch-Kincaid Grade Level	11.2	Sentences	7	Average syllables per word	1.74

Lay Summary (Simple Prompt)

Prostate cancer can range from less aggressive to highly aggressive forms, which affect a man's health differently. Accurately identifying the cancer's aggressiveness helps doctors choose the best treatment. In this study, researchers developed and tested a new, noninvasive model using MRI scans to predict whether prostate cancer is low- or high-aggressive. They analyzed MRI images from 283 men treated at four different medical centers, focusing on specific patterns in the scans. The model used advanced computer techniques to identify key features linked to cancer aggressiveness. It successfully distinguished between low- and high-aggressive prostate cancers with good accuracy. Importantly, the model explains how it makes decisions, making it easier for doctors to trust and use in clinical practice. If further validated, this approach could support doctors in diagnosing prostate cancer more accurately and planning better, personalized treatments for their patients.

**Assessment Category**	**Score**	**Assessment Category**	**Score**	**Assessment Category**	**Score**
Comprehension Score Rater 1	4	Gunning Fog Score	15.8	Words	141
Comprehension Score Rater 2	4	SMOG Index	11.4	Complex words	31
Reading Age (years)	20.5	Coleman Liau Index	18.3	Percentage of complex words	21.99
Flesch-Kincaid Reading Ease	34.1	Automated Readability Index	14.6	Average words per sentence	17.63
Flesch-Kincaid Grade Level	12.9	Sentences	8	Average syllables per word	1.83

Lay Summary (Extended Prompt)

This study focuses on developing a new tool to help doctors better understand prostate cancer and predict whether it is likely to be aggressive or not. The researchers used special MRI scans to analyze prostate cancer in 283 patients from four hospitals. They created a model that looks at certain features in the MRI images to identify aggressive cancers. The model showed good results in testing, with a score of 0.75 for its ability to correctly tell apart low- and high-aggressive cancer. The goal is to help doctors make quicker, more accurate decisions without needing invasive procedures, which could improve treatment and outcomes for patients. If this tool is further tested and used in hospitals, it could become an important part of helping doctors manage prostate cancer more effectively.

**Assessment Category**	**Score**	**Assessment Category**	**Score**	**Assessment Category**	**Score**
Comprehension Score Rater 1	4	Gunning Fog Score	13.0	Words	131
Comprehension Score Rater 2	4	SMOG Index	9.3	Complex words	18
Reading Age (years)	17.5	Coleman Liau Index	13.5	Percentage of complex words	13.74
Flesch-Kincaid Reading Ease	53.5	Automated Readability Index	11.4	Average words per sentence	18.71
Flesch-Kincaid Grade Level	10.4	Sentences	7	Average syllables per word	1.59
